# Genome-wide DNA methylation changes in CD19^+^ B cells from relapsing-remitting multiple sclerosis patients

**DOI:** 10.1038/s41598-018-35603-0

**Published:** 2018-11-27

**Authors:** Vicki E. Maltby, Rodney A. Lea, Moira C. Graves, Katherine A. Sanders, Miles C. Benton, Lotti Tajouri, Rodney J. Scott, Jeannette Lechner-Scott

**Affiliations:** 1 School of Medicine and Public Health, University of Newcastle, Newcastle, Australia; 2 Centre for Information Based Medicine, Hunter Medical Research Institute, Newcastle, Australia; 3 Institute of Health and Biomedical Innovation, Queensland University of Technology, Brisbane, Australia; 4 Faculty of Health Sciences and Medicine, Bond University, Gold Coast, Australia; 5 School of Biomedical Sciences and Pharmacy, University of Newcastle, Newcastle, Australia; 6 Medical Genetics, Pathology North, John Hunter Hospital, Newcastle, Australia; 7 Department of Neurology, John Hunter Hospital, Newcastle, Australia

## Abstract

Multiple Sclerosis (MS) is an inflammatory and neurodegenerative disease of the central nervous system. The inflammatory process in MS is driven by both T and B cells and current therapies are targeted to each of these cell types. Epigenetic mechanisms may provide a valuable link between genes and environment. DNA methylation is the best studied epigenetic mechanism and is recognized as a potential contributor to MS risk. The objective of this study was to identify DNA methylation changes associated with MS in CD19^+^ B-cells. We performed an epigenome-wide association analysis of DNA methylation in the CD19^+^ B-cells from 24 patients with relapsing-remitting MS on various treatments and 24 healthy controls using Illumina 450 K arrays. A large differentially methylated region (DMR) was observed at the lymphotoxin alpha (*LTA*) locus. This region was hypermethylated and contains 19 differentially methylated positions (DMPs) spanning 860 bp, all of which are located within the transcriptional start site. We also observed smaller DMRs at 4 MS-associated genes: *SLC44A2*, *LTBR*, *CARD11* and *CXCR5*. These preliminary findings suggest that B-cell specific DNA-methylation may be associated with MS risk or response to therapy, specifically at the *LTA* locus. Development of B-cell specific epigenetic therapies is an attractive new avenue of research in MS treatment. Further studies are now required to validate these findings and understand their functional significance.

## Introduction

Multiple Sclerosis is an inflammatory and neurogenerative disease leading to demyelination and axonal loss. Risk of developing MS is thought to be influenced by both genetic and environmental factors. The primary environmental factors that influence disease pathology are sunlight exposure, Epstein-Barr virus (EBV) infection and smoking^1^. Genome wide association studies (GWAS) have identified 149 genes associated with MS risk with approximately one third coming from variations in the major histocompatibility complex (MHC)^2,3^. Despite this, there still remains a large element of unexplained heritability in terms of disease pathology.

Epigenetic mechanisms are capable of modifying the genome without changes to the DNA sequence and can be inherited. One well-studied epigenetic mechanism is DNA methylation, which is the addition of a methyl group to CpG dinucleotides. We, and others, have used genome-wide DNA methylation technologies to identify differentially methylated positions (DMPs) in the T-cells of MS patients compared to healthy controls^4–8^. In two independent studies of CD4^+^ T-cells, we found a striking differentially methylated region (DMR) located within the major histocompatibility complex (MHC) region, with a major peak at *HLA-DRB1* and *RNF39*^4,6^. Using the same cohort of patients we assessed DMPs in CD8^+^ T-cells and found 79 DMPs, all of which showed minor association with MS but none of which overlapped with any of the DMPs found in CD4^+^ T-cells^5^. A study by Bos *et al*. also found little overlap between the methylation profiles of CD4^+^ and CD8^+^ T-cells, highlighting the importance of investigating individual cell subtypes.

There is evidence to suggest that T-cells may have a role in MS pathology (reviewed in Martin *et al*.^9^). However, it is becoming increasingly clear that B-cells may also play a substantial role in helping to drive disease. Activated B-cells may contribute to MS pathology as antibody producing cells, antigen presenting cells or as a source of pro-inflammatory cytokines (reviewed in Lehmann-Horn *et al*.^10^). Evidence for this is in the success of B-cell depleting monoclonal antibodies, such as rituximab^11^ and ocrelizumab^12^ as MS therapies. Additionally, many currently approved MS therapies, for example fingolimod and dimethyl fumarate, also have an impact on B cells through reduced numbers or a shift in phenotype towards a more anti-inflammatory cytokine profile (reviewed in Lehmann-Horn *et al*.^10^).

In an effort to identify B-cell specific DMPs associated in MS, we performed genome-wide DNA methylation study of CD19^+^ B-cells from MS patients and healthy controls. We used the same cohort and data analysis techniques as our previous studies so that the results could be compared to those from the CD4^+^ and CD8^+^ T-cells.

## Methods and Materials

### Ethics Statement

The Hunter New England Health Research Ethics Committee and University of Newcastle Ethics committee approved this study (05/04/13.09 and H-505-0607 respectively), and methods were carried out in accordance with institutional guidelines on human subject experiments. Written and informed consent was obtained from all patient and control subjects. MS patients gave written and verbal consent. The Australian Red Cross Blood Service ethics committee approved the use of blood from healthy donors.

### Sample Processing

We performed an epigenome-wide association study (EWAS) of CD19^+^ B-cells using the same patient cohort, work flow and data analysis as described in our previous study^4^. Briefly, whole blood was collected from 24 RRMS patients and 24 healthy controls. All patients were diagnosed with RRMS according to the McDonald criteria^13^. PBMCs were isolated from 45 mL of whole blood by density gradient centrifugation on lymphoprep (StemCell Technologies, Canada). CD19^+^ B cells were isolated using positive selection, magnetic separation kits (Stem Cell Technologies, Canada) according to the manufacturer’s protocols. Purity was assessed using FITC conjugated anti-CD19 antibody (clone H1B19, catalog #60005FL.1, StemCell Technologies, Canada) and the FACS CantoII flow cytometer (BD Biosciences, USA) and analyzed using the FACSDiva software (BD Biosciences, USA). All samples met a minimum purity threshold of ≥90%. DNA was extracted using the QiaAMP DNA micro kit (Qiagen, USA). DNA was then bisulphite converted and hybridized to Illumina 450 K arrays (service provided by the Australian Genome Research Facility).

### Data analysis

Raw fluorescence data were processed using a combination of R/Bioconductor and custom scripts. Raw data was parsed into the Bioconductor MINFI package. Methylation data was background corrected and quantile normalized according to MINFI routines. Data was cleaned by removing control probes, probes which map multiple times to the genome, cross-reactive probes and failed probes for which the intensity of both the methylated and the unmethylated probes was <1000 units across all samples. A threshold of 1000 units was selected based on the profile of the available negative control probes. Y chromosome probes were filtered out. All probe sequences were mapped to the human genome (buildHg19) using BOWTIE^14^ to identify potential hybridization anomalies. We chose to retain probes containing single nucleotide polymorphisms (SNPs) and filter these out post hoc where appropriate (see results section).

Measures of methylation (β values) were produced for each probe and ranged from completely unmethylated (β = 0) to completely methylated (β = 1). To identify differentially methylated positions (DMPs) associated with MS subtypes in this cohort, we first calculated the difference in median β value by subtracting the median β value of controls (median_cont_) from the median β value for cases (median_case_). This produced a Δ_meth_ score ranging from −1 (hypomethylated) to 1 (hypermethylated). A two-sample Kolmogorov-Smirnov test (K-S test) was used to determine if Δ_meth_ was statistically significant. A K-S test was chosen over the F test because of the marked variation in the distribution of the β values among the probes. Rather than base our CpG selection strictly on statistical significance (P-values) of the K-S test, which is overly limiting due to the small sample size and could miss important signal, we used a selection strategy based on a combination of P-value and effect size (ie. Δ_meth_ score). We have used this approach successfully in previous studies to implicate differential methylation at HLA in CD4+ cells with regard to MS^4–6^. A CpG was considered a DMP if the P-value was <0.05 and the absolute β value was >±0.1. Differentially methylated regions (DMRs) were called if at least two DMPs were found within a 500 base pair (bp) span of each other and were altered in the same direction (either all hypermethylated or all hypomethylated).

Over-Representation Analysis (ORA): To assess the biological relevance of DMPs in terms of MS pathology we conducted an ORA on resultant the DMP list using the WebGestalt engine (www.webgestalt.org) incorporating the KEGG pathways database.

## Results

### DMP and DMR analyses

**Figure 1:**
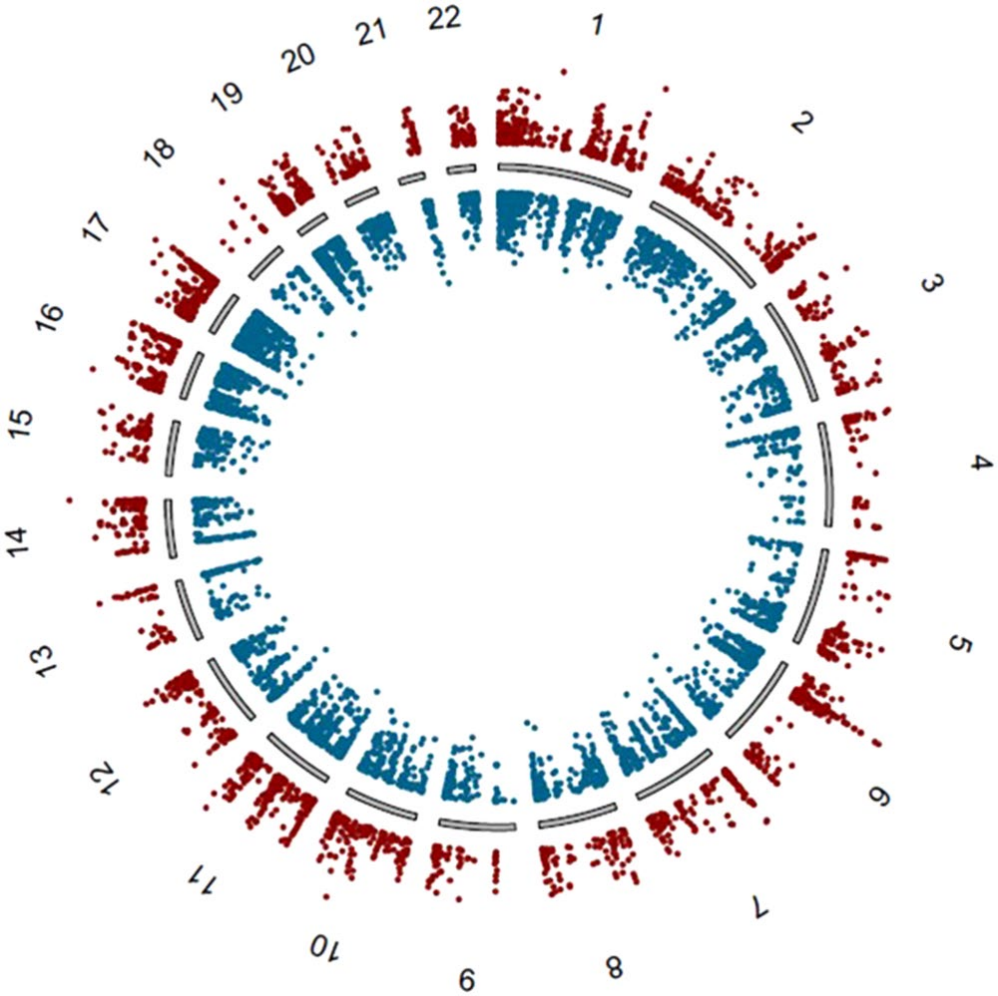
A genome-wide differential methylation plot. Data points outside the circle (red) represent increased methylation (i.e. ∆_meth_)_,_ in multiple sclerosis (MS) patients compared to controls whereas points inside the circle (blue) represent decreased methylation in MS patients compared to healthy controls.

**Figure 2:**
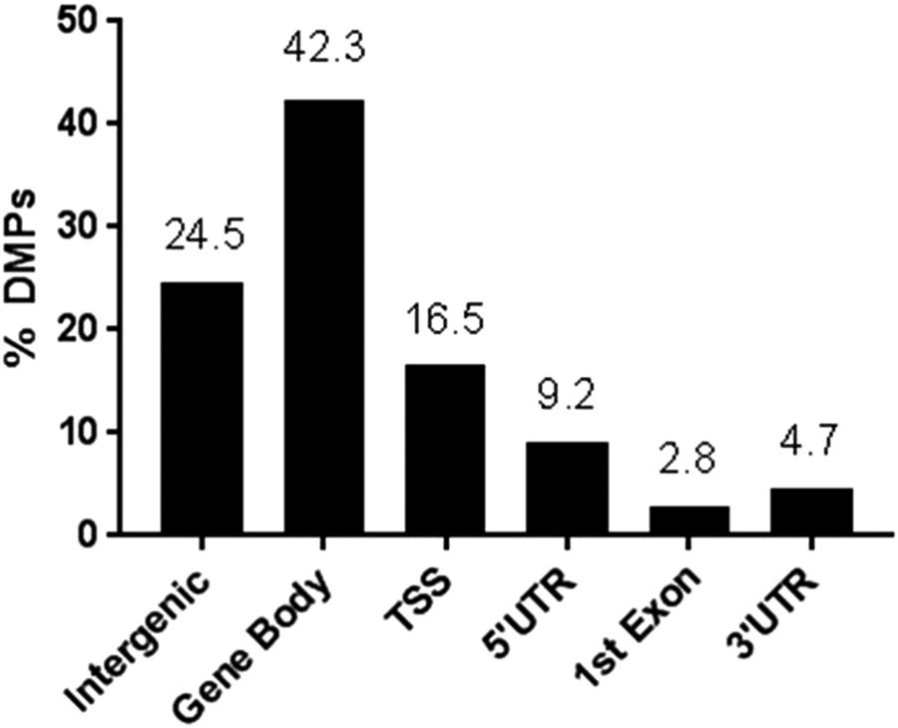
Distribution of DMPs over each of the genomic regions Y-axis represents proportion of total DMPs (7618) in each category (shown as percentage).

Table 1 shows the patient demographic for 24 MS patients and 24 healthy controls (Table 1). A total of 7618 CpGs met the criteria for a DMP (Table S1). Figure 1 shows the genome-wide distribution of differential methylation (Δ_meth_) for all DMPs. Amongst the DMPs, we observe an overall hypomethylation in MS cases, with 4731 (62%) of DMPs being hypomethylated and 2887 (38%) being hypermethylated in MS patients versus controls. When we considered genomic features for all DMPs we found 1869 (24.5%) map to intergenic regions, 3226 (42.3%) within the gene body, 1254 at the transcriptional start site (TSS1500 or TSS200) (16.5%), 699 in the 5′ untranslated region (UTR) (9.2%), 211 map to the 1^st^ exon (2.8%) and 359 in the 3′UTR (4.7%) (Fig. 2).

**Table 1:** Subject demographics.

Characteristic	MS participant (n = 24)	Control (n = 24)
Age range (yrs)	40.7 ± 8.5	43.3 ± 16.4
EDSS	2.4 ± 1.3	
Disease duration (yrs)	9.3 ± 6.6	
Treatment (n)		
• Naïve	1	
• OFF > 3 months	4	
• Interferon beta-1b	2	
• Interferon beta-1a	3	
• Glatiramer acetate	2	
• Natalizumab	4	
• Fingolimod	8

DMPs were ranked by Δ_meth_ values. The two top ranked DMPs were located within the lymphotoxin alpha (*LTA*) gene (alias: tumor necrosis factor beta, *TNF*β - hereafter referred to as *LTA*). These two sites had Δmeth values of 0.504 and 0.486 (50.4% and 48.6% hypermethylated respectively) in the MS patient group compared to the control group (P < 0.0001). Of the CpGs that met the criteria for a DMP, 19 are found in the *LTA* locus within a region of 860 bp. All sites are hypermethylated with Δmeth scores between 0.15 and 0.5 (between 15% and 50%) and are located within the TSS/5′UTR (Table 2).

**Table 2:** DMR at *LTA*.

IlmnID	MAPINFO	Element	Gene	Probe SNP	Probe SNPs10	mean MS	mean HC	Δmeth	P value
cg10995925	31539601	TSS1500	LTA			0.68	0.50	0.18	4.06E-04
cg14441276	31539735	TSS1500;TSS200	LTA		rs56161754	0.59	0.20	0.39	4.06E-04
cg09621572	31539973	TSS200;1stExon;5′UTR	LTA	rs36221306	rs56018225	0.66	0.24	0.42	4.57E-06
cg14437551	31539986	TSS200;1stExon;5′UTR	LTA	rs36221306		0.70	0.19	0.50	1.04E-04
cg14597739	31539998	TSS200;1stExon;5′UTR	LTA	rs56207507	rs36221306	0.74	0.25	0.49	1.04E-04
cg16219283	31540002	TSS200;1stExon;5′UTR	LTA	rs56207507		0.72	0.30	0.42	1.04E-04
cg21999229	31540014	TSS200;1stExon;5′UTR	LTA	rs56207507		0.65	0.27	0.39	2.34E-05
cg17169196	31540026	TSS200;1stExon;5′UTR	LTA	rs36221309	rs56207507	0.71	0.36	0.35	2.34E-05
cg02402436	31540051	TSS200;1stExon;5′UTR	LTA	rs36221309		0.47	0.18	0.29	4.57E-06
cg09736959	31540114	5′UTR	LTA	rs2239704		0.62	0.33	0.29	1.40E-03
cg24216966	31540121	5′UTR	LTA	rs2239704		0.73	0.38	0.35	1.04E-04
cg11586857	31540136	5′UTR	LTA	rs56245447	rs2239704	0.75	0.45	0.30	1.04E-04
cg10476003	31540169	5′UTR	LTA		rs56245447	0.55	0.29	0.25	4.57E-06
cg01157951	31540399	5′UTR	LTA			0.42	0.23	0.19	2.34E-05
cg22318806	31540411	5′UTR	LTA	rs4986978		0.41	0.23	0.18	4.06E-04
cg13815684	31540440	5′UTR	LTA			0.73	0.42	0.30	1.04E-04
cg17709873	31540456	5′UTR	LTA			0.53	0.38	0.15	4.32E-03
cg16280132	31540459	5′UTR	LTA			0.49	0.31	0.18	1.04E-04
cg26348243	31540461	5′UTR	LTA			0.47	0.20	0.28	2.34E-05

IlmnID = Illumina ID; MapINFO = genomic coordinates (Hg19); Element from UCSC RefGene; Probe SNP; Probe SNPs10.

### Genetic influence at the LTA locus

One technical limitation of array technology is the influence that SNPs may have on the calculated methylation levels (β values). Of the 19 DMPs identified at the *LTA* TSS, 13 of the corresponding probes contain an adjacent SNP which may potentially influence the methylation profile (Table 2). Rather than remove these sites from our analysis, we assessed the genetic influence on the methylation signal by visualizing the distribution of β values.

**Figure 3:**
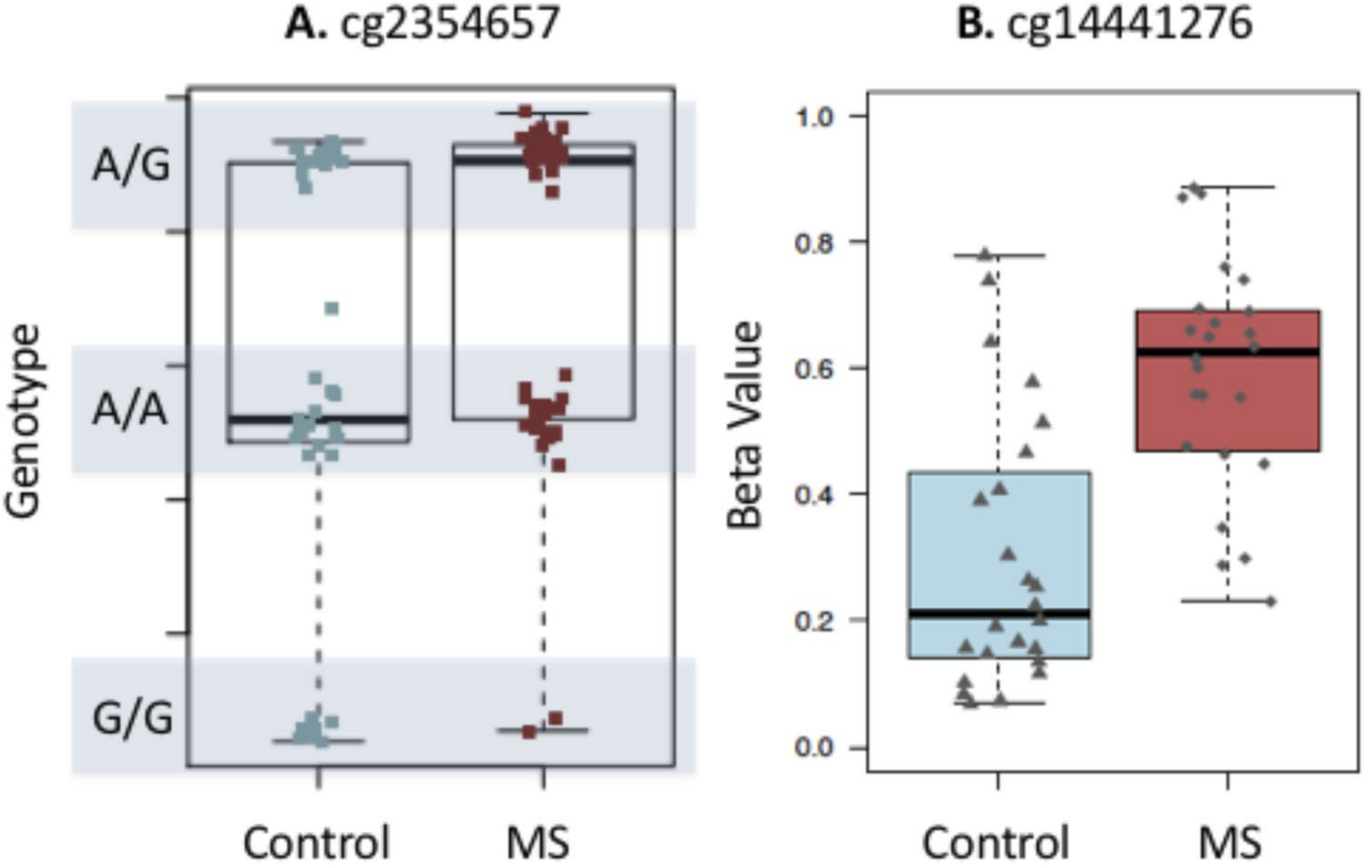
Tukey box plot showing distribution of beta values for (**A**) a probe where the SNP is driving the methylation values and (**B**) the top LTA site from this study. The box plot shows the data within the interquartile range and the median is represented by a solid black line. Whiskers show maximum and minimum values. Grey bars indicate region for each genotype (homozygous allele 1 (1/1), heterozygous (1/2), and homozygous allele 2 (2/2)). Each point represents either an individual control (blue) or MS patients (red). Y axis shows β values.

**Figure 4:**
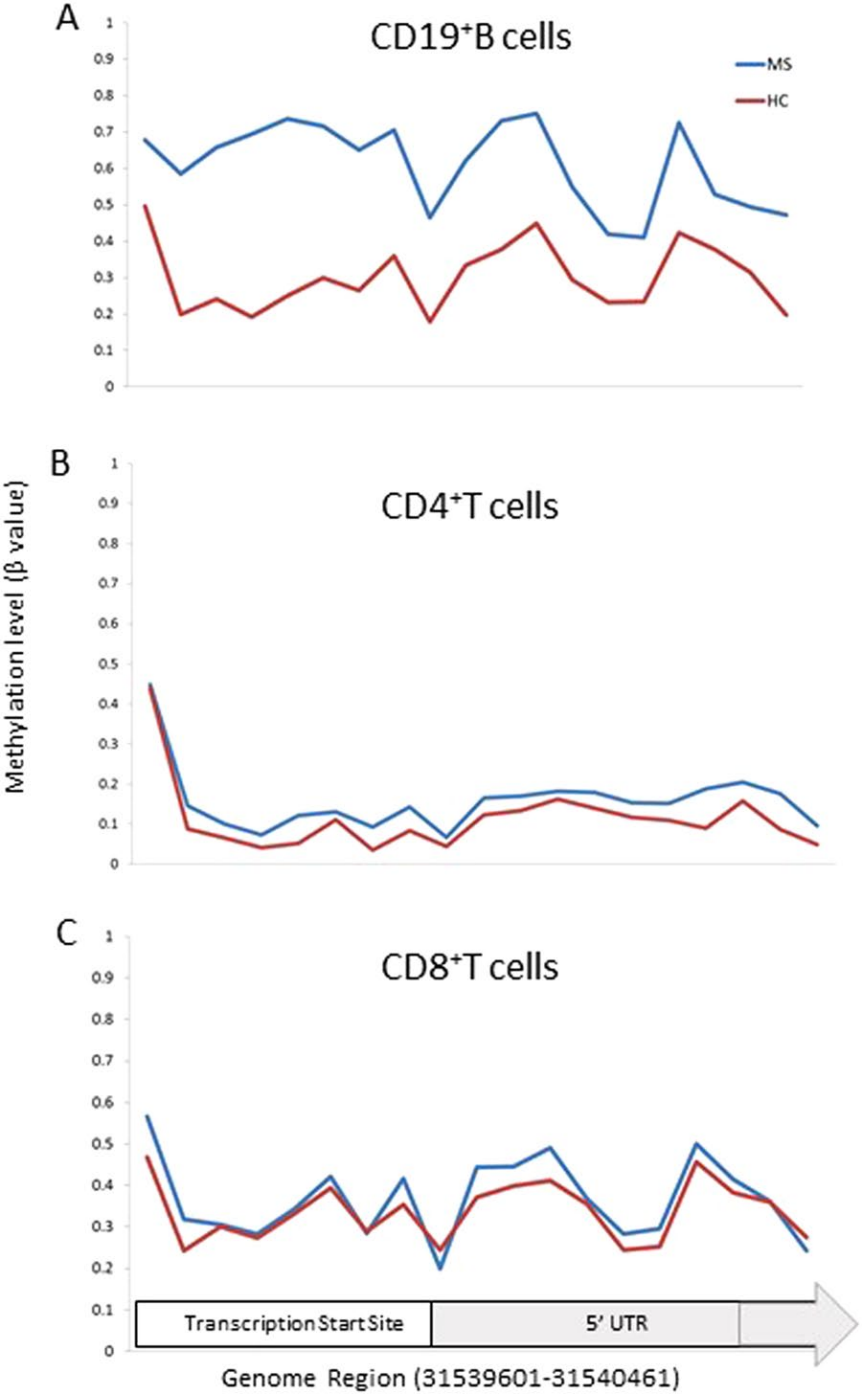
DMPs within the LTA TSS/5′UTR region Line graph showing the methylation level (β value) of MS cases (blue) versus controls (red) for the genomic region 31539601-31540461 for (**A**) CD19^+^ B cells (**B**) CD4^+^ T cells^4,6^ and (**C**) CD8^+^ T cells^5^. The region covers 19 probes contained within the *LTA* gene.

Figure 3A shows an example of a CpG site whose methylation signal is known to be influenced by a SNP located at this probe. This example shows that the β values cluster into 3 distinct regions representing the 3 possible genotypes (homozygous allele 1, homozygous allele 2 or heterozygous). Figure 3B shows the influence of SNPs on the top *LTA* CpG site in our DMR. The β values form a uniform spread, providing support that the SNP is not influencing the methylation signal at this particular CpG site. A similar result is seen in all DMPs within the *LTA* cluster (data not show). In addition to this, we compared the profiles of *LTA* in CD4^+^ and CD8^+^ T-cells from our previous data sets with the same cohort. We found that hypermethylation at the *LTA* TSS appears to be specific to CD19^+^ B-cells, providing further support that the methylation effects observed in this cohort are at least partially exclusive from the underlying genotype (Fig. 4).

### Differential methylation at other genes within the MHC locus

**Figure 5:**
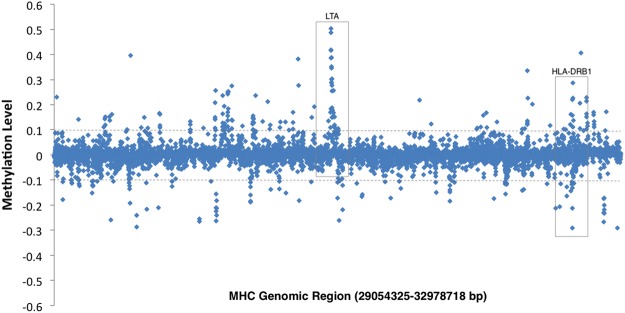
Methylation at the MHC locus in CD19^+^ B cells Manhattan plot showing methylation level (Δ_meth_) for all probes that fall within the MHC locus (Chr6: 29054321-32978719). Points above 0 represent hypermethylated sites, points below 0 represent hypomethylated sites. Grey dotted line indicates 10% change in methylation.

Our previous study in CD4^+^ T-cells identified a peak of differential methylation on chromosome 6 that mapped to the MHC region^4^. Specifically, we found a differentially methylated region that spanned 11 sites at the well-established MS risk gene, *HLA-DRB1* that was unique to CD4^+^ T-cells^4,5^. To determine if there is any overlap of DMPs in the MHC region between CD4^+^ T-cells and CD19^+^ B-cells, we performed a closer analysis of the MHC region. Figure 5 shows that although a similar distinct peak is present at the MHC region, it corresponds primarily to the DMR at *LTA* and to a lesser extent *HLA-DRB1*. However, there are 4 DMPs in CD19^+^ B-cells that overlap with the sites found in CD4^+^ T-cells (Table 3). These sites correspond to probes cg04985482, cg06032479, cg17416722, and cg24147543. The first site maps to the MHC class I polypeptide related sequence A (*MICA*) locus and the remaining three sites all map to sites within *HLA-DRB1*. All sites are altered in the same direction (hypo- or hypermethylated) and have a similar differential methylation value in both cell subsets (Table 3).

**Table 3:** Common sites at the MHC locus in CD4 and CD19.

IlmnID	CHR	MAPINFO	Gene	CD4				CD19			
				mean MS	mean HC	Δmeth	P value	mean MS	mean HC	Δmeth	P value
cg04985482	6	31382065	MICA	0.72	0.84	−0.12	9.93E-03	0.73	0.84	−0.11	2.99E-02
cg06032479	6	32552026	HLA-DRB1	0.65	0.75	−0.10	7.00E-03	0.65	0.78	−0.13	1.20E-02
cg17416722	6	32554385	HLA-DRB1	0.38	0.07	0.31	1.02E-03	0.28	0.06	0.22	1.40E-03
cg24147543	6	32554481	HLA-DRB1	0.47	0.34	0.14	6.44E-04	0.28	0.12	0.16	1.20E-02

Data from CD4^+^ T cells from Graves, M. C. *et al*. and Maltby, V. E. *et al*.^4,6^.

### Differential methylation at sites outside the MHC locus

To explore the importance of methylation outside the MHC region, we filtered DMPs outside the MHC to include only those contained within the TSS or 5′UTR (1953 DMPs). We chose the TSS and 5′UTR as an initial filtering step because DNA methylation that occurs in the promoter regions is generally associated with transcriptional repression, but its role elsewhere in the genome is more complex and less well understood^15^. We then further filtered the list to include only DMRs. A DMR was considered if i) there were 2 or more DMPs, ii) these DMPs fell within a 500 bp span iii) the DMPs were altered in the same direction. This generated a list of 276 genes which contained a DMR in their 5′UTR or TSS.

A comparison of this list to genes with a known association to MS^2,3,16–18^ revealed 4 DMRs (Table 4). Choline transporter-like 2 (*SLC44A2*) and Lymphotoxin β receptor (*LTBR*) each have 2 hypomethylated DMPs within their TSS/5′UTR which are 72 and 113 bp apart, respectively. Caspase recruitment domain-containing protein 11 (*CARD11*) has 2 DMPs at the 5′UTR. Both of which are hypermethylated and 101 bp apart. There is a third DMP which is hypermethylated at the 5′UTR of *CARD11*; however, it is located >62,000 bp downstream of the DMR so it did not fulfil the criteria to be part of the DMR. At the TSS of the CXC chemokine receptor 5 (*CXCR5*), there are 2 DMRS that are 9266 bp apart. The first of these has 5 hypermethylated DMPs within an 86 bp span. These DMPs are between 20.7% and 31.1% hypermethylated. The second contains 3 DMPs within 42 bp of each other that are 17.7%, 13.9% and 18% hypermethylated.

**Table 4:** DMRs outside the MHC locus.

IlmnID	CHR	MAPINFO	Gene	mean MS	mean HC	Δmeth	P value	Element
cg24041556	19	10736059	SLC44A2	0.59	0.69	−0.10	2.99E-02	TSS200;Body
cg06561886	19	10736299	SLC44A2	0.69	0.80	−0.11	1.40E-03	5′UTR;1stExon;Body
cg24621362	12	6492890	LTBR	0.13	0.23	−0.10	2.34E-05	TSS1500
cg23079808	12	6493003	LTBR	0.13	0.23	−0.10	1.20E-02	TSS1500
cg19014792	7	3019159	CARD11	0.52	0.32	0.20	4.32E-03	5′UTR
cg16495448	7	3019260	CARD11	0.38	0.22	0.15	1.20E-02	5′UTR
cg14168009	7	3082006	CARD11	0.69	0.42	0.27	4.06E-04	5′UTR
cg16235962	11	118754507	CXCR5	0.60	0.32	0.28	1.04E-04	TSS200
cg04625873	11	118754530	CXCR5	0.56	0.36	0.21	1.20E-02	TSS200
cg25087423	11	118754535	CXCR5	0.71	0.39	0.31	1.20E-02	TSS200
cg26164712	11	118754565	CXCR5	0.61	0.35	0.25	4.32E-03	5′UTR;1stExon
cg16280667	11	118754593	CXCR5	0.61	0.34	0.27	4.06E-04	5′UTR;1stExon
cg04537602	11	118763859	CXCR5	0.62	0.44	0.18	1.04E-04	Body;TSS1500
cg13298528	11	118763863	CXCR5	0.68	0.54	0.14	2.34E-05	Body;TSS1500
cg19791714	11	118763901	CXCR5	0.53	0.35	0.18	4.57E-06	Body;TSS200
cg07597976	16	28943019	CD19	0.55	0.24	0.31	4.06E-04	TSS1500
cg06323049	16	28943094	CD19	0.62	0.33	0.29	1.20E-02	TSS200
cg27565966	16	28943198	CD19	0.68	0.39	0.29	1.40E-03	TSS200
cg05433111	16	28943232	CD19	0.49	0.25	0.24	1.40E-03	TSS200
cg01758575	16	28943288	CD19	0.53	0.31	0.22	4.32E-03	1stExon;5′UTR
cg16454902	16	27414272	IL21R	0.25	0.15	0.10	1.40E-03	TSS200;5′UTR
cg02513379	16	27414281	IL21R	0.28	0.14	0.14	4.57E-06	TSS200;5′UTR
cg00050618	16	27414418	IL21R	0.73	0.50	0.23	2.34E-05	TSS200;5′UTR
cg02787852	16	27414536	IL21R	0.67	0.40	0.27	1.06E-07	1stExon;5′UTR;5′UTR
cg10416668	16	27437730	IL21R	0.75	0.60	0.15	1.20E-02	TSS1500;5′UTR;5′UTR
cg25341726	16	28518331	IL27	0.37	0.50	−0.12	1.20E-02	TSS200
cg00201760	16	28518385	IL27	0.32	0.45	−0.13	1.20E-02	TSS1500

Further analysis revealed several other DMRs outside the MHC region which reside in genes that may have biological significance to MS pathology. Of interest, there are 5 DMPs which lie within a 269 bp span at the cluster of differentiation 19 (*CD19*) locus. All are found within the TSS or 5′UTR and are hypermethylated by 22.4–30.7%. There is also a DMR at interleukin 21 receptor (*IL21R*) where 4 hypermethylated DMPs lie within a 264 bp span at the TSS.

### Over-Representation Analysis (ORA)

The 7618 DMPs identified in the methylation analysis were located in 2899 genes. To assess the biological relevance of this gene set in terms of MS pathology we conducted a ORA using the WebGestalt engine to identify potential pathways associated with the 2899 gene set. Pathway analysis revealed significant alignment to innate immune system (293 genes, P = 4.08E-09), B-cell receptor signaling pathway (28 genes, P = 3.31E-04), cytokine signaling in Immune system (166 genes, 4.14E-04), and signaling by interleukins (119 genes, 1.46E-03). Table 5 shows the top 10 pathways identified.

**Table 5:** Pathways Analysis of Genes with dysregulated DMPs.

Pathway (BioSystems)	Source	No. of genes	FDR P-value
Neutrophil degranulation	REACTOME	138	9.20E-10
Innate Immune System	REACTOME	293	4.08E-09
Hematopoietic cell lineage	KEGG	40	8.88E-07
Hemostasis	REACTOME	150	3.16E-05
Extracellular matrix organization	REACTOME	80	1.54E-04
Signalling events mediated by focal adhesion kinase	Pathway Interaction Database	24	1.97E-04
Phospholipase D signalling pathway	KEGG	46	3.31E-04
B cell receptor signalling pathway	KEGG	28	3.31E-04
Regulation of RAC1 activity	Pathway Interaction Database	19	3.82E-04
Cytokine Signalling in Immune system	REACTOME	166	4.14E-04

## Discussion

B-cells are gaining recognition in MS as potential regulators of disease pathology. In this study, we are the first to describe changes in the global DNA methylation profile in the CD19^+^ B-cells of MS patients compared to healthy controls. We find a slight overall hypomethylation and enrichment of genes involved in innate immunity and B-cell receptor and cytokine signaling pathways. We have identified a large, hypermethylated DMR in the TSS of *LTA* that is unique to the B-cell population. In addition, we identified four smaller DMRs at genes which contain known MS-associated SNPs, *SLC44A2, LTBR, CXCR5*, and *CARD11*.

The large DMR at *LTA* is of interest due to its longstanding, strong associations with MS. *LTA* encodes for the pro-inflammatory cytokine lymphotoxin-alpha (LT-α). *LTA* is over-expressed in CD4^+^ T-cells, CD8^+^ T-cells and CD19^+^ B-cells of RRMS patients^19^. Furthermore, in RRMS patients the *LTA* CSF/PBMC expression ratios are increased, and positively correlate with *CD19* expression in CSF cells^19^. LTA producing cells have been found in the immediate vicinity of the demyelinating process in MS patients^20^ and expression is present in acute and chronic active brain lesions in MS patients^21^. One inconsistency is that we have shown hypermethylation in the TSS, which implies potential downregulation of transcription (as opposed to over-expression). The most likely explanation for this inconsistency is the presence of hydroxymethylation. Bisulfite conversion does not distinguish between hydroxymethylated and methylated sites; thus, both are considered methylated by the methods used in this study. Unlike methylation, which negatively correlates with transcription, hydroxymethylation has been found to positively correlate with active transcription^22–25^. Therefore, it is plausible that the methylation changes at *LTA* are due to changes in hydroxymethylation which would result in the overexpression seen in previous studies.

Another explanation for the inconsistency between our findings and previous studies could be due to transcript variants. *LTA* is known to have eight transcript variants, with multiple start sites^26^. Thus, the hypermethylation seen in our study may be related to an alternate transcriptional variant to that identified in previous studies. Alternately, previous studies were conducted primarily in treatment naïve patients, whereas our cohort only contains 1 treatment naïve sample. Therefore, hypermethylation and decreased *LTA* expression may be a result of treatment effects, or simply be reflective of disease stabilization.

Although we find DMRs at four other genes previously associated with MS, the functional significance of these DMRs is unclear. *SLC44A2* is found in the peripheral tissues and has been associated with thrombosis and autoimmune hearing loss but not MS^27^. *CXCR5* is used as the defining marker for follicular B helper T-cells (T_FH_) but its expression has not been demonstrated in B-cells^28^.

A recent study found *LTBR* expression levels increased in the animal model of MS, experimental autoimmune encephalitis (EAE), and that blockage of this receptor ameliorated disease in mice^29^. The same study investigated *LTBR* expression in RRMS patients and found increased transcript levels in patients who were resistant to interferon beta (IFNβ) therapy^29^. Hypomethylation in the TSS may be correlated with increased transcription of *LTBR*; however, the study by Inoue and colleagues used PBMCs, which contain a mixture of T-cells and B-cells, so it remains to be elucidated if increased expression is occurring in B-cells.

Although *CARD11* does not yet have a demonstrated, functional role in MS, it is an essential scaffolding platform for the CARD11/ BCL10/MALT1 (CBM) complex^30^. NFκB governs the BCR-induced (B cell receptor) NFκB activation through a complex series of phosphorylation events that results in destruction of the NFκB inhibitor, IκB^30^. One known mechanism of action of the common MS therapy, dimethyl fumarate, is inhibition of the NFκB transcription factor; therefore, an intriguing possibility is that dysregulation of this pathway may play a role in MS pathology^31^.

Although not part of the MHC locus or previously linked to MS, *IL21R* is involved in other autoimmune conditions such as systemic lupus erythematosus (SLE)^32^ and arthritis^33^. It has been linked to B-cell proliferation and survival as well as B-cell apoptosis which suggesting a role in immune cell function^34,35^.

Autoimmune diseases often have overlapping aetiological and genetic backgrounds^36^. In our previous studies, we found that there is also overlap in epigenetic profiles of CD4^+^ T-cells from SLE and MS patients^6^. Recently, Julià *et al*.^37^ assessed the DNA methylation profiles of B-cells from rheumatoid arthritis (RA) patients and performed a comparison with SLE patients^37^. To determine if there is overlap in the epigenetic profiles of CD19^+^ B-cells we compared our results to this study. Of the ten probes identified in their study, five also show differential methylation in the same direction (all hypermethylated) as in our study (Table 6). This suggests a common epigenetic precursor or epigenetic effect among related autoimmune diseases.

**Table 6:** Probes which are differentially methylated in RA, SLE and MS.

CpG	Chr	Gene	RA (n = 65 cases) Δ_meth_	SLE (n = 47 cases) Δ_meth_	MS (n = 24 cases) Δ_meth_
**cg18972751**	**1**	**CD1C**	**5.7**	**3.4**	**5.3**
**cg09327855**	**1**	**NID1**	**1.3**	**1.1**	**10.2**
cg03055617	3	TNFSF10	−6.9	−5.9	
cg06613783	10	SKIDA1	2.7	1.6	
cg07285641	13	DHRS12	1.7	1	
**cg01619562**	**14**	**ITPK1**	**3.4**	**1.03**	**3.8**
cg01810713	16	IRF8	3.1		10.1
**cg04033022**	**16**	**ACSF3**	**2.6**	**0.12**	**14.8**
**cg00253346**	**22**	**TNFRSF13C**	**2**	**1.4**	**8.1**
cg08271031	22	PARVG	2.2	3	

Bold = differential methylation in all three diseases. Based on ref.^36^. Julia A*. et al*. *Hum Mol Genet*. 2017; 26(14):2803-11.

One important consideration for our study is that the patients tested were taking various medications at the time of recruitment including interferons, glatiramer acetate, natalizumab and fingolimod. Only one patient was treatment naïve and 4 had been off treatment for more than 6 months. Although this study controlled for age, sex and treatment effects (as much as possible), due to our limited size we cannot control for changes associated with various environmental factors. Additionally, we were unable to control for B cell subtype compositions. As a third of the patients were taking fingolimod, this may have caused a significant change in the circulating cells.

This study adds to our knowledge of epigenetic factors in MS and further highlights the need to investigate individual cell subtypes when assessing DNA methylation in disease. It also raises several new and important questions including i) are these changes due to treatment effects ii) is the change in methylation at *LTA* due to hydroxymethylation iii) what role do environmental factors play on methylation changes iv) are the methylation effects due to changes in B cell subtypes and v) are DNA methylation changes a pre-disposing factor for MS or are they a result of disease pathology? Further studies are required using larger, treatment naïve cohorts that include epidemiological data will help extract if these results are due to treatment effects and allow the addition of environmental factors such as vitamin D, EBV virus infection and smoking as covariates in the analysis. Additionally, further studies should attempt to extrapolate the relative effect of methylation versus hydroxymethylation, possibly using a more targeted approach such as next generation sequencing. Overall, our results suggest that B-cell specific epigenetics may play a role in MS pathology. B-cell specific epigenetic therapies which target *LTA* expression would therefore be an attractive new avenue of research in MS treatments.

## Electronic supplementary material

Supplementary table 1

## Data Availability

The datasets generated or analyzed during the current study are included in this published article (Supplementary Table 1). Raw data files are available from Assoc. Prof. Rodney A. Lea.
