# Supplementary material for: Genome-wide DNA methylation changes in CD19+ B cells from relapsing-remitting multiple sclerosis patients
Source: Sci Rep. 2018 Nov 27;8:17418. doi: 10.1038/s41598-018-35603-0 (PMC6258668; doi:10.1038/s41598-018-35603-0)
Supplement: Supplementary file 1 — Supplementary table 1 [file 41598_2018_35603_MOESM1_ESM.pdf]

Genome-wide DNA methylation changes in CD19+ B cells from relapsing-remitting multiple sclerosis patients

SUPPLEMENTAL FILES

Vicki E. Maltby[1,2]#, Rodney A. Lea[1,3]#, Moira C. Graves[1,2], Katherine A. Sanders[2,4], Miles C. Benton[1,3], Lotti Tajouri[4], Rodney J. Scott[2,5,6] and Jeannette Lechner-Scott[1,2,7*]

1. School of Medicine and Public Health, University of Newcastle, Newcastle, Australia
2. Centre for Information Based Medicine, Hunter Medical Research Institute, Newcastle, Australia
3. Institute of Health and Biomedical Innovation, Queensland University of Technology, Brisbane Australia
4. Faculty of Health Sciences and Medicine, Bond University, Gold Coast, Australia
5. School of Biomedical Sciences and Pharmacy, University of Newcastle, Newcastle, Australia
6. Medical Genetics, Pathology North, John Hunter Hospital, Newcastle, Australia
7. Department of Neurology, John Hunter Hospital, Newcastle, Australia

# These authors contributed equally to this manuscript

Author emails:
VEM: <vicki.e.maltby@newcastle.edu.au>
RAL: <rodney.a.lea@gmail.com>
MCG: <moira.graves@newcastle.edu.au>
KAS: <ksanders@bond.edu.au>
MCB: <m.benton@qut.edu.au>
LT: <ltajouri@bond.edu.au>
RJS: <rodney.scott@newcastle.edu.au>

*Corresponding Author: Jeannette Lechner-Scott
Email: Jeannette.lechner-scott@hnehealth.nsw.gov.au
Phone: +61 2 4921 3540
Mailing address: Locked Bag 1, Hunter Region Mail Centre, NSW Australia 2310

IlmnID	Infin	Genc	CHR	MAPINFO	UCSC_RefGene_Na	ks.score	ks.pval	logP	median_MS	median_HC	median_diff
cg14437551	I	37	6	31539986	LTA;LTA;LTA	0.62	0.0001	3.98291	0.69538633	0.191327929	0.504
cg14597739	I	37	6	31539998	LTA;LTA;LTA	0.62	0.0001	3.98291	0.73580609	0.249314746	0.486
cg14252149	II	37	1	236707045	LGALS8;LGALS8;LG/	0.42	0.02991	1.52413	0.50216265	0.045509279	0.457
cg20429104	I	37	18	74114570	ZNF516	0.62	0.0001	3.98291	0.40173711	0.859184844	-0.457
cg11327657	I	37	21	46388162	C21orf70	0.54	0.0014	2.85345	0.4465553	0.896435533	-0.45
cg26872907	I	37	6	161796854	PARK2;PARK2;PARK	0.54	0.0014	2.85345	0.3612249	0.798199879	-0.437
cg25131632	I	37	10	94549040		0.5	0.00432	2.36443	0.65451673	0.221307125	0.433
cg00033213	II	37	8	144399335	TOP1MT	0.42	0.02991	1.52413	0.08705082	0.50630562	-0.419
cg09621572	I	37	6	31539973	LTA;LTA;LTA	0.71	4.57E-06	5.34042	0.65896587	0.240829232	0.418
cg16219283	I	37	6	31540002	LTA;LTA;LTA	0.62	0.0001	3.98291	0.71711418	0.299699519	0.417
cg01081438	II	37	1	92417998	BRDT;BRDT	0.46	0.01197	1.92207	0.46656447	0.05393621	0.413
cg04478251	I	37	17	998432	ABR;ABR;ABR	0.54	0.0014	2.85345	0.40051125	0.812453074	-0.412
cg12165551	II	37	11	8385712		0.58	0.00041	3.39184	0.35058395	0.761202348	-0.411
cg10838410	I	37	12	6659524	IFFO1;IFFO1;IFFO1	0.54	0.0014	2.85345	0.28851717	0.695974452	-0.407
cg05205074	II	37	14	107259880		0.5	0.00432	2.36443	0.68853116	0.28268381	0.406
cg03728799	II	37	17	74978687		0.54	0.0014	2.85345	0.62975839	0.224430873	0.405
cg05260077	II	37	21	37531890		0.62	0.0001	3.98291	0.40645654	0.81018095	-0.404
cg19097880	II	37	11	128160637		0.62	0.0001	3.98291	0.60288677	0.205480441	0.397
cg25684151	II	37	8	126588554		0.54	0.0014	2.85345	0.45240362	0.846237291	-0.394
cg11597902	I	37	17	75096239		0.54	0.0014	2.85345	0.42925166	0.82268919	-0.393
cg17496921	II	37	19	11406993	TSPAN16	0.58	0.00041	3.39184	0.43359443	0.825766576	-0.392
cg01491428	II	37	16	2334163	ABCA3	0.5	0.00432	2.36443	0.780325	0.389269439	0.391
cg20078972	II	37	19	15391832	BRD4;BRD4	0.5	0.00432	2.36443	0.39697315	0.78771025	-0.391
cg08106973	II	37	1	40399833		0.58	0.00041	3.39184	0.36164548	0.750917866	-0.389
cg04493247	I	37	7	72719224	NSUN5;NSUN5;NSU	0.62	0.0001	3.98291	0.40689211	0.794585356	-0.388
cg21999229	I	37	6	31540014	LTA;LTA;LTA	0.67	2.34E-05	4.63072	0.65159786	0.265005506	0.387
cg23447233	I	37	4	1295047	MAEA;MAEA	0.54	0.0014	2.85345	0.4292295	0.814733655	-0.386
cg24801230	II	37	17	43978533	MAPT;MAPT;MAPT	0.42	0.02991	1.52413	0.4566855	0.843069079	-0.386
cg14441276	II	37	6	31539735	LTA;LTA	0.58	0.00041	3.39184	0.58501317	0.200005586	0.385
cg26709988	I	37	16	84860918	CRISPLD2	0.58	0.00041	3.39184	0.52329806	0.906781459	-0.383
cg16651347	II	37	1	175042297	TNN	0.54	0.0014	2.85345	0.4062851	0.788424417	-0.382
cg08165960	I	37	15	101777800	CHSY1	0.54	0.0014	2.85345	0.40544115	0.784040377	-0.379

cg00009523	II	37	2	1165351	SNTG2	0.5	0.00432	2.36443	0.48728992	0.864314202	-0.377
cg25376491	II	37	5	179192524	MAML1	0.62	0.0001	3.98291	0.47798731	0.852653949	-0.375
cg07714276	II	37	6	7169632	RREB1;RREB1;RREB	0.58	0.00041	3.39184	0.40044912	0.775403653	-0.375
cg06684088	II	37	7	119131		0.62	0.0001	3.98291	0.42419478	0.799525509	-0.375
cg21574822	II	37	4	2431998		0.58	0.00041	3.39184	0.3018431	0.674999564	-0.373
cg17768768	I	37	7	2948123	CARD11	0.5	0.00432	2.36443	0.64777594	0.275107139	0.373
cg07053114	I	37	10	129794994	PTPRE	0.54	0.0014	2.85345	0.51019172	0.880607819	-0.37
cg01827633	II	37	2	219610103	TTLL4	0.62	0.0001	3.98291	0.37726981	0.746304149	-0.369
cg15730481	I	37	7	1126318	GPER;C7orf50;C7or	0.58	0.00041	3.39184	0.6084354	0.239271056	0.369
cg22324981	I	37	18	77283493	NFATC1;NFATC1;NF	0.71	4.57E-06	5.34042	0.67162002	0.304073641	0.368
cg03801286	II	37	21	35884508	KCNE1	0.58	0.00041	3.39184	0.4232379	0.790599289	-0.367
cg06468347	II	37	17	3705875	ITGAE	0.58	0.00041	3.39184	0.37331492	0.737860922	-0.365
cg22528270	I	37	7	151505116	PRKAG2;PRKAG2	0.58	0.00041	3.39184	0.50073755	0.865203411	-0.364
cg05834845	I	37	3	195489306	MUC4;MUC4;MUC4	0.67	2.34E-05	4.63072	0.54201556	0.904551962	-0.363
cg21923525	II	37	18	9474143	RALBP1	0.58	0.00041	3.39184	0.37944248	0.742317369	-0.363
cg15919816	II	37	4	2431872		0.67	2.34E-05	4.63072	0.30500907	0.667170405	-0.362
cg13573745	II	37	9	97405553		0.58	0.00041	3.39184	0.39442112	0.754020757	-0.36
cg18443571	II	37	15	90547692	ZNF710	0.58	0.00041	3.39184	0.39796034	0.757273921	-0.359
cg23244761	I	37	6	161796850	PARK2;PARK2;PARK	0.54	0.0014	2.85345	0.53576916	0.894092416	-0.358
cg18554789	II	37	7	139432178	HIPK2;HIPK2	0.62	0.0001	3.98291	0.42088941	0.778496669	-0.358
cg01406317	II	37	16	4397291	Magmas	0.54	0.0014	2.85345	0.45288307	0.810730051	-0.358
cg24137448	II	37	17	46189113	SNX11;SNX11	0.5	0.00432	2.36443	0.63771093	0.279413764	0.358
cg02435083	II	37	16	8943436		0.58	0.00041	3.39184	0.42632203	0.783368946	-0.357
cg01479396	II	37	17	7529693	SHBG;SAT2	0.54	0.0014	2.85345	0.43685872	0.79342457	-0.357
cg10323962	II	37	16	87921685	CA5A	0.62	0.0001	3.98291	0.36524149	0.721262285	-0.356
cg03455225	II	37	3	23313729	UBE2E2	0.54	0.0014	2.85345	0.90285244	0.550132254	0.353
cg15212210	II	37	11	118986793	C2CD2L	0.54	0.0014	2.85345	0.33113503	0.683635289	-0.353
cg24091162	II	37	17	62010456	CD79B;CD79B;CD79	0.5	0.00432	2.36443	0.74104854	0.388043088	0.353
cg24216966	I	37	6	31540121	LTA;LTA;LTA	0.62	0.0001	3.98291	0.73085498	0.378454606	0.352
cg06743610	II	37	19	35630305	FXYD1;FXYD1	0.54	0.0014	2.85345	0.42644578	0.778448663	-0.352
cg21159128	I	37	1	54693933	SSBP3;SSBP3;SSBP3	0.5	0.00432	2.36443	0.34571367	0.696553039	-0.351
cg25505880	II	37	11	62574338	NXF1;NXF1	0.58	0.00041	3.39184	0.43125039	0.782728734	-0.351
cg11554335	II	37	11	57336941	UBE2L6	0.67	2.34E-05	4.63072	0.48148726	0.82839691	-0.347

cg19502359	II	37	11	72912441		0.67	2.34E-05	4.63072	0.46594867	0.813352199	-0.347
cg00686823	II	37	3	127311038	TPRA1	0.58	0.00041	3.39184	0.36516755	0.710945943	-0.346
cg17169196	I	37	6	31540026	LTA;LTA;LTA	0.67	2.34E-05	4.63072	0.70578347	0.359284527	0.346
cg23590660	II	37	12	8205602	FOXJ2	0.58	0.00041	3.39184	0.37244069	0.718468538	-0.346
cg08782022	I	37	16	4730136	MGRN1;MGRN1;M(	0.54	0.0014	2.85345	0.41248001	0.758436102	-0.346
cg26686361	I	37	16	85964073		0.58	0.00041	3.39184	0.45062438	0.796863683	-0.346
cg18655369	II	37	19	1177575		0.58	0.00041	3.39184	0.38847333	0.734849236	-0.346
cg09470754	II	37	19	55838021	TMEM150B	0.62	0.0001	3.98291	0.38765521	0.733837483	-0.346
cg08458487	II	37	10	81709191	SFTPD	0.54	0.0014	2.85345	0.47000517	0.813749711	-0.344
cg24012880	II	37	11	44880910	TSPAN18	0.62	0.0001	3.98291	0.49612551	0.839956312	-0.344
cg22666015	II	37	2	233981885	INPP5D;INPP5D	0.62	0.0001	3.98291	0.42681591	0.77030656	-0.343
cg00975624	II	37	3	159646501		0.54	0.0014	2.85345	0.42482861	0.767964143	-0.343
cg21326139	II	37	4	1294783	MAEA;MAEA	0.5	0.00432	2.36443	0.41929622	0.762007374	-0.343
cg24941342	II	37	11	95093809		0.54	0.0014	2.85345	0.51577687	0.857412139	-0.342
cg04072771	II	37	15	55574912	RAB27A	0.5	0.00432	2.36443	0.36101315	0.703012307	-0.342
cg06755438	II	37	2	219885614	CCDC108	0.62	0.0001	3.98291	0.38443222	0.725408479	-0.341
cg00259404	II	37	6	28885568	TRIM27	0.58	0.00041	3.39184	0.45564305	0.796921594	-0.341
cg15235987	II	37	12	132469937	EP400	0.62	0.0001	3.98291	0.44414048	0.785279661	-0.341
cg07285167	II	37	1	36948981	CSF3R;CSF3R;CSF3F	0.62	0.0001	3.98291	0.42001911	0.759894849	-0.34
cg12691572	I	37	10	114574959	VTI1A	0.54	0.0014	2.85345	0.47461067	0.815109631	-0.34
cg22907103	II	37	11	61124346	CYBASC3;CYBASC3;	0.5	0.00432	2.36443	0.68069615	0.340467467	0.34
cg04828493	I	37	13	111328783	CARS2	0.5	0.00432	2.36443	0.69014636	0.349911651	0.34
cg10718056	II	37	6	28884599	TRIM27	0.62	0.0001	3.98291	0.53546725	0.874190304	-0.339
cg02688118	II	37	13	114918456		0.62	0.0001	3.98291	0.48027418	0.817794277	-0.338
cg26076724	I	37	6	15090163		0.58	0.00041	3.39184	0.46457772	0.801372498	-0.337
cg00066854	II	37	10	113987376		0.67	2.34E-05	4.63072	0.42536862	0.762867245	-0.337
cg22111527	I	37	11	69260136		0.58	0.00041	3.39184	0.43214058	0.768696661	-0.337
cg16409562	I	37	14	21485900	NDRG2;NDRG2;NDI	0.54	0.0014	2.85345	0.55086121	0.887593354	-0.337
cg09284275	II	37	16	15923487	MYH11;MYH11;MY	0.62	0.0001	3.98291	0.44618951	0.782845588	-0.337
cg04217515	I	37	21	46325853	ITGB2;ITGB2	0.58	0.00041	3.39184	0.53544508	0.872733165	-0.337
cg11953913	II	37	5	139562873	C5orf32	0.67	2.34E-05	4.63072	0.50526837	0.840946465	-0.336
cg02956248	I	37	6	32120901	PPT2;PRRT1;PPT2	0.5	0.00432	2.36443	0.81917587	0.483487103	0.336
cg04382396	I	37	19	852311	ELANE;ELANE	0.67	2.34E-05	4.63072	0.55555333	0.891392299	-0.336

cg18503912	II	37	19	35630279	FXYD1;FXYD1	0.58	0.00041	3.39184	0.44726449	0.783455005	-0.336
cg02896872	II	37	1	226887044	ITPKB	0.5	0.00432	2.36443	0.75028478	0.415269482	0.335
cg13577149	II	37	5	134783581	C5orf20	0.5	0.00432	2.36443	0.70019725	0.365568818	0.335
cg04958236	II	37	6	74276973		0.67	2.34E-05	4.63072	0.53108506	0.865657846	-0.335
cg24788483	I	37	10	114911652	TCF7L2;TCF7L2;TCF	0.67	2.34E-05	4.63072	0.52093839	0.855263332	-0.334
cg06380691	II	37	13	114828264	RASA3	0.54	0.0014	2.85345	0.32921415	0.663060925	-0.334
cg16786178	II	37	14	75764329		0.58	0.00041	3.39184	0.44425002	0.778175376	-0.334
cg07298177	II	37	16	85577847		0.54	0.0014	2.85345	0.47507818	0.808480003	-0.333
cg17007896	II	37	11	69261044		0.67	2.34E-05	4.63072	0.42835983	0.76056709	-0.332
cg00517080	II	37	11	134098583	VPS26B	0.58	0.00041	3.39184	0.5586226	0.226613485	0.332
cg25099490	II	37	14	23583133		0.5	0.00432	2.36443	0.37326823	0.70477854	-0.332
cg21696012	II	37	1	24131000	HMGCL;HMGCL	0.58	0.00041	3.39184	0.52759659	0.85825389	-0.331
cg26226650	II	37	3	50276265	GNAI2	0.54	0.0014	2.85345	0.39862926	0.729858295	-0.331
cg06298740	II	37	1	227125826		0.62	0.0001	3.98291	0.51694367	0.846450166	-0.33
cg25634666	II	37	11	71846788	FOLR3;FOLR3	0.54	0.0014	2.85345	0.43984609	0.77005907	-0.33
cg16206504	I	37	13	114917223		0.54	0.0014	2.85345	0.53323045	0.863429784	-0.33
cg19755815	II	37	17	80873572	TBCD	0.54	0.0014	2.85345	0.6820319	0.352167391	0.33
cg03310874	I	37	7	4850260	RADIL	0.62	0.0001	3.98291	0.51653135	0.845128574	-0.329
cg06551697	II	37	17	56612362		0.54	0.0014	2.85345	0.43113221	0.759827919	-0.329
cg26831416	II	37	19	39142011	ACTN4	0.62	0.0001	3.98291	0.43049408	0.759927343	-0.329
cg00343906	II	37	1	1696692	NADK	0.58	0.00041	3.39184	0.51733738	0.845267015	-0.328
cg02505177	II	37	10	103574626	MGEA5;MGEA5	0.58	0.00041	3.39184	0.48969406	0.161725202	0.328
cg13738327	I	37	11	68139233	LRP5	0.5	0.00432	2.36443	0.61351318	0.285541867	0.328
cg12249234	II	37	17	25867613	KSR1	0.58	0.00041	3.39184	0.48331469	0.810974681	-0.328
cg17950348	II	37	7	157183466	DNAJB6	0.5	0.00432	2.36443	0.36900824	0.695627395	-0.327
cg20805133	I	37	2	242802192	PDCD1	0.75	7.61E-07	6.11857	0.59860652	0.273911488	0.325
cg03857198	II	37	3	152927597		0.54	0.0014	2.85345	0.55242006	0.877596195	-0.325
cg11638399	II	37	8	29441416		0.5	0.00432	2.36443	0.55571082	0.880498279	-0.325
cg08698943	II	37	10	3509758		0.54	0.0014	2.85345	0.38732663	0.711857278	-0.325
cg07698804	I	37	17	80829309	TBCD	0.54	0.0014	2.85345	0.45178922	0.126631473	0.325
cg01554529	I	37	1	11722935	FBXO6;FBXO44;FBX	0.62	0.0001	3.98291	0.52230268	0.845934775	-0.324
cg02570354	II	37	3	31728749	OSBPL10	0.54	0.0014	2.85345	0.45504559	0.778881698	-0.324
cg10147394	II	37	8	11350297	BLK	0.42	0.02991	1.52413	0.6675391	0.343104312	0.324

cg08400494	I	37	13	111318490	CARS2	0.54	0.0014	2.85345	0.43385125	0.758008253	-0.324
cg17540545	I	37	19	35630355	FXYD1;FXYD1	0.58	0.00041	3.39184	0.34828476	0.672209023	-0.324
cg06005892	II	37	11	3177622	OSBPL5;OSBPL5;OS	0.58	0.00041	3.39184	0.41567238	0.73883645	-0.323
cg01697902	II	37	14	25046117	CTSG	0.5	0.00432	2.36443	0.50906398	0.831825	-0.323
cg16378063	II	37	17	47329552		0.58	0.00041	3.39184	0.46164684	0.784185138	-0.323
cg02862467	II	37	1	19407897	UBR4	0.54	0.0014	2.85345	0.48269746	0.80490255	-0.322
cg17078393	II	37	1	32717002	LCK	0.54	0.0014	2.85345	0.6759174	0.353543394	0.322
cg06748146	II	37	10	71104724	HK1;HK1;HK1;HK1;I	0.54	0.0014	2.85345	0.51806672	0.196360468	0.322
cg10718809	II	37	11	64087106	PRDX5;PRDX5;PRD)	0.62	0.0001	3.98291	0.38205041	0.703624022	-0.322
cg16085171	II	37	12	123569118	PITPNM2	0.46	0.01197	1.92207	0.73550835	0.413582667	0.322
cg02659854	II	37	13	24824078	SPATA13;SPATA13	0.54	0.0014	2.85345	0.45667386	0.778921242	-0.322
cg14742445	II	37	22	30195299	ASCC2	0.58	0.00041	3.39184	0.42585025	0.747352134	-0.322
cg03321813	II	37	4	84257153	HPSE;HPSE;HPSE	0.54	0.0014	2.85345	0.3363846	0.657859443	-0.321
cg06657721	I	37	6	25027618		0.67	2.34E-05	4.63072	0.47677723	0.155750094	0.321
cg03155200	I	37	11	2833259	KCNQ1;KCNQ1	0.5	0.00432	2.36443	0.55334011	0.873956574	-0.321
cg00974864	II	37	1	161601053	FCGR3B;FCGR3B	0.54	0.0014	2.85345	0.40942335	0.729672893	-0.32
cg17660833	II	37	3	11267020	HRH1;HRH1;HRH1	0.62	0.0001	3.98291	0.51793357	0.837446145	-0.32
cg21519701	II	37	17	62252524	TEX2	0.54	0.0014	2.85345	0.50552885	0.82514458	-0.32
cg25059899	II	37	18	60904328	BCL2	0.67	2.34E-05	4.63072	0.5539513	0.874160444	-0.32
cg17356733	II	37	21	34774627	IFNGR2	0.67	2.34E-05	4.63072	0.55231673	0.872730835	-0.32
cg14138540	I	37	15	93571988		0.54	0.0014	2.85345	0.38913523	0.708160039	-0.319
cg20609803	II	37	1	161184305	FCER1G	0.58	0.00041	3.39184	0.49343614	0.811149017	-0.318
cg19701828	II	37	2	219235515		0.58	0.00041	3.39184	0.37274535	0.690620835	-0.318
cg25856090	II	37	7	143028708	CLCN1	0.58	0.00041	3.39184	0.49631828	0.814524897	-0.318
cg17206393	II	37	20	33681223	TRPC4AP;TRPC4AP	0.54	0.0014	2.85345	0.48639227	0.804764644	-0.318
cg13086983	II	37	1	21664810	ECE1	0.58	0.00041	3.39184	0.42981057	0.747050216	-0.317
cg06430688	II	37	2	242490217		0.46	0.01197	1.92207	0.74437874	0.427302336	0.317
cg08373904	I	37	11	1543275	HCCA2	0.58	0.00041	3.39184	0.62702635	0.309539355	0.317
cg23681017	I	37	4	681086	MFSD7	0.62	0.0001	3.98291	0.53677871	0.853168321	-0.316
cg25913761	I	37	15	90727560	SEMA4B	0.62	0.0001	3.98291	0.56155439	0.245694334	0.316
cg12019801	II	37	18	74114708	ZNF516	0.62	0.0001	3.98291	0.47466446	0.790187761	-0.316
cg22381248	II	37	1	42003713	HIVEP3;HIVEP3	0.58	0.00041	3.39184	0.58816273	0.272959196	0.315
cg05057534	II	37	2	28497669	BRE;BRE;BRE;BRE;B	0.58	0.00041	3.39184	0.46963673	0.784402525	-0.315

cg13709496	II	37	9	101011718	TBC1D2	0.58	0.00041	3.39184	0.41591292	0.730656539	-0.315
cg13179472	I	37	11	11177888		0.54	0.0014	2.85345	0.59792533	0.282740273	0.315
cg21638533	II	37	12	6658625	IFFO1;IFFO1;IFFO1	0.58	0.00041	3.39184	0.46701737	0.782362348	-0.315
cg08183317	II	37	13	114261934	TFDP1;TFDP1	0.54	0.0014	2.85345	0.40040204	0.715626682	-0.315
cg07110356	I	37	17	56355431	MPO	0.62	0.0001	3.98291	0.57769089	0.892609989	-0.315
cg02156723	II	37	1	12100631		0.54	0.0014	2.85345	0.56402592	0.249527946	0.314
cg07377178	II	37	6	3025064		0.62	0.0001	3.98291	0.39970719	0.712811036	-0.313
cg25888881	II	37	10	31288287	ZNF438;ZNF438;ZN	0.62	0.0001	3.98291	0.48589914	0.79846296	-0.313
cg04046364	II	37	12	58210661	AVIL	0.58	0.00041	3.39184	0.44741181	0.760556875	-0.313
cg04831327	II	37	12	114299076	RBM19;RBM19;RBM	0.58	0.00041	3.39184	0.52333552	0.835933253	-0.313
cg26847100	II	37	19	10748778	SLC44A2;SLC44A2	0.54	0.0014	2.85345	0.43107448	0.744144252	-0.313
cg08173915	II	37	21	34774164	IFNGR2	0.67	2.34E-05	4.63072	0.53435388	0.846891515	-0.313
cg18148314	II	37	1	6425319	ACOT7;ACOT7	0.54	0.0014	2.85345	0.64588016	0.33389286	0.312
cg25521400	II	37	2	62445279	B3GNT2	0.54	0.0014	2.85345	0.47977724	0.791406047	-0.312
cg02012974	II	37	3	66492992	LRIG1	0.58	0.00041	3.39184	0.47874274	0.790910138	-0.312
cg18915856	II	37	14	21483641		0.54	0.0014	2.85345	0.45906184	0.770731719	-0.312
cg00760938	II	37	2	217357159		0.46	0.01197	1.92207	0.63260889	0.321135425	0.311
cg25087423	II	37	11	118754535	CXCR5	0.46	0.01197	1.92207	0.70553428	0.394073035	0.311
cg07488141	II	37	7	47560215	TNS3	0.54	0.0014	2.85345	0.48744603	0.797637912	-0.31
cg13999433	II	37	9	117156883	AKNA	0.67	2.34E-05	4.63072	0.50012602	0.810170624	-0.31
cg16148346	II	37	11	58826475		0.5	0.00432	2.36443	0.679063	0.368599047	0.31
cg07618110	II	37	11	60225240	MS4A1;MS4A1	0.46	0.01197	1.92207	0.65302275	0.34282721	0.31
cg11077171	I	37	15	74881051	ARID3B	0.58	0.00041	3.39184	0.53563933	0.845769712	-0.31
cg03689552	II	37	16	3292971	MEFV	0.5	0.00432	2.36443	0.46244086	0.772176706	-0.31
cg01869896	II	37	1	40420299	MFSD2A;MFSD2A	0.54	0.0014	2.85345	0.43707708	0.746536499	-0.309
cg27230882	II	37	1	110976749		0.58	0.00041	3.39184	0.48683777	0.795845366	-0.309
cg16875057	II	37	2	169006003	STK39	0.62	0.0001	3.98291	0.50573305	0.814435697	-0.309
cg14266770	II	37	9	136721183	VAV2;VAV2	0.54	0.0014	2.85345	0.49491561	0.803604463	-0.309
cg23595304	II	37	11	46879516	LRP4	0.58	0.00041	3.39184	0.44061598	0.749873944	-0.309
cg16728539	II	37	12	2451169	CACNA1C;CACNA1C	0.54	0.0014	2.85345	0.46937612	0.778622091	-0.309
cg18004847	II	37	19	1155056	SBNO2	0.58	0.00041	3.39184	0.4284446	0.737603683	-0.309
cg12156512	I	37	19	47258676	FKRP;FKRP	0.54	0.0014	2.85345	0.58753512	0.896764159	-0.309
cg08933467	II	37	1	157776690	FCRL1;FCRL1;FCRL1	0.5	0.00432	2.36443	0.52259816	0.214373988	0.308

cg26313511	II	37	3	125053815	ZNF148	0.54	0.0014	2.85345	0.54426063	0.851853936	-0.308
cg19869035	II	37	7	2653955	IQCE;IQCE	0.58	0.00041	3.39184	0.48535794	0.792940993	-0.308
cg26284388	II	37	10	126289835	LHPP;LHPP	0.46	0.01197	1.92207	0.68950825	0.381408048	0.308
cg09233619	II	37	11	23421870		0.42	0.02991	1.52413	0.54933369	0.857204206	-0.308
cg17161520	II	37	11	67174843	TBC1D10C	0.54	0.0014	2.85345	0.48282778	0.174474592	0.308
cg05928849	II	37	11	122993208	ASAM	0.58	0.00041	3.39184	0.53615051	0.844210702	-0.308
cg24804768	II	37	12	754911	NINJ2	0.62	0.0001	3.98291	0.48612854	0.793727954	-0.308
cg15464148	II	37	12	6745057	LPAR5	0.5	0.00432	2.36443	0.64868873	0.340399437	0.308
cg21052932	II	37	14	51342320	ABHD12B;ABHD12E	0.54	0.0014	2.85345	0.44623962	0.138080885	0.308
cg05977333	II	37	16	86029348		0.46	0.01197	1.92207	0.72945669	0.421149842	0.308
cg12459502	II	37	18	60904237	BCL2	0.62	0.0001	3.98291	0.40554487	0.713211587	-0.308
cg26930596	II	37	1	2082315	PRKCZ;PRKCZ;PRKC	0.58	0.00041	3.39184	0.53233978	0.839361359	-0.307
cg11283860	II	37	1	8273352		0.58	0.00041	3.39184	0.47571148	0.782567171	-0.307
cg08864105	I	37	1	111743020	DENND2D	0.42	0.02991	1.52413	0.29186911	0.598702692	-0.307
cg26552743	II	37	11	58826414		0.54	0.0014	2.85345	0.65147666	0.343997975	0.307
cg11362935	I	37	11	111250201	POU2AF1	0.62	0.0001	3.98291	0.44600406	0.138725802	0.307
cg10264529	II	37	14	24562064	PCK2;PCK2	0.62	0.0001	3.98291	0.51165704	0.819091821	-0.307
cg07597976	II	37	16	28943019	CD19	0.58	0.00041	3.39184	0.54798842	0.240517523	0.307
cg14131824	II	37	17	1490487	SLC43A2	0.67	2.34E-05	4.63072	0.43886331	0.745455145	-0.307
cg12339328	II	37	20	18491484	SEC23B;SEC23B;SEC	0.58	0.00041	3.39184	0.57025872	0.877153832	-0.307
cg23036852	II	37	21	44809983		0.54	0.0014	2.85345	0.35600973	0.662796048	-0.307
cg24492202	I	37	4	2802612	SH3BP2	0.62	0.0001	3.98291	0.52123946	0.827195348	-0.306
cg10454879	I	37	8	602158		0.58	0.00041	3.39184	0.60785857	0.913742586	-0.306
cg13554018	II	37	9	91091556	SPIN1	0.54	0.0014	2.85345	0.53270295	0.226965123	0.306
cg10045881	II	37	1	111770291	CHI3L2;CHI3L2;CHI3	0.62	0.0001	3.98291	0.46517937	0.770361183	-0.305
cg23230830	II	37	1	248903100	LOC646627	0.58	0.00041	3.39184	0.4326217	0.737526636	-0.305
cg04346861	II	37	2	240291097	HDAC4	0.5	0.00432	2.36443	0.71544371	0.410914126	0.305
cg10725937	II	37	6	41130978	TREM2	0.62	0.0001	3.98291	0.47226094	0.776851864	-0.305
cg18275732	I	37	6	170365320		0.5	0.00432	2.36443	0.54549249	0.85011875	-0.305
cg19882315	II	37	8	11351846	BLK;BLK	0.46	0.01197	1.92207	0.61723969	0.311974941	0.305
cg11940526	II	37	10	80311367		0.54	0.0014	2.85345	0.60498819	0.299840566	0.305
cg00554993	II	37	14	23588616	CEBPE	0.58	0.00041	3.39184	0.40028758	0.70493153	-0.305
cg11589536	II	37	3	46250518	CCR1	0.71	4.57E-06	5.34042	0.53567256	0.839585462	-0.304

cg26002008	II	37	5	1477719	LPCAT1	0.58	0.00041	3.39184	0.37270772	0.677053218	-0.304
cg23939096	II	37	5	1555791		0.54	0.0014	2.85345	0.44869719	0.753175052	-0.304
cg09378756	II	37	14	24569543	PCK2;PCK2	0.58	0.00041	3.39184	0.34562541	0.649423215	-0.304
cg07069934	II	37	15	89192956	ISG20	0.62	0.0001	3.98291	0.52502278	0.221449112	0.304
cg16624482	I	37	21	43548126	UMODL1;UMODL1	0.58	0.00041	3.39184	0.59630799	0.899906598	-0.304
cg22013055	II	37	2	47054833	LOC100134259	0.5	0.00432	2.36443	0.60232918	0.298908483	0.303
cg18556976	II	37	3	14320523		0.54	0.0014	2.85345	0.48418088	0.786770186	-0.303
cg18599081	II	37	3	46448084	CCRL2;CCRL2	0.54	0.0014	2.85345	0.45378762	0.757014566	-0.303
cg13815684	I	37	6	31540440	LTA;LTA	0.62	0.0001	3.98291	0.7254413	0.422580722	0.303
cg21234082	II	37	9	124363848	DAB2IP	0.58	0.00041	3.39184	0.36210918	0.665397144	-0.303
cg07692929	II	37	11	7517263	OLFML1	0.5	0.00432	2.36443	0.5906738	0.287644228	0.303
cg08900384	II	37	11	72546168		0.58	0.00041	3.39184	0.40790501	0.710498653	-0.303
cg07248377	II	37	16	4732406	MGRN1;MGRN1;M	0.58	0.00041	3.39184	0.42757044	0.730802792	-0.303
cg15134583	II	37	1	224015075	TP53BP2;TP53BP2	0.54	0.0014	2.85345	0.43739467	0.738962074	-0.302
cg21190228	II	37	2	240132342	HDAC4	0.58	0.00041	3.39184	0.50910218	0.810911671	-0.302
cg25853622	II	37	3	188425256	LPP;LPP;LPP	0.54	0.0014	2.85345	0.53215373	0.834344231	-0.302
cg11586857	I	37	6	31540136	LTA;LTA;LTA	0.62	0.0001	3.98291	0.75046228	0.448607399	0.302
cg01268901	II	37	12	1744957	WNT5B;WNT5B	0.58	0.00041	3.39184	0.4668041	0.769119976	-0.302
cg07272654	I	37	16	86011988		0.62	0.0001	3.98291	0.36053868	0.662227012	-0.302
cg07721872	I	37	16	87735256	LOC100129637	0.58	0.00041	3.39184	0.69221695	0.389846799	0.302
cg12323063	II	37	17	67497879	MAP2K6	0.62	0.0001	3.98291	0.55638801	0.858228682	-0.302
cg16356013	II	37	5	134722079	H2AFY;H2AFY;H2AF	0.67	2.34E-05	4.63072	0.55876542	0.860034321	-0.301
cg21882356	II	37	14	21153451	ANG;RNASE4	0.58	0.00041	3.39184	0.54568386	0.846415403	-0.301
cg27391816	I	37	1	1672644	SLC35E2	0.58	0.00041	3.39184	0.61213803	0.912518854	-0.3
cg27485921	II	37	2	46747379	ATP6V1E2	0.58	0.00041	3.39184	0.48988433	0.7903507	-0.3
cg02244028	II	37	3	38992200	SCN11A	0.62	0.0001	3.98291	0.55287889	0.852687249	-0.3
cg03143046	II	37	6	43758007		0.54	0.0014	2.85345	0.49425165	0.794203803	-0.3
cg11723077	II	37	6	158508188	SYNJ2	0.54	0.0014	2.85345	0.40964566	0.709750555	-0.3
cg11067407	II	37	11	113599852		0.62	0.0001	3.98291	0.43561716	0.735678549	-0.3
cg23575688	II	37	11	119486443		0.58	0.00041	3.39184	0.42028147	0.72072064	-0.3
cg06107293	I	37	15	22915986	CYFIP1	0.5	0.00432	2.36443	0.48716657	0.787030683	-0.3
cg14579184	II	37	17	80873365	TBCD	0.5	0.00432	2.36443	0.6097846	0.309740714	0.3
cg14575739	II	37	19	55098069	LILRA2;LILRA2	0.46	0.01197	1.92207	0.58775456	0.287535264	0.3

cg19157819	II	37	10	75405260	SYNPO2L;SYNPO2L	0.62	0.0001	3.98291	0.44426756	0.743647978	-0.299
cg01849093	II	37	11	110876141		0.62	0.0001	3.98291	0.67251041	0.373195832	0.299
cg26776551	II	37	13	51944507	INTS6;INTS6	0.62	0.0001	3.98291	0.47363017	0.772277477	-0.299
cg06208288	I	37	1	58858074		0.71	4.57E-06	5.34042	0.67647606	0.378607077	0.298
cg24641737	II	37	1	111743271	DENND2D;DENND2	0.46	0.01197	1.92207	0.31661302	0.614438441	-0.298
cg10972897	II	37	1	165859714	UCK2	0.54	0.0014	2.85345	0.5129688	0.810528181	-0.298
cg02319829	II	37	3	169649593	SAMD7	0.5	0.00432	2.36443	0.64802146	0.349980602	0.298
cg09740468	II	37	6	3025325		0.58	0.00041	3.39184	0.44300162	0.741242134	-0.298
cg13400493	II	37	7	44826786		0.42	0.02991	1.52413	0.67610223	0.377933399	0.298
cg06072036	II	37	11	822402	PNPLA2	0.54	0.0014	2.85345	0.43938515	0.73692584	-0.298
cg20357538	I	37	15	101777761	CHSY1	0.54	0.0014	2.85345	0.36570626	0.664063716	-0.298
cg05599723	II	37	1	12241073	TNFRSF1B	0.58	0.00041	3.39184	0.48420427	0.781213929	-0.297
cg13430807	II	37	1	149903315	MTMR11;MTMR11	0.58	0.00041	3.39184	0.50687173	0.803789357	-0.297
cg05954120	II	37	1	156254757	TMEM79;TMEM79	0.54	0.0014	2.85345	0.45782409	0.755235309	-0.297
cg01584932	II	37	2	74708767	TTC31;TTC31;CCDC	0.67	2.34E-05	4.63072	0.43344511	0.730169325	-0.297
cg08944026	II	37	5	10626811	ANKRD33B	0.54	0.0014	2.85345	0.38319689	0.680139595	-0.297
cg14763104	II	37	11	2406384	CD81	0.5	0.00432	2.36443	0.48421961	0.186732859	0.297
cg27405128	II	37	15	58540402		0.62	0.0001	3.98291	0.52623508	0.822777049	-0.297
cg16270399	I	37	18	74257894	LOC284276	0.5	0.00432	2.36443	0.5418442	0.838511121	-0.297
cg12126686	II	37	19	35821634	CD22	0.46	0.01197	1.92207	0.61482035	0.317852581	0.297
cg18590995	II	37	1	226558496	PARP1	0.54	0.0014	2.85345	0.60044982	0.304849326	0.296
cg02505099	II	37	3	122718411	SEMA5B	0.42	0.02991	1.52413	0.61518815	0.318764247	0.296
cg23934477	II	37	5	149741030	TCOF1;TCOF1;TCOF	0.54	0.0014	2.85345	0.71565995	0.419796866	0.296
cg10271819	II	37	7	139426030	HIPK2;HIPK2	0.58	0.00041	3.39184	0.3977886	0.694238885	-0.296
cg08454687	I	37	7	157091435		0.58	0.00041	3.39184	0.46812488	0.764365064	-0.296
cg26661623	II	37	17	7019262	ASGR2;ASGR2;ASGI	0.5	0.00432	2.36443	0.49175617	0.788218514	-0.296
cg21341487	II	37	20	60760919	GTPBP5	0.67	2.34E-05	4.63072	0.56548127	0.861035521	-0.296
cg24575128	II	37	3	52502445	NISCH	0.58	0.00041	3.39184	0.44886818	0.743681605	-0.295
cg25709790	II	37	7	24742552	DFNA5;DFNA5;DFN	0.42	0.02991	1.52413	0.5562601	0.851473818	-0.295
cg14480046	I	37	12	122444580		0.67	2.34E-05	4.63072	0.60913264	0.314343117	0.295
cg12940993	II	37	14	21271148	RNASE1;RNASE1;RN	0.54	0.0014	2.85345	0.43966874	0.734659863	-0.295
cg11465404	II	37	15	22550643		0.58	0.00041	3.39184	0.42870282	0.723725621	-0.295
cg08493063	II	37	16	11330934		0.54	0.0014	2.85345	0.45398481	0.749367246	-0.295

cg08045301	II	37	16	71887487	ATXN1L;ATXN1L	0.5	0.00432	2.36443	0.50007278	0.204990603	0.295
cg22968622	I	37	17	43663579		0.46	0.01197	1.92207	0.32996119	0.035294996	0.295
cg11151395	I	37	17	56355299	MPO	0.58	0.00041	3.39184	0.61697147	0.912271187	-0.295
cg21221263	II	37	2	134933179		0.58	0.00041	3.39184	0.46648587	0.7604808	-0.294
cg18918831	II	37	3	195489782	MUC4;MUC4;MUC4	0.58	0.00041	3.39184	0.48023193	0.774300785	-0.294
cg14284211	II	37	6	35570224	FKBP5;FKBP5;FKBP5	0.46	0.01197	1.92207	0.35884254	0.653081931	-0.294
cg01612883	II	37	12	125023115		0.62	0.0001	3.98291	0.39367867	0.687770061	-0.294
cg02999224	II	37	14	23284559	SLC7A7;SLC7A7;SLC	0.58	0.00041	3.39184	0.40331178	0.697073779	-0.294
cg26326621	I	37	14	77332983	C14orf166B	0.54	0.0014	2.85345	0.49891745	0.793009903	-0.294
cg06823060	II	37	16	81616874	CMIP;CMIP	0.58	0.00041	3.39184	0.59032106	0.884369444	-0.294
cg04592811	II	37	17	16200581	PIGL	0.62	0.0001	3.98291	0.54355339	0.837507179	-0.294
cg17837191	II	37	17	62318673	TEX2	0.62	0.0001	3.98291	0.51356655	0.807621096	-0.294
cg05350607	II	37	1	151777000	LINGO4	0.5	0.00432	2.36443	0.51873275	0.22541921	0.293
cg20941184	II	37	1	209878178	HSD11B1;HSD11B1	0.58	0.00041	3.39184	0.4576274	0.750276454	-0.293
cg14397918	II	37	9	72078829	APBA1	0.54	0.0014	2.85345	0.51723926	0.810528033	-0.293
cg23867673	II	37	10	73404170	CDH23;CDH23	0.54	0.0014	2.85345	0.59343622	0.300796235	0.293
cg08867471	II	37	5	56738244		0.5	0.00432	2.36443	0.63694859	0.344928659	0.292
cg19480385	II	37	7	47078738		0.5	0.00432	2.36443	0.54844623	0.256712315	0.292
cg17099048	II	37	8	139734443	COL22A1	0.54	0.0014	2.85345	0.57105702	0.279082361	0.292
cg26443127	II	37	10	31987230		0.54	0.0014	2.85345	0.46792892	0.759577945	-0.292
cg26434370	II	37	16	10909361	FAM18A	0.58	0.00041	3.39184	0.52410142	0.815727084	-0.292
cg03448915	I	37	16	66583078	TK2	0.58	0.00041	3.39184	0.5980072	0.30564701	0.292
cg22911650	II	37	19	51645423	SIGLEC7;SIGLEC7	0.5	0.00432	2.36443	0.48496526	0.77724479	-0.292
cg25198049	I	37	1	151509723	CGN	0.58	0.00041	3.39184	0.43781673	0.728616792	-0.291
cg15607142	II	37	3	128420513		0.58	0.00041	3.39184	0.39998873	0.690715322	-0.291
cg04533116	II	37	5	1169063		0.54	0.0014	2.85345	0.40308828	0.694226804	-0.291
cg18888137	II	37	6	32945759	BRD2;BRD2	0.62	0.0001	3.98291	0.54178618	0.832433837	-0.291
cg03179542	II	37	7	2647333	IQCE;IQCE	0.62	0.0001	3.98291	0.42825245	0.718881346	-0.291
cg03622263	II	37	9	126101308		0.54	0.0014	2.85345	0.47618422	0.184828934	0.291
cg13549277	II	37	12	6659520	IFFO1;IFFO1;IFFO1	0.71	4.57E-06	5.34042	0.44898773	0.740302975	-0.291
cg26783127	II	37	17	79128918	AATK	0.62	0.0001	3.98291	0.56485322	0.85568429	-0.291
cg18659081	II	37	22	44588350	PARVG;PARVG;PAR	0.54	0.0014	2.85345	0.44556067	0.736085926	-0.291
cg17254229	II	37	2	121336713		0.46	0.01197	1.92207	0.58482204	0.294440529	0.29

cg09866565	II	37	3	137844436	A4GNT	0.58	0.00041	3.39184	0.43625158	0.725756791	-0.29
cg01618151	II	37	6	35707957	C6orf81	0.62	0.0001	3.98291	0.50014329	0.790182204	-0.29
cg19396666	II	37	10	125866280		0.54	0.0014	2.85345	0.54151761	0.251239812	0.29
cg24126698	II	37	13	114839662	RASA3	0.67	2.34E-05	4.63072	0.4687669	0.758515626	-0.29
cg27565966	II	37	16	28943198	CD19	0.54	0.0014	2.85345	0.68331845	0.392914839	0.29
cg09712234	II	37	16	30614413		0.58	0.00041	3.39184	0.47327404	0.763409682	-0.29
cg18351781	II	37	19	4950888	UHRF1;UHRF1	0.58	0.00041	3.39184	0.48850779	0.778754929	-0.29
cg17435831	II	37	1	2082522	PRKCZ;PRKCZ;PRKC	0.58	0.00041	3.39184	0.40744473	0.696829493	-0.289
cg13443938	I	37	1	4087214		0.58	0.00041	3.39184	0.5590851	0.270039271	0.289
cg03860768	II	37	8	11351571	BLK;BLK	0.46	0.01197	1.92207	0.56695949	0.277798405	0.289
cg21243944	I	37	9	137118148		0.54	0.0014	2.85345	0.55851293	0.847461257	-0.289
cg15481493	II	37	11	35133274		0.58	0.00041	3.39184	0.5343718	0.823384502	-0.289
cg12136387	II	37	12	116820108		0.58	0.00041	3.39184	0.37768085	0.66714734	-0.289
cg09907509	I	37	13	37248244	C13orf36;C13orf36	0.46	0.01197	1.92207	0.11400792	0.403262374	-0.289
cg23054181	II	37	15	99048945	FAM169B	0.58	0.00041	3.39184	0.55974764	0.849160503	-0.289
cg08703231	II	37	16	8738304	C16orf68	0.62	0.0001	3.98291	0.44630449	0.735614138	-0.289
cg08425760	II	37	1	11795897	AGTRAP;AGTRAP;A	0.58	0.00041	3.39184	0.42330668	0.711414706	-0.288
cg26433561	I	37	1	110426090		0.62	0.0001	3.98291	0.60141665	0.889051095	-0.288
cg09736959	II	37	6	31540114	LTA;LTA;LTA	0.54	0.0014	2.85345	0.62138932	0.333375671	0.288
cg01142676	I	37	11	117695591	FXYD2;FXYD2;FXYD	0.46	0.01197	1.92207	0.32100138	0.608574617	-0.288
cg03408945	I	37	16	8738531	C16orf68	0.54	0.0014	2.85345	0.46516	0.753450881	-0.288
cg06323049	II	37	16	28943094	CD19	0.46	0.01197	1.92207	0.6227683	0.334997671	0.288
cg02138358	II	37	17	56358318	MPO	0.62	0.0001	3.98291	0.38734018	0.675349706	-0.288
cg19573490	II	37	17	79870317	PCYT2;SIRT7	0.5	0.00432	2.36443	0.45178454	0.739541399	-0.288
cg06578434	II	37	19	1155225	SBNO2	0.58	0.00041	3.39184	0.45702611	0.744733068	-0.288
cg00578614	II	37	6	30070403		0.58	0.00041	3.39184	0.43837	0.72510648	-0.287
cg00288598	I	37	8	141555295	EIF2C2;EIF2C2	0.54	0.0014	2.85345	0.58986208	0.876612189	-0.287
cg08594651	I	37	11	47415397		0.71	4.57E-06	5.34042	0.37526925	0.662258578	-0.287
cg18316498	I	37	11	111250196	POU2AF1	0.58	0.00041	3.39184	0.44492994	0.157831696	0.287
cg20055861	II	37	15	68055293	MAP2K5;MAP2K5	0.54	0.0014	2.85345	0.53709657	0.250201453	0.287
cg04204452	II	37	17	1479213	SLC43A2	0.58	0.00041	3.39184	0.40190815	0.689299342	-0.287
cg04266202	II	37	17	56352895	MPO	0.54	0.0014	2.85345	0.55983381	0.847073753	-0.287
cg02092514	I	37	17	80873656	TBCD	0.54	0.0014	2.85345	0.79208708	0.505451229	0.287

cg03199996	II	37	20	49211573	FAM65C	0.5	0.00432	2.36443	0.55194268	0.265344779	0.287
cg03842822	II	37	5	177740106	COL23A1	0.67	2.34E-05	4.63072	0.53131568	0.817079733	-0.286
cg02402436	I	37	6	31540051	LTA;LTA;LTA	0.71	4.57E-06	5.34042	0.4652239	0.179703842	0.286
cg00995368	II	37	6	32627885	HLA-DQB1	0.5	0.00432	2.36443	0.57143157	0.285116226	0.286
cg12182708	II	37	10	99223744	MMS19	0.62	0.0001	3.98291	0.54253923	0.828939977	-0.286
cg24500630	II	37	11	110436181		0.62	0.0001	3.98291	0.46572868	0.751897903	-0.286
cg14066207	I	37	14	73030666	RGS6	0.5	0.00432	2.36443	0.58563328	0.299940607	0.286
cg06172871	II	37	16	72088244	HP;HP	0.62	0.0001	3.98291	0.43725676	0.723098455	-0.286
cg20875821	II	37	18	61557735	SERPINB2;SERPINB2	0.62	0.0001	3.98291	0.53545756	0.82173986	-0.286
cg20429981	II	37	1	1609972	CDK11B;CDK11B;CD	0.54	0.0014	2.85345	0.50601501	0.220541773	0.285
cg22694271	II	37	1	109823096	PSRC1;PSRC1;PSRC1	0.58	0.00041	3.39184	0.39351435	0.678999332	-0.285
cg10667895	II	37	2	102577876		0.58	0.00041	3.39184	0.43602491	0.721109358	-0.285
cg14306819	II	37	2	128052889	ERCC3	0.62	0.0001	3.98291	0.49613868	0.781613865	-0.285
cg06710259	II	37	5	55775841		0.58	0.00041	3.39184	0.53964158	0.254663801	0.285
cg17333291	II	37	7	1126326	GPER;C7orf50;C7or	0.54	0.0014	2.85345	0.56673421	0.281621537	0.285
cg18084554	II	37	19	929046	ARID3A	0.62	0.0001	3.98291	0.40336797	0.688619493	-0.285
cg05327789	I	37	20	61303264	SLCO4A1	0.54	0.0014	2.85345	0.47009722	0.754895837	-0.285
cg06812844	II	37	21	45773782	TRPM2	0.62	0.0001	3.98291	0.36589886	0.650846856	-0.285
cg22337407	I	37	1	11046185		0.46	0.01197	1.92207	0.60854785	0.325036358	0.284
cg06984156	II	37	1	20929640	CDA	0.62	0.0001	3.98291	0.43829961	0.722170234	-0.284
cg22084410	II	37	3	51987688		0.62	0.0001	3.98291	0.40451152	0.688301231	-0.284
cg00488091	II	37	6	144536326		0.54	0.0014	2.85345	0.43027652	0.713899732	-0.284
cg06687504	I	37	6	167263454	RPS6KA2	0.5	0.00432	2.36443	0.55803659	0.273603469	0.284
cg02088996	II	37	7	41817771	LOC285954	0.46	0.01197	1.92207	0.30276594	0.586436087	-0.284
cg25838060	II	37	7	139932729		0.58	0.00041	3.39184	0.7157243	0.432215799	0.284
cg09255910	II	37	1	221055790	HLX	0.62	0.0001	3.98291	0.45782706	0.741165239	-0.283
cg17386240	II	37	5	135384080	TGFBI	0.42	0.02991	1.52413	0.80079743	0.51818044	0.283
cg05094429	II	37	6	167536184	CCR6;CCR6	0.54	0.0014	2.85345	0.48706853	0.204089944	0.283
cg10557578	II	37	8	37758453	RAB11FIP1;RAB11F	0.62	0.0001	3.98291	0.50250103	0.785044711	-0.283
cg01598421	I	37	8	134132043	TG	0.54	0.0014	2.85345	0.74664563	0.463342501	0.283
cg08169020	I	37	14	69256888	ZFP36L1	0.75	7.61E-07	6.11857	0.53988182	0.257353345	0.283
cg22175624	I	37	17	80829261	TBCD	0.54	0.0014	2.85345	0.56522841	0.282179148	0.283
cg05891759	II	37	3	121711723	ILDR1	0.5	0.00432	2.36443	0.60322078	0.321109743	0.282

cg15431659	I	37	3	194901357	C3orf21	0.58	0.00041	3.39184	0.53309903	0.815372109	-0.282
cg09362796	I	37	4	3204792	HTT	0.71	4.57E-06	5.34042	0.73962188	0.457381903	0.282
cg25806704	II	37	4	41936863	TMEM33	0.67	2.34E-05	4.63072	0.56809369	0.849856939	-0.282
cg14109551	II	37	6	118903047	C6orf204;C6orf204	0.54	0.0014	2.85345	0.58641858	0.868174639	-0.282
cg06623899	I	37	8	142161840	DENND3	0.58	0.00041	3.39184	0.63175713	0.91409393	-0.282
cg27210863	II	37	10	82299606	SH2D4B;SH2D4B	0.54	0.0014	2.85345	0.532483	0.250507932	0.282
cg18780288	II	37	10	111659903	XPNPEP1;XPNPEP1;	0.5	0.00432	2.36443	0.36631771	0.648080525	-0.282
cg13300301	II	37	11	94279068	FUT4;FUT4	0.62	0.0001	3.98291	0.48502122	0.7673978	-0.282
cg01662869	II	37	16	4730410	MGRN1;MGRN1;M	0.54	0.0014	2.85345	0.37806139	0.659639104	-0.282
cg12162195	II	37	21	46448092		0.42	0.02991	1.52413	0.80083171	0.519190326	0.282
cg03471150	II	37	1	201797198	IPO9	0.67	2.34E-05	4.63072	0.52064456	0.801355189	-0.281
cg25124300	II	37	2	31607761	XDH	0.58	0.00041	3.39184	0.54738952	0.828491676	-0.281
cg12499316	II	37	2	106242236		0.5	0.00432	2.36443	0.56580657	0.285269355	0.281
cg07658508	I	37	4	973177	SLC26A1	0.54	0.0014	2.85345	0.50335414	0.784224527	-0.281
cg04456916	I	37	5	178902699		0.62	0.0001	3.98291	0.43865058	0.719491554	-0.281
cg11480762	II	37	10	73103395	SLC29A3	0.58	0.00041	3.39184	0.52360989	0.804705593	-0.281
cg10694914	II	37	11	111250431	POU2AF1	0.54	0.0014	2.85345	0.51250259	0.231389013	0.281
cg21210041	II	37	17	27443831	MYO18A;MYO18A	0.62	0.0001	3.98291	0.53469498	0.815302899	-0.281
cg15100426	I	37	2	219187432	PNKD;PNKD	0.58	0.00041	3.39184	0.61466282	0.894397091	-0.28
cg13752114	I	37	3	195489708	MUC4;MUC4;MUC4	0.58	0.00041	3.39184	0.67543979	0.955897198	-0.28
cg02095334	II	37	6	33138108	COL11A2;COL11A2;	0.42	0.02991	1.52413	0.58778028	0.308103679	0.28
cg02122920	II	37	6	42883534	PTCRA	0.46	0.01197	1.92207	0.65615589	0.376007283	0.28
cg22881435	II	37	8	37732086	RAB11FIP1;RAB11F	0.54	0.0014	2.85345	0.52101537	0.240577736	0.28
cg03821121	II	37	11	12148288	MICAL2	0.62	0.0001	3.98291	0.3846952	0.664314873	-0.28
cg19279310	II	37	11	47413087		0.54	0.0014	2.85345	0.35746818	0.637444065	-0.28
cg16235962	II	37	11	118754507	CXCR5	0.62	0.0001	3.98291	0.60331473	0.323690304	0.28
cg05296619	II	37	13	114834683	RASA3	0.58	0.00041	3.39184	0.41407767	0.69421001	-0.28
cg15889847	II	37	14	65183532	PLEKHG3	0.54	0.0014	2.85345	0.41581338	0.696014675	-0.28
cg26217402	I	37	14	74238381	C14orf43	0.58	0.00041	3.39184	0.49743177	0.21773749	0.28
cg21602257	II	37	20	44538675	PLTP;PLTP	0.58	0.00041	3.39184	0.46970018	0.749584735	-0.28
cg01117384	II	37	20	56274096	PMEPA1;PMEPA1;F	0.5	0.00432	2.36443	0.50062354	0.22022516	0.28
cg11986760	II	37	2	112420098		0.67	2.34E-05	4.63072	0.45211534	0.731559631	-0.279
cg14428166	I	37	10	95123753	MYOF;MYOF	0.54	0.0014	2.85345	0.63430813	0.913048604	-0.279

cg12724221	II	37	10	99448480	AVPI1	0.58	0.00041	3.39184	0.52112245	0.800320948	-0.279
cg03174521	II	37	11	3177865	OSBPL5;OSBPL5;OS	0.58	0.00041	3.39184	0.45793723	0.736537624	-0.279
cg21494075	II	37	11	129817717	PRDM10;PRDM10;I	0.5	0.00432	2.36443	0.58557209	0.306764937	0.279
cg19011001	I	37	14	93539613	ITPK1;ITPK1;ITPK1	0.58	0.00041	3.39184	0.44444142	0.723013539	-0.279
cg12058064	II	37	15	86315201	KLHL25;MIR1276	0.67	2.34E-05	4.63072	0.47627249	0.755640956	-0.279
cg04545079	II	37	16	67599924	CTCF	0.5	0.00432	2.36443	0.64118133	0.361980625	0.279
cg20814202	II	37	17	79128885	AATK	0.42	0.02991	1.52413	0.53488063	0.813506889	-0.279
cg01591579	I	37	2	234111145	INPP5D;INPP5D	0.46	0.01197	1.92207	0.74098643	0.462547833	0.278
cg26124526	II	37	5	60618932		0.62	0.0001	3.98291	0.51040369	0.788364829	-0.278
cg23361127	II	37	5	73939283		0.54	0.0014	2.85345	0.54114116	0.819258935	-0.278
cg23097878	II	37	11	45879730	CRY2;CRY2	0.54	0.0014	2.85345	0.57922671	0.857236526	-0.278
cg15823461	I	37	11	60699566	TMEM132A;TMEM	0.54	0.0014	2.85345	0.62065197	0.34266409	0.278
cg04226002	II	37	11	113953462	ZBTB16;ZBTB16	0.58	0.00041	3.39184	0.42930943	0.707122311	-0.278
cg14272075	II	37	17	55122538	RNF126P1	0.71	4.57E-06	5.34042	0.54023694	0.818325703	-0.278
cg13304825	II	37	17	79297548	TMEM105	0.54	0.0014	2.85345	0.44725303	0.725632121	-0.278
cg02481000	II	37	1	2082349	PRKCZ;PRKCZ;PRKC	0.58	0.00041	3.39184	0.38063536	0.658113313	-0.277
cg20452738	II	37	1	226828650	ITPKB	0.46	0.01197	1.92207	0.65228577	0.375059335	0.277
cg00912580	II	37	2	135169533	MGAT5	0.46	0.01197	1.92207	0.51490711	0.238369901	0.277
cg10168494	II	37	3	187635183		0.58	0.00041	3.39184	0.30476683	0.581436754	-0.277
cg16876219	II	37	3	197222311		0.46	0.01197	1.92207	0.61623088	0.339683222	0.277
cg02309230	II	37	6	4612064		0.5	0.00432	2.36443	0.63702271	0.360446884	0.277
cg08678755	II	37	6	131957011	ENPP3	0.42	0.02991	1.52413	0.66100188	0.383805479	0.277
cg03066323	II	37	7	99970779	PILRA;PILRA;PILRA	0.67	2.34E-05	4.63072	0.52963648	0.806864548	-0.277
cg15235922	II	37	8	125678187	MTSS1	0.42	0.02991	1.52413	0.61085901	0.333962519	0.277
cg07825294	II	37	19	1415757	DAZAP1;DAZAP1	0.54	0.0014	2.85345	0.52132973	0.798614891	-0.277
cg17774764	II	37	19	50921551	SPIB	0.46	0.01197	1.92207	0.64254834	0.366019226	0.277
cg11767757	I	37	21	40145404	NCRNA00114	0.58	0.00041	3.39184	0.41399724	0.137098174	0.277
cg25771026	II	37	3	196352027		0.58	0.00041	3.39184	0.46654165	0.742289351	-0.276
cg26348243	I	37	6	31540461	LTA;LTA	0.67	2.34E-05	4.63072	0.47295819	0.197379991	0.276
cg18120259	II	37	6	43894639	LOC100132354	0.58	0.00041	3.39184	0.52829569	0.803846309	-0.276
cg09358725	II	37	11	33914088	LMO2	0.62	0.0001	3.98291	0.40989151	0.68544766	-0.276
cg01890836	I	37	17	17055584	MPRIP;MPRIP	0.54	0.0014	2.85345	0.46009216	0.736188186	-0.276
cg15532942	II	37	18	77220712	NFATC1;NFATC1;NF	0.46	0.01197	1.92207	0.52749146	0.251016884	0.276

cg07168232	II	37	1	209822928	LAMB3;LAMB3;LAN	0.62	0.0001	3.98291	0.36268419	0.637930047	-0.275
cg04722215	I	37	2	97205147	ARID5A	0.67	2.34E-05	4.63072	0.59410834	0.31930546	0.275
cg26701826	II	37	4	108814604	SGMS2;SGMS2;SGN	0.58	0.00041	3.39184	0.52923749	0.80401371	-0.275
cg01715248	II	37	9	93953608		0.5	0.00432	2.36443	0.52980597	0.805246335	-0.275
cg01902758	II	37	11	8816428	ST5;ST5;ST5	0.58	0.00041	3.39184	0.3368491	0.612330364	-0.275
cg00502926	II	37	14	21510056	RNASE7	0.54	0.0014	2.85345	0.44417578	0.719292827	-0.275
cg17812123	II	37	15	101696922		0.5	0.00432	2.36443	0.72570847	0.45072555	0.275
cg03087880	II	37	16	49666012	ZNF423	0.46	0.01197	1.92207	0.57879131	0.303973738	0.275
cg14976569	I	37	19	4667105	C19orf10	0.62	0.0001	3.98291	0.54619004	0.821103224	-0.275
cg03408904	II	37	22	17956462	CECR2	0.62	0.0001	3.98291	0.41614969	0.690840366	-0.275
cg03989987	II	37	2	113885277	IL1RN;IL1RN;IL1RN;	0.5	0.00432	2.36443	0.32714221	0.601045791	-0.274
cg16325984	II	37	2	240291068	HDAC4	0.46	0.01197	1.92207	0.6282881	0.354668439	0.274
cg24700494	I	37	6	30684284	MDC1	0.58	0.00041	3.39184	0.70193255	0.427552722	0.274
cg21248060	I	37	7	1039957	C7orf50;C7orf50;C7	0.54	0.0014	2.85345	0.54834147	0.27455381	0.274
cg15586392	II	37	8	142238691	SLC45A4	0.58	0.00041	3.39184	0.56789725	0.841747508	-0.274
cg22006208	II	37	17	27309169	SEZ6;SEZ6	0.62	0.0001	3.98291	0.49005503	0.764063516	-0.274
cg15309910	I	37	20	23067691	CD93	0.54	0.0014	2.85345	0.39301973	0.666883494	-0.274
cg19510565	II	37	1	31217240	LAPTM5	0.58	0.00041	3.39184	0.57630142	0.303036247	0.273
cg01299332	II	37	5	79406275		0.46	0.01197	1.92207	0.58805851	0.315349314	0.273
cg09109411	II	37	5	149319112	PDE6A	0.54	0.0014	2.85345	0.62141891	0.894112751	-0.273
cg02192746	II	37	7	99971016	PILRA;PILRA;PILRA	0.58	0.00041	3.39184	0.33878148	0.611612348	-0.273
cg04838847	I	37	8	110587155	GOLSYN;GOLSYN;G	0.5	0.00432	2.36443	0.7506627	0.47746223	0.273
cg14557690	II	37	9	139978293	UAP1L1	0.42	0.02991	1.52413	0.51117542	0.237699508	0.273
cg22889918	II	37	10	115024317		0.46	0.01197	1.92207	0.60299895	0.329651512	0.273
cg02525637	II	37	11	2407699	CD81	0.42	0.02991	1.52413	0.64263385	0.369982926	0.273
cg27436118	I	37	16	4729905	MGRN1;MGRN1;M	0.58	0.00041	3.39184	0.58868355	0.861575013	-0.273
cg27461196	II	37	19	35630106	FXYD1;FXYD1	0.58	0.00041	3.39184	0.35979399	0.632338892	-0.273
cg25361506	II	37	1	201426290		0.67	2.34E-05	4.63072	0.48951036	0.761409307	-0.272
cg26444097	II	37	4	2432313		0.58	0.00041	3.39184	0.43375082	0.705598887	-0.272
cg19588519	II	37	10	125817817		0.62	0.0001	3.98291	0.5525584	0.824317937	-0.272
cg09372614	II	37	13	114909276		0.5	0.00432	2.36443	0.55950271	0.28765866	0.272
cg06894612	II	37	14	69042922	RAD51L1	0.58	0.00041	3.39184	0.47075047	0.742788928	-0.272
cg02308232	II	37	17	7742762	KDM6B	0.58	0.00041	3.39184	0.53997132	0.811648181	-0.272

cg12955084	II	37	19	41886265	TMEM91;TMEM91;	0.54	0.0014	2.85345	0.5746673	0.847056099	-0.272
cg21596498	II	37	19	42618407	POU2F2	0.46	0.01197	1.92207	0.68565572	0.413629706	0.272
cg12037947	I	37	20	23067752	CD93	0.58	0.00041	3.39184	0.4549515	0.726802892	-0.272
cg05162166	II	37	22	41684460		0.54	0.0014	2.85345	0.49893554	0.770999175	-0.272
cg09017001	II	37	1	31870508		0.58	0.00041	3.39184	0.45956207	0.730698077	-0.271
cg05350315	II	37	1	32716961	LCK;LCK	0.54	0.0014	2.85345	0.44213775	0.171622003	0.271
cg22610434	II	37	1	158259914	CD1C	0.46	0.01197	1.92207	0.5458814	0.274508009	0.271
cg15287850	II	37	3	186739599	ST6GAL1;ST6GAL1;S	0.46	0.01197	1.92207	0.63849982	0.36759428	0.271
cg06496803	II	37	4	6940915	TBC1D14;TBC1D14	0.5	0.00432	2.36443	0.45043648	0.72138777	-0.271
cg03980424	II	37	10	3526723		0.5	0.00432	2.36443	0.72461193	0.454097167	0.271
cg04682193	II	37	12	121867725	KDM2B;KDM2B	0.54	0.0014	2.85345	0.45483324	0.725798013	-0.271
cg24967811	II	37	12	123503709	PITPNM2	0.54	0.0014	2.85345	0.54673113	0.817802729	-0.271
cg02481950	II	37	16	21665002	METTL9;IGSF6;MET	0.58	0.00041	3.39184	0.50120509	0.772321909	-0.271
cg03739177	I	37	17	27889826	ABHD15	0.54	0.0014	2.85345	0.58680571	0.857961099	-0.271
cg22331200	II	37	17	56355362	MPO	0.58	0.00041	3.39184	0.50255192	0.77358854	-0.271
cg00058291	II	37	1	228974133		0.62	0.0001	3.98291	0.63907029	0.909560711	-0.27
cg24323726	II	37	3	111314186	ZBED2;CD96;CD96	0.46	0.01197	1.92207	0.65201883	0.381523946	0.27
cg13005202	I	37	5	1555844		0.54	0.0014	2.85345	0.37407112	0.64390174	-0.27
cg02084118	I	37	7	971027	ADAP1	0.58	0.00041	3.39184	0.58234257	0.852431415	-0.27
cg19610383	II	37	8	26485445	DPYSL2	0.46	0.01197	1.92207	0.6972015	0.427315859	0.27
cg05755408	II	37	8	27157515	TRIM35	0.5	0.00432	2.36443	0.55119405	0.82116445	-0.27
cg05623815	II	37	10	114602832		0.46	0.01197	1.92207	0.58576795	0.316077678	0.27
cg25311466	II	37	4	6277273	WFS1;WFS1	0.54	0.0014	2.85345	0.5651716	0.296438936	0.269
cg26406292	I	37	7	75292248	HIP1	0.5	0.00432	2.36443	0.55022233	0.280801245	0.269
cg15312943	II	37	11	17843855	SERGEF	0.58	0.00041	3.39184	0.59571065	0.865200643	-0.269
cg16280667	II	37	11	118754593	CXCR5;CXCR5	0.58	0.00041	3.39184	0.61184468	0.342602114	0.269
cg26385126	I	37	12	124912021	NCOR2;NCOR2	0.62	0.0001	3.98291	0.61427179	0.883200711	-0.269
cg01869288	II	37	15	31523257		0.46	0.01197	1.92207	0.5691493	0.299698472	0.269
cg09201499	I	37	16	88838113	FAM38A	0.62	0.0001	3.98291	0.70501197	0.435767214	0.269
cg22396850	II	37	17	41121153	AARSD1;AARSD1;A/	0.54	0.0014	2.85345	0.41355777	0.682582644	-0.269
cg17432627	II	37	21	40124277	NCRNA00114;NCRN	0.54	0.0014	2.85345	0.4759207	0.745296628	-0.269
cg16664584	II	37	1	94454989		0.5	0.00432	2.36443	0.45408517	0.722114649	-0.268
cg10702418	II	37	7	157090031		0.67	2.34E-05	4.63072	0.50858073	0.776607325	-0.268

cg26841425	II	37	10	45958771	MARCH8;MARCH8;	0.54	0.0014	2.85345	0.54562123	0.813625342	-0.268
cg21359303	II	37	10	106068573		0.67	2.34E-05	4.63072	0.70642907	0.438649778	0.268
cg23600372	II	37	12	56036796		0.54	0.0014	2.85345	0.55009778	0.818433481	-0.268
cg02787852	I	37	16	27414536	IL21R;IL21R;IL21R	0.79	1.06E-07	6.97389	0.66845539	0.400599676	0.268
cg05778424	II	37	17	55169508	AKAP1	0.58	0.00041	3.39184	0.44095725	0.70924052	-0.268
cg00471371	II	37	18	74114728	ZNF516	0.62	0.0001	3.98291	0.58892948	0.856705183	-0.268
cg24940706	II	37	19	1648682	TCF3;TCF3	0.5	0.00432	2.36443	0.60012907	0.332086338	0.268
cg19835973	I	37	19	54746613	LILRA6;LILRA6	0.5	0.00432	2.36443	0.55797007	0.825486591	-0.268
cg20070090	II	37	1	153363489	S100A8	0.54	0.0014	2.85345	0.48417683	0.751054006	-0.267
cg07495389	II	37	2	27233872	MAPRE3	0.54	0.0014	2.85345	0.46859172	0.735751074	-0.267
cg02053451	II	37	2	47922605		0.46	0.01197	1.92207	0.68120117	0.41414396	0.267
cg02863947	II	37	3	119499190	NR1I2;NR1I2	0.58	0.00041	3.39184	0.53770937	0.805059803	-0.267
cg12636435	II	37	5	149867965		0.58	0.00041	3.39184	0.42118927	0.6881194	-0.267
cg18664915	II	37	7	1043861	C7orf50;C7orf50;C7	0.46	0.01197	1.92207	0.58149369	0.314473902	0.267
cg20748065	II	37	7	75583421	POR	0.54	0.0014	2.85345	0.26589692	0.532767424	-0.267
cg26247508	II	37	10	82299669	SH2D4B;SH2D4B	0.5	0.00432	2.36443	0.57865276	0.311844018	0.267
cg16846069	II	37	12	6462681	SCNN1A;SCNN1A;SC	0.58	0.00041	3.39184	0.45359124	0.720480503	-0.267
cg16398451	II	37	12	51593266	POU6F1;POU6F1	0.54	0.0014	2.85345	0.50218322	0.769382917	-0.267
cg03963853	I	37	16	4732369	MGRN1;MGRN1;M	0.58	0.00041	3.39184	0.64066141	0.907891019	-0.267
cg04686354	II	37	17	73261880	MRPS7	0.67	2.34E-05	4.63072	0.50389127	0.771292427	-0.267
cg25470197	II	37	22	17598511	CECR6;CECR6;CECR	0.67	2.34E-05	4.63072	0.31738281	0.583924772	-0.267
cg14919455	II	37	6	7167468	RREB1;RREB1;RREB	0.58	0.00041	3.39184	0.54230555	0.808462764	-0.266
cg10411221	II	37	6	32904317	HLA-DMB	0.58	0.00041	3.39184	0.48926063	0.754778558	-0.266
cg14168009	II	37	7	3082006	CARD11	0.58	0.00041	3.39184	0.68682369	0.420693227	0.266
cg10396713	II	37	8	602097		0.62	0.0001	3.98291	0.54144088	0.806971982	-0.266
cg05720871	II	37	8	110587264	GOLSYN;GOLSYN;G	0.54	0.0014	2.85345	0.68662175	0.420782282	0.266
cg14087413	II	37	9	35848330	TMEM8B;TMEM8B	0.5	0.00432	2.36443	0.43299661	0.167385185	0.266
cg03580065	II	37	10	99183360		0.58	0.00041	3.39184	0.42905092	0.694993612	-0.266
cg04468741	II	37	11	12181467	MICAL2	0.62	0.0001	3.98291	0.56315728	0.829581509	-0.266
cg14331899	I	37	11	61125360	CYBASC3;CYBASC3;	0.54	0.0014	2.85345	0.68379561	0.417598279	0.266
cg16941122	I	37	13	99965936	UBAC2;UBAC2;UBA	0.54	0.0014	2.85345	0.46064115	0.726691079	-0.266
cg12873119	II	37	20	23067771	CD93	0.54	0.0014	2.85345	0.47830515	0.744357263	-0.266
cg07206827	II	37	1	151778404	LINGO4	0.46	0.01197	1.92207	0.54348901	0.27889533	0.265

cg22848646	II	37	1	203171499		0.54	0.0014	2.85345	0.5098915	0.774792042	-0.265
cg23010507	I	37	5	1555887		0.5	0.00432	2.36443	0.46308056	0.727725435	-0.265
cg01610123	I	37	5	134770100		0.54	0.0014	2.85345	0.46957947	0.734357149	-0.265
cg16182757	II	37	6	126658808		0.62	0.0001	3.98291	0.52812113	0.792693786	-0.265
cg09797463	II	37	10	90995297	LIPA;LIPA	0.62	0.0001	3.98291	0.55054881	0.815474152	-0.265
cg26578983	I	37	16	85075249	KIAA0513	0.54	0.0014	2.85345	0.61928939	0.883968371	-0.265
cg11377047	II	37	1	26881009	MIR1976;RPS6KA1;	0.5	0.00432	2.36443	0.40944096	0.673730226	-0.264
cg27128761	II	37	5	151055650	SPARC	0.62	0.0001	3.98291	0.50986427	0.774046142	-0.264
cg10462778	II	37	5	172314133	ERGIC1	0.62	0.0001	3.98291	0.55846411	0.822048501	-0.264
cg11460110	I	37	6	30530458	PRR3;PRR3	0.5	0.00432	2.36443	0.55384953	0.817876797	-0.264
cg05569131	II	37	6	36665620		0.58	0.00041	3.39184	0.3568093	0.620520117	-0.264
cg00920892	II	37	8	37732224	RAB11FIP1;RAB11F	0.54	0.0014	2.85345	0.47662035	0.21263153	0.264
cg19222784	II	37	11	20044428	NAV2;NAV2;NAV2;I	0.54	0.0014	2.85345	0.59640114	0.332651899	0.264
cg01117339	II	37	11	73737124		0.5	0.00432	2.36443	0.42749561	0.691956966	-0.264
cg20446824	II	37	12	125076771		0.58	0.00041	3.39184	0.43085631	0.694539401	-0.264
cg15070897	II	37	14	23284968	SLC7A7;SLC7A7;SLC	0.46	0.01197	1.92207	0.40913884	0.672675734	-0.264
cg14827807	II	37	1	150943828	LASS2;LASS2	0.62	0.0001	3.98291	0.38010471	0.643065756	-0.263
cg06706156	II	37	1	230183251		0.58	0.00041	3.39184	0.536923	0.799900156	-0.263
cg11608958	II	37	2	207836769		0.62	0.0001	3.98291	0.56550135	0.828800163	-0.263
cg22387323	II	37	4	24975717	CCDC149	0.58	0.00041	3.39184	0.50210311	0.765475344	-0.263
cg16586807	II	37	7	2948081	CARD11	0.46	0.01197	1.92207	0.73915743	0.47615872	0.263
cg19089701	I	37	8	126398731		0.42	0.02991	1.52413	0.37290893	0.635836987	-0.263
cg02376282	I	37	11	1543206	HCCA2	0.67	2.34E-05	4.63072	0.69105505	0.42792385	0.263
cg07920381	II	37	12	132303685		0.54	0.0014	2.85345	0.4716215	0.734677561	-0.263
cg00813162	I	37	14	69443362	ACTN1;ACTN1;ACTI	0.54	0.0014	2.85345	0.53476415	0.797899857	-0.263
cg07780528	II	37	19	35630334	FXYD1;FXYD1	0.58	0.00041	3.39184	0.30155543	0.564659769	-0.263
cg08939394	II	37	1	17614144		0.54	0.0014	2.85345	0.35109357	0.613268902	-0.262
cg04084354	II	37	1	56721795		0.5	0.00432	2.36443	0.4212029	0.682855026	-0.262
cg19131667	II	37	3	17236940	TBC1D5;TBC1D5;TE	0.42	0.02991	1.52413	0.578748	0.316526301	0.262
cg25025545	II	37	6	14136628	CD83;CD83	0.54	0.0014	2.85345	0.56029437	0.822143186	-0.262
cg09379497	II	37	6	30624467	DHX16;DHX16	0.5	0.00432	2.36443	0.54713983	0.809469905	-0.262
cg26959655	II	37	8	103379529	UBR5	0.54	0.0014	2.85345	0.60777233	0.870176199	-0.262
cg20851559	II	37	8	135613822	ZFAT;ZFAT;ZFAT	0.54	0.0014	2.85345	0.78201432	0.520259908	0.262

cg15511120	II	37	11	6598119		0.58	0.00041	3.39184	0.41478369	0.676705922	-0.262
cg08670658	II	37	11	67052992	ADRBK1	0.58	0.00041	3.39184	0.5693882	0.830926854	-0.262
cg07943111	I	37	17	1492285	SLC43A2	0.67	2.34E-05	4.63072	0.5223543	0.784519719	-0.262
cg03762242	II	37	17	9940004	GAS7;GAS7;GAS7	0.62	0.0001	3.98291	0.36993947	0.632207622	-0.262
cg03919657	II	37	17	48502312	ACSF2	0.71	4.57E-06	5.34042	0.40057064	0.66294452	-0.262
cg07564563	II	37	19	3548977	C19orf28;C19orf28	0.58	0.00041	3.39184	0.49019554	0.752318924	-0.262
cg27160007	II	37	19	41225374	ITPKC	0.58	0.00041	3.39184	0.58860558	0.326950056	0.262
cg21524538	I	37	20	1310846	SDCBP2	0.58	0.00041	3.39184	0.68898567	0.426962344	0.262
cg25724895	II	37	20	58630390	C20orf197	0.54	0.0014	2.85345	0.37076707	0.633162922	-0.262
cg17451941	II	37	1	236254344		0.67	2.34E-05	4.63072	0.50198054	0.7630177	-0.261
cg05551003	II	37	3	11267072	HRH1;HRH1;HRH1	0.67	2.34E-05	4.63072	0.55042206	0.811557995	-0.261
cg19090437	II	37	4	38080799	TBC1D1	0.54	0.0014	2.85345	0.37729195	0.638016683	-0.261
cg22185977	I	37	5	1518133	LPCAT1	0.62	0.0001	3.98291	0.52727693	0.788731681	-0.261
cg05856556	II	37	7	150104705	LOC728743	0.46	0.01197	1.92207	0.59675885	0.336054972	0.261
cg04764812	II	37	10	76582315		0.58	0.00041	3.39184	0.42032144	0.680845153	-0.261
cg04421348	II	37	10	82299737	SH2D4B;SH2D4B	0.46	0.01197	1.92207	0.56246143	0.301959816	0.261
cg20180364	II	37	10	94448532	HHEX	0.54	0.0014	2.85345	0.37335216	0.63421025	-0.261
cg09020213	II	37	13	114810096	RASA3	0.62	0.0001	3.98291	0.34848635	0.609967059	-0.261
cg26318872	II	37	14	104158729	KLC1;KLC1	0.42	0.02991	1.52413	0.67776129	0.416315113	0.261
cg06257110	II	37	16	21658497	METTL9;IGSF6;MET	0.67	2.34E-05	4.63072	0.75792457	0.497159109	0.261
cg20497635	II	37	17	998504	ABR;ABR;ABR	0.54	0.0014	2.85345	0.41031369	0.671699788	-0.261
cg13241751	II	37	17	49009658		0.62	0.0001	3.98291	0.55720885	0.817737964	-0.261
cg08472008	II	37	4	3531603	LRPAP1	0.5	0.00432	2.36443	0.48340058	0.743118163	-0.26
cg24211388	II	37	6	31582837	AIF1;AIF1;AIF1	0.58	0.00041	3.39184	0.41805674	0.677795616	-0.26
cg16809457	II	37	6	90399677	MDN1	0.54	0.0014	2.85345	0.58386025	0.843778282	-0.26
cg19708055	I	37	6	168045556		0.46	0.01197	1.92207	0.63667652	0.377112507	0.26
cg11051139	II	37	12	52580428	KRT80;KRT80	0.58	0.00041	3.39184	0.48832185	0.748025624	-0.26
cg23466769	II	37	13	114801307	RASA3	0.58	0.00041	3.39184	0.55855502	0.818999708	-0.26
cg11341011	II	37	17	2632132		0.5	0.00432	2.36443	0.70654248	0.446967182	0.26
cg01160079	II	37	17	64782413	PRKCA;MIR634	0.5	0.00432	2.36443	0.72562697	0.465748454	0.26
cg23463608	I	37	19	2607757	GNG7	0.62	0.0001	3.98291	0.6276547	0.367833785	0.26
cg16185947	II	37	19	48689918		0.5	0.00432	2.36443	0.65142827	0.390994457	0.26
cg25898577	I	37	22	22292241	PPM1F	0.5	0.00432	2.36443	0.56664432	0.826517194	-0.26

cg19404444	II	37	1	2164602	SKI	0.58	0.00041	3.39184	0.5133332	0.772205797	-0.259
cg09131339	II	37	1	109914235	SORT1	0.54	0.0014	2.85345	0.38401411	0.64265445	-0.259
cg09154256	II	37	2	65352897	RAB1A;RAB1A	0.58	0.00041	3.39184	0.5165808	0.775943741	-0.259
cg24678502	II	37	2	242139599	ANO7	0.54	0.0014	2.85345	0.51364461	0.772265997	-0.259
cg26923863	I	37	4	1221838	CTBP1;CTBP1	0.5	0.00432	2.36443	0.26778825	0.52666807	-0.259
cg21794222	I	37	6	167536063	CCR6;CCR6	0.62	0.0001	3.98291	0.5855624	0.326403941	0.259
cg06436185	I	37	7	151442351	PRKAG2;PRKAG2	0.5	0.00432	2.36443	0.52480596	0.783991692	-0.259
cg02137956	II	37	7	157092982		0.58	0.00041	3.39184	0.52820874	0.786958713	-0.259
cg19069360	II	37	12	1922058	CACNA2D4	0.62	0.0001	3.98291	0.49107976	0.750062708	-0.259
cg20909017	II	37	12	54805345	ITGA5	0.54	0.0014	2.85345	0.35346751	0.612966472	-0.259
cg25708790	I	37	12	132549361	EP400	0.62	0.0001	3.98291	0.54986134	0.291309683	0.259
cg10548805	II	37	14	101538070		0.5	0.00432	2.36443	0.5830143	0.324278988	0.259
cg19030682	I	37	17	79218991	SLC38A10	0.67	2.34E-05	4.63072	0.61908004	0.359956668	0.259
cg21837189	II	37	1	226085836		0.54	0.0014	2.85345	0.55268666	0.294842908	0.258
cg10275766	II	37	2	173591150	LOC91149	0.58	0.00041	3.39184	0.44525651	0.703052903	-0.258
cg13742400	I	37	2	225639708	DOCK10	0.58	0.00041	3.39184	0.60939263	0.867762999	-0.258
cg11236746	II	37	3	33096255	GLB1;GLB1;GLB1	0.58	0.00041	3.39184	0.37882884	0.636413804	-0.258
cg16684117	I	37	5	148808456	MIR143;LOC72826	0.62	0.0001	3.98291	0.39295643	0.65049411	-0.258
cg07507446	II	37	6	29928362		0.62	0.0001	3.98291	0.53450805	0.792433756	-0.258
cg01404750	II	37	13	113493939	ATP11A;ATP11A	0.46	0.01197	1.92207	0.67368307	0.41605216	0.258
cg24777950	II	37	14	25046121	CTSG	0.46	0.01197	1.92207	0.46206908	0.720312068	-0.258
cg08363067	II	37	16	16170085	ABCC1;ABCC1;ABC	0.62	0.0001	3.98291	0.35353874	0.612025688	-0.258
cg15286847	II	37	16	84690433	KLHL36	0.5	0.00432	2.36443	0.55241599	0.294688021	0.258
cg10732094	II	37	19	45957466		0.54	0.0014	2.85345	0.41199623	0.669644871	-0.258
cg04159878	II	37	1	110179127		0.58	0.00041	3.39184	0.45878482	0.716250467	-0.257
cg15591803	II	37	1	111733668	DENND2D	0.58	0.00041	3.39184	0.51523583	0.77263929	-0.257
cg17173896	I	37	4	24975754	CCDC149	0.58	0.00041	3.39184	0.48434191	0.741648355	-0.257
cg01360627	I	37	6	31544931	TNF	0.62	0.0001	3.98291	0.72842305	0.471527292	0.257
cg14533068	I	37	6	158507953	SYNJ2	0.62	0.0001	3.98291	0.66033814	0.91684151	-0.257
cg03567939	II	37	7	151506147	PRKAG2;PRKAG2	0.75	7.61E-07	6.11857	0.53711725	0.794462949	-0.257
cg18444544	II	37	8	22852741	RHOBTB2;RHOBTB	0.54	0.0014	2.85345	0.46733723	0.723979181	-0.257
cg02853355	II	37	16	85132373	FAM92B	0.54	0.0014	2.85345	0.5729168	0.830281995	-0.257
cg15576517	I	37	19	7711153	STXBP2;STXBP2	0.54	0.0014	2.85345	0.63009907	0.886922523	-0.257

cg17907003	II	37	1	117533414		0.62	0.0001	3.98291	0.58551634	0.841517138	-0.256
cg12073436	I	37	1	206958014		0.5	0.00432	2.36443	0.72187592	0.466166985	0.256
cg00483217	II	37	2	25321917	EFR3B	0.58	0.00041	3.39184	0.54300809	0.798881976	-0.256
cg15058645	II	37	2	175528343	WIPF1	0.62	0.0001	3.98291	0.36151144	0.617130336	-0.256
cg23275644	II	37	5	10457079	ROPN1L	0.58	0.00041	3.39184	0.56612917	0.822322593	-0.256
cg11125805	II	37	5	150678162	SLC36A3;SLC36A3	0.58	0.00041	3.39184	0.50424495	0.760630899	-0.256
cg19849557	I	37	6	30619242	C6orf136;C6orf136	0.42	0.02991	1.52413	0.61346766	0.357943424	0.256
cg20741105	II	37	7	43686889	C7orf44	0.46	0.01197	1.92207	0.57387475	0.317479814	0.256
cg01058360	II	37	7	151442371	PRKAG2;PRKAG2	0.5	0.00432	2.36443	0.57466322	0.830921118	-0.256
cg06303635	II	37	10	45958759	MARCH8;MARCH8;	0.54	0.0014	2.85345	0.56987554	0.826247525	-0.256
cg01039876	II	37	15	43559308	TGM5;TGM5	0.5	0.00432	2.36443	0.39226478	0.648050392	-0.256
cg02668248	I	37	19	16437789	KLF2	0.54	0.0014	2.85345	0.4733826	0.217055224	0.256
cg01261013	II	37	21	37691747	MORC3	0.67	2.34E-05	4.63072	0.59451997	0.850649743	-0.256
cg19356022	II	37	1	154943932	SHC1;SHC1;SHC1;SH	0.58	0.00041	3.39184	0.43165376	0.686811079	-0.255
cg22626169	II	37	6	6890951		0.54	0.0014	2.85345	0.52362249	0.778907141	-0.255
cg22164009	II	37	6	14729030		0.62	0.0001	3.98291	0.47402038	0.728560864	-0.255
cg13534791	II	37	9	35042344	C9orf131;C9orf131	0.62	0.0001	3.98291	0.41489028	0.669745823	-0.255
cg14317609	I	37	9	36986006	PAX5	0.46	0.01197	1.92207	0.20769521	0.462517019	-0.255
cg24861866	II	37	10	135106684	TUBGCP2	0.62	0.0001	3.98291	0.4991677	0.754443295	-0.255
cg18072687	II	37	11	2406272	CD81	0.46	0.01197	1.92207	0.69281245	0.438012219	0.255
cg25068347	II	37	11	128343784	ETS1;ETS1;ETS1	0.58	0.00041	3.39184	0.67465522	0.419944571	0.255
cg27255275	II	37	11	129766154	NFRKB	0.54	0.0014	2.85345	0.46725209	0.722498669	-0.255
cg05900955	II	37	13	114918509		0.67	2.34E-05	4.63072	0.5773328	0.832737721	-0.255
cg18568335	II	37	14	105792505	PACS2;PACS2	0.54	0.0014	2.85345	0.54507342	0.800211561	-0.255
cg23083936	II	37	19	41915452	BCKDHA;BCKDHA	0.62	0.0001	3.98291	0.48413046	0.73958033	-0.255
cg08599437	II	37	22	28033286		0.5	0.00432	2.36443	0.57628665	0.321283883	0.255
cg22932427	II	37	1	155615664		0.62	0.0001	3.98291	0.48236832	0.735945085	-0.254
cg17935536	II	37	2	60755743	BCL11A;BCL11A;BC	0.58	0.00041	3.39184	0.41263071	0.666719048	-0.254
cg18275512	II	37	3	10479218	ATP2B2;ATP2B2	0.5	0.00432	2.36443	0.50059478	0.247030524	0.254
cg10426370	II	37	5	54282459	ESM1;ESM1	0.54	0.0014	2.85345	0.55511483	0.301343288	0.254
cg25463483	II	37	6	30530544	PRR3;PRR3	0.62	0.0001	3.98291	0.50073423	0.754299689	-0.254
cg10476003	II	37	6	31540169	LTA;LTA;LTA	0.71	4.57E-06	5.34042	0.5485509	0.294462195	0.254
cg17622855	II	37	6	157982358	ZDHHC14;ZDHHC14	0.5	0.00432	2.36443	0.60615085	0.352536618	0.254

cg02420480	II	37	7	41919892		0.58	0.00041	3.39184	0.45898282	0.712826745	-0.254
cg06257058	II	37	7	99683264		0.54	0.0014	2.85345	0.54625412	0.800701531	-0.254
cg26208815	II	37	7	158263277	PTPRN2;PTPRN2;PT	0.58	0.00041	3.39184	0.6011826	0.855616044	-0.254
cg22249612	I	37	12	56121485	CD63;CD63	0.42	0.02991	1.52413	0.57821516	0.831900133	-0.254
cg05554494	II	37	16	3306685	MEFV	0.67	2.34E-05	4.63072	0.4208388	0.674868346	-0.254
cg01394781	II	37	16	16214213	ABCC1;ABCC1;ABC(	0.71	4.57E-06	5.34042	0.43422835	0.688378465	-0.254
cg21122199	II	37	17	58499720	C17orf64	0.58	0.00041	3.39184	0.46073435	0.714828444	-0.254
cg02147126	II	37	19	827715	AZU1	0.5	0.00432	2.36443	0.43093269	0.685196176	-0.254
cg16959758	II	37	20	36796513		0.58	0.00041	3.39184	0.38765268	0.641891455	-0.254
cg03005293	II	37	1	151805241	RORC	0.54	0.0014	2.85345	0.35259546	0.605923393	-0.253
cg16481332	II	37	7	2654053	IQCE;IQCE	0.58	0.00041	3.39184	0.45472075	0.707930563	-0.253
cg07231045	II	37	8	142219444		0.5	0.00432	2.36443	0.54174809	0.794930636	-0.253
cg22406869	II	37	11	66276941	DPP3;BBS1;DPP3	0.58	0.00041	3.39184	0.42075948	0.673778529	-0.253
cg26164712	II	37	11	118754565	CXCR5;CXCR5	0.5	0.00432	2.36443	0.60755882	0.354840238	0.253
cg23526087	II	37	14	68973466	RAD51L1	0.54	0.0014	2.85345	0.51634384	0.263004691	0.253
cg20503109	II	37	17	74565795	ST6GALNAC2	0.67	2.34E-05	4.63072	0.40776301	0.660625065	-0.253
cg07996532	II	37	1	21620812	ECE1	0.54	0.0014	2.85345	0.41345776	0.161708462	0.252
cg17833106	II	37	1	161060000	PVRL4	0.62	0.0001	3.98291	0.55179266	0.804170729	-0.252
cg24595152	II	37	3	43733831	ABHD5	0.54	0.0014	2.85345	0.46122697	0.712883792	-0.252
cg07493874	I	37	5	1342172	CLPTM1L	0.42	0.02991	1.52413	0.70555137	0.453773578	0.252
cg26004235	II	37	6	30656582	KIAA1949;NRM;KIA	0.5	0.00432	2.36443	0.5115559	0.259370647	0.252
cg04499011	II	37	6	36972127	FGD2	0.5	0.00432	2.36443	0.58839879	0.33601596	0.252
cg13844341	II	37	7	2116368	MAD1L1;MAD1L1;M	0.67	2.34E-05	4.63072	0.59732199	0.849467096	-0.252
cg08132858	II	37	7	101361395		0.54	0.0014	2.85345	0.39306242	0.645346574	-0.252
cg04188920	I	37	10	3138534	PFKP	0.54	0.0014	2.85345	0.36207957	0.61404379	-0.252
cg23615741	I	37	10	101297642		0.54	0.0014	2.85345	0.37051501	0.622488073	-0.252
cg08908247	II	37	11	66055170	YIF1A	0.71	4.57E-06	5.34042	0.38074386	0.632945138	-0.252
cg08163918	II	37	12	57628654	SHMT2;SHMT2;SHN	0.54	0.0014	2.85345	0.68056584	0.428695183	0.252
cg18294707	II	37	14	21422965	RNASE2	0.5	0.00432	2.36443	0.45493002	0.707404981	-0.252
cg02240622	II	37	15	40601467	PLCB2	0.54	0.0014	2.85345	0.48471085	0.736394996	-0.252
cg08684477	II	37	16	87521410	ZCCHC14	0.67	2.34E-05	4.63072	0.42626199	0.678651718	-0.252
cg26535158	I	37	17	40175841	NKIRAS2;NKIRAS2;N	0.54	0.0014	2.85345	0.46618737	0.71787422	-0.252
cg16732654	II	37	17	79129022	AATK	0.46	0.01197	1.92207	0.49618032	0.747682819	-0.252

cg00291213	II	37	21	36398056	RUNX1	0.54	0.0014	2.85345	0.44730832	0.699362715		-0.252
cg11798406	II	37	21	40145361	NCRNA00114	0.54	0.0014	2.85345	0.67890978	0.426546239		0.252
cg26574610	II	37	22	24095989	VPREB3	0.5	0.00432	2.36443	0.61752195	0.365222454		0.252
cg00261690	II	37	1	28856281	SNHG3-RCC1;SNHG	0.54	0.0014	2.85345	0.63284509	0.883498677		-0.251
cg02799411	II	37	3	4794061	ITPR1;ITPR1;ITPR1;	0.46	0.01197	1.92207	0.39224169	0.64367363		-0.251
cg12239580	II	37	3	43073058	FAM198A	0.5	0.00432	2.36443	0.50620343	0.757128916		-0.251
cg06012804	II	37	3	193849318		0.5	0.00432	2.36443	0.52233425	0.271609853		0.251
cg12534645	II	37	5	149636295	CAMK2A;CAMK2A	0.5	0.00432	2.36443	0.52872271	0.277549727		0.251
cg03922146	I	37	6	30656499	KIAA1949;NRM;KIA	0.42	0.02991	1.52413	0.72149545	0.470035172		0.251
cg02217713	II	37	7	643155	PRKAR1B;PRKAR1B	0.5	0.00432	2.36443	0.50881644	0.75945558		-0.251
cg03152353	II	37	9	139417194	NOTCH1	0.58	0.00041	3.39184	0.56792581	0.81847301		-0.251
cg19975346	II	37	10	126366131	FAM53B	0.54	0.0014	2.85345	0.57604893	0.826973095		-0.251
cg03509949	II	37	12	56236869	MMP19	0.54	0.0014	2.85345	0.51184304	0.763141983		-0.251
cg02212339	I	37	17	3493666	TRPV1;TRPV1;TRPV	0.54	0.0014	2.85345	0.62194754	0.371210566		0.251
cg25282976	I	37	17	79005047	FLJ90757	0.46	0.01197	1.92207	0.492825	0.743545658		-0.251
cg01367992	II	37	1	160766535	LY9;LY9	0.62	0.0001	3.98291	0.76489541	0.514453163		0.25
cg22409100	II	37	2	40658918	SLC8A1;SLC8A1;SLC	0.58	0.00041	3.39184	0.402817	0.652671013		-0.25
cg18406852	I	37	2	101010769	CHST10	0.62	0.0001	3.98291	0.7070657	0.45686718		0.25
cg19114543	II	37	3	46996484	CCDC12	0.58	0.00041	3.39184	0.52753058	0.777851564		-0.25
cg10307548	II	37	4	24795830	SOD3	0.54	0.0014	2.85345	0.37659663	0.626587773		-0.25
cg18446110	II	37	5	138714654	SLC23A1;SLC23A1	0.71	4.57E-06	5.34042	0.48111856	0.730712364		-0.25
cg13500819	II	37	5	138725400	MGC29506	0.54	0.0014	2.85345	0.46223258	0.212032029		0.25
cg05978527	II	37	6	30656692	NRM;KIAA1949	0.5	0.00432	2.36443	0.58556391	0.335594314		0.25
cg12481212	II	37	6	44020245		0.58	0.00041	3.39184	0.43877323	0.688758837		-0.25
cg15241519	I	37	11	121440892	SORL1	0.5	0.00432	2.36443	0.58251291	0.332204482		0.25
cg04440811	II	37	11	129817712	PRDM10;PRDM10;I	0.5	0.00432	2.36443	0.57345627	0.323913512		0.25
cg22491680	II	37	12	96389547	HAL	0.54	0.0014	2.85345	0.53599978	0.786319545		-0.25
cg18825221	II	37	14	68749962	RAD51L1;RAD51L1;	0.5	0.00432	2.36443	0.36808017	0.118362117		0.25
cg10377921	II	37	19	15391946	BRD4;BRD4	0.54	0.0014	2.85345	0.51806436	0.768134412		-0.25
cg01078772	II	37	20	30920486	KIF3B	0.54	0.0014	2.85345	0.78305154	0.532928001		0.25
cg15553418	II	37	1	16696547	C1orf144;C1orf144	0.58	0.00041	3.39184	0.52559809	0.774429803		-0.249
cg21301505	I	37	1	159796629	SLAMF8;SLAMF8	0.5	0.00432	2.36443	0.43920131	0.687888351		-0.249
cg13172153	II	37	1	201391747	TNNI1	0.5	0.00432	2.36443	0.61575867	0.366586653		0.249

cg00534022	II	37	2	8686087		0.62	0.0001	3.98291	0.34783167	0.596434533	-0.249
cg10499974	II	37	3	46244099	CCR1	0.54	0.0014	2.85345	0.58202516	0.831329242	-0.249
cg08548882	II	37	6	150953085	PLEKHG1	0.54	0.0014	2.85345	0.59164854	0.342404946	0.249
cg15248035	II	37	9	36169949	CCIN	0.5	0.00432	2.36443	0.55471619	0.803360079	-0.249
cg13603332	II	37	9	108053809	SLC44A1	0.58	0.00041	3.39184	0.51377309	0.762451853	-0.249
cg14482811	I	37	9	139652837	LCN8	0.54	0.0014	2.85345	0.6189967	0.369601231	0.249
cg13761364	II	37	9	140325278	NOXA1	0.46	0.01197	1.92207	0.6270399	0.378499241	0.249
cg26583584	II	37	11	75291101		0.42	0.02991	1.52413	0.56932422	0.320384972	0.249
cg18728264	I	37	11	118766491	CXCR5;CXCR5;CXCR	0.58	0.00041	3.39184	0.50958811	0.260807441	0.249
cg10363118	II	37	12	116948928		0.46	0.01197	1.92207	0.90665301	0.657825511	0.249
cg01574390	II	37	16	21623651	METTL9;METTL9	0.54	0.0014	2.85345	0.46385194	0.7133413	-0.249
cg08255481	II	37	16	88103035	BANP;BANP	0.54	0.0014	2.85345	0.49722683	0.247970865	0.249
cg02052762	II	37	17	4090525	ANKFY1	0.58	0.00041	3.39184	0.62102952	0.869607933	-0.249
cg19418318	II	37	19	17219073	MYO9B;MYO9B	0.58	0.00041	3.39184	0.46443504	0.713826924	-0.249
cg05307452	II	37	2	89128093		0.5	0.00432	2.36443	0.59455296	0.346113168	0.248
cg27572072	II	37	7	958244	ADAP1	0.58	0.00041	3.39184	0.48613759	0.733692151	-0.248
cg07609372	II	37	7	150885361	ASB10;ASB10;ASB1	0.46	0.01197	1.92207	0.56353101	0.315330067	0.248
cg22325580	II	37	8	1899943	ARHGEF10	0.58	0.00041	3.39184	0.40429983	0.651911623	-0.248
cg11307715	II	37	8	142161887	DENND3	0.58	0.00041	3.39184	0.588753	0.836828211	-0.248
cg17995557	II	37	10	126289971	LHPP;LHPP	0.46	0.01197	1.92207	0.59230783	0.344597191	0.248
cg20491695	II	37	11	45719826		0.5	0.00432	2.36443	0.66736518	0.419573815	0.248
cg16706613	II	37	12	52423671		0.46	0.01197	1.92207	0.69924524	0.451138318	0.248
cg15354065	II	37	12	56327217	DGKA;DGKA;DGKA;	0.54	0.0014	2.85345	0.47156247	0.719899811	-0.248
cg09540612	I	37	20	62492233	C20orf135	0.58	0.00041	3.39184	0.4329159	0.680430994	-0.248
cg00992048	II	37	1	1695585	NADK	0.54	0.0014	2.85345	0.48273724	0.729372784	-0.247
cg21118486	II	37	6	167263225	RPS6KA2	0.62	0.0001	3.98291	0.71019106	0.463367161	0.247
cg16523158	II	37	6	167535171	CCR6;CCR6	0.54	0.0014	2.85345	0.68274134	0.435690867	0.247
cg07733719	II	37	7	75683547	MDH2	0.54	0.0014	2.85345	0.51412116	0.761530412	-0.247
cg14255337	II	37	12	121972602	KDM2B;KDM2B	0.54	0.0014	2.85345	0.58890872	0.342347464	0.247
cg15174682	I	37	19	5132063	KDM4B	0.58	0.00041	3.39184	0.58167133	0.335014579	0.247
cg19358608	II	37	20	1924565		0.54	0.0014	2.85345	0.58671071	0.833245275	-0.247
cg05753799	II	37	20	3746315	C20orf27	0.58	0.00041	3.39184	0.40118254	0.648624256	-0.247
cg16296829	II	37	1	154832535	KCNN3;KCNN3	0.5	0.00432	2.36443	0.46924297	0.223727457	0.246

cg24830898	I	37	5	177964873	COL23A1	0.62	0.0001	3.98291	0.61182167	0.858241879	-0.246
cg26575105	II	37	6	109010960		0.67	2.34E-05	4.63072	0.57738132	0.822944207	-0.246
cg13410614	II	37	9	136341915	SLC2A6;SLC2A6	0.58	0.00041	3.39184	0.38119304	0.626845328	-0.246
cg20283498	II	37	10	73387917	CDH23;CDH23	0.54	0.0014	2.85345	0.37986027	0.62541933	-0.246
cg22241838	II	37	11	60680006	TMEM109	0.62	0.0001	3.98291	0.59941573	0.353232069	0.246
cg08447520	II	37	12	113513026	DTX1	0.46	0.01197	1.92207	0.53056886	0.28454478	0.246
cg00905101	II	37	16	1988798	SEPX1	0.62	0.0001	3.98291	0.40719938	0.653023265	-0.246
cg00867406	I	37	16	89997889	TUBB3	0.54	0.0014	2.85345	0.73505234	0.489182931	0.246
cg06559756	II	37	17	65464297	PITPNC1;PITPNC1	0.54	0.0014	2.85345	0.53473426	0.289073493	0.246
cg18406924	I	37	17	78701257	RPTOR;RPTOR	0.5	0.00432	2.36443	0.67247116	0.426572247	0.246
cg13367406	II	37	1	41481683	SLFNL1;SLFNL1	0.46	0.01197	1.92207	0.58715744	0.341986713	0.245
cg20570464	II	37	1	42144398	HIVEP3;HIVEP3	0.54	0.0014	2.85345	0.51903689	0.273689544	0.245
cg19269039	II	37	1	111743200	DENND2D;DENND2	0.54	0.0014	2.85345	0.40140166	0.646137766	-0.245
cg03730703	II	37	1	160173841	PEA15	0.54	0.0014	2.85345	0.66781246	0.42330439	0.245
cg20513777	II	37	2	238533299		0.62	0.0001	3.98291	0.49221876	0.737615369	-0.245
cg11308319	II	37	2	240291426	HDAC4	0.54	0.0014	2.85345	0.49551378	0.250210912	0.245
cg05931054	II	37	6	11654646		0.54	0.0014	2.85345	0.61897985	0.373627723	0.245
cg16906346	I	37	6	24646782	KIAA0319;KIAA031	0.46	0.01197	1.92207	0.53301733	0.288261676	0.245
cg07880727	II	37	7	47846424	C7orf69;PKD1L1	0.54	0.0014	2.85345	0.42631588	0.671734871	-0.245
cg16301004	I	37	10	106082537	ITPRIP	0.46	0.01197	1.92207	0.32335531	0.568814339	-0.245
cg22498365	II	37	17	80829028	TBCD	0.5	0.00432	2.36443	0.5721614	0.327381783	0.245
cg06655349	II	37	19	10332165	S1PR2	0.62	0.0001	3.98291	0.47811129	0.723114109	-0.245
cg02231062	II	37	22	42321087	TNFRSF13C	0.5	0.00432	2.36443	0.65890638	0.413527329	0.245
cg03961551	II	37	1	25251730	RUNX3;RUNX3	0.62	0.0001	3.98291	0.43971879	0.195932396	0.244
cg00359365	II	37	1	26880547	MIR1976;RPS6KA1;	0.46	0.01197	1.92207	0.31992462	0.564275336	-0.244
cg05962781	II	37	1	115599145	TSPAN2	0.67	2.34E-05	4.63072	0.45598072	0.70006708	-0.244
cg08621778	I	37	1	202102420	ARL8A	0.62	0.0001	3.98291	0.56029939	0.804690518	-0.244
cg07223106	II	37	1	233155919	PCNXL2	0.54	0.0014	2.85345	0.53985474	0.783559845	-0.244
cg11879354	II	37	6	159075773	SYTL3	0.58	0.00041	3.39184	0.38623952	0.630062889	-0.244
cg12002745	II	37	8	124179875		0.58	0.00041	3.39184	0.35834907	0.602780189	-0.244
cg08687540	I	37	11	1890204	LSP1;LSP1;LSP1	0.54	0.0014	2.85345	0.50090954	0.2569863	0.244
cg00832329	II	37	14	105860985	PACS2;PACS2	0.58	0.00041	3.39184	0.52140994	0.765122857	-0.244
cg16727774	I	37	16	87958281	CA5A	0.54	0.0014	2.85345	0.60182787	0.357610363	0.244

cg13618516	II	37	17	79129078	AATK	0.5	0.00432	2.36443	0.61191138	0.85584372	-0.244
cg22505006	II	37	1	154981829	ZBTB7B	0.54	0.0014	2.85345	0.49490513	0.737407176	-0.243
cg20847110	II	37	4	39482781	LOC401127	0.5	0.00432	2.36443	0.54777667	0.790936155	-0.243
cg08961187	II	37	6	37648275	MDGA1	0.46	0.01197	1.92207	0.60565079	0.362831725	0.243
cg01357222	II	37	6	137619143		0.58	0.00041	3.39184	0.47049328	0.713235238	-0.243
cg15453708	II	37	7	151493647	PRKAG2;PRKAG2	0.62	0.0001	3.98291	0.5547518	0.798243711	-0.243
cg25268422	II	37	9	136659313	VAV2;VAV2	0.46	0.01197	1.92207	0.616764	0.373954527	0.243
cg07986469	II	37	10	129795003	PTPRE	0.54	0.0014	2.85345	0.53405359	0.777402572	-0.243
cg24049888	I	37	11	111250129	POU2AF1;POU2AF1	0.67	2.34E-05	4.63072	0.35123542	0.10831583	0.243
cg09586924	II	37	11	130032815	ST14	0.54	0.0014	2.85345	0.36368114	0.606183593	-0.243
cg13371705	II	37	12	2452955	CACNA1C;CACNA1C	0.54	0.0014	2.85345	0.48494224	0.727552881	-0.243
cg12024104	II	37	12	6662581	IFFO1;IFFO1	0.62	0.0001	3.98291	0.49528413	0.738273114	-0.243
cg11688093	II	37	13	111178359	RAB20	0.46	0.01197	1.92207	0.49089783	0.247836936	0.243
cg23327851	II	37	14	21423684	RNASE2;RNASE2	0.62	0.0001	3.98291	0.42228117	0.665185984	-0.243
cg16565031	II	37	14	70186289		0.54	0.0014	2.85345	0.39518664	0.638194642	-0.243
cg06100973	II	37	19	852114	ELANE	0.5	0.00432	2.36443	0.4188517	0.661595048	-0.243
cg19021236	II	37	22	18479382	MICAL3;MICAL3;MI	0.62	0.0001	3.98291	0.45340277	0.210143351	0.243
cg04430911	II	37	1	36914349	OSCP1;OSCP1	0.58	0.00041	3.39184	0.55844943	0.800649504	-0.242
cg18455616	II	37	2	99124870	INPP4A;INPP4A;INF	0.54	0.0014	2.85345	0.56871017	0.810403584	-0.242
cg21554588	II	37	5	138720378	SLC23A1;SLC23A1	0.54	0.0014	2.85345	0.65673119	0.414469996	0.242
cg22794304	II	37	5	169734762		0.5	0.00432	2.36443	0.34814984	0.590431191	-0.242
cg00409658	II	37	6	138744549	NHSL1;NHSL1	0.67	2.34E-05	4.63072	0.34188049	0.584367815	-0.242
cg13509702	II	37	8	144896307	SCRIB;SCRIB;MIR93	0.54	0.0014	2.85345	0.53846901	0.780076726	-0.242
cg01462537	II	37	10	2992189		0.5	0.00432	2.36443	0.8014089	0.559896451	0.242
cg25170017	I	37	11	64644487	EHD1	0.54	0.0014	2.85345	0.6958882	0.454312237	0.242
cg10283505	II	37	11	94278912	FUT4;FUT4	0.58	0.00041	3.39184	0.55223077	0.794573771	-0.242
cg25616055	II	37	12	89638890		0.42	0.02991	1.52413	0.39970014	0.641503416	-0.242
cg20666585	II	37	15	31523186		0.5	0.00432	2.36443	0.57019218	0.327952645	0.242
cg02135245	II	37	17	998711	ABR;ABR;ABR	0.62	0.0001	3.98291	0.49560422	0.7376487	-0.242
cg25134567	I	37	17	64782369	PRKCA;MIR634	0.62	0.0001	3.98291	0.46292841	0.220765187	0.242
cg04946561	II	37	19	4902899	ARRDC5	0.58	0.00041	3.39184	0.51399289	0.755653376	-0.242
cg00104562	II	37	19	7767229	FCER2	0.5	0.00432	2.36443	0.62100609	0.378819226	0.242
cg21201401	I	37	20	62367884	LIME1	0.58	0.00041	3.39184	0.32716082	0.084854471	0.242

cg13698860	II	37	22	43525679	BIK	0.54	0.0014	2.85345	0.44366157	0.685286839	-0.242
cg01588592	II	37	1	157069325	ETV3L;ETV3L	0.67	2.34E-05	4.63072	0.50987583	0.751157905	-0.241
cg24686551	II	37	3	134047274		0.58	0.00041	3.39184	0.50418409	0.745678455	-0.241
cg01393945	I	37	5	10457034	ROPN1L	0.67	2.34E-05	4.63072	0.67025918	0.911566022	-0.241
cg13831136	II	37	6	30070342		0.62	0.0001	3.98291	0.39271477	0.634093946	-0.241
cg06196379	II	37	6	41254885	TREM1	0.54	0.0014	2.85345	0.38513248	0.625782432	-0.241
cg21350697	I	37	10	134149366	LRRC27;LRRC27;LRI	0.5	0.00432	2.36443	0.37121228	0.6126349	-0.241
cg27597505	II	37	12	54148217		0.58	0.00041	3.39184	0.63390088	0.393228237	0.241
cg09841788	II	37	14	65213478	SPTB	0.58	0.00041	3.39184	0.45981838	0.700816405	-0.241
cg02226192	II	37	16	89461734	ANKRD11	0.54	0.0014	2.85345	0.53825693	0.779063771	-0.241
cg20682376	II	37	17	16875542	TNFRSF13B	0.5	0.00432	2.36443	0.57966082	0.338985008	0.241
cg27196880	II	37	17	40570233	PTRF	0.54	0.0014	2.85345	0.40533794	0.646563922	-0.241
cg12122241	II	37	20	1920407	SIRPA;SIRPA;SIRPA	0.54	0.0014	2.85345	0.52601324	0.766847968	-0.241
cg16348358	II	37	1	32731477	LCK	0.58	0.00041	3.39184	0.54646257	0.78638779	-0.24
cg10718614	II	37	1	234914480		0.54	0.0014	2.85345	0.363316	0.603327909	-0.24
cg14016418	II	37	2	65065975		0.62	0.0001	3.98291	0.46742327	0.707421319	-0.24
cg03681335	II	37	2	108903965	SULT1C2;SULT1C2	0.5	0.00432	2.36443	0.54939851	0.789319701	-0.24
cg16785938	II	37	4	10022984	SLC2A9;SLC2A9	0.58	0.00041	3.39184	0.41450745	0.654574138	-0.24
cg21513254	I	37	6	30624502	DHX16;DHX16	0.58	0.00041	3.39184	0.47112623	0.710753896	-0.24
cg17540192	I	37	7	97875259	TECPR1	0.54	0.0014	2.85345	0.6403372	0.880489394	-0.24
cg25932290	II	37	15	89939252	LOC254559	0.58	0.00041	3.39184	0.41784171	0.65815013	-0.24
cg06484100	II	37	17	41799087		0.67	2.34E-05	4.63072	0.48510324	0.724765729	-0.24
cg02018291	II	37	17	65487462	PITPNC1;PITPNC1	0.58	0.00041	3.39184	0.56066882	0.800663927	-0.24
cg03318469	II	37	1	3710303	LRRC47	0.67	2.34E-05	4.63072	0.32730769	0.566508979	-0.239
cg08456420	II	37	1	39570175	MACF1	0.58	0.00041	3.39184	0.49787119	0.736969316	-0.239
cg22164207	II	37	1	152383842	CRNN	0.5	0.00432	2.36443	0.62302991	0.384005546	0.239
cg12129117	I	37	1	223940656	CAPN2;CAPN2	0.58	0.00041	3.39184	0.51949077	0.758970801	-0.239
cg17954142	II	37	1	230417096	GALNT2	0.5	0.00432	2.36443	0.66430295	0.425340103	0.239
cg08622198	II	37	1	239979505	CHRM3	0.54	0.0014	2.85345	0.51947843	0.758763759	-0.239
cg13325231	I	37	5	1342170	CLPTM1L	0.46	0.01197	1.92207	0.68717616	0.448423547	0.239
cg12684668	II	37	5	150403466	GPX3	0.58	0.00041	3.39184	0.57795636	0.816653972	-0.239
cg11898695	II	37	6	42883504	PTCRA	0.46	0.01197	1.92207	0.65310144	0.414117497	0.239
cg01877450	II	37	7	97915802	BRI3;BRI3	0.62	0.0001	3.98291	0.41280179	0.651637804	-0.239

cg18132262	I	37	7	99971238	PILRA;PILRA;PILRA;	0.58	0.00041	3.39184	0.33681542	0.576180452	-0.239
cg06889975	II	37	8	22558722		0.46	0.01197	1.92207	0.61949232	0.38063679	0.239
cg06449094	II	37	11	822397	PNPLA2	0.58	0.00041	3.39184	0.58693227	0.825795127	-0.239
cg18415791	II	37	11	3178024	OSBPL5;OSBPL5;OS	0.54	0.0014	2.85345	0.56040966	0.799374648	-0.239
cg03626208	II	37	12	2443169	CACNA1C;CACNA1(	0.54	0.0014	2.85345	0.46238068	0.701748032	-0.239
cg02582213	II	37	16	89441654	ANKRD11	0.46	0.01197	1.92207	0.73366462	0.494577453	0.239
cg07353685	II	37	1	153536278	S100A2	0.58	0.00041	3.39184	0.53058319	0.768322066	-0.238
cg06987246	II	37	7	6659785	ZNF853	0.71	4.57E-06	5.34042	0.51461884	0.276827108	0.238
cg12894711	II	37	7	43821949	BLVRA	0.58	0.00041	3.39184	0.48545834	0.723411865	-0.238
cg26541218	II	37	7	47826387	PKD1L1	0.58	0.00041	3.39184	0.46556223	0.703396235	-0.238
cg25115537	I	37	8	123801352	ZHX2	0.46	0.01197	1.92207	0.44181898	0.20365859	0.238
cg00697658	II	37	8	145808575	KIAA1688	0.54	0.0014	2.85345	0.56335692	0.325681721	0.238
cg15964132	I	37	11	3175636	OSBPL5;OSBPL5;OS	0.54	0.0014	2.85345	0.57645217	0.814702463	-0.238
cg05949397	II	37	11	44976423		0.58	0.00041	3.39184	0.52767732	0.765295694	-0.238
cg11268190	II	37	12	6442526	TNFRSF1A	0.5	0.00432	2.36443	0.42302389	0.660882435	-0.238
cg09365924	II	37	12	53611399	RARG;RARG	0.58	0.00041	3.39184	0.35826483	0.595819199	-0.238
cg06317209	II	37	12	58210878	AVIL	0.58	0.00041	3.39184	0.55064927	0.788862779	-0.238
cg04234631	II	37	16	8738441	C16orf68	0.54	0.0014	2.85345	0.51806741	0.756526725	-0.238
cg23582644	II	37	18	61557687	SERPINB2;SERPINB:	0.62	0.0001	3.98291	0.6186232	0.856963445	-0.238
cg22501942	II	37	1	11798278	AGTRAP;AGTRAP;A	0.54	0.0014	2.85345	0.34181431	0.578615695	-0.237
cg07625783	II	37	1	159796617	SLAMF8;SLAMF8	0.54	0.0014	2.85345	0.37606423	0.613493136	-0.237
cg24426483	II	37	2	20738271		0.5	0.00432	2.36443	0.5765875	0.339428841	0.237
cg20954219	II	37	2	29745303	ALK	0.58	0.00041	3.39184	0.34697812	0.584014474	-0.237
cg22485298	II	37	6	30853333	DDR1;DDR1	0.46	0.01197	1.92207	0.47908943	0.241923136	0.237
cg02677635	II	37	9	129099507	FAM125B;FAM125I	0.54	0.0014	2.85345	0.51486318	0.752354391	-0.237
cg11311053	II	37	12	124912049	NCOR2;NCOR2	0.62	0.0001	3.98291	0.58888342	0.826216722	-0.237
cg11792874	II	37	14	24014501		0.62	0.0001	3.98291	0.41934272	0.656499798	-0.237
cg24361265	I	37	15	44068668	ELL3	0.5	0.00432	2.36443	0.58342181	0.346020828	0.237
cg01858712	II	37	16	12156525	SNX29	0.62	0.0001	3.98291	0.32841515	0.56515823	-0.237
cg05433111	II	37	16	28943232	CD19	0.54	0.0014	2.85345	0.4905922	0.253502476	0.237
cg06465011	II	37	16	84860871	CRISPLD2	0.71	4.57E-06	5.34042	0.45149333	0.688970417	-0.237
cg27586417	II	37	16	85577800		0.5	0.00432	2.36443	0.5556141	0.792804793	-0.237
cg09076077	II	37	20	58630315	C20orf197	0.54	0.0014	2.85345	0.4189524	0.65644339	-0.237

cg12299361	II	37	1	53796769		0.62	0.0001	3.98291	0.50226317	0.737978188	-0.236
cg11832395	II	37	5	178057845		0.54	0.0014	2.85345	0.62952217	0.393095255	0.236
cg06366374	II	37	6	6543659	LOC285780	0.5	0.00432	2.36443	0.4181503	0.65419918	-0.236
cg11435441	I	37	6	25027994		0.62	0.0001	3.98291	0.75571848	0.51939288	0.236
cg07076751	II	37	6	30647539	KIAA1949;KIAA194	0.62	0.0001	3.98291	0.62487967	0.389034957	0.236
cg17830204	II	37	7	99819110	GATS;GATS;GATS;G	0.62	0.0001	3.98291	0.38415372	0.147923071	0.236
cg07095530	I	37	7	100859728	ZNHIT1;PLOD3	0.58	0.00041	3.39184	0.56543892	0.801282185	-0.236
cg25526001	II	37	10	85939451	C10orf99	0.62	0.0001	3.98291	0.57791454	0.342375967	0.236
cg21208539	II	37	11	93864142	PANX1	0.54	0.0014	2.85345	0.39712876	0.633321502	-0.236
cg06567290	II	37	11	118900111	SLC37A4;SLC37A4;S	0.46	0.01197	1.92207	0.50176325	0.737971937	-0.236
cg02648589	II	37	13	114877260	RASA3	0.54	0.0014	2.85345	0.62121679	0.857226054	-0.236
cg02178957	II	37	16	85964200		0.46	0.01197	1.92207	0.45699956	0.693081694	-0.236
cg22242539	II	37	17	1665220	SERPINF1	0.46	0.01197	1.92207	0.39726737	0.63370766	-0.236
cg12361262	II	37	17	36571637		0.54	0.0014	2.85345	0.36590519	0.602202913	-0.236
cg11977716	I	37	18	77284742	NFATC1;NFATC1;NF	0.71	4.57E-06	5.34042	0.32124792	0.085324644	0.236
cg14165142	II	37	20	3778655	CDC25B;CDC25B;CD	0.62	0.0001	3.98291	0.52606629	0.28984558	0.236
cg26300857	II	37	1	6142719	KCNAB2;KCNAB2	0.62	0.0001	3.98291	0.54344584	0.778389469	-0.235
cg18770149	II	37	1	149903180	MTMR11;MTMR11	0.58	0.00041	3.39184	0.60204049	0.837259731	-0.235
cg03836615	II	37	3	4856096	ITPR1;ITPR1;ITPR1	0.62	0.0001	3.98291	0.40766323	0.642406302	-0.235
cg21988461	II	37	4	88008667	AFF1;AFF1	0.62	0.0001	3.98291	0.31034533	0.545727984	-0.235
cg02435538	II	37	7	75507337	RHBDD2;RHBDD2	0.67	2.34E-05	4.63072	0.58593224	0.821423672	-0.235
cg04789529	II	37	8	25072319	DOCK5	0.5	0.00432	2.36443	0.4856637	0.720877098	-0.235
cg18457731	II	37	8	139734484	COL22A1	0.46	0.01197	1.92207	0.63180814	0.397179411	0.235
cg13410764	II	37	9	90407888		0.54	0.0014	2.85345	0.40491958	0.639758194	-0.235
cg10381771	II	37	10	97515398	ENTPD1;ENTPD1;EN	0.5	0.00432	2.36443	0.34139839	0.575933222	-0.235
cg03302822	II	37	10	99928067	C10orf28	0.46	0.01197	1.92207	0.59360068	0.359035467	0.235
cg25934954	II	37	11	64567121	MAP4K2	0.5	0.00432	2.36443	0.52501283	0.289820466	0.235
cg23645046	II	37	11	64582244		0.46	0.01197	1.92207	0.55794091	0.323086564	0.235
cg08669096	II	37	11	121229765		0.54	0.0014	2.85345	0.47860574	0.713160083	-0.235
cg02902617	II	37	14	93031178	RIN3	0.58	0.00041	3.39184	0.58890669	0.824076349	-0.235
cg09086087	II	37	16	68000763	SLC12A4;SLC12A4;S	0.67	2.34E-05	4.63072	0.38797663	0.623017599	-0.235
cg27260684	II	37	16	85063742	KIAA0513	0.58	0.00041	3.39184	0.54790822	0.78322778	-0.235
cg22312249	II	37	17	72779428	TMEM104	0.5	0.00432	2.36443	0.57288487	0.807612437	-0.235

cg16308790	I	37	18	77225973	NFATC1;NFATC1;NF	0.5	0.00432	2.36443	0.51065956	0.276069825	0.235
cg23347094	II	37	19	15699028		0.54	0.0014	2.85345	0.51155934	0.746891485	-0.235
cg02231590	II	37	2	231737958	ITM2C;ITM2C;ITM2	0.5	0.00432	2.36443	0.44139037	0.207057738	0.234
cg20541456	II	37	5	156696520	CYFIP2;CYFIP2;CYFI	0.46	0.01197	1.92207	0.27356107	0.50735	-0.234
cg05206633	II	37	5	177913434	COL23A1	0.58	0.00041	3.39184	0.66647817	0.900152996	-0.234
cg21235678	I	37	7	1130967	GPER;C7orf50;GPEI	0.62	0.0001	3.98291	0.58406953	0.817856317	-0.234
cg24292612	II	37	8	6735472	DEFB1;DEFB1	0.62	0.0001	3.98291	0.39825238	0.632223397	-0.234
cg15361215	II	37	10	120873630	FAM45B;FAM45A	0.5	0.00432	2.36443	0.42816611	0.662465061	-0.234
cg24626659	I	37	10	134754286	C10orf93	0.54	0.0014	2.85345	0.83153439	0.597303564	0.234
cg07194984	II	37	11	2128771		0.5	0.00432	2.36443	0.51389785	0.280055486	0.234
cg25137372	II	37	12	96390059	HAL;HAL	0.54	0.0014	2.85345	0.3972378	0.630954656	-0.234
cg17541002	II	37	12	121714889	CAMKK2;CAMKK2;(	0.5	0.00432	2.36443	0.3840179	0.618370346	-0.234
cg00050618	II	37	16	27414418	IL21R;IL21R	0.67	2.34E-05	4.63072	0.73377753	0.499518182	0.234
cg22688566	II	37	17	27459835	MYO18A;MYO18A	0.58	0.00041	3.39184	0.57388679	0.808310349	-0.234
cg07824483	II	37	17	79882042	MAFG;MAFG	0.67	2.34E-05	4.63072	0.45325429	0.68724945	-0.234
cg20450689	I	37	18	77586147		0.5	0.00432	2.36443	0.34556038	0.5791814	-0.234
cg24938761	II	37	19	2091406	MOBKL2A	0.58	0.00041	3.39184	0.38308262	0.617366858	-0.234
cg20513976	I	37	20	62367893	LIME1	0.62	0.0001	3.98291	0.37884206	0.144946839	0.234
cg19273683	II	37	1	21656047	ECE1	0.71	4.57E-06	5.34042	0.62681555	0.393765775	0.233
cg08106792	II	37	1	153509323	S100A6	0.58	0.00041	3.39184	0.37014233	0.602713809	-0.233
cg08476244	II	37	2	120867222	EPB41L5	0.58	0.00041	3.39184	0.36840689	0.601514897	-0.233
cg05146536	II	37	4	77140678		0.58	0.00041	3.39184	0.32786318	0.56098115	-0.233
cg17390918	II	37	5	10502512		0.54	0.0014	2.85345	0.51962139	0.752657882	-0.233
cg00377497	II	37	8	27167110	TRIM35	0.67	2.34E-05	4.63072	0.55700795	0.79032845	-0.233
cg13924015	II	37	9	107911118		0.54	0.0014	2.85345	0.62465119	0.391412911	0.233
cg11276093	II	37	10	95123774	MYOF;MYOF	0.5	0.00432	2.36443	0.53769634	0.770854517	-0.233
cg20636248	II	37	10	121439845		0.62	0.0001	3.98291	0.42179276	0.654636629	-0.233
cg07143125	II	37	11	48110045	PTPRJ;PTPRJ	0.62	0.0001	3.98291	0.53966452	0.772281331	-0.233
cg09802818	II	37	12	52604609	LOC283404	0.5	0.00432	2.36443	0.52067959	0.754162238	-0.233
cg02420027	II	37	13	41632640		0.5	0.00432	2.36443	0.60061706	0.833168439	-0.233
cg04958055	II	37	14	69404437	ACTN1;ACTN1;ACTI	0.58	0.00041	3.39184	0.36445458	0.597668076	-0.233
cg16411445	I	37	21	40145923	NCRNA00114	0.46	0.01197	1.92207	0.67106217	0.43767665	0.233
cg25821399	II	37	3	128997055	C3orf37;C3orf37	0.54	0.0014	2.85345	0.55682591	0.324992519	0.232

cg26714425	II	37	5	10330845		0.58	0.00041	3.39184	0.46280491	0.695232656	-0.232
cg16312968	II	37	6	6900945		0.54	0.0014	2.85345	0.2562688	0.488643373	-0.232
cg04903089	II	37	6	32905190	HLA-DMB	0.5	0.00432	2.36443	0.30243491	0.534212729	-0.232
cg23169883	I	37	10	72435301	ADAMTS14;ADAMT	0.54	0.0014	2.85345	0.71089481	0.942720175	-0.232
cg06154903	I	37	11	64642558	EHD1	0.58	0.00041	3.39184	0.82359166	0.591216999	0.232
cg23140706	II	37	12	54689278	NFE2;NFE2	0.54	0.0014	2.85345	0.49574162	0.727800152	-0.232
cg09552641	II	37	19	55173510	LILRB4;LILRB4	0.5	0.00432	2.36443	0.61637962	0.384226953	0.232
cg17988326	II	37	1	41091703	RIMS3	0.54	0.0014	2.85345	0.53182183	0.301103792	0.231
cg19024599	II	37	2	109886730	SH3RF3	0.54	0.0014	2.85345	0.70663552	0.475638595	0.231
cg01715572	II	37	2	232093057	ARMC9	0.54	0.0014	2.85345	0.4969949	0.265896202	0.231
cg25643229	II	37	5	139120209		0.54	0.0014	2.85345	0.61591977	0.384962693	0.231
cg21473545	II	37	5	178203432	AACSL	0.58	0.00041	3.39184	0.45224953	0.682964425	-0.231
cg22662844	I	37	7	2116648	MAD1L1;MAD1L1;M	0.58	0.00041	3.39184	0.64904408	0.880014097	-0.231
cg25589371	II	37	7	2477790		0.5	0.00432	2.36443	0.66024323	0.429197254	0.231
cg06567596	I	37	7	97832830	LMTK2	0.54	0.0014	2.85345	0.77931784	0.547831364	0.231
cg17222829	II	37	11	70433293	SHANK2;SHANK2	0.5	0.00432	2.36443	0.47288469	0.703832487	-0.231
cg19260718	II	37	11	75989014		0.46	0.01197	1.92207	0.60358577	0.372748717	0.231
cg08830502	II	37	16	87588559		0.54	0.0014	2.85345	0.56637188	0.797480302	-0.231
cg24777505	II	37	17	80829604	TBCD	0.5	0.00432	2.36443	0.62038342	0.389700518	0.231
cg07805542	I	37	1	9779309	PIK3CD	0.46	0.01197	1.92207	0.45817794	0.688455154	-0.23
cg12380764	II	37	1	206971195	IL19	0.58	0.00041	3.39184	0.52060803	0.750947759	-0.23
cg14414903	I	37	2	240171712	HDAC4	0.54	0.0014	2.85345	0.40934065	0.639799798	-0.23
cg13303534	II	37	3	171261809		0.62	0.0001	3.98291	0.49516833	0.725402389	-0.23
cg17304531	II	37	3	197401639	MIR922;KIAA0226;I	0.5	0.00432	2.36443	0.58738753	0.357882209	0.23
cg17229371	II	37	5	1485470	LPCAT1	0.46	0.01197	1.92207	0.56683081	0.33707045	0.23
cg20978247	II	37	6	32905085	HLA-DMB	0.58	0.00041	3.39184	0.61900754	0.849024457	-0.23
cg07507418	II	37	6	42372284	TRERF1	0.5	0.00432	2.36443	0.39401864	0.624345176	-0.23
cg01950058	II	37	7	77044434	PION	0.46	0.01197	1.92207	0.70476473	0.474546403	0.23
cg26426488	II	37	7	100461644	SLC12A9	0.62	0.0001	3.98291	0.34653991	0.577007187	-0.23
cg13413384	II	37	9	137302231	RXRA	0.62	0.0001	3.98291	0.46925941	0.698909219	-0.23
cg25941075	II	37	11	73729854		0.67	2.34E-05	4.63072	0.51220191	0.742150797	-0.23
cg06559575	II	37	12	53490352	IGFBP6	0.62	0.0001	3.98291	0.53434284	0.764760052	-0.23
cg15903956	II	37	15	74676231		0.5	0.00432	2.36443	0.42122821	0.191203821	0.23

cg07102368	II	37	15	76455848	C15orf27	0.54	0.0014	2.85345	0.36876124	0.598561761	-0.23
cg02596819	II	37	16	9045869	USP7	0.54	0.0014	2.85345	0.37230785	0.142405208	0.23
cg05382012	II	37	16	67517841	AGRP;AGRP	0.5	0.00432	2.36443	0.58487462	0.814791327	-0.23
cg22153994	II	37	17	43484399	ARHGAP27;ARHGAI	0.5	0.00432	2.36443	0.55847463	0.328201915	0.23
cg12570429	I	37	17	56345839	LPO;LPO;LPO	0.58	0.00041	3.39184	0.59803225	0.828131784	-0.23
cg22250546	II	37	19	33791370	CEBPA;CEBPA	0.58	0.00041	3.39184	0.41009426	0.640313432	-0.23
cg18920397	II	37	1	160765805	LY9;LY9	0.67	2.34E-05	4.63072	0.45052406	0.221365268	0.229
cg00852924	II	37	1	179061154	TOR3A	0.62	0.0001	3.98291	0.45937108	0.688694494	-0.229
cg27044455	II	37	5	149878183		0.58	0.00041	3.39184	0.58985774	0.818556668	-0.229
cg16079645	II	37	7	1039875	C7orf50;C7orf50;C7	0.46	0.01197	1.92207	0.50137529	0.272364332	0.229
cg17581870	I	37	7	1062965	C7orf50;C7orf50;M	0.58	0.00041	3.39184	0.74127337	0.511958156	0.229
cg23261443	I	37	7	101596340	CUX1;CUX1;CUX1	0.58	0.00041	3.39184	0.44475178	0.67411526	-0.229
cg10589094	II	37	12	6452596	TNFRSF1A	0.46	0.01197	1.92207	0.72372025	0.494254169	0.229
cg12707346	II	37	12	64960957		0.58	0.00041	3.39184	0.49399987	0.722918211	-0.229
cg18817487	II	37	12	96390143	HAL	0.58	0.00041	3.39184	0.20395185	0.432493761	-0.229
cg03568673	II	37	13	20796216	GJB6;GJB6;GJB6;GJ	0.5	0.00432	2.36443	0.59287672	0.821598309	-0.229
cg19903805	II	37	14	92333771	TC2N;TC2N	0.67	2.34E-05	4.63072	0.43611657	0.207175121	0.229
cg05331340	II	37	17	7083064	ASGR1	0.58	0.00041	3.39184	0.48401891	0.712983207	-0.229
cg02779913	II	37	17	40088680	TTC25	0.46	0.01197	1.92207	0.38131002	0.610546541	-0.229
cg01022219	I	37	18	13641735	C18orf1;C18orf1;C1	0.46	0.01197	1.92207	0.5021322	0.730676524	-0.229
cg16606773	I	37	20	19955806	RIN2	0.54	0.0014	2.85345	0.56698793	0.796143597	-0.229
cg16828576	II	37	22	42318379		0.46	0.01197	1.92207	0.47105396	0.241630581	0.229
cg08850169	II	37	1	161171469	NDUFS2;NDUFS2	0.58	0.00041	3.39184	0.52569114	0.753396724	-0.228
cg20806175	II	37	1	161186839	FCER1G	0.46	0.01197	1.92207	0.38302668	0.610693262	-0.228
cg17852326	II	37	1	221055964	HLX	0.54	0.0014	2.85345	0.43169157	0.659808328	-0.228
cg04245131	II	37	1	235133338		0.79	1.06E-07	6.97389	0.56540596	0.793118311	-0.228
cg15700582	II	37	2	68960656	ARHGAP25;ARHGAI	0.67	2.34E-05	4.63072	0.42823307	0.655961896	-0.228
cg22989958	II	37	2	74783039	DOK1	0.54	0.0014	2.85345	0.40154654	0.629573917	-0.228
cg08947915	I	37	4	1742463	TACC3	0.5	0.00432	2.36443	0.57493453	0.803390773	-0.228
cg12094903	II	37	6	32808689	PSMB8;PSMB8	0.54	0.0014	2.85345	0.4274796	0.199887222	0.228
cg23206115	II	37	6	158066900	ZDHHC14;ZDHHC14	0.54	0.0014	2.85345	0.60354291	0.831264661	-0.228
cg18683606	II	37	7	100471612	SRRT;SRRT;SRRT;SR	0.54	0.0014	2.85345	0.56154389	0.789117791	-0.228
cg22826986	II	37	8	11351507	BLK	0.46	0.01197	1.92207	0.52700628	0.298617591	0.228

cg14422498	II	37	9	100639423		0.58	0.00041	3.39184	0.3039193	0.531863753	-0.228
cg20618826	II	37	10	81148896	ZCCHC24	0.46	0.01197	1.92207	0.56640509	0.33800368	0.228
cg06760238	II	37	10	134400036	INPP5A	0.58	0.00041	3.39184	0.34520026	0.573249869	-0.228
cg19733736	II	37	12	56115029	RDH5	0.46	0.01197	1.92207	0.54483737	0.77313617	-0.228
cg03919488	II	37	12	63179691	PPM1H	0.5	0.00432	2.36443	0.49119987	0.718708368	-0.228
cg15545247	II	37	12	123201372	GPR109B;GPR109B	0.54	0.0014	2.85345	0.38300883	0.611113421	-0.228
cg01329756	II	37	17	80346912		0.5	0.00432	2.36443	0.42871068	0.65677773	-0.228
cg20152841	II	37	19	2073190	MOBKL2A	0.58	0.00041	3.39184	0.52229498	0.750481877	-0.228
cg19825589	I	37	19	42379710	CD79A;CD79A	0.67	2.34E-05	4.63072	0.301201	0.072723638	0.228
cg01015663	II	37	1	23729692	TCEA3	0.58	0.00041	3.39184	0.49486998	0.721519379	-0.227
cg23018689	II	37	1	159173540	DARC	0.62	0.0001	3.98291	0.53236513	0.759740061	-0.227
cg07030287	II	37	2	242174797	HDLBP;HDLBP	0.58	0.00041	3.39184	0.37668409	0.603762748	-0.227
cg06677890	II	37	5	149887486	NDST1	0.62	0.0001	3.98291	0.25144751	0.478662791	-0.227
cg16674484	II	37	5	149887497	NDST1	0.5	0.00432	2.36443	0.3669598	0.593975384	-0.227
cg14021478	I	37	6	33384537	CUTA;CUTA;CUTA;C	0.67	2.34E-05	4.63072	0.68199928	0.455010517	0.227
cg04848693	II	37	7	2078786	MAD1L1;MAD1L1;M	0.54	0.0014	2.85345	0.70905898	0.482370336	0.227
cg03995434	II	37	7	141395853	KIAA1147	0.5	0.00432	2.36443	0.67911339	0.451690006	0.227
cg14206140	I	37	9	136303481	ADAMTS13;ADAMT	0.58	0.00041	3.39184	0.68785482	0.460948121	0.227
cg01860774	II	37	14	64969374	ZBTB25	0.62	0.0001	3.98291	0.74571084	0.518923316	0.227
cg23222617	II	37	14	89653143	FOXN3;FOXN3	0.5	0.00432	2.36443	0.5445906	0.317157053	0.227
cg08027123	II	37	16	29296186		0.46	0.01197	1.92207	0.65019505	0.423520254	0.227
cg03309308	II	37	16	68525329		0.58	0.00041	3.39184	0.41018848	0.182705182	0.227
cg03376089	II	37	17	17839409	TOM1L2;TOM1L2	0.58	0.00041	3.39184	0.6029693	0.830240166	-0.227
cg04071967	II	37	17	53511321		0.62	0.0001	3.98291	0.52491634	0.752292086	-0.227
cg01041239	I	37	18	13222581	C18orf1;C18orf1	0.58	0.00041	3.39184	0.41266163	0.639645513	-0.227
cg27064845	II	37	19	48252623	GLTSCR2	0.54	0.0014	2.85345	0.49943717	0.272828345	0.227
cg22458693	II	37	19	55173503	LILRB4;LILRB4	0.5	0.00432	2.36443	0.734109	0.50683685	0.227
cg22874652	II	37	1	1107439		0.58	0.00041	3.39184	0.51116023	0.285163034	0.226
cg13440692	II	37	1	1186357		0.5	0.00432	2.36443	0.65569478	0.429775054	0.226
cg17494034	II	37	1	12513497	VPS13D;VPS13D	0.54	0.0014	2.85345	0.54486324	0.318435111	0.226
cg23202722	II	37	1	33793808	PHC2;PHC2	0.54	0.0014	2.85345	0.49442342	0.720846052	-0.226
cg22119466	II	37	2	9331371		0.54	0.0014	2.85345	0.43020984	0.656399234	-0.226
cg04864807	I	37	2	121412139		0.54	0.0014	2.85345	0.26448057	0.490253006	-0.226

cg20496896	II	37	3	46579532	LRRC2;LRRC2	0.67	2.34E-05	4.63072	0.5502266	0.32468251	0.226
cg23359665	I	37	6	32120907	PPT2;PRRT1;PPT2	0.46	0.01197	1.92207	0.76859753	0.54303236	0.226
cg01702009	II	37	6	42883762	PTCRA;PTCRA	0.5	0.00432	2.36443	0.63373702	0.407489779	0.226
cg00639635	II	37	6	143837891		0.62	0.0001	3.98291	0.54649888	0.77253934	-0.226
cg15177211	II	37	7	75567469	POR	0.5	0.00432	2.36443	0.39505503	0.620605619	-0.226
cg10287786	II	37	11	117666000	DSCAML1	0.67	2.34E-05	4.63072	0.50391017	0.729570432	-0.226
cg17865265	II	37	12	56236845	MMP19	0.62	0.0001	3.98291	0.54708917	0.772817777	-0.226
cg18887769	II	37	14	22945181		0.58	0.00041	3.39184	0.64567416	0.419357171	0.226
cg19905757	II	37	15	68924127	CORO2B	0.46	0.01197	1.92207	0.52061763	0.294327247	0.226
cg00970435	II	37	17	66380327	ARSG	0.46	0.01197	1.92207	0.40918317	0.635461803	-0.226
cg16638580	II	37	19	39890911	MED29	0.5	0.00432	2.36443	0.62829898	0.402717833	0.226
cg05305327	II	37	20	32156436	CBFA2T2;CBFA2T2;	0.5	0.00432	2.36443	0.61412019	0.388120585	0.226
cg00446123	I	37	20	62367888	LIME1	0.67	2.34E-05	4.63072	0.35530816	0.129075053	0.226
cg17571559	II	37	3	11267525	HRH1;HRH1;HRH1	0.58	0.00041	3.39184	0.49991343	0.72494548	-0.225
cg06300880	II	37	3	119279147	CD80	0.46	0.01197	1.92207	0.7662906	0.541530817	0.225
cg25752703	I	37	3	128710390	KIAA1257	0.54	0.0014	2.85345	0.64988541	0.874392377	-0.225
cg23344523	II	37	6	160380572		0.58	0.00041	3.39184	0.5764446	0.801683246	-0.225
cg13650156	II	37	7	99970502	PILRA;PILRA;PILRA	0.58	0.00041	3.39184	0.5322414	0.757086561	-0.225
cg17433678	II	37	10	3135764	PFKP	0.5	0.00432	2.36443	0.51982974	0.745283071	-0.225
cg08181251	II	37	10	99443455	AVPI1	0.42	0.02991	1.52413	0.32367324	0.548387231	-0.225
cg19160629	II	37	11	116742145	SIK3	0.67	2.34E-05	4.63072	0.33939251	0.564127833	-0.225
cg06738887	II	37	15	60688010	ANXA2;ANXA2;ANX	0.5	0.00432	2.36443	0.39429341	0.618944149	-0.225
cg01666796	I	37	15	70364327	TLE3;TLE3;TLE3	0.5	0.00432	2.36443	0.71004549	0.485066083	0.225
cg01735277	II	37	15	75077691	CSK;CSK	0.62	0.0001	3.98291	0.51472256	0.290126428	0.225
cg14023999	I	37	15	90543224		0.54	0.0014	2.85345	0.65990566	0.884502718	-0.225
cg03919650	II	37	16	3306855	MEFV	0.62	0.0001	3.98291	0.3558481	0.581137375	-0.225
cg09577144	I	37	17	76573499	DNAH17	0.54	0.0014	2.85345	0.52522953	0.300046098	0.225
cg02348449	II	37	19	58630429	ZSCAN18;ZSCAN18	0.54	0.0014	2.85345	0.51817514	0.742883104	-0.225
cg22281206	II	37	22	31522176	INPP5J	0.46	0.01197	1.92207	0.60143378	0.376007799	0.225
cg02847588	II	37	1	8271997		0.58	0.00041	3.39184	0.38916476	0.613318594	-0.224
cg24400630	I	37	1	89728035	GBP5;GBP5	0.54	0.0014	2.85345	0.62649323	0.850058778	-0.224
cg00405190	II	37	2	175545838	WIPF1	0.58	0.00041	3.39184	0.61492978	0.839268238	-0.224
cg03025465	II	37	3	141163704	ZBTB38	0.58	0.00041	3.39184	0.56967117	0.793833557	-0.224

cg01211396	II	37	6	30624478	DHX16;DHX16	0.58	0.00041	3.39184	0.57769832	0.801964491	-0.224
cg02445907	II	37	6	36294404	C6orf222	0.5	0.00432	2.36443	0.55771753	0.333892084	0.224
cg15527515	II	37	14	23630709	SLC7A8	0.58	0.00041	3.39184	0.52330899	0.74714056	-0.224
cg01758575	II	37	16	28943288	CD19;CD19	0.5	0.00432	2.36443	0.53274673	0.308430388	0.224
cg06857116	II	37	17	15885326	ZSWIM7;ZSWIM7	0.54	0.0014	2.85345	0.60104772	0.825041096	-0.224
cg23990942	II	37	19	40946878	SERTAD3;SERTAD3	0.58	0.00041	3.39184	0.47927167	0.703121834	-0.224
cg06291107	II	37	20	36157675	BLCAP;BLCAP	0.67	2.34E-05	4.63072	0.51941002	0.743564993	-0.224
cg15078958	II	37	1	10997656		0.54	0.0014	2.85345	0.47463195	0.252080152	0.223
cg00913954	II	37	1	36852956	STK40	0.54	0.0014	2.85345	0.54776665	0.770945468	-0.223
cg15830864	II	37	1	44877749	RNF220	0.58	0.00041	3.39184	0.59614785	0.818862138	-0.223
cg00785482	II	37	1	208058986		0.58	0.00041	3.39184	0.63016209	0.853042338	-0.223
cg05415606	II	37	2	38357299	C2orf58	0.62	0.0001	3.98291	0.3949771	0.618308134	-0.223
cg05758467	II	37	2	240035894	HDAC4	0.58	0.00041	3.39184	0.43904724	0.661948409	-0.223
cg11229273	II	37	3	57015101	ARHGEF3	0.54	0.0014	2.85345	0.33813079	0.560656003	-0.223
cg15612947	II	37	5	14464064	TRIO	0.62	0.0001	3.98291	0.54662492	0.769332389	-0.223
cg05088356	II	37	5	66124167	MAST4;MAST4;MA	0.58	0.00041	3.39184	0.63113142	0.408406136	0.223
cg00060800	II	37	5	141600994		0.46	0.01197	1.92207	0.37956377	0.602588326	-0.223
cg21932542	II	37	7	4850345	RADIL	0.58	0.00041	3.39184	0.56367211	0.786374682	-0.223
cg04088945	II	37	7	157090146		0.58	0.00041	3.39184	0.62194483	0.844679048	-0.223
cg01081737	II	37	8	142238752	SLC45A4	0.54	0.0014	2.85345	0.62995734	0.853339916	-0.223
cg13823257	II	37	9	35666823		0.42	0.02991	1.52413	0.56592871	0.343254166	0.223
cg19726711	II	37	10	6183872		0.58	0.00041	3.39184	0.71659384	0.494029752	0.223
cg08145373	II	37	11	2407008	CD81	0.5	0.00432	2.36443	0.45568248	0.232946936	0.223
cg21720802	II	37	11	66232921	PELI3;PELI3	0.5	0.00432	2.36443	0.55823204	0.335468334	0.223
cg09303642	II	37	12	54690818	NFE2;NFE2	0.54	0.0014	2.85345	0.44324692	0.666690424	-0.223
cg18020065	I	37	13	114829733	RASA3	0.46	0.01197	1.92207	0.21811049	0.441528851	-0.223
cg07102001	II	37	16	87734816	LOC100129637	0.46	0.01197	1.92207	0.53794219	0.314637355	0.223
cg15530560	II	37	19	851290	ELANE	0.67	2.34E-05	4.63072	0.46890053	0.691940617	-0.223
cg27340480	II	37	19	44170526	PLAUR;PLAUR;PLAU	0.54	0.0014	2.85345	0.64302727	0.86576904	-0.223
cg24898863	II	37	1	153363580	S100A8	0.58	0.00041	3.39184	0.32427267	0.546241986	-0.222
cg12275370	II	37	2	10425661		0.67	2.34E-05	4.63072	0.43039447	0.65208085	-0.222
cg27485075	II	37	2	47054963	LOC100134259	0.5	0.00432	2.36443	0.53470751	0.312850104	0.222
cg11963365	II	37	5	141320784	KIAA0141;KIAA014	0.5	0.00432	2.36443	0.5236753	0.745390474	-0.222

cg02393092	II	37	9	131444897	SET	0.46	0.01197	1.92207	0.60094017	0.37908602	0.222
cg07441944	II	37	11	44623459	CD82;CD82	0.54	0.0014	2.85345	0.625433	0.403844869	0.222
cg01336858	II	37	15	70154572		0.5	0.00432	2.36443	0.43122147	0.209450363	0.222
cg00531137	II	37	16	57643932		0.54	0.0014	2.85345	0.33692781	0.11500997	0.222
cg05546038	II	37	16	67207033	NOL3	0.5	0.00432	2.36443	0.62545214	0.847389965	-0.222
cg03778909	II	37	17	80833393	TBCD	0.5	0.00432	2.36443	0.61440281	0.836300269	-0.222
cg21913632	II	37	21	46505130	ADARB1;ADARB1;A	0.58	0.00041	3.39184	0.60614369	0.383948026	0.222
cg01431057	II	37	1	153362927	S100A8	0.58	0.00041	3.39184	0.49271314	0.714025619	-0.221
cg10441691	II	37	5	139520490		0.5	0.00432	2.36443	0.67906094	0.45804752	0.221
cg17416722	II	37	6	32554385	HLA-DRB1	0.54	0.0014	2.85345	0.2786362	0.057593226	0.221
cg06449934	II	37	7	1130697	GPER;C7orf50;GPEF	0.58	0.00041	3.39184	0.47621162	0.697529315	-0.221
cg01618660	I	37	9	100882376	TRIM14;TRIM14;TR	0.46	0.01197	1.92207	0.65414633	0.875458486	-0.221
cg04451770	II	37	10	97515372	ENTPD1;ENTPD1;EN	0.42	0.02991	1.52413	0.36382393	0.58502832	-0.221
cg19126615	II	37	11	47290188	MADD;MADD;NR1H	0.62	0.0001	3.98291	0.5630112	0.783682432	-0.221
cg05436845	II	37	11	65378622	MAP3K11	0.58	0.00041	3.39184	0.36778446	0.588331752	-0.221
cg09244312	II	37	13	42120657		0.58	0.00041	3.39184	0.55221886	0.773703087	-0.221
cg27170268	I	37	14	104171695	XRCC3;XRCC3;XRCC	0.54	0.0014	2.85345	0.44442863	0.665326486	-0.221
cg24595580	II	37	15	100890996	FLJ42289;FLJ42289	0.5	0.00432	2.36443	0.530971	0.751643168	-0.221
cg05105330	I	37	16	85063708	KIAA0513	0.58	0.00041	3.39184	0.57633693	0.796974712	-0.221
cg00835825	I	37	16	85608058		0.58	0.00041	3.39184	0.71313207	0.934173548	-0.221
cg01565508	II	37	17	8869961	PIK3R5	0.62	0.0001	3.98291	0.44120365	0.662105727	-0.221
cg27576485	I	37	17	40558063	PTRF	0.62	0.0001	3.98291	0.56798231	0.789274278	-0.221
cg17628249	II	37	17	58499854	C17orf64	0.46	0.01197	1.92207	0.53218885	0.753003177	-0.221
cg00274965	I	37	21	34405681		0.5	0.00432	2.36443	0.422216	0.20089623	0.221
cg25139649	II	37	1	2165579	SKI	0.5	0.00432	2.36443	0.47295673	0.692518246	-0.22
cg00619207	II	37	1	111743368	DENND2D	0.42	0.02991	1.52413	0.39892808	0.618504448	-0.22
cg07356342	II	37	1	161183820	NDUFS2;NDUFS2;F(	0.54	0.0014	2.85345	0.63178843	0.851803174	-0.22
cg27293155	II	37	2	38044012		0.54	0.0014	2.85345	0.66717732	0.887519789	-0.22
cg12177944	I	37	3	129322537	PLXND1	0.46	0.01197	1.92207	0.59285981	0.372504285	0.22
cg27203560	II	37	3	193629645		0.54	0.0014	2.85345	0.48651591	0.706083007	-0.22
cg25213720	II	37	5	176734343	MXD3;MXD3	0.5	0.00432	2.36443	0.29023797	0.510033268	-0.22
cg19295314	II	37	6	1635640	GMDS	0.54	0.0014	2.85345	0.57219489	0.791915118	-0.22
cg03097134	II	37	6	37598264		0.62	0.0001	3.98291	0.57717873	0.797398001	-0.22

cg01980222	II	37	6	41130917	TREM2;TREM2	0.5	0.00432	2.36443	0.41629312	0.636393695	-0.22
cg17404534	II	37	7	6461991	DAGLB;DAGLB	0.62	0.0001	3.98291	0.58244595	0.802555294	-0.22
cg26673980	II	37	7	151493820	PRKAG2;PRKAG2	0.67	2.34E-05	4.63072	0.54201215	0.761781191	-0.22
cg14927855	I	37	7	155151427		0.5	0.00432	2.36443	0.65248318	0.87212488	-0.22
cg24414325	II	37	12	56414442	IKZF4	0.67	2.34E-05	4.63072	0.44738161	0.667355588	-0.22
cg09789874	II	37	12	125262045		0.58	0.00041	3.39184	0.56507853	0.785431862	-0.22
cg27510257	I	37	13	111305863	CARS2	0.62	0.0001	3.98291	0.69925086	0.479381266	0.22
cg01332683	II	37	16	16190393	ABCC1;ABCC1;ABC(	0.67	2.34E-05	4.63072	0.40504503	0.625504576	-0.22
cg08708790	II	37	16	22176873		0.54	0.0014	2.85345	0.47759865	0.25716682	0.22
cg08351131	II	37	17	43242591	HEXIM2	0.62	0.0001	3.98291	0.406353	0.626031225	-0.22
cg23760945	II	37	19	11665140	ELOF1	0.54	0.0014	2.85345	0.56599746	0.345806731	0.22
cg05234415	II	37	19	38743744	PPP1R14A	0.46	0.01197	1.92207	0.6168445	0.396741942	0.22
cg21581845	I	37	1	15127680	KIAA1026;KIAA102(	0.62	0.0001	3.98291	0.66012781	0.878916485	-0.219
cg19268695	II	37	1	111743411	DENND2D	0.46	0.01197	1.92207	0.43045141	0.649412034	-0.219
cg27304328	II	37	1	160519425	CD84	0.46	0.01197	1.92207	0.54280087	0.324106861	0.219
cg13464573	II	37	2	7172097	RNF144A	0.54	0.0014	2.85345	0.54973968	0.768593159	-0.219
cg14965639	II	37	2	48795994	STON1-GTF2A1L	0.46	0.01197	1.92207	0.51868377	0.737776395	-0.219
cg24371033	II	37	2	241807924	AGXT	0.58	0.00041	3.39184	0.41353174	0.632843859	-0.219
cg15275312	II	37	3	50363059	TUSC2	0.58	0.00041	3.39184	0.34537245	0.564688206	-0.219
cg27383859	II	37	3	122830175	PDIA5;PDIA5	0.5	0.00432	2.36443	0.60828948	0.389512406	0.219
cg00774102	II	37	6	31590870	BAT2;SNORA38	0.54	0.0014	2.85345	0.51113494	0.730203399	-0.219
cg23254569	II	37	6	35451487	TEAD3	0.62	0.0001	3.98291	0.54602194	0.765518285	-0.219
cg14933494	I	37	7	2661902		0.54	0.0014	2.85345	0.40901093	0.62810836	-0.219
cg15200418	II	37	7	4755010	FOXK1	0.46	0.01197	1.92207	0.57627128	0.795365635	-0.219
cg15030712	II	37	7	29304984	CHN2	0.54	0.0014	2.85345	0.40128865	0.620346842	-0.219
cg14089503	II	37	8	37755555	RAB11FIP1;RAB11F	0.5	0.00432	2.36443	0.61338025	0.394657046	0.219
cg01888395	II	37	9	132145105		0.62	0.0001	3.98291	0.60136407	0.820800584	-0.219
cg08726522	II	37	11	8739587	ST5;ST5;ST5	0.54	0.0014	2.85345	0.35561259	0.575048988	-0.219
cg02049955	II	37	11	118045910	SCN2B	0.58	0.00041	3.39184	0.49344005	0.712913423	-0.219
cg07658702	I	37	12	110182938	MGC14436;MGC14	0.5	0.00432	2.36443	0.24006233	0.458734926	-0.219
cg22301418	I	37	13	113514151	ATP11A;ATP11A	0.67	2.34E-05	4.63072	0.70838738	0.489380815	0.219
cg13738075	II	37	13	114793125	RASA3	0.46	0.01197	1.92207	0.57608086	0.356637295	0.219
cg09985344	II	37	16	84648441	COTL1	0.54	0.0014	2.85345	0.76426783	0.545523938	0.219

cg04444104	I	37	16	89059436		0.62	0.0001	3.98291	0.50152951	0.282298389	0.219
cg14377681	II	37	17	37875709	ERBB2;ERBB2	0.5	0.00432	2.36443	0.5351359	0.754506823	-0.219
cg09380069	II	37	17	53847116	PCTP;PCTP	0.54	0.0014	2.85345	0.4482057	0.667597309	-0.219
cg03823431	II	37	17	79229385	SLC38A10;SLC38A1	0.62	0.0001	3.98291	0.38532938	0.604538778	-0.219
cg14761989	II	37	17	79544417	NPLOC4	0.58	0.00041	3.39184	0.39752423	0.616302525	-0.219
cg23357708	II	37	19	8580509	ZNF414;ZNF414	0.42	0.02991	1.52413	0.39351264	0.612158683	-0.219
cg07835236	II	37	20	62549301	MIR941-1;DNAJC5	0.46	0.01197	1.92207	0.6862754	0.466866936	0.219
cg17923377	II	37	22	50174057	BRD1;LOC90834	0.62	0.0001	3.98291	0.47813003	0.697479153	-0.219
cg26406563	II	37	1	55117536	C1orf175;C1orf175	0.54	0.0014	2.85345	0.42611885	0.644205305	-0.218
cg25213452	II	37	1	206630385	SRGAP2;SRGAP2	0.58	0.00041	3.39184	0.57616385	0.794628899	-0.218
cg12338137	II	37	2	218701429	TNS1	0.62	0.0001	3.98291	0.5008352	0.719005175	-0.218
cg20382154	II	37	2	239463716		0.46	0.01197	1.92207	0.31735583	0.535294658	-0.218
cg17500228	I	37	5	448790	EXOC3	0.62	0.0001	3.98291	0.68340194	0.90095038	-0.218
cg05876069	II	37	5	150020288	SYNPO;SYNPO;SYNI	0.54	0.0014	2.85345	0.41620908	0.634407183	-0.218
cg00970279	I	37	6	31797416	HSPA1B	0.5	0.00432	2.36443	0.34279973	0.124384529	0.218
cg01374870	II	37	6	32905127	HLA-DMB	0.54	0.0014	2.85345	0.36044205	0.57851774	-0.218
cg23464284	II	37	6	166996837	RPS6KA2;RPS6KA2	0.62	0.0001	3.98291	0.65430364	0.871969381	-0.218
cg23184226	II	37	7	97880563	TECPR1	0.62	0.0001	3.98291	0.61748187	0.399481582	0.218
cg23033749	II	37	7	116610238	ST7;ST7	0.62	0.0001	3.98291	0.57781446	0.795493772	-0.218
cg10692363	I	37	10	80327890		0.54	0.0014	2.85345	0.69355429	0.912054257	-0.218
cg15228694	II	37	11	7692131	CYB5R2	0.62	0.0001	3.98291	0.63475882	0.852701577	-0.218
cg20645966	II	37	12	117131719		0.5	0.00432	2.36443	0.53058578	0.312451865	0.218
cg00872354	II	37	14	54080601		0.5	0.00432	2.36443	0.47497772	0.256543141	0.218
cg00736299	I	37	16	4730465	MGRN1;MGRN1;M(	0.58	0.00041	3.39184	0.56725338	0.785025248	-0.218
cg04202511	I	37	16	68117991	NFATC3;NFATC3;NI	0.5	0.00432	2.36443	0.62762631	0.845920107	-0.218
cg08289350	II	37	17	74524371	CYGB	0.46	0.01197	1.92207	0.4308303	0.212632782	0.218
cg15816012	I	37	19	54726870	LILRB3;LILRB3;LILRE	0.46	0.01197	1.92207	0.51609938	0.733723197	-0.218
cg06650246	I	37	1	206897270	MAPKAPK2;MAPKA	0.58	0.00041	3.39184	0.39036062	0.607839628	-0.217
cg16967583	II	37	2	241807859	AGXT	0.5	0.00432	2.36443	0.41575262	0.633147102	-0.217
cg16945415	II	37	3	125980095		0.54	0.0014	2.85345	0.32374215	0.540678303	-0.217
cg12670963	II	37	5	1511422	LPCAT1	0.46	0.01197	1.92207	0.80098739	0.584473839	0.217
cg01204911	II	37	5	142187317	ARHGAP26;ARHGAI	0.46	0.01197	1.92207	0.46133182	0.677950103	-0.217
cg06625767	II	37	5	176836695	F12	0.54	0.0014	2.85345	0.59975105	0.816476326	-0.217

cg02087075	II	37	6	44226395	SLC35B2;NFKBIE	0.42	0.02991	1.52413	0.54955282	0.332511711	0.217
cg12897164	II	37	8	124529273	FBXO32;FBXO32	0.5	0.00432	2.36443	0.3939831	0.176878947	0.217
cg24103651	II	37	10	113987401		0.5	0.00432	2.36443	0.3711759	0.587844456	-0.217
cg14183540	II	37	11	3175007	OSBPL5;OSBPL5;OS	0.58	0.00041	3.39184	0.43904359	0.655937741	-0.217
cg27652459	II	37	11	46709238	ARHGAP1	0.67	2.34E-05	4.63072	0.56372375	0.781157105	-0.217
cg07188863	II	37	11	110055425		0.62	0.0001	3.98291	0.46376833	0.681132637	-0.217
cg14102807	II	37	16	28943677	CD19	0.42	0.02991	1.52413	0.54171206	0.324811576	0.217
cg07396047	II	37	16	87735077	LOC100129637	0.42	0.02991	1.52413	0.50049322	0.283206048	0.217
cg04940329	II	37	17	39093054	KRT23	0.46	0.01197	1.92207	0.50642139	0.723920939	-0.217
cg20925233	II	37	17	47661136	NXPH3	0.58	0.00041	3.39184	0.57716794	0.79457657	-0.217
cg20086579	I	37	19	11665102	ELOF1	0.5	0.00432	2.36443	0.64513748	0.428326935	0.217
cg06779113	II	37	1	228273674	ARF1;ARF1;ARF1;AI	0.54	0.0014	2.85345	0.62675545	0.842590801	-0.216
cg24827600	II	37	2	169355957	LASS6	0.54	0.0014	2.85345	0.6392315	0.855535914	-0.216
cg06453088	II	37	4	5887725	CRMP1;CRMP1	0.5	0.00432	2.36443	0.54531175	0.328928747	0.216
cg11021317	II	37	4	11471730		0.5	0.00432	2.36443	0.75748221	0.541217902	0.216
cg22902505	II	37	4	81119473	PRDM8;PRDM8	0.58	0.00041	3.39184	0.43871454	0.654851481	-0.216
cg00372886	II	37	6	30126176	TRIM10;TRIM10	0.54	0.0014	2.85345	0.58350569	0.799764325	-0.216
cg12926693	II	37	6	36665611		0.58	0.00041	3.39184	0.38135016	0.597763312	-0.216
cg09373037	I	37	10	46970625	SYT15;SYT15	0.5	0.00432	2.36443	0.10192109	0.318294367	-0.216
cg19126910	II	37	11	111249659	POU2AF1	0.58	0.00041	3.39184	0.52531028	0.309704044	0.216
cg19186056	II	37	11	113903985		0.54	0.0014	2.85345	0.31353671	0.529746025	-0.216
cg04930596	II	37	12	124864528	NCOR2;NCOR2	0.62	0.0001	3.98291	0.46377804	0.679386772	-0.216
cg12423658	II	37	13	111318555	CARS2	0.58	0.00041	3.39184	0.54513957	0.760668836	-0.216
cg02315513	II	37	14	22974951		0.58	0.00041	3.39184	0.60225592	0.38651713	0.216
cg07635227	I	37	16	4714815	MGRN1;MGRN1;M	0.42	0.02991	1.52413	0.28831161	0.503825508	-0.216
cg26985681	II	37	16	15135397	NTAN1	0.71	4.57E-06	5.34042	0.3779314	0.594090228	-0.216
cg10020520	II	37	16	30976186	SETD1A	0.62	0.0001	3.98291	0.55953712	0.775433048	-0.216
cg08782002	II	37	17	61847885	CCDC47	0.54	0.0014	2.85345	0.63888561	0.854568244	-0.216
cg04034685	II	37	17	62085532	ICAM2;ICAM2;ICAN	0.67	2.34E-05	4.63072	0.5793767	0.795753209	-0.216
cg17846016	II	37	17	80181015		0.58	0.00041	3.39184	0.63666081	0.853009177	-0.216
cg09455342	II	37	17	80346849		0.58	0.00041	3.39184	0.59407209	0.810190534	-0.216
cg13076829	II	37	18	45530698		0.54	0.0014	2.85345	0.59030403	0.374194632	0.216
cg00965578	II	37	22	36560801	APOL3;APOL3;APOI	0.46	0.01197	1.92207	0.30129149	0.517240539	-0.216

cg20633875	II	37	1	32572611	KPNA6	0.5	0.00432	2.36443	0.45967676	0.674955018	-0.215
cg02736746	II	37	1	208263446	PLXNA2	0.71	4.57E-06	5.34042	0.55461871	0.769908557	-0.215
cg05644602	II	37	2	74795186	C2orf65	0.67	2.34E-05	4.63072	0.66126896	0.876765941	-0.215
cg06277638	II	37	3	195424838	MIR570	0.67	2.34E-05	4.63072	0.47419857	0.6893443	-0.215
cg05065948	II	37	4	26275089		0.54	0.0014	2.85345	0.60685127	0.82141946	-0.215
cg26913344	II	37	4	185872829		0.58	0.00041	3.39184	0.40501752	0.619662556	-0.215
cg00332146	II	37	5	175975901	PCDH24	0.46	0.01197	1.92207	0.28407353	0.499283731	-0.215
cg04569651	II	37	5	177956199	COL23A1	0.58	0.00041	3.39184	0.44241447	0.657399433	-0.215
cg11381564	I	37	6	32808619	PSMB8;PSMB8	0.67	2.34E-05	4.63072	0.29921663	0.084332309	0.215
cg17291166	II	37	6	37051189		0.54	0.0014	2.85345	0.54185605	0.757193597	-0.215
cg24045212	II	37	7	2020131	MAD1L1;MAD1L1;N	0.46	0.01197	1.92207	0.5809721	0.366312368	0.215
cg17291423	II	37	7	158263204	PTPRN2;PTPRN2;PT	0.62	0.0001	3.98291	0.4448443	0.660077176	-0.215
cg04007350	II	37	7	158263221	PTPRN2;PTPRN2;PT	0.67	2.34E-05	4.63072	0.6143035	0.829033256	-0.215
cg07472373	II	37	10	50595181	DRGX	0.58	0.00041	3.39184	0.67722392	0.891962918	-0.215
cg09010707	II	37	12	26392567		0.5	0.00432	2.36443	0.46075388	0.675333309	-0.215
cg25808809	II	37	13	114917199		0.5	0.00432	2.36443	0.49075758	0.705782437	-0.215
cg26889953	I	37	15	22915992	CYFIP1	0.54	0.0014	2.85345	0.50805056	0.722975965	-0.215
cg02585724	II	37	15	81116461	KIAA1199	0.46	0.01197	1.92207	0.55179821	0.336784543	0.215
cg13856674	II	37	15	93722590		0.54	0.0014	2.85345	0.5120732	0.727434358	-0.215
cg23815491	II	37	16	72088622	HP;HP	0.58	0.00041	3.39184	0.58673725	0.80181728	-0.215
cg09395612	II	37	16	86018936		0.5	0.00432	2.36443	0.44026615	0.655410374	-0.215
cg04320476	II	37	17	1492206	SLC43A2	0.62	0.0001	3.98291	0.5953078	0.810602483	-0.215
cg18369972	II	37	17	9940121	GAS7;GAS7	0.58	0.00041	3.39184	0.4859616	0.701448762	-0.215
cg23948703	II	37	17	42928238	EFTUD2;EFTUD2	0.46	0.01197	1.92207	0.63392988	0.419286553	0.215
cg18038894	II	37	17	48506385	ACSF2	0.54	0.0014	2.85345	0.6494951	0.864263289	-0.215
cg19879906	I	37	19	16392219		0.5	0.00432	2.36443	0.58380122	0.798554241	-0.215
cg04993279	II	37	1	8940460		0.54	0.0014	2.85345	0.37008953	0.584529557	-0.214
cg15122621	II	37	1	156341523	RHBG;RHBG	0.5	0.00432	2.36443	0.35126553	0.565763256	-0.214
cg04891053	II	37	1	161053558	PVRL4	0.58	0.00041	3.39184	0.42648325	0.64004234	-0.214
cg04316353	II	37	6	38123312		0.46	0.01197	1.92207	0.36404571	0.577713237	-0.214
cg14192130	II	37	6	167535764	CCR6;CCR6	0.54	0.0014	2.85345	0.4910428	0.277487178	0.214
cg09539720	II	37	7	150885458	ASB10;ASB10;ASB1	0.58	0.00041	3.39184	0.3495145	0.135381251	0.214
cg20657903	II	37	10	50387348	C10orf128	0.42	0.02991	1.52413	0.55029046	0.335873953	0.214

cg27093403	II	37	12	25083077	BCAT1	0.62	0.0001	3.98291	0.5810683	0.795121573	-0.214
cg08189198	II	37	14	51290136	NIN;NIN;NIN;NIN;N	0.5	0.00432	2.36443	0.74665519	0.532896274	0.214
cg01626885	II	37	15	45937757	SQRDL	0.58	0.00041	3.39184	0.38540673	0.599892372	-0.214
cg03901454	II	37	16	87588683		0.58	0.00041	3.39184	0.45701067	0.670688787	-0.214
cg00227665	II	37	16	87735443	LOC100129637	0.5	0.00432	2.36443	0.64391251	0.430138853	0.214
cg00858400	II	37	16	87904580	SLC7A5	0.54	0.0014	2.85345	0.6111407	0.825061291	-0.214
cg25112590	I	37	17	3870415		0.54	0.0014	2.85345	0.54854183	0.334225643	0.214
cg05784862	II	37	17	25799018	KSR1	0.46	0.01197	1.92207	0.67773973	0.463871066	0.214
cg25070639	II	37	17	41170693	VAT1	0.5	0.00432	2.36443	0.4053659	0.619194164	-0.214
cg12212103	II	37	17	48231729		0.54	0.0014	2.85345	0.47681746	0.690745418	-0.214
cg14619064	II	37	17	56355331	MPO	0.58	0.00041	3.39184	0.62208188	0.836413144	-0.214
cg16131053	II	37	18	45459276		0.62	0.0001	3.98291	0.38665458	0.600765037	-0.214
cg15200801	II	37	19	48999042	LMTK3	0.58	0.00041	3.39184	0.59079539	0.804466899	-0.214
cg07377519	II	37	19	50269083	AP2A1;AP2A1	0.58	0.00041	3.39184	0.32653988	0.540541905	-0.214
cg15033013	II	37	1	2082458	PRKCZ;PRKCZ;PRKC	0.54	0.0014	2.85345	0.40363864	0.61621949	-0.213
cg20489909	II	37	2	242711046		0.71	4.57E-06	5.34042	0.37252657	0.585480048	-0.213
cg27516100	II	37	6	30624520	DHX16;DHX16	0.58	0.00041	3.39184	0.5188445	0.73231219	-0.213
cg14438453	II	37	6	39786566	DAAM2	0.58	0.00041	3.39184	0.52151914	0.734180461	-0.213
cg04932082	II	37	6	91113506		0.58	0.00041	3.39184	0.53979814	0.752748492	-0.213
cg19627093	I	37	8	1900892	ARHGEF10	0.46	0.01197	1.92207	0.63340259	0.420488735	0.213
cg10076006	II	37	8	6735559	DEFB1	0.58	0.00041	3.39184	0.3714279	0.584573636	-0.213
cg24917065	II	37	8	23418389	SLC25A37	0.54	0.0014	2.85345	0.61758684	0.831001036	-0.213
cg07769015	II	37	8	142238770	SLC45A4	0.54	0.0014	2.85345	0.65932415	0.87276427	-0.213
cg25252561	II	37	10	75610927	CAMK2G;CAMK2G;	0.54	0.0014	2.85345	0.29889492	0.512313139	-0.213
cg04350202	II	37	10	111653363	XPNPEP1;XPNPEP1;	0.54	0.0014	2.85345	0.63055403	0.843286509	-0.213
cg18169610	II	37	11	2415617	CD81	0.46	0.01197	1.92207	0.54929955	0.336465056	0.213
cg24102222	I	37	12	129280449	SLC15A4	0.54	0.0014	2.85345	0.40585318	0.193027329	0.213
cg05349024	II	37	14	101471543		0.54	0.0014	2.85345	0.31420544	0.526851804	-0.213
cg18176723	II	37	15	75336436	PPCDC	0.5	0.00432	2.36443	0.47134396	0.684659107	-0.213
cg02889001	II	37	16	1519785	CLCN7;CLCN7	0.54	0.0014	2.85345	0.40814185	0.620650296	-0.213
cg16598508	II	37	16	88228364		0.58	0.00041	3.39184	0.45825159	0.671034836	-0.213
cg08413427	II	37	17	42828304	DBF4B	0.58	0.00041	3.39184	0.58834254	0.801082976	-0.213
cg16555866	I	37	17	80830922	TBCD	0.58	0.00041	3.39184	0.63813543	0.425194	0.213

cg07979271	II	37	19	50921353	SPIB	0.5	0.00432	2.36443	0.61901668	0.406289999	0.213
cg03894068	II	37	20	19955537	RIN2	0.67	2.34E-05	4.63072	0.46005942	0.673517261	-0.213
cg25767870	II	37	1	118188756		0.58	0.00041	3.39184	0.6225419	0.834333451	-0.212
cg05350800	I	37	1	178550844		0.46	0.01197	1.92207	0.45539446	0.243676162	0.212
cg25570328	II	37	2	108903952	SULT1C2;SULT1C2	0.54	0.0014	2.85345	0.62759185	0.839591131	-0.212
cg03052004	II	37	6	11702389		0.54	0.0014	2.85345	0.54098486	0.753033147	-0.212
cg13185046	II	37	6	32165321	NOTCH4	0.46	0.01197	1.92207	0.33246839	0.54453165	-0.212
cg23185774	II	37	6	36972163	FGD2	0.46	0.01197	1.92207	0.60121222	0.389344573	0.212
cg08044454	II	37	7	37024552	ELMO1;ELMO1;ELM	0.62	0.0001	3.98291	0.42997417	0.64238	-0.212
cg06399735	II	37	8	18836280	PSD3	0.5	0.00432	2.36443	0.34456334	0.556824218	-0.212
cg01060444	II	37	10	88100354	GRID1	0.42	0.02991	1.52413	0.52627993	0.314515338	0.212
cg15342452	II	37	10	127823065	ADAM12;ADAM12	0.5	0.00432	2.36443	0.51740733	0.7295624	-0.212
cg04948649	I	37	11	1901748	LSP1;LSP1;LSP1;LSP	0.58	0.00041	3.39184	0.5662944	0.778105111	-0.212
cg22212560	II	37	11	65175482	FRMD8	0.42	0.02991	1.52413	0.55761627	0.345437741	0.212
cg26620147	II	37	12	58210716	AVIL	0.54	0.0014	2.85345	0.42277577	0.634280806	-0.212
cg03515464	II	37	14	101015513	BEGAIN;BEGAIN	0.46	0.01197	1.92207	0.64795492	0.4355544	0.212
cg10243855	II	37	17	1096705		0.5	0.00432	2.36443	0.50727717	0.719023518	-0.212
cg11122944	II	37	17	9924800	GAS7;GAS7;GAS7	0.54	0.0014	2.85345	0.34364283	0.555322254	-0.212
cg16896911	II	37	19	33895690	PEPD;PEPD;PEPD	0.54	0.0014	2.85345	0.38780522	0.599939817	-0.212
cg26160564	II	37	1	3826643	LOC100133612	0.5	0.00432	2.36443	0.45188378	0.241191477	0.211
cg15030789	II	37	1	27438378	SLC9A1	0.46	0.01197	1.92207	0.41396023	0.625335526	-0.211
cg11939496	II	37	1	160833560	CD244;CD244;CD24	0.71	4.57E-06	5.34042	0.51793284	0.729219157	-0.211
cg07957995	II	37	1	224644750		0.54	0.0014	2.85345	0.41954019	0.630735909	-0.211
cg07833951	II	37	1	234891473		0.54	0.0014	2.85345	0.42136327	0.632531714	-0.211
cg07729527	II	37	2	174889845		0.46	0.01197	1.92207	0.42360229	0.63457742	-0.211
cg07038243	II	37	5	149868855		0.54	0.0014	2.85345	0.34016074	0.550803369	-0.211
cg19988490	II	37	6	30167065	TRIM26	0.54	0.0014	2.85345	0.33077562	0.541980897	-0.211
cg11748260	II	37	6	30624395	DHX16;DHX16	0.62	0.0001	3.98291	0.41761149	0.628322287	-0.211
cg14078070	II	37	6	167051518	RPS6KA2	0.5	0.00432	2.36443	0.53624107	0.3257272	0.211
cg23386895	II	37	11	65083974	CDC42EP2	0.62	0.0001	3.98291	0.59831188	0.809704132	-0.211
cg02929855	II	37	12	1922067	CACNA2D4	0.5	0.00432	2.36443	0.50438705	0.715029724	-0.211
cg04103645	II	37	13	51898948		0.42	0.02991	1.52413	0.54504983	0.33450643	0.211
cg27340723	I	37	16	4061608	ADCY9	0.67	2.34E-05	4.63072	0.69047457	0.901163584	-0.211

cg04264560	II	37	16	27125068		0.5	0.00432	2.36443	0.47026377	0.259733138	0.211
cg12111500	II	37	21	44863962		0.46	0.01197	1.92207	0.55508051	0.344504393	0.211
cg09208331	II	37	21	44864004		0.5	0.00432	2.36443	0.52157672	0.310244277	0.211
cg14220262	II	37	1	1114963	TTLL10;TTLL10	0.5	0.00432	2.36443	0.48843378	0.278098559	0.21
cg22677556	II	37	1	12238390	TNFRSF1B	0.5	0.00432	2.36443	0.45712235	0.666853576	-0.21
cg23691781	II	37	1	28212827	C1orf38;C1orf38;C1	0.46	0.01197	1.92207	0.45578553	0.66551314	-0.21
cg24681307	II	37	1	110526191	AHCYL1	0.62	0.0001	3.98291	0.50547553	0.715319063	-0.21
cg21195376	II	37	1	204449827	PIK3C2B	0.5	0.00432	2.36443	0.41813616	0.208622735	0.21
cg13429095	II	37	1	206913187		0.54	0.0014	2.85345	0.3808004	0.170592828	0.21
cg21241410	II	37	2	43267816		0.5	0.00432	2.36443	0.36888077	0.578877603	-0.21
cg09308580	II	37	2	43405947		0.67	2.34E-05	4.63072	0.41310364	0.623237635	-0.21
cg05242065	II	37	2	85060011	C2orf89	0.54	0.0014	2.85345	0.62625872	0.835880068	-0.21
cg11051295	II	37	4	185876283		0.5	0.00432	2.36443	0.68898053	0.478879912	0.21
cg16478536	I	37	6	408730	IRF4	0.46	0.01197	1.92207	0.23420939	0.444666624	-0.21
cg23280720	II	37	6	139483193	HECA	0.75	7.61E-07	6.11857	0.48185426	0.271783491	0.21
cg18332229	II	37	7	971130	ADAP1	0.58	0.00041	3.39184	0.51747224	0.727227738	-0.21
cg16735495	II	37	7	105690812		0.58	0.00041	3.39184	0.54317771	0.7535085	-0.21
cg21764708	II	37	7	151493629	PRKAG2;PRKAG2	0.62	0.0001	3.98291	0.58951834	0.799602113	-0.21
cg17117981	I	37	8	144410972	TOP1MT	0.42	0.02991	1.52413	0.50619506	0.716245416	-0.21
cg25587233	II	37	9	131872005	CRAT;CRAT;PPP2R4	0.58	0.00041	3.39184	0.37130337	0.581754248	-0.21
cg05934015	I	37	12	129280265	SLC15A4	0.46	0.01197	1.92207	0.80912363	0.598685129	0.21
cg23237276	II	37	14	101827449		0.46	0.01197	1.92207	0.41717449	0.207031936	0.21
cg08842616	II	37	16	70733772	VAC14	0.58	0.00041	3.39184	0.57448731	0.784896207	-0.21
cg00471368	II	37	16	81817629	PLCG2	0.54	0.0014	2.85345	0.74467946	0.53474766	0.21
cg00473501	II	37	17	56354594	MPO	0.54	0.0014	2.85345	0.54744218	0.757222998	-0.21
cg13533061	II	37	17	74712429	JMJD6	0.42	0.02991	1.52413	0.44084078	0.650650677	-0.21
cg03330678	II	37	17	75316233	SEPT9;SEPT9;SEPT9	0.5	0.00432	2.36443	0.36319868	0.5728322	-0.21
cg01791634	II	37	17	76130139	TMC8	0.54	0.0014	2.85345	0.39829034	0.187795661	0.21
cg20317872	II	37	1	111743202	DENND2D;DENND2	0.54	0.0014	2.85345	0.48614436	0.695433483	-0.209
cg01741999	I	37	2	219137824	PNKD	0.5	0.00432	2.36443	0.53708914	0.328473399	0.209
cg26395694	II	37	3	4783306	ITPR1;ITPR1;ITPR1	0.67	2.34E-05	4.63072	0.23271173	0.442128295	-0.209
cg02494703	II	37	11	64659387	MIR194-2;MIR192	0.5	0.00432	2.36443	0.51073563	0.301870633	0.209
cg16449084	II	37	11	67069814	SSH3;ANKRD13D;AI	0.58	0.00041	3.39184	0.42538865	0.634806728	-0.209

cg20184271	II	37	12	14413090		0.54	0.0014	2.85345	0.42672079	0.636172481	-0.209
cg05169499	II	37	12	52673946		0.62	0.0001	3.98291	0.4551569	0.663949064	-0.209
cg17230002	II	37	12	54689504	NFE2;NFE2;NFE2	0.54	0.0014	2.85345	0.61464966	0.823250049	-0.209
cg04853218	II	37	14	55769688	FBXO34;FBXO34	0.62	0.0001	3.98291	0.53477808	0.744068151	-0.209
cg08368934	II	37	16	57701455	GPR97	0.62	0.0001	3.98291	0.50408773	0.71309397	-0.209
cg07512993	II	37	16	66582592	TK2	0.67	2.34E-05	4.63072	0.53504899	0.744530828	-0.209
cg05246522	II	37	17	25798973	KSR1	0.54	0.0014	2.85345	0.70250964	0.493152945	0.209
cg12131208	I	37	17	58499700	C17orf64	0.54	0.0014	2.85345	0.65709865	0.866496083	-0.209
cg27262850	II	37	18	74826769	MBP;MBP	0.67	2.34E-05	4.63072	0.32099359	0.529861916	-0.209
cg22023664	II	37	19	2619608	GNG7	0.54	0.0014	2.85345	0.53022073	0.738998165	-0.209
cg24414363	II	37	22	42336273	CENPM;CENPM;CEI	0.54	0.0014	2.85345	0.41045912	0.201592776	0.209
cg20061654	I	37	22	45608492	C22orf9;C22orf9	0.58	0.00041	3.39184	0.73180626	0.941292556	-0.209
cg12279968	I	37	1	3826425	LOC100133612	0.54	0.0014	2.85345	0.78104683	0.573177685	0.208
cg03945538	II	37	1	173447427	PRDX6	0.75	7.61E-07	6.11857	0.27029148	0.478035953	-0.208
cg13584784	I	37	6	30619190	C6orf136;C6orf136	0.54	0.0014	2.85345	0.68577011	0.478150933	0.208
cg00226923	II	37	6	36972027	FGD2	0.5	0.00432	2.36443	0.53481868	0.3266343	0.208
cg17977099	II	37	7	73001879		0.62	0.0001	3.98291	0.56252707	0.770155452	-0.208
cg18929814	I	37	7	97875293	TECPR1	0.54	0.0014	2.85345	0.62009435	0.828397423	-0.208
cg14512326	II	37	9	86240166	C9orf103	0.5	0.00432	2.36443	0.64848898	0.856490164	-0.208
cg18065599	II	37	11	47968995		0.54	0.0014	2.85345	0.36861089	0.576218965	-0.208
cg06341100	II	37	11	68138908	LRP5	0.46	0.01197	1.92207	0.70953655	0.50198484	0.208
cg24520862	II	37	11	75943228		0.54	0.0014	2.85345	0.43463298	0.226255597	0.208
cg20533957	II	37	11	94278538	FUT4	0.54	0.0014	2.85345	0.45480259	0.663185688	-0.208
cg22274234	II	37	12	109888858	KCTD10	0.5	0.00432	2.36443	0.41880748	0.627204389	-0.208
cg27180555	II	37	12	112822378		0.58	0.00041	3.39184	0.5748538	0.782951304	-0.208
cg07152597	II	37	14	106152163		0.54	0.0014	2.85345	0.74054231	0.53270108	0.208
cg04709771	II	37	16	646395	RAB40C	0.54	0.0014	2.85345	0.39408247	0.602549698	-0.208
cg08428292	I	37	16	85981373		0.71	4.57E-06	5.34042	0.43061782	0.222182557	0.208
cg13221924	II	37	17	6495080	KIAA0753	0.54	0.0014	2.85345	0.64214975	0.850468781	-0.208
cg16030878	II	37	17	77914307	TBC1D16	0.62	0.0001	3.98291	0.55390097	0.761834344	-0.208
cg24259291	II	37	20	47874072	ZNFX1	0.62	0.0001	3.98291	0.52262036	0.730910036	-0.208
cg15125868	II	37	21	35746798	FAM165B	0.58	0.00041	3.39184	0.41333997	0.621009244	-0.208
cg14043316	II	37	22	50743420	PLXNB2	0.54	0.0014	2.85345	0.42184291	0.630068941	-0.208

cg16686279	II	37	1	4087226		0.42	0.02991	1.52413	0.62924075	0.421787882	0.207
cg09366519	II	37	1	209877970	HSD11B1;HSD11B1	0.62	0.0001	3.98291	0.65708282	0.864211868	-0.207
cg13359689	II	37	2	55636555	CCDC88A;CCDC88A	0.46	0.01197	1.92207	0.34417021	0.551392126	-0.207
cg11464160	II	37	2	131870398	PLEKHB2;PLEKHB2	0.58	0.00041	3.39184	0.50259514	0.709440488	-0.207
cg25138484	I	37	2	233242758	ALPP	0.5	0.00432	2.36443	0.54113561	0.334536858	0.207
cg00812557	II	37	4	38073835	TBC1D1	0.67	2.34E-05	4.63072	0.30087634	0.508374914	-0.207
cg05524458	II	37	5	10566782	ANKRD33B	0.42	0.02991	1.52413	0.57853941	0.371314178	0.207
cg17781669	II	37	5	149868359		0.67	2.34E-05	4.63072	0.36189237	0.569242835	-0.207
cg10578938	II	37	5	156695410	CYFIP2;CYFIP2;CYFI	0.58	0.00041	3.39184	0.40682428	0.199898645	0.207
cg26683398	II	37	5	179220589	LTC4S	0.62	0.0001	3.98291	0.43072054	0.638125303	-0.207
cg09698826	II	37	6	12114403	HIVEP1	0.58	0.00041	3.39184	0.80931748	0.601893435	0.207
cg20241876	II	37	6	32180045	NOTCH4	0.5	0.00432	2.36443	0.60340382	0.81076118	-0.207
cg00904258	II	37	7	968194	ADAP1	0.54	0.0014	2.85345	0.49373183	0.700298944	-0.207
cg16569650	II	37	7	2773072	GNA12	0.62	0.0001	3.98291	0.46175188	0.668684333	-0.207
cg09060789	II	37	11	1884988	LSP1	0.54	0.0014	2.85345	0.40557524	0.612106857	-0.207
cg11468085	I	37	11	67435577	ALDH3B2;ALDH3B2	0.5	0.00432	2.36443	0.35749923	0.150412835	0.207
cg10687644	II	37	11	72527565	ATG16L2	0.67	2.34E-05	4.63072	0.41972692	0.627128546	-0.207
cg04625873	II	37	11	118754530	CXCR5	0.46	0.01197	1.92207	0.5640459	0.356782096	0.207
cg25700432	II	37	13	20797446	GJB6;GJB6;GJB6;GJ	0.67	2.34E-05	4.63072	0.57066507	0.777172542	-0.207
cg14184693	II	37	15	44085947	SERF2;MIR1282	0.54	0.0014	2.85345	0.40880886	0.615714466	-0.207
cg04962621	II	37	16	4714733	MGRN1;MGRN1;M(	0.62	0.0001	3.98291	0.43329133	0.639870447	-0.207
cg12799029	II	37	17	984184	ABR;ABR;ABR	0.54	0.0014	2.85345	0.25995356	0.46718443	-0.207
cg25930786	II	37	19	54584636	TARM1	0.58	0.00041	3.39184	0.57461366	0.781366213	-0.207
cg15091747	II	37	21	36262896	RUNX1	0.58	0.00041	3.39184	0.45684259	0.663409298	-0.207
cg21935981	I	37	22	45608465	C22orf9;C22orf9	0.62	0.0001	3.98291	0.73559053	0.942656715	-0.207
cg19595244	II	37	1	110526158	AHCYL1	0.54	0.0014	2.85345	0.60447509	0.810280899	-0.206
cg16139316	II	37	1	153330758	S100A9	0.58	0.00041	3.39184	0.36535657	0.57086729	-0.206
cg11827101	II	37	2	8468422		0.62	0.0001	3.98291	0.49551002	0.701861601	-0.206
cg20730966	II	37	3	33095886	GLB1;GLB1;GLB1	0.46	0.01197	1.92207	0.31623366	0.5220998	-0.206
cg09693588	II	37	3	66633255		0.71	4.57E-06	5.34042	0.56056491	0.354309857	0.206
cg04855678	I	37	3	195946921	OSTalpha	0.5	0.00432	2.36443	0.68016211	0.885984519	-0.206
cg04576021	II	37	6	32784255	HLA-DOB	0.46	0.01197	1.92207	0.63484044	0.429166881	0.206
cg19263161	I	37	7	1144982	C7orf50;C7orf50;C7	0.62	0.0001	3.98291	0.68223134	0.47617244	0.206

cg26565544	II	37	7	101361527		0.62	0.0001	3.98291	0.3699115	0.575466885	-0.206
cg13699650	II	37	9	126102244		0.46	0.01197	1.92207	0.55200216	0.346054457	0.206
cg09233392	I	37	10	50887488	C10orf53;C10orf53	0.62	0.0001	3.98291	0.21198077	0.4180469	-0.206
cg07652788	II	37	10	133598589		0.5	0.00432	2.36443	0.75683018	0.551065582	0.206
cg17635080	II	37	12	109030115		0.5	0.00432	2.36443	0.3805498	0.174167894	0.206
cg20043258	I	37	14	103468582	CDC42BPB	0.58	0.00041	3.39184	0.84507442	0.638897619	0.206
cg09837656	II	37	15	65149245	PLEKHO2	0.5	0.00432	2.36443	0.45629857	0.662033643	-0.206
cg04781080	II	37	15	68924114	CORO2B	0.46	0.01197	1.92207	0.49968151	0.293405209	0.206
cg05575505	II	37	15	101262162		0.46	0.01197	1.92207	0.49300874	0.698758128	-0.206
cg04329347	II	37	16	30063148	ALDOA	0.71	4.57E-06	5.34042	0.52432597	0.730629959	-0.206
cg26954174	II	37	16	50730813	NOD2	0.46	0.01197	1.92207	0.3385226	0.544500752	-0.206
cg07127456	II	37	16	66583081	TK2	0.54	0.0014	2.85345	0.52200804	0.315701783	0.206
cg00582663	II	37	17	7815834	CHD3;CHD3;CHD3	0.62	0.0001	3.98291	0.41461058	0.620562856	-0.206
cg04658021	II	37	17	8056967	PER1	0.5	0.00432	2.36443	0.4196167	0.625168732	-0.206
cg21028142	II	37	17	79581711	NPLOC4	0.54	0.0014	2.85345	0.66909909	0.462707461	0.206
cg06705017	II	37	18	77552402		0.5	0.00432	2.36443	0.55584431	0.761547521	-0.206
cg07073964	II	37	19	698371		0.62	0.0001	3.98291	0.60217883	0.807909725	-0.206
cg23312431	II	37	21	45773997	TRPM2	0.58	0.00041	3.39184	0.36985158	0.576288604	-0.206
cg03125765	II	37	22	30686808	GATSL3	0.58	0.00041	3.39184	0.46656381	0.260813841	0.206
cg05249836	II	37	22	45609402	C22orf9;C22orf9	0.67	2.34E-05	4.63072	0.49908911	0.705197263	-0.206
cg26256901	II	37	1	23918838		0.54	0.0014	2.85345	0.62528883	0.420074415	0.205
cg14708360	II	37	2	129396420		0.5	0.00432	2.36443	0.58686367	0.791536155	-0.205
cg18452449	II	37	3	52260671	TLR9	0.42	0.02991	1.52413	0.51927054	0.314256436	0.205
cg07341007	I	37	3	195489909	MUC4;MUC4;MUC4	0.67	2.34E-05	4.63072	0.55331952	0.75813441	-0.205
cg14647287	II	37	4	6927183	TBC1D14;TBC1D14	0.58	0.00041	3.39184	0.43057519	0.635665788	-0.205
cg26689780	II	37	4	10079554	WDR1;WDR1	0.5	0.00432	2.36443	0.38665804	0.591595851	-0.205
cg25015371	II	37	6	108874950		0.62	0.0001	3.98291	0.5494644	0.754778863	-0.205
cg13821176	II	37	8	126448338	TRIB1	0.62	0.0001	3.98291	0.66455978	0.869401897	-0.205
cg14565465	II	37	9	74298933	TMEM2;TMEM2	0.54	0.0014	2.85345	0.40818136	0.613374219	-0.205
cg07608867	II	37	10	49879854		0.42	0.02991	1.52413	0.52757608	0.32255966	0.205
cg03141090	II	37	10	127262277	LOC100169752	0.5	0.00432	2.36443	0.76511872	0.560310093	0.205
cg16698623	II	37	10	131412968	MGMT	0.42	0.02991	1.52413	0.57515929	0.370177979	0.205
cg07080244	I	37	11	120051676		0.46	0.01197	1.92207	0.34040839	0.545462828	-0.205

cg17281677	II	37	12	6658557	IFFO1;IFFO1;IFFO1	0.58	0.00041	3.39184	0.63118722	0.83609832	-0.205
cg02416333	II	37	13	22523390		0.46	0.01197	1.92207	0.4498113	0.245147936	0.205
cg10877241	II	37	13	52131904		0.58	0.00041	3.39184	0.67307269	0.878155355	-0.205
cg27627493	II	37	15	63767360		0.54	0.0014	2.85345	0.51638345	0.311202619	0.205
cg23035449	II	37	15	90578555	ZNF710	0.58	0.00041	3.39184	0.37401495	0.579103818	-0.205
cg00528616	II	37	16	75036318	ZNRF1	0.62	0.0001	3.98291	0.18780041	0.392759066	-0.205
cg01530032	II	37	17	19435805	SLC47A1	0.54	0.0014	2.85345	0.37859345	0.583097299	-0.205
cg15610437	II	37	19	827821	AZU1	0.5	0.00432	2.36443	0.45945423	0.664782418	-0.205
cg19821297	II	37	19	12890029		0.58	0.00041	3.39184	0.44155778	0.64665747	-0.205
cg15224348	I	37	19	45543538	SFRS16	0.67	2.34E-05	4.63072	0.63537541	0.840481585	-0.205
cg23511285	II	37	19	48205761	GLTSCR1	0.62	0.0001	3.98291	0.54505591	0.750418549	-0.205
cg10416593	II	37	22	50966123	TYMP;SCO2;TYMP;T	0.58	0.00041	3.39184	0.52033643	0.725364584	-0.205
cg26933869	II	37	1	19218007	ALDH4A1;ALDH4A1	0.58	0.00041	3.39184	0.43081289	0.63522091	-0.204
cg04087271	II	37	1	20915334	CDA	0.42	0.02991	1.52413	0.21538691	0.41929694	-0.204
cg27217350	II	37	2	242813634	C2orf85	0.5	0.00432	2.36443	0.5050217	0.301266166	0.204
cg18731398	I	37	3	123414733	MYLK;MYLK;MYLK;I	0.62	0.0001	3.98291	0.52975841	0.73359725	-0.204
cg16571794	II	37	4	7648627	SORCS2	0.54	0.0014	2.85345	0.39576219	0.599336901	-0.204
cg17590710	II	37	5	177740069	COL23A1	0.54	0.0014	2.85345	0.4561482	0.659835678	-0.204
cg03116466	II	37	7	2116512	MAD1L1;MAD1L1;M	0.62	0.0001	3.98291	0.41887045	0.622849206	-0.204
cg19014792	II	37	7	3019159	CARD11	0.5	0.00432	2.36443	0.51964146	0.316056907	0.204
cg03190219	I	37	7	25898688		0.62	0.0001	3.98291	0.24182914	0.445494375	-0.204
cg14722693	II	37	8	19436451	CSGALNACT1;CSGA	0.5	0.00432	2.36443	0.38108537	0.584989761	-0.204
cg11893955	I	37	8	28918821		0.54	0.0014	2.85345	0.7419078	0.537570216	0.204
cg04653308	II	37	9	131872018	CRAT;CRAT;PPP2R4	0.58	0.00041	3.39184	0.29941287	0.503891043	-0.204
cg09455513	II	37	10	3792283		0.67	2.34E-05	4.63072	0.1748934	0.379042149	-0.204
cg08863777	I	37	11	94278457	FUT4	0.58	0.00041	3.39184	0.52201074	0.726121452	-0.204
cg20856545	II	37	13	114917297		0.67	2.34E-05	4.63072	0.59977047	0.803908571	-0.204
cg26118358	II	37	14	23321005		0.5	0.00432	2.36443	0.55566456	0.759903053	-0.204
cg02042712	II	37	16	57702239	GPR97;GPR97	0.58	0.00041	3.39184	0.34536602	0.548904189	-0.204
cg27326306	II	37	16	66442374		0.54	0.0014	2.85345	0.44255789	0.646886601	-0.204
cg04819180	II	37	17	80829157	TBCD	0.46	0.01197	1.92207	0.46545792	0.261408203	0.204
cg19649900	I	37	19	1155030	SBNO2	0.54	0.0014	2.85345	0.51560377	0.719751147	-0.204
cg10357682	II	37	20	3745817	C20orf27	0.62	0.0001	3.98291	0.60971632	0.813957059	-0.204

cg11525020	II	37	20	17674233	BANF2	0.62	0.0001	3.98291	0.80202405	0.598184563	0.204
cg12504284	II	37	22	31601057	RNF185;RNF185;RN	0.71	4.57E-06	5.34042	0.49783125	0.702222846	-0.204
cg07233343	II	37	1	17944660	ARHGEF10L;ARHGE	0.62	0.0001	3.98291	0.60736671	0.810226061	-0.203
cg06958535	II	37	1	203734478	LAX1;LAX1;LAX1	0.62	0.0001	3.98291	0.72742787	0.52402081	0.203
cg07091529	II	37	2	16804409	FAM49A	0.58	0.00041	3.39184	0.52142969	0.724155991	-0.203
cg23924526	II	37	2	45998548	PRKCE	0.5	0.00432	2.36443	0.30562183	0.508744124	-0.203
cg07262620	II	37	2	47982632		0.5	0.00432	2.36443	0.57919539	0.375784404	0.203
cg00810971	II	37	2	65085764		0.58	0.00041	3.39184	0.50126272	0.704473172	-0.203
cg12571570	II	37	2	145267984	ZEB2;ZEB2	0.54	0.0014	2.85345	0.3472886	0.550725675	-0.203
cg14012546	II	37	2	233981788	INPP5D;INPP5D	0.58	0.00041	3.39184	0.52725837	0.730291922	-0.203
cg06360820	II	37	2	242988706		0.5	0.00432	2.36443	0.71137268	0.914207155	-0.203
cg20438472	II	37	3	184293365	EPHB3	0.62	0.0001	3.98291	0.56007232	0.762627904	-0.203
cg24748631	II	37	5	598724		0.67	2.34E-05	4.63072	0.45689351	0.25426599	0.203
cg15253304	I	37	6	209809		0.58	0.00041	3.39184	0.71246884	0.509828385	0.203
cg12629909	II	37	6	32126616	PPT2;PPT2	0.46	0.01197	1.92207	0.67676157	0.474078458	0.203
cg18542546	II	37	7	1982333	MAD1L1;MAD1L1;M	0.5	0.00432	2.36443	0.6641969	0.866841727	-0.203
cg13008174	II	37	8	11640960	NEIL2;NEIL2;NEIL2;	0.58	0.00041	3.39184	0.38507853	0.588167032	-0.203
cg06043201	II	37	8	28974428	KIF13B	0.62	0.0001	3.98291	0.46072088	0.663760222	-0.203
cg00219816	I	37	8	96280555	C8orf37	0.54	0.0014	2.85345	0.65364051	0.856264919	-0.203
cg10533990	II	37	11	71846733	FOLR3	0.54	0.0014	2.85345	0.4449892	0.647865872	-0.203
cg18589858	II	37	11	74860985	SLCO2B1;SLCO2B1	0.62	0.0001	3.98291	0.52473483	0.72772783	-0.203
cg06495803	II	37	12	6553489	CD27;LOC678655	0.58	0.00041	3.39184	0.69624574	0.493363385	0.203
cg11848173	II	37	12	56121015	CD63;CD63	0.54	0.0014	2.85345	0.38697494	0.590428397	-0.203
cg07051833	II	37	12	93808395	UBE2N	0.58	0.00041	3.39184	0.45474167	0.658184419	-0.203
cg16422365	I	37	14	106041833		0.62	0.0001	3.98291	0.53867941	0.335988079	0.203
cg17827208	II	37	15	99470414	IGF1R	0.58	0.00041	3.39184	0.61845686	0.821856975	-0.203
cg27090678	II	37	16	85514649		0.5	0.00432	2.36443	0.53886329	0.335958396	0.203
cg02371935	II	37	16	87521363	ZCCHC14	0.54	0.0014	2.85345	0.51304332	0.715634516	-0.203
cg11972721	II	37	17	17394732	MED9	0.46	0.01197	1.92207	0.65948327	0.456782184	0.203
cg22961402	II	37	17	27913793	GIT1;GIT1	0.58	0.00041	3.39184	0.62398944	0.826557277	-0.203
cg19389293	II	37	18	13641872	C18orf1;C18orf1;C1	0.46	0.01197	1.92207	0.2897263	0.492845726	-0.203
cg10819238	I	37	19	1155184	SBNO2	0.54	0.0014	2.85345	0.35384947	0.557300524	-0.203
cg23001905	II	37	19	1265302		0.58	0.00041	3.39184	0.6568298	0.453843437	0.203

cg07841371	I	37	1	1114912	TTLL10;TTLL10	0.58	0.00041	3.39184	0.60498701	0.403291728	0.202
cg10818566	II	37	2	47197798	TTC7A	0.46	0.01197	1.92207	0.33067257	0.532759587	-0.202
cg20738719	II	37	3	122728088	SEMA5B	0.54	0.0014	2.85345	0.37413191	0.576219239	-0.202
cg10880973	II	37	6	157982213	ZDHHC14;ZDHHC14	0.5	0.00432	2.36443	0.74970704	0.547586641	0.202
cg07180197	II	37	8	67039155	TRIM55;TRIM55;TR	0.54	0.0014	2.85345	0.64748009	0.445668594	0.202
cg10639435	II	37	8	146104221	ZNF250;ZNF250	0.67	2.34E-05	4.63072	0.67369931	0.472087972	0.202
cg13681496	II	37	9	95817516		0.58	0.00041	3.39184	0.27446529	0.476283527	-0.202
cg04236137	II	37	9	123655887		0.5	0.00432	2.36443	0.56377448	0.361832997	0.202
cg14611767	II	37	9	134127575		0.54	0.0014	2.85345	0.58248344	0.784786414	-0.202
cg04369957	II	37	11	8754756	ST5;ST5;ST5	0.46	0.01197	1.92207	0.69417617	0.491837634	0.202
cg01110839	II	37	11	47437783	SLC39A13;SLC39A1	0.62	0.0001	3.98291	0.60876164	0.810281239	-0.202
cg24422316	II	37	11	60930346	VPS37C	0.54	0.0014	2.85345	0.53269875	0.735031128	-0.202
cg01183510	II	37	12	66582582	IRAK3;IRAK3	0.46	0.01197	1.92207	0.38292257	0.585340604	-0.202
cg09075968	II	37	13	113841799	PCID2;PCID2;PCID2	0.54	0.0014	2.85345	0.54145535	0.743205404	-0.202
cg12437013	II	37	13	114161939	TMCO3	0.46	0.01197	1.92207	0.65740163	0.858917081	-0.202
cg01815801	II	37	13	114270609	TFDP1;TFDP1	0.58	0.00041	3.39184	0.54907939	0.750910666	-0.202
cg23013137	II	37	14	24676542	TSSK4	0.5	0.00432	2.36443	0.50159034	0.299108342	0.202
cg27518631	II	37	16	2846005		0.62	0.0001	3.98291	0.61224305	0.814295242	-0.202
cg10472651	II	37	16	66555859	TK2	0.54	0.0014	2.85345	0.35752168	0.559374835	-0.202
cg16768966	II	37	17	9940227	GAS7;GAS7	0.5	0.00432	2.36443	0.58961784	0.791419683	-0.202
cg05526498	II	37	17	49012893		0.54	0.0014	2.85345	0.55787379	0.759798193	-0.202
cg14885762	II	37	17	75446450	SEPT9;SEPT9;SEPT9	0.46	0.01197	1.92207	0.47148934	0.269706898	0.202
cg04425701	II	37	19	35630474	FXYD1;FXYD1;FXYD	0.58	0.00041	3.39184	0.48362164	0.685693896	-0.202
cg02524754	II	37	21	44985005	HSF2BP	0.58	0.00041	3.39184	0.41441157	0.616872461	-0.202
cg10511985	II	37	1	24053853		0.54	0.0014	2.85345	0.36190432	0.562561509	-0.201
cg10104487	II	37	1	27329335		0.62	0.0001	3.98291	0.4732879	0.674525539	-0.201
cg15518113	II	37	1	167400121	CD247;CD247	0.54	0.0014	2.85345	0.73633447	0.535089402	0.201
cg24537993	II	37	1	204275926	PLEKHA6	0.58	0.00041	3.39184	0.33807731	0.539116603	-0.201
cg09161446	I	37	2	240697468		0.58	0.00041	3.39184	0.61998992	0.419338495	0.201
cg23002590	II	37	6	167012092	RPS6KA2;RPS6KA2	0.5	0.00432	2.36443	0.55192745	0.75283063	-0.201
cg07212384	II	37	7	99761053	GAL3ST4	0.71	4.57E-06	5.34042	0.64221153	0.44147482	0.201
cg05992079	II	37	9	35650999	SIT1	0.58	0.00041	3.39184	0.44796179	0.24741612	0.201
cg24757160	I	37	11	67804112	NDUFS8	0.58	0.00041	3.39184	0.51984116	0.720619854	-0.201

cg07235053	II	37	11	94278581	FUT4	0.71	4.57E-06	5.34042	0.54737425	0.748596984	-0.201
cg12371569	II	37	12	13278271		0.5	0.00432	2.36443	0.24727344	0.448317846	-0.201
cg06611426	I	37	12	52404161	GRASP	0.62	0.0001	3.98291	0.46267604	0.663683543	-0.201
cg17707487	II	37	13	114261869	TFDP1;TFDP1	0.5	0.00432	2.36443	0.4427233	0.643323501	-0.201
cg09756109	I	37	16	1593178	IFT140;TMEM204	0.75	7.61E-07	6.11857	0.66474197	0.865663258	-0.201
cg08276042	I	37	16	2740348	KCTD5	0.54	0.0014	2.85345	0.47874466	0.680025097	-0.201
cg07089942	II	37	16	49666504	ZNF423	0.5	0.00432	2.36443	0.44868432	0.247863818	0.201
cg04466886	II	37	16	66521191		0.62	0.0001	3.98291	0.37250875	0.573796788	-0.201
cg04561791	II	37	16	85478769		0.54	0.0014	2.85345	0.45144581	0.25026956	0.201
cg16484912	II	37	17	74464334	AANAT;AANAT	0.46	0.01197	1.92207	0.54015928	0.339536216	0.201
cg04157865	II	37	17	77914162	TBC1D16	0.58	0.00041	3.39184	0.55403848	0.755279242	-0.201
cg12119693	II	37	17	80581925	WDR45L	0.58	0.00041	3.39184	0.48620481	0.687566132	-0.201
cg05641903	II	37	19	7767584	FCER2	0.58	0.00041	3.39184	0.4387228	0.639604759	-0.201
cg01772743	II	37	21	45576085		0.62	0.0001	3.98291	0.66317161	0.462596158	0.201
cg20686403	II	37	22	38438206		0.5	0.00432	2.36443	0.51492964	0.715927876	-0.201
cg05933510	II	37	1	9257092		0.67	2.34E-05	4.63072	0.60506286	0.804838616	-0.2
cg07571745	II	37	1	32715428	LCK	0.54	0.0014	2.85345	0.40156292	0.201726161	0.2
cg13804478	II	37	1	155158319	MUC1;MUC1;MUC[illegible]	0.46	0.01197	1.92207	0.49315355	0.292904286	0.2
cg15288326	II	37	1	200994098	KIF21B	0.62	0.0001	3.98291	0.63636098	0.836241091	-0.2
cg22489510	II	37	2	8675599		0.58	0.00041	3.39184	0.42962685	0.629325338	-0.2
cg10991454	II	37	2	30834674	LCLAT1;LCLAT1	0.62	0.0001	3.98291	0.5900597	0.790463347	-0.2
cg23410501	II	37	3	12937954		0.58	0.00041	3.39184	0.34246686	0.542866409	-0.2
cg01870865	II	37	3	48507087	TREX1;TREX1	0.5	0.00432	2.36443	0.25065142	0.45060919	-0.2
cg27022853	II	37	3	196377815	LRRC33	0.71	4.57E-06	5.34042	0.61237465	0.812011151	-0.2
cg15686615	II	37	4	6781565		0.58	0.00041	3.39184	0.46943152	0.669783775	-0.2
cg19268453	II	37	6	32905114	HLA-DMB	0.5	0.00432	2.36443	0.54561597	0.74552121	-0.2
cg11854981	II	37	6	42219847	TRERF1	0.54	0.0014	2.85345	0.48923595	0.689261224	-0.2
cg20332503	II	37	7	143081287	ZYX;ZYX	0.62	0.0001	3.98291	0.62035741	0.820655324	-0.2
cg00042325	II	37	7	150104655	LOC728743	0.5	0.00432	2.36443	0.51080581	0.310729834	0.2
cg16396223	II	37	7	151422150	PRKAG2;PRKAG2	0.42	0.02991	1.52413	0.52310653	0.323358848	0.2
cg24998197	II	37	8	60197574		0.5	0.00432	2.36443	0.52988145	0.329938154	0.2
cg07466463	I	37	8	141574207	EIF2C2;EIF2C2	0.5	0.00432	2.36443	0.60887476	0.808387686	-0.2
cg14120703	II	37	9	139416102	NOTCH1	0.54	0.0014	2.85345	0.52236492	0.7227816	-0.2

cg04930661	II	37	10	1531405	ADARB2	0.5	0.00432	2.36443	0.63041387	0.430867854	0.2
cg26169081	I	37	10	12648338	CAMK1D;CAMK1D	0.67	2.34E-05	4.63072	0.38752793	0.187511952	0.2
cg08560387	II	37	10	82247853	TSPAN14;TSPAN14	0.42	0.02991	1.52413	0.27149706	0.471694228	-0.2
cg17841267	I	37	10	112117449		0.54	0.0014	2.85345	0.77325357	0.573056669	0.2
cg03324175	II	37	11	61124464	CYBASC3;CYBASC3;	0.5	0.00432	2.36443	0.5037346	0.304229122	0.2
cg27317813	I	37	12	4398508	CCND2	0.58	0.00041	3.39184	0.27131068	0.470997564	-0.2
cg09842118	II	37	14	21359737	RNASE3	0.54	0.0014	2.85345	0.3463939	0.546558786	-0.2
cg23288563	II	37	14	21491469	NDRG2;NDRG2;NDI	0.5	0.00432	2.36443	0.36078594	0.560932368	-0.2
cg21141089	II	37	17	46949704		0.42	0.02991	1.52413	0.56472213	0.365134618	0.2
cg22992772	I	37	17	79030294	BAIAP2;BAIAP2;BAI	0.46	0.01197	1.92207	0.6972911	0.897199682	-0.2
cg04250920	II	37	17	79798629		0.62	0.0001	3.98291	0.40464557	0.604657792	-0.2
cg01627405	II	37	19	2235127	MIR1227;PLEKHJ1	0.5	0.00432	2.36443	0.50098582	0.700788608	-0.2
cg08418670	I	37	19	4374567	SH3GL1	0.58	0.00041	3.39184	0.71418654	0.914585687	-0.2
cg25734726	II	37	19	40948296	SERTAD3;SERTAD3;	0.62	0.0001	3.98291	0.59397747	0.794359737	-0.2
cg15089806	II	37	1	43418199	SLC2A1	0.58	0.00041	3.39184	0.44313103	0.243901167	0.199
cg23647554	I	37	3	42700674	ZBTB47	0.58	0.00041	3.39184	0.69233146	0.890993895	-0.199
cg18566313	II	37	4	186733428	SORBS2;SORBS2;SC	0.46	0.01197	1.92207	0.67774046	0.478503643	0.199
cg25578949	II	37	5	964230		0.46	0.01197	1.92207	0.66575297	0.466843616	0.199
cg04586126	I	37	5	176734633	MXD3;MXD3	0.58	0.00041	3.39184	0.52955163	0.72900648	-0.199
cg05352838	II	37	6	33384391	CUTA;CUTA;CUTA;C	0.54	0.0014	2.85345	0.7971694	0.597865005	0.199
cg01003666	II	37	7	139929429		0.67	2.34E-05	4.63072	0.6045706	0.405597543	0.199
cg26651303	I	37	7	158533204	ESYT2	0.5	0.00432	2.36443	0.26413678	0.46285265	-0.199
cg07160014	II	37	11	47959926		0.54	0.0014	2.85345	0.44037327	0.639667009	-0.199
cg15436385	II	37	11	72858122		0.5	0.00432	2.36443	0.52931144	0.330666845	0.199
cg15921911	II	37	14	69156160		0.54	0.0014	2.85345	0.57333581	0.772541644	-0.199
cg09873524	II	37	15	23062853	NIPA1;NIPA1	0.54	0.0014	2.85345	0.6814399	0.482058702	0.199
cg02849507	II	37	16	14051717		0.62	0.0001	3.98291	0.31703845	0.515565978	-0.199
cg10962223	II	37	17	16931836		0.5	0.00432	2.36443	0.57639451	0.376987782	0.199
cg06091647	II	37	17	78899392	RPTOR;RPTOR	0.46	0.01197	1.92207	0.80085909	0.601545099	0.199
cg16755833	I	37	17	80866063	TBCD	0.58	0.00041	3.39184	0.78448559	0.58536883	0.199
cg25103337	II	37	1	9293583	H6PD	0.54	0.0014	2.85345	0.46157921	0.659662355	-0.198
cg24375627	II	37	1	153509284	S100A6	0.67	2.34E-05	4.63072	0.38169165	0.579417282	-0.198
cg12449916	II	37	2	207847811		0.5	0.00432	2.36443	0.65421407	0.852408281	-0.198

cg00816240	II	37	2	232477075		0.54	0.0014	2.85345	0.50413584	0.702593902	-0.198
cg03169059	II	37	2	240145022	HDAC4	0.5	0.00432	2.36443	0.33511424	0.532991428	-0.198
cg01669161	II	37	7	1632265		0.46	0.01197	1.92207	0.38787128	0.190260487	0.198
cg21161403	I	37	8	1894604	ARHGEF10	0.58	0.00041	3.39184	0.57938314	0.77696844	-0.198
cg25642069	II	37	8	144408230	TOP1MT	0.54	0.0014	2.85345	0.53247371	0.730695444	-0.198
cg02304370	II	37	11	587926	PHRF1	0.46	0.01197	1.92207	0.5812427	0.779721034	-0.198
cg23478547	I	37	11	69259265		0.54	0.0014	2.85345	0.71862176	0.916147497	-0.198
cg11412713	II	37	11	94278413	FUT4	0.67	2.34E-05	4.63072	0.51836619	0.716080179	-0.198
cg03363565	II	37	16	474528	RAB11FIP3	0.58	0.00041	3.39184	0.46582334	0.664142745	-0.198
cg23599820	II	37	17	73456199	KIAA0195	0.46	0.01197	1.92207	0.35931811	0.556875894	-0.198
cg02807192	II	37	17	75524111		0.58	0.00041	3.39184	0.41083575	0.212541034	0.198
cg13103051	II	37	18	44260790	ST8SIA5	0.5	0.00432	2.36443	0.58036941	0.382192378	0.198
cg27585074	II	37	19	1947960	CSNK1G2	0.46	0.01197	1.92207	0.48681118	0.685051258	-0.198
cg05955436	II	37	20	17660838	RRBP1;RRBP1	0.46	0.01197	1.92207	0.3191582	0.51765472	-0.198
cg08721026	II	37	1	31575349		0.62	0.0001	3.98291	0.34885579	0.545572683	-0.197
cg17411016	II	37	2	47100912		0.54	0.0014	2.85345	0.35230822	0.549406957	-0.197
cg26394380	II	37	2	85896001	SFTPB;SFTPB	0.46	0.01197	1.92207	0.50326771	0.306399957	0.197
cg14813485	II	37	2	196460621		0.5	0.00432	2.36443	0.66095536	0.463525547	0.197
cg08180187	II	37	4	24796689	SOD3	0.62	0.0001	3.98291	0.58140882	0.778603461	-0.197
cg03957124	II	37	6	37016869		0.5	0.00432	2.36443	0.44206152	0.245029308	0.197
cg04153882	I	37	7	5270928	WIPI2;WIPI2;WIPI2	0.54	0.0014	2.85345	0.6381336	0.440837558	0.197
cg07807757	II	37	7	50894570		0.58	0.00041	3.39184	0.4654222	0.662197993	-0.197
cg20450123	II	37	8	19540210	CSGALNACT1;CSGA	0.5	0.00432	2.36443	0.35687893	0.553915904	-0.197
cg16335643	II	37	8	101569405	ANKRD46	0.54	0.0014	2.85345	0.65678208	0.853298426	-0.197
cg00005619	II	37	11	47608722	FAM180B	0.54	0.0014	2.85345	0.62105359	0.818130134	-0.197
cg09175485	II	37	11	67020555	KDM2A;KDM2A	0.46	0.01197	1.92207	0.29652886	0.493915473	-0.197
cg20140034	II	37	11	111250355	POU2AF1	0.46	0.01197	1.92207	0.46736163	0.270004173	0.197
cg14830166	II	37	12	11821908	ETV6	0.58	0.00041	3.39184	0.2618173	0.458722332	-0.197
cg08164191	II	37	14	65623632		0.46	0.01197	1.92207	0.39742871	0.594334107	-0.197
cg20417351	II	37	17	424358	VPS53	0.5	0.00432	2.36443	0.56619776	0.762784283	-0.197
cg14018648	II	37	17	7083015	ASGR1	0.62	0.0001	3.98291	0.48702532	0.684343334	-0.197
cg08044694	II	37	19	15391927	BRD4;BRD4	0.46	0.01197	1.92207	0.41577391	0.612369145	-0.197
cg01613294	II	37	22	36557607	APOL3;APOL3;APOI	0.54	0.0014	2.85345	0.56969346	0.37276235	0.197

cg13798679	II	37	1	36617570		0.54	0.0014	2.85345	0.52264872	0.32637226	0.196
cg20240347	I	37	1	204465584		0.54	0.0014	2.85345	0.57714759	0.380881533	0.196
cg06721411	II	37	2	74753759	DQX1	0.5	0.00432	2.36443	0.35604595	0.552544104	-0.196
cg27368523	II	37	2	113544348	IL1A	0.46	0.01197	1.92207	0.29343872	0.489505185	-0.196
cg14652403	I	37	4	993803	IDUA	0.62	0.0001	3.98291	0.55445784	0.358842272	0.196
cg00802478	II	37	4	40588553	RBM47	0.58	0.00041	3.39184	0.24493331	0.440802563	-0.196
cg03840351	II	37	5	290917	PDCD6	0.5	0.00432	2.36443	0.76582284	0.569792629	0.196
cg22809017	II	37	5	169373347	FAM196B;DOCK2	0.67	2.34E-05	4.63072	0.57146656	0.767160138	-0.196
cg19878482	I	37	8	144655026	C8orf73	0.5	0.00432	2.36443	0.48276247	0.678492811	-0.196
cg26382697	II	37	11	2406712	CD81	0.54	0.0014	2.85345	0.40215689	0.206041658	0.196
cg04353769	II	37	11	59951557	MS4A6A;MS4A6A;M	0.54	0.0014	2.85345	0.3794324	0.5750745	-0.196
cg13361558	I	37	11	73359962	PLEKHB1;PLEKHB1;	0.54	0.0014	2.85345	0.54179245	0.345695117	0.196
cg27149179	II	37	11	86451848		0.54	0.0014	2.85345	0.49701239	0.692794592	-0.196
cg14592365	II	37	11	111288100		0.5	0.00432	2.36443	0.35335039	0.157620726	0.196
cg20997661	II	37	11	125120940	PKNOX2	0.5	0.00432	2.36443	0.46708455	0.663472566	-0.196
cg09636302	I	37	12	96389483	HAL	0.5	0.00432	2.36443	0.70545502	0.901851048	-0.196
cg26426437	I	37	12	121686502	CAMKK2;CAMKK2;C	0.58	0.00041	3.39184	0.74402771	0.54787758	0.196
cg26962618	I	37	15	70767183		0.5	0.00432	2.36443	0.26693111	0.463350947	-0.196
cg00401972	II	37	16	66400404	CDH5	0.67	2.34E-05	4.63072	0.43825052	0.634197086	-0.196
cg27027055	II	37	16	79306292		0.58	0.00041	3.39184	0.67495734	0.479439707	0.196
cg09144769	II	37	17	34053056	AP2B1;AP2B1	0.54	0.0014	2.85345	0.56981802	0.765654233	-0.196
cg08857797	II	37	17	40927699	VPS25	0.54	0.0014	2.85345	0.44505026	0.640993891	-0.196
cg24726137	II	37	17	74524852	CYGB	0.5	0.00432	2.36443	0.49371221	0.297936207	0.196
cg05604874	II	37	17	80200785		0.46	0.01197	1.92207	0.54992902	0.746361504	-0.196
cg17477806	I	37	19	4543865	SEMA6B	0.54	0.0014	2.85345	0.59371606	0.790120342	-0.196
cg01468567	II	37	19	49843922	TEAD2	0.46	0.01197	1.92207	0.47170872	0.275588748	0.196
cg10126923	II	37	19	51875451	NKG7	0.54	0.0014	2.85345	0.42225118	0.61823629	-0.196
cg11806633	II	37	1	17887065	ARHGEF10L	0.58	0.00041	3.39184	0.50133476	0.696042032	-0.195
cg01479187	II	37	2	43158610		0.62	0.0001	3.98291	0.38931699	0.584785198	-0.195
cg11752275	II	37	2	85921036	GNLY;GNLY	0.54	0.0014	2.85345	0.53005183	0.724576138	-0.195
cg00691123	II	37	3	11632974	VGLL4;VGLL4	0.54	0.0014	2.85345	0.68897432	0.88390383	-0.195
cg15501219	II	37	3	44375962		0.5	0.00432	2.36443	0.55410775	0.749014453	-0.195
cg05380759	II	37	3	45579354	LARS2	0.67	2.34E-05	4.63072	0.38572493	0.581090311	-0.195

cg18244487	I	37	4	681280	MFSD7	0.58	0.00041	3.39184	0.50170987	0.696320949	-0.195
cg03234557	II	37	4	99404186	TSPAN5	0.62	0.0001	3.98291	0.74305798	0.547913444	0.195
cg19769715	II	37	5	149636363	CAMK2A;CAMK2A	0.46	0.01197	1.92207	0.67835685	0.483257531	0.195
cg11562411	II	37	6	35206618	SCUBE3	0.62	0.0001	3.98291	0.45796857	0.652910955	-0.195
cg10575547	II	37	6	42283846	TRERF1	0.54	0.0014	2.85345	0.50053444	0.695823796	-0.195
cg18995788	I	37	8	62673444		0.46	0.01293	1.88854	0.74140949	0.936766334	-0.195
cg13718827	I	37	9	131008520	MIR199B;DNM1;DN	0.54	0.0014	2.85345	0.65680854	0.851522362	-0.195
cg13807386	II	37	9	139098502	QSOX2	0.58	0.00041	3.39184	0.44756232	0.642749598	-0.195
cg18034368	II	37	10	127262250	LOC100169752	0.54	0.0014	2.85345	0.6705517	0.475503812	0.195
cg09854615	I	37	11	69259788		0.62	0.0001	3.98291	0.71768709	0.912690188	-0.195
cg11035519	II	37	11	130051458	ST14	0.5	0.00432	2.36443	0.46402954	0.268894119	0.195
cg03624316	II	37	12	53693054	PFDN5;C12orf10;PF	0.58	0.00041	3.39184	0.39650862	0.591954233	-0.195
cg02867735	II	37	14	22975384		0.5	0.00432	2.36443	0.76649671	0.571124663	0.195
cg26290716	II	37	14	91862813	CCDC88C	0.54	0.0014	2.85345	0.48744056	0.682314016	-0.195
cg19009471	I	37	14	101908998		0.67	2.34E-05	4.63072	0.40012299	0.205305397	0.195
cg12626076	II	37	15	92399195	SLCO3A1;SLCO3A1	0.54	0.0014	2.85345	0.70904944	0.513818201	0.195
cg06502279	II	37	16	474467	RAB11FIP3	0.58	0.00041	3.39184	0.58560144	0.781082038	-0.195
cg03678039	I	37	17	74525066	CYGB	0.5	0.00432	2.36443	0.78523181	0.590271386	0.195
cg04661929	II	37	17	75320035	SEPT9;SEPT9;SEPT9	0.54	0.0014	2.85345	0.35624653	0.161635235	0.195
cg23514211	I	37	17	79304188	TMEM105;TMEM10	0.71	4.57E-06	5.34042	0.55168096	0.746662108	-0.195
cg12106403	II	37	19	667808		0.71	4.57E-06	5.34042	0.28203053	0.477509482	-0.195
cg14389547	II	37	19	3398778	NFIC;NFIC	0.54	0.0014	2.85345	0.47744152	0.672318829	-0.195
cg00147638	II	37	1	25228039	RUNX3;RUNX3	0.5	0.00432	2.36443	0.79217592	0.598071133	0.194
cg15694146	II	37	1	27823372		0.46	0.01197	1.92207	0.50018903	0.306319553	0.194
cg01935086	II	37	1	32674191	IQCC;IQCC;DCDC2B	0.5	0.00432	2.36443	0.53609782	0.342258418	0.194
cg16110541	I	37	1	68679600	GPR177;GPR177	0.46	0.01197	1.92207	0.52950338	0.723984958	-0.194
cg13222915	II	37	1	184598594		0.58	0.00041	3.39184	0.5002196	0.694633293	-0.194
cg20334115	II	37	1	226107899	PYCR2	0.62	0.0001	3.98291	0.39228964	0.586376797	-0.194
cg19284751	II	37	2	119699789	MARCO;MARCO	0.5	0.00432	2.36443	0.43528994	0.629001009	-0.194
cg17102910	II	37	7	1126249	GPER;C7orf50;C7or	0.5	0.00432	2.36443	0.52980014	0.335387464	0.194
cg18041271	II	37	8	11756069		0.54	0.0014	2.85345	0.64918574	0.842798709	-0.194
cg17910564	II	37	8	42248702	VDAC3;VDAC3	0.5	0.00432	2.36443	0.71123403	0.516991118	0.194
cg09376835	II	37	8	131347294	ASAP1	0.58	0.00041	3.39184	0.40730311	0.60095388	-0.194

cg22637435	I	37	8	141361108	TRAPPC9;TRAPPC9	0.5	0.00432	2.36443	0.62264348	0.81704111	-0.194
cg24949488	II	37	10	98064362	DNTT;DNTT	0.46	0.01197	1.92207	0.63788071	0.443527657	0.194
cg24427660	II	37	11	818892	PNPLA2	0.58	0.00041	3.39184	0.52589032	0.719943557	-0.194
cg19546057	II	37	11	128776216	C11orf45;KCNJ5	0.58	0.00041	3.39184	0.33428404	0.528159508	-0.194
cg01668099	II	37	11	130026798		0.62	0.0001	3.98291	0.40467665	0.598986153	-0.194
cg14622549	I	37	12	132549292	EP400	0.46	0.01197	1.92207	0.70266944	0.508776203	0.194
cg06617636	I	37	14	69256690	ZFP36L1	0.58	0.00041	3.39184	0.77508613	0.580607385	0.194
cg09734761	II	37	16	85569506		0.58	0.00041	3.39184	0.54953701	0.743467233	-0.194
cg20719001	II	37	17	79297435	TMEM105	0.46	0.01197	1.92207	0.35506687	0.549497634	-0.194
cg22137236	II	37	18	46506533		0.54	0.0014	2.85345	0.51199507	0.705725551	-0.194
cg23682913	II	37	1	2080710	PRKCZ;PRKCZ;PRKC	0.46	0.01197	1.92207	0.42630942	0.619272503	-0.193
cg27540865	I	37	1	243053934		0.58	0.00041	3.39184	0.17216505	0.365350193	-0.193
cg00718513	II	37	2	89215272		0.46	0.01197	1.92207	0.62934792	0.436414053	0.193
cg18352516	II	37	3	177315226		0.5	0.00432	2.36443	0.31992609	0.512603759	-0.193
cg06305891	II	37	3	195897904		0.5	0.00432	2.36443	0.33771437	0.530772617	-0.193
cg12828656	II	37	5	132577108	FSTL4	0.58	0.00041	3.39184	0.34296812	0.536288925	-0.193
cg12117227	II	37	6	485971	EXOC2	0.46	0.01197	1.92207	0.51854241	0.325980463	0.193
cg00010853	II	37	6	30653167	KIAA1949;KIAA194	0.62	0.0001	3.98291	0.36133421	0.168544556	0.193
cg09667606	I	37	6	158507930	SYNJ2	0.54	0.0014	2.85345	0.54915385	0.742639786	-0.193
cg02850815	II	37	8	144896183	SCRIB;SCRIB;MIR93	0.54	0.0014	2.85345	0.6245903	0.817764123	-0.193
cg12756527	II	37	10	11284548	CUGBP2;CUGBP2;C	0.46	0.01197	1.92207	0.40694908	0.214103691	0.193
cg12661092	II	37	10	98861029	SLIT1	0.42	0.02991	1.52413	0.5907625	0.398172625	0.193
cg26391219	II	37	11	3189207		0.58	0.00041	3.39184	0.4680715	0.661006278	-0.193
cg24255209	I	37	11	72863808		0.54	0.0014	2.85345	0.69364347	0.500197517	0.193
cg09680149	II	37	12	6487052	SCNN1A	0.62	0.0001	3.98291	0.54547049	0.738794737	-0.193
cg20181887	I	37	12	123753272	CDK2AP1	0.42	0.02991	1.52413	0.34968178	0.542344512	-0.193
cg24659858	I	37	13	24270321		0.58	0.00041	3.39184	0.53859337	0.345780123	0.193
cg18981338	II	37	14	24422419	DHRS4;C14orf167;C	0.62	0.0001	3.98291	0.44455466	0.637426798	-0.193
cg19769147	I	37	14	105860954	PACS2;PACS2	0.71	4.57E-06	5.34042	0.49778035	0.690995555	-0.193
cg08494738	II	37	16	4369512		0.58	0.00041	3.39184	0.63220875	0.439614133	0.193
cg09481483	II	37	16	11734383		0.67	2.34E-05	4.63072	0.31277818	0.505660185	-0.193
cg04703221	II	37	16	69967063	WWP2;MIR140;WV	0.46	0.01197	1.92207	0.43261334	0.625451478	-0.193
cg27579854	II	37	17	80818992	TBCD	0.5	0.00432	2.36443	0.65383018	0.461265134	0.193

cg22459924	II	37	19	2607850	GNG7	0.54	0.0014	2.85345	0.65422239	0.461546989	0.193
cg20117260	II	37	19	41937434	ATP5SL;ATP5SL;ATF	0.58	0.00041	3.39184	0.53401742	0.727133418	-0.193
cg19327615	II	37	20	19955436	RIN2	0.54	0.0014	2.85345	0.39119827	0.583784557	-0.193
cg16394290	II	37	1	36788416	FAM176B	0.54	0.0014	2.85345	0.18881867	0.380779786	-0.192
cg07198150	II	37	1	86623101	COL24A1	0.5	0.00432	2.36443	0.4839661	0.675554926	-0.192
cg24197477	II	37	1	202975221	TMEM183B;TMEM:	0.58	0.00041	3.39184	0.67092267	0.862584264	-0.192
cg13359998	II	37	1	230241764	GALNT2	0.5	0.00432	2.36443	0.28948336	0.480999372	-0.192
cg15582102	II	37	2	47746468		0.5	0.00432	2.36443	0.82432091	0.632691241	0.192
cg18329052	II	37	2	219246707	SLC11A1	0.42	0.02991	1.52413	0.29713191	0.488746518	-0.192
cg13388731	II	37	2	220143225	DNAJB2;DNAJB2	0.5	0.00432	2.36443	0.62580472	0.433512428	0.192
cg24169315	I	37	2	242048359	PASK	0.54	0.0014	2.85345	0.46141155	0.26980653	0.192
cg08510456	II	37	3	49591008	BSN	0.5	0.00432	2.36443	0.33318575	0.524988467	-0.192
cg01381934	I	37	3	52529064	STAB1	0.62	0.0001	3.98291	0.37479896	0.567276938	-0.192
cg20954870	II	37	5	173070254		0.5	0.00432	2.36443	0.67201707	0.86430159	-0.192
cg10568066	I	37	6	30039442	RNF39;RNF39	0.54	0.0014	2.85345	0.12652154	0.318473654	-0.192
cg07346187	II	37	6	149775621	ZC3H12D	0.58	0.00041	3.39184	0.27576408	0.468083952	-0.192
cg25834613	I	37	7	1915315	MAD1L1;MAD1L1;N	0.5	0.00432	2.36443	0.43966771	0.631625575	-0.192
cg03121834	II	37	7	99970448	PILRA;PILRA;PILRA	0.5	0.00432	2.36443	0.38525161	0.577398053	-0.192
cg21210409	II	37	7	140015868		0.5	0.00432	2.36443	0.57429725	0.766750321	-0.192
cg04407063	II	37	11	67051977	ADRBK1	0.54	0.0014	2.85345	0.44178595	0.633820897	-0.192
cg13766864	II	37	11	74172851	KCNE3	0.5	0.00432	2.36443	0.6455493	0.837927995	-0.192
cg14343713	II	37	11	74742501		0.58	0.00041	3.39184	0.48958308	0.681311698	-0.192
cg14089267	I	37	13	23412409		0.42	0.02991	1.52413	0.43160494	0.239385184	0.192
cg11366363	II	37	13	51641069	GUCY1B2	0.67	2.34E-05	4.63072	0.51373084	0.705268763	-0.192
cg20847766	II	37	13	111840490	ARHGEF7;ARHGEF7	0.54	0.0014	2.85345	0.65717385	0.465258109	0.192
cg06618497	II	37	14	102198604	C14orf72	0.62	0.0001	3.98291	0.58282272	0.774392797	-0.192
cg07202353	II	37	15	42210119	EHD4	0.71	4.57E-06	5.34042	0.45268449	0.644869038	-0.192
cg05484949	I	37	17	38708465		0.46	0.01197	1.92207	0.78612563	0.594581009	0.192
cg08539991	II	37	19	36203832	ZBTB32;ZBTB32	0.58	0.00041	3.39184	0.67323865	0.481263136	0.192
cg21534299	II	37	1	44821474	ERI3	0.58	0.00041	3.39184	0.63268345	0.823343446	-0.191
cg26394055	II	37	1	161184921	FCER1G	0.54	0.0014	2.85345	0.33063133	0.521883745	-0.191
cg19922137	I	37	1	210111561	SYT14;SYT14;SYT14	0.54	0.0014	2.85345	0.08245993	0.273700659	-0.191
cg00027232	II	37	2	85547231	TGOLN2	0.58	0.00041	3.39184	0.68584733	0.876983625	-0.191

cg08462501	II	37	4	38138950	TBC1D1	0.54	0.0014	2.85345	0.65648907	0.847717015	-0.191
cg06612130	II	37	4	185269299		0.46	0.01197	1.92207	0.52292221	0.331674176	0.191
cg24721647	II	37	4	185726836	ACSL1	0.54	0.0014	2.85345	0.71074293	0.519762443	0.191
cg18135683	II	37	5	171856063	SH3PXD2B	0.62	0.0001	3.98291	0.50386388	0.695055018	-0.191
cg20017123	I	37	6	2763919		0.54	0.0014	2.85345	0.66255618	0.85350439	-0.191
cg23232773	II	37	6	31431902	HCP5	0.5	0.00432	2.36443	0.66504914	0.473819179	0.191
cg09371409	I	37	7	1632025		0.46	0.01197	1.92207	0.46265923	0.271529483	0.191
cg12964697	II	37	7	2140231	MAD1L1;MAD1L1;I	0.54	0.0014	2.85345	0.48444041	0.293077525	0.191
cg04261496	II	37	7	4753002	FOXK1	0.42	0.02991	1.52413	0.41471578	0.605323681	-0.191
cg26065247	II	37	7	37734052		0.62	0.0001	3.98291	0.38627113	0.577496904	-0.191
cg19911880	II	37	8	131353969	ASAP1	0.58	0.00041	3.39184	0.63923249	0.830409892	-0.191
cg25389145	II	37	8	141220490	TRAPPC9;TRAPPC9	0.67	2.34E-05	4.63072	0.59729072	0.788685192	-0.191
cg08081323	II	37	11	57232407	RTN4RL2	0.54	0.0014	2.85345	0.5464953	0.737837928	-0.191
cg25752272	II	37	11	61110107	DAK	0.62	0.0001	3.98291	0.50986511	0.700560774	-0.191
cg21702188	II	37	11	62574403	STX5;NXF1;NXF1	0.46	0.01197	1.92207	0.28837795	0.479867535	-0.191
cg12016746	II	37	11	68084453	LRP5	0.54	0.0014	2.85345	0.53286212	0.723902884	-0.191
cg11566441	II	37	11	111887338	DIXDC1;DIXDC1	0.46	0.01197	1.92207	0.66646498	0.47558026	0.191
cg22335223	I	37	11	117698911	FXYD2	0.62	0.0001	3.98291	0.52398003	0.33340635	0.191
cg00808175	II	37	12	6949119	GNB3	0.5	0.00432	2.36443	0.62805887	0.437037985	0.191
cg12962778	II	37	12	54778312	ZNF385A;ZNF385A;	0.67	2.34E-05	4.63072	0.53830298	0.72904184	-0.191
cg03514660	II	37	12	131418057		0.58	0.00041	3.39184	0.28345875	0.474844711	-0.191
cg25936902	II	37	13	30982971		0.54	0.0014	2.85345	0.47260892	0.663971674	-0.191
cg16962442	I	37	13	114828455	RASA3	0.58	0.00041	3.39184	0.45721157	0.648292096	-0.191
cg02947214	II	37	14	105998115		0.42	0.02991	1.52413	0.48156118	0.290804668	0.191
cg08008352	II	37	16	27322356		0.46	0.01197	1.92207	0.44678553	0.637842191	-0.191
cg23770271	II	37	17	3704471	ITGAE;ITGAE	0.5	0.00432	2.36443	0.50898466	0.699957679	-0.191
cg23115933	II	37	17	7814172	CHD3;CHD3;CHD3	0.54	0.0014	2.85345	0.41646871	0.607299761	-0.191
cg17753124	II	37	19	13259872	IER2;STX10	0.58	0.00041	3.39184	0.55616869	0.746738529	-0.191
cg27420674	II	37	19	49974121	ALDH16A1;ALDH16	0.5	0.00432	2.36443	0.62945299	0.438361988	0.191
cg24516259	I	37	1	2428864	PLCH2	0.54	0.0014	2.85345	0.75053706	0.560684485	0.19
cg20282814	II	37	1	43423072	SLC2A1	0.62	0.0001	3.98291	0.81025149	0.619888421	0.19
cg18854666	II	37	2	219247055	SLC11A1;SLC11A1	0.58	0.00041	3.39184	0.42557146	0.615108285	-0.19
cg10621597	II	37	4	140481801		0.54	0.0014	2.85345	0.32868171	0.518870255	-0.19

cg26209169	II	37	5	1316264		0.58	0.00041	3.39184	0.34394097	0.154114426	0.19
cg06947286	II	37	5	131596602	PDLIM4;PDLIM4	0.54	0.0014	2.85345	0.37709901	0.566725897	-0.19
cg01551441	II	37	5	158477707	EBF1	0.54	0.0014	2.85345	0.66455179	0.474421413	0.19
cg01702055	II	37	6	13303065		0.5	0.00432	2.36443	0.23089789	0.420636196	-0.19
cg18964319	II	37	6	36665554		0.5	0.00432	2.36443	0.37393403	0.563441115	-0.19
cg08442088	II	37	7	44085357	DBNL;DBNL;DBNL	0.58	0.00041	3.39184	0.41327124	0.603152937	-0.19
cg06576340	II	37	7	99954188	PILRB;PILRB;PILRB	0.5	0.00432	2.36443	0.29541449	0.485165289	-0.19
cg10789956	II	37	8	26467541	DPYSL2	0.54	0.0014	2.85345	0.3134405	0.503005433	-0.19
cg13819552	I	37	9	95799870		0.67	2.34E-05	4.63072	0.44831226	0.638343666	-0.19
cg24838825	II	37	10	111693923		0.5	0.00432	2.36443	0.32407711	0.514067156	-0.19
cg04886849	II	37	10	118997146		0.5	0.00432	2.36443	0.41932803	0.608986105	-0.19
cg25844587	II	37	15	91586374		0.46	0.01197	1.92207	0.57656709	0.386649913	0.19
cg00201196	II	37	16	8738469	C16orf68	0.58	0.00041	3.39184	0.63846581	0.828231455	-0.19
cg03916694	II	37	17	18150707	FLII	0.54	0.0014	2.85345	0.41029889	0.6004519	-0.19
cg16810031	I	37	17	38024146	ZPBP2;ZPBP2	0.58	0.00041	3.39184	0.75370441	0.563274527	0.19
cg23353000	II	37	17	42120896	LSM12	0.5	0.00432	2.36443	0.45232921	0.642288166	-0.19
cg08858426	II	37	17	75273896		0.67	2.34E-05	4.63072	0.65966662	0.849201628	-0.19
cg07786220	I	37	17	78683082	RPTOR;RPTOR	0.46	0.01197	1.92207	0.5247324	0.71506368	-0.19
cg02251850	II	37	17	78851503	RPTOR;RPTOR	0.5	0.00432	2.36443	0.5432078	0.353396923	0.19
cg10039047	I	37	17	80922528	B3GNTL1	0.58	0.00041	3.39184	0.58213549	0.392030942	0.19
cg17344770	II	37	19	3537694	C19orf71	0.46	0.01197	1.92207	0.58222105	0.772653378	-0.19
cg27172287	II	37	22	46974976		0.62	0.0001	3.98291	0.51567451	0.706092423	-0.19
cg06865642	II	37	22	50174028	BRD1;LOC90834	0.54	0.0014	2.85345	0.61304272	0.802575442	-0.19
cg23275914	II	37	1	1114914	TTLL10;TTLL10	0.54	0.0014	2.85345	0.52243396	0.333825945	0.189
cg13897348	II	37	1	1549699	MIB2;MIB2;MIB2;M	0.5	0.00432	2.36443	0.37422248	0.562774601	-0.189
cg19620994	II	37	1	12774904	AADACL3;AADACL3	0.5	0.00432	2.36443	0.60542858	0.794810644	-0.189
cg07242931	II	37	1	26034297	MAN1C1	0.71	4.57E-06	5.34042	0.49985255	0.689078678	-0.189
cg11225405	II	37	1	117038246		0.54	0.0014	2.85345	0.6157161	0.804610595	-0.189
cg08202226	II	37	1	153896590	GATAD2B	0.54	0.0014	2.85345	0.42421601	0.612762854	-0.189
cg12079904	II	37	2	43195545		0.58	0.00041	3.39184	0.41161129	0.601046682	-0.189
cg17327171	II	37	2	161156221	RBMS1;RBMS1	0.5	0.00432	2.36443	0.45229875	0.641645058	-0.189
cg03771282	II	37	2	198062750	ANKRD44;ANKRD44	0.62	0.0001	3.98291	0.62646927	0.437865368	0.189
cg03887528	II	37	2	231090531	SP140;SP140;SP140	0.62	0.0001	3.98291	0.43234276	0.242897163	0.189

cg04519775	II	37	2	231849693		0.54	0.0014	2.85345	0.70409113	0.51494293	0.189
cg16620233	II	37	3	53781107	CACNA1D;CACNA1I	0.58	0.00041	3.39184	0.51272339	0.701904526	-0.189
cg06704969	I	37	3	194797099	C3orf21	0.5	0.00432	2.36443	0.64124876	0.830038811	-0.189
cg15270235	II	37	3	195682595		0.54	0.0014	2.85345	0.58645193	0.775946821	-0.189
cg26873329	II	37	5	32522933		0.62	0.0001	3.98291	0.59020377	0.779482321	-0.189
cg01143547	I	37	6	41903527	CCND3;CCND3;CCN	0.71	4.57E-06	5.34042	0.56409555	0.375532261	0.189
cg16245431	II	37	7	1005089	COX19	0.54	0.0014	2.85345	0.33668218	0.525866327	-0.189
cg03840694	II	37	7	139991704		0.54	0.0014	2.85345	0.49011634	0.678844598	-0.189
cg03600318	II	37	10	81708991	SFTPD	0.46	0.01197	1.92207	0.54462168	0.733193415	-0.189
cg15994604	II	37	11	34676683	EHF	0.67	2.34E-05	4.63072	0.45114578	0.639790125	-0.189
cg12161228	I	37	11	89224506	NOX4;NOX4;NOX4;	0.46	0.01197	1.92207	0.07251152	0.261718727	-0.189
cg27041424	II	37	12	6232979	VWF	0.5	0.00432	2.36443	0.82251021	0.633935248	0.189
cg15438951	II	37	12	96389694	HAL	0.58	0.00041	3.39184	0.65488506	0.84359735	-0.189
cg24673600	II	37	14	76005929	BATF	0.5	0.00432	2.36443	0.79787444	0.60868329	0.189
cg24405567	II	37	15	70787565		0.5	0.00432	2.36443	0.40723397	0.596402201	-0.189
cg07877964	II	37	16	9023363	USP7	0.42	0.02991	1.52413	0.6179632	0.429424955	0.189
cg19060895	II	37	17	5419436	NLRP1;NLRP1;NLRP	0.58	0.00041	3.39184	0.39382032	0.582361101	-0.189
cg14012082	I	37	17	56274407	EPX	0.46	0.01197	1.92207	0.51884118	0.707655812	-0.189
cg24489015	II	37	17	56316162	LPO;LPO;LPO	0.62	0.0001	3.98291	0.54024085	0.728910105	-0.189
cg21073459	II	37	19	54604098	OSCAR;OSCAR;OSC/	0.58	0.00041	3.39184	0.39485696	0.583910239	-0.189
cg14609407	II	37	20	43883172	SLPI	0.58	0.00041	3.39184	0.48864156	0.677144735	-0.189
cg27544405	II	37	1	14108975	PRDM2;PRDM2;PRI	0.58	0.00041	3.39184	0.51079767	0.699283807	-0.188
cg16555909	II	37	1	23721285	TCEA3	0.54	0.0014	2.85345	0.63211045	0.820052392	-0.188
cg21451906	II	37	1	161192549	APOA2	0.54	0.0014	2.85345	0.4136067	0.601635112	-0.188
cg22793142	II	37	1	207995752	LOC148696	0.42	0.02991	1.52413	0.61009326	0.422106316	0.188
cg01627252	II	37	2	31397283	CAPN14	0.5	0.00432	2.36443	0.68049563	0.868202809	-0.188
cg00901982	II	37	2	70257298		0.67	2.34E-05	4.63072	0.42359425	0.611517256	-0.188
cg05991009	II	37	2	102080674	RFX8	0.58	0.00041	3.39184	0.44461709	0.632936024	-0.188
cg06804344	I	37	3	128778699	GP9	0.46	0.01197	1.92207	0.42185538	0.233892806	0.188
cg09390241	I	37	4	8174148		0.67	2.34E-05	4.63072	0.4778056	0.289472029	0.188
cg14049990	II	37	5	1514140	LPCAT1	0.5	0.00432	2.36443	0.479126	0.667523271	-0.188
cg05573654	II	37	5	1518179	LPCAT1	0.5	0.00432	2.36443	0.56620071	0.754421525	-0.188
cg12845808	II	37	5	141338604	PCDH12;PCDH12	0.54	0.0014	2.85345	0.59983614	0.787599942	-0.188

cg06869454	II	37	6	3770466		0.54	0.0014	2.85345	0.61197275	0.424339669	0.188
cg16137928	II	37	6	30720491		0.58	0.00041	3.39184	0.34558618	0.533719013	-0.188
cg01157951	II	37	6	31540399	LTA;LTA	0.67	2.34E-05	4.63072	0.42022104	0.232636666	0.188
cg08246318	II	37	6	42894099		0.54	0.0014	2.85345	0.65383632	0.465430275	0.188
cg10389644	II	37	7	1575621	MAFK	0.62	0.0001	3.98291	0.42408076	0.612124268	-0.188
cg06971659	II	37	8	27941552	C8orf80	0.67	2.34E-05	4.63072	0.4564639	0.268044242	0.188
cg26953469	II	37	10	31872296		0.62	0.0001	3.98291	0.67613888	0.864167282	-0.188
cg09519218	II	37	10	126390317	FAM53B	0.62	0.0001	3.98291	0.37617847	0.564016011	-0.188
cg25427871	I	37	11	2920735	SLC22A18AS;SLC22	0.58	0.00041	3.39184	0.30421392	0.492436875	-0.188
cg04822495	II	37	12	94676688	PLXNC1	0.5	0.00432	2.36443	0.36573464	0.553493199	-0.188
cg24178897	II	37	14	69170010		0.54	0.0014	2.85345	0.50276435	0.69063958	-0.188
cg10278046	II	37	16	4802848	ZNF500	0.42	0.02991	1.52413	0.63699827	0.448750962	0.188
cg04045544	II	37	16	27298091		0.5	0.00432	2.36443	0.62855251	0.816209019	-0.188
cg14587065	II	37	17	3807247	P2RX1	0.58	0.00041	3.39184	0.51427273	0.702183895	-0.188
cg15787636	II	37	17	80866113	TBCD	0.58	0.00041	3.39184	0.6284343	0.440670738	0.188
cg08034986	II	37	1	26860677	RPS6KA1	0.54	0.0014	2.85345	0.4156274	0.228344718	0.187
cg21113746	II	37	1	59039556		0.54	0.0014	2.85345	0.81107969	0.624432107	0.187
cg03653817	II	37	1	181065498		0.5	0.00432	2.36443	0.58626111	0.399267504	0.187
cg06354916	I	37	1	223738388	CAPN8	0.62	0.0001	3.98291	0.49550084	0.682258007	-0.187
cg02829654	II	37	1	236046939		0.5	0.00432	2.36443	0.50882525	0.695572327	-0.187
cg19075787	I	37	2	105372087		0.5	0.00432	2.36443	0.29062671	0.477644778	-0.187
cg13071069	II	37	3	13005914	IQSEC1;IQSEC1	0.54	0.0014	2.85345	0.53836042	0.725834675	-0.187
cg17054060	I	37	5	3606081		0.46	0.01197	1.92207	0.30644473	0.493459072	-0.187
cg24561419	II	37	5	79553606		0.54	0.0014	2.85345	0.4558184	0.642360158	-0.187
cg22192069	II	37	6	13305324	TBC1D7;TBC1D7;TE	0.62	0.0001	3.98291	0.72624786	0.53922315	0.187
cg03591753	II	37	6	35659141	FKBP5	0.58	0.00041	3.39184	0.58362064	0.770147991	-0.187
cg23416081	II	37	6	35693573	FKBP5	0.67	2.34E-05	4.63072	0.24485339	0.432327342	-0.187
cg02566775	II	37	6	144382964	PLAGL1;PLAGL1	0.58	0.00041	3.39184	0.4567526	0.643516465	-0.187
cg01911077	II	37	11	64531743		0.54	0.0014	2.85345	0.29757116	0.11029208	0.187
cg23896972	II	37	12	122079102	ORAI1	0.54	0.0014	2.85345	0.5425307	0.355483401	0.187
cg14502319	II	37	12	125167409		0.71	4.57E-06	5.34042	0.51009494	0.697438982	-0.187
cg16045731	II	37	14	24777708	LTB4R2;CIDEB	0.58	0.00041	3.39184	0.62237631	0.809429016	-0.187
cg07122905	II	37	14	92333765	TC2N;TC2N	0.75	7.61E-07	6.11857	0.43764707	0.250647888	0.187

cg03836521	I	37	16	30721270	SNORA30;SRCAP	0.58	0.00041	3.39184	0.60285698	0.789530213	-0.187
cg22319147	II	37	16	66400599	CDH5;CDH5	0.54	0.0014	2.85345	0.52396567	0.710783887	-0.187
cg05453411	II	37	16	89300123		0.62	0.0001	3.98291	0.49883983	0.685727876	-0.187
cg21846903	II	37	17	26697281	VTN;VTN	0.54	0.0014	2.85345	0.51825825	0.705529448	-0.187
cg18406792	II	37	18	13620807	C18orf1;C18orf1;C1	0.46	0.01197	1.92207	0.56517577	0.378547042	0.187
cg01418951	II	37	19	2486431		0.42	0.02991	1.52413	0.46682605	0.2793569	0.187
cg08244301	II	37	19	17610751	SLC27A1	0.5	0.00432	2.36443	0.36944093	0.556084642	-0.187
cg18467978	II	37	19	47134906		0.62	0.0001	3.98291	0.62487471	0.811966018	-0.187
cg00516092	II	37	20	31177275		0.54	0.0014	2.85345	0.32139178	0.508106334	-0.187
cg23889010	II	37	20	43882990	SLPI	0.54	0.0014	2.85345	0.57100064	0.758415985	-0.187
cg08155625	I	37	21	44851244		0.67	2.34E-05	4.63072	0.43480167	0.248255945	0.187
cg15536230	II	37	21	44985092	HSF2BP	0.54	0.0014	2.85345	0.39806768	0.584598549	-0.187
cg22595920	II	37	1	9716050	C1orf200;PIK3CD	0.62	0.0001	3.98291	0.36186962	0.547546556	-0.186
cg20416769	II	37	1	209824825	LAMB3;LAMB3;LAM	0.5	0.00432	2.36443	0.3308073	0.517185574	-0.186
cg13911707	II	37	1	247496404	ZNF496	0.58	0.00041	3.39184	0.50158306	0.687435284	-0.186
cg00710862	II	37	2	85545908	TGOLN2	0.58	0.00041	3.39184	0.48482048	0.670779787	-0.186
cg26047334	II	37	2	218785909	TNS1	0.58	0.00041	3.39184	0.60585054	0.419351178	0.186
cg05564251	II	37	2	231090640	SP140;SP140	0.54	0.0014	2.85345	0.66438513	0.478847612	0.186
cg05720721	II	37	3	123803128		0.46	0.01197	1.92207	0.42920188	0.243313416	0.186
cg03640151	II	37	4	8248912		0.54	0.0014	2.85345	0.65281354	0.838628664	-0.186
cg06758827	II	37	5	14492774	TRIO	0.58	0.00041	3.39184	0.65354948	0.839770971	-0.186
cg02637031	II	37	5	114887230		0.54	0.0014	2.85345	0.46612647	0.651756101	-0.186
cg20726195	I	37	6	2892148	SERPINB9	0.54	0.0014	2.85345	0.55673272	0.371158597	0.186
cg06762457	II	37	6	149806635	ZC3H12D	0.58	0.00041	3.39184	0.57830293	0.392035399	0.186
cg07698196	II	37	7	150485638	LOC100128542	0.54	0.0014	2.85345	0.62784923	0.813973978	-0.186
cg19016062	II	37	10	5904561	ANKRD16	0.5	0.00432	2.36443	0.72097832	0.534856452	0.186
cg22959742	II	37	10	13913931	FRMD4A	0.54	0.0014	2.85345	0.62363985	0.809605554	-0.186
cg03072035	II	37	10	73076664		0.62	0.0001	3.98291	0.56566044	0.751442182	-0.186
cg27449572	II	37	10	100024278	LOXL4	0.54	0.0014	2.85345	0.4209649	0.607404756	-0.186
cg10233454	II	37	12	57529389	LRP1	0.5	0.00432	2.36443	0.4697112	0.655493846	-0.186
cg19257402	II	37	12	132356988		0.5	0.00432	2.36443	0.41037911	0.596537259	-0.186
cg03029090	II	37	15	58812960	LIPC	0.5	0.00432	2.36443	0.39348074	0.579450929	-0.186
cg06208158	II	37	16	29168730		0.46	0.01197	1.92207	0.315492	0.501760281	-0.186

cg21658670	II	37	19	41930894	BCKDHA;BCKDHA	0.5	0.00432	2.36443	0.4624355	0.648717574	-0.186
cg26130864	II	37	20	3208100	SLC4A11	0.5	0.00432	2.36443	0.58929489	0.775716014	-0.186
cg21088514	II	37	21	35884376	KCNE1	0.67	2.34E-05	4.63072	0.65532519	0.840843579	-0.186
cg10871721	II	37	21	46235052	SUMO3	0.54	0.0014	2.85345	0.5151968	0.701505497	-0.186
cg24585377	II	37	1	26857774	RPS6KA1	0.58	0.00041	3.39184	0.32067915	0.505274674	-0.185
cg05802386	II	37	1	43422920	SLC2A1	0.54	0.0014	2.85345	0.75680409	0.57164893	0.185
cg03818307	II	37	1	150480534	ECM1;ECM1;ECM1;	0.58	0.00041	3.39184	0.4443175	0.629574002	-0.185
cg10951380	II	37	1	158029978	KIRREL	0.58	0.00041	3.39184	0.51275763	0.697921707	-0.185
cg01902845	II	37	1	214170993	PROX1	0.62	0.0001	3.98291	0.44231019	0.627340531	-0.185
cg24936095	II	37	2	109204230	LIMS1	0.58	0.00041	3.39184	0.46208892	0.646673323	-0.185
cg20287234	II	37	2	231789465	GPR55	0.54	0.0014	2.85345	0.41947501	0.604016722	-0.185
cg00116699	II	37	2	240205260	HDAC4	0.5	0.00432	2.36443	0.64909153	0.834441605	-0.185
cg17033891	II	37	3	159852976		0.5	0.00432	2.36443	0.15676981	0.341408207	-0.185
cg08928958	II	37	5	149178783	PPARGC1B	0.54	0.0014	2.85345	0.47317894	0.658350892	-0.185
cg17478979	I	37	6	149772150	ZC3H12D	0.5	0.00432	2.36443	0.49841987	0.683846344	-0.185
cg22311403	II	37	6	168352521	MLLT4;MLLT4;MLL	0.5	0.00432	2.36443	0.85058832	0.665134895	0.185
cg01259220	II	37	7	914964	C7orf20	0.42	0.02991	1.52413	0.70593907	0.520709026	0.185
cg21855816	II	37	7	1066024	C7orf50;C7orf50;C7	0.5	0.00432	2.36443	0.71688354	0.532311924	0.185
cg09187338	II	37	7	130588508		0.58	0.00041	3.39184	0.5536807	0.738358995	-0.185
cg24864097	II	37	10	121127614	GRK5	0.62	0.0001	3.98291	0.43320612	0.618417998	-0.185
cg13276580	II	37	10	125792941	CHST15	0.5	0.00432	2.36443	0.575311	0.760545392	-0.185
cg12394201	II	37	11	43942418		0.5	0.00432	2.36443	0.43595145	0.62111226	-0.185
cg21170682	II	37	11	122205382		0.58	0.00041	3.39184	0.68319227	0.868638771	-0.185
cg15007123	II	37	12	111798634	FAM109A	0.5	0.00432	2.36443	0.47296213	0.288372415	0.185
cg14127336	II	37	14	96180319	TCL1A;TCL1A	0.46	0.01197	1.92207	0.45169968	0.266984921	0.185
cg13164814	II	37	14	103468473	CDC42BPB	0.5	0.00432	2.36443	0.74213481	0.556725621	0.185
cg27664674	II	37	15	63654609	CA12;CA12	0.54	0.0014	2.85345	0.74628209	0.561145553	0.185
cg07076836	II	37	15	65175729		0.54	0.0014	2.85345	0.2159206	0.401343452	-0.185
cg03549146	I	37	16	69966902	MIR140;WWP2;WV	0.46	0.01197	1.92207	0.75285445	0.937521864	-0.185
cg06987053	II	37	16	87947779	CA5A	0.62	0.0001	3.98291	0.51773735	0.702238896	-0.185
cg18709110	I	37	17	1094555		0.62	0.0001	3.98291	0.65284194	0.838271943	-0.185
cg24678438	II	37	17	79484403		0.5	0.00432	2.36443	0.47474204	0.289371415	0.185
cg19787694	I	37	19	846117	PRTN3	0.46	0.01197	1.92207	0.55330534	0.738676223	-0.185

cg07366300	II	37	19	41934715	B3GNT8	0.67	2.34E-05	4.63072	0.40619654	0.591328181	-0.185
cg02737268	I	37	20	3780182	CDC25B;CDC25B;CI	0.58	0.00041	3.39184	0.37043777	0.185137263	0.185
cg26703511	I	37	21	44817420		0.58	0.00041	3.39184	0.71135145	0.525860756	0.185
cg24074477	II	37	22	17956455	CECR2	0.54	0.0014	2.85345	0.54520688	0.729717159	-0.185
cg25076881	II	37	1	159409836	OR10J1	0.54	0.0014	2.85345	0.74638071	0.562230876	0.184
cg13188263	II	37	1	160517539	CD84	0.42	0.02991	1.52413	0.55909244	0.375136656	0.184
cg05656486	II	37	1	161171383	NDUFS2;NDUFS2	0.54	0.0014	2.85345	0.39958859	0.583321329	-0.184
cg23738708	II	37	1	206240661	C1orf186	0.58	0.00041	3.39184	0.61291319	0.796567006	-0.184
cg20090162	I	37	2	47261900	TTC7A	0.42	0.02991	1.52413	0.35376874	0.537570446	-0.184
cg11690884	II	37	2	60533586		0.62	0.0001	3.98291	0.24769821	0.43126282	-0.184
cg24721964	II	37	3	150662697	CLRN1;CLRN1	0.46	0.01197	1.92207	0.40044246	0.584518268	-0.184
cg10959668	II	37	3	195897912		0.46	0.01197	1.92207	0.4182561	0.602353301	-0.184
cg03720100	II	37	6	30720263		0.54	0.0014	2.85345	0.21390047	0.398355226	-0.184
cg18338984	II	37	7	2646955	IQCE;IQCE	0.54	0.0014	2.85345	0.48728489	0.671165983	-0.184
cg14344864	II	37	7	101972144		0.42	0.02991	1.52413	0.71061788	0.526461376	0.184
cg06401414	I	37	8	141599436	EIF2C2;EIF2C2	0.58	0.00041	3.39184	0.65246962	0.468110088	0.184
cg14126884	II	37	9	134533116	RAPGEF1;RAPGEF1	0.62	0.0001	3.98291	0.7510518	0.567093821	0.184
cg01944288	II	37	9	135036217	NTNG2	0.5	0.00432	2.36443	0.33809634	0.522346986	-0.184
cg07011168	II	37	10	49797259	ARHGAP22	0.67	2.34E-05	4.63072	0.55107795	0.734775315	-0.184
cg15580458	II	37	10	121155124	GRK5	0.58	0.00041	3.39184	0.34842243	0.532306756	-0.184
cg04057956	II	37	12	6339200	CD9	0.54	0.0014	2.85345	0.63557185	0.819536518	-0.184
cg25015038	II	37	14	89604062		0.54	0.0014	2.85345	0.34252127	0.52609544	-0.184
cg04352288	I	37	16	87958407	CA5A	0.5	0.00432	2.36443	0.7124173	0.528091019	0.184
cg02525435	II	37	16	88977768	CBFA2T3;CBFA2T3	0.42	0.02991	1.52413	0.51702671	0.333092554	0.184
cg26960939	II	37	17	38717206	CCR7	0.5	0.00432	2.36443	0.32237049	0.138499199	0.184
cg15649236	II	37	17	46657504	MIR10A	0.62	0.0001	3.98291	0.23988897	0.423593823	-0.184
cg21715751	II	37	19	33795390	LOC80054	0.54	0.0014	2.85345	0.4382467	0.622369581	-0.184
cg26633373	II	37	19	54377836	MYADM;MYADM;M	0.54	0.0014	2.85345	0.55521888	0.739016136	-0.184
cg14030719	II	37	20	32266794	E2F1	0.46	0.01197	1.92207	0.60673371	0.422645231	0.184
cg19593285	I	37	20	32267661	E2F1	0.62	0.0001	3.98291	0.78071262	0.596515922	0.184
cg22726155	II	37	21	45575559		0.54	0.0014	2.85345	0.65792605	0.473998501	0.184
cg15121304	II	37	22	22380100		0.42	0.02991	1.52413	0.50614872	0.322385477	0.184
cg23659250	II	37	22	50174065	BRD1;LOC90834	0.58	0.00041	3.39184	0.61083473	0.794919161	-0.184

cg03693486	II	37	1	20914322	CDA	0.5	0.00432	2.36443	0.53816284	0.721443746	-0.183
cg09725874	II	37	1	27480106	SLC9A1	0.58	0.00041	3.39184	0.53470035	0.351768913	0.183
cg17329534	II	37	1	154980743	ZBTB7B	0.46	0.01197	1.92207	0.29187394	0.474627608	-0.183
cg10075819	II	37	2	109229337	LIMS1	0.54	0.0014	2.85345	0.67393257	0.491174243	0.183
cg17781925	II	37	3	49726151	MST1;MST1;RNF12	0.58	0.00041	3.39184	0.53941864	0.722317405	-0.183
cg20299697	I	37	3	138069423	MRAS;MRAS	0.62	0.0001	3.98291	0.64620346	0.829598495	-0.183
cg16272981	I	37	5	1489889	LPCAT1	0.54	0.0014	2.85345	0.69046342	0.507643531	0.183
cg02347002	II	37	5	138719090	SLC23A1;SLC23A1	0.54	0.0014	2.85345	0.41096963	0.593832128	-0.183
cg14646977	II	37	6	30656667	NRM;KIAA1949	0.46	0.01197	1.92207	0.53152679	0.348430955	0.183
cg10995925	II	37	6	31539601	LTA;LTA	0.58	0.00041	3.39184	0.67862418	0.49548345	0.183
cg07249939	II	37	6	31867995	ZBTB12	0.42	0.02991	1.52413	0.39822999	0.581670373	-0.183
cg26954951	II	37	6	167507568		0.54	0.0014	2.85345	0.43863929	0.255398551	0.183
cg19920134	II	37	7	38227870	STARD3NL	0.42	0.02991	1.52413	0.53165363	0.348488201	0.183
cg21210642	II	37	9	100881995	TRIM14;TRIM14;TR	0.54	0.0014	2.85345	0.51070717	0.694059429	-0.183
cg06374610	II	37	10	50328992		0.46	0.01197	1.92207	0.68727201	0.50425328	0.183
cg17737835	II	37	12	2027805	CACNA2D4;CACNA2	0.62	0.0001	3.98291	0.28475097	0.467946003	-0.183
cg17056618	II	37	13	50216117		0.58	0.00041	3.39184	0.72435228	0.907579436	-0.183
cg07036035	II	37	14	96133072	TCL6	0.46	0.01197	1.92207	0.62743104	0.444524259	0.183
cg06856840	II	37	15	80446451	FAH	0.62	0.0001	3.98291	0.32494861	0.507948689	-0.183
cg05413628	I	37	16	1521656	CLCN7;CLCN7	0.46	0.01197	1.92207	0.54392013	0.72717203	-0.183
cg16286735	II	37	17	45371778	ITGB3	0.42	0.02991	1.52413	0.58196085	0.399191206	0.183
cg02172058	II	37	17	58499911	C17orf64;C17orf64	0.54	0.0014	2.85345	0.54314717	0.725690458	-0.183
cg11746924	II	37	17	62007912	CD79B;CD79B;CD79	0.58	0.00041	3.39184	0.46589449	0.282762146	0.183
cg26701815	II	37	18	3446566	TGIF1;TGIF1	0.5	0.00432	2.36443	0.52109583	0.703943513	-0.183
cg09432758	II	37	19	1649123	TCF3;TCF3	0.46	0.01197	1.92207	0.52856327	0.345929171	0.183
cg10583119	II	37	19	54237755	MIR518D	0.5	0.00432	2.36443	0.44459702	0.26194389	0.183
cg18447740	II	37	19	54604187	OSCAR;OSCAR;OSCA	0.58	0.00041	3.39184	0.32828492	0.510923034	-0.183
cg13733708	II	37	20	44597685	ZNF335	0.58	0.00041	3.39184	0.62696255	0.809913564	-0.183
cg08139729	II	37	1	41984243	HIVEP3;HIVEP3	0.46	0.01197	1.92207	0.70110213	0.518794941	0.182
cg08127462	II	37	1	153514264	S100A5	0.58	0.00041	3.39184	0.57167308	0.754156899	-0.182
cg02543462	II	37	2	113885116	IL1RN;IL1RN;IL1RN;	0.5	0.00432	2.36443	0.27792012	0.459703098	-0.182
cg03192919	II	37	3	48412245	FBXW12;FBXW12;F	0.54	0.0014	2.85345	0.43326201	0.614985024	-0.182
cg10627428	II	37	3	122514814	DIRC2	0.71	4.57E-06	5.34042	0.35447239	0.536515131	-0.182

cg19118972	II	37	3	184323327		0.5	0.00432	2.36443	0.57586426	0.758288815	-0.182
cg01938023	II	37	5	138855699	TMEM173	0.58	0.00041	3.39184	0.4464049	0.628530537	-0.182
cg01538969	II	37	6	30624636	DHX16;DHX16	0.58	0.00041	3.39184	0.56374098	0.745414472	-0.182
cg03120555	I	37	7	630473	PRKAR1B;PRKAR1B	0.42	0.02991	1.52413	0.29032214	0.472641669	-0.182
cg03796224	II	37	8	30383628	RBPMS;RBPMS;RBF	0.42	0.02991	1.52413	0.74292947	0.561092216	0.182
cg13740985	II	37	9	80930413	PSAT1;PSAT1	0.58	0.00041	3.39184	0.23501542	0.417051789	-0.182
cg11261261	II	37	10	128772459	DOCK1	0.54	0.0014	2.85345	0.67112208	0.489383838	0.182
cg18112681	II	37	11	63303399	RARRES3	0.5	0.00432	2.36443	0.53293227	0.71524851	-0.182
cg22110158	II	37	11	130036542	ST14	0.54	0.0014	2.85345	0.60461223	0.422487172	0.182
cg26021714	II	37	14	21424117	RNASE2	0.54	0.0014	2.85345	0.59482201	0.776678759	-0.182
cg07989851	II	37	15	38117466		0.58	0.00041	3.39184	0.39342711	0.57528364	-0.182
cg14004161	I	37	15	64442561	SNX22	0.46	0.01197	1.92207	0.27783923	0.459619279	-0.182
cg07196571	I	37	15	64442578	SNX22	0.46	0.01197	1.92207	0.36329802	0.545543851	-0.182
cg16778107	II	37	16	381873	AXIN1;AXIN1	0.54	0.0014	2.85345	0.54497521	0.726965728	-0.182
cg09050670	I	37	16	1521617	CLCN7;CLCN7	0.54	0.0014	2.85345	0.16492607	0.346825197	-0.182
cg12125117	II	37	16	57701461	GPR97	0.62	0.0001	3.98291	0.45563268	0.637912168	-0.182
cg16392942	II	37	16	89909677	SPIRE2	0.62	0.0001	3.98291	0.43391691	0.615716979	-0.182
cg27039898	I	37	17	79799411		0.46	0.01197	1.92207	0.73990814	0.922353033	-0.182
cg22707397	I	37	19	49020528		0.54	0.0014	2.85345	0.6172913	0.435300852	0.182
cg21685427	II	37	20	42187356	SGK2	0.46	0.01197	1.92207	0.67035323	0.852426785	-0.182
cg22954818	II	37	22	39354115	APOBEC3A	0.58	0.00041	3.39184	0.53358141	0.715287478	-0.182
cg22627753	II	37	1	988623	AGRN	0.5	0.00432	2.36443	0.79136693	0.61083939	0.181
cg24955196	I	37	1	154982621	ZBTB7B	0.54	0.0014	2.85345	0.56858501	0.749410852	-0.181
cg14919082	II	37	2	54901055		0.54	0.0014	2.85345	0.50910209	0.690531382	-0.181
cg05007997	II	37	2	219246985	SLC11A1;SLC11A1	0.58	0.00041	3.39184	0.41956664	0.600146152	-0.181
cg16788857	II	37	3	192577951	C3orf59	0.42	0.02991	1.52413	0.62718826	0.446241118	0.181
cg24560729	I	37	4	1342394	KIAA1530	0.5	0.00432	2.36443	0.75911768	0.940285319	-0.181
cg16223546	II	37	5	158768976		0.5	0.00432	2.36443	0.28968024	0.470469466	-0.181
cg07768103	I	37	5	175956344	RNF44	0.5	0.00432	2.36443	0.69116203	0.510602399	0.181
cg24219974	II	37	6	14729722		0.58	0.00041	3.39184	0.56604703	0.384862219	0.181
cg05642546	II	37	7	37298927	ELMO1	0.58	0.00041	3.39184	0.48316565	0.664077731	-0.181
cg23258615	II	37	10	81961468	ANXA11;ANXA11;A	0.5	0.00432	2.36443	0.34677619	0.528085053	-0.181
cg12807237	II	37	10	134729045		0.42	0.02991	1.52413	0.56134967	0.380742408	0.181

cg10927968	I	37	11	1807333		0.5	0.00432	2.36443	0.61934884	0.800392143	-0.181
cg24909660	II	37	11	47276469	NR1H3	0.62	0.0001	3.98291	0.39149534	0.572654021	-0.181
cg13048008	II	37	11	71725340	NUMA1	0.71	4.57E-06	5.34042	0.54225456	0.723224058	-0.181
cg02423534	II	37	12	49160180	ADCY6;ADCY6	0.54	0.0014	2.85345	0.52869013	0.709339277	-0.181
cg04036049	I	37	12	57923710	MBD6	0.54	0.0014	2.85345	0.7184129	0.89977295	-0.181
cg00748494	I	37	13	23412343		0.42	0.02991	1.52413	0.41033747	0.229785924	0.181
cg16728323	II	37	13	111212174	RAB20	0.58	0.00041	3.39184	0.69266411	0.87317328	-0.181
cg01424562	II	37	14	69256677	ZFP36L1	0.58	0.00041	3.39184	0.68994209	0.509090144	0.181
cg25582185	I	37	14	106032933		0.54	0.0014	2.85345	0.46621034	0.285345012	0.181
cg27184903	II	37	15	29285727	APBA2;APBA2	0.54	0.0014	2.85345	0.37051313	0.551348664	-0.181
cg08180028	II	37	15	41796044	LTK;LTK;LTK	0.62	0.0001	3.98291	0.49204022	0.673436394	-0.181
cg06527213	II	37	15	86315211	KLHL25;MIR1276	0.62	0.0001	3.98291	0.60263952	0.783417001	-0.181
cg10313337	II	37	16	68823690	CDH1	0.62	0.0001	3.98291	0.64641289	0.827817825	-0.181
cg11692409	II	37	17	1665181	SERPINF1	0.58	0.00041	3.39184	0.55592648	0.737079799	-0.181
cg02294870	II	37	17	41479663		0.5	0.00432	2.36443	0.63093792	0.44963198	0.181
cg25291653	II	37	20	19867145		0.58	0.00041	3.39184	0.70294616	0.883514877	-0.181
cg04776489	II	37	22	22597965	VPREB1	0.46	0.01197	1.92207	0.52535198	0.344781823	0.181
cg04223044	II	37	22	45608428	C22orf9;C22orf9	0.54	0.0014	2.85345	0.64061234	0.822096105	-0.181
cg02058628	I	37	1	2164542	SKI	0.5	0.00432	2.36443	0.52336047	0.703695281	-0.18
cg09517873	I	37	1	12656315	DHRS3	0.46	0.01197	1.92207	0.61371434	0.794087053	-0.18
cg19260606	II	37	1	15100030	KIAA1026;KIAA102(	0.5	0.00432	2.36443	0.49080739	0.670520155	-0.18
cg00326300	II	37	1	33772032		0.46	0.01197	1.92207	0.58936052	0.408922533	0.18
cg09132102	II	37	1	56900436		0.67	2.34E-05	4.63072	0.63926442	0.459054074	0.18
cg21742790	II	37	3	138581702		0.62	0.0001	3.98291	0.52464144	0.704736058	-0.18
cg09530650	II	37	5	137024163	KLHL3	0.58	0.00041	3.39184	0.69371199	0.873817284	-0.18
cg16280132	I	37	6	31540459	LTA;LTA	0.62	0.0001	3.98291	0.49466636	0.314229135	0.18
cg11744817	II	37	6	166876826	RPS6KA2;RPS6KA2	0.58	0.00041	3.39184	0.16590569	0.346112165	-0.18
cg16409368	II	37	7	128047452	IMPDH1;IMPDH1;IN	0.5	0.00432	2.36443	0.66282227	0.482519611	0.18
cg07611843	II	37	7	128453143	CCDC136	0.67	2.34E-05	4.63072	0.53928803	0.718799975	-0.18
cg09069886	II	37	8	131000415		0.5	0.00432	2.36443	0.516766	0.696930528	-0.18
cg04189187	I	37	10	6183528		0.54	0.0014	2.85345	0.90192623	0.721538297	0.18
cg19148410	II	37	11	111250352	POU2AF1	0.54	0.0014	2.85345	0.43475372	0.254391569	0.18
cg19791714	II	37	11	118763901	CXCR5;CXCR5	0.71	4.57E-06	5.34042	0.52782236	0.347360907	0.18

cg27292547	II	37	12	1672440		0.5	0.00432	2.36443	0.52462028	0.704705219	-0.18
cg15460816	II	37	12	25150402	C12orf77	0.42	0.02991	1.52413	0.71271108	0.53319909	0.18
cg14874216	II	37	12	122651623	LRRC43	0.42	0.02991	1.52413	0.78964092	0.609437816	0.18
cg01867764	II	37	14	22986466		0.46	0.01197	1.92207	0.35527067	0.17529878	0.18
cg23893332	II	37	14	94576048	IFI27;IFI27	0.5	0.00432	2.36443	0.55736406	0.736945473	-0.18
cg06939852	II	37	16	51068651		0.46	0.01197	1.92207	0.40844414	0.228576757	0.18
cg07377994	II	37	17	55533142	MSI2;MSI2	0.54	0.0014	2.85345	0.28560018	0.465219707	-0.18
cg26534812	II	37	17	75524155		0.46	0.01197	1.92207	0.6351678	0.454774256	0.18
cg08363114	I	37	19	54711292	RPS9	0.62	0.0001	3.98291	0.68785619	0.867664551	-0.18
cg21211882	II	37	20	34770405	EPB41L1;EPB41L1	0.5	0.00432	2.36443	0.38409207	0.563980065	-0.18
cg21310090	II	37	20	44538669	PLTP;PLTP	0.58	0.00041	3.39184	0.50339327	0.683070041	-0.18
cg26236922	I	37	20	60912673	LAMA5	0.54	0.0014	2.85345	0.6068838	0.426516317	0.18
cg00388871	I	37	20	62530407	DNAJC5	0.46	0.01197	1.92207	0.53454289	0.714398392	-0.18
cg01183122	II	37	21	35884348	KCNE1	0.58	0.00041	3.39184	0.63520733	0.815199814	-0.18
cg12465678	II	37	1	27953336	FGR;FGR;FGR	0.46	0.01197	1.92207	0.3046752	0.4832545	-0.179
cg16815882	II	37	1	35908609	KIAA0319L	0.54	0.0014	2.85345	0.54280142	0.72146098	-0.179
cg08457158	I	37	1	41367714		0.54	0.0014	2.85345	0.49228387	0.312883181	0.179
cg17734802	II	37	1	54358862	DIO1;DIO1;DIO1;DI	0.46	0.01197	1.92207	0.31744548	0.496896841	-0.179
cg21812313	II	37	1	94158618		0.62	0.0001	3.98291	0.63979953	0.461033404	0.179
cg06872185	II	37	1	151831144		0.58	0.00041	3.39184	0.60146267	0.779992027	-0.179
cg16586406	II	37	1	154164994	TPM3	0.54	0.0014	2.85345	0.31923738	0.498174898	-0.179
cg05756492	II	37	1	206729685	RASSF5;RASSF5;RAS	0.58	0.00041	3.39184	0.75867468	0.580173763	0.179
cg01623771	II	37	2	239047774	KLHL30	0.46	0.01197	1.92207	0.5329154	0.354309501	0.179
cg18338046	II	37	5	133452188	TCF7;TCF7;TCF7;TC	0.54	0.0014	2.85345	0.72010774	0.541414969	0.179
cg10120652	II	37	8	9182252		0.58	0.00041	3.39184	0.53801361	0.71682031	-0.179
cg14412639	II	37	9	34098451	DCAF12	0.62	0.0001	3.98291	0.8001568	0.621338482	0.179
cg01544270	II	37	9	140300227	EXD3	0.62	0.0001	3.98291	0.34152463	0.520211049	-0.179
cg18825531	II	37	11	62321136		0.46	0.01197	1.92207	0.27017103	0.448803381	-0.179
cg01688936	II	37	16	30770794	C16orf93	0.67	2.34E-05	4.63072	0.47919033	0.657983909	-0.179
cg02814118	II	37	16	81528945	CMIP;CMIP	0.5	0.00432	2.36443	0.16248977	0.34112525	-0.179
cg05938409	II	37	16	85290060		0.5	0.00432	2.36443	0.61195869	0.43247744	0.179
cg14177325	II	37	17	1541763	SCARF1;SCARF1;SC/	0.5	0.00432	2.36443	0.58329974	0.762688173	-0.179
cg22443212	II	37	17	78253912	RNF213;RNF213	0.5	0.00432	2.36443	0.61735253	0.79683817	-0.179

cg01905773	II	37	17	79297618	TMEM105		0.54	0.0014	2.85345	0.47380162	0.653051458	-0.179
cg26620021	II	37	19	40788926	MIR641;AKT2		0.54	0.0014	2.85345	0.73258961	0.554043969	0.179
cg13760683	II	37	20	46988477	LOC284749		0.67	2.34E-05	4.63072	0.68441315	0.50587078	0.179
cg16531348	II	37	21	45377217	AGPAT3;AGPAT3		0.46	0.01197	1.92207	0.72602295	0.54681307	0.179
cg08039560	I	37	21	45575832			0.58	0.00041	3.39184	0.80732369	0.628778318	0.179
cg03432618	II	37	22	19704196		Aug-01	0.5	0.00432	2.36443	0.75116369	0.572177168	0.179
cg12509665	I	37	22	47082044	CERK		0.46	0.01197	1.92207	0.58083012	0.759876509	-0.179
cg20700740	I	37	1	9339683			0.54	0.0014	2.85345	0.71371632	0.89220077	-0.178
cg03537386	II	37	1	32155667	COL16A1		0.62	0.0001	3.98291	0.66626139	0.84440263	-0.178
cg03154580	II	37	2	121009176	RALB		0.46	0.01197	1.92207	0.36225971	0.540101878	-0.178
cg17098979	II	37	2	241562085			0.58	0.00041	3.39184	0.25705929	0.43461547	-0.178
cg14630001	II	37	3	66633408			0.67	2.34E-05	4.63072	0.4682174	0.290247135	0.178
cg09668964	II	37	3	129335695			0.5	0.00432	2.36443	0.35905823	0.537054426	-0.178
cg06801385	II	37	5	1152362			0.58	0.00041	3.39184	0.5574769	0.379395093	0.178
cg19696103	I	37	5	132354130	ZCCHC10		0.54	0.0014	2.85345	0.63598227	0.813507483	-0.178
cg00737979	II	37	5	134990285	LOC340074		0.58	0.00041	3.39184	0.71608306	0.537921909	0.178
cg18643093	II	37	5	150521257	ANXA6;ANXA6		0.62	0.0001	3.98291	0.27429919	0.095827903	0.178
cg10803218	II	37	5	177544994	N4BP3		0.42	0.02991	1.52413	0.57321946	0.395242038	0.178
cg24452282	II	37	6	31542740	TNF		0.46	0.01197	1.92207	0.47187259	0.294219379	0.178
cg06665622	II	37	6	46293571	RCAN2		0.54	0.0014	2.85345	0.75750013	0.579688614	0.178
cg08601457	II	37	6	112115117	FYN		0.46	0.01197	1.92207	0.27721406	0.455223319	-0.178
cg23143090	II	37	6	169689939			0.58	0.00041	3.39184	0.52423879	0.702409063	-0.178
cg01843768	II	37	7	2078650	MAD1L1;MAD1L1;M		0.54	0.0014	2.85345	0.68929583	0.51086318	0.178
cg20305578	II	37	8	1908301			0.54	0.0014	2.85345	0.34594223	0.524244763	-0.178
cg06576318	II	37	8	134249800	NDRG1;NDRG1		0.58	0.00041	3.39184	0.44446527	0.622088545	-0.178
cg04091927	II	37	8	141519270			0.46	0.01197	1.92207	0.45020988	0.272140762	0.178
cg13451093	II	37	9	137040612			0.54	0.0014	2.85345	0.63144558	0.809927572	-0.178
cg11169848	II	37	11	67142030	LOC100130987;CLC		0.67	2.34E-05	4.63072	0.30028345	0.478344265	-0.178
cg01686739	II	37	12	107855547	BTBD11		0.42	0.02991	1.52413	0.35567845	0.533615398	-0.178
cg05132828	II	37	12	123560479	PITPNM2		0.71	4.57E-06	5.34042	0.46906977	0.29122232	0.178
cg20911718	II	37	14	24867491	NYNRIN		0.54	0.0014	2.85345	0.51052418	0.688933853	-0.178
cg14191953	II	37	14	107048701			0.54	0.0014	2.85345	0.42519575	0.247180113	0.178
cg02463844	I	37	16	88152170			0.46	0.01197	1.92207	0.51515254	0.693252956	-0.178

cg04069539	II	37	16	89168963	ACSF3;ACSF3;ACSF3	0.71	4.57E-06	5.34042	0.63652477	0.814919478	-0.178
cg08003321	II	37	17	8815843	PIK3R5;PIK3R5	0.54	0.0014	2.85345	0.7913915	0.613372916	0.178
cg07898949	II	37	17	30821957	MYO1D	0.58	0.00041	3.39184	0.59279589	0.770409989	-0.178
cg08939850	I	37	17	78800806	RPTOR;RPTOR	0.62	0.0001	3.98291	0.2236624	0.401644315	-0.178
cg00926657	I	37	17	79880822	MAFG;MAFG	0.58	0.00041	3.39184	0.68700911	0.864853435	-0.178
cg00176309	II	37	21	46303258		0.62	0.0001	3.98291	0.69554123	0.873814245	-0.178
cg13868520	II	37	1	12139206	TNFRSF8	0.58	0.00041	3.39184	0.73448276	0.557002113	0.177
cg10708189	II	37	1	58898793		0.42	0.02991	1.52413	0.59499364	0.418075953	0.177
cg18081760	II	37	1	114483597	HIPK1;HIPK1	0.42	0.02991	1.52413	0.34414161	0.520896279	-0.177
cg04705952	II	37	1	160066587	IGSF8	0.71	4.57E-06	5.34042	0.64526965	0.821982874	-0.177
cg25436766	II	37	2	106792426	UXS1	0.5	0.00432	2.36443	0.46407515	0.287182094	0.177
cg26874367	II	37	2	129381792		0.46	0.01197	1.92207	0.18133147	0.357919523	-0.177
cg09681977	II	37	2	240153103	HDAC4	0.54	0.0014	2.85345	0.38720771	0.564006423	-0.177
cg03893701	II	37	3	139395765	NMNAT3	0.67	2.34E-05	4.63072	0.58354054	0.760253094	-0.177
cg05486924	II	37	3	194979565	C3orf21	0.5	0.00432	2.36443	0.39640291	0.573149999	-0.177
cg20969424	II	37	5	73690902		0.5	0.00432	2.36443	0.38774483	0.565230073	-0.177
cg02824029	II	37	6	29570008	GABBR1;GABBR1;G	0.5	0.00432	2.36443	0.55259169	0.729503297	-0.177
cg01646461	II	37	6	167536086	CCR6;CCR6	0.5	0.00432	2.36443	0.49244081	0.315046404	0.177
cg25326952	II	37	7	148720088	PDIA4	0.75	7.61E-07	6.11857	0.54602459	0.723404507	-0.177
cg02854313	II	37	8	19409023	CSGALNACT1;CSGA	0.5	0.00432	2.36443	0.59022632	0.767028701	-0.177
cg13126206	I	37	8	22930189		0.54	0.0014	2.85345	0.4829965	0.6604063	-0.177
cg13650740	II	37	10	80516517		0.67	2.34E-05	4.63072	0.36470581	0.541260932	-0.177
cg02921623	I	37	10	105250537		0.62	0.0001	3.98291	0.35176096	0.174686779	0.177
cg04719721	II	37	11	9787121		0.46	0.01197	1.92207	0.53126952	0.354314351	0.177
cg06526620	II	37	11	94278324	FUT4	0.58	0.00041	3.39184	0.49427874	0.670852528	-0.177
cg04537602	II	37	11	118763859	CXCR5;CXCR5	0.62	0.0001	3.98291	0.61901088	0.442441828	0.177
cg16303353	II	37	12	2393684	CACNA1C;CACNA1C	0.67	2.34E-05	4.63072	0.43967108	0.616379331	-0.177
cg21163444	II	37	12	54765670	ZNF385A;ZNF385A;	0.71	4.57E-06	5.34042	0.47274693	0.650163055	-0.177
cg19631815	I	37	12	113528791	DTX1	0.54	0.0014	2.85345	0.87901119	0.701656097	0.177
cg03646740	II	37	13	114813015	RASA3	0.5	0.00432	2.36443	0.67630793	0.49882646	0.177
cg02497428	II	37	16	21665138	METTL9;IGSF6;MET	0.5	0.00432	2.36443	0.65680142	0.833743872	-0.177
cg05597945	II	37	16	88059049	BANP;BANP	0.46	0.01197	1.92207	0.34953909	0.526066438	-0.177
cg20309353	II	37	17	18089940	ALKBH5	0.42	0.02991	1.52413	0.43356104	0.610195823	-0.177

cg04413853	II	37	17	58499706	C17orf64	0.58	0.00041	3.39184	0.63213585	0.809465262	-0.177
cg09187107	I	37	17	79006087	FLJ90757	0.5	0.00432	2.36443	0.64457336	0.821241106	-0.177
cg22898924	II	37	19	4903952	ARRDC5	0.62	0.0001	3.98291	0.40901602	0.586140929	-0.177
cg25605731	I	37	19	13054434	CALR	0.5	0.00432	2.36443	0.68913613	0.865697933	-0.177
cg11324504	I	37	22	38474147	SLC16A8	0.67	2.34E-05	4.63072	0.57262532	0.749676829	-0.177
cg19851816	I	37	22	50657907	TUBGCP6	0.46	0.01197	1.92207	0.33266443	0.509707675	-0.177
cg05119467	II	37	1	1765440	GNB1	0.54	0.0014	2.85345	0.69228858	0.516248017	0.176
cg20971147	II	37	1	14866944		0.58	0.00041	3.39184	0.53992959	0.363436437	0.176
cg26558485	I	37	1	47489282	CYP4X1;CYP4X1	0.62	0.0001	3.98291	0.23980712	0.416286025	-0.176
cg24461171	II	37	1	197745025	DENND1B;DENND1	0.42	0.02991	1.52413	0.38190985	0.557587347	-0.176
cg04287574	I	37	1	201619622	NAV1	0.42	0.02991	1.52413	0.44815171	0.272370835	0.176
cg17981790	II	37	2	16807234	FAM49A	0.5	0.00432	2.36443	0.49863315	0.322297798	0.176
cg17051239	II	37	2	27850192	CCDC121;CCDC121	0.5	0.00432	2.36443	0.4247797	0.600622475	-0.176
cg07015784	I	37	2	219257760	SLC11A1	0.5	0.00432	2.36443	0.73594075	0.559927536	0.176
cg15367000	II	37	2	239017149	ESPNL	0.42	0.02991	1.52413	0.68622408	0.510515614	0.176
cg16846518	II	37	3	128062608	EEFSEC	0.58	0.00041	3.39184	0.49932634	0.675477556	-0.176
cg06336230	II	37	3	197401572	MIR922;KIAA0226;I	0.46	0.01197	1.92207	0.69245447	0.51662867	0.176
cg18763629	II	37	4	1199037	LOC100130872;LO(	0.5	0.00432	2.36443	0.59799519	0.77441299	-0.176
cg22318806	II	37	6	31540411	LTA;LTA	0.58	0.00041	3.39184	0.41049529	0.234323055	0.176
cg07188523	I	37	6	44528793		0.54	0.0014	2.85345	0.64495041	0.821217402	-0.176
cg09377088	II	37	6	108910284	FOXO3;FOXO3	0.62	0.0001	3.98291	0.57311922	0.749152437	-0.176
cg06001419	II	37	7	4717725		0.58	0.00041	3.39184	0.57880708	0.754832924	-0.176
cg08930413	II	37	7	151548036	PRKAG2	0.71	4.57E-06	5.34042	0.55039252	0.726420281	-0.176
cg21440764	II	37	8	11755993		0.58	0.00041	3.39184	0.64798916	0.823783971	-0.176
cg26610247	I	37	8	142297175		0.46	0.01197	1.92207	0.52994931	0.706139541	-0.176
cg15721448	II	37	8	144239503	LY6H;LY6H;LY6H	0.67	2.34E-05	4.63072	0.37301803	0.548689365	-0.176
cg04190888	II	37	10	49879914		0.42	0.02991	1.52413	0.58968239	0.413932423	0.176
cg18591228	II	37	11	3175552	OSBPL5;OSBPL5;OS	0.54	0.0014	2.85345	0.50472641	0.680858045	-0.176
cg09761846	II	37	11	57117162	P2RX3	0.58	0.00041	3.39184	0.65879066	0.83476917	-0.176
cg12808596	II	37	11	60640645	ZP1	0.54	0.0014	2.85345	0.59910831	0.774997703	-0.176
cg27246571	II	37	12	96389588	HAL	0.54	0.0014	2.85345	0.71200521	0.887851037	-0.176
cg05946118	I	37	16	8985638		0.46	0.01197	1.92207	0.56042127	0.384353956	0.176
cg01502811	II	37	16	49869755		0.42	0.02991	1.52413	0.69233912	0.515882414	0.176

cg02226672	II	37	16	68398533	SMPD3	0.62	0.0001	3.98291	0.66950038	0.845343642	-0.176
cg01445100	II	37	16	88103339	BANP;BANP	0.54	0.0014	2.85345	0.47490077	0.29843429	0.176
cg26360197	II	37	17	78821604	RPTOR;RPTOR	0.5	0.00432	2.36443	0.59885451	0.422740208	0.176
cg13484467	II	37	17	81047784	METRNL	0.54	0.0014	2.85345	0.67220209	0.848495703	-0.176
cg24339704	II	37	19	2529022	GNG7	0.5	0.00432	2.36443	0.70156538	0.877538131	-0.176
cg05921699	II	37	19	42380725	CD79A;CD79A	0.54	0.0014	2.85345	0.74553654	0.56933829	0.176
cg08844849	II	37	19	51921812	SIGLEC10;SIGLEC10	0.67	2.34E-05	4.63072	0.56988352	0.394173795	0.176
cg03730622	II	37	22	46760678	CELSR1	0.5	0.00432	2.36443	0.48858638	0.312895653	0.176
cg03976856	II	37	1	2764669		0.5	0.00432	2.36443	0.6369889	0.461935379	0.175
cg05710479	II	37	1	7120104	CAMTA1	0.5	0.00432	2.36443	0.76366769	0.589111932	0.175
cg17952939	I	37	1	9154250		0.58	0.00041	3.39184	0.47681713	0.651875812	-0.175
cg10895547	II	37	1	78071255	ZZZ3	0.58	0.00041	3.39184	0.26995856	0.444722962	-0.175
cg25773695	II	37	1	116191657	VANGL1	0.54	0.0014	2.85345	0.63826983	0.463471751	0.175
cg22153407	II	37	1	230290089	GALNT2	0.5	0.00432	2.36443	0.67480322	0.849554353	-0.175
cg11256132	II	37	1	244515893	C1orf100	0.42	0.02991	1.52413	0.70961413	0.534364317	0.175
cg05654103	II	37	2	24586381		0.62	0.0001	3.98291	0.69544321	0.870140675	-0.175
cg02708956	II	37	2	240300118	HDAC4	0.46	0.01197	1.92207	0.6381172	0.813299938	-0.175
cg18030943	II	37	3	182876556	LAMP3	0.54	0.0014	2.85345	0.67866833	0.503627281	0.175
cg07241925	II	37	4	1294566	MAEA;MAEA	0.5	0.00432	2.36443	0.58469292	0.759954427	-0.175
cg06904356	I	37	5	1849983		0.54	0.0014	2.85345	0.55836155	0.732932412	-0.175
cg15712777	II	37	5	78204392	ARSB;ARSB	0.62	0.0001	3.98291	0.21418461	0.389198819	-0.175
cg13948857	II	37	5	131763756	C5orf56	0.5	0.00432	2.36443	0.30707442	0.481681249	-0.175
cg02855778	II	37	7	157462914	PTPRN2;PTPRN2;PT	0.58	0.00041	3.39184	0.42610684	0.250796058	0.175
cg03454775	I	37	8	2012595	MYOM2	0.58	0.00041	3.39184	0.54724559	0.722620256	-0.175
cg14109456	II	37	9	127040562	NEK6;NEK6;NEK6;N	0.42	0.02991	1.52413	0.41464504	0.589653378	-0.175
cg13992008	II	37	9	130739838	FAM102A	0.5	0.00432	2.36443	0.8241776	0.649420198	0.175
cg03062944	I	37	10	6183455		0.58	0.00041	3.39184	0.77256042	0.597482519	0.175
cg16788050	I	37	11	8053406		0.42	0.02991	1.52413	0.82426867	0.649126195	0.175
cg26215727	II	37	12	6485537	SCNN1A;SCNN1A;S	0.54	0.0014	2.85345	0.54106911	0.71588127	-0.175
cg12496975	II	37	12	48149863	RAPGEF3;RAPGEF3;	0.54	0.0014	2.85345	0.68926538	0.51458493	0.175
cg18818866	II	37	12	50221061	NCKAP5L;LOC1002	0.75	7.61E-07	6.11857	0.64672184	0.821663449	-0.175
cg00115639	II	37	17	54701692		0.42	0.02991	1.52413	0.59983401	0.774586457	-0.175
cg05774614	II	37	17	78864539	RPTOR;RPTOR	0.58	0.00041	3.39184	0.46048285	0.635878531	-0.175

cg01219924	II	37	17	79004947	FLJ90757	0.58	0.00041	3.39184	0.47079919	0.645672812	-0.175
cg09138892	I	37	17	79005662	FLJ90757	0.5	0.00432	2.36443	0.6986818	0.873397393	-0.175
cg18050634	I	37	17	79420145	BAHCC1	0.5	0.00432	2.36443	0.5332734	0.358141756	0.175
cg26221801	I	37	20	62183966	C20orf195	0.71	4.57E-06	5.34042	0.55036957	0.375011628	0.175
cg07507493	II	37	20	62574095	UCKL1;MIR1914;MI	0.46	0.01197	1.92207	0.48724418	0.662108785	-0.175
cg07247610	II	37	1	9097422	SLC2A5;SLC2A5	0.62	0.0001	3.98291	0.68424289	0.510169054	0.174
cg02927682	I	37	1	54844424	SSBP3;SSBP3;SSBP3	0.46	0.01197	1.92207	0.24427826	0.418637599	-0.174
cg17933936	II	37	1	226885709	ITPKB	0.58	0.00041	3.39184	0.71495318	0.541152754	0.174
cg17177006	II	37	3	5232410	EDEM1	0.58	0.00041	3.39184	0.45429902	0.280711827	0.174
cg07892374	II	37	3	130568122		0.62	0.0001	3.98291	0.26772232	0.441949484	-0.174
cg01919999	II	37	5	180668568	GNB2L1	0.58	0.00041	3.39184	0.36620051	0.53973312	-0.174
cg26964592	II	37	6	32904621	HLA-DMB	0.42	0.02991	1.52413	0.54642902	0.720517564	-0.174
cg03595161	II	37	7	1127767	GPER;C7orf50;GPEF	0.62	0.0001	3.98291	0.59641949	0.770463581	-0.174
cg13710553	II	37	7	129598625		0.54	0.0014	2.85345	0.3558742	0.529896934	-0.174
cg01012242	II	37	7	140043078	SLC37A3;SLC37A3	0.54	0.0014	2.85345	0.40349439	0.577799271	-0.174
cg10225865	I	37	8	54605566		0.42	0.02991	1.52413	0.289078	0.463560538	-0.174
cg25596082	II	37	10	43626561		0.67	2.34E-05	4.63072	0.24112709	0.414651224	-0.174
cg04902811	II	37	10	99478719	LOC100270710	0.54	0.0014	2.85345	0.30906216	0.482753808	-0.174
cg18390025	II	37	10	103986736	ELOVL3	0.58	0.00041	3.39184	0.46960588	0.643714015	-0.174
cg13104938	II	37	11	843956	TSPAN4;TSPAN4;TS	0.5	0.00432	2.36443	0.38557374	0.559592094	-0.174
cg01909245	II	37	11	1873757	LSP1	0.58	0.00041	3.39184	0.72118505	0.547216659	0.174
cg05463966	II	37	11	67803951	NDUFS8	0.54	0.0014	2.85345	0.3143688	0.488618497	-0.174
cg24313592	II	37	11	72020031	CLPB	0.46	0.01197	1.92207	0.71272186	0.538292626	0.174
cg07367607	II	37	12	131314725	STX2;STX2	0.5	0.00432	2.36443	0.37388525	0.199810863	0.174
cg15972148	I	37	13	114880889	RASA3	0.46	0.01197	1.92207	0.41439432	0.588092797	-0.174
cg22991506	II	37	14	50468241	C14orf182	0.67	2.34E-05	4.63072	0.48673868	0.312943394	0.174
cg09306188	I	37	16	4733253	MGRN1;MGRN1;MG	0.62	0.0001	3.98291	0.76498892	0.938695776	-0.174
cg09426937	II	37	16	70453185	ST3GAL2	0.46	0.01197	1.92207	0.57578898	0.401345974	0.174
cg24247482	II	37	17	42293627	UBTF;UBTF;UBTF	0.71	4.57E-06	5.34042	0.38325452	0.209270409	0.174
cg09121543	I	37	17	61774794	LIMD2	0.75	7.61E-07	6.11857	0.36997598	0.196347884	0.174
cg07855221	II	37	17	79877314	MAFG;MAFG;SIRT7	0.58	0.00041	3.39184	0.41772787	0.591989433	-0.174
cg23866916	II	37	19	1155738	SBNO2	0.62	0.0001	3.98291	0.60105799	0.774824494	-0.174
cg12002047	II	37	19	7767279	FCER2	0.46	0.01197	1.92207	0.4616318	0.287832968	0.174

cg24168413	I	37	19	35630388	FXYD1;FXYD1	0.54	0.0014	2.85345	0.47694642	0.651252575	-0.174
cg21966754	II	37	19	54584816	TARM1	0.46	0.01197	1.92207	0.17682024	0.350514934	-0.174
cg26331247	II	37	20	31239171	LOC284805	0.46	0.01197	1.92207	0.44427952	0.270735986	0.174
cg00546897	II	37	21	45232232	LOC284837	0.58	0.00041	3.39184	0.4653616	0.639123112	-0.174
cg06624244	II	37	1	16332335	C1orf64	0.54	0.0014	2.85345	0.50516758	0.332543023	0.173
cg26721382	II	37	1	31196596	MATN1	0.71	4.57E-06	5.34042	0.55338278	0.726765539	-0.173
cg24535918	II	37	1	52429399	RAB3B	0.46	0.01197	1.92207	0.42708791	0.25395343	0.173
cg17443007	II	37	1	101659419		0.54	0.0014	2.85345	0.64238807	0.814993885	-0.173
cg11193281	II	37	1	154547585	CHRNB2	0.5	0.00432	2.36443	0.47372479	0.30040686	0.173
cg23619936	II	37	2	866258		0.54	0.0014	2.85345	0.53748228	0.364710599	0.173
cg01331275	II	37	2	9908576		0.5	0.00432	2.36443	0.4874979	0.314707228	0.173
cg18008247	II	37	3	124719009	HEG1	0.62	0.0001	3.98291	0.5453523	0.718680297	-0.173
cg03446062	II	37	6	14477353		0.46	0.01197	1.92207	0.39165557	0.56479123	-0.173
cg05569328	II	37	6	32044384	TNXB	0.67	2.34E-05	4.63072	0.22568268	0.398396016	-0.173
cg09487078	II	37	6	167525398	CCR6;CCR6	0.54	0.0014	2.85345	0.46677876	0.293487407	0.173
cg21078077	I	37	7	997617		0.54	0.0014	2.85345	0.63945651	0.811985788	-0.173
cg01495332	II	37	7	50798564	GRB10;GRB10;GRB	0.54	0.0014	2.85345	0.6618562	0.488844965	0.173
cg04952324	II	37	7	73727108	CLIP2;CLIP2	0.58	0.00041	3.39184	0.46399512	0.637100301	-0.173
cg05862438	II	37	7	142920096	TAS2R40	0.5	0.00432	2.36443	0.26707436	0.440066787	-0.173
cg11969064	II	37	8	1845598	ARHGEF10	0.54	0.0014	2.85345	0.48905106	0.662245258	-0.173
cg16225800	II	37	8	126591906		0.46	0.01197	1.92207	0.56148854	0.388770755	0.173
cg13713821	II	37	9	95899302		0.5	0.00432	2.36443	0.70882845	0.5359552	0.173
cg07161179	II	37	11	698201	TMEM80;TMEM80	0.5	0.00432	2.36443	0.42387559	0.596619737	-0.173
cg12907644	II	37	11	18270341	SAA2;SAA2	0.62	0.0001	3.98291	0.56663076	0.739536854	-0.173
cg02647874	II	37	11	47380751	SPI1;SPI1	0.5	0.00432	2.36443	0.5424712	0.71531507	-0.173
cg11054816	II	37	11	117183853	BACE1;BACE1;BACE	0.46	0.01197	1.92207	0.64315912	0.816449006	-0.173
cg16766036	I	37	13	114800309	RASA3	0.54	0.0014	2.85345	0.75122373	0.924088031	-0.173
cg01811815	II	37	13	114881103	RASA3	0.5	0.00432	2.36443	0.4073875	0.580634861	-0.173
cg21507807	II	37	13	114888458	RASA3	0.54	0.0014	2.85345	0.50865794	0.681553586	-0.173
cg08190125	I	37	14	106145435		0.5	0.00432	2.36443	0.77119527	0.597713159	0.173
cg21898299	II	37	15	68924220	CORO2B	0.46	0.01197	1.92207	0.46467723	0.291561284	0.173
cg00709979	I	37	16	85866348		0.58	0.00041	3.39184	0.5577317	0.384551574	0.173
cg02835823	II	37	16	85979060		0.42	0.02991	1.52413	0.39660511	0.223538476	0.173

cg09816821	I	37	16	88709302		0.54	0.0014	2.85345	0.66132089	0.834775313	-0.173
cg02909097	II	37	17	2843206	RAP1GAP2;RAP1GA	0.58	0.00041	3.39184	0.62790505	0.801084062	-0.173
cg16530881	II	37	17	38708399		0.46	0.01197	1.92207	0.55344518	0.3800898	0.173
cg16643542	I	37	19	827843	AZU1;AZU1	0.54	0.0014	2.85345	0.56447839	0.737202483	-0.173
cg02849894	II	37	19	2608971	GNG7	0.54	0.0014	2.85345	0.71934416	0.546308726	0.173
cg13733403	II	37	19	6528291		0.5	0.00432	2.36443	0.77061493	0.598050688	0.173
cg17514528	II	37	1	11862907	MTHFR	0.5	0.00432	2.36443	0.37544106	0.547110767	-0.172
cg01823958	II	37	1	53557455	SLC1A7	0.54	0.0014	2.85345	0.46526629	0.637066465	-0.172
cg04360793	I	37	1	79472361	ELTD1;ELTD1	0.5	0.00432	2.36443	0.29607204	0.468332161	-0.172
cg26303281	II	37	1	153515622	S100A5	0.62	0.0001	3.98291	0.53565464	0.707488413	-0.172
cg11379081	I	37	1	209405050		0.54	0.0014	2.85345	0.25593296	0.428316324	-0.172
cg16256243	II	37	2	85648512		0.62	0.0001	3.98291	0.39486045	0.566683964	-0.172
cg03035167	II	37	2	201336269	SPATS2L;SPATS2L;S	0.54	0.0014	2.85345	0.63800453	0.465748235	0.172
cg01177100	II	37	3	9176211	SRGAP3;SRGAP3	0.54	0.0014	2.85345	0.51648688	0.687988006	-0.172
cg02352281	I	37	3	46139305		0.5	0.00432	2.36443	0.2846587	0.456841465	-0.172
cg22074114	II	37	3	134326341	KY	0.54	0.0014	2.85345	0.57042023	0.742079372	-0.172
cg11579059	II	37	4	156300245		0.58	0.00041	3.39184	0.68305234	0.854889479	-0.172
cg13026137	II	37	6	31701260	CLIC1	0.46	0.01197	1.92207	0.45529937	0.626841571	-0.172
cg24833027	II	37	8	1897969	ARHGEF10	0.46	0.01197	1.92207	0.59743432	0.769296814	-0.172
cg09529783	II	37	8	1958930		0.54	0.0014	2.85345	0.7254345	0.553121386	0.172
cg11639815	I	37	8	37379119		0.54	0.0014	2.85345	0.72217436	0.894456346	-0.172
cg07054804	II	37	11	10621492	MRVI1;MRVI1;MRV	0.62	0.0001	3.98291	0.67359492	0.845194298	-0.172
cg01219426	II	37	11	95856658	MAML2	0.62	0.0001	3.98291	0.44815071	0.275752843	0.172
cg10264003	II	37	11	110198965		0.5	0.00432	2.36443	0.36831608	0.196716813	0.172
cg13126638	II	37	12	6487080	SCNN1A	0.58	0.00041	3.39184	0.70473317	0.876640145	-0.172
cg14276580	II	37	13	40527020		0.62	0.0001	3.98291	0.37070876	0.198899962	0.172
cg05878073	II	37	14	74766220	ABCD4;ABCD4	0.46	0.01197	1.92207	0.53875413	0.710935617	-0.172
cg05313263	II	37	14	100906689	WDR25;WDR25	0.5	0.00432	2.36443	0.16642271	0.338712127	-0.172
cg04614155	II	37	15	42209202	EHD4	0.54	0.0014	2.85345	0.30348305	0.475970818	-0.172
cg01426968	II	37	16	15596369	C16orf45;C16orf45	0.62	0.0001	3.98291	0.63528105	0.807198328	-0.172
cg13629783	II	37	17	5689617		0.62	0.0001	3.98291	0.65037834	0.478493956	0.172
cg13195185	II	37	17	16875596	TNFRSF13B	0.5	0.00432	2.36443	0.50401966	0.332225994	0.172
cg25183263	II	37	17	25949090	KSR1	0.5	0.00432	2.36443	0.61373191	0.441721619	0.172

cg01636591	II	37	17	32646156	CCL8;CCL8	0.54	0.0014	2.85345	0.60053892	0.772464287	-0.172
cg03469804	II	37	17	72732432	RAB37;RAB37;RAB3	0.5	0.00432	2.36443	0.34804656	0.520209514	-0.172
cg08097359	II	37	19	50064054	NOSIP	0.54	0.0014	2.85345	0.32662536	0.498486345	-0.172
cg13650654	II	37	19	55435064	NLRP7;NLRP7;NLRP	0.67	2.34E-05	4.63072	0.60231834	0.774297642	-0.172
cg24334029	II	37	20	58296208	PHACTR3;PHACTR3	0.62	0.0001	3.98291	0.64259212	0.814383812	-0.172
cg10486610	I	37	20	61298677	LOC100127888;SLC	0.58	0.00041	3.39184	0.61245394	0.784554572	-0.172
cg02315187	II	37	21	46317913	ITGB2;ITGB2	0.67	2.34E-05	4.63072	0.44035348	0.612265925	-0.172
cg04011995	II	37	1	41326317		0.5	0.00432	2.36443	0.71071534	0.881413578	-0.171
cg25739016	II	37	1	167638918	RCSD1	0.46	0.01197	1.92207	0.63147515	0.460328655	0.171
cg20944315	II	37	1	200839460		0.46	0.01197	1.92207	0.67931051	0.508531357	0.171
cg18535415	II	37	1	200983238	KIF21B	0.5	0.00432	2.36443	0.59608931	0.767259529	-0.171
cg07434244	II	37	1	209847729	G0S2	0.58	0.00041	3.39184	0.3844029	0.555244674	-0.171
cg00809399	II	37	1	249153756	ZNF692;ZNF692	0.54	0.0014	2.85345	0.37432465	0.544905372	-0.171
cg15914335	II	37	2	1746253	PXDN	0.5	0.00432	2.36443	0.76127261	0.590231196	0.171
cg10329928	II	37	2	20414196	SDC1;SDC1	0.42	0.02991	1.52413	0.47991859	0.309229998	0.171
cg02458141	II	37	2	20817916	HS1BP3	0.46	0.01197	1.92207	0.31623307	0.487595768	-0.171
cg01994190	II	37	2	119768574		0.58	0.00041	3.39184	0.26940697	0.440634232	-0.171
cg24172324	II	37	2	232258363		0.54	0.0014	2.85345	0.32495409	0.153466951	0.171
cg10179004	I	37	3	48601902	COL7A1;UCN2	0.62	0.0001	3.98291	0.528529	0.699973447	-0.171
cg24121168	II	37	3	58652503	FAM3D;FAM3D	0.75	7.61E-07	6.11857	0.49405141	0.665342598	-0.171
cg23250593	II	37	3	196065688	TM4SF19	0.5	0.00432	2.36443	0.5284513	0.699659851	-0.171
cg09875213	II	37	5	134786596	TIFAB	0.54	0.0014	2.85345	0.69993767	0.528480358	0.171
cg10287970	II	37	6	7115345	RREB1;RREB1;RREB	0.5	0.00432	2.36443	0.41932804	0.590788267	-0.171
cg10975897	I	37	6	15504844	JARID2	0.5	0.00432	2.36443	0.65068065	0.821268875	-0.171
cg20052079	I	37	6	15504923	JARID2	0.46	0.01197	1.92207	0.68184665	0.852630596	-0.171
cg03739609	II	37	6	31555016	LST1;LST1;LST1;LST	0.54	0.0014	2.85345	0.37524687	0.545992286	-0.171
cg21588594	II	37	6	32917979	HLA-DMA	0.54	0.0014	2.85345	0.41094745	0.239818937	0.171
cg03078690	II	37	6	33235504	VPS52	0.71	4.57E-06	5.34042	0.59321788	0.764564585	-0.171
cg06702850	II	37	6	166876890	RPS6KA2;RPS6KA2	0.54	0.0014	2.85345	0.19921325	0.370637722	-0.171
cg26139131	II	37	7	72420870	NSUN5C;NSUN5C;N	0.54	0.0014	2.85345	0.58479179	0.755396483	-0.171
cg04996195	I	37	7	87105398	ABCB4;ABCB4;ABCE	0.5	0.00432	2.36443	0.44666873	0.275949673	0.171
cg17479131	II	37	7	149567078	LOC401431	0.67	2.34E-05	4.63072	0.67637584	0.505655531	0.171
cg02048922	I	37	8	143546858	BAI1	0.42	0.02991	1.52413	0.5464265	0.375441372	0.171

cg19215266	II	37	10	35417163	CREM;CREM;CREM	0.42	0.02991	1.52413	0.68598458	0.514763434	0.171
cg04084348	II	37	10	75677011	PLAU;C10orf55;PLA	0.5	0.00432	2.36443	0.29694553	0.467472529	-0.171
cg25306480	I	37	10	134226517	PWWP2B;PWWP2E	0.58	0.00041	3.39184	0.48245167	0.653627929	-0.171
cg20566897	II	37	11	313527	IFITM1	0.54	0.0014	2.85345	0.43112637	0.259633646	0.171
cg25976340	II	37	11	18611549	UEVLD;UEVLD	0.5	0.00432	2.36443	0.52588078	0.696548334	-0.171
cg15939287	II	37	11	64406051	NRXN2;NRXN2;NRX	0.5	0.00432	2.36443	0.66014791	0.489163382	0.171
cg09383860	II	37	11	71709837	IL18BP;IL18BP;IL18	0.42	0.02991	1.52413	0.24768459	0.418957904	-0.171
cg14596967	II	37	11	113956944	ZBTB16;ZBTB16	0.54	0.0014	2.85345	0.58593364	0.756554313	-0.171
cg19998289	II	37	12	121861616	RNF34;RNF34	0.71	4.57E-06	5.34042	0.68294668	0.853699253	-0.171
cg10082165	I	37	14	25043758	CTSG	0.58	0.00041	3.39184	0.64157624	0.813053769	-0.171
cg06341731	II	37	14	99641017	BCL11B;BCL11B	0.46	0.01197	1.92207	0.72772744	0.898375286	-0.171
cg02032125	II	37	14	101000186		0.58	0.00041	3.39184	0.57012882	0.740798021	-0.171
cg15022400	II	37	15	45028161	TRIM69;TRIM69	0.42	0.02991	1.52413	0.24475558	0.415669985	-0.171
cg19348484	II	37	15	91413236	FURIN	0.54	0.0014	2.85345	0.31977621	0.490797093	-0.171
cg02023973	II	37	16	1519973	CLCN7;CLCN7	0.54	0.0014	2.85345	0.45658344	0.627236105	-0.171
cg04856396	I	37	17	3589140	P2RX5;P2RX5	0.58	0.00041	3.39184	0.28429815	0.112799544	0.171
cg21554670	II	37	17	9967417	GAS7	0.5	0.00432	2.36443	0.45227407	0.622840823	-0.171
cg11690666	II	37	17	80415469	NARF;NARF;NARF;N	0.5	0.00432	2.36443	0.7344021	0.905544884	-0.171
cg06432200	I	37	18	77238375	NFATC1;NFATC1;NF	0.58	0.00041	3.39184	0.62329624	0.452434142	0.171
cg22035959	I	37	19	951681	ARID3A	0.46	0.01197	1.92207	0.73206139	0.561180257	0.171
cg12138514	II	37	19	14625610	DNAJB1	0.5	0.00432	2.36443	0.67935555	0.508606749	0.171
cg12530021	II	37	19	52004630	SIGLEC12;SIGLEC12	0.58	0.00041	3.39184	0.39260628	0.563595576	-0.171
cg12594933	II	37	1	3806817	C1orf174	0.46	0.01197	1.92207	0.40691071	0.577266107	-0.17
cg00502254	II	37	1	12201600	TNFRSF8;TNFRSF8	0.58	0.00041	3.39184	0.6752076	0.844947728	-0.17
cg17901584	II	37	1	55353706	DHCR24	0.5	0.00432	2.36443	0.56999	0.74001157	-0.17
cg02566391	II	37	1	67805528	IL12RB2	0.62	0.0001	3.98291	0.60840259	0.437970569	0.17
cg07234097	II	37	1	184761873	FAM129A	0.5	0.00432	2.36443	0.25158595	0.421679636	-0.17
cg14422093	II	37	1	227009245		0.54	0.0014	2.85345	0.65876557	0.489252343	0.17
cg08858998	II	37	1	229123641		0.54	0.0014	2.85345	0.57371536	0.744009715	-0.17
cg26750893	II	37	2	38043481		0.46	0.01197	1.92207	0.68553114	0.855230225	-0.17
cg24532083	II	37	3	44100380		0.58	0.00041	3.39184	0.41130602	0.241670168	0.17
cg26763394	II	37	3	45957664		0.54	0.0014	2.85345	0.68788448	0.517403354	0.17
cg22065514	II	37	3	48602040	COL7A1;UCN2	0.58	0.00041	3.39184	0.51249069	0.682770443	-0.17

cg26300517	II	37	4	38732661		0.67	2.34E-05	4.63072	0.59873961	0.42920154	0.17
cg14137381	I	37	5	502291	SLC9A3	0.54	0.0014	2.85345	0.36378932	0.193320883	0.17
cg01408486	II	37	5	139027024	CXXC5	0.5	0.00432	2.36443	0.21591124	0.386200504	-0.17
cg02343823	I	37	5	150284419	ZNF300	0.5	0.00432	2.36443	0.34640466	0.51640288	-0.17
cg08743794	II	37	6	30656577	KIAA1949;NRM;KIA	0.46	0.01197	1.92207	0.47641923	0.306303375	0.17
cg06632549	II	37	6	31529993		0.58	0.00041	3.39184	0.62310446	0.453114006	0.17
cg13775306	II	37	6	33577711		0.42	0.02991	1.52413	0.45845971	0.288708506	0.17
cg26918117	II	37	7	101603298	CUX1;CUX1;CUX1	0.54	0.0014	2.85345	0.72634969	0.896834098	-0.17
cg14774440	II	37	8	37730745	RAB11FIP1;RAB11F	0.5	0.00432	2.36443	0.41851813	0.588039986	-0.17
cg20611272	II	37	8	103548145		0.62	0.0001	3.98291	0.59325931	0.423367404	0.17
cg24752563	I	37	11	2410796	CD81	0.71	4.57E-06	5.34042	0.43995454	0.269969557	0.17
cg21746120	II	37	11	68142234	LRP5	0.46	0.01197	1.92207	0.41228779	0.581927349	-0.17
cg03139377	II	37	12	323242	SLC6A12;SLC6A12;S	0.5	0.00432	2.36443	0.39705044	0.567494546	-0.17
cg26269286	II	37	14	103848982		0.62	0.0001	3.98291	0.25946329	0.42944523	-0.17
cg09744966	II	37	15	31668476	KLF13	0.54	0.0014	2.85345	0.81736928	0.647207645	0.17
cg05799962	II	37	16	31404385	ITGAD	0.58	0.00041	3.39184	0.37233655	0.202822735	0.17
cg05083852	I	37	16	81480610	CMIP	0.42	0.02991	1.52413	0.40854885	0.578825883	-0.17
cg21949830	I	37	17	1510041	SLC43A2	0.5	0.00432	2.36443	0.77571168	0.606022696	0.17
cg05744184	II	37	17	40463806	STAT5A	0.62	0.0001	3.98291	0.34642204	0.516169377	-0.17
cg11872040	I	37	17	80819028	TBCD	0.5	0.00432	2.36443	0.79143221	0.621093952	0.17
cg22313519	II	37	19	18385724	KIAA1683;KIAA168	0.46	0.01197	1.92207	0.33371837	0.503818591	-0.17
cg12033822	II	37	20	44990591	SLC35C2;SLC35C2;S	0.54	0.0014	2.85345	0.44616896	0.616365344	-0.17
cg20519581	II	37	20	48959844		0.58	0.00041	3.39184	0.47412542	0.643757575	-0.17
cg19344545	II	37	21	45575573		0.62	0.0001	3.98291	0.63873401	0.468440658	0.17
cg10058766	I	37	1	12186079	TNFRSF8;TNFRSF8;	0.62	0.0001	3.98291	0.37451737	0.543079739	-0.169
cg23728867	II	37	1	12304452	VPS13D;VPS13D	0.54	0.0014	2.85345	0.56988058	0.739133054	-0.169
cg06774445	II	37	1	36793290		0.58	0.00041	3.39184	0.62594769	0.794853378	-0.169
cg13567986	I	37	1	38310056	MTF1	0.54	0.0014	2.85345	0.72225311	0.890781227	-0.169
cg09025625	II	37	1	154942509	SHC1;SHC1;SHC1;SH	0.46	0.01197	1.92207	0.34703313	0.516298735	-0.169
cg18882687	II	37	1	228275828	ARF1;ARF1;ARF1;AI	0.58	0.00041	3.39184	0.24840702	0.416994551	-0.169
cg25101396	II	37	2	27346296	ABHD1	0.5	0.00432	2.36443	0.31932774	0.488376365	-0.169
cg12454169	II	37	2	30669597	LCLAT1;LCLAT1	0.46	0.01197	1.92207	0.29648029	0.465219207	-0.169
cg05933789	II	37	2	97190408		0.62	0.0001	3.98291	0.83590838	0.667213843	0.169

cg09004241	II	37	2	232259431	B3GNT7	0.58	0.00041	3.39184	0.49342828	0.324080149	0.169
cg04438064	II	37	2	240161619	HDAC4	0.54	0.0014	2.85345	0.53406154	0.703434991	-0.169
cg26233331	II	37	4	6695614	S100P;S100P	0.5	0.00432	2.36443	0.42605902	0.59470034	-0.169
cg10925991	II	37	4	40324096		0.62	0.0001	3.98291	0.20517313	0.374215994	-0.169
cg22051146	II	37	5	177895047	COL23A1	0.46	0.01197	1.92207	0.26040614	0.429567987	-0.169
cg24111025	II	37	6	32819921	TAP1	0.58	0.00041	3.39184	0.87169509	0.702939993	0.169
cg10950593	II	37	7	6212775	CYTH3	0.58	0.00041	3.39184	0.64801286	0.817283836	-0.169
cg18325315	II	37	7	73897230	GTF2IRD1;GTF2IRD	0.5	0.00432	2.36443	0.66886874	0.838107789	-0.169
cg13471712	II	37	11	45952237	PHF21A;PHF21A	0.5	0.00432	2.36443	0.4992067	0.329942283	0.169
cg01550445	II	37	11	72929983	P2RY2;P2RY2;P2RY	0.58	0.00041	3.39184	0.62418147	0.793217949	-0.169
cg05754179	II	37	11	125985044		0.46	0.01197	1.92207	0.59472811	0.425899284	0.169
cg23807570	II	37	12	110787107	ATP2A2;ATP2A2;AT	0.58	0.00041	3.39184	0.60091542	0.769804409	-0.169
cg10024730	II	37	12	123402350		0.58	0.00041	3.39184	0.64454964	0.814005602	-0.169
cg15278374	I	37	12	131576088	GPR133	0.54	0.0014	2.85345	0.8174442	0.648587837	0.169
cg14643399	II	37	13	44706965		0.46	0.01197	1.92207	0.42288628	0.591779674	-0.169
cg19635644	II	37	13	100007277	UBAC2;MIR623;UB	0.5	0.00432	2.36443	0.81031301	0.641350136	0.169
cg24327132	II	37	15	72520632	PKM2;PKM2;PKM2	0.5	0.00432	2.36443	0.29385054	0.124529595	0.169
cg02102075	II	37	16	474430	RAB11FIP3	0.58	0.00041	3.39184	0.58403726	0.753420227	-0.169
cg02223351	II	37	16	66402367	CDH5	0.58	0.00041	3.39184	0.31578554	0.484537246	-0.169
cg02549595	II	37	16	85947219	IRF8	0.62	0.0001	3.98291	0.53447612	0.702981806	-0.169
cg04708264	II	37	16	87323420		0.58	0.00041	3.39184	0.68353656	0.852245722	-0.169
cg19776793	I	37	17	79225974	SLC38A10;SLC38A1	0.54	0.0014	2.85345	0.75288515	0.583497451	0.169
cg02224021	II	37	19	836716		0.58	0.00041	3.39184	0.32721752	0.496220106	-0.169
cg19867991	II	37	19	13051573	CALR	0.58	0.00041	3.39184	0.45758302	0.626280844	-0.169
cg18109874	II	37	19	14115611	RFX1	0.67	2.34E-05	4.63072	0.45416415	0.622664771	-0.169
cg02812809	I	37	19	39361447	RINL	0.62	0.0001	3.98291	0.72307854	0.892571726	-0.169
cg07747220	I	37	20	3052115	OXT	0.5	0.00432	2.36443	0.63974439	0.808564012	-0.169
cg00009196	II	37	20	19954588	RIN2	0.58	0.00041	3.39184	0.23617363	0.405386911	-0.169
cg00125455	II	37	20	44574271	PCIF1	0.62	0.0001	3.98291	0.62822418	0.797617558	-0.169
cg18731680	II	37	22	19953712	COMT;COMT;COM	0.58	0.00041	3.39184	0.43087437	0.600301708	-0.169
cg23098018	I	37	1	9775755	PIK3CD	0.42	0.02991	1.52413	0.89255448	0.724057401	0.168
cg21471199	II	37	1	19218212	ALDH4A1;ALDH4A1	0.54	0.0014	2.85345	0.67434844	0.842586688	-0.168
cg05202204	II	37	1	38460950		0.54	0.0014	2.85345	0.42869659	0.596485768	-0.168

cg00177203	II	37	1	149901142	MTMR11;MTMR11	0.58	0.00041	3.39184	0.73037104	0.898812726	-0.168
cg06764092	I	37	1	159796658	SLAMF8;SLAMF8	0.5	0.00432	2.36443	0.47112972	0.63919834	-0.168
cg02334333	II	37	2	60687392	BCL11A;BCL11A;BC	0.54	0.0014	2.85345	0.44106519	0.609034711	-0.168
cg26253134	II	37	2	70751721	TGFA;TGFA	0.54	0.0014	2.85345	0.33219924	0.500674834	-0.168
cg08202287	II	37	2	232259281	B3GNT7	0.46	0.01197	1.92207	0.4422367	0.274474225	0.168
cg15871206	I	37	2	239009118	ESPNL	0.62	0.0001	3.98291	0.67019239	0.838608809	-0.168
cg13419330	II	37	3	10247487	IRAK2	0.62	0.0001	3.98291	0.66930535	0.837148902	-0.168
cg00505318	II	37	3	69130918	UBA3;UBA3	0.54	0.0014	2.85345	0.50139894	0.333045769	0.168
cg21593835	II	37	3	123354036	MYLK;MYLK;MYLK;I	0.58	0.00041	3.39184	0.48013159	0.648177946	-0.168
cg21964798	I	37	5	1503259	LPCAT1	0.46	0.01197	1.92207	0.88473334	0.71651783	0.168
cg05187549	II	37	5	138904075		0.46	0.01197	1.92207	0.65992089	0.492042612	0.168
cg17038235	II	37	5	172370524	ERGIC1	0.5	0.00432	2.36443	0.63667962	0.804360932	-0.168
cg20477259	II	37	6	31544960	TNF	0.54	0.0014	2.85345	0.59809683	0.43048165	0.168
cg08784966	II	37	6	37248768	TBC1D22B	0.62	0.0001	3.98291	0.65443062	0.822000794	-0.168
cg04074908	II	37	6	44101190	TMEM63B	0.5	0.00432	2.36443	0.60093997	0.432679525	0.168
cg16640008	I	37	6	159515404		0.54	0.0014	2.85345	0.76323394	0.595074142	0.168
cg05076730	II	37	8	129060815	MIR1207;PVT1	0.5	0.00432	2.36443	0.32361538	0.491803507	-0.168
cg14283140	II	37	9	135763667	C9orf9	0.46	0.01197	1.92207	0.4812227	0.313481652	0.168
cg01709312	II	37	10	112630835	LOC282997;PDCD4	0.42	0.02991	1.52413	0.6346489	0.466615411	0.168
cg21130221	II	37	11	2848310	KCNQ1;KCNQ1	0.67	2.34E-05	4.63072	0.39696349	0.565376297	-0.168
cg16389345	II	37	11	46697382		0.54	0.0014	2.85345	0.6371993	0.804790583	-0.168
cg21599336	II	37	11	133910341	LOC100128239	0.46	0.01197	1.92207	0.41814101	0.586399974	-0.168
cg00159243	I	37	12	109023799	SELPLG	0.42	0.02991	1.52413	0.56853405	0.736815924	-0.168
cg05294112	II	37	13	112617069		0.46	0.01197	1.92207	0.39991009	0.568173764	-0.168
cg08162124	I	37	13	113428094	ATP11A;ATP11A	0.5	0.00432	2.36443	0.85486476	0.687158494	0.168
cg12798040	I	37	14	104171840	XRCC3;XRCC3;XRCC	0.58	0.00041	3.39184	0.74228976	0.910568359	-0.168
cg14372792	II	37	15	93188381	FAM174B	0.5	0.00432	2.36443	0.32507861	0.493395295	-0.168
cg17792616	II	37	15	93580327		0.5	0.00432	2.36443	0.65652417	0.824704094	-0.168
cg00599219	I	37	16	3307037	MEFV	0.54	0.0014	2.85345	0.55537526	0.723527046	-0.168
cg07967665	II	37	16	27542328	GTF3C1	0.54	0.0014	2.85345	0.53170339	0.699651204	-0.168
cg01298987	II	37	16	30005856	HIRIP3	0.58	0.00041	3.39184	0.42840907	0.596115906	-0.168
cg05246530	II	37	16	70441937	ST3GAL2	0.5	0.00432	2.36443	0.72982125	0.56223955	0.168
cg01900006	I	37	16	88814613	FAM38A	0.5	0.00432	2.36443	0.6486734	0.480640894	0.168

cg25673591	II	37	17	39945814		0.58	0.00041	3.39184	0.52927003	0.697217082	-0.168
cg14275281	II	37	17	43189622	PLCD3	0.46	0.01197	1.92207	0.48980415	0.321377474	0.168
cg13480197	II	37	17	79799358		0.62	0.0001	3.98291	0.54719448	0.71548448	-0.168
cg09907228	II	37	19	838964		0.58	0.00041	3.39184	0.66492119	0.83332953	-0.168
cg09134726	II	37	19	841082	PRTN3	0.67	2.34E-05	4.63072	0.44964832	0.61767638	-0.168
cg17680611	II	37	19	16191217	TPM4;TPM4	0.5	0.00432	2.36443	0.76066551	0.592858124	0.168
cg26623753	II	37	19	39224370	CAPN12	0.46	0.01197	1.92207	0.38992967	0.558143294	-0.168
cg11182965	II	37	22	19864308	TXNRD2	0.46	0.01197	1.92207	0.48657521	0.318711815	0.168
cg08465307	I	37	1	3827081	LOC100133612	0.5	0.00432	2.36443	0.78932194	0.622273451	0.167
cg10210690	II	37	1	55138059	C1orf175;C1orf175	0.46	0.01197	1.92207	0.64329498	0.476692657	0.167
cg06207961	II	37	1	108661230		0.62	0.0001	3.98291	0.21112325	0.378142983	-0.167
cg21422623	II	37	1	226912213	ITPKB	0.5	0.00432	2.36443	0.71837916	0.551514875	0.167
cg13043454	II	37	2	48980605	LHCGR	0.46	0.01197	1.92207	0.69221515	0.525216487	0.167
cg08003402	II	37	3	15311021	SH3BP5;SH3BP5	0.54	0.0014	2.85345	0.3522896	0.519434069	-0.167
cg20133901	II	37	3	51602121	RAD54L2	0.58	0.00041	3.39184	0.230017	0.396561347	-0.167
cg01290904	II	37	4	5708474	EVC2;EVC2	0.62	0.0001	3.98291	0.56512136	0.731903785	-0.167
cg10136560	II	37	4	140737518	MAML3	0.75	7.61E-07	6.11857	0.54575478	0.713254507	-0.167
cg08400424	II	37	5	142357042	ARHGAP26;ARHGAI	0.46	0.01197	1.92207	0.38051424	0.547981234	-0.167
cg17328407	II	37	5	157288521		0.54	0.0014	2.85345	0.75952757	0.592144579	0.167
cg07446753	II	37	7	2527391		0.46	0.01197	1.92207	0.59352242	0.426907468	0.167
cg21607965	II	37	7	2648289	IQCE;IQCE	0.58	0.00041	3.39184	0.25949431	0.426628245	-0.167
cg23304647	II	37	7	2778058	GNA12	0.5	0.00432	2.36443	0.23003721	0.396727686	-0.167
cg19809988	II	37	7	149569883	ATP6V0E2;ATP6V0E	0.58	0.00041	3.39184	0.33695039	0.504432253	-0.167
cg11937033	I	37	7	155150681		0.42	0.02991	1.52413	0.64346438	0.810703577	-0.167
cg08224773	II	37	8	11311868	FAM167A	0.5	0.00432	2.36443	0.60065319	0.434054313	0.167
cg02046475	II	37	8	28047708	ELP3	0.46	0.01197	1.92207	0.41526574	0.248483199	0.167
cg08243626	II	37	10	6442501		0.5	0.00432	2.36443	0.20412496	0.371400404	-0.167
cg24951886	II	37	10	13526236	BEND7;BEND7	0.54	0.0014	2.85345	0.69696003	0.529627881	0.167
cg26378073	II	37	10	105213818	CALHM1	0.5	0.00432	2.36443	0.25889523	0.425791399	-0.167
cg20833786	II	37	11	18141393	MRGPRX3	0.54	0.0014	2.85345	0.4786086	0.645461011	-0.167
cg23090046	II	37	14	104094619	KLC1;KLC1;KLC1	0.54	0.0014	2.85345	0.39712222	0.564186504	-0.167
cg27545630	II	37	16	2013058	SNORA64;SNORA1(	0.5	0.00432	2.36443	0.73911054	0.572416337	0.167
cg27050111	II	37	16	8738240	C16orf68	0.54	0.0014	2.85345	0.32497004	0.49148614	-0.167

cg27087781	II	37	16	30108404	YPEL3;YPEL3	0.5	0.00432	2.36443	0.34266536	0.510021595	-0.167
cg16460860	II	37	17	17744282		0.67	2.34E-05	4.63072	0.53111735	0.698611964	-0.167
cg17980404	II	37	17	38601676	IGFBP4	0.54	0.0014	2.85345	0.35242898	0.519824972	-0.167
cg20772590	II	37	17	75446431	SEPT9;SEPT9;SEPT9	0.54	0.0014	2.85345	0.55289825	0.385619301	0.167
cg16638092	I	37	17	78800774	RPTOR;RPTOR	0.42	0.02991	1.52413	0.31358666	0.480784795	-0.167
cg11609556	I	37	17	79006044	FLJ90757	0.58	0.00041	3.39184	0.60674826	0.773545524	-0.167
cg01558916	II	37	19	11804727		0.5	0.00432	2.36443	0.59947032	0.766777242	-0.167
cg04624110	I	37	20	13976093	MACROD2	0.79	1.06E-07	6.97389	0.1054464	0.272703697	-0.167
cg04087207	II	37	20	31669392	C20orf186	0.46	0.01197	1.92207	0.43486511	0.602148921	-0.167
cg02455383	II	37	22	27014116	CRYBB1	0.62	0.0001	3.98291	0.30760081	0.474381038	-0.167
cg14830045	I	37	1	1173264		0.42	0.02991	1.52413	0.56082069	0.395077599	0.166
cg00560747	II	37	1	31279227		0.54	0.0014	2.85345	0.44869588	0.282978176	0.166
cg09825327	II	37	1	211503219	TRAF5;TRAF5	0.67	2.34E-05	4.63072	0.63820764	0.472196538	0.166
cg01054402	II	37	2	85551850	TGOLN2	0.46	0.01197	1.92207	0.49683844	0.662791809	-0.166
cg23546106	II	37	2	216764063		0.5	0.00432	2.36443	0.54088427	0.70717783	-0.166
cg12425057	II	37	3	15107106	MRPS25	0.5	0.00432	2.36443	0.35295777	0.518606857	-0.166
cg14788242	II	37	3	127773954	SEC61A1	0.46	0.01197	1.92207	0.57165762	0.737505873	-0.166
cg05445326	II	37	3	196065569	TM4SF19	0.46	0.01197	1.92207	0.66080976	0.826587543	-0.166
cg13995774	II	37	5	179189810	MAML1	0.42	0.02991	1.52413	0.63476119	0.800549748	-0.166
cg14741870	II	37	6	30174091	TRIM26	0.5	0.00432	2.36443	0.70947743	0.543174156	0.166
cg13747899	II	37	6	33171911	HSD17B8;SLC39A7;	0.54	0.0014	2.85345	0.42548426	0.591555422	-0.166
cg09130674	II	37	6	39195019	KCNK5	0.58	0.00041	3.39184	0.3036196	0.470068162	-0.166
cg18905161	II	37	6	84742998	MRAP2	0.5	0.00432	2.36443	0.52991525	0.363682263	0.166
cg22340370	II	37	7	2019882	MAD1L1;MAD1L1;M	0.5	0.00432	2.36443	0.52620374	0.36028919	0.166
cg22679120	II	37	7	2353402	SNX8	0.5	0.00432	2.36443	0.48601764	0.320445989	0.166
cg25075684	II	37	7	2933297		0.46	0.01197	1.92207	0.28660026	0.120578381	0.166
cg15825027	II	37	8	586336		0.46	0.01197	1.92207	0.71167993	0.877490852	-0.166
cg08170427	II	37	8	144410815	TOP1MT	0.54	0.0014	2.85345	0.37085371	0.537002518	-0.166
cg13979562	II	37	9	138967844	NACC2	0.5	0.00432	2.36443	0.19550896	0.361679858	-0.166
cg04453371	II	37	10	30496461		0.46	0.01197	1.92207	0.61024853	0.443855974	0.166
cg06266973	II	37	10	43811183		0.67	2.34E-05	4.63072	0.47679532	0.642719542	-0.166
cg24517899	II	37	10	126290093	LHPP;LHPP	0.46	0.01197	1.92207	0.50997107	0.344276202	0.166
cg08623810	II	37	11	64427416	NRXN2;NRXN2	0.5	0.00432	2.36443	0.58022759	0.746328125	-0.166

cg18832738	II	37	12	95210053		0.67	2.34E-05	4.63072	0.67738639	0.511176592	0.166
cg25772418	II	37	12	131519998	GPR133	0.42	0.02991	1.52413	0.71917621	0.553114668	0.166
cg27026786	I	37	13	24798133	SPATA13;SPATA13	0.67	2.34E-05	4.63072	0.61314348	0.447200355	0.166
cg16505704	II	37	13	80378331		0.54	0.0014	2.85345	0.60982621	0.775958369	-0.166
cg24730612	II	37	14	75649973		0.58	0.00041	3.39184	0.70893441	0.874499459	-0.166
cg20981219	II	37	14	77385200		0.58	0.00041	3.39184	0.65219163	0.818174137	-0.166
cg11667061	II	37	14	101909021		0.67	2.34E-05	4.63072	0.42264222	0.256251376	0.166
cg05603784	I	37	16	88499083	ZNF469	0.5	0.00432	2.36443	0.69332805	0.85907324	-0.166
cg16802439	I	37	16	88907184	GALNS	0.5	0.00432	2.36443	0.75474624	0.920844765	-0.166
cg27615938	II	37	17	16344574	NCRNA00188;NCRN	0.58	0.00041	3.39184	0.45955082	0.625477758	-0.166
cg12188928	II	37	17	27309139	SEZ6;SEZ6	0.58	0.00041	3.39184	0.62616745	0.792652791	-0.166
cg06966839	II	37	17	55685941	MSI2;MSI2	0.5	0.00432	2.36443	0.62175975	0.788202446	-0.166
cg13545633	II	37	17	75524350		0.46	0.01197	1.92207	0.64560577	0.479385297	0.166
cg05377587	I	37	17	80845231	TBCD	0.58	0.00041	3.39184	0.73077564	0.564313689	0.166
cg19746982	II	37	18	77552568		0.46	0.01197	1.92207	0.45883613	0.625069389	-0.166
cg26580869	I	37	19	611506	HCN2	0.58	0.00041	3.39184	0.61462891	0.449069724	0.166
cg09479818	II	37	19	33880790	PEPD;PEPD;PEPD	0.54	0.0014	2.85345	0.67576623	0.841415833	-0.166
cg22801149	II	37	19	50472470	SIGLEC16	0.54	0.0014	2.85345	0.59736958	0.763139138	-0.166
cg11453079	II	37	20	43058872	HNF4A;HNF4A	0.54	0.0014	2.85345	0.64595898	0.47958693	0.166
cg01565160	II	37	20	61512901	DIDO1	0.67	2.34E-05	4.63072	0.67160511	0.837897422	-0.166
cg17050807	I	37	22	42548356		0.71	4.57E-06	5.34042	0.74443955	0.910053163	-0.166
cg21428954	I	37	22	46760505	CELSR1	0.46	0.01197	1.92207	0.82657468	0.660152138	0.166
cg05656688	I	37	1	25254088	RUNX3;RUNX3	0.62	0.0001	3.98291	0.70630145	0.541792966	0.165
cg16395432	I	37	1	26880807	MIR1976;RPS6KA1;	0.46	0.01197	1.92207	0.65420201	0.818907789	-0.165
cg24332710	II	37	1	46016173	AKR1A1;AKR1A1	0.67	2.34E-05	4.63072	0.38858409	0.553584594	-0.165
cg09472600	II	37	1	183537770	NCF2;NCF2	0.46	0.01197	1.92207	0.24360933	0.408354359	-0.165
cg23633330	II	37	1	207510679	CD55;CD55	0.62	0.0001	3.98291	0.29144055	0.126194451	0.165
cg21473786	II	37	2	44311918		0.62	0.0001	3.98291	0.65463522	0.819386878	-0.165
cg04579254	II	37	2	231090745	SP140;SP140	0.5	0.00432	2.36443	0.78226291	0.617669745	0.165
cg21199854	II	37	3	52724044	GNL3;GNL3;GNL3;S	0.42	0.02991	1.52413	0.47556349	0.640116578	-0.165
cg04571130	II	37	3	128967393	COPG	0.54	0.0014	2.85345	0.5286806	0.693703975	-0.165
cg03840289	II	37	4	2262318	MXD4	0.54	0.0014	2.85345	0.50417501	0.669500279	-0.165
cg05612654	I	37	6	2375895		0.42	0.02991	1.52413	0.6914211	0.856767655	-0.165

cg12586150	II	37	6	2840792	SERPINB1	0.62	0.0001	3.98291	0.29235841	0.456939244	-0.165
cg26969933	II	37	6	31629900	C6orf47;BAT4	0.42	0.02991	1.52413	0.50062884	0.665743691	-0.165
cg02571055	I	37	6	158896790	TULP4;TULP4	0.62	0.0001	3.98291	0.76384874	0.598707301	0.165
cg04593460	II	37	7	2045488	MAD1L1;MAD1L1;M	0.46	0.01197	1.92207	0.65773547	0.493200433	0.165
cg06889086	II	37	7	55638230	VOPP1	0.54	0.0014	2.85345	0.76670711	0.601943432	0.165
cg11774624	I	37	8	41480639	AGPAT6	0.42	0.02991	1.52413	0.7630736	0.598350309	0.165
cg15442737	I	37	8	141370229	TRAPPC9;TRAPPC9	0.46	0.01197	1.92207	0.28055578	0.445693373	-0.165
cg13408655	II	37	9	101820844	COL15A1	0.5	0.00432	2.36443	0.40991019	0.574677384	-0.165
cg13430755	II	37	9	110310795		0.54	0.0014	2.85345	0.41476966	0.579836008	-0.165
cg27479162	II	37	10	98450737	PIK3AP1	0.58	0.00041	3.39184	0.4061705	0.570727749	-0.165
cg13424029	I	37	10	101297508		0.71	4.57E-06	5.34042	0.37901787	0.544205968	-0.165
cg24680439	II	37	10	134778467		0.42	0.02991	1.52413	0.4650955	0.630052794	-0.165
cg19668951	II	37	11	47430812	SLC39A13;SLC39A1	0.5	0.00432	2.36443	0.39273337	0.557304752	-0.165
cg12070987	I	37	11	67804055	NDUFS8	0.58	0.00041	3.39184	0.55294488	0.718399859	-0.165
cg05154234	II	37	12	48204680	HDAC7;HDAC7	0.62	0.0001	3.98291	0.60790148	0.443156486	0.165
cg11881599	II	37	12	92814084	CLLU1OS;CLLU1;CLI	0.46	0.01197	1.92207	0.57557625	0.410122191	0.165
cg01878807	II	37	14	24422368	DHRS4;C14orf167;C	0.54	0.0014	2.85345	0.41390401	0.578779494	-0.165
cg07004075	II	37	14	24649335	REC8;REC8	0.5	0.00432	2.36443	0.57104411	0.405772387	0.165
cg13067714	II	37	14	69091453		0.67	2.34E-05	4.63072	0.54252741	0.707873789	-0.165
cg27563121	II	37	15	52405647	BCL2L10	0.62	0.0001	3.98291	0.67743369	0.842209413	-0.165
cg16103421	II	37	15	102068632		0.54	0.0014	2.85345	0.45887969	0.624162816	-0.165
cg27193519	II	37	16	4714443	MGRN1;MGRN1;M	0.5	0.00432	2.36443	0.35505213	0.520352184	-0.165
cg05035061	II	37	16	85028831	ZDHHC7;ZDHHC7	0.58	0.00041	3.39184	0.65726262	0.821905133	-0.165
cg04782146	II	37	16	85112370	KIAA0513	0.58	0.00041	3.39184	0.63337369	0.797991846	-0.165
cg00463732	II	37	17	3704621	ITGAE	0.54	0.0014	2.85345	0.57543525	0.740796665	-0.165
cg27372422	II	37	19	41086247	SHKBP1	0.46	0.01197	1.92207	0.32825447	0.492789199	-0.165
cg13226290	II	37	20	1448595	NSFL1C;NSFL1C;NSI	0.5	0.00432	2.36443	0.35970679	0.525198649	-0.165
cg22100563	I	37	20	62505188	TPD52L2;TPD52L2;T	0.54	0.00175	2.75713	0.79018582	0.955658837	-0.165
cg05523906	II	37	1	17751761	RCC2;RCC2	0.62	0.0001	3.98291	0.5599962	0.724467783	-0.164
cg00970057	II	37	1	42272694	HIVEP3;HIVEP3	0.5	0.00432	2.36443	0.59651142	0.432226465	0.164
cg20309640	II	37	1	178027432		0.62	0.0001	3.98291	0.40304159	0.239315322	0.164
cg00942920	II	37	1	203734559	LAX1;LAX1;LAX1	0.62	0.0001	3.98291	0.61135979	0.447573946	0.164
cg17517296	II	37	2	43107458		0.58	0.00041	3.39184	0.30288439	0.466465316	-0.164

cg26207766	II	37	2	242763794		0.5	0.00432	2.36443	0.52283118	0.686996914	-0.164
cg24462247	II	37	4	1770837		0.42	0.02991	1.52413	0.57510921	0.411029489	0.164
cg26076905	II	37	5	67522298	PIK3R1	0.46	0.01197	1.92207	0.66776406	0.8315535	-0.164
cg22685009	II	37	5	71597700	MRPS27	0.42	0.02991	1.52413	0.72618468	0.562440851	0.164
cg16176675	II	37	5	134786942	TIFAB	0.5	0.00432	2.36443	0.60942673	0.773460752	-0.164
cg17485265	II	37	6	3848790	FAM50B	0.62	0.0001	3.98291	0.70546741	0.869295434	-0.164
cg10145196	II	37	6	30647649	KIAA1949;KIAA194	0.62	0.0001	3.98291	0.66964575	0.505901791	0.164
cg17039645	II	37	6	32294503	C6orf10	0.58	0.00041	3.39184	0.61575628	0.779492705	-0.164
cg04904815	I	37	6	41010654	TSPO2;TSPO2	0.54	0.0014	2.85345	0.55383719	0.71802196	-0.164
cg01281718	II	37	6	71376634	SMAP1;SMAP1	0.67	2.34E-05	4.63072	0.73680234	0.901065449	-0.164
cg22059211	I	37	8	735312		0.54	0.0014	2.85345	0.79836401	0.634028679	0.164
cg22835851	II	37	10	49892302	WDFY4	0.67	2.34E-05	4.63072	0.67724055	0.513722596	0.164
cg10689689	II	37	11	63677197	MARK2;MARK2;MA	0.54	0.0014	2.85345	0.63491239	0.798646666	-0.164
cg26761618	II	37	11	72928926	P2RY2;P2RY2;P2RY	0.58	0.00041	3.39184	0.55583363	0.719438884	-0.164
cg10287137	I	37	11	72929054	P2RY2;P2RY2;P2RY	0.58	0.00041	3.39184	0.51175421	0.675679016	-0.164
cg07240557	II	37	12	27396937	STK38L	0.42	0.02991	1.52413	0.3069323	0.470890534	-0.164
cg08445469	II	37	12	53970794	ATF7;ATF7;ATF7	0.58	0.00041	3.39184	0.52912155	0.693531826	-0.164
cg23975840	II	37	12	117042895		0.58	0.00041	3.39184	0.70402985	0.867964259	-0.164
cg12701674	I	37	13	114908876		0.71	4.57E-06	5.34042	0.59081485	0.426614395	0.164
cg22242148	I	37	15	74215283		0.5	0.00432	2.36443	0.40977511	0.57399218	-0.164
cg12644285	II	37	15	93570953	CHD2	0.58	0.00041	3.39184	0.6591323	0.823024087	-0.164
cg06458258	II	37	16	1440604		0.58	0.00041	3.39184	0.75850241	0.59490027	0.164
cg05058976	I	37	16	3637956	BTBD12	0.67	2.34E-05	4.63072	0.71695874	0.552821412	0.164
cg04231085	I	37	16	85561302		0.54	0.0014	2.85345	0.72395312	0.887654171	-0.164
cg02834449	II	37	16	85786110	C16orf74	0.42	0.02991	1.52413	0.47701304	0.312570258	0.164
cg16603012	I	37	16	88879593	APRT;APRT	0.54	0.0014	2.85345	0.75265009	0.916885231	-0.164
cg05953373	II	37	16	89023633	CBFA2T3	0.67	2.34E-05	4.63072	0.60955816	0.773778521	-0.164
cg11204139	I	37	17	3907470		0.67	2.34E-05	4.63072	0.62844422	0.792652959	-0.164
cg18346531	II	37	17	9967431	GAS7	0.54	0.0014	2.85345	0.4660369	0.629624998	-0.164
cg18152830	II	37	17	16875129	TNFRSF13B	0.46	0.01197	1.92207	0.63889347	0.474987217	0.164
cg19439043	II	37	17	37719913		0.46	0.01197	1.92207	0.34739244	0.511263204	-0.164
cg06752482	II	37	17	58499816	C17orf64	0.54	0.0014	2.85345	0.44796949	0.611863955	-0.164
cg11297046	II	37	19	49657000	HRC	0.58	0.00041	3.39184	0.51901159	0.682924213	-0.164

cg13285174	I	37	20	3052221	OXT	0.58	0.00041	3.39184	0.60212514	0.766381743	-0.164
cg03082779	II	37	20	45947025	ZMYND8;LOC10013	0.58	0.00041	3.39184	0.38858296	0.553077378	-0.164
cg03364381	II	37	21	43099460	NCRNA00111	0.42	0.02991	1.52413	0.42927385	0.593040887	-0.164
cg09160123	II	37	1	41981735	HIVEP3;HIVEP3	0.5	0.00432	2.36443	0.44412404	0.607568132	-0.163
cg17479280	I	37	1	156466088	MEF2D	0.54	0.0014	2.85345	0.44060302	0.277516362	0.163
cg09122593	II	37	1	203020141	PPFIA4	0.5	0.00432	2.36443	0.3697115	0.532742846	-0.163
cg18113803	II	37	1	220977858	MOSC1	0.54	0.0014	2.85345	0.8125022	0.649941509	0.163
cg12057242	II	37	2	9603687	CPSF3	0.46	0.01197	1.92207	0.60942987	0.772882939	-0.163
cg16170614	II	37	4	24802387	SOD3	0.67	2.34E-05	4.63072	0.28110332	0.444558317	-0.163
cg26235369	II	37	5	173286145		0.5	0.00432	2.36443	0.35418155	0.517433597	-0.163
cg17840719	I	37	5	178368253	ZNF454;ZNF454	0.54	0.0014	2.85345	0.2522644	0.415659624	-0.163
cg18505752	II	37	6	32808752	PSMB8;PSMB8	0.67	2.34E-05	4.63072	0.56333687	0.400698341	0.163
cg13446584	II	37	7	74024953		0.5	0.00432	2.36443	0.66280839	0.826106871	-0.163
cg02913553	II	37	8	1951383	KBTBD11	0.54	0.0014	2.85345	0.53810698	0.700973743	-0.163
cg22234712	I	37	8	96085385		0.54	0.0014	2.85345	0.29681147	0.133616669	0.163
cg22644321	II	37	8	126446923	TRIB1	0.46	0.01197	1.92207	0.24559137	0.408739096	-0.163
cg07971089	II	37	8	144629702		0.5	0.00432	2.36443	0.21660977	0.379338716	-0.163
cg07629149	I	37	10	334731	DIP2C	0.54	0.0014	2.85345	0.82254768	0.659733307	0.163
cg24199006	II	37	10	106089203	ITPRIP	0.42	0.02991	1.52413	0.30195177	0.465307878	-0.163
cg07298473	I	37	11	47279183	NR1H3;NR1H3;NR1	0.58	0.00041	3.39184	0.32742354	0.490038068	-0.163
cg09010791	II	37	11	72448539	ARAP1	0.62	0.0001	3.98291	0.69058412	0.854033604	-0.163
cg18838431	I	37	11	74178800	KCNE3	0.58	0.00041	3.39184	0.30814625	0.470712267	-0.163
cg23474890	I	37	12	122467179	BCL7A;BCL7A	0.54	0.0014	2.85345	0.79678454	0.634266325	0.163
cg09294084	I	37	13	113646732	MCF2L;MCF2L	0.58	0.00041	3.39184	0.49408246	0.657261374	-0.163
cg16502747	II	37	14	23531625	ACIN1;ACIN1;ACIN1	0.46	0.01197	1.92207	0.67450909	0.837823247	-0.163
cg23689615	II	37	15	74228839	LOXL1	0.58	0.00041	3.39184	0.64001348	0.802519905	-0.163
cg16455376	II	37	16	8985720		0.58	0.00041	3.39184	0.63643999	0.473239224	0.163
cg05849013	II	37	16	12411139	SNX29	0.42	0.02991	1.52413	0.76860735	0.605145314	0.163
cg27583010	I	37	16	30198505	CORO1A;LOC60672	0.46	0.01197	1.92207	0.71338268	0.550759396	0.163
cg09019347	II	37	17	3493618	TRPV1;TRPV1;TRPV	0.42	0.02991	1.52413	0.59154248	0.428478154	0.163
cg21097733	II	37	17	46623729	HOXB2	0.67	2.34E-05	4.63072	0.60101031	0.764102651	-0.163
cg23939642	II	37	17	79225757	SLC38A10;SLC38A1	0.42	0.02991	1.52413	0.70215613	0.539319528	0.163
cg06663615	II	37	17	79423959	BAHCC1	0.46	0.01197	1.92207	0.45042649	0.613849356	-0.163

cg21786191	II	37	18	72916012	ZADH2	0.58	0.00041	3.39184	0.43912645	0.602259104	-0.163
cg10655021	I	37	19	21769430		0.62	0.0001	3.98291	0.06721019	0.230028363	-0.163
cg00748432	II	37	22	18042722	SLC25A18	0.5	0.00432	2.36443	0.46246016	0.62573206	-0.163
cg00791190	I	37	22	50223573		0.42	0.02991	1.52413	0.79047308	0.62749663	0.163
cg24578937	II	37	1	2090814	PRKCZ;PRKCZ;PRKC	0.46	0.01197	1.92207	0.38408058	0.546252549	-0.162
cg26028440	II	37	1	27982716		0.54	0.0014	2.85345	0.40888763	0.571262526	-0.162
cg09007354	II	37	1	54100163	GLIS1	0.5	0.00432	2.36443	0.56443284	0.72692798	-0.162
cg07029002	II	37	1	64358899	ROR1;ROR1	0.46	0.01197	1.92207	0.44179272	0.279306208	0.162
cg00563824	I	37	1	209405064		0.46	0.01197	1.92207	0.21057838	0.372494172	-0.162
cg14789818	I	37	1	227748712		0.42	0.02991	1.52413	0.40734326	0.569118719	-0.162
cg08231648	II	37	1	235011814		0.54	0.0014	2.85345	0.32513108	0.486837286	-0.162
cg18638581	II	37	2	75059602	HK2	0.54	0.0014	2.85345	0.33226764	0.494674578	-0.162
cg21340821	II	37	2	134855457		0.62	0.0001	3.98291	0.69848532	0.86022274	-0.162
cg19145607	II	37	3	45983792	CXCR6;FYCO1	0.54	0.0014	2.85345	0.64162813	0.803457347	-0.162
cg06628679	II	37	4	10609880	CLNK	0.5	0.00432	2.36443	0.64073926	0.802821796	-0.162
cg07814567	I	37	4	100736658	DAPP1	0.58	0.00041	3.39184	0.75123911	0.913346479	-0.162
cg08277306	I	37	5	598817		0.5	0.00432	2.36443	0.72229509	0.560642637	0.162
cg14606478	II	37	6	30174190	TRIM26	0.5	0.00432	2.36443	0.68223718	0.519842692	0.162
cg13336690	II	37	6	32976484	HLA-DOA	0.46	0.01197	1.92207	0.70537035	0.543400307	0.162
cg09287933	II	37	6	33384473	CUTA;CUTA;CUTA;(	0.58	0.00041	3.39184	0.79088001	0.629329108	0.162
cg25444339	II	37	7	75194698	HIP1	0.54	0.0014	2.85345	0.43864768	0.600702319	-0.162
cg15549637	II	37	7	105489998	ATXN7L1;ATXN7L1	0.54	0.0014	2.85345	0.72143892	0.559304336	0.162
cg20849109	II	37	8	1954777	KBTBD11	0.54	0.0014	2.85345	0.35461944	0.516590657	-0.162
cg23314364	I	37	8	29230998		0.46	0.01197	1.92207	0.27118566	0.432947729	-0.162
cg23731089	I	37	8	141599208	EIF2C2;EIF2C2	0.5	0.00432	2.36443	0.82612821	0.66453008	0.162
cg07168939	II	37	8	143763412	PSCA	0.62	0.0001	3.98291	0.44620202	0.608455239	-0.162
cg14459032	II	37	9	78905335		0.5	0.00432	2.36443	0.56393242	0.726206804	-0.162
cg23758703	II	37	10	114610508	LOC143188	0.58	0.00041	3.39184	0.59358878	0.431165481	0.162
cg13428066	II	37	11	2677768	KCNQ1;KCNQ1OT1;	0.58	0.00041	3.39184	0.26699602	0.428827208	-0.162
cg06708720	II	37	12	1099075	ERC1;ERC1;ERC1;EF	0.67	2.34E-05	4.63072	0.46013981	0.622288845	-0.162
cg15920906	II	37	12	111619414	CUX2	0.54	0.0014	2.85345	0.43137159	0.59343289	-0.162
cg21830849	II	37	12	129283486	SLC15A4	0.46	0.01197	1.92207	0.85555502	0.693223824	0.162
cg24018814	II	37	12	133348116	GOLGA3	0.46	0.01197	1.92207	0.38861873	0.22630547	0.162

cg25939371	II	37	15	72598286	BRUNOL6	0.58	0.00041	3.39184	0.59900707	0.761148636	-0.162
cg00268476	II	37	16	2740589	KCTD5	0.62	0.0001	3.98291	0.36727974	0.529418327	-0.162
cg03890691	I	37	16	30023615	DOC2A	0.5	0.00432	2.36443	0.52908568	0.367564091	0.162
cg05573626	II	37	16	49672687	ZNF423	0.54	0.0014	2.85345	0.36187604	0.524044619	-0.162
cg08580187	II	37	16	84027871	NECAB2	0.5	0.00432	2.36443	0.27781101	0.44010903	-0.162
cg14467066	II	37	17	79419739	BAHCC1	0.46	0.01197	1.92207	0.62145333	0.458987887	0.162
cg22092521	II	37	19	859330	CFD	0.5	0.00432	2.36443	0.23677818	0.398972642	-0.162
cg21495704	I	37	19	36399346	TYROBP;TYROBP	0.46	0.01197	1.92207	0.4352857	0.597755454	-0.162
cg08228249	II	37	22	22062300	YPEL1	0.5	0.00432	2.36443	0.78029701	0.618519583	0.162
cg10097977	II	37	22	33670007	LARGE;LARGE	0.46	0.01197	1.92207	0.71881439	0.557254785	0.162
cg14686297	II	37	22	46650375		0.54	0.0014	2.85345	0.34631771	0.507827058	-0.162
cg15822328	II	37	1	1072197		0.5	0.00432	2.36443	0.43188465	0.270809266	0.161
cg00546248	II	37	1	2412743	PLCH2	0.42	0.02991	1.52413	0.59280449	0.432095904	0.161
cg15015996	I	37	1	3382565	ARHGEF16	0.54	0.0014	2.85345	0.53642281	0.374950285	0.161
cg03403093	II	37	2	99132041	INPP4A;INPP4A;INF	0.58	0.00041	3.39184	0.73650182	0.897273439	-0.161
cg24695950	I	37	2	162364264		0.46	0.01197	1.92207	0.44829241	0.287675082	0.161
cg25941354	II	37	2	218989983	CXCR2;CXCR2	0.58	0.00041	3.39184	0.40021273	0.561564944	-0.161
cg01368900	II	37	3	13113959	IQSEC1	0.42	0.02991	1.52413	0.46982726	0.30920061	0.161
cg07672051	II	37	5	149887787	NDST1;NDST1	0.58	0.00041	3.39184	0.59999095	0.761122492	-0.161
cg03857664	II	37	5	171864134	SH3PXD2B	0.58	0.00041	3.39184	0.84680131	0.685431885	0.161
cg26813604	II	37	5	172256357		0.58	0.00041	3.39184	0.73112112	0.570389261	0.161
cg14989988	II	37	7	105319819	ATXN7L1;ATXN7L1	0.5	0.00432	2.36443	0.39880223	0.55962737	-0.161
cg13264840	II	37	7	142494213		0.58	0.00041	3.39184	0.64945594	0.488397165	0.161
cg09912552	II	37	8	134250937	NDRG1;NDRG1	0.58	0.00041	3.39184	0.66968328	0.83073575	-0.161
cg14827056	I	37	8	141550539	EIF2C2;EIF2C2	0.5	0.00432	2.36443	0.73412552	0.895361227	-0.161
cg14789659	II	37	10	63809073	ARID5B	0.54	0.0014	2.85345	0.83007614	0.668738834	0.161
cg19286437	I	37	10	88428117	LDB3;LDB3;LDB3;LD	0.62	0.0001	3.98291	0.6521834	0.491450664	0.161
cg20728490	II	37	10	98064175	DNTT;DNTT;DNTT;D	0.67	2.34E-05	4.63072	0.33778658	0.176760323	0.161
cg10133725	II	37	11	64981081	SLC22A20	0.46	0.01197	1.92207	0.18695455	0.348181784	-0.161
cg23261640	I	37	11	70280489	CTTN;CTTN	0.5	0.00432	2.36443	0.74867262	0.587524147	0.161
cg18023065	I	37	11	94278603	FUT4	0.5	0.00432	2.36443	0.60563724	0.766932574	-0.161
cg25574603	II	37	12	13256766	GSG1;GSG1	0.54	0.0014	2.85345	0.62555999	0.464797589	0.161
cg22032626	II	37	12	50203811	NCKAP5L	0.58	0.00041	3.39184	0.42251768	0.583239322	-0.161

cg00951869	II	37	14	24805349	ADCY4;RIPK3	0.58	0.00041	3.39184	0.29642137	0.457063483	-0.161
cg02268192	II	37	14	92981666	RIN3	0.46	0.01197	1.92207	0.39676667	0.557335359	-0.161
cg27456487	I	37	17	56349062	MPO	0.62	0.0001	3.98291	0.77102391	0.931987713	-0.161
cg14648237	II	37	17	64422393	PRKCA	0.46	0.01197	1.92207	0.4888245	0.650281808	-0.161
cg24723883	II	37	19	2608495	GNG7	0.67	2.34E-05	4.63072	0.6471518	0.485889936	0.161
cg27573549	I	37	19	56189952	EPN1;EPN1;EPN1	0.46	0.01197	1.92207	0.74126112	0.579845562	0.161
cg26472802	I	37	21	45713719	AIRE;AIRE	0.58	0.00041	3.39184	0.89884212	0.73831629	0.161
cg01394461	II	37	1	887576	NOC2L	0.58	0.00041	3.39184	0.60599922	0.765577211	-0.16
cg04211179	II	37	1	16301562	ZBTB17	0.54	0.0014	2.85345	0.49340644	0.33317474	0.16
cg13977835	II	37	1	23883338		0.62	0.0001	3.98291	0.75820803	0.598251392	0.16
cg01966665	II	37	1	151137376	SCNM1;LYSMD1;LY	0.62	0.0001	3.98291	0.6667738	0.827006663	-0.16
cg15392364	II	37	1	206976689	IL19	0.58	0.00041	3.39184	0.84106401	0.68074774	0.16
cg25743622	II	37	1	223403446	SUSD4	0.71	4.57E-06	5.34042	0.73680217	0.576523531	0.16
cg23014549	I	37	1	245727587	KIF26B	0.5	0.00432	2.36443	0.79840335	0.638371628	0.16
cg11938718	II	37	2	10526453	HPCAL1;HPCAL1	0.54	0.0014	2.85345	0.41210022	0.572130784	-0.16
cg03438773	II	37	2	219974186	NHEJ1	0.5	0.00432	2.36443	0.28129447	0.441008269	-0.16
cg18471664	II	37	3	5028143		0.58	0.00041	3.39184	0.25307725	0.41354996	-0.16
cg08033130	II	37	3	45983597	CXCR6;FYCO1	0.46	0.01197	1.92207	0.54938867	0.709853051	-0.16
cg04083553	II	37	3	128778575	GP9	0.54	0.0014	2.85345	0.58980258	0.429979766	0.16
cg07181209	II	37	5	153585979	GALNT10	0.5	0.00432	2.36443	0.6736531	0.83370027	-0.16
cg13027183	I	37	6	15504872	JARID2	0.46	0.01197	1.92207	0.60401983	0.764478268	-0.16
cg19913563	II	37	6	30720261		0.62	0.0001	3.98291	0.20485015	0.36482002	-0.16
cg26033526	II	37	6	32819858	TAP1	0.42	0.02991	1.52413	0.62647485	0.466065005	0.16
cg26338202	II	37	6	163768326		0.54	0.0014	2.85345	0.70634139	0.86625511	-0.16
cg04342202	II	37	7	1961968	MAD1L1;MAD1L1;N	0.5	0.00432	2.36443	0.43602285	0.595980146	-0.16
cg25817701	I	37	7	2140143	MAD1L1;MAD1L1;N	0.58	0.00041	3.39184	0.36596437	0.206186141	0.16
cg03676485	II	37	7	2563816	LFNG;LFNG;LFNG;LI	0.46	0.01197	1.92207	0.5283059	0.688674625	-0.16
cg02052217	II	37	7	99970869	PILRA;PILRA;PILRA	0.62	0.0001	3.98291	0.64887374	0.809238336	-0.16
cg07243202	II	37	8	134315520		0.67	2.34E-05	4.63072	0.35493581	0.515095962	-0.16
cg14333394	II	37	9	139812357	TRAF2	0.58	0.00041	3.39184	0.59588628	0.756310744	-0.16
cg09491962	II	37	10	98900771	SLIT1	0.58	0.00041	3.39184	0.59361412	0.753787841	-0.16
cg26983198	II	37	11	1952756	TNNT3;TNNT3;TNN	0.5	0.00432	2.36443	0.18510176	0.345073293	-0.16
cg24305156	II	37	11	45871936	CRY2;CRY2	0.5	0.00432	2.36443	0.53299854	0.69290031	-0.16

cg09374353	II	37	11	64620859	EHD1	0.54	0.0014	2.85345	0.34361857	0.183433921	0.16
cg11886358	II	37	11	128561007	FLI1	0.58	0.00041	3.39184	0.67689704	0.837240381	-0.16
cg10665892	II	37	12	1921115	CACNA2D4	0.54	0.0014	2.85345	0.60500539	0.765225715	-0.16
cg12018809	II	37	12	6745171	LPAR5;LPAR5	0.54	0.0014	2.85345	0.28180228	0.121759045	0.16
cg00476608	II	37	12	7044787	ATN1;ATN1	0.5	0.00432	2.36443	0.72314282	0.882799437	-0.16
cg08059719	II	37	12	122444450		0.67	2.34E-05	4.63072	0.57778476	0.41731725	0.16
cg02869235	II	37	12	124726864		0.5	0.00432	2.36443	0.63664046	0.796807729	-0.16
cg18960218	II	37	14	23285365	SLC7A7;SLC7A7;SLC	0.5	0.00432	2.36443	0.64685829	0.806569745	-0.16
cg17002328	II	37	14	91751773	CCDC88C	0.5	0.00432	2.36443	0.60697965	0.446602745	0.16
cg21220247	II	37	14	106321936		0.5	0.00432	2.36443	0.55156878	0.391244837	0.16
cg09924669	II	37	16	1593214	IFT140;TMEM204	0.58	0.00041	3.39184	0.37451915	0.534183288	-0.16
cg07451762	II	37	16	28383216		0.46	0.01197	1.92207	0.29587105	0.455772117	-0.16
cg10183965	II	37	17	3824497		0.54	0.0014	2.85345	0.40775094	0.247672339	0.16
cg03366884	II	37	17	8844506	PIK3R5	0.54	0.0014	2.85345	0.56197381	0.401674521	0.16
cg01502320	II	37	17	16875558	TNFRSF13B	0.54	0.0014	2.85345	0.53820688	0.378316492	0.16
cg13053608	II	37	17	40345673	GHDC;GHDC;GHDC;	0.54	0.0014	2.85345	0.34287587	0.502679236	-0.16
cg16338321	II	37	17	48994958		0.58	0.00041	3.39184	0.52530853	0.365666245	0.16
cg27200257	II	37	17	79816678	P4HB	0.5	0.00432	2.36443	0.6833988	0.843050217	-0.16
cg23141183	II	37	17	80185318	SLC16A3	0.54	0.0014	2.85345	0.68857126	0.848296964	-0.16
cg24112097	II	37	22	50196862	BRD1	0.42	0.02991	1.52413	0.56471446	0.404666834	0.16
cg06219660	II	37	1	1695509	NADK	0.5	0.00432	2.36443	0.59482864	0.754281113	-0.159
cg10250177	II	37	1	32739752	LCK;LCK;LCK	0.58	0.00041	3.39184	0.30240108	0.143533271	0.159
cg17171539	II	37	1	59398690		0.42	0.02991	1.52413	0.34916415	0.508070915	-0.159
cg08654091	II	37	1	227015418		0.54	0.0014	2.85345	0.5102768	0.66976688	-0.159
cg00050692	II	37	2	25524877	DNMT3A;DNMT3A;	0.54	0.0014	2.85345	0.2544575	0.41368887	-0.159
cg11800635	I	37	2	74783088	DOK1	0.58	0.00041	3.39184	0.63201873	0.79057556	-0.159
cg00514486	II	37	3	49837348	CDH29	0.5	0.00432	2.36443	0.56562341	0.724538708	-0.159
cg03921696	I	37	3	156848137		0.54	0.0014	2.85345	0.69284972	0.5343297	0.159
cg26668042	II	37	5	1514077	LPCAT1	0.5	0.00432	2.36443	0.47994774	0.639432702	-0.159
cg17071868	II	37	6	33047056	HLA-DPB1	0.46	0.01197	1.92207	0.69705309	0.537640786	0.159
cg04494800	II	37	6	149775853	ZC3H12D	0.58	0.00041	3.39184	0.39949491	0.558207384	-0.159
cg19730982	I	37	7	1062729	C7orf50;MIR339;C7	0.46	0.01197	1.92207	0.56497673	0.40589283	0.159
cg11884933	II	37	7	2774414	GNA12	0.46	0.01197	1.92207	0.19430026	0.353472494	-0.159

cg03817667	I	37	7	54609953	VSTM2A	0.5	0.00432	2.36443	0.1089011	0.268158104	-0.159
cg12743031	II	37	8	27219512	PTK2B;PTK2B;PTK2	0.58	0.00041	3.39184	0.58173732	0.422850538	0.159
cg26542567	II	37	8	142986670		0.46	0.01197	1.92207	0.66481246	0.505624115	0.159
cg14186336	II	37	9	124045139	GSN;GSN;GSN;GSN;	0.58	0.00041	3.39184	0.31249509	0.471717841	-0.159
cg05514971	II	37	9	130329588	FAM129B;FAM129I	0.62	0.0001	3.98291	0.36291517	0.521722952	-0.159
cg22697239	I	37	11	44626708	CD82;CD82	0.5	0.00432	2.36443	0.67586934	0.516772839	0.159
cg13408712	II	37	11	129817591	PRDM10;PRDM10;I	0.54	0.0014	2.85345	0.47684175	0.318135337	0.159
cg21892295	II	37	12	121157589	UNC119B	0.5	0.00432	2.36443	0.31662704	0.475860073	-0.159
cg15355713	II	37	12	125431744	DHX37	0.5	0.00432	2.36443	0.50725286	0.66622522	-0.159
cg19662895	II	37	14	69074455		0.5	0.00432	2.36443	0.33652577	0.495412012	-0.159
cg06142108	II	37	16	19195208	SYT17	0.58	0.00041	3.39184	0.73885818	0.897597194	-0.159
cg08851837	II	37	16	57558820	CCDC102A	0.58	0.00041	3.39184	0.67553146	0.834539481	-0.159
cg21808406	II	37	17	7482475	CD68;CD68	0.5	0.00432	2.36443	0.33727813	0.496342743	-0.159
cg02057796	II	37	17	74530987	CYGB	0.42	0.02991	1.52413	0.60393504	0.444463414	0.159
cg07126783	I	37	17	78800767	RPTOR;RPTOR	0.5	0.00432	2.36443	0.29061932	0.449387226	-0.159
cg23948811	I	37	17	79485043		0.54	0.0014	2.85345	0.69945281	0.540907051	0.159
cg00775818	II	37	19	2431171	LMNB2	0.5	0.00432	2.36443	0.5729887	0.7317012	-0.159
cg02987482	II	37	19	6274208	MLLT1	0.5	0.00432	2.36443	0.31882278	0.477590396	-0.159
cg00637477	II	37	20	62525797	DNAJC5	0.5	0.00432	2.36443	0.17213719	0.330840951	-0.159
cg26861460	II	37	22	44575455	PARVG;PARVG;PAR	0.46	0.01197	1.92207	0.61472928	0.774138608	-0.159
cg00209037	II	37	22	46443395		0.54	0.0014	2.85345	0.50961947	0.668898122	-0.159
cg01229567	II	37	1	1549818	MIB2;MIB2;MIB2;M	0.5	0.00432	2.36443	0.33754046	0.495501466	-0.158
cg10001720	II	37	1	31231363	LAPTM5	0.58	0.00041	3.39184	0.59984054	0.757381138	-0.158
cg04482628	II	37	1	36581751		0.58	0.00041	3.39184	0.43359019	0.591566283	-0.158
cg16723381	II	37	2	127590178		0.5	0.00432	2.36443	0.28229302	0.44005644	-0.158
cg05861291	II	37	2	207987538	KLF7	0.46	0.01197	1.92207	0.6504403	0.492120783	0.158
cg05583921	I	37	3	42700678	ZBTB47	0.54	0.0014	2.85345	0.67890211	0.836802846	-0.158
cg02241759	II	37	3	119528300	NR1I2;NR1I2;NR1I2	0.54	0.0014	2.85345	0.27562694	0.43357912	-0.158
cg08763102	II	37	4	3079751	HTT	0.5	0.00432	2.36443	0.35868165	0.200886794	0.158
cg15834072	I	37	4	155411603	DCHS2;DCHS2	0.42	0.02991	1.52413	0.11941131	0.277421044	-0.158
cg16248329	I	37	4	187644739	FAT1	0.5	0.00432	2.36443	0.06115178	0.219581776	-0.158
cg26249135	II	37	6	32026147	TNXB	0.42	0.02991	1.52413	0.72824569	0.570269743	0.158
cg24147543	II	37	6	32554481	HLA-DRB1	0.46	0.01197	1.92207	0.28187458	0.124209785	0.158

cg22904406	II	37	6	33288296	DAXX;DAXX;DAXX;D	0.58	0.00041	3.39184	0.69143179	0.849071566	-0.158
cg14407179	I	37	7	963768	ADAP1	0.54	0.0014	2.85345	0.79894947	0.640562107	0.158
cg17974145	II	37	7	101671556	CUX1;CUX1;CUX1	0.46	0.01197	1.92207	0.62448771	0.782132405	-0.158
cg19882784	II	37	7	134856562	C7orf49;C7orf49;C7	0.5	0.00432	2.36443	0.51727049	0.675651954	-0.158
cg02490460	II	37	8	1365502		0.46	0.01197	1.92207	0.61772506	0.775300778	-0.158
cg10327067	II	37	8	37549202		0.58	0.00041	3.39184	0.25551954	0.413597323	-0.158
cg23677272	II	37	8	70748063	SLCO5A1	0.58	0.00041	3.39184	0.40677956	0.564806093	-0.158
cg20133200	I	37	10	3141887	PFKP	0.58	0.00041	3.39184	0.64681737	0.804581232	-0.158
cg16704703	II	37	10	3509710		0.54	0.0014	2.85345	0.54657427	0.704503865	-0.158
cg11199437	II	37	10	39023045		0.5	0.00432	2.36443	0.32747997	0.48510546	-0.158
cg15009090	II	37	11	64163340		0.54	0.0014	2.85345	0.2809411	0.439076398	-0.158
cg11973132	II	37	11	67052656	ADRBK1	0.71	4.57E-06	5.34042	0.59563986	0.75384651	-0.158
cg25429672	II	37	11	73692155	UCP2	0.54	0.0014	2.85345	0.70999423	0.551986243	0.158
cg19100884	I	37	13	114825082	RASA3	0.46	0.01197	1.92207	0.68594322	0.84376163	-0.158
cg10975863	II	37	14	68830704	RAD51L1;RAD51L1;	0.46	0.01197	1.92207	0.57400932	0.731756868	-0.158
cg22908922	II	37	14	105855008	PACS2;PACS2	0.62	0.0001	3.98291	0.57840418	0.73593439	-0.158
cg23731826	II	37	15	90371692		0.5	0.00432	2.36443	0.63246204	0.790917595	-0.158
cg16649560	II	37	16	27338391	IL4R;IL4R	0.54	0.0014	2.85345	0.29174403	0.449629129	-0.158
cg17074014	I	37	17	3704494	ITGAE;ITGAE	0.5	0.00432	2.36443	0.71492902	0.872493229	-0.158
cg10115918	I	37	17	80922807	B3GNTL1	0.58	0.00041	3.39184	0.64854536	0.490649288	0.158
cg24165638	II	37	19	831456	AZU1	0.5	0.00432	2.36443	0.64092355	0.798886568	-0.158
cg15770046	II	37	19	35764710	USF2;USF2	0.58	0.00041	3.39184	0.59183082	0.749443293	-0.158
cg26540515	II	37	20	896349	ANGPT4	0.62	0.0001	3.98291	0.61965378	0.777578005	-0.158
cg22335872	II	37	20	896876	ANGPT4;ANGPT4	0.54	0.0014	2.85345	0.66341853	0.505033023	0.158
cg26576937	II	37	20	2739938	EBF4	0.46	0.01197	1.92207	0.57955732	0.42165897	0.158
cg01377358	II	37	20	30948211	ASXL1;ASXL1	0.42	0.02991	1.52413	0.64291084	0.484884381	0.158
cg07211972	II	37	21	44473496	CBS	0.58	0.00041	3.39184	0.73285152	0.890850246	-0.158
cg24349631	I	37	22	46449752	C22orf26;LOC15038	0.42	0.02991	1.52413	0.12550351	0.283317745	-0.158
cg20018425	II	37	22	50614877	PANX2;PANX2;PAN	0.42	0.02991	1.52413	0.45546188	0.29699249	0.158
cg24534743	II	37	1	27884345	AHDC1	0.5	0.00432	2.36443	0.39630415	0.553437708	-0.157
cg11257300	II	37	1	113229103	MOV10;MOV10	0.46	0.01197	1.92207	0.73265818	0.575196591	0.157
cg08247583	I	37	1	120927113	FCGR1B;FCGR1B	0.54	0.0014	2.85345	0.55506194	0.712512102	-0.157
cg19907725	II	37	1	153539397	S100A2	0.62	0.0001	3.98291	0.57093976	0.728143959	-0.157

cg21567649	II	37	2	43245642		0.71	4.57E-06	5.34042	0.37523615	0.532424367	-0.157
cg12082609	II	37	2	66671727	MEIS1	0.54	0.0014	2.85345	0.23609859	0.392642864	-0.157
cg17161250	I	37	2	106682209	C2orf40;C2orf40	0.54	0.0014	2.85345	0.03736286	0.193882742	-0.157
cg21865845	II	37	3	79815848	ROBO1	0.54	0.0014	2.85345	0.31553019	0.472866783	-0.157
cg19982684	II	37	3	128403035		0.54	0.0014	2.85345	0.42044728	0.577384811	-0.157
cg04761746	II	37	4	141177756	SCOC	0.58	0.00041	3.39184	0.49911798	0.655743319	-0.157
cg17542330	II	37	5	79445875	SERINC5	0.58	0.00041	3.39184	0.54861115	0.705238222	-0.157
cg18899797	II	37	5	139120060		0.5	0.00432	2.36443	0.48776991	0.33083505	0.157
cg23327334	II	37	5	176855304	GRK6;GRK6;GRK6	0.58	0.00041	3.39184	0.27551542	0.432129904	-0.157
cg23740758	I	37	6	11324433	NEDD9	0.54	0.0014	2.85345	0.67844432	0.835102112	-0.157
cg23666117	II	37	6	31629909	C6orf47;BAT4	0.5	0.00432	2.36443	0.43792485	0.595185107	-0.157
cg18336453	II	37	6	43082296	PTK7;PTK7;PTK7;PT	0.58	0.00041	3.39184	0.41507803	0.571777795	-0.157
cg10089657	II	37	7	1062820	C7orf50;MIR339;C7	0.46	0.01197	1.92207	0.45650059	0.299438944	0.157
cg19972822	II	37	7	138778744	ZC3HAV1;ZC3HAV1	0.62	0.0001	3.98291	0.5976113	0.440620346	0.157
cg13439189	II	37	9	93926553		0.62	0.0001	3.98291	0.58717437	0.430370883	0.157
cg04813697	I	37	10	22920025	PIP4K2A	0.46	0.01197	1.92207	0.62006349	0.462665005	0.157
cg24505713	II	37	11	10770035		0.5	0.00432	2.36443	0.18950052	0.346354414	-0.157
cg17125472	I	37	11	69259831		0.54	0.0014	2.85345	0.61011325	0.767515717	-0.157
cg25570864	II	37	13	112838390		0.62	0.0001	3.98291	0.61289814	0.769852531	-0.157
cg00105306	II	37	15	85194978	WDR73	0.5	0.00432	2.36443	0.69514481	0.851801174	-0.157
cg09408143	II	37	16	524778	RAB11FIP3;RAB11F	0.46	0.01197	1.92207	0.58732454	0.744322685	-0.157
cg06767314	II	37	17	1085381	ABR	0.42	0.02991	1.52413	0.77684792	0.619737731	0.157
cg13203480	II	37	17	16875349	TNFRSF13B	0.46	0.01197	1.92207	0.7327053	0.576142152	0.157
cg26628751	II	37	17	80981644	B3GNTL1	0.5	0.00432	2.36443	0.61997253	0.777200607	-0.157
cg15224459	II	37	17	81042023	METRNL	0.54	0.0014	2.85345	0.32998463	0.487167682	-0.157
cg14663914	II	37	19	827739	AZU1	0.54	0.0014	2.85345	0.37342461	0.530234767	-0.157
cg05257528	II	37	19	846179	PRTN3	0.67	2.34E-05	4.63072	0.58858247	0.745752205	-0.157
cg14781778	II	37	22	45608692	C22orf9;C22orf9	0.62	0.0001	3.98291	0.74516639	0.901918881	-0.157
cg10177197	II	37	1	55316481	DHCR24	0.62	0.0001	3.98291	0.71223883	0.868465297	-0.156
cg20335425	I	37	1	153363264	S100A8	0.46	0.01197	1.92207	0.53534422	0.690937433	-0.156
cg10503610	II	37	1	154833121	KCNN3;KCNN3	0.42	0.02991	1.52413	0.42239575	0.266726448	0.156
cg19272720	II	37	1	204289444	PLEKHA6	0.58	0.00041	3.39184	0.77224219	0.616136714	0.156
cg02641801	II	37	2	26213508		0.67	2.34E-05	4.63072	0.38418337	0.539846756	-0.156

cg15135286	II	37	2	33359281	LTBP1;LTBP1;LTBP1	0.46	0.01197	1.92207	0.69039163	0.846577381	-0.156
cg09133511	II	37	2	113384907		0.62	0.0001	3.98291	0.27973329	0.43546822	-0.156
cg06541968	II	37	2	240431144		0.62	0.0001	3.98291	0.59752948	0.75381237	-0.156
cg24277586	II	37	3	52099561	C3orf74	0.62	0.0001	3.98291	0.40862207	0.564173945	-0.156
cg06164260	II	37	3	187454439	BCL6;BCL6	0.62	0.0001	3.98291	0.32220991	0.477823008	-0.156
cg11697194	I	37	5	964196		0.5	0.00432	2.36443	0.60321028	0.446710925	0.156
cg23384708	II	37	6	31544934	TNF	0.58	0.00041	3.39184	0.59878265	0.442607799	0.156
cg21769820	II	37	7	625136	PRKAR1B;PRKAR1B	0.54	0.0014	2.85345	0.74357912	0.587772297	0.156
cg21511321	II	37	7	1004997	COX19	0.67	2.34E-05	4.63072	0.34942774	0.505242245	-0.156
cg02827175	II	37	7	1986245	MAD1L1;MAD1L1;M	0.58	0.00041	3.39184	0.70522397	0.861492849	-0.156
cg04222728	II	37	7	138549665	KIAA1549;KIAA154	0.62	0.0001	3.98291	0.72093441	0.876941879	-0.156
cg01049678	II	37	7	150147536	GIMAP8	0.54	0.0014	2.85345	0.29427669	0.450231554	-0.156
cg04058799	II	37	7	151547969	PRKAG2	0.58	0.00041	3.39184	0.53813004	0.694470952	-0.156
cg11201447	II	37	8	128808063	MIR1204;PVT1	0.46	0.01197	1.92207	0.46157622	0.305737936	0.156
cg00918944	II	37	9	35908117	LOC158376	0.58	0.00041	3.39184	0.23925541	0.395586659	-0.156
cg06083652	II	37	10	1531529	ADARB2	0.5	0.00432	2.36443	0.50347958	0.347480954	0.156
cg25124276	I	37	10	25464008	LOC100128811;GPI	0.54	0.0014	2.85345	0.09735061	0.25378448	-0.156
cg20671781	I	37	10	43626471		0.58	0.00041	3.39184	0.76876858	0.925106171	-0.156
cg20276630	I	37	10	97055439		0.71	4.57E-06	5.34042	0.35512477	0.19910397	0.156
cg16783349	I	37	10	130854343		0.5	0.00432	2.36443	0.83016629	0.674621541	0.156
cg25522867	II	37	11	34236648	ABTB2	0.54	0.0014	2.85345	0.72778614	0.571744291	0.156
cg23746050	II	37	11	69063559	MYEOV	0.5	0.00432	2.36443	0.52039407	0.363918042	0.156
cg17150898	I	37	12	1702116	FBXL14	0.67	2.34E-05	4.63072	0.65087124	0.495037453	0.156
cg17208953	II	37	12	108054842		0.5	0.00432	2.36443	0.71866726	0.874657707	-0.156
cg08289839	II	37	13	111318640	CARS2	0.5	0.00432	2.36443	0.60565293	0.761258012	-0.156
cg07296256	I	37	13	113438010	ATP11A;ATP11A	0.67	2.34E-05	4.63072	0.25939266	0.415187653	-0.156
cg00158530	II	37	16	69966973	MIR140;WWP2;WV	0.46	0.01197	1.92207	0.68072221	0.83651765	-0.156
cg20551922	II	37	17	4391282	SPNS3	0.75	7.61E-07	6.11857	0.66176941	0.817958076	-0.156
cg22433759	II	37	17	71824272	C17orf54	0.54	0.0014	2.85345	0.59421156	0.74993134	-0.156
cg08012219	II	37	17	76913036	TIMP2	0.46	0.01197	1.92207	0.5649486	0.720587632	-0.156
cg02569115	II	37	17	76922138	TIMP2	0.62	0.0001	3.98291	0.43622437	0.5918901	-0.156
cg10194602	II	37	17	78038139	CCDC40	0.5	0.00432	2.36443	0.44110581	0.597381661	-0.156
cg25256099	II	37	17	79484960		0.42	0.02991	1.52413	0.34432734	0.187996074	0.156

cg07083818	II	37	19	18385672	KIAA1683;KIAA168[illegible]	0.58	0.00041	3.39184	0.6689819	0.824690889	-0.156
cg13496662	I	37	19	45416369		0.54	0.0014	2.85345	0.37494363	0.531191442	-0.156
cg14792008	II	37	19	49607581	SNRNP70	0.46	0.01197	1.92207	0.61727107	0.461133632	0.156
cg26267561	II	37	20	3052224	OXT	0.58	0.00041	3.39184	0.49253241	0.648907612	-0.156
cg05056497	II	37	21	35899448	RCAN1;RCAN1;RCA	0.5	0.00432	2.36443	0.64304565	0.486849856	0.156
cg04096368	II	37	21	43988782	SLC37A1	0.42	0.02991	1.52413	0.63787029	0.481885848	0.156
cg06430509	II	37	21	44853470		0.54	0.0014	2.85345	0.73704182	0.581205236	0.156
cg05992904	I	37	22	48892994	FAM19A5	0.42	0.02991	1.52413	0.69420727	0.849756382	-0.156
cg25940513	II	37	1	9326282	H6PD	0.62	0.0001	3.98291	0.52719547	0.682479355	-0.155
cg12547807	II	37	1	9473751		0.58	0.00041	3.39184	0.29345788	0.138363641	0.155
cg22799499	II	37	1	40773045	COL9A2	0.5	0.00432	2.36443	0.60608622	0.760716317	-0.155
cg02988730	II	37	1	110167509	AMPD2;AMPD2;AN	0.42	0.02991	1.52413	0.6036991	0.449045528	0.155
cg27159979	II	37	2	60763331	BCL11A;BCL11A;BC	0.5	0.00432	2.36443	0.66825878	0.513063953	0.155
cg07220903	II	37	3	46973284	CCDC12	0.54	0.0014	2.85345	0.24669046	0.401550476	-0.155
cg14092988	I	37	3	52407081	DNAH1	0.54	0.0014	2.85345	0.7308521	0.886087234	-0.155
cg01016119	II	37	3	56835282	ARHGEF3;ARHGEF3	0.58	0.00041	3.39184	0.50336485	0.348545044	0.155
cg02855045	II	37	3	72777245		0.46	0.01197	1.92207	0.44714197	0.602385529	-0.155
cg07333191	II	37	4	13526769		0.54	0.0014	2.85345	0.40402321	0.558870842	-0.155
cg17582074	II	37	4	83189641		0.62	0.0001	3.98291	0.22575235	0.380747222	-0.155
cg08354216	II	37	4	146951349		0.67	2.34E-05	4.63072	0.44869691	0.293717101	0.155
cg10967114	II	37	5	150442668	TNIP1	0.58	0.00041	3.39184	0.76588699	0.920439483	-0.155
cg01999566	II	37	6	30624449	DHX16;DHX16	0.62	0.0001	3.98291	0.28112787	0.436283476	-0.155
cg13423389	I	37	6	30656508	KIAA1949;NRM;KIA	0.54	0.0014	2.85345	0.5146237	0.35992928	0.155
cg08469255	I	37	6	30851069	DDR1;DDR1	0.58	0.00041	3.39184	0.58560374	0.430776483	0.155
cg10365886	I	37	6	32063874	TNXB	0.54	0.0014	2.85345	0.6191432	0.773848483	-0.155
cg02382532	II	37	6	42110867	C6orf132	0.58	0.00041	3.39184	0.57562045	0.730336512	-0.155
cg03081478	II	37	7	2773127	GNA12	0.54	0.0014	2.85345	0.16302061	0.318294027	-0.155
cg16248756	II	37	7	127795594		0.54	0.0014	2.85345	0.23938477	0.39390791	-0.155
cg14023009	I	37	8	1870798	ARHGEF10	0.5	0.00432	2.36443	0.25787691	0.413157895	-0.155
cg06049177	II	37	8	30583924	GSR	0.54	0.0014	2.85345	0.66197476	0.816528928	-0.155
cg03481855	II	37	8	129089293	PVT1	0.5	0.00432	2.36443	0.74191672	0.586547932	0.155
cg17088014	II	37	8	134066590	SLA;TG;SLA;SLA	0.5	0.00432	2.36443	0.59586306	0.750901945	-0.155
cg00152340	I	37	8	144297019	GPIHBP1	0.58	0.00041	3.39184	0.8276953	0.672655825	0.155

cg21170085	I	37	9	137645730	COL5A1	0.46	0.01197	1.92207	0.78279934	0.627910761	0.155
cg03171770	I	37	10	43393728		0.54	0.0014	2.85345	0.19012388	0.345345945	-0.155
cg04894537	II	37	11	2763171	KCNQ1;KCNQ1	0.5	0.00432	2.36443	0.31558539	0.471009605	-0.155
cg20518446	I	37	11	62315034	AHNAK;AHNAK	0.5	0.00432	2.36443	0.61227693	0.767499626	-0.155
cg16024530	II	37	11	128673534	FLI1;FLI1	0.54	0.0014	2.85345	0.3282557	0.482999403	-0.155
cg10623198	II	37	12	6949114	GNB3	0.62	0.0001	3.98291	0.61947601	0.464880579	0.155
cg04473902	II	37	12	14721288	PLBD1	0.58	0.00041	3.39184	0.57730297	0.732057429	-0.155
cg03468072	II	37	12	39539422		0.67	2.34E-05	4.63072	0.34903051	0.504497074	-0.155
cg04920032	II	37	12	50262986	FAIM2	0.54	0.0014	2.85345	0.5168583	0.671807627	-0.155
cg08875297	II	37	12	56127068		0.54	0.0014	2.85345	0.55716029	0.402516339	0.155
cg18795232	I	37	12	121890163	KDM2B;KDM2B	0.58	0.00041	3.39184	0.52736112	0.68200053	-0.155
cg20628942	II	37	13	114171501	TMCO3	0.46	0.01197	1.92207	0.68899112	0.84370021	-0.155
cg04928930	II	37	14	20926227	TMEM55B;TMEM5!	0.5	0.00432	2.36443	0.65684624	0.811351141	-0.155
cg24231804	II	37	15	67316861		0.67	2.34E-05	4.63072	0.22817839	0.382768204	-0.155
cg13079123	I	37	15	67331913		0.54	0.0014	2.85345	0.30542898	0.460489577	-0.155
cg12349571	I	37	15	70364359	TLE3;TLE3;TLE3	0.5	0.00432	2.36443	0.8145799	0.659366872	0.155
cg00657780	II	37	16	50285254		0.58	0.00041	3.39184	0.70666414	0.861750042	-0.155
cg18177814	II	37	16	50730881	NOD2	0.62	0.0001	3.98291	0.37950649	0.534400153	-0.155
cg05757530	II	37	16	57038916	NLRC5	0.62	0.0001	3.98291	0.68752303	0.53263386	0.155
cg03987842	II	37	16	68321858	SLC7A6;SLC7A6	0.5	0.00432	2.36443	0.71756473	0.562625115	0.155
cg04324917	II	37	16	87734877	LOC100129637	0.42	0.02991	1.52413	0.57119604	0.416544876	0.155
cg09437994	I	37	16	89035147	CBFA2T3	0.58	0.00041	3.39184	0.74395168	0.588720049	0.155
cg13984928	I	37	17	3704574	ITGAE	0.54	0.0014	2.85345	0.79426148	0.948923149	-0.155
cg26284483	II	37	17	30184997	C17orf79	0.58	0.00041	3.39184	0.52807704	0.683277984	-0.155
cg11804928	II	37	17	38220298	THRA;THRA	0.62	0.0001	3.98291	0.35345782	0.507981415	-0.155
cg12812662	II	37	17	41857603	DUSP3;C17orf105	0.75	7.61E-07	6.11857	0.25083779	0.405339356	-0.155
cg04014328	I	37	17	46653615	HOXB4	0.58	0.00041	3.39184	0.63391956	0.788631149	-0.155
cg07728250	II	37	17	55655754	MSI2;MSI2	0.58	0.00041	3.39184	0.62356837	0.7782653	-0.155
cg01698392	II	37	17	76871734	TIMP2	0.62	0.0001	3.98291	0.44219129	0.596973828	-0.155
cg09253473	II	37	17	79877389	MAFG;MAFG;SIRT7	0.67	2.34E-05	4.63072	0.55331449	0.708358209	-0.155
cg25298189	II	37	19	935259	ARID3A	0.54	0.0014	2.85345	0.40030825	0.555392096	-0.155
cg11355029	I	37	19	6276660	MLLT1	0.58	0.00041	3.39184	0.61475795	0.76970347	-0.155
cg19644670	II	37	19	17185523	MYO9B;MYO9B;HA	0.46	0.01197	1.92207	0.30499574	0.149622368	0.155

cg13554818	II	37	19	43979316	PHLDB3	0.46	0.01197	1.92207	0.26230922	0.417022823	-0.155
cg14562523	II	37	21	43528734	C21orf128;UMODL	0.46	0.01197	1.92207	0.44671179	0.291944504	0.155
cg12222277	II	37	1	2191658	SKI	0.5	0.00432	2.36443	0.62421629	0.778027282	-0.154
cg09322534	II	37	1	43427192		0.5	0.00432	2.36443	0.7112733	0.557394123	0.154
cg07426848	II	37	1	153521712	S100A3;S100A3	0.5	0.00432	2.36443	0.60201248	0.448461815	0.154
cg03511957	II	37	1	167765211		0.62	0.0001	3.98291	0.6044567	0.758171955	-0.154
cg05455036	II	37	1	202828149		0.58	0.00041	3.39184	0.28991382	0.444333436	-0.154
cg10574566	II	37	1	203377808		0.46	0.01197	1.92207	0.37249018	0.526007335	-0.154
cg19823512	II	37	1	203489725		0.58	0.00041	3.39184	0.5120686	0.666025595	-0.154
cg10117369	II	37	1	203734256	LAX1;LAX1	0.62	0.0001	3.98291	0.50845638	0.354762281	0.154
cg14118850	II	37	2	10447890	HPCAL1;HPCAL1	0.58	0.00041	3.39184	0.51657632	0.670599343	-0.154
cg22805603	II	37	2	32490766	NLRC4;NLRC4	0.46	0.01197	1.92207	0.70059158	0.854803809	-0.154
cg17258387	II	37	2	174924430		0.46	0.01197	1.92207	0.30874956	0.462447278	-0.154
cg08162476	I	37	3	13064001	IQSEC1	0.5	0.00432	2.36443	0.4766986	0.32270856	0.154
cg01357892	II	37	3	126191181	ZXDC;ZXDC	0.54	0.0014	2.85345	0.27534448	0.429110203	-0.154
cg02832697	II	37	4	154126481	TRIM2;TRIM2	0.5	0.00432	2.36443	0.3407521	0.187043495	0.154
cg13826452	II	37	6	26758395		0.46	0.01197	1.92207	0.42923822	0.583127279	-0.154
cg14205216	II	37	6	33877308		0.5	0.00432	2.36443	0.68184933	0.836293442	-0.154
cg19382157	I	37	7	2124566	MAD1L1;MAD1L1;M	0.54	0.0014	2.85345	0.65157599	0.805409552	-0.154
cg26438325	II	37	7	28176718	JAZF1	0.62	0.0001	3.98291	0.69974512	0.8536516	-0.154
cg14519534	II	37	7	102082149	ORAI2;ORAI2	0.46	0.01197	1.92207	0.35583258	0.20192071	0.154
cg16703934	II	37	7	128786732	TSPAN33	0.54	0.0014	2.85345	0.73972	0.585436247	0.154
cg03979241	I	37	8	21916824	EPB49;EPB49;EPB4	0.67	2.34E-05	4.63072	0.54515779	0.699529145	-0.154
cg24568796	II	37	8	99916985		0.54	0.0014	2.85345	0.69625281	0.850119495	-0.154
cg03066050	II	37	9	110249749	KLF4	0.5	0.00432	2.36443	0.23956873	0.393268569	-0.154
cg14288424	II	37	9	132429922	PRRX2	0.62	0.0001	3.98291	0.40692633	0.560845582	-0.154
cg21228270	II	37	9	133871033		0.67	2.34E-05	4.63072	0.31920605	0.473232412	-0.154
cg14121282	I	37	9	137268074	RXRA	0.62	0.0001	3.98291	0.2762722	0.430322707	-0.154
cg24480146	II	37	10	43430536		0.67	2.34E-05	4.63072	0.67419729	0.828605314	-0.154
cg22679940	II	37	10	71097650	HK1;HK1;HK1;HK1;I	0.58	0.00041	3.39184	0.45576197	0.609802649	-0.154
cg19262315	II	37	11	67325980		0.42	0.02991	1.52413	0.81223026	0.658131706	0.154
cg04356090	I	37	12	129280133	SLC15A4	0.42	0.02991	1.52413	0.85038782	0.696556528	0.154
cg09660810	II	37	12	132270673	SFRS8	0.58	0.00041	3.39184	0.56622355	0.720110149	-0.154

cg11381282	I	37	13	20763366	GJB2	0.46	0.01197	1.92207	0.5936857	0.748060063	-0.154
cg11288831	II	37	13	114880831	RASA3	0.42	0.02991	1.52413	0.37649306	0.53077866	-0.154
cg02078710	II	37	14	59066094		0.5	0.00432	2.36443	0.69714113	0.543563139	0.154
cg06634140	II	37	14	95956325		0.46	0.01197	1.92207	0.69635409	0.850395032	-0.154
cg26146569	II	37	15	31637592	KLF13	0.5	0.00432	2.36443	0.64533984	0.491810639	0.154
cg00154119	II	37	15	68174054		0.54	0.0014	2.85345	0.54802192	0.702208417	-0.154
cg09782560	II	37	15	92058845		0.46	0.01197	1.92207	0.27808454	0.432316297	-0.154
cg02074956	I	37	16	4730657	MGRN1;MGRN1;M(	0.46	0.01197	1.92207	0.65031261	0.804278166	-0.154
cg03699843	II	37	16	50701064	SNX20;SNX20	0.46	0.01197	1.92207	0.31323496	0.467569663	-0.154
cg07508446	II	37	16	85966197		0.46	0.01197	1.92207	0.66307686	0.509443019	0.154
cg23792308	II	37	17	14104659	COX10	0.42	0.02991	1.52413	0.43284091	0.587152464	-0.154
cg24718015	II	37	17	40489721	STAT3;STAT3;STAT3	0.46	0.01197	1.92207	0.22867322	0.382731833	-0.154
cg08217545	I	37	19	3388013	NFIC;NFIC	0.67	2.34E-05	4.63072	0.70176466	0.856162314	-0.154
cg18217136	II	37	20	36157651	BLCAP;BLCAP	0.71	4.57E-06	5.34042	0.61130114	0.765431147	-0.154
cg06850285	II	37	22	21921269	UBE2L3;UBE2L3;UB	0.5	0.00432	2.36443	0.18513288	0.339456334	-0.154
cg15685223	II	37	22	25084556		0.54	0.0014	2.85345	0.82791064	0.67350717	0.154
cg20026613	I	37	22	45609419	C22orf9;C22orf9	0.62	0.0001	3.98291	0.47455033	0.628501415	-0.154
cg27430637	II	37	1	26646801	CD52	0.62	0.0001	3.98291	0.49708626	0.343921837	0.153
cg09502149	II	37	1	43406792	SLC2A1	0.62	0.0001	3.98291	0.4587432	0.611956082	-0.153
cg10505902	I	37	1	144892111	PDE4DIP;PDE4DIP;F	0.58	0.00041	3.39184	0.73639316	0.889582328	-0.153
cg00791854	II	37	1	154392070	IL6R;IL6R	0.5	0.00432	2.36443	0.30676141	0.460067488	-0.153
cg21824010	II	37	1	247578859	NLRP3;NLRP3	0.54	0.0014	2.85345	0.43095007	0.583533057	-0.153
cg06202737	I	37	2	128166279		0.5	0.00432	2.36443	0.51042623	0.663824555	-0.153
cg05795849	II	37	3	4794082	ITPR1;ITPR1;ITPR1;	0.46	0.01197	1.92207	0.20921488	0.362433853	-0.153
cg13035046	II	37	3	13115843	IQSEC1	0.58	0.00041	3.39184	0.54141238	0.69455844	-0.153
cg06806891	II	37	3	128323079		0.62	0.0001	3.98291	0.52400194	0.67743345	-0.153
cg02499139	II	37	3	171146337	TNIK;TNIK;TNIK;TNI	0.46	0.01197	1.92207	0.16965454	0.323027478	-0.153
cg14480507	II	37	4	6021538		0.58	0.00041	3.39184	0.36244275	0.515451648	-0.153
cg20682563	II	37	5	133460874	TCF7;TCF7;TCF7;TC	0.54	0.0014	2.85345	0.55819635	0.404943835	0.153
cg25944717	II	37	5	180648843	TRIM41;TRIM41	0.42	0.02991	1.52413	0.73286474	0.580323134	0.153
cg02029843	II	37	6	11278005	NEDD9	0.5	0.00432	2.36443	0.83008788	0.676907422	0.153
cg19400926	II	37	6	28889996	TRIM27	0.42	0.02991	1.52413	0.34026097	0.493128448	-0.153
cg17043247	II	37	6	33083989	HLA-DPB2	0.58	0.00041	3.39184	0.55901696	0.711770572	-0.153

cg01719223	II	37	7	2205727	MAD1L1;MAD1L1;N	0.5	0.00432	2.36443	0.48920862	0.336517479	0.153
cg22439641	II	37	7	142045212		0.5	0.00432	2.36443	0.59433728	0.441659303	0.153
cg07632771	II	37	7	148611823		0.46	0.01197	1.92207	0.70621541	0.553165458	0.153
cg19223782	I	37	11	503807	RNH1;RNH1;RNH1;	0.54	0.0014	2.85345	0.53923363	0.692366227	-0.153
cg26438284	I	37	11	2406307	CD81	0.5	0.00432	2.36443	0.56242196	0.409211652	0.153
cg14449910	II	37	11	2919689	SLC22A18AS;SLC22	0.46	0.01197	1.92207	0.48458799	0.637100051	-0.153
cg01425746	II	37	11	45671369	CHST1	0.54	0.0014	2.85345	0.63375446	0.787004347	-0.153
cg06928797	II	37	11	116966699	SIK3	0.62	0.0001	3.98291	0.18628533	0.339688684	-0.153
cg23128949	I	37	12	124986124	NCOR2;NCOR2	0.5	0.00432	2.36443	0.30007633	0.453161814	-0.153
cg21253130	II	37	13	32609104	FRY	0.5	0.00432	2.36443	0.66576624	0.818629107	-0.153
cg10843707	II	37	14	52510701	NID2	0.42	0.02991	1.52413	0.73829464	0.58558384	0.153
cg23369670	II	37	14	104171944	XRCC3;XRCC3;XRCC	0.54	0.0014	2.85345	0.47094992	0.624227964	-0.153
cg05059480	II	37	15	40633202	C15orf52	0.54	0.0014	2.85345	0.41942814	0.572059958	-0.153
cg25875049	II	37	15	43531947	TGM5;TGM5	0.58	0.00041	3.39184	0.3065998	0.459809493	-0.153
cg25290178	I	37	15	93591806	RGMA;RGMA;RGM	0.58	0.00041	3.39184	0.62065158	0.773611583	-0.153
cg27326750	II	37	16	11367302	PRM3	0.71	4.57E-06	5.34042	0.57061483	0.723940082	-0.153
cg07625774	I	37	16	89036057	CBFA2T3	0.62	0.0001	3.98291	0.81418462	0.660951899	0.153
cg09695851	I	37	17	3907499		0.62	0.0001	3.98291	0.7187722	0.871376409	-0.153
cg14089110	II	37	17	7018627	ASGR2;ASGR2;ASGI	0.58	0.00041	3.39184	0.28705686	0.440420524	-0.153
cg14039779	II	37	17	41857714	DUSP3;C17orf105	0.5	0.00432	2.36443	0.4826048	0.635940561	-0.153
cg12840847	II	37	17	75208309	SEC14L1;SEC14L1;S	0.5	0.00432	2.36443	0.81433265	0.661132897	0.153
cg09930046	II	37	19	840737	PRTN3	0.62	0.0001	3.98291	0.38737707	0.540639484	-0.153
cg07239938	II	37	19	852813	ELANE	0.58	0.00041	3.39184	0.38806938	0.5411954	-0.153
cg02319986	II	37	19	8568712	PRAM1	0.5	0.00432	2.36443	0.62202181	0.775076136	-0.153
cg01279933	II	37	19	10663970	ATG4D;KRI1	0.5	0.00432	2.36443	0.41210117	0.258882311	0.153
cg06579354	II	37	19	41618922	CYP2F1	0.54	0.0014	2.85345	0.69260708	0.539658136	0.153
cg08425610	II	37	19	46029456	VASP	0.67	2.34E-05	4.63072	0.54468435	0.697318424	-0.153
cg11399589	II	37	20	36147549	BLCAP;BLCAP;BLCA	0.58	0.00041	3.39184	0.67594411	0.829251806	-0.153
cg27585822	II	37	20	49253552	FAM65C	0.75	7.61E-07	6.11857	0.44021571	0.593395772	-0.153
cg07912723	II	37	1	2032058	PRKCZ;PRKCZ	0.54	0.0014	2.85345	0.7261725	0.574264628	0.152
cg11717881	II	37	1	44513921		0.71	4.57E-06	5.34042	0.21514467	0.367305963	-0.152
cg06946837	I	37	1	53972003	GLIS1	0.5	0.00432	2.36443	0.57834444	0.730223173	-0.152
cg24897320	II	37	1	110042528	CYB561D1;CYB561I	0.62	0.0001	3.98291	0.65340893	0.805873642	-0.152

cg10528484	II	37	1	204265410	PLEKHA6	0.46	0.01197	1.92207	0.4646427	0.616258539	-0.152
cg10809134	II	37	2	47879734		0.46	0.01197	1.92207	0.73890438	0.587011126	0.152
cg19867579	II	37	2	175643491		0.62	0.0001	3.98291	0.51446681	0.66623142	-0.152
cg00458932	II	37	2	208199465		0.58	0.00041	3.39184	0.70253526	0.550782503	0.152
cg20296298	II	37	2	210074020		0.42	0.02991	1.52413	0.85330362	0.701316447	0.152
cg20793665	I	37	2	232549224		0.58	0.00041	3.39184	0.62558103	0.473567557	0.152
cg15072976	I	37	2	242715549	GAL3ST2	0.54	0.0014	2.85345	0.67237822	0.823901283	-0.152
cg11600734	II	37	3	4794020	ITPR1;ITPR1;ITPR1;	0.54	0.0014	2.85345	0.3139527	0.466110742	-0.152
cg00907288	I	37	3	9178249	SRGAP3;SRGAP3	0.58	0.00041	3.39184	0.14839518	0.300580447	-0.152
cg04475307	II	37	3	9694905	MTMR14;MTMR14	0.58	0.00041	3.39184	0.78102516	0.628742287	0.152
cg24052964	II	37	5	142226498	ARHGAP26;ARHGAI	0.46	0.01197	1.92207	0.64974907	0.80156299	-0.152
cg06654628	II	37	5	150018914	SYNPO;SYNPO;SYNI	0.54	0.0014	2.85345	0.30921875	0.461111045	-0.152
cg15601361	II	37	6	11771493	C6orf105;C6orf105	0.54	0.0014	2.85345	0.62748026	0.779272764	-0.152
cg12044213	II	37	6	31124978	CCHCR1;TCF19;CCH	0.54	0.0014	2.85345	0.3569318	0.508436163	-0.152
cg09637172	I	37	6	31545252	TNF	0.46	0.01197	1.92207	0.81379728	0.66171175	0.152
cg12048225	II	37	6	32808669	PSMB8;PSMB8	0.5	0.00432	2.36443	0.29755339	0.145665354	0.152
cg18818075	II	37	6	44528865		0.67	2.34E-05	4.63072	0.37150409	0.523363093	-0.152
cg23045991	II	37	7	101943005	SH2B2	0.5	0.00432	2.36443	0.32723068	0.479145582	-0.152
cg13726789	II	37	8	37678323	GPR124	0.67	2.34E-05	4.63072	0.5785923	0.730162517	-0.152
cg00799984	II	37	8	134307105	NDRG1;NDRG1	0.54	0.0014	2.85345	0.67399116	0.825628493	-0.152
cg14151976	II	37	9	37565559	FBXO10	0.5	0.00432	2.36443	0.81626063	0.664037728	0.152
cg02127980	II	37	9	137252116	RXRA	0.62	0.0001	3.98291	0.32593645	0.47770817	-0.152
cg07676145	II	37	10	73506646	CDH23	0.58	0.00041	3.39184	0.37043866	0.522431439	-0.152
cg15092239	I	37	10	129678749	CLRN3	0.54	0.0014	2.85345	0.46272764	0.614943147	-0.152
cg06061966	II	37	11	46366643	DGKZ;DGKZ	0.67	2.34E-05	4.63072	0.43812683	0.590340588	-0.152
cg07104417	I	37	11	69259247		0.58	0.00041	3.39184	0.78624787	0.938313672	-0.152
cg18567954	I	37	12	113496168	DTX1	0.5	0.00432	2.36443	0.69457467	0.542098263	0.152
cg04706229	II	37	14	50550589	C14orf183	0.5	0.00432	2.36443	0.8240122	0.672238131	0.152
cg02057747	II	37	14	69408849	ACTN1;ACTN1;ACTI	0.58	0.00041	3.39184	0.38942592	0.541276744	-0.152
cg10070185	I	37	14	94857151	SERPINA1;SERPINA	0.5	0.00432	2.36443	0.55353498	0.705233353	-0.152
cg13496568	II	37	14	103374508		0.5	0.00432	2.36443	0.82526216	0.673297948	0.152
cg25290779	II	37	15	63886947		0.62	0.0001	3.98291	0.75854189	0.606149785	0.152
cg25607876	II	37	15	93580312		0.54	0.0014	2.85345	0.63074526	0.78248518	-0.152

cg04787728	II	37	16	3598600	NLRC3	0.46	0.01197	1.92207	0.4503097	0.298049483	0.152
cg03726236	I	37	16	28935666	RABEP2	0.58	0.00041	3.39184	0.41906131	0.267237202	0.152
cg16583186	I	37	16	81526361	CMIP	0.5	0.00432	2.36443	0.52576223	0.677569458	-0.152
cg26603047	I	37	16	88838368	FAM38A	0.54	0.0014	2.85345	0.61880804	0.467006838	0.152
cg27102649	I	37	17	1665172	SERPINF1	0.54	0.0014	2.85345	0.72370018	0.876073962	-0.152
cg20765716	I	37	17	3704602	ITGAE	0.54	0.0014	2.85345	0.73642874	0.888924135	-0.152
cg22640868	II	37	17	26661374	TNFAIP1;IFT20	0.58	0.00041	3.39184	0.20577663	0.35752732	-0.152
cg24223942	II	37	17	29152934	CRLF3	0.46	0.01197	1.92207	0.51666509	0.668724048	-0.152
cg04967578	II	37	18	13641894	C18orf1;C18orf1;C1	0.58	0.00041	3.39184	0.43658427	0.588759828	-0.152
cg16387467	II	37	18	72166016	CNDP2;CNDP2	0.46	0.01197	1.92207	0.28345088	0.435787465	-0.152
cg03502002	I	37	18	74962133	GALR1;GALR1	0.46	0.01197	1.92207	0.07849968	0.23002668	-0.152
cg17173442	I	37	19	19305340	RFXANK;RFXANK	0.5	0.00432	2.36443	0.52597601	0.677518652	-0.152
cg17618872	II	37	20	32235621	CBFA2T2;CBFA2T2;	0.62	0.0001	3.98291	0.79273076	0.64059629	0.152
cg01332882	I	37	20	47874155	ZNFX1	0.54	0.0014	2.85345	0.74897953	0.90059534	-0.152
cg01557221	II	37	22	36558091	APOL3;APOL3;APOl	0.58	0.00041	3.39184	0.77026178	0.617817944	0.152
cg03834031	II	37	22	46465717		0.5	0.00432	2.36443	0.13740915	0.289143951	-0.152
cg00300303	II	37	1	1067223		0.5	0.00432	2.36443	0.62335529	0.774244123	-0.151
cg05957749	II	37	1	39875787	KIAA0754;KIAA075	0.58	0.00041	3.39184	0.56492341	0.716385866	-0.151
cg07313701	II	37	1	42447165		0.5	0.00432	2.36443	0.26260808	0.413939611	-0.151
cg15562346	I	37	2	239403295		0.5	0.00432	2.36443	0.82377401	0.672760872	0.151
cg11550064	II	37	2	240148191	HDAC4	0.54	0.0014	2.85345	0.68143945	0.83224143	-0.151
cg03610527	I	37	2	242174889	HDLBP;HDLBP	0.54	0.0014	2.85345	0.773192	0.924190357	-0.151
cg25234117	II	37	3	155423168	PLCH1	0.46	0.01197	1.92207	0.44438354	0.595820519	-0.151
cg04431508	II	37	3	171693162		0.54	0.0014	2.85345	0.50084131	0.651886665	-0.151
cg22831526	II	37	3	194706168		0.62	0.0001	3.98291	0.35069568	0.501458025	-0.151
cg09249657	II	37	4	40518806	RBM47;RBM47	0.58	0.00041	3.39184	0.58851167	0.437755639	0.151
cg26646659	II	37	5	55776364		0.54	0.0014	2.85345	0.57325168	0.422575957	0.151
cg19008097	II	37	5	140012986	CD14;CD14;CD14	0.67	2.34E-05	4.63072	0.36907247	0.520408311	-0.151
cg25613180	II	37	5	149513303	PDGFRB	0.5	0.00432	2.36443	0.56527319	0.715914996	-0.151
cg15382302	II	37	6	3848866	FAM50B	0.58	0.00041	3.39184	0.6557723	0.806587977	-0.151
cg25637655	I	37	6	29911542	HLA-A	0.5	0.00432	2.36443	0.50612217	0.35490792	0.151
cg23204396	II	37	6	31548107		0.54	0.0014	2.85345	0.64279323	0.491332065	0.151
cg01300096	II	37	6	33384490	CUTA;CUTA;CUTA;(	0.58	0.00041	3.39184	0.64413774	0.493313217	0.151

cg12070911	II	37	6	150209640	RAET1E	0.46	0.01197	1.92207	0.69258306	0.541298966	0.151
cg20867674	II	37	6	163768411		0.58	0.00041	3.39184	0.67138726	0.822803379	-0.151
cg04450052	II	37	6	170525426		0.42	0.02991	1.52413	0.5976332	0.44652284	0.151
cg16492851	II	37	7	1062681	C7orf50;MIR339;C7	0.46	0.01197	1.92207	0.57676781	0.425954041	0.151
cg16495448	II	37	7	3019260	CARD11	0.46	0.01197	1.92207	0.376356	0.224864616	0.151
cg27342333	II	37	7	75592418	POR	0.58	0.00041	3.39184	0.36459483	0.515500393	-0.151
cg06959773	II	37	7	76033795	SRCRB4D;ZP3	0.67	2.34E-05	4.63072	0.67896919	0.830298359	-0.151
cg25823085	I	37	7	87105416	ABCB4;ABCB4;ABCE	0.58	0.00041	3.39184	0.53944981	0.388489787	0.151
cg12797746	I	37	8	1110520		0.58	0.00041	3.39184	0.72903669	0.578060797	0.151
cg22062741	II	37	8	1897075	ARHGEF10	0.54	0.0014	2.85345	0.70193096	0.853340105	-0.151
cg14918744	II	37	8	23018464	TNFRSF10D	0.5	0.00432	2.36443	0.76292477	0.611627917	0.151
cg00053916	II	37	8	37457329		0.62	0.0001	3.98291	0.6900732	0.840720516	-0.151
cg27040700	II	37	8	91017761	DECR1	0.42	0.02991	1.52413	0.29187926	0.442646961	-0.151
cg20173011	II	37	8	101507705		0.58	0.00041	3.39184	0.26925638	0.117969966	0.151
cg09723776	II	37	8	126320143	NSMCE2	0.5	0.00432	2.36443	0.73631411	0.887017845	-0.151
cg13569051	II	37	9	124051703	GSN;GSN;GSN;GSN;	0.58	0.00041	3.39184	0.54236705	0.693740381	-0.151
cg21234265	II	37	9	132370351		0.54	0.0014	2.85345	0.35417951	0.504989307	-0.151
cg13655250	II	37	9	134544528	RAPGEF1;RAPGEF1	0.54	0.0014	2.85345	0.28483264	0.133523795	0.151
cg14108380	I	37	9	139299357	SDCCAG3;SDCCAG3	0.46	0.01197	1.92207	0.88682577	0.735509276	0.151
cg26555531	II	37	10	1507708	ADARB2	0.5	0.00432	2.36443	0.71183495	0.561239253	0.151
cg14038949	II	37	10	6262673	PFKFB3;PFKFB3	0.42	0.02991	1.52413	0.48878775	0.337854788	0.151
cg21607649	II	37	10	134884177		0.62	0.0001	3.98291	0.57972379	0.429050076	0.151
cg18101140	II	37	11	67142001	LOC100130987;CLC	0.54	0.0014	2.85345	0.27200788	0.422918937	-0.151
cg10346364	II	37	11	67142268	LOC100130987;CLC	0.58	0.00041	3.39184	0.37021834	0.52075801	-0.151
cg02845204	II	37	11	71259439	KRTAP5-9	0.54	0.0014	2.85345	0.37460306	0.525186961	-0.151
cg25221207	II	37	11	119555633	PVRL1;PVRL1;PVRL1	0.54	0.0014	2.85345	0.23559251	0.386193065	-0.151
cg06715628	II	37	12	52482292		0.54	0.0014	2.85345	0.42148973	0.270681691	0.151
cg03690812	II	37	12	111875483	SH2B3	0.67	2.34E-05	4.63072	0.6108312	0.762178012	-0.151
cg27431877	II	37	12	124911924	NCOR2;NCOR2	0.54	0.0014	2.85345	0.66932022	0.820167752	-0.151
cg06777732	I	37	12	131118426		0.42	0.02991	1.52413	0.25868778	0.107410408	0.151
cg00465247	II	37	13	50703477		0.5	0.00432	2.36443	0.6424147	0.491077166	0.151
cg15839421	II	37	17	1636454	WDR81;WDR81;WD	0.5	0.00432	2.36443	0.36903307	0.218247203	0.151
cg07323451	II	37	17	3635580	ITGAE	0.42	0.02991	1.52413	0.49275206	0.341694613	0.151

cg14394837	II	37	17	25802282	KSR1	0.5	0.00432	2.36443	0.69759733	0.546617039	0.151
cg08681966	II	37	17	29054723		0.67	2.34E-05	4.63072	0.39501903	0.545860867	-0.151
cg05655281	I	37	18	76322900		0.5	0.00432	2.36443	0.65010685	0.801126948	-0.151
cg23690893	I	37	19	14671371	TECR	0.54	0.0014	2.85345	0.42882704	0.579767079	-0.151
cg19010566	II	37	20	23062796	CD93	0.46	0.01197	1.92207	0.57033031	0.72146121	-0.151
cg13374901	II	37	20	60639404	TAF4	0.58	0.00041	3.39184	0.50367548	0.654244472	-0.151
cg21213332	II	37	21	45345433	AGPAT3;AGPAT3	0.54	0.0014	2.85345	0.54833184	0.698972612	-0.151
cg10107292	II	37	1	15504438	TMEM51;TMEM51;	0.58	0.00041	3.39184	0.20533372	0.355789157	-0.15
cg20812722	II	37	1	25525951		0.46	0.01197	1.92207	0.60572112	0.455508343	0.15
cg06659338	II	37	1	54562040	C1orf83	0.71	4.57E-06	5.34042	0.39769328	0.54798756	-0.15
cg27058077	I	37	1	200337470		0.46	0.01197	1.92207	0.24073589	0.391204203	-0.15
cg27386431	I	37	1	200990482	KIF21B	0.58	0.00041	3.39184	0.57362769	0.423663213	0.15
cg09264489	II	37	1	204257406	PLEKHA6	0.58	0.00041	3.39184	0.65829915	0.808379496	-0.15
cg23228450	II	37	1	205618102		0.62	0.0001	3.98291	0.60089718	0.450874253	0.15
cg06093152	II	37	1	212662017		0.5	0.00432	2.36443	0.56878514	0.718993818	-0.15
cg04694619	II	37	2	242127759	ANO7;ANO7	0.54	0.0014	2.85345	0.72643339	0.57674602	0.15
cg26062204	II	37	3	150661892	CLRN1;CLRN1;CLRN	0.54	0.0014	2.85345	0.80389383	0.654357044	0.15
cg07277038	II	37	3	196373543	LRRC33	0.5	0.00432	2.36443	0.58354317	0.73399547	-0.15
cg17055207	II	37	4	8412736	ACOX3;ACOX3	0.46	0.01197	1.92207	0.78426731	0.63416034	0.15
cg16234490	II	37	4	77138082		0.58	0.00041	3.39184	0.6023845	0.751995076	-0.15
cg05017994	I	37	5	964562		0.46	0.01197	1.92207	0.65831079	0.507829018	0.15
cg11095383	I	37	5	140174887	PCDHA1;PCDHA1;P	0.46	0.01197	1.92207	0.28228516	0.432686117	-0.15
cg02214623	II	37	5	180671896	GNB2L1	0.46	0.01197	1.92207	0.45280773	0.602370956	-0.15
cg17709873	II	37	6	31540456	LTA;LTA	0.5	0.00432	2.36443	0.52820829	0.378127554	0.15
cg14997321	II	37	6	134436460		0.58	0.00041	3.39184	0.54672927	0.69642529	-0.15
cg03125341	II	37	6	157882207	ZDHHC14;ZDHHC14	0.54	0.0014	2.85345	0.26883303	0.11856234	0.15
cg18998543	II	37	7	2769865	GNA12	0.54	0.0014	2.85345	0.30722616	0.457703819	-0.15
cg16329896	II	37	7	47515060	TNS3	0.46	0.01197	1.92207	0.30358787	0.45312267	-0.15
cg25345520	II	37	7	116210196		0.5	0.00432	2.36443	0.68440784	0.534691447	0.15
cg18739367	II	37	8	38330740		0.58	0.00041	3.39184	0.40629806	0.555939521	-0.15
cg14184780	I	37	9	95475751	BICD2;BICD2	0.5	0.00432	2.36443	0.47604272	0.626405237	-0.15
cg14508093	I	37	9	98862825		0.46	0.01197	1.92207	0.30981544	0.45936557	-0.15
cg09045574	II	37	10	101686925	DNMBP;NCRNA000	0.42	0.02991	1.52413	0.48045182	0.630633735	-0.15

cg11727383	I	37	10	102988389	LBX1;FLJ41350	0.46	0.01197	1.92207	0.12629942	0.276031729	-0.15
cg07556018	II	37	11	2677341	KCNQ1;KCNQ1OT1;	0.62	0.0001	3.98291	0.42975751	0.580031277	-0.15
cg22766888	II	37	11	3188566		0.58	0.00041	3.39184	0.5788658	0.729228439	-0.15
cg20217257	II	37	11	64109158	CCDC88B	0.54	0.0014	2.85345	0.64203365	0.791757864	-0.15
cg25824218	II	37	12	25104798		0.46	0.01197	1.92207	0.7924343	0.642266493	0.15
cg12669088	II	37	12	25541364		0.5	0.00432	2.36443	0.79896011	0.649062288	0.15
cg09039672	II	37	12	52367989	ACVR1B;ACVR1B;A(	0.46	0.01197	1.92207	0.66213249	0.81246727	-0.15
cg16782719	II	37	12	129280334	SLC15A4	0.46	0.01197	1.92207	0.63717795	0.486856649	0.15
cg23251248	I	37	14	55907374	TBPL2	0.54	0.0014	2.85345	0.60523273	0.755319915	-0.15
cg24183098	II	37	14	101296297	MEG3;MEG3;MEG3	0.54	0.0014	2.85345	0.74211202	0.592199384	0.15
cg02256455	I	37	14	104165475	XRCC3;XRCC3;XRCC	0.54	0.0014	2.85345	0.57246016	0.422573085	0.15
cg19676553	II	37	14	105751629	BRF1	0.5	0.00432	2.36443	0.59563131	0.745147116	-0.15
cg26983304	II	37	14	106044101		0.46	0.01197	1.92207	0.39682125	0.247001293	0.15
cg06926377	II	37	15	40443853		0.54	0.0014	2.85345	0.65024193	0.800524584	-0.15
cg13048962	II	37	15	66790410	SNAPC5	0.54	0.0014	2.85345	0.25319626	0.402985198	-0.15
cg01678084	I	37	15	67022087	SMAD6;SMAD6;SM	0.62	0.0001	3.98291	0.85995484	0.709460548	0.15
cg02462661	II	37	15	74275622	STOML1	0.5	0.00432	2.36443	0.38567188	0.53527607	-0.15
cg20329220	II	37	15	78286521	LOC91450	0.5	0.00432	2.36443	0.37170878	0.222045438	0.15
cg05027554	I	37	16	646579	RAB40C	0.54	0.0014	2.85345	0.72934662	0.879181255	-0.15
cg05765011	II	37	16	4103225	ADCY9	0.58	0.00041	3.39184	0.49494152	0.64462857	-0.15
cg09005651	I	37	16	4740694	MGRN1;MGRN1;M(	0.62	0.0001	3.98291	0.60963972	0.759473246	-0.15
cg26843872	II	37	16	30368792	TBC1D10B	0.54	0.0014	2.85345	0.45048872	0.600030777	-0.15
cg05820066	II	37	16	75145843	LDHD;LDHD	0.54	0.0014	2.85345	0.44974519	0.599902817	-0.15
cg08675364	II	37	16	85362963		0.5	0.00432	2.36443	0.49809404	0.648348292	-0.15
cg05166473	I	37	16	88103629	BANP;BANP	0.54	0.0014	2.85345	0.74076932	0.590930555	0.15
cg09594635	I	37	16	89126866		0.5	0.00432	2.36443	0.81638618	0.666422668	0.15
cg05944840	II	37	16	89401621	ANKRD11	0.5	0.00432	2.36443	0.42203611	0.572274123	-0.15
cg09597638	II	37	17	3907349		0.58	0.00041	3.39184	0.58144468	0.731076436	-0.15
cg09098720	II	37	17	49412842		0.46	0.01197	1.92207	0.70071701	0.550722538	0.15
cg10723617	II	37	17	55533389	MSI2;MSI2	0.62	0.0001	3.98291	0.60044012	0.750437429	-0.15
cg06418113	II	37	18	43915901	RNF165	0.5	0.00432	2.36443	0.3297913	0.480192338	-0.15
cg03004350	I	37	19	847943	PRTN3	0.58	0.00041	3.39184	0.52078115	0.67088816	-0.15
cg22761077	I	37	19	850975	ELANE	0.5	0.00432	2.36443	0.30403448	0.454147403	-0.15

cg19357865	II	37	19	13947262	MIR27A;MIR24-2	0.58	0.00041	3.39184	0.41634952	0.566723082	-0.15
cg03214420	II	37	19	44257589	C19orf61	0.46	0.01197	1.92207	0.74532043	0.5955027	0.15
cg25739142	II	37	20	3206940		0.42	0.02991	1.52413	0.75163835	0.601818936	0.15
cg26482164	II	37	20	58630240	C20orf197	0.42	0.02991	1.52413	0.29655959	0.446120715	-0.15
cg24157349	II	37	22	47081751	CERK	0.46	0.01197	1.92207	0.18781925	0.337801598	-0.15
cg06939447	I	37	1	3589971	TP73	0.54	0.0014	2.85345	0.74222377	0.593485137	0.149
cg01208873	II	37	1	15127769	KIAA1026;KIAA102(	0.67	2.34E-05	4.63072	0.57979203	0.728653497	-0.149
cg26554265	II	37	1	23206348	EPHB2;EPHB2	0.58	0.00041	3.39184	0.66730602	0.518138641	0.149
cg02343604	II	37	1	23884703	ID3	0.62	0.0001	3.98291	0.50893656	0.359803269	0.149
cg26292918	II	37	1	151826205	THEM5	0.42	0.02991	1.52413	0.59136956	0.740737256	-0.149
cg10232893	II	37	1	230961405		0.67	2.34E-05	4.63072	0.61389449	0.762497849	-0.149
cg08031024	II	37	2	121617597	GLI2	0.46	0.01197	1.92207	0.70915464	0.559669613	0.149
cg17109049	II	37	2	220072753	ZFAND2B	0.54	0.0014	2.85345	0.61786562	0.766573073	-0.149
cg00638210	II	37	3	126732518	PLXNA1	0.5	0.00432	2.36443	0.6278502	0.77647326	-0.149
cg27312961	II	37	3	191048361	UTS2D;CCDC50;CCI	0.5	0.00432	2.36443	0.53511229	0.386133242	0.149
cg25512381	II	37	3	195342063		0.42	0.02991	1.52413	0.65869817	0.5094638	0.149
cg12811871	I	37	4	2322078	ZFYVE28	0.42	0.02991	1.52413	0.74903474	0.599744411	0.149
cg01831454	II	37	5	148417172	SH3TC2	0.62	0.0001	3.98291	0.38295776	0.531470272	-0.149
cg00987918	II	37	6	30115829	TRIM40	0.67	2.34E-05	4.63072	0.66978735	0.520381719	0.149
cg22752886	II	37	6	30619221	C6orf136;C6orf136	0.54	0.0014	2.85345	0.7492591	0.600238621	0.149
cg16553272	II	37	6	31847028	SLC44A4	0.42	0.02991	1.52413	0.67007077	0.819010469	-0.149
cg12397463	II	37	6	33128825		0.54	0.0014	2.85345	0.53175496	0.680508364	-0.149
cg24035447	I	37	6	48036613	C6orf138	0.46	0.01197	1.92207	0.13739366	0.286206254	-0.149
cg25028542	II	37	7	36429120	ANLN;KIAA0895	0.5	0.00432	2.36443	0.21659919	0.36609299	-0.149
cg13659700	II	37	7	97832841	LMTK2	0.54	0.0014	2.85345	0.73531125	0.586388531	0.149
cg01077501	II	37	7	134617592	CALD1;CALD1;CALD	0.5	0.00432	2.36443	0.67579799	0.527099582	0.149
cg17500680	II	37	8	134039099	TG	0.58	0.00041	3.39184	0.46944827	0.618629498	-0.149
cg25405138	II	37	10	34488427	PARD3	0.46	0.01197	1.92207	0.79982928	0.650469857	0.149
cg17237804	II	37	10	71199355		0.5	0.00432	2.36443	0.6784	0.82737554	-0.149
cg04304802	II	37	10	73499965	CDH23	0.58	0.00041	3.39184	0.2955626	0.444360871	-0.149
cg10552523	II	37	11	313478	IFITM1	0.54	0.0014	2.85345	0.3204293	0.171409081	0.149
cg23816537	II	37	11	60634869	ZP1	0.46	0.01197	1.92207	0.67455783	0.525204106	0.149
cg18395636	I	37	11	87908785	RAB38	0.54	0.0014	2.85345	0.12916087	0.277817963	-0.149

cg21585512	II	37	11	122030076	LOC399959	0.42	0.02991	1.52413	0.40281076	0.253903156	0.149
cg00765705	I	37	12	124865130	NCOR2;NCOR2	0.42	0.02991	1.52413	0.2287633	0.377269314	-0.149
cg00813509	I	37	12	124941317	NCOR2;NCOR2	0.67	2.34E-05	4.63072	0.52100242	0.372371991	0.149
cg14849578	II	37	12	125282480	SCARB1;SCARB1	0.58	0.00041	3.39184	0.63259517	0.78178679	-0.149
cg23670055	II	37	14	34481752		0.5	0.00432	2.36443	0.62162644	0.472848778	0.149
cg21960110	II	37	16	202482	HBZ	0.58	0.00041	3.39184	0.65954246	0.51090699	0.149
cg08409642	I	37	16	56641373	MT2A	0.54	0.0014	2.85345	0.44056017	0.589730432	-0.149
cg13375828	II	37	17	3598755	P2RX5;P2RX5;P2RX	0.5	0.00432	2.36443	0.34504667	0.195780598	0.149
cg24738036	II	37	17	3704476	ITGAE;ITGAE	0.5	0.00432	2.36443	0.60206713	0.750673162	-0.149
cg14192542	II	37	17	64187581	CCDC46	0.46	0.01197	1.92207	0.41306368	0.561928299	-0.149
cg26450228	II	37	17	64575581	PRKCA	0.54	0.0014	2.85345	0.6411071	0.790424722	-0.149
cg01040259	II	37	17	73314451	GRB2;GRB2	0.71	4.57E-06	5.34042	0.67106583	0.819907118	-0.149
cg21984481	II	37	17	79567631	NPLOC4	0.46	0.01197	1.92207	0.62686679	0.77577182	-0.149
cg24236839	II	37	19	5038822	KDM4B	0.67	2.34E-05	4.63072	0.4303356	0.579659974	-0.149
cg22322184	II	37	19	7734203	RETN	0.71	4.57E-06	5.34042	0.36190981	0.511235887	-0.149
cg24141382	II	37	1	41446726	CTPS	0.46	0.01197	1.92207	0.61404097	0.465675916	0.148
cg20697417	II	37	1	41786797		0.46	0.01197	1.92207	0.35814001	0.506425309	-0.148
cg26924218	II	37	1	155950963		0.42	0.02991	1.52413	0.63173013	0.483812079	0.148
cg23088126	II	37	1	207078912	FAIM3;FAIM3;FAIM	0.54	0.0014	2.85345	0.65477303	0.507117909	0.148
cg15557309	II	37	1	231155948	MIR1182;FAM89A	0.54	0.0014	2.85345	0.68831899	0.83649568	-0.148
cg22413209	I	37	2	112917281	FBLN7;FBLN7	0.5	0.00432	2.36443	0.78117323	0.633326565	0.148
cg18904855	II	37	2	231693469		0.58	0.00041	3.39184	0.6212814	0.473571643	0.148
cg11564239	II	37	2	241644188		0.54	0.0014	2.85345	0.58247701	0.43399786	0.148
cg13781758	II	37	3	122823409	PDIA5;PDIA5	0.42	0.02991	1.52413	0.69708705	0.548696284	0.148
cg02291010	II	37	5	96079433	CAST;CAST;CAST;CA	0.46	0.01197	1.92207	0.75421285	0.902393289	-0.148
cg02283238	II	37	5	118691126	TNFAIP8;TNFAIP8	0.54	0.0014	2.85345	0.67092546	0.523057249	0.148
cg05941376	II	37	5	167836834	WWC1;WWC1;WW	0.46	0.01197	1.92207	0.53058521	0.678932403	-0.148
cg13329862	II	37	6	30850876	DDR1;DDR1	0.5	0.00432	2.36443	0.74730333	0.599749463	0.148
cg25110523	II	37	6	31868025	ZBTB12	0.42	0.02991	1.52413	0.5022932	0.650372107	-0.148
cg16203210	II	37	6	41905026	CCND3;CCND3;CCN	0.54	0.0014	2.85345	0.72682728	0.874467312	-0.148
cg09471887	II	37	6	90660913	BACH2;BACH2	0.58	0.00041	3.39184	0.71198325	0.85999635	-0.148
cg04963607	II	37	7	598459	PRKAR1B;PRKAR1B	0.75	7.61E-07	6.11857	0.6863781	0.834475088	-0.148
cg17432857	II	37	7	27184438	HOXA5	0.5	0.00432	2.36443	0.59774082	0.746147308	-0.148

cg21252523	II	37	7	27254071		0.5	0.00432	2.36443	0.60679699	0.755054745	-0.148
cg27405731	I	37	7	101571203	CUX1;CUX1;CUX1	0.58	0.00041	3.39184	0.52724922	0.674832982	-0.148
cg05371552	I	37	7	158854564	VIPR2	0.54	0.0014	2.85345	0.36677314	0.514302484	-0.148
cg03280622	II	37	8	145023013	PLEC1;PLEC1;PLEC1	0.58	0.00041	3.39184	0.30816939	0.456557593	-0.148
cg15582789	II	37	9	99145525	SLC35D2	0.54	0.0014	2.85345	0.27083477	0.418654566	-0.148
cg13896650	II	37	9	116894310		0.62	0.0001	3.98291	0.68002712	0.531850767	0.148
cg01870113	II	37	9	140106010	NDOR1;NDOR1;ND(	0.5	0.00432	2.36443	0.52194343	0.669905799	-0.148
cg23797615	II	37	11	67132472	CLCF1;LOC1001309	0.46	0.01197	1.92207	0.44502093	0.296564627	0.148
cg03962678	II	37	11	118398094	TTC36	0.5	0.00432	2.36443	0.50006632	0.648482803	-0.148
cg25194194	II	37	12	69143431	SLC35E3	0.54	0.0014	2.85345	0.67685037	0.824663616	-0.148
cg24727480	II	37	12	109901083	KCTD10	0.58	0.00041	3.39184	0.27634471	0.423854167	-0.148
cg15985905	II	37	12	128078475		0.54	0.0014	2.85345	0.6896087	0.541620492	0.148
cg14366598	II	37	14	23841754	IL25;IL25	0.54	0.0014	2.85345	0.72334537	0.575623728	0.148
cg08140459	II	37	14	75086513		0.71	4.57E-06	5.34042	0.48503072	0.632925016	-0.148
cg11942439	II	37	15	41775282	RTF1	0.62	0.0001	3.98291	0.86638507	0.718359781	0.148
cg14573810	II	37	15	66270794	MEGF11	0.46	0.01197	1.92207	0.76048274	0.612572005	0.148
cg22095582	II	37	15	90131512	C15orf42	0.67	2.34E-05	4.63072	0.48435886	0.632789405	-0.148
cg08121317	II	37	15	101327691		0.54	0.0014	2.85345	0.55418046	0.702103334	-0.148
cg08766256	II	37	16	81527504	CMIP;CMIP	0.54	0.0014	2.85345	0.42387641	0.571596766	-0.148
cg04033022	II	37	16	89189383	ACSF3;ACSF3;ACSF3	0.54	0.0014	2.85345	0.734704	0.587071157	0.148
cg14509809	I	37	17	935884	ABR;ABR;ABR	0.42	0.02991	1.52413	0.66463028	0.516138261	0.148
cg23123250	I	37	17	4440116	SPNS2	0.58	0.00041	3.39184	0.4584641	0.606182858	-0.148
cg02329670	II	37	17	6921400	MIR497;MIR195	0.42	0.02991	1.52413	0.51004192	0.657993745	-0.148
cg13984330	II	37	17	58637589		0.46	0.01197	1.92207	0.71457088	0.862912397	-0.148
cg13647973	I	37	19	56061337		0.46	0.01197	1.92207	0.7536779	0.605807174	0.148
cg13951069	I	37	21	43919801	SLC37A1;SLC37A1	0.54	0.0014	2.85345	0.77368018	0.921940632	-0.148
cg23955417	II	37	1	26880928	MIR1976;RPS6KA1;	0.54	0.0014	2.85345	0.33215558	0.478731214	-0.147
cg02947253	II	37	1	112046700	ADORA3;ADORA3;A	0.58	0.00041	3.39184	0.67118842	0.818147962	-0.147
cg25349990	II	37	1	229294380		0.54	0.0014	2.85345	0.39993205	0.546724374	-0.147
cg13985485	I	37	1	239550283		0.46	0.01197	1.92207	0.12001369	0.266532024	-0.147
cg08102294	II	37	3	13063918	IQSEC1	0.46	0.01197	1.92207	0.49885185	0.352210055	0.147
cg03891318	II	37	3	139372675	NMNAT3	0.46	0.01197	1.92207	0.56887658	0.716366699	-0.147
cg07757358	II	37	3	187897193	LPP;FLJ42393	0.46	0.01197	1.92207	0.7848974	0.638383415	0.147

cg14643001	II	37	3	193848133		0.46	0.01197	1.92207	0.66769702	0.521035807	0.147
cg12407867	I	37	4	1167369	LOC100130872-SP(	0.62	0.0001	3.98291	0.75730384	0.904453016	-0.147
cg08968034	II	37	4	3022836	GRK4;GRK4;GRK4	0.58	0.00041	3.39184	0.67012345	0.523021303	0.147
cg21811204	I	37	5	132158625	SHROOM1	0.54	0.0014	2.85345	0.45881335	0.311594052	0.147
cg06019763	I	37	6	25028080		0.5	0.00432	2.36443	0.77720892	0.630364337	0.147
cg00920254	II	37	6	44189984	SLC29A1;SLC29A1;S	0.58	0.00041	3.39184	0.57222487	0.719723475	-0.147
cg16111231	II	37	6	160399700	IGF2R	0.54	0.0014	2.85345	0.51294145	0.365995228	0.147
cg18439339	II	37	7	151441268	PRKAG2;PRKAG2	0.54	0.0014	2.85345	0.47130879	0.618627961	-0.147
cg00004996	II	37	8	17271535	MTMR7	0.5	0.00432	2.36443	0.69813078	0.845458529	-0.147
cg05459517	II	37	8	123795904	ZHX2	0.5	0.00432	2.36443	0.84166858	0.694645752	0.147
cg07441152	II	37	8	144814911	FAM83H	0.42	0.02991	1.52413	0.75161218	0.604149567	0.147
cg03376308	II	37	10	3497811		0.58	0.00041	3.39184	0.34595352	0.492975937	-0.147
cg24760467	II	37	10	102760784	LZTS2	0.46	0.01197	1.92207	0.48160168	0.62831155	-0.147
cg10900641	I	37	11	1767793	HCCA2;LOC402778	0.62	0.0001	3.98291	0.79754072	0.944254883	-0.147
cg18458509	II	37	11	2920189	SLC22A18AS;SLC22	0.5	0.00432	2.36443	0.26928885	0.416355539	-0.147
cg10266336	II	37	11	18270324	SAA2;SAA2	0.5	0.00432	2.36443	0.31923245	0.466483666	-0.147
cg21994822	II	37	11	66741747	C11orf86	0.46	0.01197	1.92207	0.62728344	0.774456356	-0.147
cg26335578	II	37	11	68722738		0.71	4.57E-06	5.34042	0.44424754	0.590805175	-0.147
cg25273707	II	37	11	76037066		0.54	0.0014	2.85345	0.50514915	0.357854063	0.147
cg04468671	II	37	12	132337407		0.67	2.34E-05	4.63072	0.44720118	0.594155214	-0.147
cg24741430	II	37	15	67095711		0.75	7.61E-07	6.11857	0.6985679	0.845910828	-0.147
cg12157761	II	37	15	71005778	UACA	0.46	0.01197	1.92207	0.72881523	0.876078736	-0.147
cg04004158	I	37	16	1152474		0.42	0.02991	1.52413	0.87460835	0.728057125	0.147
cg02484343	I	37	16	2284734	E4F1	0.54	0.0014	2.85345	0.77058535	0.6235328	0.147
cg08065963	I	37	16	8985593		0.46	0.01197	1.92207	0.51141929	0.36484951	0.147
cg00649632	II	37	16	16108802	ABCC1;ABCC1;ABC(	0.5	0.00432	2.36443	0.70978183	0.562465981	0.147
cg08181850	II	37	16	17200244	XYLT1	0.54	0.0014	2.85345	0.53686003	0.683722507	-0.147
cg07434438	II	37	16	72961899	ZFHX3;ZFHX3	0.58	0.00041	3.39184	0.70348417	0.850566351	-0.147
cg22210779	I	37	17	80818772	TBCD	0.46	0.01197	1.92207	0.9270989	0.780056378	0.147
cg21121609	II	37	19	7766960	FCER2;FCER2	0.42	0.02991	1.52413	0.3661308	0.219311833	0.147
cg06905453	I	37	19	14089284	RFX1	0.46	0.01197	1.92207	0.222664	0.369988313	-0.147
cg19587280	I	37	19	18770726	KLHL26	0.67	2.34E-05	4.63072	0.65398457	0.506576707	0.147
cg15581429	II	37	19	39369353	SIRT2;SIRT2;RINL	0.67	2.34E-05	4.63072	0.58930446	0.442420415	0.147

cg26476925	II	37	19	45245446		0.58	0.00041	3.39184	0.29720822	0.443768271	-0.147
cg24737324	II	37	20	19867143		0.58	0.00041	3.39184	0.52395955	0.67061318	-0.147
cg12049875	II	37	20	19955868	RIN2	0.58	0.00041	3.39184	0.48686098	0.634119043	-0.147
cg06036288	II	37	20	61971412		0.62	0.0001	3.98291	0.39203017	0.53942035	-0.147
cg21291385	II	37	21	35448215	MRPS6;SLC5A3	0.54	0.0014	2.85345	0.47273723	0.620006503	-0.147
cg21871091	II	37	21	38349937	HLCS	0.46	0.01197	1.92207	0.52826798	0.674979873	-0.147
cg07048516	II	37	22	24105087	C22orf15	0.54	0.0014	2.85345	0.31159619	0.458819921	-0.147
cg26001125	II	37	22	24823050	ADORA2A	0.58	0.00041	3.39184	0.55814385	0.411184457	0.147
cg01403010	II	37	22	38609534	MAFF;MAFF;MAFF;	0.5	0.00432	2.36443	0.34333189	0.4905855	-0.147
cg24004483	I	37	1	944783		0.42	0.02991	1.52413	0.42102706	0.567238531	-0.146
cg17163404	II	37	1	19414438	UBR4	0.42	0.02991	1.52413	0.74189058	0.595439189	0.146
cg26527263	I	37	1	53875972		0.58	0.00041	3.39184	0.68330782	0.537099866	0.146
cg25103275	II	37	1	111025075	CYMP	0.5	0.00432	2.36443	0.67864469	0.824161279	-0.146
cg01417714	II	37	1	155223726	FAM189B;FAM189I	0.54	0.0014	2.85345	0.4921068	0.638193857	-0.146
cg22787719	II	37	1	209570949		0.58	0.00041	3.39184	0.22849111	0.374045523	-0.146
cg02961280	II	37	2	220115026	STK16;STK16;TUBA	0.54	0.0014	2.85345	0.27357438	0.419388991	-0.146
cg14512813	II	37	3	187618877		0.46	0.01197	1.92207	0.78823946	0.642311352	0.146
cg02014217	II	37	4	1015600	FGFRL1;FGFRL1;FGI	0.46	0.01197	1.92207	0.44029537	0.294685505	0.146
cg24403649	II	37	4	39172243		0.5	0.00432	2.36443	0.59750316	0.74371622	-0.146
cg22021794	II	37	5	54177306		0.54	0.0014	2.85345	0.76113358	0.614995324	0.146
cg26704043	II	37	6	5282702	FARS2	0.46	0.01197	1.92207	0.5641281	0.710177188	-0.146
cg06694826	II	37	6	15675176		0.58	0.00041	3.39184	0.35430794	0.500068014	-0.146
cg05859264	II	37	6	36097692	MAPK13	0.5	0.00432	2.36443	0.56690492	0.420600196	0.146
cg22719623	II	37	6	154360732	OPRM1;OPRM1;OP	0.5	0.00432	2.36443	0.50159776	0.647241334	-0.146
cg14587213	II	37	7	213703	FAM20C	0.62	0.0001	3.98291	0.59060041	0.736346657	-0.146
cg04453501	I	37	7	533963		0.42	0.02991	1.52413	0.70913346	0.855254465	-0.146
cg21768702	II	37	7	1040081	C7orf50;C7orf50;C7	0.46	0.01197	1.92207	0.61119557	0.464977463	0.146
cg17633015	I	37	7	2020501	MAD1L1;MAD1L1;N	0.42	0.02991	1.52413	0.7884938	0.642048393	0.146
cg23532927	II	37	7	45977067		0.54	0.0014	2.85345	0.43216994	0.578232372	-0.146
cg01092213	II	37	7	48127925	UPP1;UPP1	0.5	0.00432	2.36443	0.26902213	0.415340418	-0.146
cg18245976	II	37	7	158708271	WDR60	0.58	0.00041	3.39184	0.36254723	0.508572769	-0.146
cg13582028	II	37	8	631115	ERICH1	0.71	4.57E-06	5.34042	0.34769647	0.493384678	-0.146
cg16292768	II	37	8	27467783	CLU;CLU;CLU	0.54	0.0014	2.85345	0.62477929	0.771016544	-0.146

cg03400695	II	37	8	142162582	DENND3	0.58	0.00041	3.39184	0.71175556	0.857743318	-0.146
cg08841552	II	37	9	140444378	PNPLA7;PNPLA7	0.42	0.02991	1.52413	0.5094479	0.363781254	0.146
cg10427868	II	37	10	80936798	ZMIZ1	0.5	0.00432	2.36443	0.47133449	0.324918925	0.146
cg17879101	I	37	10	126329354	FAM53B	0.42	0.02991	1.52413	0.38891044	0.534636207	-0.146
cg20456620	II	37	10	134377343	INPP5A	0.42	0.02991	1.52413	0.79488851	0.648678022	0.146
cg03411376	I	37	11	2432720	TRPM5	0.62	0.0001	3.98291	0.59420496	0.73972323	-0.146
cg13332953	II	37	11	12003759	DKK3;DKK3;DKK3	0.46	0.01197	1.92207	0.70612769	0.560335116	0.146
cg20792833	II	37	11	67205195	PTPRCAP	0.58	0.00041	3.39184	0.27997807	0.134081564	0.146
cg06213635	I	37	11	129488336		0.5	0.00432	2.36443	0.61860259	0.764199882	-0.146
cg23702046	II	37	12	3371110	TSPAN9;TSPAN9	0.67	2.34E-05	4.63072	0.70443893	0.850892152	-0.146
cg04772575	I	37	12	123431865	ABCB9;ABCB9;ABCI	0.58	0.00041	3.39184	0.42996776	0.575591123	-0.146
cg24722198	II	37	13	113567732		0.42	0.02991	1.52413	0.6468401	0.500796029	0.146
cg03875678	II	37	14	25103546	GZMB	0.42	0.02991	1.52413	0.26557094	0.411293127	-0.146
cg21752660	II	37	15	52824726		0.46	0.01197	1.92207	0.31672514	0.463217767	-0.146
cg26891150	I	37	15	79575634	ANKRD34C	0.58	0.00041	3.39184	0.24191985	0.388199149	-0.146
cg06578342	II	37	16	4349215		0.67	2.34E-05	4.63072	0.39388228	0.53957343	-0.146
cg24366564	I	37	17	2843149	RAP1GAP2;RAP1GA	0.58	0.00041	3.39184	0.65894919	0.804974134	-0.146
cg13330559	I	37	17	11144043	SHISA6	0.46	0.01197	1.92207	0.03829844	0.184498648	-0.146
cg15668538	II	37	17	15931082	TTC19	0.46	0.01197	1.92207	0.72838291	0.582846935	0.146
cg04308167	II	37	17	19440767	SLC47A1	0.5	0.00432	2.36443	0.30932601	0.45537144	-0.146
cg10334489	II	37	17	25798878	KSR1	0.58	0.00041	3.39184	0.59612837	0.450437975	0.146
cg20673721	II	37	17	25859382	KSR1	0.62	0.0001	3.98291	0.59352952	0.739861565	-0.146
cg25246246	II	37	17	43503131	ARHGAP27	0.54	0.0014	2.85345	0.46522856	0.611371103	-0.146
cg11434671	II	37	17	74524611	CYGB	0.67	2.34E-05	4.63072	0.46327191	0.316863813	0.146
cg18437039	II	37	19	1444202		0.46	0.01197	1.92207	0.29811549	0.443709555	-0.146
cg24723690	II	37	19	52213977		0.54	0.0014	2.85345	0.6295775	0.483691051	0.146
cg11254700	I	37	19	53561386		0.42	0.02991	1.52413	0.34481586	0.491159277	-0.146
cg09835408	II	37	20	31352399	DNMT3B;DNMT3B;	0.5	0.00432	2.36443	0.19089402	0.337180506	-0.146
cg00929635	I	37	20	44035918	DBNDD2;DBNDD2;I	0.5	0.00432	2.36443	0.3661033	0.512448811	-0.146
cg19067730	II	37	20	44518824	CTSA;NEURL2;CTSA	0.62	0.0001	3.98291	0.18758289	0.333776009	-0.146
cg15765398	II	37	21	46409994		0.42	0.02991	1.52413	0.50441735	0.358750241	0.146
cg07924575	II	37	22	26881231	HPS4;SRRD	0.5	0.00432	2.36443	0.67858856	0.824800814	-0.146
cg15702277	II	37	1	8477935	RERE;RERE;RERE	0.62	0.0001	3.98291	0.43555045	0.580933109	-0.145

cg07155422	II	37	1	12114402		0.5	0.00432	2.36443	0.7424752	0.597179289	0.145
cg23840027	II	37	1	29225533	EPB41;EPB41;EPB4	0.54	0.0014	2.85345	0.47453889	0.619474733	-0.145
cg13470063	II	37	1	43766206	TIE1	0.67	2.34E-05	4.63072	0.52573923	0.670861572	-0.145
cg15715337	II	37	1	85600447		0.46	0.01197	1.92207	0.55981321	0.70471004	-0.145
cg13531460	II	37	2	231790813	GPR55	0.46	0.01197	1.92207	0.74371302	0.598673563	0.145
cg13475822	II	37	3	9210116	SRGAP3;SRGAP3	0.5	0.00432	2.36443	0.65296829	0.507862344	0.145
cg16588163	I	37	3	190251464	IL1RAP;IL1RAP;IL1R	0.42	0.02991	1.52413	0.30291583	0.447527329	-0.145
cg18713687	II	37	3	195489789	MUC4;MUC4;MUC4	0.62	0.0001	3.98291	0.74336388	0.8887156	-0.145
cg13731523	I	37	4	3047190		0.58	0.00041	3.39184	0.60241319	0.747075934	-0.145
cg26562462	I	37	4	6929045	TBC1D14;TBC1D14	0.42	0.02991	1.52413	0.46588364	0.611042525	-0.145
cg22197936	II	37	5	76325203	AGGF1	0.5	0.00432	2.36443	0.67948354	0.824348375	-0.145
cg10787197	II	37	6	11779941	C6orf105;C6orf105	0.58	0.00041	3.39184	0.65006739	0.795558872	-0.145
cg23953820	I	37	6	30851051	DDR1;DDR1	0.54	0.0014	2.85345	0.49774793	0.352547655	0.145
cg14482998	II	37	6	111984370	FYN;FYN;FYN	0.58	0.00041	3.39184	0.41445904	0.558960699	-0.145
cg14030904	II	37	6	149806732	ZC3H12D	0.58	0.00041	3.39184	0.38829425	0.242885486	0.145
cg03634833	II	37	7	965534	ADAP1	0.62	0.0001	3.98291	0.35697302	0.502282858	-0.145
cg09363068	II	37	7	1004748	COX19	0.54	0.0014	2.85345	0.66828752	0.813315994	-0.145
cg04682977	II	37	7	1062711	C7orf50;MIR339;C7	0.46	0.01197	1.92207	0.50838519	0.363657442	0.145
cg05729490	II	37	7	27404187		0.5	0.00432	2.36443	0.56825146	0.713667791	-0.145
cg23723486	II	37	7	101361745		0.54	0.0014	2.85345	0.37533601	0.51993193	-0.145
cg08913523	II	37	8	126649807		0.42	0.02991	1.52413	0.28325651	0.42849171	-0.145
cg07752613	II	37	8	141554311	EIF2C2;EIF2C2	0.54	0.0014	2.85345	0.6789103	0.824366926	-0.145
cg21197594	II	37	9	140656981	EHMT1;FLJ40292;E	0.42	0.02991	1.52413	0.49253498	0.347192982	0.145
cg23863328	II	37	10	49879704		0.42	0.02991	1.52413	0.56914019	0.424546328	0.145
cg25268100	I	37	10	134457731	INPP5A	0.62	0.0001	3.98291	0.73164382	0.586548175	0.145
cg15484375	II	37	11	18287647	SAA1;SAA1	0.67	2.34E-05	4.63072	0.54533453	0.690531456	-0.145
cg27636058	II	37	11	57443308	ZDHHC5	0.5	0.00432	2.36443	0.83962109	0.694841333	0.145
cg09445967	II	37	11	60634850	ZP1	0.54	0.0014	2.85345	0.49493708	0.349474248	0.145
cg24755189	II	37	11	62475373	BSCL2;BSCL2;GNG3	0.46	0.01197	1.92207	0.74822867	0.602857445	0.145
cg21592954	II	37	11	126304039	KIRREL3;KIRREL3	0.5	0.00432	2.36443	0.36617303	0.511567639	-0.145
cg22158248	II	37	12	112204136	ALDH2	0.46	0.01197	1.92207	0.59831718	0.452893391	0.145
cg19445588	II	37	12	116904641		0.46	0.01197	1.92207	0.72461525	0.869202573	-0.145
cg10901569	II	37	12	121582875	P2RX7	0.46	0.01197	1.92207	0.60030278	0.744836517	-0.145

cg09931783	II	37	12	123469669	PITPNM2	0.58	0.00041	3.39184	0.52985774	0.675339413	-0.145
cg24598712	II	37	12	133072859	FBRSL1	0.58	0.00041	3.39184	0.65557222	0.800801564	-0.145
cg23489384	I	37	14	21199099		0.46	0.01293	1.88854	0.51151292	0.366941274	0.145
cg14389122	II	37	15	74945851	EDC3;EDC3;EDC3	0.5	0.00432	2.36443	0.57222321	0.427437671	0.145
cg17241841	II	37	15	89158993		0.46	0.01197	1.92207	0.73134552	0.586423393	0.145
cg10416668	II	37	16	27437730	IL21R;IL21R;IL21R	0.46	0.01197	1.92207	0.74706462	0.602103411	0.145
cg27463141	II	37	16	56689112		0.58	0.00041	3.39184	0.6680515	0.522733149	0.145
cg02961798	II	37	16	67687392	RLTPR	0.46	0.01197	1.92207	0.70437162	0.849657216	-0.145
cg07793033	II	37	16	85256423		0.42	0.02991	1.52413	0.57962008	0.724548197	-0.145
cg07548791	I	37	16	86012053		0.5	0.00432	2.36443	0.27451191	0.419295808	-0.145
cg05829375	II	37	16	86012085		0.5	0.00432	2.36443	0.33141848	0.476902651	-0.145
cg01591830	II	37	16	89666000		0.58	0.00041	3.39184	0.51113276	0.365684004	0.145
cg02723774	II	37	17	1378119	MYO1C;MYO1C;MY	0.46	0.01197	1.92207	0.6405817	0.49597169	0.145
cg07324245	II	37	17	75445905	SEPT9;SEPT9;SEPT9	0.58	0.00041	3.39184	0.68150274	0.536849558	0.145
cg25826463	I	37	19	3369820	NFIC;NFIC	0.5	0.00432	2.36443	0.42919898	0.574197121	-0.145
cg05898333	II	37	20	62549385	MIR941-1;DNAJC5;I	0.46	0.01197	1.92207	0.65961191	0.514292472	0.145
cg14606328	II	37	21	45177639	PDXK	0.67	2.34E-05	4.63072	0.69401361	0.83873266	-0.145
cg03733278	II	37	22	20760922	ZNF74;ZNF74	0.54	0.0014	2.85345	0.81527768	0.670067277	0.145
cg03382805	I	37	22	23905582		0.5	0.00432	2.36443	0.60656724	0.461761582	0.145
cg13253729	II	37	22	24032426	LOC91316;RGL4	0.62	0.0001	3.98291	0.64504036	0.790342636	-0.145
cg00488514	II	37	22	26877690	HPS4	0.5	0.00432	2.36443	0.49297206	0.638090261	-0.145
cg18396403	II	37	22	38615368	TMEM184B	0.46	0.01197	1.92207	0.32558377	0.470367779	-0.145
cg16068833	II	37	1	26644515	UBXN11;CD52	0.62	0.0001	3.98291	0.25748321	0.113566352	0.144
cg10208610	II	37	1	27326422	TRNP1	0.58	0.00041	3.39184	0.55109577	0.695199605	-0.144
cg15459165	II	37	1	31223850	LAPTM5	0.5	0.00432	2.36443	0.23684523	0.092701319	0.144
cg16356622	II	37	1	36351841	EIF2C1	0.42	0.02991	1.52413	0.4528225	0.597131409	-0.144
cg09856274	II	37	1	43304307	ERMAP;ERMAP	0.42	0.02991	1.52413	0.45079907	0.595166567	-0.144
cg00056754	II	37	1	167063958	DUSP27	0.5	0.00432	2.36443	0.70359555	0.847972777	-0.144
cg22014289	II	37	2	109952974	SH3RF3	0.42	0.02991	1.52413	0.58408253	0.439751853	0.144
cg25758828	II	37	2	113976143	PAX8;PAX8;PAX8;P/	0.54	0.0014	2.85345	0.7343332	0.878802296	-0.144
cg01796438	II	37	3	11312864	ATG7;ATG7;ATG7	0.54	0.0014	2.85345	0.66074784	0.804325417	-0.144
cg23202468	I	37	3	42705828	ZBTB47	0.42	0.02991	1.52413	0.46132112	0.317581466	0.144
cg03655330	II	37	3	50337494	HYAL1;HYAL1;HYAL	0.5	0.00432	2.36443	0.2785241	0.422795859	-0.144

cg05068866	II	37	3	66633374		0.5	0.00432	2.36443	0.40098095	0.257165134	0.144
cg20424311	II	37	4	141221527	SCOC	0.5	0.00432	2.36443	0.59526554	0.738955518	-0.144
cg16707952	II	37	6	30851632	DDR1;DDR1	0.58	0.00041	3.39184	0.66414357	0.520171354	0.144
cg16580935	I	37	6	33246149	B3GALT4	0.54	0.0014	2.85345	0.78612466	0.641688233	0.144
cg12682870	II	37	6	43766804		0.46	0.01197	1.92207	0.24927595	0.39351424	-0.144
cg02213045	II	37	6	43967803	C6orf223	0.46	0.01197	1.92207	0.83373766	0.689890212	0.144
cg15222091	II	37	6	167536069	CCR6;CCR6	0.42	0.02991	1.52413	0.46873247	0.324817704	0.144
cg19723715	II	37	7	1125817	GPER;C7orf50;C7or	0.46	0.01197	1.92207	0.79062053	0.646447514	0.144
cg20848988	II	37	7	36247779	EEPD1	0.62	0.0001	3.98291	0.20218285	0.345962031	-0.144
cg14714629	II	37	7	99495452	TRIM4;TRIM4	0.46	0.01197	1.92207	0.47801811	0.622280361	-0.144
cg12782180	I	37	7	127880932	LEP	0.46	0.01197	1.92207	0.50693669	0.650495462	-0.144
cg18734433	II	37	7	150086169	ZNF775	0.58	0.00041	3.39184	0.45560916	0.600079085	-0.144
cg10012512	I	37	7	157224041		0.42	0.02991	1.52413	0.8836255	0.739740881	0.144
cg11908155	II	37	8	11625764	NEIL2;NEIL2;NEIL2;	0.5	0.00432	2.36443	0.64639412	0.502498609	0.144
cg01854491	I	37	8	144815021	FAM83H	0.42	0.02991	1.52413	0.71938521	0.57541677	0.144
cg14294859	I	37	9	130862556	SLC25A25;SLC25A2	0.5	0.00432	2.36443	0.73139379	0.875491302	-0.144
cg14297444	II	37	9	130978697	DNM1;DNM1	0.5	0.00432	2.36443	0.71696215	0.572782114	0.144
cg27382409	II	37	10	47088062	PPYR1	0.62	0.0001	3.98291	0.61086478	0.467301761	0.144
cg24417845	II	37	10	70816008		0.46	0.01197	1.92207	0.34164716	0.485549151	-0.144
cg10521450	II	37	10	105439280	SH3PXD2A	0.5	0.00432	2.36443	0.46916489	0.324909506	0.144
cg02082273	II	37	11	10473038	AMPD3	0.5	0.00432	2.36443	0.48251437	0.338774901	0.144
cg04926244	II	37	12	24737251	C12orf67	0.42	0.02991	1.52413	0.53439507	0.678842509	-0.144
cg17772430	II	37	12	33052596		0.54	0.0014	2.85345	0.35794948	0.501965542	-0.144
cg21455901	II	37	13	21012516	CRYL1	0.71	4.57E-06	5.34042	0.48093665	0.625019029	-0.144
cg06470855	I	37	13	112997365		0.58	0.00041	3.39184	0.59947958	0.74320916	-0.144
cg21230021	II	37	14	101530770	MIR412;MIR410;M	0.46	0.01197	1.92207	0.78067897	0.63628305	0.144
cg02287710	I	37	14	102027660	DIO3;MIR1247	0.58	0.00041	3.39184	0.37687535	0.520710972	-0.144
cg18991417	II	37	14	104391679		0.62	0.0001	3.98291	0.58307024	0.727491908	-0.144
cg27155939	II	37	15	40347163		0.5	0.00432	2.36443	0.28649265	0.142460753	0.144
cg09803904	II	37	15	89939709	LOC254559	0.62	0.0001	3.98291	0.65688733	0.80063788	-0.144
cg08166214	II	37	16	1027357		0.46	0.01197	1.92207	0.67875883	0.534414104	0.144
cg02513379	II	37	16	27414281	IL21R;IL21R	0.71	4.57E-06	5.34042	0.28461764	0.140765234	0.144
cg04767841	II	37	16	85656678	KIAA0182;KIAA018[illegible]	0.54	0.0014	2.85345	0.44123147	0.585117081	-0.144

cg07308381	II	37	17	6917370	RNASEK;C17orf49;C	0.46	0.01197	1.92207	0.77290806	0.628839487	0.144
cg05533539	II	37	17	44104521	MAPT;MAPT;MAPT	0.42	0.02991	1.52413	0.34879102	0.492803613	-0.144
cg19026231	II	37	17	73761710	GALK1	0.46	0.01197	1.92207	0.7942636	0.650338359	0.144
cg18437480	II	37	17	76130305	TMC8	0.58	0.00041	3.39184	0.35786544	0.213820469	0.144
cg22233020	II	37	19	1926143		0.46	0.01197	1.92207	0.64074142	0.49701915	0.144
cg19907483	I	37	19	6040198	RFX2;RFX2	0.46	0.01197	1.92207	0.81919193	0.675099068	0.144
cg11588903	II	37	19	17208493	MYO9B;MYO9B	0.67	2.34E-05	4.63072	0.68953036	0.833256234	-0.144
cg07569288	I	37	19	50002551	RPS11	0.42	0.02991	1.52413	0.7834522	0.639062669	0.144
cg18112953	II	37	20	47448545		0.5	0.00432	2.36443	0.35173641	0.495397626	-0.144
cg15815827	II	37	20	61546407	DIDO1;DIDO1;DIDC	0.54	0.0014	2.85345	0.2974528	0.441808102	-0.144
cg00805193	II	37	20	62609362	SAMD10	0.46	0.01197	1.92207	0.39175156	0.535774466	-0.144
cg17972162	II	37	22	39496387	APOBEC3H;APOBEC	0.5	0.00432	2.36443	0.65442295	0.509951098	0.144
cg24878051	II	37	1	3305115	PRDM16;PRDM16	0.54	0.0014	2.85345	0.76898282	0.625680343	0.143
cg24003955	II	37	1	9912508	CTNNBIP1;CTNNBIF	0.5	0.00432	2.36443	0.61823414	0.76105055	-0.143
cg02053477	II	37	1	22615900		0.5	0.00432	2.36443	0.7471835	0.603734624	0.143
cg21805788	II	37	1	109134372	FAM102B	0.46	0.01197	1.92207	0.61560628	0.758627991	-0.143
cg20169988	II	37	1	153330776	S100A9	0.54	0.0014	2.85345	0.26369506	0.406971263	-0.143
cg06470727	II	37	2	26723062	OTOF	0.46	0.01197	1.92207	0.54260486	0.399930054	0.143
cg15736127	II	37	2	157292127	GPD2;GPD2;GPD2	0.46	0.01197	1.92207	0.24473931	0.387529881	-0.143
cg15476790	II	37	2	218786102	TNS1	0.58	0.00041	3.39184	0.76881292	0.62628909	0.143
cg15033269	II	37	3	42631489	SS18L2	0.46	0.01197	1.92207	0.63700235	0.779575838	-0.143
cg15157945	II	37	3	47023603	NBEAL2	0.67	2.34E-05	4.63072	0.35713368	0.500630686	-0.143
cg19463199	II	37	3	197401858	MIR922;KIAA0226;I	0.42	0.02991	1.52413	0.63440914	0.491466813	0.143
cg12044923	II	37	4	5207312	STK32B	0.5	0.00432	2.36443	0.6850271	0.542196564	0.143
cg26560699	II	37	5	171918659		0.46	0.01197	1.92207	0.75409453	0.611413554	0.143
cg01157650	II	37	5	177809164	COL23A1	0.54	0.0014	2.85345	0.64848644	0.505607934	0.143
cg11191555	II	37	5	177895442	COL23A1	0.67	2.34E-05	4.63072	0.67042428	0.81338862	-0.143
cg20986887	II	37	6	28887284	TRIM27	0.5	0.00432	2.36443	0.4377951	0.580303106	-0.143
cg15708909	II	37	6	32487314	HLA-DRB5	0.46	0.01197	1.92207	0.52845759	0.671379504	-0.143
cg02835756	II	37	6	41442369		0.58	0.00041	3.39184	0.72888677	0.585482831	0.143
cg06840743	II	37	7	155150876		0.42	0.02991	1.52413	0.56533403	0.708467652	-0.143
cg21919729	I	37	8	11719367	CTSB;CTSB;CTSB;CT	0.54	0.0014	2.85345	0.77765192	0.920363501	-0.143
cg14016344	I	37	9	35616726	CD72	0.54	0.0014	2.85345	0.49902318	0.356509115	0.143

cg13672736	I	37	9	135114066	NTNG2	0.42	0.02991	1.52413	0.90738522	0.764537133	0.143
cg12037550	II	37	10	1705054	ADARB2	0.5	0.00432	2.36443	0.61570955	0.473004516	0.143
cg24376955	II	37	10	43637359	CSGALNACT2	0.5	0.00432	2.36443	0.65996442	0.803133946	-0.143
cg04641860	II	37	10	43891547	HNRNPF;HNRNPF;H	0.5	0.00432	2.36443	0.67867807	0.535659714	0.143
cg11095743	II	37	10	49815243		0.58	0.00041	3.39184	0.34701761	0.490266027	-0.143
cg14447240	II	37	10	134823349		0.58	0.00041	3.39184	0.65852335	0.801592261	-0.143
cg00509007	II	37	11	1781557	HCCA2;CTSD	0.54	0.0014	2.85345	0.71857677	0.861570506	-0.143
cg14600987	II	37	11	1952678	TNNT3;TNNT3;TNN	0.5	0.00432	2.36443	0.28131968	0.424079934	-0.143
cg11342234	II	37	11	2710838	KCNQ1;KCNQ1OT1;	0.46	0.01197	1.92207	0.50741152	0.364727401	0.143
cg13859639	II	37	11	2846716	KCNQ1;KCNQ1	0.54	0.0014	2.85345	0.42638394	0.569208207	-0.143
cg06433350	II	37	11	3115015	OSBPL5;OSBPL5;OS	0.5	0.00432	2.36443	0.43236754	0.575723013	-0.143
cg12535280	I	37	11	12399498	PARVA	0.46	0.01197	1.92207	0.09677733	0.240090792	-0.143
cg07631435	II	37	11	114043903	ZBTB16;ZBTB16	0.54	0.0014	2.85345	0.5160576	0.372927687	0.143
cg24951335	II	37	12	1609463	LOC100292680	0.5	0.00432	2.36443	0.51052568	0.653380477	-0.143
cg18071202	II	37	12	1609470	LOC100292680	0.58	0.00041	3.39184	0.51026963	0.653256094	-0.143
cg19345219	II	37	12	6747405	ACRBP	0.46	0.01197	1.92207	0.58720155	0.443855008	0.143
cg04381888	II	37	12	48195549	HDAC7;HDAC7	0.54	0.0014	2.85345	0.28238234	0.42573071	-0.143
cg12800105	II	37	12	121972412	KDM2B;KDM2B	0.67	2.34E-05	4.63072	0.79853218	0.655774831	0.143
cg10097313	II	37	14	25591076		0.67	2.34E-05	4.63072	0.68771017	0.830472578	-0.143
cg18655522	I	37	14	69424756	ACTN1;ACTN1;ACTI	0.46	0.01197	1.92207	0.68208126	0.824907182	-0.143
cg07163845	II	37	14	106071651		0.42	0.02991	1.52413	0.36412326	0.50694471	-0.143
cg20908276	I	37	14	106134542		0.5	0.00432	2.36443	0.72838393	0.585311049	0.143
cg04055913	II	37	15	44173036	FRMD5	0.5	0.00432	2.36443	0.580363	0.437029819	0.143
cg26510178	I	37	16	728470		0.42	0.02991	1.52413	0.83326708	0.69018199	0.143
cg02878222	II	37	16	5117565	C16orf89;C16orf89	0.54	0.0014	2.85345	0.81307049	0.670181298	0.143
cg06897661	I	37	16	50322074	ADCY7;ADCY7	0.58	0.00041	3.39184	0.73263108	0.875975204	-0.143
cg26946015	II	37	16	66178419		0.54	0.0014	2.85345	0.80574943	0.662823952	0.143
cg00036723	II	37	17	32688878	CCL1	0.5	0.00432	2.36443	0.39279013	0.249718802	0.143
cg08329754	II	37	17	78797015	RPTOR;RPTOR	0.42	0.02991	1.52413	0.71643651	0.859428557	-0.143
cg27316369	II	37	17	79799376		0.54	0.0014	2.85345	0.62413585	0.766707507	-0.143
cg10356204	II	37	19	2255744	JSRP1	0.42	0.02991	1.52413	0.46176853	0.3192465	0.143
cg05343021	II	37	19	10773698	ILF3;ILF3;ILF3;ILF3;I	0.54	0.0014	2.85345	0.47788778	0.335057397	0.143
cg22676000	II	37	19	42259081	CEACAM6	0.46	0.01197	1.92207	0.68733538	0.54454869	0.143

cg18687314	II	37	20	43733672		0.58	0.00041	3.39184	0.74198828	0.59870641	0.143
cg21723559	II	37	20	44048174	PIGT	0.46	0.01197	1.92207	0.78265505	0.640145799	0.143
cg18468842	II	37	20	45313109	TP53RK;SLC13A3	0.42	0.02991	1.52413	0.49895509	0.64192835	-0.143
cg06893362	II	37	20	47897124	C20orf199;C20orf1	0.46	0.01197	1.92207	0.73472968	0.59197319	0.143
cg07900444	II	37	21	46419208	NCRNA00162	0.5	0.00432	2.36443	0.34788742	0.20489088	0.143
cg08474826	II	37	1	1099630		0.42	0.02991	1.52413	0.58793607	0.446153689	0.142
cg22862319	II	37	1	3289994	PRDM16;PRDM16	0.5	0.00432	2.36443	0.64219328	0.499987207	0.142
cg20305595	II	37	1	9293833	H6PD	0.58	0.00041	3.39184	0.5049975	0.646520871	-0.142
cg21385052	II	37	1	149903249	MTMR11;MTMR11	0.58	0.00041	3.39184	0.76602174	0.90771596	-0.142
cg06355720	II	37	1	153333350	S100A9	0.5	0.00432	2.36443	0.5113437	0.653642631	-0.142
cg13765621	II	37	1	158149228	CD1D	0.46	0.01197	1.92207	0.28701694	0.429056379	-0.142
cg17250262	II	37	2	30457110	LBH	0.62	0.0001	3.98291	0.46943592	0.327409328	0.142
cg09154591	II	37	2	36923828	VIT	0.42	0.02991	1.52413	0.65775403	0.799597417	-0.142
cg02541444	II	37	2	37996000		0.67	2.34E-05	4.63072	0.58103719	0.722989228	-0.142
cg26775087	I	37	3	15382808	SH3BP5;SH3BP5	0.5	0.00432	2.36443	0.79664095	0.654817099	0.142
cg03461641	II	37	4	26454190		0.42	0.02991	1.52413	0.88613833	0.743757348	0.142
cg07516556	II	37	5	1475096	LPCAT1	0.42	0.02991	1.52413	0.68094052	0.823391925	-0.142
cg05134426	II	37	5	124080479	ZNF608	0.5	0.00432	2.36443	0.7374601	0.595742812	0.142
cg11010552	II	37	5	131338478	ACSL6;ACSL6	0.62	0.0001	3.98291	0.51378196	0.655386963	-0.142
cg05201300	II	37	5	172443740	ATP6V0E1	0.5	0.00432	2.36443	0.6109755	0.752728007	-0.142
cg10500084	II	37	5	174172044		0.5	0.00432	2.36443	0.73102693	0.589473491	0.142
cg02979839	II	37	5	176957063	FAM193B;FAM193I	0.58	0.00041	3.39184	0.72025726	0.862030481	-0.142
cg14622996	II	37	6	32109801		0.54	0.0014	2.85345	0.37497364	0.517397788	-0.142
cg00115458	I	37	6	33141305	COL11A2;COL11A2;	0.54	0.0014	2.85345	0.34776314	0.205272313	0.142
cg01063280	II	37	6	108167599		0.54	0.0014	2.85345	0.64442427	0.786137887	-0.142
cg21143896	I	37	7	2802374	GNA12	0.67	2.34E-05	4.63072	0.63058792	0.772451763	-0.142
cg12091331	II	37	8	42065314	PLAT;PLAT	0.58	0.00041	3.39184	0.50432874	0.646023532	-0.142
cg19991046	II	37	8	142183677	DENND3	0.5	0.00432	2.36443	0.44656372	0.588095942	-0.142
cg08267399	II	37	8	142349160	LOC731779	0.58	0.00041	3.39184	0.53886396	0.396929257	0.142
cg13580827	II	37	9	72081155	APBA1	0.54	0.0014	2.85345	0.72245447	0.580601124	0.142
cg13931640	I	37	9	137277819	RXRA	0.5	0.00432	2.36443	0.52216759	0.664297387	-0.142
cg00972313	I	37	10	1531243	ADARB2	0.54	0.0014	2.85345	0.57553422	0.433912063	0.142
cg27099293	II	37	10	71895572		0.54	0.0014	2.85345	0.70633919	0.848318202	-0.142

cg18614702	II	37	10	72215990		0.58	0.00041	3.39184	0.57016233	0.712503777	-0.142
cg24437408	I	37	10	134661502		0.71	4.57E-06	5.34042	0.391211	0.248957554	0.142
cg25327296	II	37	11	44927093	TSPAN18;TSPAN18	0.5	0.00432	2.36443	0.69013997	0.547855466	0.142
cg02713832	II	37	11	60772559	CD6	0.46	0.01197	1.92207	0.60761506	0.465261461	0.142
cg08450021	II	37	11	111101517		0.5	0.00432	2.36443	0.50986384	0.36825072	0.142
cg22434506	I	37	12	6657818	IFFO1;IFFO1;IFFO1	0.62	0.0001	3.98291	0.80560389	0.947948911	-0.142
cg04061752	II	37	12	19350090	PLEKHA5;PLEKHA5	0.42	0.02991	1.52413	0.2620776	0.403956405	-0.142
cg23378033	II	37	12	65672031	MSRB3;MSRB3	0.5	0.00432	2.36443	0.18812808	0.329724622	-0.142
cg14486477	II	37	12	117256672	RNFT2;RNFT2	0.62	0.0001	3.98291	0.34696511	0.205026813	0.142
cg12421110	II	37	13	42035731	C13orf15	0.62	0.0001	3.98291	0.77606717	0.634403067	0.142
cg21836117	II	37	14	73012519	RGS6	0.46	0.01197	1.92207	0.52522031	0.382984529	0.142
cg13614617	II	37	15	65656635	IGDCC3	0.54	0.0014	2.85345	0.75622018	0.614560934	0.142
cg05412396	II	37	15	76631569	ISL2	0.46	0.01197	1.92207	0.27937371	0.421132501	-0.142
cg19293468	I	37	17	1973400	SMG6;SMG6	0.42	0.02991	1.52413	0.72165195	0.579332257	0.142
cg23399011	II	37	17	10742274	PIRT	0.5	0.00432	2.36443	0.6989663	0.55701443	0.142
cg16047279	I	37	17	38717242	CCR7	0.58	0.00041	3.39184	0.20626715	0.0645367	0.142
cg19003337	I	37	17	39780836	KRT17;KRT17	0.42	0.02991	1.52413	0.40153157	0.543374169	-0.142
cg11669285	II	37	17	40558061	PTRF	0.71	4.57E-06	5.34042	0.6370215	0.778535841	-0.142
cg08817507	I	37	17	79924068		0.46	0.01197	1.92207	0.6730531	0.530988145	0.142
cg27400313	I	37	18	74824280	MBP;MBP	0.46	0.01197	1.92207	0.6065854	0.74849436	-0.142
cg08432509	II	37	20	62317768	RTEL1;RTEL1	0.58	0.00041	3.39184	0.59364392	0.735537978	-0.142
cg00334274	II	37	22	30474915		0.5	0.00432	2.36443	0.67927386	0.537104752	0.142
cg24296621	II	37	1	1444995		0.75	7.61E-07	6.11857	0.33340302	0.47462278	-0.141
cg22226775	II	37	1	3008033	PRDM16;PRDM16	0.46	0.01197	1.92207	0.70533412	0.564674043	0.141
cg08129092	II	37	1	153746211	INTS3	0.46	0.01197	1.92207	0.26499188	0.405955553	-0.141
cg12617080	II	37	1	156509844	IQGAP3	0.54	0.0014	2.85345	0.28012699	0.421101344	-0.141
cg17100176	II	37	1	207096358	FAIM3;FAIM3;FAIM	0.62	0.0001	3.98291	0.46427869	0.323464945	0.141
cg06966462	II	37	2	9612807	CPSF3	0.67	2.34E-05	4.63072	0.74853084	0.607834556	0.141
cg02431562	II	37	2	96809970	DUSP2	0.5	0.00432	2.36443	0.64203283	0.50065111	0.141
cg24777399	II	37	2	109855574	SH3RF3	0.46	0.01197	1.92207	0.30893941	0.449856307	-0.141
cg02930866	II	37	3	15482580	EAF1	0.46	0.01197	1.92207	0.22769825	0.369146087	-0.141
cg20540428	II	37	3	73045686	PPP4R2	0.46	0.01197	1.92207	0.42283454	0.563504028	-0.141
cg25574175	II	37	3	119500929	NR1I2;NR1I2;NR1I2	0.5	0.00432	2.36443	0.7284315	0.869645092	-0.141

cg16322565	II	37	3	119526221	NR1I2;NR1I2;NR1I2	0.54	0.0014	2.85345	0.56615219	0.707048345	-0.141
cg12146673	I	37	3	187387555	SST	0.46	0.01197	1.92207	0.22717436	0.367931005	-0.141
cg25646029	II	37	5	139584772	C5orf32	0.67	2.34E-05	4.63072	0.65703518	0.798517195	-0.141
cg21242144	II	37	5	148808487	LOC728264;MIR143	0.58	0.00041	3.39184	0.362593	0.504073237	-0.141
cg08537847	I	37	5	148810203	MIR145;LOC728264	0.5	0.00432	2.36443	0.7767525	0.917381292	-0.141
cg01676996	I	37	6	30619167	C6orf136;C6orf136	0.46	0.01197	1.92207	0.72989059	0.589105248	0.141
cg16176069	II	37	7	1062784	C7orf50;MIR339;C7	0.42	0.02991	1.52413	0.50676474	0.365522331	0.141
cg23593387	II	37	7	1864317	MAD1L1;MAD1L1;M	0.5	0.00432	2.36443	0.71510912	0.574083742	0.141
cg15896696	I	37	7	2059921	MAD1L1;MAD1L1;M	0.42	0.02991	1.52413	0.17778351	0.318749985	-0.141
cg26369382	II	37	7	100240341	TFR2	0.42	0.02991	1.52413	0.73367818	0.593110488	0.141
cg07886914	II	37	8	28198639	PNOC	0.5	0.00432	2.36443	0.77871976	0.637338757	0.141
cg07205823	II	37	8	49229620		0.54	0.0014	2.85345	0.68155444	0.540349011	0.141
cg17207736	II	37	8	142237307	SLC45A4	0.54	0.0014	2.85345	0.51872212	0.659317537	-0.141
cg14604066	I	37	9	139590572		0.46	0.01197	1.92207	0.62895745	0.487674884	0.141
cg11189134	II	37	10	1531498	ADARB2	0.5	0.00432	2.36443	0.50467022	0.363704458	0.141
cg25368482	I	37	10	47653939		0.5	0.00432	2.36443	0.08542207	0.225996951	-0.141
cg17389077	II	37	10	128810904	DOCK1	0.5	0.00432	2.36443	0.39315047	0.534077469	-0.141
cg09983885	II	37	11	4415245	TRIM21	0.58	0.00041	3.39184	0.3566267	0.497590991	-0.141
cg08202743	II	37	11	34182570	ABTB2	0.42	0.02991	1.52413	0.68315737	0.54263756	0.141
cg05481452	II	37	11	61717684	BEST1;BEST1;BEST1	0.62	0.0001	3.98291	0.7050347	0.563869087	0.141
cg23695131	I	37	11	94642928		0.62	0.0001	3.98291	0.69371451	0.834914765	-0.141
cg04362096	II	37	11	123016010	ASAM	0.46	0.01197	1.92207	0.39308271	0.533882787	-0.141
cg13298466	II	37	12	6658164	IFFO1;IFFO1;IFFO1;	0.5	0.00432	2.36443	0.65577567	0.797271637	-0.141
cg02564061	II	37	12	49998963	FAM186B;FAM186I	0.5	0.00432	2.36443	0.47213683	0.613111819	-0.141
cg24260327	II	37	12	125023590		0.62	0.0001	3.98291	0.58742511	0.728831281	-0.141
cg01663970	II	37	12	125299686	SCARB1;SCARB1	0.67	2.34E-05	4.63072	0.75914674	0.900577052	-0.141
cg02088785	II	37	13	99223336	STK24	0.54	0.0014	2.85345	0.55284915	0.411653974	0.141
cg10197238	II	37	14	21269744	RNASE1;RNASE1;RN	0.58	0.00041	3.39184	0.3106722	0.4521467	-0.141
cg23092040	II	37	14	65068504		0.67	2.34E-05	4.63072	0.45381411	0.594483725	-0.141
cg01498829	II	37	14	100608798	EVL	0.58	0.00041	3.39184	0.59369866	0.734289665	-0.141
cg05873568	II	37	14	105155145	INF2;INF2;INF2	0.54	0.0014	2.85345	0.23806857	0.379018477	-0.141
cg17383853	II	37	15	63166381		0.5	0.00432	2.36443	0.7086558	0.56717242	0.141
cg22513955	II	37	17	1665123	SERPINF1	0.46	0.01197	1.92207	0.52103579	0.662517367	-0.141

cg01346501	II	37	17	38254714	NR1D1	0.54	0.0014	2.85345	0.29962757	0.44048134	-0.141
cg07787614	II	37	17	76037364	TNRC6C;TNRC6C	0.42	0.02991	1.52413	0.434284	0.575241094	-0.141
cg23508887	II	37	18	13502053	C18orf1;C18orf1	0.5	0.00432	2.36443	0.81250704	0.671820481	0.141
cg25389087	II	37	18	74824413	MBP;MBP	0.58	0.00041	3.39184	0.51445871	0.655216015	-0.141
cg25960854	II	37	19	1160184	SBNO2	0.58	0.00041	3.39184	0.5874781	0.728449318	-0.141
cg21062760	II	37	19	36205574	ZBTB32	0.58	0.00041	3.39184	0.63253154	0.491445669	0.141
cg06484123	II	37	19	53107200		0.67	2.34E-05	4.63072	0.7613705	0.902143717	-0.141
cg24368848	I	37	19	58545160	ZSCAN1	0.58	0.00041	3.39184	0.07066923	0.211366273	-0.141
cg20177522	II	37	20	62410437	ZBTB46	0.5	0.00432	2.36443	0.62320581	0.764352301	-0.141
cg15683970	II	37	21	35747081	FAM165B	0.54	0.0014	2.85345	0.32876183	0.469971943	-0.141
cg10142237	II	37	22	38714395	CSNK1E;CSNK1E	0.5	0.00432	2.36443	0.38907559	0.530098475	-0.141
cg25201541	II	37	1	1444416		0.75	7.61E-07	6.11857	0.46995506	0.610340059	-0.14
cg07813851	II	37	1	7466976	CAMTA1	0.5	0.00432	2.36443	0.83253951	0.692476256	0.14
cg08922729	II	37	1	21913557		0.58	0.00041	3.39184	0.49011584	0.630137612	-0.14
cg07191152	II	37	1	25061987		0.46	0.01197	1.92207	0.64870336	0.509150411	0.14
cg03654504	II	37	1	37495105	GRIK3	0.46	0.01197	1.92207	0.47052727	0.330182885	0.14
cg15925478	II	37	1	94081080	BCAR3	0.5	0.00432	2.36443	0.60637656	0.466575458	0.14
cg24406771	II	37	1	229002047		0.46	0.01197	1.92207	0.75670416	0.616381504	0.14
cg22222999	II	37	1	234894712		0.46	0.01197	1.92207	0.39458696	0.53440611	-0.14
cg23859635	II	37	2	42795262	MTA3	0.42	0.02991	1.52413	0.65677048	0.517191265	0.14
cg19805943	II	37	2	85933069		0.54	0.0014	2.85345	0.70825134	0.848185172	-0.14
cg19043574	II	37	2	99439533	C2orf55	0.42	0.02991	1.52413	0.13632755	0.275928093	-0.14
cg05870586	II	37	2	240196769	HDAC4	0.46	0.01197	1.92207	0.41121364	0.550980595	-0.14
cg18861762	II	37	2	242127852	ANO7;ANO7	0.46	0.01197	1.92207	0.69886357	0.558926574	0.14
cg27275023	II	37	3	107676222		0.62	0.0001	3.98291	0.74128477	0.881053139	-0.14
cg07262457	II	37	3	128777371		0.5	0.00432	2.36443	0.48898046	0.348884447	0.14
cg14313576	II	37	3	134339988	KY	0.5	0.00432	2.36443	0.76649978	0.626825694	0.14
cg01135648	II	37	4	41983611	DCAF4L1	0.62	0.0001	3.98291	0.73755559	0.877270045	-0.14
cg24020152	II	37	4	154419554	KIAA0922;KIAA092	0.42	0.02991	1.52413	0.79601829	0.65609222	0.14
cg10902033	II	37	5	471171	LOC25845	0.54	0.0014	2.85345	0.64662845	0.506963263	0.14
cg11042320	II	37	5	149534497	PDGFRB	0.42	0.02991	1.52413	0.60702736	0.467051365	0.14
cg11334730	II	37	6	6750455		0.54	0.0014	2.85345	0.40894291	0.549154801	-0.14
cg06315208	II	37	6	30684406	MDC1	0.42	0.02991	1.52413	0.72180437	0.581569244	0.14

cg17152981	II	37	6	110299835	GPR6	0.54	0.0014	2.85345	0.32079311	0.460979082	-0.14
cg09865593	II	37	7	645796	PRKAR1B;PRKAR1B	0.46	0.01197	1.92207	0.66654285	0.807037216	-0.14
cg00749672	II	37	7	26437192		0.42	0.02991	1.52413	0.54552787	0.685033311	-0.14
cg08641579	I	37	7	79083447	MAGI2	0.46	0.01197	1.92207	0.11663312	0.256507278	-0.14
cg17701146	I	37	7	157387361	PTPRN2;PTPRN2;PT	0.5	0.00432	2.36443	0.78285241	0.643293899	0.14
cg09050832	II	37	8	1365175		0.46	0.01197	1.92207	0.55237412	0.692361303	-0.14
cg06895124	II	37	8	141588750	EIF2C2;EIF2C2	0.58	0.00041	3.39184	0.6670696	0.807420051	-0.14
cg04124606	I	37	8	142095021		0.5	0.00496	2.30474	0.29261184	0.432116386	-0.14
cg25392060	II	37	8	142297121		0.46	0.01197	1.92207	0.41968216	0.559632095	-0.14
cg06894628	I	37	8	143822543	SLURP1	0.71	4.57E-06	5.34042	0.66270385	0.523156386	0.14
cg14221252	II	37	9	139589413		0.58	0.00041	3.39184	0.64123842	0.781311252	-0.14
cg00932063	II	37	10	126234138	LHPP;LHPP	0.62	0.0001	3.98291	0.46890832	0.32906994	0.14
cg07918545	II	37	10	129535669	FOXI2	0.58	0.00041	3.39184	0.12588123	0.266309654	-0.14
cg03402926	II	37	11	27340767		0.58	0.00041	3.39184	0.69956179	0.559414784	0.14
cg01240599	I	37	11	67418045	ACY3	0.46	0.01197	1.92207	0.25727535	0.117033	0.14
cg15104126	II	37	12	6150167	VWF	0.58	0.00041	3.39184	0.67201866	0.811733288	-0.14
cg16642299	II	37	12	7071383	MIR200C	0.46	0.01197	1.92207	0.24736473	0.386936269	-0.14
cg14872454	II	37	12	54685301		0.54	0.0014	2.85345	0.66617601	0.806423091	-0.14
cg02580045	II	37	12	110449223	ANKRD13A	0.42	0.02991	1.52413	0.73791072	0.597986201	0.14
cg07744430	II	37	12	132345502		0.54	0.0014	2.85345	0.45863944	0.599010981	-0.14
cg27506442	II	37	13	30948716	LOC100188949	0.54	0.0014	2.85345	0.20245202	0.062639776	0.14
cg14214797	II	37	14	91860613	CCDC88C	0.5	0.00432	2.36443	0.71623107	0.576566167	0.14
cg26188131	I	37	14	106095796		0.71	4.57E-06	5.34042	0.86568525	0.72549644	0.14
cg24184689	II	37	15	74723271	SEMA7A;SEMA7A;S	0.46	0.01197	1.92207	0.67364328	0.53395122	0.14
cg03905718	II	37	16	4289417	SRL	0.58	0.00041	3.39184	0.52136695	0.661341791	-0.14
cg18219562	II	37	17	41773643		0.54	0.0014	2.85345	0.66118759	0.801355874	-0.14
cg20433386	II	37	17	48702158	CACNA1G;CACNA1(	0.54	0.0014	2.85345	0.37888106	0.519128792	-0.14
cg08314949	II	37	17	78851213	RPTOR;RPTOR	0.46	0.01197	1.92207	0.51512381	0.375157882	0.14
cg03624195	I	37	19	14090310	RFX1	0.54	0.0014	2.85345	0.28867105	0.428370431	-0.14
cg00540464	II	37	19	16655751		0.54	0.0014	2.85345	0.83842455	0.698912809	0.14
cg07418126	II	37	19	49838101	CD37;CD37	0.5	0.00432	2.36443	0.60758721	0.467716908	0.14
cg01966791	I	37	20	62572875	MIR1914;UCKL1	0.42	0.02991	1.52413	0.33540757	0.475460251	-0.14
cg04723534	II	37	21	43373136	C2CD2	0.54	0.0014	2.85345	0.26995185	0.410235767	-0.14

cg06501716	II	37	22	19436948	C22orf39;C22orf39	0.42	0.02991	1.52413	0.69761337	0.557345456	0.14
cg05580073	II	37	22	24803248	CYTSA;CYTSA	0.5	0.00432	2.36443	0.30592441	0.445492702	-0.14
cg10096929	I	37	1	156261403	TMEM79;TMEM79	0.42	0.02991	1.52413	0.35935301	0.497888802	-0.139
cg00152117	II	37	1	156928845	ARHGEF11;ARHGEF	0.5	0.00432	2.36443	0.67242631	0.533168415	0.139
cg21400549	II	37	1	212588848	TMEM206	0.42	0.02991	1.52413	0.33983497	0.479000479	-0.139
cg22350835	I	37	2	1868857	MYT1L	0.42	0.02991	1.52413	0.41341034	0.274811968	0.139
cg22495058	II	37	2	11752373	GREB1	0.54	0.0014	2.85345	0.22664763	0.366014851	-0.139
cg27426835	II	37	2	19963298		0.5	0.00432	2.36443	0.60401879	0.464925589	0.139
cg15440661	II	37	2	43395871		0.42	0.02991	1.52413	0.50309873	0.642455259	-0.139
cg20460227	II	37	2	120452632		0.5	0.00432	2.36443	0.50244546	0.641196092	-0.139
cg21214743	II	37	2	130481001		0.58	0.00041	3.39184	0.79567234	0.656277673	0.139
cg00786685	II	37	2	218842585		0.5	0.00432	2.36443	0.71153048	0.572421415	0.139
cg07071449	II	37	2	238777806	RAMP1	0.42	0.02991	1.52413	0.80530535	0.666433472	0.139
cg21861151	II	37	2	239478365		0.54	0.0014	2.85345	0.49324505	0.63204928	-0.139
cg24231380	I	37	2	242813914	C2orf85	0.5	0.00432	2.36443	0.70271703	0.563378583	0.139
cg21442998	I	37	3	11597936	VGLL4;ATG7;VGLL4	0.54	0.0014	2.85345	0.5550958	0.694391041	-0.139
cg11902408	II	37	3	14859299	FGD5	0.5	0.00432	2.36443	0.75278201	0.614260798	0.139
cg12424624	II	37	3	46718941	ALS2CL;ALS2CL	0.67	2.34E-05	4.63072	0.72302493	0.86245545	-0.139
cg04208434	II	37	3	129513427	TMCC1;TMCC1	0.5	0.00432	2.36443	0.14225517	0.281208529	-0.139
cg05827190	II	37	4	681440	MFSD7	0.62	0.0001	3.98291	0.60139402	0.740442631	-0.139
cg19277389	II	37	4	7802545	AFAP1;AFAP1	0.46	0.01197	1.92207	0.79626706	0.656970315	0.139
cg05497253	II	37	5	77830465	LHFPL2	0.5	0.00432	2.36443	0.6869429	0.826315429	-0.139
cg13667243	I	37	5	157001561	ADAM19	0.67	2.34E-05	4.63072	0.53684295	0.397469441	0.139
cg11092486	II	37	6	5087604		0.42	0.02991	1.52413	0.548107	0.686786604	-0.139
cg16867657	I	37	6	11044877	ELOVL2	0.5	0.00432	2.36443	0.5966626	0.735194366	-0.139
cg08421910	II	37	6	11771840	C6orf105;C6orf105	0.46	0.01197	1.92207	0.7401121	0.879377459	-0.139
cg08766762	II	37	6	37521698		0.67	2.34E-05	4.63072	0.80693274	0.667580058	0.139
cg27184438	II	37	7	4690819		0.42	0.02991	1.52413	0.80548047	0.666903695	0.139
cg07103517	II	37	7	50348485	IKZF1	0.54	0.0014	2.85345	0.61364016	0.474921378	0.139
cg16548840	II	37	7	56149441	PHKG1	0.58	0.00041	3.39184	0.67389261	0.812854074	-0.139
cg14080050	II	37	9	33159025	B4GALT1	0.54	0.0014	2.85345	0.26147831	0.400412088	-0.139
cg13717350	II	37	9	136566991	SARDH;SARDH	0.42	0.02991	1.52413	0.50574425	0.366359991	0.139
cg23459363	II	37	10	115033723		0.5	0.00432	2.36443	0.48802587	0.627178089	-0.139

cg20345915	I	37	10	121296042	RGS10;RGS10;RGS1	0.58	0.00041	3.39184	0.70430056	0.843188167	-0.139
cg24181174	II	37	10	123900861	TACC2;TACC2	0.58	0.00041	3.39184	0.40623735	0.545362192	-0.139
cg26388730	II	37	11	64405764	NRXN2;NRXN2;NRX	0.5	0.00432	2.36443	0.47320989	0.334135493	0.139
cg09936933	II	37	11	72469551	STARD10	0.67	2.34E-05	4.63072	0.59009417	0.729375672	-0.139
cg13298528	II	37	11	118763863	CXCR5;CXCR5	0.67	2.34E-05	4.63072	0.6770038	0.537512644	0.139
cg14834893	II	37	12	4398032	CCND2	0.5	0.00432	2.36443	0.22309219	0.361851341	-0.139
cg17955329	II	37	12	113528764	DTX1	0.46	0.01197	1.92207	0.77869193	0.639276133	0.139
cg01687878	II	37	12	123616817		0.42	0.02991	1.52413	0.41426328	0.275495904	0.139
cg13750802	II	37	13	41055185	LOC646982;LOC646	0.46	0.01197	1.92207	0.73888327	0.59982676	0.139
cg16696317	I	37	14	96180406	TCL1A;TCL1A;TCL1A	0.67	2.34E-05	4.63072	0.36398464	0.225378868	0.139
cg07438412	II	37	14	105852272	PACS2;PACS2	0.54	0.0014	2.85345	0.5488573	0.687416818	-0.139
cg24663971	I	37	14	106410668		0.46	0.01197	1.92207	0.79557259	0.656122109	0.139
cg26987928	II	37	15	62767926		0.46	0.01197	1.92207	0.71415023	0.575087561	0.139
cg03328299	II	37	15	74281983	STOML1	0.62	0.0001	3.98291	0.66259422	0.801670737	-0.139
cg23335299	II	37	15	90291756		0.58	0.00041	3.39184	0.68483769	0.82353196	-0.139
cg02593884	II	37	16	2984574	FLYWCH1;FLYWCH1	0.54	0.0014	2.85345	0.66754326	0.806540776	-0.139
cg07427438	II	37	16	4819304		0.5	0.00432	2.36443	0.58985215	0.728437254	-0.139
cg01461211	II	37	16	85478908		0.42	0.02991	1.52413	0.46431409	0.325668658	0.139
cg02976843	II	37	17	2843257	RAP1GAP2;RAP1GA	0.54	0.0014	2.85345	0.5668403	0.705808326	-0.139
cg08860136	I	37	17	7111414	DLG4;DLG4	0.67	2.34E-05	4.63072	0.72458815	0.585976928	0.139
cg25708364	II	37	17	18905779	SLC5A10;FAM83G;S	0.46	0.01197	1.92207	0.41709451	0.556324889	-0.139
cg22186223	II	37	17	32690367	CCL1	0.54	0.0014	2.85345	0.49012558	0.628991797	-0.139
cg02780988	II	37	17	39646994	KRT36	0.5	0.00432	2.36443	0.57498354	0.71356488	-0.139
cg20769177	I	37	17	44928516	WNT9B	0.5	0.00432	2.36443	0.12995659	0.269242823	-0.139
cg27016107	I	37	17	47329321		0.67	2.34E-05	4.63072	0.78252162	0.921258923	-0.139
cg26475911	II	37	17	73056187	KCTD2	0.58	0.00041	3.39184	0.40056678	0.539681173	-0.139
cg04476874	II	37	19	345778	MIER2	0.62	0.0001	3.98291	0.56637943	0.705370405	-0.139
cg03875330	II	37	19	11455923	TMEM205;TMEM20	0.5	0.00432	2.36443	0.39116894	0.530229179	-0.139
cg00509772	II	37	20	32273129	E2F1	0.46	0.01197	1.92207	0.4093294	0.270415121	0.139
cg04250930	II	37	22	24823141	ADORA2A	0.46	0.01197	1.92207	0.62247743	0.483959674	0.139
cg21271945	II	37	1	2080081	PRKCZ;PRKCZ;PRKC	0.42	0.02991	1.52413	0.49930844	0.636997208	-0.138
cg22344254	II	37	1	3826803	LOC100133612	0.42	0.02991	1.52413	0.64341346	0.50512559	0.138
cg04226804	II	37	1	16556249		0.58	0.00041	3.39184	0.55440218	0.415940736	0.138

cg10776377	II	37	1	85359334	LPAR3	0.46	0.01197	1.92207	0.41529376	0.277514042	0.138
cg18346412	II	37	1	111929363	LOC441897	0.46	0.01197	1.92207	0.45142327	0.313264157	0.138
cg16104450	II	37	1	243645911	SDCCAG8	0.67	2.34E-05	4.63072	0.4093179	0.547812983	-0.138
cg01154283	II	37	2	36603543	CRIM1	0.62	0.0001	3.98291	0.48715708	0.625273087	-0.138
cg08879579	II	37	2	239478440		0.62	0.0001	3.98291	0.45920126	0.597207179	-0.138
cg14473924	II	37	3	73674170	PDZRN3	0.62	0.0001	3.98291	0.26059846	0.399012088	-0.138
cg25510614	II	37	4	683772	MFSD7	0.54	0.0014	2.85345	0.36099466	0.498866985	-0.138
cg27661394	II	37	4	4661952		0.67	2.34E-05	4.63072	0.71594292	0.854406958	-0.138
cg25987564	I	37	4	6010075		0.58	0.00041	3.39184	0.42735532	0.565837275	-0.138
cg23411150	II	37	6	1643624	GMDS	0.5	0.00432	2.36443	0.26307179	0.401306015	-0.138
cg14114133	II	37	6	10839408		0.46	0.01197	1.92207	0.68874897	0.550426934	0.138
cg11946459	I	37	6	29911558	HLA-A	0.54	0.0014	2.85345	0.63622477	0.49804488	0.138
cg00128100	II	37	6	30656094	KIAA1949;NRM;KIA	0.42	0.02991	1.52413	0.77753005	0.63940982	0.138
cg21105955	II	37	6	30684478	MDC1	0.46	0.01197	1.92207	0.62987055	0.492098302	0.138
cg08827454	II	37	6	30922981		0.58	0.00041	3.39184	0.24141317	0.379490816	-0.138
cg10180404	I	37	6	32632334	HLA-DQB1	0.42	0.02991	1.52413	0.41862226	0.280297625	0.138
cg01915609	II	37	6	146864388	RAB32	0.58	0.00041	3.39184	0.13557218	0.273386619	-0.138
cg17372657	I	37	7	1216933		0.46	0.01197	1.92207	0.45642527	0.318061	0.138
cg05533001	II	37	7	2019608	MAD1L1;MAD1L1;M	0.5	0.00432	2.36443	0.59941515	0.461808489	0.138
cg03744763	II	37	7	27184737	HOXA5	0.54	0.0014	2.85345	0.39374638	0.531825083	-0.138
cg02183564	II	37	7	76874892	CCDC146	0.5	0.00432	2.36443	0.71145036	0.8492996	-0.138
cg07876788	II	37	7	134849633	TMEM140	0.46	0.01197	1.92207	0.42540034	0.563446621	-0.138
cg16697850	II	37	7	141946928	MOXD2	0.5	0.00432	2.36443	0.74810333	0.609884485	0.138
cg20088245	II	37	8	1321375		0.42	0.02991	1.52413	0.3905613	0.528567507	-0.138
cg26392737	II	37	8	21916853	EPB49;EPB49;EPB4	0.5	0.00432	2.36443	0.46090562	0.599392383	-0.138
cg26980244	II	37	8	24772513	NEFM;NEFM;NEFM	0.54	0.0014	2.85345	0.15148136	0.289917472	-0.138
cg06992846	II	37	8	126448837	TRIB1	0.5	0.00432	2.36443	0.50175905	0.640094918	-0.138
cg20071744	I	37	8	141057329	TRAPPC9;TRAPPC9	0.42	0.02991	1.52413	0.75492602	0.616877302	0.138
cg14435659	II	37	9	84308250		0.42	0.02991	1.52413	0.67953784	0.541136786	0.138
cg13549345	I	37	9	138904964	NACC2	0.5	0.00432	2.36443	0.86042778	0.722639413	0.138
cg05254221	II	37	10	61050062	FAM13C;FAM13C;F	0.54	0.0014	2.85345	0.78920225	0.65140386	0.138
cg02909446	II	37	10	73849124	SPOCK2;SPOCK2	0.54	0.0014	2.85345	0.68049659	0.542324659	0.138
cg12083893	II	37	10	91111929		0.46	0.01197	1.92207	0.76124109	0.623080439	0.138

cg02980499	II	37	10	98031964	BLNK;BLNK	0.46	0.01197	1.92207	0.72494943	0.587112913	0.138
cg15280185	II	37	10	103500442		0.5	0.00432	2.36443	0.71530139	0.577170912	0.138
cg09985802	II	37	10	126234119	LHPP;LHPP	0.62	0.0001	3.98291	0.51978427	0.381390973	0.138
cg00568164	II	37	11	44927222	TSPAN18;TSPAN18	0.46	0.01197	1.92207	0.67199645	0.53389218	0.138
cg21667069	II	37	11	67778641	ALDH3B1;ALDH3B1	0.54	0.0014	2.85345	0.40896913	0.547407898	-0.138
cg01508380	II	37	14	23305585	MMP14	0.54	0.0014	2.85345	0.30452018	0.442182986	-0.138
cg02072834	II	37	14	76589894		0.5	0.00432	2.36443	0.57161657	0.433518169	0.138
cg07076915	I	37	16	2174754	PKD1;PKD1	0.46	0.01197	1.92207	0.36821333	0.506134108	-0.138
cg06833564	I	37	16	4665307	FAM100A	0.62	0.0001	3.98291	0.8714561	0.733027559	0.138
cg04904276	I	37	16	11835562	TXNDC11	0.5	0.00432	2.36443	0.68597105	0.547791039	0.138
cg00204512	II	37	16	28754710		0.46	0.01197	1.92207	0.27493127	0.413328252	-0.138
cg07574924	II	37	16	81682484	CMIP;CMIP	0.54	0.0014	2.85345	0.58403485	0.722055544	-0.138
cg26697517	II	37	16	88152986		0.54	0.0014	2.85345	0.49884927	0.637221065	-0.138
cg04364752	II	37	16	88906534	GALNS	0.46	0.01197	1.92207	0.436208	0.574649861	-0.138
cg02679012	I	37	17	773283	NXN	0.5	0.00432	2.36443	0.73353444	0.595456912	0.138
cg27060340	I	37	17	43502999	ARHGAP27;ARHGAI	0.46	0.01197	1.92207	0.26285498	0.401198495	-0.138
cg20830994	II	37	17	62401400	PECAM1	0.54	0.0014	2.85345	0.58774267	0.449654285	0.138
cg04334723	II	37	19	13054427	CALR	0.42	0.02991	1.52413	0.49774577	0.635865886	-0.138
cg09231418	II	37	19	36202390	ZBTB32	0.5	0.00432	2.36443	0.64417633	0.506042877	0.138
cg24548817	II	37	21	45774294	TRPM2	0.58	0.00041	3.39184	0.24988183	0.387902047	-0.138
cg20376123	I	37	22	46760595	CELSR1	0.46	0.01197	1.92207	0.55781427	0.420176359	0.138
cg25392995	II	37	22	50625926	TRABD	0.46	0.01197	1.92207	0.53766067	0.400049264	0.138
cg23629166	I	37	1	2345368	PEX10;PEX10	0.5	0.00432	2.36443	0.73922827	0.601941176	0.137
cg27573298	II	37	1	14697589		0.5	0.00432	2.36443	0.58492724	0.44769978	0.137
cg24593372	II	37	1	54562121	C1orf83	0.54	0.0014	2.85345	0.28850435	0.425646807	-0.137
cg19645639	I	37	1	113500384	SLC16A1	0.46	0.01197	1.92207	0.69524507	0.558391523	0.137
cg11815057	I	37	1	153670855		0.54	0.0014	2.85345	0.45978417	0.322537538	0.137
cg06980173	II	37	1	154376344	IL6R;IL6R	0.5	0.00432	2.36443	0.41241288	0.549320795	-0.137
cg22018051	II	37	1	154943349	SHC1;SHC1;SHC1;SI	0.54	0.0014	2.85345	0.3315891	0.468972288	-0.137
cg13861536	II	37	1	180904914	KIAA1614	0.42	0.02991	1.52413	0.43197689	0.294850793	0.137
cg06511330	II	37	1	234635574		0.42	0.02991	1.52413	0.61758547	0.75457287	-0.137
cg10929758	II	37	2	54857270	SPTBN1;SPTBN1	0.46	0.01197	1.92207	0.78864957	0.651716551	0.137
cg26476820	II	37	2	85637851	CAPG	0.5	0.00432	2.36443	0.45714729	0.594133705	-0.137

cg00605777	II	37	2	97533635	SEMA4C	0.58	0.00041	3.39184	0.42585041	0.562508386	-0.137
cg00010078	II	37	2	109967172	SH3RF3	0.5	0.00432	2.36443	0.36639025	0.503806028	-0.137
cg04738464	II	37	2	218868754		0.5	0.00432	2.36443	0.28413117	0.421182078	-0.137
cg15002163	II	37	2	240161924	HDAC4	0.67	2.34E-05	4.63072	0.5845691	0.72183126	-0.137
cg22870429	II	37	3	114027859	TIGIT	0.54	0.0014	2.85345	0.70655943	0.569548381	0.137
cg05480110	I	37	3	195534854	MUC4;MUC4;MUC4	0.54	0.0014	2.85345	0.61756698	0.480698986	0.137
cg24826020	II	37	4	38070998	TBC1D1	0.58	0.00041	3.39184	0.21861857	0.355612322	-0.137
cg02375208	II	37	5	77804381	LHFPL2	0.58	0.00041	3.39184	0.25843869	0.395666757	-0.137
cg07875121	II	37	5	139641530	PFDN1	0.58	0.00041	3.39184	0.67995206	0.817084962	-0.137
cg01823925	II	37	5	156967901	ADAM19	0.54	0.0014	2.85345	0.50980556	0.372480276	0.137
cg01970383	II	37	5	177729224	COL23A1	0.42	0.02991	1.52413	0.7104287	0.573491759	0.137
cg08376141	II	37	6	32116591	PRRT1	0.54	0.0014	2.85345	0.25787185	0.394846532	-0.137
cg06902099	II	37	6	35002597	ANKS1A	0.5	0.00432	2.36443	0.19939107	0.336078256	-0.137
cg00807871	I	37	6	37617124	MDGA1	0.42	0.02991	1.52413	0.23666167	0.374161397	-0.137
cg23317857	II	37	6	41696297	TFEB;TFEB	0.62	0.0001	3.98291	0.5791249	0.441701002	0.137
cg09293890	II	37	6	43682839		0.46	0.01197	1.92207	0.44178713	0.304790742	0.137
cg14835981	II	37	7	752715	PRKAR1B;PRKAR1B	0.62	0.0001	3.98291	0.48968988	0.626276389	-0.137
cg02185182	II	37	7	2185550	MAD1L1;MAD1L1;M	0.5	0.00432	2.36443	0.68032732	0.817268836	-0.137
cg06898168	II	37	7	5447353	TNRC18	0.58	0.00041	3.39184	0.58452898	0.721926289	-0.137
cg00852675	I	37	7	150105086	LOC728743	0.46	0.01197	1.92207	0.74361595	0.606484232	0.137
cg17082719	I	37	8	144896176	SCRIB;SCRIB;MIR93	0.58	0.00041	3.39184	0.78885798	0.925802594	-0.137
cg13863007	II	37	9	95766607	FGD3;FGD3	0.5	0.00432	2.36443	0.6149431	0.752116919	-0.137
cg13458384	II	37	9	136567339	SARDH;SARDH	0.54	0.0014	2.85345	0.63313688	0.496258127	0.137
cg14135809	II	37	9	140502926	ARRDC1	0.5	0.00432	2.36443	0.35456562	0.491122143	-0.137
cg25024734	II	37	10	112152815		0.46	0.01197	1.92207	0.21147471	0.348265306	-0.137
cg12323347	II	37	10	112256517	DUSP5	0.5	0.00432	2.36443	0.36005025	0.496619682	-0.137
cg05261592	II	37	11	8215201		0.46	0.01197	1.92207	0.63056272	0.493086891	0.137
cg10437839	II	37	11	64701910	PPP2R5B	0.5	0.00432	2.36443	0.68994223	0.826599499	-0.137
cg03371384	II	37	11	69260271		0.62	0.0001	3.98291	0.73855089	0.875059466	-0.137
cg19388930	II	37	11	129564373		0.58	0.00041	3.39184	0.66916417	0.532254442	0.137
cg03392386	I	37	11	133800800	IGSF9B	0.58	0.00041	3.39184	0.80166678	0.664686482	0.137
cg24437859	II	37	12	7066614	PTPN6;PTPN6;PTPN	0.42	0.02991	1.52413	0.67721661	0.540297545	0.137
cg06395232	II	37	12	15105654	ARHGDIB	0.46	0.01197	1.92207	0.41122216	0.27461887	0.137

cg17332198	II	37	12	58209913	AVIL	0.58	0.00041	3.39184	0.52051122	0.657420067	-0.137
cg04456029	I	37	12	113496126	DTX1	0.46	0.01197	1.92207	0.72642706	0.589909205	0.137
cg04716447	I	37	12	122444621		0.54	0.0014	2.85345	0.47096386	0.333826493	0.137
cg21598343	II	37	13	114261986	TFDP1;TFDP1	0.58	0.00041	3.39184	0.36616428	0.502868313	-0.137
cg12938556	I	37	13	114812918	RASA3	0.62	0.0001	3.98291	0.62251299	0.485765824	0.137
cg02039171	II	37	14	23588162	CEBPE	0.58	0.00041	3.39184	0.54118637	0.678102162	-0.137
cg01098955	II	37	14	106322163		0.46	0.01197	1.92207	0.69011339	0.553339873	0.137
cg23934731	I	37	15	89904873		0.42	0.02991	1.52413	0.55821915	0.695311134	-0.137
cg04848686	II	37	16	88747591	MGC23284;SNAI3	0.5	0.00432	2.36443	0.60621323	0.469026816	0.137
cg09115713	I	37	16	88832476	FAM38A	0.5	0.00432	2.36443	0.82867297	0.691591363	0.137
cg11801411	II	37	17	1628727	WDR81;WDR81;WI	0.58	0.00041	3.39184	0.41861421	0.555880933	-0.137
cg14918082	II	37	17	7833237	KCNAB3	0.46	0.01197	1.92207	0.69199611	0.555219545	0.137
cg13190608	II	37	17	37322730	ARL5C	0.42	0.02991	1.52413	0.44572901	0.582631114	-0.137
cg26336935	II	37	17	39769213	KRT16	0.62	0.0001	3.98291	0.52608923	0.662608477	-0.137
cg05465955	I	37	17	40715236	COASY;COASY;COA:	0.46	0.01197	1.92207	0.77903074	0.642057265	0.137
cg19106478	II	37	17	40800053		0.54	0.0014	2.85345	0.24363656	0.106858845	0.137
cg13948956	II	37	17	55210898		0.46	0.01197	1.92207	0.61656313	0.479240417	0.137
cg11699517	I	37	17	79419796	BAHCC1	0.42	0.02991	1.52413	0.63751193	0.500688783	0.137
cg24380489	II	37	17	79793082	DYSFIP1	0.62	0.0001	3.98291	0.65817836	0.795406024	-0.137
cg00830755	II	37	17	80819020	TBCD	0.5	0.00432	2.36443	0.672121	0.534979846	0.137
cg26052586	I	37	18	20717411	CABLES1;CABLES1	0.46	0.01197	1.92207	0.30309289	0.439974074	-0.137
cg12062672	I	37	19	643555	FGF22	0.42	0.02991	1.52413	0.72196625	0.585357357	0.137
cg24926276	II	37	19	4539943	LRG1	0.58	0.00041	3.39184	0.61757303	0.75467004	-0.137
cg20234640	II	37	19	7767089	FCER2	0.46	0.01197	1.92207	0.42118821	0.284460869	0.137
cg09277709	II	37	19	46224285	FBXO46	0.54	0.0014	2.85345	0.74381537	0.880653694	-0.137
cg25975071	II	37	19	55173527	LILRB4;LILRB4	0.46	0.01197	1.92207	0.56280383	0.425344847	0.137
cg00087601	II	37	21	35747094	FAM165B	0.5	0.00432	2.36443	0.73088804	0.867969616	-0.137
cg12746557	II	37	21	39644332	KCNJ15;KCNJ15;KCI	0.42	0.02991	1.52413	0.38413752	0.521575816	-0.137
cg03364108	I	37	22	24890794	C22orf45;C22orf45	0.54	0.0014	2.85345	0.12611982	0.26270778	-0.137
cg21757633	II	37	22	50847567	SAPS2	0.62	0.0001	3.98291	0.50215047	0.639272265	-0.137
cg21775570	II	37	1	2171834	SKI	0.62	0.0001	3.98291	0.36464916	0.501130506	-0.136
cg13551754	II	37	1	21619913	ECE1	0.5	0.00432	2.36443	0.38787314	0.252004889	0.136
cg04573706	II	37	1	22216432	HSPG2	0.54	0.0014	2.85345	0.78714903	0.651566739	0.136

cg03655701	II	37	1	24885664	C1orf130	0.5	0.00432	2.36443	0.31301107	0.449086174	-0.136
cg07794010	II	37	1	114665617	SYT6	0.54	0.0014	2.85345	0.56156499	0.697878254	-0.136
cg16409409	I	37	1	200861597	C1orf106	0.5	0.00432	2.36443	0.29554864	0.431521422	-0.136
cg07561747	II	37	1	200983313	KIF21B	0.54	0.0014	2.85345	0.65572876	0.791427615	-0.136
cg01057552	II	37	1	228415374	OBSCN;OBSCN	0.54	0.0014	2.85345	0.6653443	0.801514201	-0.136
cg02844899	II	37	2	47182345	TTC7A	0.54	0.0014	2.85345	0.49627629	0.631923791	-0.136
cg13000080	II	37	2	75043142		0.46	0.01197	1.92207	0.4609462	0.596626409	-0.136
cg22670147	I	37	2	115419740	DPP10	0.5	0.00432	2.36443	0.17905775	0.314801209	-0.136
cg00748072	II	37	2	174862824		0.5	0.00432	2.36443	0.73359824	0.869118957	-0.136
cg19955928	I	37	2	240152257	HDAC4	0.54	0.0014	2.85345	0.30128454	0.437231259	-0.136
cg18644782	II	37	3	13045896	IQSEC1	0.5	0.00432	2.36443	0.66394081	0.528207491	0.136
cg24296397	II	37	3	49692537	BSN	0.46	0.01197	1.92207	0.39680493	0.260874028	0.136
cg05622438	II	37	3	51419127	DOCK3	0.54	0.0014	2.85345	0.65572176	0.792018161	-0.136
cg17491449	II	37	4	3344154	RGS12;RGS12	0.54	0.0014	2.85345	0.26856688	0.404086214	-0.136
cg13929988	I	37	4	88140436	KLHL8	0.58	0.00041	3.39184	0.31962567	0.455841758	-0.136
cg17598923	II	37	5	101119084		0.46	0.01197	1.92207	0.51026588	0.646330394	-0.136
cg18331412	I	37	5	135364986	TGFBI	0.54	0.0014	2.85345	0.50875026	0.644519795	-0.136
cg08747807	II	37	5	176005051	PCDH24	0.58	0.00041	3.39184	0.69907123	0.563299175	0.136
cg12633154	I	37	6	30039435	RNF39;RNF39	0.46	0.01197	1.92207	0.14067382	0.276900089	-0.136
cg14136042	II	37	6	31513306	ATP6V1G2;NFKBIL1	0.54	0.0014	2.85345	0.78370595	0.647876877	0.136
cg10871120	II	37	6	39891273	MOCS1;MOCS1;MC	0.46	0.01197	1.92207	0.50503335	0.641489258	-0.136
cg00806239	II	37	6	43456984	TJAP1;TJAP1;TJAP1	0.5	0.00432	2.36443	0.71736335	0.581647965	0.136
cg00782811	II	37	6	46293734	RCAN2	0.5	0.00432	2.36443	0.47244871	0.336837547	0.136
cg12366968	II	37	7	1883876	MAD1L1;MAD1L1;M	0.42	0.02991	1.52413	0.38928354	0.253367283	0.136
cg20947553	II	37	7	6440601	RAC1;RAC1	0.67	2.34E-05	4.63072	0.72335355	0.859460465	-0.136
cg18463876	II	37	7	75906035	SRRM3	0.58	0.00041	3.39184	0.71049843	0.574929454	0.136
cg07125725	II	37	7	99612773	ZKSCAN1	0.67	2.34E-05	4.63072	0.46716618	0.603438857	-0.136
cg19889856	II	37	7	134856654	C7orf49;C7orf49;C7	0.5	0.00432	2.36443	0.62182033	0.758218939	-0.136
cg27497928	II	37	7	143049069	CLCN1	0.58	0.00041	3.39184	0.56981025	0.43369047	0.136
cg13538006	II	37	9	95897313	NINJ1	0.46	0.01197	1.92207	0.3640378	0.228090893	0.136
cg23680451	II	37	10	3823790	KLF6;KLF6;KLF6;KLF	0.42	0.02991	1.52413	0.34076656	0.476608314	-0.136
cg06784824	II	37	11	47376985	SPI1;SPI1	0.5	0.00432	2.36443	0.62102872	0.756684704	-0.136
cg10675058	II	37	11	63858880	MACROD1	0.5	0.00432	2.36443	0.41203114	0.547643017	-0.136

cg02023728	II	37	11	77925099	USP35	0.46	0.01197	1.92207	0.58916726	0.724905786	-0.136
cg08182171	II	37	11	130291156	ADAMTS8	0.5	0.00432	2.36443	0.48289413	0.618963774	-0.136
cg19369955	I	37	12	2030178		0.58	0.00041	3.39184	0.11110925	0.247242801	-0.136
cg27478242	I	37	12	54611928		0.46	0.01197	1.92207	0.45322888	0.317063305	0.136
cg18236066	II	37	12	113293823	RPH3A;RPH3A	0.58	0.00041	3.39184	0.62673915	0.763130675	-0.136
cg18449739	II	37	12	113495701	DTX1;DTX1	0.58	0.00041	3.39184	0.66410359	0.527880204	0.136
cg24191847	II	37	14	91037045	TTC7B	0.71	4.57E-06	5.34042	0.59993669	0.735693264	-0.136
cg09717923	I	37	16	1440498		0.5	0.00432	2.36443	0.73702319	0.600773184	0.136
cg01389428	II	37	16	11594557		0.5	0.00432	2.36443	0.50351532	0.639749206	-0.136
cg26685404	II	37	16	23957272	PRKCB;PRKCB	0.46	0.01197	1.92207	0.41536928	0.551111202	-0.136
cg04416111	II	37	16	87234895		0.46	0.01197	1.92207	0.79551041	0.659892696	0.136
cg03272932	II	37	16	87588893		0.5	0.00432	2.36443	0.61340626	0.749189528	-0.136
cg24476449	II	37	17	25799212	KSR1	0.46	0.01197	1.92207	0.60459553	0.468267945	0.136
cg01351315	II	37	17	46667737	LOC404266;LOC404	0.54	0.0014	2.85345	0.22306991	0.358970601	-0.136
cg11598872	II	37	17	75881232	FLJ45079	0.62	0.0001	3.98291	0.53113206	0.394983976	0.136
cg21320221	II	37	17	79792997	DYSFIP1	0.58	0.00041	3.39184	0.68658954	0.822157594	-0.136
cg06394229	II	37	19	39303775	LGALS4	0.54	0.0014	2.85345	0.5087074	0.644466917	-0.136
cg06580800	II	37	19	39921535		0.46	0.01197	1.92207	0.27991707	0.415617766	-0.136
cg03138287	I	37	19	49440536	DHDH	0.58	0.00041	3.39184	0.71472705	0.850748134	-0.136
cg22321036	II	37	19	56061348		0.42	0.02991	1.52413	0.79191675	0.655941819	0.136
cg04368843	II	37	20	3660815	ADAM33;ADAM33	0.54	0.0014	2.85345	0.65790726	0.521492766	0.136
cg09273716	II	37	20	35494835		0.5	0.00432	2.36443	0.59707905	0.733199838	-0.136
cg01064979	II	37	22	37309487	CSF2RB	0.46	0.01197	1.92207	0.39092804	0.526767637	-0.136
cg02769477	I	37	1	17012728		0.54	0.0014	2.85345	0.64934664	0.514352617	0.135
cg22558673	II	37	1	35257858	GJA4	0.5	0.00432	2.36443	0.54903265	0.414398359	0.135
cg17434008	II	37	1	38974111		0.58	0.00041	3.39184	0.44775001	0.582707549	-0.135
cg23656322	II	37	1	153533922	S100A2	0.58	0.00041	3.39184	0.5565598	0.691869219	-0.135
cg13077784	II	37	1	201706363	NAV1	0.46	0.01197	1.92207	0.36063006	0.225440922	0.135
cg04222159	I	37	1	204981786	NFASC;NFASC;NFAS	0.62	0.0001	3.98291	0.51528618	0.38003506	0.135
cg02035751	II	37	1	206729099	RASSF5;RASSF5;RAS	0.54	0.0014	2.85345	0.6258531	0.490778325	0.135
cg03054277	I	37	1	228400217	OBSCN;OBSCN	0.5	0.00432	2.36443	0.7801617	0.645367448	0.135
cg07052737	II	37	2	26224428		0.5	0.00432	2.36443	0.52712133	0.662147547	-0.135
cg04526104	II	37	2	217839249		0.54	0.0014	2.85345	0.72173738	0.586271753	0.135

cg06753787	II	37	2	220074208	ZFAND2B	0.71	4.57E-06	5.34042	0.43912546	0.574045722	-0.135
cg05058748	II	37	2	235903542	SH3BP4	0.46	0.01197	1.92207	0.5006116	0.635751456	-0.135
cg11557929	II	37	3	48489291	ATRIP;ATRIP	0.58	0.00041	3.39184	0.7193023	0.854431939	-0.135
cg04967039	II	37	4	8292202	HTRA3	0.54	0.0014	2.85345	0.5475923	0.412539209	0.135
cg19668476	II	37	4	153506267		0.67	2.34E-05	4.63072	0.80507927	0.66972507	0.135
cg10586870	II	37	5	75722317	IQGAP2	0.46	0.01197	1.92207	0.25972133	0.394764435	-0.135
cg27300619	II	37	5	139120130		0.42	0.02991	1.52413	0.61130657	0.476600836	0.135
cg27315555	II	37	5	142211162	ARHGAP26;ARHGAI	0.62	0.0001	3.98291	0.6745041	0.809841457	-0.135
cg18307303	II	37	5	158757456	IL12B;IL12B	0.58	0.00041	3.39184	0.65997203	0.79519764	-0.135
cg19576697	II	37	5	176837920	F12	0.42	0.02991	1.52413	0.42716386	0.562551965	-0.135
cg16907934	II	37	6	3848904	FAM50B	0.58	0.00041	3.39184	0.69638796	0.831597522	-0.135
cg24735489	II	37	6	31088352	CDSN;PSORS1C1	0.54	0.0014	2.85345	0.36622767	0.501233293	-0.135
cg17113856	II	37	6	32120895	PPT2;PRRT1;PPT2	0.5	0.00432	2.36443	0.71879645	0.584189994	0.135
cg07417146	I	37	6	33400528	SYNGAP1	0.5	0.00432	2.36443	0.63056643	0.765117613	-0.135
cg05568549	II	37	6	41907198	CCND3;CCND3;CCN	0.58	0.00041	3.39184	0.30526196	0.440066714	-0.135
cg03316570	II	37	6	43877657	LOC100132354	0.42	0.02991	1.52413	0.64692585	0.512128133	0.135
cg16313758	II	37	7	2647039	IQCE;IQCE	0.62	0.0001	3.98291	0.65901183	0.794337274	-0.135
cg11554295	II	37	7	5523691	FBXL18	0.5	0.00432	2.36443	0.5320329	0.667428084	-0.135
cg13736939	II	37	7	157647241	PTPRN2;PTPRN2;PT	0.67	2.34E-05	4.63072	0.31867279	0.453530318	-0.135
cg13434842	I	37	8	11567896	GATA4	0.46	0.01197	1.92207	0.28045952	0.415229226	-0.135
cg26209487	I	37	8	56931739		0.62	0.0001	3.98291	0.50734088	0.372268284	0.135
cg14162361	II	37	9	82449859		0.5	0.00432	2.36443	0.76969165	0.634855021	0.135
cg11884704	II	37	9	130854313	SLC25A25;SLC25A2	0.67	2.34E-05	4.63072	0.58553711	0.720555933	-0.135
cg13572237	II	37	9	139266293	CARD9;CARD9	0.5	0.00432	2.36443	0.66328039	0.528229635	0.135
cg23054840	II	37	10	45920485	ALOX5	0.5	0.00432	2.36443	0.33674168	0.47202851	-0.135
cg16265932	II	37	10	112460964	RBM20	0.46	0.01197	1.92207	0.78924994	0.654031707	0.135
cg19317226	I	37	10	130726701		0.42	0.02991	1.52413	0.73734813	0.872373732	-0.135
cg19149132	I	37	10	130854436		0.5	0.00432	2.36443	0.76928464	0.634247556	0.135
cg16498504	II	37	11	2043711		0.5	0.00432	2.36443	0.42626883	0.561276581	-0.135
cg02258534	II	37	11	2555497	KCNQ1;KCNQ1	0.75	7.61E-07	6.11857	0.49253086	0.627652126	-0.135
cg03211593	II	37	11	44731693		0.62	0.0001	3.98291	0.41071542	0.545345276	-0.135
cg21002528	II	37	11	45903754	CRY2;CRY2	0.5	0.00432	2.36443	0.66323492	0.798636241	-0.135
cg12980788	II	37	11	55641084		0.58	0.00041	3.39184	0.56674072	0.702004037	-0.135

cg25249849	II	37	11	117695486	FXYD2;FXYD2;FXYD	0.5	0.00432	2.36443	0.56053236	0.695868435	-0.135
cg01572652	II	37	12	103695883	C12orf42;C12orf42	0.46	0.01197	1.92207	0.279734	0.415202001	-0.135
cg05404091	II	37	12	133182886		0.54	0.0014	2.85345	0.70284103	0.567743617	0.135
cg17292667	II	37	13	110787523		0.58	0.00041	3.39184	0.70147776	0.836810291	-0.135
cg08264895	II	37	14	70060335		0.5	0.00432	2.36443	0.65089987	0.516002785	0.135
cg05859308	II	37	14	71712645		0.46	0.01197	1.92207	0.67640139	0.54184975	0.135
cg13754877	II	37	14	101943654		0.5	0.00432	2.36443	0.69220255	0.557316845	0.135
cg26488183	II	37	15	75195105	C15orf17	0.5	0.00432	2.36443	0.27823638	0.412891028	-0.135
cg00044665	II	37	16	66400411	CDH5	0.67	2.34E-05	4.63072	0.30004619	0.435295555	-0.135
cg07248223	I	37	17	38717275	CCR7	0.58	0.00057	3.24571	0.20837639	0.073849235	0.135
cg03244997	II	37	17	43503144	ARHGAP27	0.5	0.00432	2.36443	0.34788441	0.48260473	-0.135
cg26933683	II	37	17	61918738	SMARCD2;SMARCD	0.5	0.00432	2.36443	0.38826851	0.25368136	0.135
cg26928788	II	37	17	76973328	LGALS3BP	0.42	0.02991	1.52413	0.53110232	0.396349748	0.135
cg00504599	II	37	17	79428036	BAHCC1	0.5	0.00432	2.36443	0.49434593	0.629395774	-0.135
cg24431966	II	37	17	79801787	P4HB	0.62	0.0001	3.98291	0.71777416	0.852727751	-0.135
cg06360427	I	37	18	74962672	GALR1	0.5	0.00432	2.36443	0.12912066	0.263881957	-0.135
cg05345154	I	37	19	2250901	AMH	0.42	0.02991	1.52413	0.22644896	0.361889531	-0.135
cg08196968	I	37	19	38746749	PPP1R14A	0.58	0.00041	3.39184	0.37551369	0.240113167	0.135
cg05670991	II	37	19	51628115	SIGLEC9	0.58	0.00041	3.39184	0.3385565	0.473574312	-0.135
cg19853760	II	37	22	38071677	LGALS1;LGALS1	0.54	0.0014	2.85345	0.38134769	0.516583209	-0.135
cg24424889	II	37	22	39494175	APOBEC3H;APOBEC	0.42	0.02991	1.52413	0.68040644	0.545286172	0.135
cg17194270	II	37	22	39759992	SYNGR1;SYNGR1;SY	0.54	0.0014	2.85345	0.51962515	0.654394917	-0.135
cg19159092	II	37	1	856059	FLJ39609	0.54	0.0014	2.85345	0.36969758	0.235456657	0.134
cg03265564	II	37	1	9748971	PIK3CD	0.5	0.00432	2.36443	0.72519404	0.591371255	0.134
cg03769939	I	37	1	25254129	RUNX3;RUNX3	0.54	0.0014	2.85345	0.83077732	0.696307529	0.134
cg01523881	II	37	1	200985689	KIF21B	0.58	0.00041	3.39184	0.22462401	0.35885733	-0.134
cg06233202	II	37	2	64501134		0.58	0.00041	3.39184	0.25410965	0.387787944	-0.134
cg19051213	I	37	2	128281334	IWS1	0.46	0.01197	1.92207	0.75881071	0.625029974	0.134
cg22033586	II	37	2	157292113	GPD2;GPD2;GPD2	0.5	0.00432	2.36443	0.20539829	0.339028065	-0.134
cg12073466	II	37	2	173069253		0.5	0.00432	2.36443	0.7736573	0.908051968	-0.134
cg01786048	II	37	2	239035964	ESPNL	0.42	0.02991	1.52413	0.25862186	0.392480162	-0.134
cg09690215	II	37	2	241216697		0.58	0.00041	3.39184	0.6325356	0.498225036	0.134
cg25585279	II	37	4	2763026		0.46	0.01197	1.92207	0.63605383	0.501813693	0.134

cg18371052	I	37	5	8457721		0.46	0.01197	1.92207	0.42985552	0.295487446	0.134
cg14815361	I	37	5	131589552		0.54	0.0014	2.85345	0.70041178	0.566673455	0.134
cg06033531	II	37	5	176980153	FAM193B;FAM193I	0.58	0.00041	3.39184	0.65984311	0.794169269	-0.134
cg14029759	II	37	6	5764816	FARS2	0.42	0.02991	1.52413	0.72517642	0.591278567	0.134
cg13500852	II	37	6	15505460	JARID2	0.67	2.34E-05	4.63072	0.65471401	0.788413661	-0.134
cg26655361	II	37	6	28323128	ZNF323;ZKSCAN3;Z	0.54	0.0014	2.85345	0.66270049	0.796318826	-0.134
cg10661769	I	37	6	30881484	VARS2;VARS2;VARS	0.46	0.01197	1.92207	0.47601427	0.341961505	0.134
cg03499675	II	37	6	31744339	C6orf27	0.71	4.57E-06	5.34042	0.39456389	0.528343853	-0.134
cg17568962	II	37	6	35456312	TEAD3	0.54	0.0014	2.85345	0.25857444	0.393010036	-0.134
cg27250236	I	37	6	48036617	C6orf138	0.5	0.00432	2.36443	0.20789096	0.342297445	-0.134
cg15506609	I	37	7	27206073	HOXA9	0.46	0.01197	1.92207	0.18280628	0.317072869	-0.134
cg19198358	II	37	8	1725179	CLN8	0.58	0.00041	3.39184	0.72053089	0.854854659	-0.134
cg23303108	I	37	8	23083578	TNFRSF10A	0.46	0.01197	1.92207	0.81728194	0.683207797	0.134
cg27443224	II	37	9	34710135	CCL21;CCL21	0.42	0.02991	1.52413	0.45431093	0.588089346	-0.134
cg09501025	II	37	10	73312955	CDH23;CDH23	0.5	0.00432	2.36443	0.76072831	0.62698926	0.134
cg05379597	II	37	10	75627144	CAMK2G;CAMK2G;	0.58	0.00041	3.39184	0.64051007	0.774617851	-0.134
cg12069540	II	37	10	82173570	C10orf58;C10orf58	0.54	0.0014	2.85345	0.55574929	0.68932131	-0.134
cg07535628	II	37	11	829035	EFCAB4A	0.62	0.0001	3.98291	0.59034422	0.724041724	-0.134
cg26071135	II	37	11	843943	TSPAN4;TSPAN4;TS	0.62	0.0001	3.98291	0.23360908	0.367772241	-0.134
cg01199327	I	37	11	44626750	CD82;CD82	0.54	0.0014	2.85345	0.54018527	0.405893867	0.134
cg24524285	I	37	11	64405919	NRXN2;NRXN2;NRX	0.54	0.0014	2.85345	0.56121767	0.426869331	0.134
cg05235171	II	37	11	117958104	TMPRSS4;TMPRSS4	0.62	0.0001	3.98291	0.58505317	0.718898921	-0.134
cg20001810	II	37	12	52829316	KRT75	0.5	0.00432	2.36443	0.6949184	0.560979954	0.134
cg05758804	II	37	12	57917389	MBD6	0.67	2.34E-05	4.63072	0.18185289	0.31542075	-0.134
cg19190762	II	37	13	44806055		0.58	0.00041	3.39184	0.73063549	0.597018228	0.134
cg13688202	II	37	14	65230287	SPTB	0.42	0.02991	1.52413	0.47015656	0.335775582	0.134
cg16650630	II	37	15	40364862		0.42	0.02991	1.52413	0.44690604	0.581143936	-0.134
cg13045913	II	37	15	41100308	DNAJC17;ZFYVE19	0.42	0.02991	1.52413	0.13291263	0.266657014	-0.134
cg23737062	I	37	15	63894296	FBXL22	0.62	0.0001	3.98291	0.5092406	0.375100942	0.134
cg12057576	II	37	15	78918529	CHRNB4	0.54	0.0014	2.85345	0.66981543	0.535457822	0.134
cg01054354	II	37	15	100272751	LYSMD4	0.58	0.00041	3.39184	0.48096594	0.615154448	-0.134
cg00201760	II	37	16	28518385	IL27	0.46	0.01197	1.92207	0.31687632	0.450489587	-0.134
cg27152190	II	37	16	50096867		0.58	0.00041	3.39184	0.79766387	0.66403626	0.134

cg04172533	II	37	16	50743027	NOD2	0.42	0.02991	1.52413	0.29044369	0.424019016	-0.134
cg06835772	II	37	16	85296220		0.58	0.00041	3.39184	0.33047636	0.464090857	-0.134
cg07543400	II	37	17	3824384		0.46	0.01197	1.92207	0.4424189	0.308439647	0.134
cg24251850	II	37	17	8815841	PIK3R5;PIK3R5	0.58	0.00041	3.39184	0.76037504	0.626789968	0.134
cg26673195	II	37	17	41052729	G6PC	0.46	0.01197	1.92207	0.67072838	0.536962905	0.134
cg23643375	II	37	17	45933306	SP6	0.58	0.00041	3.39184	0.69604527	0.830469317	-0.134
cg02240252	II	37	17	47931591		0.54	0.0014	2.85345	0.60383609	0.738260788	-0.134
cg19134130	II	37	17	72830151	TMEM104	0.42	0.02991	1.52413	0.57635838	0.442027546	0.134
cg21373806	I	37	17	79419834	BAHCC1	0.54	0.0014	2.85345	0.60714739	0.472724338	0.134
cg15229496	II	37	18	45939850		0.58	0.00041	3.39184	0.6061716	0.739901716	-0.134
cg24304425	II	37	19	840985	PRTN3;PRTN3	0.62	0.0001	3.98291	0.32415438	0.458235758	-0.134
cg24866700	II	37	19	2235103	MIR1227;PLEKHJ1	0.46	0.01197	1.92207	0.33038914	0.464612976	-0.134
cg08006956	II	37	19	18418056	LSM4	0.62	0.0001	3.98291	0.69858851	0.564954757	0.134
cg24074448	I	37	19	18768688	KLHL26	0.58	0.00041	3.39184	0.61421311	0.479930789	0.134
cg10665321	I	37	19	19221392	SLC25A42	0.5	0.00432	2.36443	0.26600845	0.131882271	0.134
cg21965980	II	37	19	35614992	FXYD3;FXYD3;FXYD	0.42	0.02991	1.52413	0.60834117	0.474047759	0.134
cg19648552	I	37	19	56200391	EPN1;EPN1;EPN1	0.58	0.00041	3.39184	0.66409948	0.530139988	0.134
cg27391267	I	37	19	58545333	ZSCAN1	0.46	0.01197	1.92207	0.10310951	0.237174631	-0.134
cg11284582	II	37	20	62270540		0.54	0.0014	2.85345	0.29215544	0.157968965	0.134
cg13299324	I	37	21	40145949	NCRNA00114	0.42	0.02991	1.52413	0.82741289	0.693058493	0.134
cg23205858	II	37	22	21088806	PI4KA;PI4KA	0.54	0.0014	2.85345	0.52954406	0.663766579	-0.134
cg11163555	II	37	22	31736976	PATZ1;PATZ1;PATZ1	0.46	0.01197	1.92207	0.39229721	0.525970836	-0.134
cg17908947	II	37	22	38600334	MAFF;MAFF;MAFF;	0.54	0.0014	2.85345	0.58464949	0.45042529	0.134
cg01800148	II	37	1	9130886	SLC2A5;SLC2A5;SLC	0.42	0.02991	1.52413	0.77537909	0.641935248	0.133
cg20680726	I	37	1	12254723	TNFRSF1B	0.62	0.0001	3.98291	0.62664071	0.75917741	-0.133
cg00426377	II	37	1	36785859	C1orf113;C1orf113	0.46	0.01197	1.92207	0.40653249	0.539258826	-0.133
cg23217983	II	37	1	67018825	SGIP1	0.5	0.00432	2.36443	0.70753368	0.574282487	0.133
cg05941840	II	37	1	94074034	BCAR3	0.5	0.00432	2.36443	0.83019223	0.697288006	0.133
cg12200412	II	37	1	158324331	CD1E;CD1E;CD1E;C	0.42	0.02991	1.52413	0.65269642	0.785747263	-0.133
cg22449085	II	37	1	201392102	TNNI1	0.54	0.0014	2.85345	0.81861503	0.685852616	0.133
cg08161480	II	37	1	226114565		0.54	0.0014	2.85345	0.55749354	0.690279293	-0.133
cg20991420	II	37	2	173061715		0.46	0.01197	1.92207	0.19933999	0.332310483	-0.133
cg12001120	II	37	2	219751342	WNT10A	0.46	0.01197	1.92207	0.66664145	0.533180318	0.133

cg06574960	II	37	2	232265084	B3GNT7	0.5	0.00432	2.36443	0.64445647	0.777755298	-0.133
cg01997813	I	37	2	242710939		0.71	4.57E-06	5.34042	0.76591373	0.898590024	-0.133
cg01832712	II	37	3	4910674		0.46	0.01197	1.92207	0.21277128	0.34593605	-0.133
cg02316506	II	37	3	42694803	ZBTB47	0.46	0.01197	1.92207	0.40565225	0.538575436	-0.133
cg06240200	II	37	3	46974965	CCDC12	0.54	0.0014	2.85345	0.81616344	0.682694574	0.133
cg17546247	II	37	3	147125714	ZIC4;ZIC1	0.67	2.34E-05	4.63072	0.34582994	0.47893824	-0.133
cg07064537	II	37	3	171515212	PLD1;PLD1	0.54	0.0014	2.85345	0.6968605	0.563995625	0.133
cg01310397	II	37	3	195490033	MUC4;MUC4;MUC4	0.54	0.0014	2.85345	0.73521091	0.867824231	-0.133
cg26860935	I	37	4	1407989		0.58	0.00041	3.39184	0.12788987	0.261113599	-0.133
cg11168104	I	37	5	1857477		0.54	0.0014	2.85345	0.64804331	0.781080345	-0.133
cg26655340	II	37	5	154230141	C5orf4;C5orf4	0.46	0.01197	1.92207	0.20025119	0.333578635	-0.133
cg23963071	II	37	6	2901712	SERPINB9	0.5	0.00432	2.36443	0.61455325	0.481713597	0.133
cg06499368	II	37	6	6725372		0.54	0.0014	2.85345	0.6493233	0.782439696	-0.133
cg06473288	II	37	6	32820102	TAP1	0.46	0.01197	1.92207	0.80398257	0.671025708	0.133
cg06885468	II	37	6	158129101		0.62	0.0001	3.98291	0.51329498	0.646017092	-0.133
cg08352774	II	37	6	158985825	TMEM181	0.42	0.02991	1.52413	0.16203725	0.295281919	-0.133
cg02072875	II	37	7	2606796	IQCE;IQCE	0.5	0.00432	2.36443	0.80960312	0.676787807	0.133
cg00232092	II	37	7	5518887	FBXL18	0.46	0.01197	1.92207	0.58962097	0.722376724	-0.133
cg26966828	I	37	8	10208257	MSRA;MSRA;MSRA	0.42	0.02991	1.52413	0.07865419	0.21209538	-0.133
cg02929961	II	37	8	22525659	BIN3	0.5	0.00432	2.36443	0.77089516	0.637767034	0.133
cg16625218	II	37	8	30272502	RBPMS;RBPMS;RBP	0.46	0.01197	1.92207	0.66112384	0.528111858	0.133
cg15450349	I	37	8	42269042		0.58	0.00041	3.39184	0.75307923	0.885761179	-0.133
cg10806146	I	37	8	42356871	SLC20A2	0.5	0.00432	2.36443	0.14830195	0.2809467	-0.133
cg14424376	II	37	9	98513318		0.54	0.0014	2.85345	0.64758385	0.514705897	0.133
cg13403259	II	37	9	123145884		0.5	0.00432	2.36443	0.61931184	0.486805841	0.133
cg14446165	I	37	9	136833899	VAV2;VAV2	0.58	0.00041	3.39184	0.72034057	0.587396847	0.133
cg14186779	II	37	9	139243399	GPSM1	0.42	0.02991	1.52413	0.54294002	0.410291632	0.133
cg18666379	II	37	10	44503867		0.46	0.01197	1.92207	0.80980087	0.677009756	0.133
cg09924848	II	37	10	126308486	FAM53B	0.46	0.01197	1.92207	0.47728081	0.343954288	0.133
cg16544463	I	37	10	134407942	INPP5A	0.75	7.61E-07	6.11857	0.45833522	0.324860986	0.133
cg26241460	II	37	11	1767775	HCCA2;LOC402778	0.54	0.0014	2.85345	0.67847563	0.811172678	-0.133
cg04085571	I	37	11	1872753	LSP1	0.54	0.0014	2.85345	0.60782508	0.475121841	0.133
cg12151545	II	37	11	47373000	MYBPC3	0.54	0.0014	2.85345	0.65595505	0.789091505	-0.133

cg11439695	II	37	12	2561024	CACNA1C;CACNA1(	0.5	0.00432	2.36443	0.67622234	0.543308747	0.133
cg05484458	II	37	12	6949260	GNB3	0.5	0.00432	2.36443	0.6672109	0.53447012	0.133
cg06872313	I	37	12	14413185		0.5	0.00432	2.36443	0.43132113	0.564552651	-0.133
cg13696135	II	37	12	49396782	PRKAG1;PRKAG1	0.42	0.02991	1.52413	0.71254471	0.579138335	0.133
cg02317313	I	37	12	122235206	LOC338799	0.62	0.0001	3.98291	0.7164051	0.849241225	-0.133
cg05336395	I	37	13	53421688	PCDH8;PCDH8	0.5	0.00432	2.36443	0.29624591	0.428982803	-0.133
cg11997468	I	37	13	114834922	RASA3	0.54	0.0014	2.85345	0.65188613	0.785153364	-0.133
cg13038618	II	37	14	77467391		0.42	0.02991	1.52413	0.29389979	0.160782652	0.133
cg00905584	I	37	14	105058234		0.46	0.01197	1.92207	0.77082091	0.638243803	0.133
cg08959144	II	37	16	29141694		0.46	0.01197	1.92207	0.34111459	0.474511642	-0.133
cg10104480	II	37	16	72955250	ZFHX3;ZFHX3	0.54	0.0014	2.85345	0.34484174	0.478064675	-0.133
cg02637247	II	37	16	86524562		0.5	0.00432	2.36443	0.66223958	0.529139841	0.133
cg08565003	I	37	16	87447920	ZCCHC14	0.58	0.00041	3.39184	0.8645534	0.731970269	0.133
cg00031162	II	37	17	7453377	TNFSF12;TNFSF12-T	0.46	0.01197	1.92207	0.72662948	0.594100783	0.133
cg05508862	II	37	17	18885437	SLC5A10;FAM83G;S	0.54	0.0014	2.85345	0.41811389	0.550911749	-0.133
cg24069602	I	37	17	62045522	SCN4A	0.46	0.01197	1.92207	0.70042418	0.567624736	0.133
cg16736055	II	37	17	72028714		0.58	0.00041	3.39184	0.7920197	0.659351397	0.133
cg06094707	II	37	17	78445867	NPTX1	0.54	0.0014	2.85345	0.44020996	0.307641684	0.133
cg01810575	II	37	17	79415682	BAHCC1	0.42	0.02991	1.52413	0.42998598	0.297067518	0.133
cg17069873	II	37	19	40732784	CNTD2	0.62	0.0001	3.98291	0.36862133	0.501691861	-0.133
cg13407883	II	37	19	51627843	SIGLEC9	0.54	0.0014	2.85345	0.68051814	0.813803676	-0.133
cg18471488	I	37	20	30105550	HM13;HM13;HM13	0.67	2.34E-05	4.63072	0.51521812	0.648568653	-0.133
cg05085169	I	37	20	61443751	OGFR	0.54	0.0014	2.85345	0.81881815	0.952301476	-0.133
cg14505256	II	37	22	20978594		0.58	0.00041	3.39184	0.81517621	0.682409165	0.133
cg04392554	I	37	22	46685472	TTC38	0.5	0.00432	2.36443	0.95721438	0.824340652	0.133
cg20995573	II	37	22	51038379	MAPK8IP2	0.67	2.34E-05	4.63072	0.1765756	0.309727405	-0.133
cg22123711	II	37	1	12185845	TNFRSF8;TNFRSF8	0.54	0.0014	2.85345	0.68694712	0.819076821	-0.132
cg00664416	I	37	1	38261192	MANEAL;MANEAL;I	0.5	0.00432	2.36443	0.27794058	0.409594951	-0.132
cg03884592	II	37	1	42384474	HIVEP3;HIVEP3;HIV	0.42	0.02991	1.52413	0.22939352	0.361841789	-0.132
cg13390284	II	37	1	65531864		0.46	0.01197	1.92207	0.58443203	0.716550163	-0.132
cg26833883	II	37	1	154965572	FLAD1;LENEP;FLAD	0.5	0.00432	2.36443	0.30669915	0.438488215	-0.132
cg21022868	II	37	1	159923861	SLAMF9;SLAMF9;SL	0.54	0.0014	2.85345	0.70121755	0.569687187	0.132
cg03040292	II	37	1	200847096		0.42	0.02991	1.52413	0.75155324	0.619118544	0.132

cg26841040	II	37	1	228332263	GUK1;GUK1;GUK1	0.42	0.02991	1.52413	0.47913855	0.347223232	0.132
cg12167564	II	37	1	236047034		0.46	0.01197	1.92207	0.49419963	0.626032792	-0.132
cg14021880	II	37	2	27301369	EMILIN1	0.67	2.34E-05	4.63072	0.57014816	0.702369905	-0.132
cg23432368	II	37	2	47882601		0.5	0.00432	2.36443	0.62960505	0.497173518	0.132
cg03198066	II	37	2	159311068	CCDC148;CCDC148	0.54	0.0014	2.85345	0.3260031	0.458300539	-0.132
cg09128944	II	37	2	169967580		0.62	0.0001	3.98291	0.27887196	0.411062537	-0.132
cg10004897	II	37	4	7054700	TADA2B	0.54	0.0014	2.85345	0.22471669	0.356848502	-0.132
cg10198664	II	37	4	83631584	SCD5;SCD5	0.54	0.0014	2.85345	0.64376042	0.512120441	0.132
cg09473510	I	37	4	187476573	MTNR1A	0.42	0.02991	1.52413	0.08192677	0.213450163	-0.132
cg04654716	II	37	5	74162924	FAM169A	0.71	4.57E-06	5.34042	0.42911175	0.560820292	-0.132
cg19459094	II	37	5	176856845	GRK6;GRK6;GRK6	0.42	0.02991	1.52413	0.22308161	0.354983818	-0.132
cg14674856	II	37	5	179220274	LTC4S	0.62	0.0001	3.98291	0.47207298	0.603764143	-0.132
cg10126181	II	37	6	21832051	FLJ22536	0.5	0.00432	2.36443	0.17069077	0.302854599	-0.132
cg17604312	I	37	6	30850829	DDR1;DDR1	0.5	0.00432	2.36443	0.58708801	0.454794784	0.132
cg21550612	II	37	6	31094185	PSORS1C1	0.54	0.0014	2.85345	0.5538006	0.421705527	0.132
cg01928820	II	37	6	42883624	PTCRA	0.54	0.0014	2.85345	0.31816068	0.185686298	0.132
cg14994513	II	37	6	146919932	C6orf103	0.5	0.00432	2.36443	0.19340985	0.325031573	-0.132
cg00775915	II	37	7	580703		0.42	0.02991	1.52413	0.55450492	0.422581468	0.132
cg14405813	II	37	7	139414573	HIPK2;HIPK2	0.46	0.01197	1.92207	0.39075629	0.258385531	0.132
cg15901999	II	37	8	41682580	ANK1	0.58	0.00041	3.39184	0.72760064	0.859505602	-0.132
cg08598221	II	37	8	121824929	SNTB1	0.58	0.00041	3.39184	0.43830227	0.570520135	-0.132
cg13738729	II	37	9	36989127	PAX5	0.46	0.01197	1.92207	0.35735346	0.225115849	0.132
cg14473838	II	37	9	131490040		0.58	0.00041	3.39184	0.59156661	0.459360587	0.132
cg12754854	II	37	9	131682859	PHYHD1;PHYHD1	0.5	0.00432	2.36443	0.7894128	0.657181228	0.132
cg16885557	II	37	10	48601822		0.5	0.00432	2.36443	0.81325349	0.681126505	0.132
cg19207017	II	37	10	104316063	SUFU	0.5	0.00432	2.36443	0.75985019	0.891535464	-0.132
cg25030266	II	37	10	106077741	ITPRIP	0.42	0.02991	1.52413	0.60286312	0.471218634	0.132
cg23999801	II	37	10	126254661	LHPP;LHPP	0.46	0.01197	1.92207	0.53853785	0.406857079	0.132
cg27209072	II	37	10	127702584		0.58	0.00041	3.39184	0.71163701	0.58009153	0.132
cg00476955	II	37	11	3648013	TRPC2	0.58	0.00041	3.39184	0.25315922	0.385247398	-0.132
cg09781307	II	37	11	27648324	BDNFOS	0.5	0.00432	2.36443	0.69730952	0.565212569	0.132
cg16177481	II	37	11	61740158		0.54	0.0014	2.85345	0.19942873	0.331141407	-0.132
cg10465696	II	37	11	124767948	ROBO4	0.5	0.00432	2.36443	0.07736933	0.209266529	-0.132

cg07690768	II	37	12	32370933	BICD1;BICD1	0.58	0.00041	3.39184	0.69115791	0.82359756	-0.132
cg00026033	I	37	12	56414490	IKZF4	0.46	0.01197	1.92207	0.3833448	0.515627891	-0.132
cg26244575	II	37	12	76354015		0.46	0.01197	1.92207	0.56802766	0.436476826	0.132
cg00357958	I	37	12	123215010	GPR81;GPR81	0.5	0.00432	2.36443	0.63950507	0.507067281	0.132
cg07675647	II	37	12	125359265		0.54	0.0014	2.85345	0.29082035	0.422543382	-0.132
cg10583311	II	37	12	133082545	FBRSL1	0.67	2.34E-05	4.63072	0.57306445	0.705396231	-0.132
cg17574785	II	37	13	20933879		0.42	0.02991	1.52413	0.7089	0.577232342	0.132
cg26609631	I	37	13	28366814	GSX1;GSX1	0.42	0.02991	1.52413	0.07193823	0.203827616	-0.132
cg14332524	II	37	14	65770225		0.5	0.00432	2.36443	0.78028194	0.648685947	0.132
cg13152690	II	37	14	91695017		0.5	0.00432	2.36443	0.58287649	0.450952091	0.132
cg10040594	I	37	14	93556554	ITPK1;ITPK1;ITPK1	0.46	0.01197	1.92207	0.65764686	0.525485698	0.132
cg10319640	II	37	14	95090384	SERPINA3	0.58	0.00041	3.39184	0.71460646	0.582788116	0.132
cg07150062	I	37	14	104552032	ASPG	0.46	0.01197	1.92207	0.09135278	0.223435695	-0.132
cg16050468	I	37	14	105011965		0.46	0.01197	1.92207	0.68724045	0.819504394	-0.132
cg16279237	I	37	14	106174668		0.46	0.01197	1.92207	0.87918085	0.747275439	0.132
cg05852824	II	37	15	26543817		0.46	0.01197	1.92207	0.69292556	0.561355038	0.132
cg07994696	II	37	16	11680075	LITAF;LITAF;LITAF;L	0.58	0.00041	3.39184	0.52261692	0.390187986	0.132
cg09685060	I	37	16	28621286	SULT1A1;SULT1A1;S	0.58	0.00041	3.39184	0.50854645	0.640173845	-0.132
cg09889350	II	37	16	56995813	CETP	0.67	2.34E-05	4.63072	0.52153138	0.653721305	-0.132
cg27229664	II	37	16	85096666	KIAA0513	0.46	0.01197	1.92207	0.56052287	0.692822558	-0.132
cg07450021	II	37	16	85097151	KIAA0513	0.5	0.00432	2.36443	0.67354595	0.805489711	-0.132
cg01830154	II	37	17	1134911		0.5	0.00432	2.36443	0.29123753	0.423659741	-0.132
cg03572260	II	37	17	37808339	STARD3;STARD3;ST	0.46	0.01197	1.92207	0.73081691	0.862917086	-0.132
cg04988978	II	37	17	56359578	MPO	0.46	0.01197	1.92207	0.2792629	0.410856657	-0.132
cg14691671	II	37	17	73642503	RECQL5;LOC10013(	0.58	0.00041	3.39184	0.3884456	0.520660497	-0.132
cg20923885	II	37	17	75473610	SEPT9;SEPT9;SEPT9	0.46	0.01197	1.92207	0.62247892	0.754616441	-0.132
cg18826274	II	37	17	76335184		0.46	0.01197	1.92207	0.60638425	0.474247248	0.132
cg12654199	II	37	17	78753826	RPTOR;RPTOR	0.58	0.00041	3.39184	0.74626885	0.87870176	-0.132
cg12593411	II	37	19	10213722	ANGPTL6	0.42	0.02991	1.52413	0.37038814	0.238473051	0.132
cg03272225	II	37	19	17957106	JAK3	0.5	0.00432	2.36443	0.20002972	0.331843364	-0.132
cg05983315	II	37	19	58545837	ZSCAN1	0.58	0.00041	3.39184	0.14263262	0.274934378	-0.132
cg14251267	II	37	20	62406428	ZBTB46	0.46	0.01197	1.92207	0.65148097	0.783916965	-0.132
cg23163200	II	37	21	34186122	C21orf62;C21orf62	0.58	0.00041	3.39184	0.25155453	0.383711669	-0.132

cg27477277	II	37	21	45811432	TRPM2	0.54	0.0014	2.85345	0.63038836	0.762085748	-0.132
cg00495443	II	37	22	17568163	IL17RA	0.5	0.00432	2.36443	0.69939312	0.831098137	-0.132
cg18576206	II	37	22	44392384	SAMM50	0.46	0.01197	1.92207	0.31714941	0.185392705	0.132
cg08471335	II	37	22	46685653	TTC38	0.42	0.02991	1.52413	0.72632258	0.594290045	0.132
cg08469752	II	37	22	50243719		0.42	0.02991	1.52413	0.19695949	0.32862969	-0.132
cg23606775	II	37	1	9790616	CLSTN1;CLSTN1	0.46	0.01197	1.92207	0.59645926	0.72727176	-0.131
cg26528311	II	37	1	38462546	FHL3	0.46	0.01197	1.92207	0.27454486	0.405671552	-0.131
cg01804134	II	37	1	44965140	RNF220	0.42	0.02991	1.52413	0.33407707	0.464888693	-0.131
cg12160664	II	37	1	114521853	OLFML3	0.46	0.01197	1.92207	0.42545362	0.55677554	-0.131
cg09791504	II	37	1	151701849	C1orf230	0.5	0.00432	2.36443	0.53122253	0.400050993	0.131
cg09036468	I	37	1	151806893		0.54	0.0014	2.85345	0.33432868	0.465536357	-0.131
cg13098855	II	37	1	153588927	S100A14	0.67	2.34E-05	4.63072	0.68276116	0.814023377	-0.131
cg17711527	II	37	1	203734396	LAX1;LAX1;LAX1;LA	0.5	0.00432	2.36443	0.46287597	0.331559496	0.131
cg00609227	II	37	1	226307233		0.54	0.0014	2.85345	0.51036578	0.378925046	0.131
cg15358723	II	37	1	245750662	KIF26B	0.46	0.01197	1.92207	0.64081829	0.509449654	0.131
cg10960375	II	37	3	42694144	ZBTB47	0.5	0.00432	2.36443	0.30086008	0.431616872	-0.131
cg25445707	II	37	3	46719086	ALS2CL;ALS2CL	0.67	2.34E-05	4.63072	0.53984241	0.670463825	-0.131
cg23487312	II	37	3	62401106	CADPS;CADPS;CADI	0.46	0.01197	1.92207	0.42312244	0.291640192	0.131
cg12226735	II	37	3	98610584	DCBLD2	0.5	0.00432	2.36443	0.78765137	0.656210219	0.131
cg09010699	II	37	3	195171693		0.58	0.00041	3.39184	0.76732412	0.89843369	-0.131
cg05618934	I	37	4	1407592		0.54	0.0014	2.85345	0.44577245	0.577073251	-0.131
cg06851151	II	37	5	133436368		0.67	2.34E-05	4.63072	0.46831335	0.337001856	0.131
cg14873515	II	37	5	149887461	NDST1	0.54	0.0014	2.85345	0.60995179	0.741037926	-0.131
cg22646937	II	37	5	169816792	KCNMB1;KCNIP1	0.54	0.0014	2.85345	0.45933865	0.589955596	-0.131
cg05869491	II	37	6	209763		0.5	0.00432	2.36443	0.73943696	0.608729308	0.131
cg13064679	I	37	6	32049953	TNXB	0.46	0.01197	1.92207	0.72066858	0.58928825	0.131
cg06032479	I	37	6	32552026	HLA-DRB1	0.46	0.01197	1.92207	0.64890997	0.779733673	-0.131
cg05460965	II	37	6	36645100	CDKN1A;CDKN1A	0.54	0.0014	2.85345	0.56955422	0.701021839	-0.131
cg07436074	II	37	6	162071140	PARK2;PARK2;PARK	0.5	0.00432	2.36443	0.64863609	0.517273283	0.131
cg05788125	II	37	6	170559721		0.62	0.0001	3.98291	0.5066021	0.637799315	-0.131
cg23597162	II	37	7	28102341	JAZF1	0.5	0.00432	2.36443	0.69927546	0.830273957	-0.131
cg11961845	II	37	7	129008179	AHCYL2;AHCYL2;AH	0.5	0.00432	2.36443	0.2220481	0.352699915	-0.131
cg20380768	II	37	7	130125511	MEST	0.5	0.00432	2.36443	0.79184909	0.661274498	0.131

cg07963670	II	37	8	23161764	LOXL2	0.5	0.00432	2.36443	0.4095778	0.27848539	0.131
cg15936066	II	37	8	42356637	SLC20A2	0.5	0.00432	2.36443	0.2900511	0.421229371	-0.131
cg20076442	II	37	8	72745197		0.54	0.0014	2.85345	0.41097674	0.542127006	-0.131
cg17783213	II	37	8	139883385	COL22A1	0.46	0.01197	1.92207	0.75644427	0.625228811	0.131
cg12073251	II	37	8	141568652	EIF2C2;EIF2C2	0.46	0.01197	1.92207	0.46551891	0.597007486	-0.131
cg08962185	II	37	8	143336052	TSNARE1	0.5	0.00432	2.36443	0.2790294	0.14837846	0.131
cg14390143	II	37	9	97095135		0.5	0.00432	2.36443	0.47736745	0.608162183	-0.131
cg23016776	II	37	10	99081496	FRAT1;FRAT1	0.42	0.02991	1.52413	0.4403839	0.571466657	-0.131
cg09504612	II	37	10	116467988		0.5	0.00432	2.36443	0.68745833	0.556525885	0.131
cg05971678	I	37	10	125770089	CHST15	0.67	2.34E-05	4.63072	0.81934334	0.950695402	-0.131
cg23202388	II	37	11	914849	CHID1;CHID1	0.79	1.06E-07	6.97389	0.61775708	0.74834295	-0.131
cg08066376	II	37	11	59577361	MRPL16	0.67	2.34E-05	4.63072	0.45237773	0.583714729	-0.131
cg23220439	II	37	11	66104481	RIN1	0.54	0.0014	2.85345	0.6346364	0.503189532	0.131
cg08870587	I	37	11	70455278	SHANK2;SHANK2	0.58	0.00041	3.39184	0.69471321	0.825996874	-0.131
cg05975710	II	37	11	73078836	ARHGEF17	0.58	0.00041	3.39184	0.48686595	0.355855043	0.131
cg01212326	II	37	11	111250286	POU2AF1	0.54	0.0014	2.85345	0.22938224	0.098664353	0.131
cg07690882	II	37	11	128694303		0.5	0.00432	2.36443	0.25755312	0.38868643	-0.131
cg01511480	II	37	12	6471251	SCNN1A;SCNN1A;S(	0.62	0.0001	3.98291	0.67629799	0.807755071	-0.131
cg23348081	II	37	12	14413690		0.5	0.00432	2.36443	0.66079559	0.792116255	-0.131
cg13984040	II	37	12	125258948		0.54	0.0014	2.85345	0.34668106	0.477304714	-0.131
cg06555661	II	37	12	132259779	SFRS8	0.42	0.02991	1.52413	0.87164313	0.740289452	0.131
cg03992114	II	37	13	113343376	ATP11A;ATP11A	0.46	0.01197	1.92207	0.33721179	0.468196636	-0.131
cg02181506	II	37	14	94856984	SERPINA1;SERPINA	0.58	0.00041	3.39184	0.22111661	0.352363476	-0.131
cg15933120	II	37	14	102102737		0.42	0.02991	1.52413	0.76673561	0.635422646	0.131
cg19994779	II	37	14	105482622	CDCA4;CDCA4	0.58	0.00041	3.39184	0.38825546	0.519208669	-0.131
cg15144068	II	37	14	106321751		0.58	0.00041	3.39184	0.6389839	0.508316203	0.131
cg11869499	II	37	15	89872984	POLG;POLG	0.42	0.02991	1.52413	0.30114133	0.432516258	-0.131
cg05515414	II	37	16	21511211	LOC100271836	0.54	0.0014	2.85345	0.25489539	0.124193009	0.131
cg09529437	II	37	16	24136792	PRKCB;PRKCB	0.46	0.01197	1.92207	0.29533497	0.426030752	-0.131
cg26907472	II	37	16	69006828	TMCO7	0.58	0.00041	3.39184	0.65717474	0.787885922	-0.131
cg20021790	II	37	17	181288	RPH3AL	0.5	0.00432	2.36443	0.37508692	0.506307495	-0.131
cg17224892	II	37	17	1094496		0.58	0.00041	3.39184	0.39609247	0.527199006	-0.131
cg11964099	II	37	17	3905835		0.5	0.00432	2.36443	0.55152854	0.682375266	-0.131

cg14692950	II	37	17	7644073	DNAH2	0.42	0.02991	1.52413	0.65665673	0.787893784	-0.131
cg21187669	II	37	17	17929033	ATPAF2	0.5	0.00432	2.36443	0.55884491	0.69009964	-0.131
cg21605283	II	37	17	30615925	RHBDL3	0.42	0.02991	1.52413	0.21984304	0.351103461	-0.131
cg01890568	I	37	17	31437649	ACCN1;ACCN1	0.5	0.00432	2.36443	0.83858439	0.707908989	0.131
cg11420633	II	37	17	39150330	KRTAP3-3	0.5	0.00432	2.36443	0.53553971	0.404972263	0.131
cg16672637	II	37	17	74138356	FOXJ1	0.54	0.0014	2.85345	0.48702867	0.618093102	-0.131
cg09803959	II	37	17	78865514	RPTOR;RPTOR	0.5	0.00432	2.36443	0.230223	0.361209126	-0.131
cg17831694	I	37	17	78929900	RPTOR;RPTOR	0.5	0.00432	2.36443	0.47082288	0.340048234	0.131
cg26985878	I	37	17	79799549		0.58	0.00041	3.39184	0.77355207	0.904201816	-0.131
cg23881099	I	37	17	79924020		0.46	0.01197	1.92207	0.70726289	0.576516818	0.131
cg10310700	I	37	17	80872461	TBCD	0.58	0.00041	3.39184	0.57321202	0.441811369	0.131
cg14758525	II	37	18	12041672		0.46	0.01197	1.92207	0.52307046	0.653893614	-0.131
cg05165553	I	37	18	77171010	NFATC1;NFATC1;NF	0.42	0.02991	1.52413	0.71716919	0.848376057	-0.131
cg19865472	I	37	19	617133	HCN2	0.42	0.02991	1.52413	0.84429876	0.713530931	0.131
cg23357789	I	37	19	848026	PRTN3	0.42	0.02991	1.52413	0.39253687	0.523361069	-0.131
cg04848343	I	37	19	4544095	SEMA6B	0.5	0.00432	2.36443	0.7638417	0.894991393	-0.131
cg07543883	II	37	19	10213271	ANGPTL6;ANGPTL6	0.42	0.02991	1.52413	0.68132574	0.550301396	0.131
cg14592928	II	37	20	897600	ANGPT4	0.42	0.02991	1.52413	0.78399561	0.653039296	0.131
cg21001667	II	37	20	32954013	ITCH	0.5	0.00432	2.36443	0.27982762	0.410788005	-0.131
cg26206598	II	37	20	47445432	PREX1	0.42	0.02991	1.52413	0.50749218	0.638341447	-0.131
cg22990871	II	37	21	43377577		0.58	0.00041	3.39184	0.34076092	0.471346172	-0.131
cg17939585	II	37	22	50747040	PLXNB2	0.5	0.00432	2.36443	0.35373087	0.4848791	-0.131
cg17109725	II	37	1	18707479		0.54	0.0014	2.85345	0.5408079	0.410539271	0.13
cg22125902	II	37	1	26002535	MAN1C1	0.58	0.00041	3.39184	0.58070862	0.451141565	0.13
cg10710218	II	37	1	55012989	ACOT11;ACOT11	0.5	0.00432	2.36443	0.3841626	0.514147415	-0.13
cg21448423	II	37	1	55013956	ACOT11;ACOT11;A(	0.42	0.02991	1.52413	0.49857239	0.62853427	-0.13
cg16269755	II	37	1	151586058	SNX27	0.54	0.0014	2.85345	0.62486213	0.755203026	-0.13
cg20535085	II	37	1	160616604	SLAMF1	0.5	0.00432	2.36443	0.66350302	0.533029521	0.13
cg03421657	II	37	1	166039754	FAM78B	0.58	0.00041	3.39184	0.75861541	0.628851156	0.13
cg12298872	II	37	1	228352449	C1orf69	0.58	0.00041	3.39184	0.67611589	0.806602898	-0.13
cg14209730	II	37	2	64632636		0.5	0.00432	2.36443	0.77266857	0.902451484	-0.13
cg12833765	II	37	2	69470878	ANTXR1	0.5	0.00432	2.36443	0.76808117	0.638474468	0.13
cg24803346	II	37	2	73201477	SFXN5	0.62	0.0001	3.98291	0.56501735	0.695324804	-0.13

cg11344744	II	37	2	97526122	SEMA4C	0.58	0.00041	3.39184	0.44812908	0.578343583	-0.13
cg25725843	II	37	2	107504169	ST6GAL2;ST6GAL2	0.62	0.0001	3.98291	0.25202678	0.381800809	-0.13
cg14102128	I	37	2	110371632	SEPT10;SEPT10;SEP	0.5	0.00432	2.36443	0.08032827	0.210281907	-0.13
cg02551646	II	37	2	216478293		0.46	0.01197	1.92207	0.76194541	0.631955628	0.13
cg13877916	II	37	2	240499800		0.54	0.0014	2.85345	0.68866463	0.819000987	-0.13
cg11184109	II	37	3	53164962	RFT1	0.46	0.01197	1.92207	0.30065713	0.430895167	-0.13
cg15321908	II	37	3	53187213		0.58	0.00041	3.39184	0.61487812	0.74455513	-0.13
cg10464130	II	37	3	123991925	KALRN;KALRN	0.54	0.0014	2.85345	0.6181441	0.487966292	0.13
cg00218914	II	37	3	129146731	C3orf25	0.54	0.0014	2.85345	0.28490411	0.415354774	-0.13
cg14875327	I	37	3	197081654		0.58	0.00041	3.39184	0.82091028	0.69091503	0.13
cg17330176	II	37	4	2389058	ZFYVE28	0.67	2.34E-05	4.63072	0.6668678	0.796468581	-0.13
cg05012661	II	37	4	6891264		0.42	0.02991	1.52413	0.46425045	0.593978482	-0.13
cg03999941	I	37	5	957511		0.46	0.01197	1.92207	0.72911642	0.599497081	0.13
cg07019857	II	37	5	106822849	EFNA5	0.5	0.00432	2.36443	0.64763726	0.777989401	-0.13
cg16104139	I	37	5	134829480		0.58	0.00041	3.39184	0.53649105	0.666617919	-0.13
cg14741236	II	37	5	171985354		0.46	0.01197	1.92207	0.73437617	0.60438299	0.13
cg05871756	II	37	5	173216171		0.5	0.00432	2.36443	0.35350634	0.483869459	-0.13
cg24686957	II	37	6	5214950	LYRM4;LYRM4;LYRI	0.54	0.0014	2.85345	0.76554128	0.895130479	-0.13
cg19917989	II	37	6	14925093		0.46	0.01197	1.92207	0.60548559	0.735254704	-0.13
cg05085500	II	37	6	19692172		0.54	0.0014	2.85345	0.13518606	0.265556519	-0.13
cg24994319	II	37	6	25027586		0.58	0.00041	3.39184	0.26868624	0.138802318	0.13
cg02680487	I	37	6	30851529	DDR1;DDR1	0.58	0.00041	3.39184	0.40598984	0.275978787	0.13
cg05631194	II	37	6	31146417	PSORS1C3	0.46	0.01197	1.92207	0.80913102	0.67891996	0.13
cg10552964	II	37	6	35991802	SLC26A8;SLC26A8	0.5	0.00432	2.36443	0.42521362	0.555283843	-0.13
cg12649238	II	37	6	36669758		0.67	2.34E-05	4.63072	0.73565875	0.865934288	-0.13
cg25318189	II	37	7	2800436	GNA12	0.5	0.00432	2.36443	0.37466727	0.504299049	-0.13
cg01322214	II	37	7	25219198	C7orf31	0.5	0.00432	2.36443	0.39002005	0.520417015	-0.13
cg18202127	I	37	7	36061063		0.46	0.01197	1.92207	0.76457275	0.634486856	0.13
cg01973676	I	37	7	101596404	CUX1;CUX1;CUX1	0.54	0.0014	2.85345	0.15715887	0.287403361	-0.13
cg22103219	II	37	7	101934892	SH2B2	0.58	0.00041	3.39184	0.31376267	0.183911112	0.13
cg09159022	II	37	8	4849522	CSMD1	0.58	0.00041	3.39184	0.17558558	0.30535926	-0.13
cg06076277	II	37	8	49783056		0.54	0.0014	2.85345	0.18616982	0.316018639	-0.13
cg13874498	II	37	9	132247010		0.42	0.02991	1.52413	0.81476652	0.685020169	0.13

cg18886109	II	37	10	72514348	ADAMTS14;ADAMT	0.58	0.00041	3.39184	0.40063053	0.530480998	-0.13
cg16933922	II	37	10	126106614	OAT	0.54	0.0014	2.85345	0.16577372	0.296132071	-0.13
cg07244202	II	37	10	127750970	ADAM12;ADAM12	0.58	0.00041	3.39184	0.61915388	0.489408742	0.13
cg01512840	I	37	10	134258819	C10orf91	0.46	0.01197	1.92207	0.17821899	0.308544496	-0.13
cg10042645	II	37	11	2308589		0.54	0.0014	2.85345	0.72441001	0.854074963	-0.13
cg16075139	I	37	11	2406923	CD81	0.58	0.00041	3.39184	0.55295566	0.422765032	0.13
cg16587707	II	37	11	2920265	SLC22A18AS;SLC22	0.75	7.61E-07	6.11857	0.54234678	0.672263543	-0.13
cg23272978	I	37	11	65315030	LTBP3;LTBP3;LTBP3	0.67	2.34E-05	4.63072	0.84232861	0.712441251	0.13
cg14562054	II	37	11	66104485	RIN1	0.46	0.01197	1.92207	0.62804529	0.497980246	0.13
cg23756264	II	37	11	68605511	CPT1A;CPT1A	0.5	0.00432	2.36443	0.2287711	0.358299951	-0.13
cg07322003	II	37	11	76384620		0.46	0.01197	1.92207	0.7524885	0.622424309	0.13
cg15376097	II	37	11	118135270	MPZL2;MPZL2	0.46	0.01197	1.92207	0.3239598	0.453996784	-0.13
cg11606444	I	37	11	121353563	SORL1	0.62	0.0001	3.98291	0.53172946	0.401424346	0.13
cg13422817	II	37	12	4550927	FGF6	0.46	0.01197	1.92207	0.49992908	0.370332973	0.13
cg05405094	II	37	12	20141722		0.54	0.0014	2.85345	0.68924216	0.559342	0.13
cg09001549	I	37	12	129281454	SLC15A4	0.46	0.01197	1.92207	0.85272124	0.722225619	0.13
cg26885400	II	37	13	27842278		0.5	0.00432	2.36443	0.41631823	0.546605541	-0.13
cg09624466	II	37	14	57278710	OTX2OS1	0.58	0.00041	3.39184	0.14465031	0.274208921	-0.13
cg23305899	I	37	14	101539489		0.58	0.00041	3.39184	0.83808033	0.708190092	0.13
cg02513364	II	37	14	103373997		0.54	0.0014	2.85345	0.67621035	0.546205141	0.13
cg04715649	II	37	14	103416122	CDC42BPB	0.58	0.00041	3.39184	0.75169139	0.621800984	0.13
cg08334034	II	37	15	93615039	RGMA;RGMA;RGM	0.46	0.01197	1.92207	0.59130284	0.720814495	-0.13
cg04330371	I	37	15	96875656	NR2F2;NR2F2;NR2F	0.46	0.01197	1.92207	0.17791594	0.308289843	-0.13
cg00003900	II	37	16	2897074		0.42	0.02991	1.52413	0.55046236	0.420260986	0.13
cg07906046	II	37	16	4131584	ADCY9	0.46	0.01197	1.92207	0.5425705	0.412376049	0.13
cg02106534	II	37	16	4561545	C16orf5	0.5	0.00432	2.36443	0.36829664	0.498080674	-0.13
cg26706803	II	37	16	50293530		0.42	0.02991	1.52413	0.19069415	0.320271536	-0.13
cg16771652	II	37	16	50730385	NOD2	0.62	0.0001	3.98291	0.33809913	0.467646969	-0.13
cg06178179	II	37	16	66881892	CA7;CA7	0.46	0.01197	1.92207	0.68848852	0.81861743	-0.13
cg18520696	II	37	17	4631822		0.54	0.0014	2.85345	0.8620497	0.732479464	0.13
cg23545250	II	37	17	6348091	FAM64A	0.67	2.34E-05	4.63072	0.29017693	0.42044118	-0.13
cg25322720	II	37	17	19628292		0.58	0.00041	3.39184	0.25958715	0.389485004	-0.13
cg02650512	II	37	17	27048975	RPL23A;SNORD4A;S	0.46	0.01197	1.92207	0.74507088	0.614988781	0.13

cg22589778	II	37	17	46059110	CDK5RAP3	0.5	0.00432	2.36443	0.2830991	0.412982811	-0.13
cg10165801	I	37	17	46651186	HOXB3	0.42	0.02991	1.52413	0.3285924	0.458916294	-0.13
cg05941027	II	37	17	61774174	LIMD2	0.5	0.00432	2.36443	0.26688387	0.136394697	0.13
cg16375358	II	37	17	64873337	CACNG5;CACNG5	0.54	0.0014	2.85345	0.74063674	0.610898147	0.13
cg00900735	I	37	17	73316505	GRB2;GRB2	0.42	0.02991	1.52413	0.85826278	0.728591439	0.13
cg23019125	II	37	17	78820391	RPTOR;RPTOR	0.46	0.01197	1.92207	0.5203465	0.390178919	0.13
cg11220060	II	37	19	12997418	KLF1	0.54	0.0014	2.85345	0.45452642	0.584204301	-0.13
cg01542019	I	37	19	14673053	TECR	0.42	0.02991	1.52413	0.47079539	0.341288369	0.13
cg10614223	II	37	19	18111389	ARRDC2	0.62	0.0001	3.98291	0.51603576	0.385979574	0.13
cg01743841	I	37	20	61992494	CHRNA4	0.42	0.02991	1.52413	0.04407186	0.173860999	-0.13
cg15557168	I	37	22	42548783		0.62	0.0001	3.98291	0.75164503	0.881501839	-0.13
cg00007800	II	37	1	1838518		0.42	0.02991	1.52413	0.6681408	0.539247322	0.129
cg26332488	I	37	1	2230640	SKI	0.42	0.02991	1.52413	0.81599153	0.945184804	-0.129
cg09185998	II	37	1	2428936	PLCH2	0.5	0.00432	2.36443	0.63772388	0.508791543	0.129
cg03515844	II	37	1	16277545	ZBTB17	0.46	0.01197	1.92207	0.55469026	0.425268274	0.129
cg07965774	II	37	1	17746286	RCC2;RCC2	0.42	0.02991	1.52413	0.58763481	0.716891718	-0.129
cg17086398	II	37	1	31896392	SERINC2	0.5	0.00432	2.36443	0.22775017	0.098737054	0.129
cg12072789	II	37	1	110735112	SLC6A17	0.54	0.0014	2.85345	0.70919269	0.58001177	0.129
cg03514239	II	37	1	153329781	S100A9	0.5	0.00432	2.36443	0.27959307	0.408148643	-0.129
cg21196487	II	37	1	153538964	S100A2	0.54	0.0014	2.85345	0.22167678	0.3511326	-0.129
cg01352906	II	37	1	206679195		0.46	0.01197	1.92207	0.452373	0.323822434	0.129
cg04341343	II	37	2	8784813		0.54	0.0014	2.85345	0.25969867	0.388454795	-0.129
cg00055073	II	37	2	43398118		0.46	0.01197	1.92207	0.23971758	0.368824586	-0.129
cg16619576	II	37	2	45398036		0.58	0.00041	3.39184	0.57854662	0.707249499	-0.129
cg03549739	II	37	2	86163631		0.62	0.0001	3.98291	0.72079619	0.850025779	-0.129
cg13389146	II	37	2	96808802		0.58	0.00041	3.39184	0.75296575	0.624143074	0.129
cg00858840	II	37	2	171573839	SP5	0.58	0.00041	3.39184	0.11310501	0.242429488	-0.129
cg25124402	II	37	2	219125749	GPBAR1;GPBAR1;G	0.5	0.00432	2.36443	0.3240628	0.453539667	-0.129
cg02464551	II	37	2	232482571		0.75	7.61E-07	6.11857	0.44975189	0.578642944	-0.129
cg21672829	II	37	2	236619825	AGAP1;AGAP1	0.67	2.34E-05	4.63072	0.83145763	0.702480391	0.129
cg15058210	II	37	2	240196877	HDAC4	0.46	0.01197	1.92207	0.36619785	0.495168018	-0.129
cg26266876	II	37	3	23957486	RPL15;NKIRAS1	0.46	0.01197	1.92207	0.24054256	0.369471613	-0.129
cg26057969	II	37	3	127310291	TPRA1;TPRA1	0.46	0.01197	1.92207	0.14318886	0.271842098	-0.129

cg07356486	II	37	3	133646095		0.67	2.34E-05	4.63072	0.24616931	0.374824964	-0.129
cg20534585	I	37	3	196373598	LRRC33	0.46	0.01197	1.92207	0.61230867	0.741328713	-0.129
cg01132407	II	37	4	645781	PDE6B;PDE6B;PDE6	0.42	0.02991	1.52413	0.6655342	0.79476283	-0.129
cg16417416	II	37	4	6284031	WFS1;WFS1	0.5	0.00432	2.36443	0.64913397	0.777899324	-0.129
cg14342707	II	37	6	168053444		0.5	0.00432	2.36443	0.33901873	0.468142623	-0.129
cg07136111	II	37	7	30829005	FAM188B	0.42	0.02991	1.52413	0.63101307	0.760380291	-0.129
cg16459265	II	37	7	45025080	C7orf40;SNORA9	0.5	0.00432	2.36443	0.71567656	0.587147343	0.129
cg00122406	II	37	8	26431736		0.58	0.00041	3.39184	0.20122457	0.329833334	-0.129
cg25900943	II	37	8	103820614		0.5	0.00432	2.36443	0.6009829	0.730149109	-0.129
cg22459081	II	37	8	142095187		0.54	0.0014	2.85345	0.72978719	0.858743742	-0.129
cg01975786	II	37	8	142428968		0.42	0.02991	1.52413	0.62622888	0.496900603	0.129
cg27033805	I	37	8	145499436	BOP1	0.54	0.0014	2.85345	0.54361886	0.672788389	-0.129
cg14505439	II	37	9	134605210	RAPGEF1	0.58	0.00041	3.39184	0.63825949	0.509285303	0.129
cg22992966	II	37	10	43867024	FXYD4	0.62	0.0001	3.98291	0.63343537	0.76233898	-0.129
cg16352527	I	37	10	72476178	ADAMTS14;ADAMT	0.42	0.02991	1.52413	0.53134539	0.660792113	-0.129
cg14308082	II	37	10	131568021		0.62	0.0001	3.98291	0.6065771	0.735202309	-0.129
cg12822816	I	37	10	134361019	INPP5A	0.46	0.01197	1.92207	0.69769154	0.568713134	0.129
cg16802508	II	37	11	67070738	SSH3	0.5	0.00432	2.36443	0.33240658	0.460952798	-0.129
cg25256924	I	37	11	67205739	CORO1B;CORO1B;P	0.46	0.01197	1.92207	0.65186175	0.522970423	0.129
cg08037478	I	37	12	38545085		0.58	0.00041	3.39184	0.81791293	0.689200385	0.129
cg04472592	II	37	12	52585786	KRT80;KRT80	0.54	0.0014	2.85345	0.62418743	0.495499787	0.129
cg14507533	II	37	13	114066074		0.58	0.00041	3.39184	0.68137569	0.810855622	-0.129
cg07734106	II	37	14	96117415	TCL6	0.54	0.0014	2.85345	0.687585	0.558617476	0.129
cg01545493	II	37	14	103659071		0.54	0.0014	2.85345	0.64200187	0.77146654	-0.129
cg02072589	II	37	14	104917314		0.5	0.00432	2.36443	0.54887472	0.677870823	-0.129
cg04792777	II	37	14	106322429		0.54	0.0014	2.85345	0.5816924	0.452393449	0.129
cg27157619	II	37	15	74714463	SEMA7A;SEMA7A;S	0.46	0.01197	1.92207	0.82876697	0.699315232	0.129
cg17500686	II	37	15	79621783		0.54	0.0014	2.85345	0.74287597	0.871765923	-0.129
cg02498072	II	37	16	12088037	RUNDC2A	0.5	0.00432	2.36443	0.72839649	0.599775467	0.129
cg06352352	II	37	16	67181466	C16orf70	0.5	0.00432	2.36443	0.74471805	0.87339728	-0.129
cg07719604	II	37	16	67232460	ELMO3;E2F4	0.54	0.0014	2.85345	0.56357427	0.692133134	-0.129
cg06201861	II	37	16	84877964	CRISPLD2	0.54	0.0014	2.85345	0.67855482	0.807973659	-0.129
cg06763375	II	37	16	89025547	CBFA2T3	0.67	2.34E-05	4.63072	0.46302713	0.591789468	-0.129

cg06772580	II	37	17	27899576	TP53I13	0.54	0.0014	2.85345	0.47164103	0.600972461	-0.129
cg10035737	II	37	17	42840005	ADAM11	0.5	0.00432	2.36443	0.47339097	0.602535046	-0.129
cg16781907	II	37	17	48273001	COL1A1	0.42	0.02991	1.52413	0.70442885	0.575910954	0.129
cg23679492	II	37	17	55828084		0.62	0.0001	3.98291	0.58341015	0.712569056	-0.129
cg22091236	II	37	17	78853966	RPTOR;RPTOR	0.46	0.01197	1.92207	0.60672254	0.736199867	-0.129
cg26729913	II	37	17	79135071	AATK	0.67	2.34E-05	4.63072	0.36306164	0.491883161	-0.129
cg26551026	II	37	18	20717693	CABLES1;CABLES1	0.58	0.00041	3.39184	0.64332173	0.772445652	-0.129
cg10752406	I	37	19	827776	AZU1	0.42	0.02991	1.52413	0.40644744	0.534998872	-0.129
cg14779825	II	37	19	43099845	CEACAM8	0.46	0.01197	1.92207	0.40037305	0.529655357	-0.129
cg12986110	II	37	19	48551504	PLA2G4C;PLA2G4C;	0.54	0.0014	2.85345	0.78785469	0.658730019	0.129
cg01450566	II	37	20	9484340		0.67	2.34E-05	4.63072	0.46489294	0.593641214	-0.129
cg07727594	II	37	20	45187423	SLC13A3;SLC13A3	0.5	0.00432	2.36443	0.50454549	0.633617529	-0.129
cg16785077	II	37	21	42791867	MX1	0.54	0.0014	2.85345	0.67763068	0.806293366	-0.129
cg23512275	II	37	21	45232595	LOC284837	0.5	0.00432	2.36443	0.35833516	0.486926491	-0.129
cg06230805	I	37	21	45391366	AGPAT3;AGPAT3	0.58	0.00041	3.39184	0.77012574	0.899139361	-0.129
cg03296761	II	37	22	30822510	MTP18;MTP18	0.5	0.00432	2.36443	0.64451534	0.515775145	0.129
cg02079700	II	37	22	43166347		0.46	0.01197	1.92207	0.74840612	0.61957112	0.129
cg13863076	II	37	22	44392508		0.54	0.0014	2.85345	0.67799693	0.548708195	0.129
cg14942952	II	37	22	44576268	PARVG;PARVG;PAR	0.46	0.01197	1.92207	0.50092796	0.630242249	-0.129
cg09556700	I	37	1	2230668	SKI	0.42	0.02991	1.52413	0.71247194	0.840097812	-0.128
cg06099439	II	37	1	3146905	PRDM16;PRDM16	0.46	0.01197	1.92207	0.66269791	0.534956535	0.128
cg24371425	I	37	1	11561497	PTCHD2	0.42	0.02991	1.52413	0.46903826	0.596818908	-0.128
cg02086195	I	37	1	12656232	DHRS3	0.46	0.01197	1.92207	0.52672008	0.655205247	-0.128
cg11136886	II	37	1	33609509		0.5	0.00432	2.36443	0.74857166	0.876515906	-0.128
cg02289754	II	37	1	47068636	MKNK1;MKNK1;MK	0.5	0.00432	2.36443	0.74529943	0.873150494	-0.128
cg16527877	II	37	1	92036018		0.58	0.00041	3.39184	0.5637599	0.691473878	-0.128
cg19808620	I	37	1	153514442	S100A5	0.58	0.00041	3.39184	0.2159883	0.344033052	-0.128
cg22441770	II	37	1	153929592	CRTC2	0.46	0.01197	1.92207	0.60983519	0.482296778	0.128
cg09988805	II	37	2	43278552		0.46	0.01197	1.92207	0.69666077	0.568462229	0.128
cg18146074	II	37	2	232242955		0.46	0.01197	1.92207	0.34050733	0.468855848	-0.128
cg15868235	I	37	3	12486114		0.54	0.0014	2.85345	0.77235186	0.644668427	0.128
cg13296238	II	37	3	127323965	MCM2	0.42	0.02991	1.52413	0.56714619	0.69515552	-0.128
cg14637144	I	37	3	196750506	MFI2;MFI2	0.46	0.01197	1.92207	0.34236644	0.214774551	0.128

cg13941682	II	37	4	702545	PCGF3	0.54	0.0014	2.85345	0.73490413	0.8629894	-0.128
cg14724492	II	37	4	1015211	FGFRL1;FGFRL1;FGI	0.54	0.0014	2.85345	0.54180527	0.413623124	0.128
cg10742523	II	37	4	55650393		0.54	0.0014	2.85345	0.60999956	0.738495956	-0.128
cg02215171	II	37	4	89379156	HERC5	0.42	0.02991	1.52413	0.37013932	0.49773613	-0.128
cg09682727	II	37	5	139953966		0.42	0.02991	1.52413	0.54242854	0.670264751	-0.128
cg05016408	I	37	5	150326174	LOC134466	0.46	0.01197	1.92207	0.2474895	0.375219541	-0.128
cg07347294	II	37	5	176080248	TSPAN17;TSPAN17;	0.46	0.01197	1.92207	0.70872641	0.580535294	0.128
cg23672659	II	37	6	30648020	KIAA1949;KIAA194	0.54	0.0014	2.85345	0.59504009	0.466701265	0.128
cg06422467	II	37	6	30720484		0.46	0.01197	1.92207	0.22939969	0.357198225	-0.128
cg16537676	I	37	6	30851624	DDR1;DDR1	0.62	0.0001	3.98291	0.40092311	0.272611301	0.128
cg20132791	II	37	6	35992595	SLC26A8;SLC26A8	0.62	0.0001	3.98291	0.6223813	0.750603349	-0.128
cg04326499	II	37	6	45627080		0.54	0.0014	2.85345	0.79528563	0.666961559	0.128
cg06516476	II	37	6	86174584	NT5E	0.5	0.00432	2.36443	0.67015977	0.797826327	-0.128
cg08900363	II	37	7	637454	PRKAR1B;PRKAR1B	0.46	0.01197	1.92207	0.81450157	0.686839579	0.128
cg04095257	II	37	7	1062669	C7orf50;MIR339;C7	0.46	0.01197	1.92207	0.64064563	0.512754243	0.128
cg03590328	I	37	7	2684462	TTYH3	0.67	2.34E-05	4.63072	0.50295289	0.630523332	-0.128
cg14882265	II	37	7	27184375	HOXA5	0.5	0.00432	2.36443	0.59997971	0.727562003	-0.128
cg19706515	II	37	7	31376488		0.5	0.00432	2.36443	0.20280754	0.330462215	-0.128
cg13912964	II	37	7	100867281	ZNHIT1	0.58	0.00041	3.39184	0.77730355	0.905224574	-0.128
cg27269962	II	37	7	127540997	SND1	0.5	0.00432	2.36443	0.6389449	0.767389881	-0.128
cg27494647	I	37	7	150038898	RARRES2	0.5	0.00432	2.36443	0.33363508	0.461766177	-0.128
cg20902975	II	37	8	11138403		0.5	0.00432	2.36443	0.6791358	0.551228938	0.128
cg16103712	II	37	8	99023869	MATN2;MATN2	0.54	0.0014	2.85345	0.21894285	0.346968569	-0.128
cg10172675	II	37	8	104132683		0.54	0.0014	2.85345	0.33501452	0.463175638	-0.128
cg27270962	II	37	8	134498381	ST3GAL1;ST3GAL1	0.42	0.02991	1.52413	0.53511373	0.663574827	-0.128
cg03753385	I	37	8	145579317	FBXL6;C8ORFK29;FI	0.54	0.0014	2.85345	0.19163881	0.319513323	-0.128
cg26795312	II	37	8	146231199	C8orf77	0.54	0.0014	2.85345	0.76200211	0.633910328	0.128
cg16832407	II	37	9	73736539	TRPM3	0.62	0.0001	3.98291	0.24812531	0.375720501	-0.128
cg24044501	II	37	10	72056549		0.5	0.00432	2.36443	0.19721317	0.325219368	-0.128
cg02138953	II	37	10	75666279		0.62	0.0001	3.98291	0.73226337	0.86007014	-0.128
cg03339956	II	37	10	114818235	TCF7L2;TCF7L2;TCF	0.46	0.01197	1.92207	0.54603394	0.417639555	0.128
cg10902549	II	37	10	135202522	PAOX;PAOX;PAOX	0.42	0.02991	1.52413	0.27979331	0.152144487	0.128
cg18313182	II	37	11	818903	PNPLA2;PNPLA2	0.54	0.0014	2.85345	0.53580603	0.663964649	-0.128

cg09631059	II	37	11	856942	TSPAN4;TSPAN4;TS	0.54	0.0014	2.85345	0.80760966	0.679176396	0.128
cg11368628	II	37	11	1856183	SYT8	0.58	0.00041	3.39184	0.41025139	0.537953225	-0.128
cg11691844	II	37	11	85460604	SYTL2;SYTL2;SYTL2	0.5	0.00432	2.36443	0.62029675	0.748309115	-0.128
cg05824594	II	37	12	2734503	CACNA1C;CACNA1C	0.54	0.0014	2.85345	0.31463919	0.442455321	-0.128
cg18460107	II	37	12	7902153	CLEC4C;CLEC4C	0.46	0.01197	1.92207	0.37091837	0.242556736	0.128
cg19743522	I	37	12	113495566	DTX1	0.46	0.01197	1.92207	0.76212326	0.63434376	0.128
cg21726551	I	37	12	131590460	GPR133	0.46	0.01197	1.92207	0.63422346	0.506280747	0.128
cg09208010	II	37	14	23305780	MMP14	0.46	0.01197	1.92207	0.19032427	0.318047887	-0.128
cg14974772	II	37	14	92414425	FBLN5	0.42	0.02991	1.52413	0.36844466	0.496461429	-0.128
cg18416503	II	37	14	93475974	ITPK1;ITPK1;ITPK1	0.46	0.01197	1.92207	0.74205082	0.870156011	-0.128
cg11987068	II	37	15	77900788		0.46	0.01197	1.92207	0.78398642	0.656231393	0.128
cg08937107	II	37	16	3136857		0.5	0.00432	2.36443	0.34453138	0.472454852	-0.128
cg10359235	II	37	16	12135470	RUNDC2A	0.62	0.0001	3.98291	0.74711161	0.874931384	-0.128
cg07866632	II	37	16	28584802	CCDC101	0.46	0.01197	1.92207	0.27844472	0.406651744	-0.128
cg10341242	II	37	16	50347849	ADCY7	0.5	0.00432	2.36443	0.33580853	0.463576548	-0.128
cg09400037	I	37	16	84822801		0.62	0.0001	3.98291	0.7565665	0.884860224	-0.128
cg03040848	I	37	16	88993053	CBFA2T3;CBFA2T3	0.5	0.00432	2.36443	0.65996399	0.531685946	0.128
cg26756782	I	37	16	89922218	SPIRE2	0.42	0.02991	1.52413	0.61921208	0.747343391	-0.128
cg02656560	II	37	17	19967600		0.46	0.01197	1.92207	0.30198756	0.430090953	-0.128
cg23527902	II	37	17	61515708	CYB561;CYB561;CY	0.58	0.00041	3.39184	0.28876901	0.416855238	-0.128
cg07739927	II	37	17	62778162	LOC146880;LOC146	0.71	4.57E-06	5.34042	0.46654996	0.594272725	-0.128
cg20094462	I	37	19	848071	PRTN3	0.62	0.0001	3.98291	0.49216255	0.619902302	-0.128
cg20974196	II	37	19	859983	CFD	0.5	0.00432	2.36443	0.27781416	0.406188357	-0.128
cg08149682	II	37	19	1355918	MUM1;MUM1	0.62	0.0001	3.98291	0.20096667	0.328618364	-0.128
cg19577671	I	37	19	42782524		0.58	0.00041	3.39184	0.60378452	0.731527699	-0.128
cg11911305	II	37	19	45428924	APOC1P1;APOC1P1	0.58	0.00041	3.39184	0.33710346	0.464700178	-0.128
cg05492306	II	37	19	45927594	ERCC1;ERCC1;ERCC	0.46	0.01197	1.92207	0.19064268	0.318475595	-0.128
cg11847636	II	37	19	50015523	FCGRT;FCGRT	0.67	2.34E-05	4.63072	0.46116871	0.588999147	-0.128
cg11446693	II	37	19	59074994	MZF1;MZF1;LOC10	0.46	0.01197	1.92207	0.42052539	0.29278531	0.128
cg00170438	II	37	20	60792389	HRH3	0.46	0.01197	1.92207	0.38675956	0.258998583	0.128
cg22871485	I	37	22	22901830	PRAME;LOC648691	0.42	0.02991	1.52413	0.33147232	0.2031496	0.128
cg01843272	II	37	22	50911936	SBF1	0.67	2.34E-05	4.63072	0.56157074	0.433892702	0.128
cg07147033	II	37	1	1549615	MIB2;MIB2;MIB2;M	0.46	0.01197	1.92207	0.57700463	0.703929202	-0.127

cg16355231	I	37	1	2344979	PEX10;PEX10	0.5	0.00432	2.36443	0.37472841	0.247472969	0.127
cg03082523	II	37	1	3155965	PRDM16;PRDM16	0.54	0.0014	2.85345	0.6101447	0.483119213	0.127
cg12764201	II	37	1	10510123	CORT;APITD1;CORT	0.58	0.00041	3.39184	0.71295036	0.840142014	-0.127
cg01905489	II	37	1	26616534	UBXN11;UBXN11;U	0.58	0.00041	3.39184	0.32089626	0.194338544	0.127
cg15891991	II	37	1	117396695		0.58	0.00041	3.39184	0.18880769	0.315375	-0.127
cg12796135	II	37	1	153916764	DENND4B	0.46	0.01197	1.92207	0.57482926	0.702048294	-0.127
cg23166773	II	37	1	243659016	SDCCAG8;AKT3	0.46	0.01197	1.92207	0.47289265	0.600259942	-0.127
cg21806273	II	37	1	247578953	NLRP3;NLRP3	0.5	0.00432	2.36443	0.35930575	0.486457323	-0.127
cg22076676	II	37	2	43503935	THADA;THADA	0.54	0.0014	2.85345	0.5469924	0.673925601	-0.127
cg02444957	II	37	2	69440951	ANTXR1	0.42	0.02991	1.52413	0.70946528	0.582830025	0.127
cg20945085	I	37	2	74875227	C2orf65	0.58	0.00041	3.39184	0.33719936	0.463858995	-0.127
cg23051299	I	37	2	75938289	C2orf3	0.5	0.00432	2.36443	0.29877492	0.172059524	0.127
cg13149147	II	37	2	105757726		0.46	0.01197	1.92207	0.40937841	0.536349124	-0.127
cg26084700	I	37	2	231693479		0.67	2.34E-05	4.63072	0.56383098	0.437183506	0.127
cg04528931	II	37	2	240162187	HDAC4	0.42	0.02991	1.52413	0.1236549	0.25018618	-0.127
cg06578111	II	37	3	13546418	HDAC11;HDAC11	0.5	0.00432	2.36443	0.71085164	0.58416153	0.127
cg04363536	I	37	3	49466872	NICN1	0.62	0.0001	3.98291	0.600771	0.474226102	0.127
cg18521796	II	37	3	128713631	KIAA1257	0.58	0.00041	3.39184	0.58069493	0.707584803	-0.127
cg16824282	II	37	3	128779590	GP9	0.5	0.00432	2.36443	0.18114623	0.308210796	-0.127
cg07578772	II	37	3	150420821	FAM194A	0.54	0.0014	2.85345	0.59473976	0.467551571	0.127
cg03805566	II	37	3	183245952	KLHL6	0.58	0.00041	3.39184	0.80303823	0.675660514	0.127
cg24394172	II	37	4	46995203	GABRA4	0.5	0.00432	2.36443	0.36311819	0.489999096	-0.127
cg11677852	II	37	5	1108876	SLC12A7	0.54	0.0014	2.85345	0.57207836	0.699080531	-0.127
cg26876242	II	37	5	149115085	PPARGC1B	0.46	0.01197	1.92207	0.73340505	0.860809946	-0.127
cg04699519	II	37	6	16306096	ATXN1;ATXN1	0.42	0.02991	1.52413	0.6696425	0.542176438	0.127
cg06108383	II	37	6	32120899	PPT2;PRRT1;PPT2	0.42	0.02991	1.52413	0.79981577	0.672721762	0.127
cg11453837	II	37	6	32810551	PSMB8;PSMB8	0.5	0.00432	2.36443	0.72292222	0.595857437	0.127
cg05454562	II	37	6	33254447	WDR46;WDR46	0.5	0.00432	2.36443	0.77616659	0.903044755	-0.127
cg06639585	II	37	6	36236936	PNPLA1;PNPLA1;PN	0.5	0.00432	2.36443	0.57933963	0.706130956	-0.127
cg07017242	II	37	7	128047441	IMPDH1;IMPDH1;IN	0.54	0.0014	2.85345	0.57568588	0.448381542	0.127
cg21579209	II	37	7	138620362	KIAA1549;KIAA154	0.5	0.00432	2.36443	0.25559061	0.382127663	-0.127
cg03489712	II	37	7	143076881	ZYX;ZYX	0.46	0.01197	1.92207	0.53027078	0.657106776	-0.127
cg15316716	I	37	8	21769797	DOK2	0.5	0.00432	2.36443	0.69940408	0.572121791	0.127

cg22586603	II	37	8	129985596		0.5	0.00432	2.36443	0.56729746	0.694031771	-0.127
cg16305292	II	37	8	142219965		0.46	0.01197	1.92207	0.68132031	0.808451198	-0.127
cg09911401	II	37	10	29827566	SVIL;SVIL	0.46	0.01197	1.92207	0.88079342	0.753367809	0.127
cg22715761	II	37	10	88428147	LDB3;LDB3;LDB3;LD	0.46	0.01197	1.92207	0.68807472	0.561545064	0.127
cg01418188	II	37	11	3145609	OSBPL5;OSBPL5;OS	0.46	0.01197	1.92207	0.31636869	0.443429094	-0.127
cg16801797	II	37	11	20043509	NAV2;NAV2;NAV2;I	0.46	0.01197	1.92207	0.38148363	0.508844327	-0.127
cg19349861	I	37	11	133402246	OPCML;OPCML	0.67	2.34E-05	4.63072	0.41408755	0.287117665	0.127
cg03224812	II	37	12	3807404	EFCAB4B;EFCAB4B;	0.5	0.00432	2.36443	0.73434467	0.861116287	-0.127
cg02082571	II	37	12	8276718	CLEC4A;CLEC4A;CLE	0.5	0.00432	2.36443	0.40898472	0.53580829	-0.127
cg22958090	I	37	12	52627438	KRT7	0.46	0.01197	1.92207	0.25570197	0.382829109	-0.127
cg26477856	II	37	12	56324915	DGKA;DGKA;DGKA;	0.62	0.0001	3.98291	0.53681457	0.410219263	0.127
cg17651972	II	37	12	57620054	NXPH4	0.54	0.0014	2.85345	0.2757146	0.403155687	-0.127
cg16134678	I	37	13	25670187	PABPC3	0.42	0.02991	1.52413	0.32121522	0.194230083	0.127
cg03185794	II	37	13	101237439		0.5	0.00432	2.36443	0.49936329	0.372464188	0.127
cg10807643	I	37	14	55907417	TBPL2	0.42	0.02991	1.52413	0.63396883	0.760501672	-0.127
cg21235823	II	37	14	93260203	GOLGA5	0.71	4.57E-06	5.34042	0.19726786	0.324383606	-0.127
cg03462322	II	37	16	126397	MPG	0.5	0.00432	2.36443	0.19874882	0.325705671	-0.127
cg01573635	II	37	16	10788751	TEKT5;TEKT5	0.54	0.0014	2.85345	0.72539235	0.597897623	0.127
cg27097542	II	37	16	11706435		0.46	0.01197	1.92207	0.20310069	0.329827175	-0.127
cg27565277	II	37	16	17228474	XYLT1	0.42	0.02991	1.52413	0.78369429	0.65658668	0.127
cg07531182	II	37	16	17299114	XYLT1	0.62	0.0001	3.98291	0.56685549	0.693670983	-0.127
cg00888605	II	37	16	71523475	ZNF19	0.58	0.00041	3.39184	0.66274212	0.789573562	-0.127
cg06190046	II	37	16	83986382	OSGIN1;OSGIN1;OS	0.42	0.02991	1.52413	0.28710108	0.413648993	-0.127
cg06948222	II	37	16	87958493	CA5A	0.5	0.00432	2.36443	0.76173344	0.635126582	0.127
cg22664962	II	37	17	22191979		0.46	0.01197	1.92207	0.56143414	0.688908655	-0.127
cg14173815	II	37	17	27049557	RPL23A;SNORD42A	0.42	0.02991	1.52413	0.65505289	0.528099369	0.127
cg15295469	II	37	17	37080300		0.54	0.0014	2.85345	0.38215565	0.509490368	-0.127
cg12610859	II	37	17	37822780	TCAP	0.5	0.00432	2.36443	0.68888557	0.815431276	-0.127
cg25649895	II	37	17	48356195	TMEM92;TMEM92	0.54	0.0014	2.85345	0.61477151	0.741888915	-0.127
cg24809269	II	37	17	74260692	FAM100B	0.46	0.01197	1.92207	0.63252408	0.50543005	0.127
cg15796340	II	37	17	74527554	CYGB	0.42	0.02991	1.52413	0.57572217	0.449120361	0.127
cg03221619	II	37	19	7767348	FCER2	0.46	0.01197	1.92207	0.58524645	0.457806538	0.127
cg17114866	II	37	19	35222640		0.79	1.06E-07	6.97389	0.58733312	0.714026974	-0.127

cg23942508	II	37	19	45201924	CEACAM16	0.5	0.00432	2.36443	0.5503541	0.422985404	0.127
cg17507897	II	37	20	17943694	SNX5;SNORD17;SN	0.46	0.01197	1.92207	0.76822141	0.641001429	0.127
cg10167508	II	37	20	46988400	LOC284749	0.54	0.0014	2.85345	0.69399759	0.567221941	0.127
cg11632679	II	37	20	56759082		0.46	0.01197	1.92207	0.71072324	0.58406279	0.127
cg09468832	II	37	21	45199094		0.54	0.0014	2.85345	0.20461592	0.331276529	-0.127
cg02687883	II	37	1	2266431	MORN1	0.58	0.00041	3.39184	0.69326882	0.56679784	0.126
cg15131146	II	37	1	26101755	MAN1C1	0.5	0.00432	2.36443	0.3078643	0.181827252	0.126
cg22228439	II	37	1	46766431	LRRC41	0.5	0.00432	2.36443	0.53879191	0.664425047	-0.126
cg16416987	II	37	1	155177561	THBS3;MTX1;MTX1	0.54	0.0014	2.85345	0.27215032	0.398571898	-0.126
cg20741040	II	37	1	230566509		0.42	0.02991	1.52413	0.24666043	0.372987827	-0.126
cg01569295	II	37	1	231155862	MIR1182;FAM89A	0.67	2.34E-05	4.63072	0.72047904	0.846541601	-0.126
cg05962382	II	37	2	130345044		0.5	0.00432	2.36443	0.61562172	0.741319313	-0.126
cg12004641	II	37	2	218750749	TNS1	0.54	0.0014	2.85345	0.80550627	0.679927071	0.126
cg13916298	II	37	3	108896099	C3orf66	0.58	0.00041	3.39184	0.31312804	0.439035068	-0.126
cg04224041	II	37	4	1220556	CTBP1;CTBP1	0.54	0.0014	2.85345	0.65439579	0.780039877	-0.126
cg14967987	II	37	4	3204843	HTT	0.46	0.01197	1.92207	0.5654138	0.439759609	0.126
cg11080651	II	37	5	10445523	ROPN1L	0.5	0.00432	2.36443	0.25307267	0.379206389	-0.126
cg27223047	II	37	5	127874825	FBN2	0.46	0.01197	1.92207	0.17786206	0.303863705	-0.126
cg02125316	I	37	5	170878209	FGF18	0.42	0.02991	1.52413	0.0384002	0.164040177	-0.126
cg12247101	I	37	6	30653549	KIAA1949;KIAA194	0.54	0.0014	2.85345	0.47854126	0.352420609	0.126
cg02673107	II	37	6	33138131	COL11A2;COL11A2;	0.5	0.00432	2.36443	0.3035472	0.177495663	0.126
cg04813787	II	37	6	167814397		0.46	0.01197	1.92207	0.67350649	0.547706934	0.126
cg05006142	II	37	6	170536124		0.62	0.0001	3.98291	0.71215413	0.838538411	-0.126
cg05107246	II	37	7	31441		0.46	0.01197	1.92207	0.49528168	0.621533686	-0.126
cg16784234	II	37	7	530475		0.54	0.0014	2.85345	0.65201741	0.526151385	0.126
cg25315312	I	37	7	2020528	MAD1L1;MAD1L1;M	0.5	0.00432	2.36443	0.60362114	0.478024694	0.126
cg08933276	II	37	7	2770410	GNA12	0.46	0.01197	1.92207	0.66986234	0.796044591	-0.126
cg04682905	II	37	7	5523636	FBXL18	0.54	0.0014	2.85345	0.58905443	0.715429904	-0.126
cg07621224	II	37	7	50355506	IKZF1	0.46	0.01197	1.92207	0.57502913	0.449408476	0.126
cg23129573	I	37	7	73442487	ELN;ELN;ELN;ELN;E	0.62	0.0001	3.98291	0.14175509	0.268203912	-0.126
cg03461678	II	37	7	100859600	ZNHIT1;PLOD3	0.67	2.34E-05	4.63072	0.65056571	0.776752284	-0.126
cg22619824	II	37	7	116786606	ST7;ST7;ST7OT2	0.46	0.01197	1.92207	0.61889805	0.745106188	-0.126
cg00158770	II	37	8	19271585	CSGALNACT1;CSGA	0.46	0.01197	1.92207	0.7511398	0.877505588	-0.126

cg23494863	I	37	8	38009476	STAR;STAR	0.5	0.00432	2.36443	0.59379306	0.719542893	-0.126
cg01976921	II	37	8	38342087		0.54	0.0014	2.85345	0.54231697	0.667908989	-0.126
cg19352832	II	37	8	42608976	CHRNA6	0.5	0.00432	2.36443	0.66274796	0.537139885	0.126
cg19611886	II	37	8	103821171		0.5	0.00432	2.36443	0.27984811	0.405565938	-0.126
cg11006453	I	37	8	141599185	EIF2C2;EIF2C2	0.42	0.02991	1.52413	0.76400102	0.638454261	0.126
cg16899498	I	37	8	144513907	MAFA	0.62	0.0001	3.98291	0.26557996	0.1393692	0.126
cg08006727	II	37	9	116061614	RNF183	0.42	0.02991	1.52413	0.73701585	0.611208141	0.126
cg14443472	II	37	9	129266352	FAM125B	0.5	0.00432	2.36443	0.70261666	0.577067321	0.126
cg14611112	II	37	9	139643351	LCN6	0.5	0.00432	2.36443	0.30640701	0.432345884	-0.126
cg07191657	II	37	10	14478541	MIR1265	0.5	0.00432	2.36443	0.45686512	0.582837112	-0.126
cg11889730	II	37	10	37967356		0.5	0.00432	2.36443	0.53450594	0.408651415	0.126
cg06259934	I	37	10	81034374	ZMIZ1	0.5	0.00432	2.36443	0.75836526	0.884745265	-0.126
cg02795981	II	37	10	81045119	ZMIZ1	0.5	0.00432	2.36443	0.65328596	0.527469764	0.126
cg22593342	II	37	10	82219410	TSPAN14;TSPAN14	0.46	0.01197	1.92207	0.67454975	0.548344607	0.126
cg09614415	I	37	10	129535509	FOXI2	0.58	0.00041	3.39184	0.11720014	0.243379365	-0.126
cg02323003	II	37	10	130525659		0.46	0.01197	1.92207	0.49629668	0.370534749	0.126
cg17416793	II	37	11	2846932	KCNQ1;KCNQ1	0.54	0.0014	2.85345	0.45336215	0.579103916	-0.126
cg15172739	II	37	11	62621258	SNORD30;SNORD2[illegible]	0.5	0.00432	2.36443	0.76974112	0.643619591	0.126
cg08672956	II	37	11	116640933	BUD13;BUD13	0.62	0.0001	3.98291	0.7632198	0.889084093	-0.126
cg17318719	II	37	11	117959349	TMPRSS4;TMPRSS4	0.62	0.0001	3.98291	0.24134116	0.367108325	-0.126
cg15712304	I	37	11	125227489	PKNOX2	0.5	0.00432	2.36443	0.76111961	0.63485756	0.126
cg21793437	II	37	12	2734591	CACNA1C;CACNA1[illegible]	0.58	0.00041	3.39184	0.29279995	0.419142168	-0.126
cg03539765	I	37	12	9217390	LOC144571	0.46	0.01197	1.92207	0.09650354	0.222462187	-0.126
cg24279017	II	37	12	11877740	ETV6	0.54	0.0014	2.85345	0.58148789	0.707306301	-0.126
cg10205310	II	37	12	117131820		0.5	0.00432	2.36443	0.71355168	0.587525759	0.126
cg01816936	II	37	12	123518888	PITPNM2	0.58	0.00041	3.39184	0.21095521	0.33654005	-0.126
cg17187521	II	37	12	125003379	NCOR2;NCOR2	0.42	0.02991	1.52413	0.25431933	0.380188651	-0.126
cg11334728	II	37	12	132697723	GALNT9	0.58	0.00041	3.39184	0.74909686	0.623007514	0.126
cg08357012	I	37	13	99090919	FARP1	0.42	0.02991	1.52413	0.88445899	0.758539913	0.126
cg00320765	II	37	14	69264333	C14orf181	0.46	0.01197	1.92207	0.7500554	0.623755839	0.126
cg25561904	II	37	14	104165401	XRCC3;XRCC3;XRC[illegible]	0.42	0.02991	1.52413	0.69322145	0.567444386	0.126
cg01767544	I	37	14	106321115		0.46	0.01197	1.92207	0.87466683	0.748845141	0.126
cg02574101	II	37	15	78286737	LOC91450	0.42	0.02991	1.52413	0.69464631	0.568976907	0.126

cg11407210	II	37	15	79199774		0.54	0.0014	2.85345	0.54106875	0.667134286	-0.126
cg04188862	I	37	15	88801474		0.5	0.00432	2.36443	0.34019688	0.46612053	-0.126
cg10160612	II	37	16	30751899		0.5	0.00432	2.36443	0.32532108	0.451161324	-0.126
cg10482356	II	37	16	56328421	GNAO1;GNAO1	0.54	0.0014	2.85345	0.6937152	0.820123815	-0.126
cg04187039	II	37	16	89048704		0.54	0.0014	2.85345	0.55434939	0.428110646	0.126
cg07474797	II	37	16	89185916	ACSF3;ACSF3;ACSF3	0.58	0.00041	3.39184	0.31042895	0.436102797	-0.126
cg21333338	II	37	17	2808456	RAP1GAP2;RAP1GA	0.42	0.02991	1.52413	0.61372948	0.740095555	-0.126
cg21327235	II	37	17	7643798	DNAH2	0.42	0.02991	1.52413	0.58717454	0.713503054	-0.126
cg22787468	II	37	17	27309113	SEZ6;SEZ6	0.54	0.0014	2.85345	0.72437032	0.850193137	-0.126
cg00950381	I	37	17	74272364	QRICH2	0.5	0.00432	2.36443	0.6152814	0.488937164	0.126
cg19536407	I	37	17	79431331	BAHCC1	0.54	0.0014	2.85345	0.80126463	0.675104521	0.126
cg02862897	II	37	17	79793088	DYSFIP1	0.54	0.0014	2.85345	0.66331365	0.788945342	-0.126
cg10168457	II	37	17	79882876	MAFG;MAFG	0.5	0.00432	2.36443	0.37987366	0.505539764	-0.126
cg15407058	II	37	17	80175989		0.54	0.0014	2.85345	0.53639565	0.662397833	-0.126
cg27658601	I	37	17	80794081	TBCD;ZNF750	0.54	0.0014	2.85345	0.799021	0.925281196	-0.126
cg14679780	I	37	19	4059525	ZBTB7A	0.5	0.00432	2.36443	0.70438506	0.57825836	0.126
cg16526705	II	37	19	17284481	MYO9B;MYO9B	0.46	0.01197	1.92207	0.64772956	0.773521343	-0.126
cg02189888	II	37	19	46582065		0.54	0.0014	2.85345	0.29637966	0.422378414	-0.126
cg26415633	II	37	19	51327356	KLK1	0.54	0.0014	2.85345	0.6363301	0.510429614	0.126
cg02928664	I	37	19	54976506	CDC42EP5	0.46	0.01197	1.92207	0.08890387	0.215336795	-0.126
cg09971811	II	37	20	24930099	CST7;CST7	0.71	4.57E-06	5.34042	0.4485445	0.574159864	-0.126
cg24956391	I	37	20	36011999	SRC;SRC	0.5	0.00432	2.36443	0.76807582	0.641699488	0.126
cg06287548	II	37	21	34773372		0.42	0.02991	1.52413	0.4447007	0.570306977	-0.126
cg04335714	II	37	22	25799070		0.62	0.0001	3.98291	0.24185044	0.36808265	-0.126
cg24579970	II	37	1	7123315	CAMTA1	0.42	0.02991	1.52413	0.81637317	0.690903132	0.125
cg25369262	II	37	1	46649132	TSPAN1	0.46	0.01197	1.92207	0.50143879	0.626350172	-0.125
cg19587237	II	37	1	54797076	SSBP3;SSBP3;SSBP3	0.54	0.0014	2.85345	0.21049016	0.335716394	-0.125
cg23633856	II	37	1	170636363	PRRX1;PRRX1	0.58	0.00041	3.39184	0.31548008	0.440099883	-0.125
cg05783585	II	37	1	201118532	TMEM9	0.5	0.00432	2.36443	0.85758529	0.733010866	0.125
cg19081101	I	37	1	203156625	CHI3L1	0.5	0.00432	2.36443	0.73713209	0.861712468	-0.125
cg16278077	I	37	1	205635579	SLC45A3	0.5	0.00432	2.36443	0.85674	0.731784798	0.125
cg15561613	I	37	1	245851610	KIF26B	0.46	0.01197	1.92207	0.37040434	0.495492504	-0.125
cg23926598	II	37	2	47406858		0.5	0.00432	2.36443	0.65059384	0.775574312	-0.125

cg21512179	II	37	2	85971548		0.54	0.0014	2.85345	0.79919213	0.674412074	0.125
cg00533827	I	37	2	86163827		0.54	0.0014	2.85345	0.82093553	0.945691747	-0.125
cg10236239	II	37	2	108994514	SULT1C4;SULT1C4	0.58	0.00041	3.39184	0.2425498	0.367915804	-0.125
cg20563269	I	37	2	129104576		0.5	0.00432	2.36443	0.7835559	0.908541184	-0.125
cg15175143	II	37	2	139538356	NXPH2	0.42	0.02991	1.52413	0.21774345	0.342678101	-0.125
cg02639108	I	37	2	242711009		0.42	0.02991	1.52413	0.78193752	0.906577628	-0.125
cg05020203	II	37	2	242968829		0.5	0.00432	2.36443	0.58850697	0.463840072	0.125
cg15044270	II	37	3	196065485	TM4SF19	0.5	0.00432	2.36443	0.32499861	0.449626531	-0.125
cg26131315	II	37	4	140787316	MAML3	0.58	0.00041	3.39184	0.89801646	0.773456515	0.125
cg14817490	II	37	5	392920	AHRR	0.42	0.02991	1.52413	0.42674205	0.551575571	-0.125
cg00911351	II	37	5	140767255	PCDHGA4;PCDHGA	0.5	0.00432	2.36443	0.16939173	0.294353331	-0.125
cg08491188	II	37	5	140777503	PCDHGA4;PCDHGA	0.62	0.0001	3.98291	0.19273247	0.31777927	-0.125
cg11671363	I	37	5	148810177	MIR145;LOC72826	0.5	0.00432	2.36443	0.77217757	0.897302013	-0.125
cg11327408	II	37	6	30297329	TRIM39;TRIM39	0.42	0.02991	1.52413	0.75087145	0.876122147	-0.125
cg25351606	I	37	6	100917427		0.46	0.01197	1.92207	0.19245467	0.317065303	-0.125
cg08250108	II	37	6	170425832		0.67	2.34E-05	4.63072	0.80073089	0.675533912	0.125
cg13151425	I	37	7	2561116	LFNG;LFNG;LFNG;LI	0.79	1.06E-07	6.97389	0.43795202	0.313329565	0.125
cg12738008	I	37	8	2037954	MYOM2	0.54	0.0014	2.85345	0.77812908	0.652976368	0.125
cg12054981	I	37	8	42037387	PLAT;PLAT	0.46	0.01197	1.92207	0.27117781	0.396464765	-0.125
cg02471183	II	37	8	67456683		0.42	0.02991	1.52413	0.58970907	0.464875145	0.125
cg21157725	II	37	9	136809652	VAV2;VAV2	0.42	0.02991	1.52413	0.54798536	0.422645375	0.125
cg13941235	II	37	9	137270186	RXRA	0.46	0.01197	1.92207	0.44817654	0.573522209	-0.125
cg09838169	I	37	10	134377481	INPP5A	0.5	0.00432	2.36443	0.86238847	0.737790809	0.125
cg06080948	I	37	10	135202877	PAOX;PAOX;PAOX	0.42	0.02991	1.52413	0.24711119	0.122084607	0.125
cg26236329	II	37	11	57200325		0.58	0.00041	3.39184	0.27440013	0.399348214	-0.125
cg11775521	I	37	11	74178795	KCNE3	0.42	0.02991	1.52413	0.23010517	0.355172174	-0.125
cg19907915	I	37	11	133821252	IGSF9B	0.5	0.00432	2.36443	0.79638117	0.671859253	0.125
cg00522935	II	37	12	1964603	CACNA2D4	0.54	0.0014	2.85345	0.74446459	0.619394661	0.125
cg01833890	II	37	12	2564063	CACNA1C;CACNA1C	0.5	0.00432	2.36443	0.40872135	0.533479463	-0.125
cg26538140	II	37	12	6996791		0.42	0.02991	1.52413	0.39857705	0.523495425	-0.125
cg02883147	II	37	12	9217769	LOC144571	0.46	0.01197	1.92207	0.44469856	0.569424548	-0.125
cg04020713	II	37	12	53457968	TENC1;TENC1;TENC	0.46	0.01197	1.92207	0.54691944	0.422331547	0.125
cg25684349	II	37	12	107725084	BTBD11	0.54	0.0014	2.85345	0.36501635	0.240248893	0.125

cg26262840	II	37	14	90767686	C14orf102;C14orf1(	0.5	0.00432	2.36443	0.77381023	0.899221758	-0.125
cg10761141	II	37	14	94844776	SERPINA1;SERPINA	0.54	0.0014	2.85345	0.75666618	0.631695019	0.125
cg24761597	I	37	14	103984978		0.62	0.0001	3.98291	0.61678933	0.491737611	0.125
cg14905514	II	37	14	105998745		0.46	0.01197	1.92207	0.59150459	0.466246555	0.125
cg19938199	II	37	15	63233164		0.5	0.00432	2.36443	0.46313904	0.588579264	-0.125
cg21275690	II	37	16	3072970	HCFC1R1;HCFC1R1;	0.67	2.34E-05	4.63072	0.32214736	0.44730037	-0.125
cg08367804	II	37	16	3639160	BTBD12	0.54	0.0014	2.85345	0.40762631	0.532295015	-0.125
cg06627532	II	37	16	81491312	CMIP	0.67	2.34E-05	4.63072	0.34378867	0.469025433	-0.125
cg16374343	II	37	17	1014352	ABR;ABR	0.58	0.00041	3.39184	0.30093253	0.425467142	-0.125
cg06191091	I	37	17	30583855		0.42	0.02991	1.52413	0.82392222	0.699137098	0.125
cg23901967	II	37	17	36890321	CISD3;PCGF2	0.54	0.0014	2.85345	0.70412764	0.578934753	0.125
cg05117208	II	37	17	40438312	STAT5A	0.46	0.01197	1.92207	0.44932636	0.574304375	-0.125
cg20359349	II	37	17	41994531	C17orf88	0.5	0.00432	2.36443	0.5711891	0.445948668	0.125
cg24434387	II	37	17	45051692		0.54	0.0014	2.85345	0.59499238	0.719731007	-0.125
cg06942183	II	37	17	46622607	HOXB2	0.54	0.0014	2.85345	0.26519784	0.389894676	-0.125
cg16766914	I	37	17	55962703	CUEDC1	0.46	0.01197	1.92207	0.65828553	0.782912468	-0.125
cg10023862	I	37	17	55962841	CUEDC1	0.5	0.00432	2.36443	0.75097727	0.875499023	-0.125
cg23641597	II	37	17	70162036		0.42	0.02991	1.52413	0.65020893	0.775023474	-0.125
cg16776035	II	37	17	74542680		0.42	0.02991	1.52413	0.46297284	0.587993277	-0.125
cg27551910	II	37	17	79083905	BAIAP2;BAIAP2;BAI	0.42	0.02991	1.52413	0.44531515	0.31984982	0.125
cg06511678	II	37	18	7038746	LAMA1	0.5	0.00432	2.36443	0.30779743	0.433151085	-0.125
cg23693749	II	37	18	76399434		0.46	0.01197	1.92207	0.50071938	0.625530098	-0.125
cg21394039	II	37	18	77116310	ATP9B	0.46	0.01197	1.92207	0.72067703	0.595908555	0.125
cg15664905	II	37	19	44009429	PHLDB3	0.67	2.34E-05	4.63072	0.68948526	0.814011834	-0.125
cg10129493	II	37	19	51728586	CD33;CD33	0.58	0.00041	3.39184	0.14977312	0.274824249	-0.125
cg24713204	II	37	19	57019373	ZNF471	0.54	0.0014	2.85345	0.24275061	0.36793198	-0.125
cg26955850	II	37	20	3052345	OXT	0.58	0.00041	3.39184	0.32412066	0.449120317	-0.125
cg12485727	II	37	20	39762753		0.58	0.00041	3.39184	0.7880343	0.663086995	0.125
cg01373166	II	37	22	24823389	ADORA2A	0.62	0.0001	3.98291	0.52763964	0.402945975	0.125
cg05718255	II	37	22	35790141	HMOX1	0.5	0.00432	2.36443	0.59182709	0.467120848	0.125
cg17806661	II	37	22	37096133	CACNG2	0.5	0.00432	2.36443	0.37565874	0.500384387	-0.125
cg02539402	II	37	22	43061182		0.5	0.00432	2.36443	0.52575885	0.401166301	0.125
cg02393107	II	37	1	2515692		0.54	0.0014	2.85345	0.6929417	0.569109433	0.124

cg08371190	II	37	1	87598514	LOC339524;LOC339	0.58	0.00041	3.39184	0.56650509	0.690659379	-0.124
cg07213548	II	37	1	104616267		0.5	0.00432	2.36443	0.60241399	0.726484303	-0.124
cg17496887	II	37	1	153387707	S100A7A	0.46	0.01197	1.92207	0.74717934	0.871478684	-0.124
cg26400546	II	37	1	205499355	CDK18;CDK18;CDK1	0.42	0.02991	1.52413	0.72291837	0.598660149	0.124
cg22719308	I	37	1	214725918	PTPN14	0.5	0.00432	2.36443	0.19468827	0.318698983	-0.124
cg01498249	II	37	2	312940		0.5	0.00432	2.36443	0.60920953	0.485465316	0.124
cg21486510	I	37	2	7171998	RNF144A	0.54	0.0014	2.85345	0.37800098	0.502227587	-0.124
cg12018969	II	37	2	43350413		0.46	0.01197	1.92207	0.68860841	0.812914507	-0.124
cg15742777	II	37	2	55339218		0.46	0.01197	1.92207	0.13362449	0.257631154	-0.124
cg12910810	II	37	2	96662438		0.46	0.01197	1.92207	0.45899156	0.582699346	-0.124
cg00423969	II	37	2	97359879	FER1L5;FER1L5	0.54	0.0014	2.85345	0.25804857	0.382040609	-0.124
cg05886087	II	37	2	102758186		0.54	0.0014	2.85345	0.46085408	0.584530665	-0.124
cg04361749	II	37	2	240980455		0.46	0.01197	1.92207	0.56186617	0.437872505	0.124
cg01637169	II	37	3	13459281	NUP210	0.58	0.00041	3.39184	0.49309144	0.368840363	0.124
cg01965673	II	37	3	126700750		0.42	0.02991	1.52413	0.5661251	0.442093498	0.124
cg08140055	II	37	3	134369339	KY	0.62	0.0001	3.98291	0.56084775	0.68472288	-0.124
cg14666404	II	37	3	183016948	MCF2L2	0.62	0.0001	3.98291	0.47502266	0.599302409	-0.124
cg10134910	II	37	4	1076041	RNF212;RNF212	0.54	0.0014	2.85345	0.37532512	0.251415654	0.124
cg10869531	II	37	4	2808489	SH3BP2	0.46	0.01197	1.92207	0.61111799	0.487475287	0.124
cg19241973	I	37	4	8262688		0.42	0.02991	1.52413	0.48079063	0.604881212	-0.124
cg09988671	II	37	4	8351738		0.46	0.01197	1.92207	0.70179315	0.578020864	0.124
cg17854066	II	37	4	55575932	KIT;KIT	0.42	0.02991	1.52413	0.19787798	0.321448035	-0.124
cg26703534	II	37	5	377358	AHRR	0.58	0.00041	3.39184	0.63374178	0.757709628	-0.124
cg22649349	II	37	6	3848898	FAM50B	0.58	0.00041	3.39184	0.6779693	0.801881817	-0.124
cg08696107	II	37	6	29759949	HCG4	0.46	0.01197	1.92207	0.16931115	0.293743437	-0.124
cg13401893	I	37	6	30039432	RNF39;RNF39	0.5	0.00432	2.36443	0.16078025	0.28506336	-0.124
cg00174718	II	37	6	47136110		0.46	0.01197	1.92207	0.44819294	0.571796007	-0.124
cg07561610	I	37	6	149771807	ZC3H12D	0.58	0.00041	3.39184	0.66715435	0.791469609	-0.124
cg07143083	I	37	7	70597921	WBSCR17	0.46	0.01197	1.92207	0.16074141	0.284286583	-0.124
cg03431524	II	37	7	100142441	AGFG2	0.46	0.01197	1.92207	0.70933424	0.585748744	0.124
cg11747183	I	37	7	149918226		0.46	0.01197	1.92207	0.15751542	0.281844897	-0.124
cg07811198	II	37	7	153585368	DPP6	0.67	2.34E-05	4.63072	0.25564745	0.379466514	-0.124
cg06152586	I	37	7	157890659	PTPRN2;PTPRN2;PT	0.46	0.01197	1.92207	0.77494829	0.898675652	-0.124

cg22307444	I	37	8	672057	ERICH1	0.46	0.01197	1.92207	0.37980069	0.503443649	-0.124
cg15518883	II	37	9	35650561	SIT1	0.67	2.34E-05	4.63072	0.59056229	0.466990244	0.124
cg13447566	I	37	9	133801544	FIBCD1;FIBCD1	0.42	0.02991	1.52413	0.6987505	0.574818878	0.124
cg00056066	I	37	10	25010738	ARHGAP21	0.42	0.02991	1.52413	0.53514823	0.659582886	-0.124
cg25427524	I	37	10	38739819	LOC399744	0.46	0.01197	1.92207	0.53715008	0.661361804	-0.124
cg19499884	II	37	10	102760724	LZTS2	0.46	0.01197	1.92207	0.34144645	0.46563206	-0.124
cg03022891	I	37	11	1947791	TNNT3;TNNT3;TNN	0.42	0.02991	1.52413	0.55644289	0.680227636	-0.124
cg00491064	II	37	11	19681786	NAV2	0.42	0.02991	1.52413	0.79043782	0.666262701	0.124
cg07092212	II	37	11	46382544	DGKZ;DGKZ;DGKZ;D	0.42	0.02991	1.52413	0.26996061	0.394228047	-0.124
cg03778108	II	37	11	47360129	MYBPC3	0.46	0.01197	1.92207	0.75185494	0.628186004	0.124
cg19240569	II	37	11	63331821	HRASLS2	0.54	0.0014	2.85345	0.68822378	0.564689099	0.124
cg00157359	II	37	11	66233183	PELI3;PELI3	0.42	0.02991	1.52413	0.3335591	0.209245601	0.124
cg20705781	II	37	11	67070238	SSH3	0.58	0.00041	3.39184	0.35448684	0.478382605	-0.124
cg24101009	II	37	11	93863858	PANX1	0.62	0.0001	3.98291	0.64672584	0.770457739	-0.124
cg17983957	II	37	11	118083938	AMICA1;AMICA1;AI	0.54	0.0014	2.85345	0.76659006	0.890719626	-0.124
cg02150262	II	37	11	124761914	ROBO4	0.5	0.00432	2.36443	0.77841369	0.654748217	0.124
cg24860589	II	37	11	129941906	APLP2;APLP2;APLP2	0.71	4.57E-06	5.34042	0.80911761	0.932927918	-0.124
cg26244013	II	37	12	6470176	SCNN1A;SCNN1A;SC	0.46	0.01197	1.92207	0.78814887	0.664190321	0.124
cg20050826	II	37	12	52995295	KRT72;KRT72;KRT7	0.46	0.01197	1.92207	0.2633895	0.387130838	-0.124
cg14408356	I	37	12	113528845	DTX1	0.58	0.00041	3.39184	0.73006878	0.606189363	0.124
cg20566484	II	37	14	94489832		0.46	0.01197	1.92207	0.71553268	0.591604083	0.124
cg18371471	I	37	14	101029794	BEGAIN;BEGAIN	0.42	0.02991	1.52413	0.90526761	0.781051718	0.124
cg12395299	II	37	15	29349218	APBA2;APBA2	0.58	0.00041	3.39184	0.58544839	0.461063013	0.124
cg17594424	II	37	15	44086040	MIR1282;SERF2	0.54	0.0014	2.85345	0.66445579	0.788369235	-0.124
cg19169932	II	37	15	62861955		0.58	0.00041	3.39184	0.63942964	0.763728774	-0.124
cg22235407	I	37	15	66209223	MEGF11	0.46	0.01197	1.92207	0.80829694	0.684656124	0.124
cg14155397	II	37	15	66678782	MAP2K1	0.42	0.02991	1.52413	0.19394433	0.318393081	-0.124
cg20654462	II	37	15	93580092		0.42	0.02991	1.52413	0.6126642	0.737065872	-0.124
cg01922891	II	37	16	4714647	MGRN1;MGRN1;M	0.54	0.0014	2.85345	0.62137791	0.745413277	-0.124
cg02115302	II	37	16	27237719	NSMCE1	0.54	0.0014	2.85345	0.53137037	0.407469511	0.124
cg02697427	I	37	16	29196668		0.58	0.00041	3.39184	0.81248243	0.936063574	-0.124
cg09742346	II	37	16	81527501	CMIP;CMIP	0.54	0.0014	2.85345	0.70566182	0.829977971	-0.124
cg02185464	I	37	16	84867338	CRISPLD2	0.42	0.02991	1.52413	0.93082941	0.806982983	0.124

cg08732950	II	37	16	89023389	CBFA2T3	0.5	0.00432	2.36443	0.30975774	0.433375193	-0.124
cg14897188	II	37	17	6917185	RNASEK;C17orf49;(	0.42	0.02991	1.52413	0.66491969	0.540792	0.124
cg23365801	I	37	17	7832909	KCNAB3	0.42	0.02991	1.52413	0.70033819	0.576637472	0.124
cg12130768	II	37	17	9550545	USP43	0.46	0.01197	1.92207	0.46217606	0.337851083	0.124
cg17442269	II	37	17	15650385		0.46	0.01197	1.92207	0.7316227	0.607512879	0.124
cg16928487	II	37	17	17741425	SREBF1;SREBF1	0.5	0.00432	2.36443	0.33527752	0.458983346	-0.124
cg23682934	II	37	17	42885322	GJC1;GJC1	0.42	0.02991	1.52413	0.55072159	0.426249821	0.124
cg18402166	II	37	17	62778279	LOC146880;LOC146	0.54	0.0014	2.85345	0.6585944	0.782896822	-0.124
cg04912316	I	37	17	74266324	FAM100B	0.58	0.00041	3.39184	0.72081488	0.596404563	0.124
cg17922695	II	37	17	75451809	SEPT9;SEPT9;SEPT9	0.42	0.02991	1.52413	0.70518608	0.581441621	0.124
cg24759654	II	37	17	76801065	USP36	0.46	0.01197	1.92207	0.8402155	0.71609397	0.124
cg16115689	II	37	17	78764256	RPTOR;RPTOR	0.5	0.00432	2.36443	0.37983539	0.256227138	0.124
cg19563510	I	37	17	79881483	MAFG;MAFG	0.46	0.01197	1.92207	0.13359975	0.257356192	-0.124
cg12223090	II	37	17	80128788	CCDC57	0.62	0.0001	3.98291	0.74389687	0.86830091	-0.124
cg03956820	I	37	17	80190154	SLC16A3;SLC16A3;S	0.46	0.01197	1.92207	0.44449363	0.568603734	-0.124
cg24061208	II	37	18	14748250	ANKRD30B;ANKRD3	0.46	0.01197	1.92207	0.19821839	0.321743981	-0.124
cg17004025	II	37	19	840795	PRTN3	0.58	0.00041	3.39184	0.27145119	0.395543791	-0.124
cg26928195	I	37	19	1624734	TCF3;TCF3	0.62	0.0001	3.98291	0.61731161	0.741799269	-0.124
cg17617491	II	37	19	2607726	GNG7	0.46	0.01197	1.92207	0.69542173	0.571007206	0.124
cg26756949	I	37	19	4654722	TNFAIP8L1;TNFAIP8	0.5	0.00432	2.36443	0.76997258	0.646394267	0.124
cg16672562	I	37	19	46801672	HIF3A;HIF3A;HIF3A	0.46	0.01197	1.92207	0.57397247	0.6977601	-0.124
cg03078586	II	37	19	49469953	FTL	0.5	0.00432	2.36443	0.68936904	0.813329678	-0.124
cg02412123	II	37	19	51628204	SIGLEC9;SIGLEC9	0.67	2.34E-05	4.63072	0.31854361	0.442352945	-0.124
cg20962215	II	37	19	54713514		0.5	0.00432	2.36443	0.38958045	0.513102549	-0.124
cg13396318	II	37	20	19866974		0.58	0.00041	3.39184	0.60238027	0.726533295	-0.124
cg11173131	II	37	20	45179226	C20orf123	0.42	0.02991	1.52413	0.13880205	0.26312312	-0.124
cg10309886	II	37	20	57407747	GNASAS	0.42	0.02991	1.52413	0.25312008	0.377314109	-0.124
cg21221377	II	37	20	62574092	UCKL1;MIR1914;MI	0.54	0.0014	2.85345	0.35664559	0.480425712	-0.124
cg14065109	II	37	21	47715006	C21orf57	0.5	0.00432	2.36443	0.35842083	0.482846043	-0.124
cg06346307	II	37	22	19949965	COMT;COMT;COMT	0.46	0.01197	1.92207	0.55960686	0.43563334	0.124
cg03903451	II	37	22	44568913	PARVG;PARVG;PAR	0.62	0.0001	3.98291	0.53212768	0.655904311	-0.124
cg12251779	II	37	22	45608440	C22orf9;C22orf9	0.62	0.0001	3.98291	0.73502016	0.858939823	-0.124
cg23022809	II	37	1	2230923	SKI	0.54	0.0014	2.85345	0.67832704	0.800956057	-0.123

cg09820729	II	37	1	12039712	MFN2;MFN2	0.54	0.0014	2.85345	0.68336106	0.806268069	-0.123
cg07319459	II	37	1	17448608		0.42	0.02991	1.52413	0.6454607	0.522348234	0.123
cg03220543	II	37	1	18092930	ACTL8	0.54	0.0014	2.85345	0.53879877	0.415411613	0.123
cg11257888	II	37	1	24828096	RCAN3	0.5	0.00432	2.36443	0.6026827	0.479942894	0.123
cg18128887	I	37	1	24861708	RCAN3	0.54	0.0014	2.85345	0.24009014	0.116605311	0.123
cg06903031	II	37	1	110644949		0.58	0.00041	3.39184	0.34798023	0.47072102	-0.123
cg20946037	II	37	1	150582344		0.54	0.0014	2.85345	0.79512364	0.917682058	-0.123
cg21937128	II	37	1	150971889	FAM63A;FAM63A;F	0.46	0.01197	1.92207	0.3228131	0.44533724	-0.123
cg16097041	II	37	1	154965544	FLAD1;LENEP;FLAD	0.62	0.0001	3.98291	0.67767413	0.800609667	-0.123
cg27541604	II	37	1	159046451	AIM2;AIM2	0.42	0.02991	1.52413	0.71169422	0.588943109	0.123
cg20284239	II	37	1	168091000	GPR161	0.54	0.0014	2.85345	0.73481007	0.857809084	-0.123
cg17932662	II	37	1	202123442	PTPN7;PTPN7	0.54	0.0014	2.85345	0.74342244	0.866661556	-0.123
cg10901806	II	37	1	203055422	MYOG	0.54	0.0014	2.85345	0.73276819	0.609736306	0.123
cg01876531	II	37	1	208405868	PLXNA2	0.42	0.02991	1.52413	0.74532463	0.622393873	0.123
cg17163729	I	37	2	554372		0.5	0.00432	2.36443	0.4308985	0.308145873	0.123
cg24744014	II	37	2	11113304		0.46	0.01197	1.92207	0.68727013	0.564696259	0.123
cg19764540	II	37	2	26782423	OTOF	0.62	0.0001	3.98291	0.68911086	0.566126939	0.123
cg24032691	II	37	2	31030372	CAPN13	0.5	0.00432	2.36443	0.56255212	0.439311515	0.123
cg17639959	II	37	2	73297338	SFXN5	0.46	0.01197	1.92207	0.31340309	0.436376501	-0.123
cg25741837	I	37	2	73452813	SMYD5	0.54	0.0014	2.85345	0.40863512	0.531226581	-0.123
cg13878360	II	37	3	64069147		0.42	0.02991	1.52413	0.42733364	0.550000862	-0.123
cg11209289	II	37	3	138101415	MRAS;MRAS	0.54	0.0014	2.85345	0.84206365	0.71892251	0.123
cg07766263	II	37	3	170303045	SLC7A14	0.58	0.00041	3.39184	0.36235065	0.485567336	-0.123
cg24973755	I	37	4	1304972	MAEA;MAEA	0.46	0.01197	1.92207	0.7466326	0.623422565	0.123
cg13117948	II	37	4	16086051	PROM1;PROM1	0.58	0.00041	3.39184	0.25284898	0.375918547	-0.123
cg25251374	II	37	4	185305932		0.42	0.02991	1.52413	0.44636407	0.323369266	0.123
cg21747070	I	37	5	957535		0.67	2.34E-05	4.63072	0.47492695	0.352256534	0.123
cg20790798	II	37	5	1857306		0.46	0.01197	1.92207	0.53971419	0.662438946	-0.123
cg11080540	II	37	5	54897272		0.54	0.0014	2.85345	0.43110005	0.553700539	-0.123
cg05457480	II	37	5	139139201		0.5	0.00432	2.36443	0.20147988	0.324017179	-0.123
cg11100481	II	37	5	149867577		0.5	0.00432	2.36443	0.64203637	0.764717564	-0.123
cg01560676	II	37	5	172260799	ERGIC1	0.5	0.00432	2.36443	0.63861621	0.51545855	0.123
cg20001791	II	37	6	16239799	GMPR	0.42	0.02991	1.52413	0.24936189	0.371952553	-0.123

cg17670496	I	37	6	29759972	HCG4	0.5	0.00432	2.36443	0.09272033	0.215334586	-0.123
cg00252230	II	37	6	66013901	EYS	0.67	2.34E-05	4.63072	0.72409474	0.846862655	-0.123
cg23752752	I	37	7	4778908	FOXK1	0.5	0.00432	2.36443	0.52870691	0.652120307	-0.123
cg21341645	II	37	7	155091523	INSIG1;INSIG1;INSI(	0.42	0.02991	1.52413	0.52996391	0.406872631	0.123
cg08772302	II	37	8	37826337		0.54	0.0014	2.85345	0.28346129	0.406518377	-0.123
cg16231917	II	37	8	128930166	PVT1	0.5	0.00432	2.36443	0.38889418	0.512378171	-0.123
cg15628518	I	37	8	145025059	PLEC1;PLEC1;PLEC1	0.46	0.01197	1.92207	0.39301886	0.515992916	-0.123
cg13820205	II	37	9	132448040	PRRX2	0.5	0.00432	2.36443	0.75767622	0.634302295	0.123
cg06431514	II	37	10	11299076	CUGBP2;CUGBP2;C	0.46	0.01197	1.92207	0.65577942	0.532911506	0.123
cg10603156	II	37	10	13732625	FRMD4A	0.54	0.0014	2.85345	0.75342516	0.630162516	0.123
cg08834573	II	37	10	77005666		0.5	0.00432	2.36443	0.69002009	0.567389449	0.123
cg07081759	II	37	10	126330905	FAM53B	0.42	0.02991	1.52413	0.47438009	0.596985725	-0.123
cg08160246	II	37	11	2709914	KCNQ1;KCNQ1OT1;	0.46	0.01197	1.92207	0.60560149	0.482616462	0.123
cg26703066	II	37	11	10813944		0.42	0.02991	1.52413	0.53194883	0.409117197	0.123
cg19323289	II	37	11	19463903	NAV2	0.5	0.00432	2.36443	0.63332197	0.510590475	0.123
cg25402137	II	37	11	67792744	ALDH3B1;ALDH3B1	0.5	0.00432	2.36443	0.3938273	0.27057815	0.123
cg09868299	II	37	11	69259957		0.54	0.0014	2.85345	0.44242419	0.565104221	-0.123
cg13802316	I	37	11	70253460	CTTN;CTTN	0.5	0.00432	2.36443	0.77025158	0.893066097	-0.123
cg27154627	II	37	11	74398649		0.58	0.00041	3.39184	0.74543702	0.8680073	-0.123
cg03102841	II	37	11	85646010		0.46	0.01197	1.92207	0.57248946	0.695581942	-0.123
cg25354657	II	37	11	129991445	APLP2;APLP2;APLP2	0.54	0.0014	2.85345	0.63186864	0.508488184	0.123
cg20460652	II	37	12	132936672		0.46	0.01197	1.92207	0.43500627	0.311975572	0.123
cg19107655	I	37	13	112838528		0.62	0.0001	3.98291	0.68100228	0.803725965	-0.123
cg04272613	II	37	14	59721173	DAAM1	0.54	0.0014	2.85345	0.84759972	0.725038135	0.123
cg18490614	I	37	15	27213362		0.46	0.01197	1.92207	0.31532741	0.438621024	-0.123
cg19574915	II	37	15	89195555	ISG20	0.58	0.00041	3.39184	0.35840871	0.23492067	0.123
cg02405193	II	37	16	3639839	BTBD12	0.5	0.00432	2.36443	0.31142403	0.434467911	-0.123
cg04945379	II	37	16	10970200	CIITA	0.54	0.0014	2.85345	0.59076387	0.46783477	0.123
cg25341726	II	37	16	28518331	IL27	0.46	0.01197	1.92207	0.37368994	0.496710356	-0.123
cg03099790	II	37	16	56456455	AMFR	0.46	0.01197	1.92207	0.72751715	0.604319711	0.123
cg01932823	II	37	16	57701726	GPR97	0.5	0.00432	2.36443	0.61324156	0.736154713	-0.123
cg09652746	II	37	16	67225437	E2F4;EXOC3L	0.54	0.0014	2.85345	0.79313539	0.669854388	0.123
cg09685472	II	37	16	81527427	CMIP	0.42	0.02991	1.52413	0.49535382	0.618035662	-0.123

cg16672810	I	37	17	8815876	PIK3R5;PIK3R5	0.5	0.00432	2.36443	0.66327637	0.540183762	0.123
cg16533363	II	37	17	17267288		0.46	0.01197	1.92207	0.56705882	0.444449131	0.123
cg15246895	II	37	17	17672475	RAI1	0.58	0.00041	3.39184	0.53310909	0.655892842	-0.123
cg13832201	II	37	17	19282521	MAPK7;MAPK7;MA	0.67	2.34E-05	4.63072	0.6446037	0.767867943	-0.123
cg04384031	II	37	17	19631485		0.62	0.0001	3.98291	0.18344586	0.306405388	-0.123
cg05281206	II	37	17	32682770	CCL13	0.46	0.01197	1.92207	0.86433259	0.741597079	0.123
cg26611328	II	37	17	38698893		0.54	0.0014	2.85345	0.61821469	0.495291694	0.123
cg21400896	II	37	17	47288569	ABI3;ABI3	0.58	0.00041	3.39184	0.65820852	0.535069172	0.123
cg00041989	II	37	17	55828038		0.54	0.0014	2.85345	0.37663639	0.499931872	-0.123
cg24136318	I	37	17	75454130	SEPT9;SEPT9;SEPT9	0.75	7.61E-07	6.11857	0.40812333	0.285242596	0.123
cg17906851	I	37	17	78935070	RPTOR;RPTOR	0.46	0.01197	1.92207	0.93878161	0.81589677	0.123
cg26766064	II	37	17	79099711	MIR657;AATK;MIR3	0.42	0.02991	1.52413	0.74581174	0.62252538	0.123
cg11326429	I	37	17	79827632	ARHGDIA	0.58	0.00041	3.39184	0.64335866	0.520451978	0.123
cg23267759	I	37	18	19751335	GATA6	0.46	0.01197	1.92207	0.11681911	0.240148682	-0.123
cg04603976	II	37	19	4052706	ZBTB7A	0.62	0.0001	3.98291	0.29785262	0.420973538	-0.123
cg12110801	II	37	19	5992284		0.54	0.0014	2.85345	0.45856702	0.581700683	-0.123
cg01618851	II	37	19	7812265	CD209;CD209;CD2(	0.5	0.00432	2.36443	0.44016641	0.562966394	-0.123
cg12102973	II	37	19	13320368	CACNA1A;CACNA1/	0.42	0.02991	1.52413	0.67498385	0.551516906	0.123
cg01644611	I	37	20	3052253	OXT	0.5	0.00432	2.36443	0.72117282	0.843828293	-0.123
cg08587504	II	37	20	34639686	LOC647979	0.71	4.57E-06	5.34042	0.66650481	0.789511838	-0.123
cg21108851	II	37	21	43255700	PRDM15;PRDM15	0.58	0.00041	3.39184	0.89132147	0.768501949	0.123
cg02074274	II	37	21	45178331	PDXK	0.58	0.00041	3.39184	0.71784828	0.840919994	-0.123
cg01267908	II	37	21	46686908	POFUT2;POFUT2;P(	0.42	0.02991	1.52413	0.58043352	0.457714909	0.123
cg17016101	II	37	1	1907127	KIAA1751	0.58	0.00041	3.39184	0.71971692	0.842054065	-0.122
cg16585946	II	37	1	6831719		0.42	0.02991	1.52413	0.60200239	0.479727082	0.122
cg25978327	II	37	1	8065039		0.58	0.00041	3.39184	0.77987587	0.902326042	-0.122
cg19743168	II	37	1	23544995		0.46	0.01197	1.92207	0.23456962	0.356722365	-0.122
cg15712177	I	37	1	25236296	RUNX3;RUNX3	0.58	0.00041	3.39184	0.80174507	0.923527352	-0.122
cg17973115	II	37	1	25333445		0.42	0.02991	1.52413	0.20603777	0.327954918	-0.122
cg25203916	II	37	1	26392876	TRIM63	0.5	0.00432	2.36443	0.63750956	0.515492392	0.122
cg26328951	II	37	1	28906514	SNHG12;SNORD99;	0.46	0.01197	1.92207	0.78384732	0.66164951	0.122
cg16349331	II	37	1	32782341	HDAC1	0.46	0.01197	1.92207	0.69594802	0.573561697	0.122
cg05798664	II	37	1	36825645	STK40	0.46	0.01197	1.92207	0.53309115	0.411193075	0.122

cg12746947	II	37	1	38227094	EPHA10;EPHA10	0.5	0.00432	2.36443	0.59536521	0.717227079	-0.122
cg26038582	I	37	1	42384390	HIVEP3;HIVEP3;HIV	0.42	0.02991	1.52413	0.41621002	0.538639256	-0.122
cg14849855	II	37	1	67807960	IL12RB2	0.46	0.01197	1.92207	0.77284384	0.651258989	0.122
cg21859421	II	37	1	150969592	FAM63A;FAM63A;F	0.46	0.01197	1.92207	0.80720796	0.685488071	0.122
cg16564156	II	37	1	200859871	C1orf106	0.5	0.00432	2.36443	0.4698537	0.591750047	-0.122
cg00921097	II	37	1	202090980	GPR37L1	0.42	0.02991	1.52413	0.60119719	0.479065865	0.122
cg04830316	II	37	1	203465361	OPTC	0.46	0.01197	1.92207	0.59177838	0.469371419	0.122
cg01328473	II	37	2	1711966	PXDN	0.46	0.01197	1.92207	0.53224586	0.653852449	-0.122
cg13286582	I	37	2	37883934	CDC42EP3	0.46	0.01197	1.92207	0.56211762	0.440431539	0.122
cg08449164	II	37	2	54784402	SPTBN1;SPTBN1	0.5	0.00432	2.36443	0.66086513	0.538957514	0.122
cg05403316	II	37	2	55339939		0.42	0.02991	1.52413	0.18989764	0.312063963	-0.122
cg14900246	I	37	2	112655153	MERTK	0.42	0.02991	1.52413	0.59215499	0.713690217	-0.122
cg24980995	II	37	2	218219798	DIRC3	0.5	0.00432	2.36443	0.76421097	0.642430492	0.122
cg22286764	II	37	3	37428639		0.42	0.02991	1.52413	0.76774861	0.889686838	-0.122
cg02083676	II	37	3	127311156		0.46	0.01197	1.92207	0.63185531	0.754197504	-0.122
cg16098726	II	37	3	128778738	GP9	0.46	0.01197	1.92207	0.59754556	0.475249035	0.122
cg14051544	I	37	3	170303286	SLC7A14	0.58	0.00041	3.39184	0.10259024	0.224336663	-0.122
cg08822118	II	37	3	184037481	EIF4G1;EIF4G1;EIF4	0.58	0.00041	3.39184	0.62808782	0.750285923	-0.122
cg14180696	II	37	3	194089473	LRRC15;LRRC15	0.54	0.0014	2.85345	0.4308721	0.308612706	0.122
cg00150882	II	37	4	171011502	AADAT;AADAT	0.54	0.0014	2.85345	0.17646848	0.298240787	-0.122
cg18334345	II	37	4	184462457		0.62	0.0001	3.98291	0.83821804	0.716319266	0.122
cg24839529	II	37	4	185668393		0.46	0.01197	1.92207	0.53986942	0.662131044	-0.122
cg20682639	II	37	5	31799285	PDZD2;PDZD2	0.5	0.00432	2.36443	0.58920178	0.467401177	0.122
cg17645677	II	37	5	149185704	PPARGC1B	0.46	0.01197	1.92207	0.46333661	0.585461403	-0.122
cg16958939	I	37	6	7225223	RREB1;RREB1;RREB	0.58	0.00041	3.39184	0.31037978	0.432507717	-0.122
cg22146593	II	37	6	24919003		0.42	0.02991	1.52413	0.63315863	0.510727629	0.122
cg10199913	I	37	6	26045987	HIST1H3C	0.83	1.21E-08	7.91837	0.33807406	0.21613835	0.122
cg21366673	II	37	6	30459512	HLA-E	0.46	0.01197	1.92207	0.6867955	0.56484291	0.122
cg18020072	II	37	6	31590640	SNORA38;BAT2	0.5	0.00432	2.36443	0.68281755	0.804496619	-0.122
cg12484688	II	37	6	31868021	ZBTB12	0.46	0.01197	1.92207	0.30840729	0.430565466	-0.122
cg25426302	II	37	6	32120826	PPT2;PRRT1;PPT2	0.42	0.02991	1.52413	0.85056071	0.72883296	0.122
cg23598378	II	37	6	42072986	C6orf132	0.46	0.01197	1.92207	0.71257013	0.834753833	-0.122
cg06068369	II	37	6	105837504	PREP	0.5	0.00432	2.36443	0.7682813	0.890655259	-0.122

cg11387340	II	37	6	166970727	RPS6KA2;RPS6KA2	0.5	0.00432	2.36443	0.26129854	0.383555356	-0.122
cg05877104	I	37	6	167507447		0.58	0.00041	3.39184	0.5419364	0.420334838	0.122
cg27638126	II	37	7	15725322	MEOX2	0.54	0.0014	2.85345	0.09532312	0.21754896	-0.122
cg07537562	II	37	7	51539514		0.42	0.02991	1.52413	0.3225106	0.444773827	-0.122
cg20274462	II	37	8	95980625		0.62	0.0001	3.98291	0.25375196	0.375786777	-0.122
cg14752089	II	37	8	128773042		0.46	0.01197	1.92207	0.32033329	0.442641393	-0.122
cg00521239	II	37	8	145015964	PLEC1;PLEC1;PLEC1	0.46	0.01197	1.92207	0.25964527	0.137522188	0.122
cg10769767	II	37	9	37024153	PAX5	0.54	0.0014	2.85345	0.54607631	0.42401247	0.122
cg14018735	II	37	9	136270734	C9orf96	0.58	0.00041	3.39184	0.49665138	0.619141638	-0.122
cg06902558	I	37	10	3141803	PFKP	0.58	0.00041	3.39184	0.78699798	0.908891256	-0.122
cg25907132	I	37	10	51489510		0.42	0.02991	1.52413	0.08291736	0.204636194	-0.122
cg00661347	II	37	10	71661228	COL13A1;COL13A1;	0.5	0.00432	2.36443	0.58591213	0.464215981	0.122
cg26679004	I	37	10	88023135	GRID1	0.46	0.01197	1.92207	0.22740395	0.349216369	-0.122
cg00547480	II	37	10	103875961	LDB1;LDB1	0.46	0.01197	1.92207	0.53364929	0.656071423	-0.122
cg26969179	II	37	10	127738328	ADAM12;ADAM12	0.58	0.00041	3.39184	0.31063853	0.432314987	-0.122
cg06603309	I	37	11	2724144	KCNQ1;KCNQ1	0.54	0.0014	2.85345	0.65342491	0.531790638	0.122
cg20897479	II	37	11	12292577		0.5	0.00432	2.36443	0.68769438	0.566071309	0.122
cg14742715	II	37	11	18286807	SAA1;SAA1	0.54	0.0014	2.85345	0.15101504	0.273332253	-0.122
cg09878888	II	37	11	57529614	CTNND1;CTNND1;C	0.46	0.01197	1.92207	0.10845433	0.230920943	-0.122
cg07298431	II	37	11	129817649	PRDM10;PRDM10;I	0.58	0.00041	3.39184	0.48814403	0.366099115	0.122
cg07925587	II	37	12	52583324	KRT80;KRT80	0.46	0.01197	1.92207	0.16432422	0.286658924	-0.122
cg14212748	II	37	12	53343703	KRT18;KRT18	0.54	0.0014	2.85345	0.50767336	0.630057325	-0.122
cg22630748	II	37	12	57848870	INHBE	0.54	0.0014	2.85345	0.61843794	0.740501174	-0.122
cg08514385	II	37	13	32596585		0.54	0.0014	2.85345	0.73742091	0.859843657	-0.122
cg09469566	I	37	13	112717244		0.42	0.02991	1.52413	0.1839336	0.3054416	-0.122
cg02318784	II	37	15	27213174		0.46	0.01197	1.92207	0.29562943	0.417773983	-0.122
cg07097876	II	37	15	71409997		0.46	0.01197	1.92207	0.63781648	0.515651175	0.122
cg10850792	II	37	15	89531046		0.42	0.02991	1.52413	0.57832511	0.69984369	-0.122
cg06219695	II	37	16	434256	LOC100134368	0.58	0.00041	3.39184	0.29915589	0.421653925	-0.122
cg27190398	I	37	16	731844	STUB1;JMJD8	0.5	0.00432	2.36443	0.60831955	0.729936464	-0.122
cg02485642	I	37	16	832958	MSLNL	0.46	0.01197	1.92207	0.85468681	0.732876387	0.122
cg26598348	II	37	16	875626		0.5	0.00432	2.36443	0.59869715	0.476296014	0.122
cg09234161	II	37	16	57565147	CCDC102A	0.46	0.01197	1.92207	0.32502341	0.447051124	-0.122

cg04851702	II	37	16	81528974	CMIP;CMIP;CMIP	0.54	0.0014	2.85345	0.25253496	0.37491963	-0.122
cg26655856	II	37	16	88977887	CBFA2T3;CBFA2T3	0.58	0.00041	3.39184	0.51885391	0.396880536	0.122
cg19504184	II	37	17	1170806		0.58	0.00041	3.39184	0.21872209	0.341205328	-0.122
cg23403980	II	37	17	2060623	SMG6;SMG6	0.54	0.0014	2.85345	0.32686393	0.448512717	-0.122
cg17579446	II	37	17	7217367	GPS2	0.46	0.01197	1.92207	0.68376813	0.561387743	0.122
cg04118102	II	37	17	15824118		0.58	0.00041	3.39184	0.780149	0.901819554	-0.122
cg13274938	II	37	17	38493822	RARA;RARA;RARA	0.54	0.0014	2.85345	0.67749733	0.799628132	-0.122
cg08783253	II	37	17	40996565	AOC2;AOC2	0.54	0.0014	2.85345	0.62832297	0.750095943	-0.122
cg16861241	I	37	17	74138396	FOXJ1	0.58	0.00041	3.39184	0.57465393	0.696954705	-0.122
cg14210726	II	37	17	76136952	TMC8	0.46	0.01197	1.92207	0.52916934	0.651543313	-0.122
cg03249047	II	37	17	80872472	TBCD	0.42	0.02991	1.52413	0.57881308	0.456703022	0.122
cg10904070	II	37	18	3012017	LPIN2	0.5	0.00432	2.36443	0.67574273	0.798220815	-0.122
cg12230709	I	37	19	840873	PRTN3	0.5	0.00432	2.36443	0.2758351	0.397485952	-0.122
cg08700306	II	37	19	33686390	LRP3	0.58	0.00041	3.39184	0.38625782	0.507930521	-0.122
cg03301498	II	37	19	39889222	MED29	0.5	0.00432	2.36443	0.28601989	0.407888885	-0.122
cg24446326	II	37	20	19952018	RIN2	0.62	0.0001	3.98291	0.71773343	0.839694627	-0.122
cg15133734	II	37	20	48728113	UBE2V1;UBE2V1;UI	0.5	0.00432	2.36443	0.46846144	0.346602309	0.122
cg08480461	II	37	1	39553001	MACF1	0.62	0.0001	3.98291	0.33746145	0.458222033	-0.121
cg20168837	II	37	1	54562213	C1orf83	0.42	0.02991	1.52413	0.47835694	0.598872542	-0.121
cg02573357	II	37	1	203738799	LAX1;LAX1	0.42	0.02991	1.52413	0.26304041	0.384055567	-0.121
cg04323713	II	37	1	212738603	ATF3	0.46	0.01197	1.92207	0.85431714	0.733265109	0.121
cg11847933	II	37	1	227130394	CABC1	0.42	0.02991	1.52413	0.67600931	0.797043151	-0.121
cg24312489	II	37	1	228109169	WNT9A	0.54	0.0014	2.85345	0.60876634	0.488163396	0.121
cg24756642	II	37	2	1597123		0.5	0.00432	2.36443	0.7633348	0.642214544	0.121
cg24837370	II	37	2	5833774	SOX11	0.5	0.00432	2.36443	0.15201066	0.272895282	-0.121
cg05627557	II	37	2	37418009		0.5	0.00432	2.36443	0.68300897	0.804077708	-0.121
cg19220272	II	37	2	43312367		0.54	0.0014	2.85345	0.2190702	0.340116228	-0.121
cg17803713	II	37	2	109873891	SH3RF3	0.54	0.0014	2.85345	0.54055408	0.661770096	-0.121
cg01750780	I	37	2	131451266		0.42	0.02991	1.52413	0.36265143	0.241747969	0.121
cg09287629	II	37	2	203036208		0.46	0.01197	1.92207	0.1803164	0.30161744	-0.121
cg11886884	II	37	3	138062860		0.42	0.02991	1.52413	0.69932726	0.577916398	0.121
cg18623216	II	37	3	155421970	PLCH1;PLCH1	0.5	0.00432	2.36443	0.14613094	0.267376304	-0.121
cg08704934	I	37	3	194826585	C3orf21	0.46	0.01197	1.92207	0.78314573	0.903998366	-0.121

cg09225701	II	37	4	8248877		0.67	2.34E-05	4.63072	0.75549959	0.876619146	-0.121
cg00876141	II	37	4	47837775	CORIN	0.54	0.0014	2.85345	0.32930254	0.449847114	-0.121
cg07671752	II	37	5	176734858	MXD3;MXD3	0.58	0.00041	3.39184	0.55002988	0.671289348	-0.121
cg25550425	II	37	5	178645682	ADAMTS2;ADAMTS	0.5	0.00432	2.36443	0.82826288	0.707248264	0.121
cg03680932	I	37	6	16306683	ATXN1;ATXN1	0.54	0.0014	2.85345	0.90288288	0.781765091	0.121
cg23565821	II	37	6	33385056	CUTA;CUTA;CUTA;(	0.46	0.01197	1.92207	0.31987245	0.440789679	-0.121
cg20201675	II	37	6	112230657		0.5	0.00432	2.36443	0.68650529	0.8074834	-0.121
cg25291978	II	37	6	144386422	PLAGL1;PLAGL1	0.5	0.00432	2.36443	0.80178032	0.680689599	0.121
cg01851450	II	37	6	146864335	RAB32	0.54	0.0014	2.85345	0.10180818	0.222662459	-0.121
cg23560388	II	37	6	155542639	TIAM2;TIAM2	0.54	0.0014	2.85345	0.77642563	0.897173623	-0.121
cg09462806	II	37	7	150084		0.58	0.00041	3.39184	0.16825507	0.289350902	-0.121
cg02123534	II	37	7	965485	ADAP1	0.5	0.00432	2.36443	0.37980892	0.500521061	-0.121
cg23093589	II	37	7	20824932	SP8;SP8	0.42	0.02991	1.52413	0.14412072	0.265559231	-0.121
cg10421188	I	37	7	28824953	CREB5;CREB5;CREB	0.46	0.01197	1.92207	0.78554695	0.90665247	-0.121
cg18429196	II	37	7	73609326	EIF4H;EIF4H	0.62	0.0001	3.98291	0.66340087	0.784493772	-0.121
cg17115737	II	37	7	75580813	POR	0.58	0.00041	3.39184	0.75714599	0.878623937	-0.121
cg21298978	II	37	8	143961391	CYP11B1;CYP11B1	0.42	0.02991	1.52413	0.7331018	0.612059748	0.121
cg02679745	II	37	9	139927646	C9orf139;FUT7	0.54	0.0014	2.85345	0.25882645	0.380076125	-0.121
cg13871921	I	37	9	140076357	ANAPC2	0.58	0.00041	3.39184	0.83370986	0.954765078	-0.121
cg13741304	II	37	9	140501305	ARRDC1	0.46	0.01197	1.92207	0.58360145	0.704575191	-0.121
cg17460183	II	37	10	48370158	ZNF488	0.46	0.01197	1.92207	0.68608888	0.564680275	0.121
cg06219732	II	37	10	72118317	LRRC20;LRRC20;LRI	0.46	0.01197	1.92207	0.69712824	0.5756595	0.121
cg13740185	I	37	10	73486801	C10orf105;CDH23	0.83	1.21E-08	7.91837	0.43972697	0.31860041	0.121
cg03110921	II	37	10	79597461	DLG5	0.42	0.02991	1.52413	0.7299466	0.609420879	0.121
cg25104397	II	37	10	104535920	C10orf26;C10orf26	0.5	0.00432	2.36443	0.30296027	0.424053963	-0.121
cg24644436	I	37	10	135202906	PAOX;PAOX;PAOX	0.5	0.00432	2.36443	0.27944761	0.158709346	0.121
cg27072025	II	37	10	135203200	PAOX;PAOX;PAOX	0.5	0.00432	2.36443	0.4105605	0.289388629	0.121
cg17990365	II	37	11	319718	IFITM3	0.62	0.0001	3.98291	0.56749124	0.688722486	-0.121
cg07824422	II	37	11	2555406	KCNQ1;KCNQ1	0.5	0.00432	2.36443	0.34060989	0.461782015	-0.121
cg22961513	II	37	11	14280813	SPON1	0.5	0.00432	2.36443	0.47763434	0.598253194	-0.121
cg13255398	II	37	11	16836827	PLEKHA7	0.54	0.0014	2.85345	0.22398321	0.344637903	-0.121
cg27395922	I	37	11	50257633	LOC441601	0.5	0.00432	2.36443	0.64286804	0.764182166	-0.121
cg20179907	II	37	11	72336908	PDE2A;PDE2A;PDE2	0.54	0.0014	2.85345	0.45217576	0.573457299	-0.121

cg09799980	II	37	12	4398618	CCND2	0.58	0.00041	3.39184	0.24382435	0.364987117	-0.121
cg19942083	I	37	12	7070562		0.5	0.00432	2.36443	0.18937577	0.068713193	0.121
cg07855572	II	37	12	55028956	LACRT	0.58	0.00041	3.39184	0.7392028	0.618150214	0.121
cg12564437	II	37	12	96464899		0.42	0.02991	1.52413	0.52115979	0.400230812	0.121
cg20884605	II	37	12	112205368	ALDH2	0.42	0.02991	1.52413	0.22598909	0.346940257	-0.121
cg08658787	I	37	12	113916646		0.5	0.00432	2.36443	0.52710269	0.64820449	-0.121
cg05957567	I	37	12	122467228	BCL7A;BCL7A	0.54	0.0014	2.85345	0.66076032	0.540110636	0.121
cg09719269	II	37	12	125033803		0.54	0.0014	2.85345	0.63600484	0.756518906	-0.121
cg17582615	II	37	13	44986657		0.54	0.0014	2.85345	0.66921295	0.789989719	-0.121
cg16238149	II	37	13	111173329		0.46	0.01197	1.92207	0.20123307	0.322525541	-0.121
cg16312609	II	37	14	65801447		0.42	0.02991	1.52413	0.75179926	0.630458725	0.121
cg04977856	II	37	14	89886941	FOXN3	0.5	0.00432	2.36443	0.77163141	0.892171696	-0.121
cg14019523	II	37	14	94407033	ASB2	0.54	0.0014	2.85345	0.66054331	0.781918094	-0.121
cg04179148	II	37	14	94858339	SERPINA1;SERPINA	0.62	0.0001	3.98291	0.6131709	0.734482436	-0.121
cg23592421	II	37	14	105147461		0.42	0.02991	1.52413	0.29738648	0.418790209	-0.121
cg24971846	I	37	14	105618576	JAG2;JAG2	0.46	0.01197	1.92207	0.7764018	0.655680618	0.121
cg18545991	II	37	15	45740677		0.46	0.01197	1.92207	0.75580799	0.877144419	-0.121
cg01380884	II	37	15	74923934	EDC3;EDC3;EDC3	0.58	0.00041	3.39184	0.67297686	0.793758725	-0.121
cg17846127	II	37	15	89940505	LOC254559	0.46	0.01197	1.92207	0.67480481	0.796151377	-0.121
cg08438529	I	37	16	1052939		0.62	0.0001	3.98291	0.62385048	0.503070321	0.121
cg01489441	I	37	16	1764111	MAPK8IP3;MAPK8I	0.54	0.0014	2.85345	0.37345382	0.252257427	0.121
cg12031863	II	37	16	4587854	C16orf5	0.62	0.0001	3.98291	0.31106443	0.432503725	-0.121
cg05846851	II	37	16	10172054	GRIN2A;GRIN2A;GF	0.5	0.00432	2.36443	0.73836831	0.617482783	0.121
cg09109383	II	37	16	16083164	ABCC1;ABCC1;ABC(	0.54	0.0014	2.85345	0.58794482	0.708733081	-0.121
cg01025883	II	37	16	23867088	PRKCB;PRKCB	0.5	0.00432	2.36443	0.67792293	0.798511683	-0.121
cg26864036	I	37	16	83974716		0.54	0.0014	2.85345	0.76044324	0.639075062	0.121
cg06652210	II	37	17	1944594	OVCA2;DPH1	0.58	0.00041	3.39184	0.63816	0.759500899	-0.121
cg10193804	II	37	17	16876525	TNFRSF13B	0.42	0.02991	1.52413	0.43859926	0.317892963	0.121
cg18410271	II	37	17	43472435	ARHGAP27;ARHGAI	0.54	0.0014	2.85345	0.41964432	0.540546847	-0.121
cg05363382	II	37	17	45767653		0.5	0.00432	2.36443	0.7706324	0.649553702	0.121
cg22632947	II	37	17	64787784	PRKCA	0.46	0.01197	1.92207	0.388116	0.267318145	0.121
cg11158979	I	37	17	80557661	FOXK2	0.46	0.01197	1.92207	0.85437851	0.733779421	0.121
cg01859460	II	37	19	2273050	OAZ1	0.46	0.01197	1.92207	0.68538241	0.564871212	0.121

cg22898082	II	37	19	11074428	SMARCA4;SMARCA	0.67	2.34E-05	4.63072	0.55854579	0.43746521	0.121
cg22617898	I	37	19	18313388	RAB3A	0.67	2.34E-05	4.63072	0.735007	0.856498468	-0.121
cg27285056	II	37	19	50868879	NAPSA;NAPSA	0.42	0.02991	1.52413	0.74920835	0.628290425	0.121
cg16190478	II	37	21	45789122	TRPM2	0.58	0.00041	3.39184	0.51277463	0.634075345	-0.121
cg14112356	II	37	21	46348443	ITGB2	0.5	0.00432	2.36443	0.57050892	0.449594428	0.121
cg26666978	II	37	21	47038531		0.5	0.00432	2.36443	0.25599044	0.377455434	-0.121
cg27200006	II	37	22	25003854	GGT1;GGT1;GGT1;(	0.5	0.00432	2.36443	0.28071423	0.401291646	-0.121
cg11837181	I	37	1	3001002	PRDM16;PRDM16	0.58	0.00041	3.39184	0.71724219	0.597031175	0.12
cg15059176	II	37	1	3306447	PRDM16;PRDM16	0.42	0.02991	1.52413	0.74021676	0.620535876	0.12
cg11186344	I	37	1	19253694	IFFO2	0.67	2.34E-05	4.63072	0.4036387	0.283698392	0.12
cg20777437	II	37	1	54619703	CDCP2	0.67	2.34E-05	4.63072	0.66749991	0.787757004	-0.12
cg13369817	II	37	1	55116866	C1orf175;C1orf175	0.54	0.0014	2.85345	0.79728979	0.677752641	0.12
cg24429974	I	37	1	161195180	TOMM40L	0.54	0.0014	2.85345	0.72866391	0.848577483	-0.12
cg17814814	II	37	1	222060676		0.54	0.0014	2.85345	0.69252036	0.812871552	-0.12
cg26614229	II	37	1	231156204	MIR1182;FAM89A	0.46	0.01197	1.92207	0.1538231	0.274221949	-0.12
cg12641739	II	37	1	235062535		0.46	0.01197	1.92207	0.68566288	0.565383742	0.12
cg08579962	II	37	2	445224		0.46	0.01197	1.92207	0.69235	0.572631611	0.12
cg09034753	II	37	2	242810748	C2orf85	0.42	0.02991	1.52413	0.60064682	0.480162917	0.12
cg25214561	II	37	3	11494984	ATG7;ATG7;ATG7	0.62	0.0001	3.98291	0.72330807	0.843241715	-0.12
cg16143049	II	37	3	124768294	HEG1	0.54	0.0014	2.85345	0.48654265	0.607024237	-0.12
cg07853489	II	37	3	183411982		0.5	0.00432	2.36443	0.36611997	0.486130694	-0.12
cg08116915	II	37	4	8597048	CPZ;CPZ;CPZ	0.5	0.00432	2.36443	0.77179167	0.651734082	0.12
cg00177388	II	37	5	72677886		0.5	0.00432	2.36443	0.14472044	0.264224852	-0.12
cg10205431	I	37	5	140215762	PCDHA6;PCDHA2;P	0.58	0.00041	3.39184	0.80226857	0.922568914	-0.12
cg15621178	II	37	5	148663537	AFAP1L1;AFAP1L1	0.58	0.00041	3.39184	0.77520362	0.655619511	0.12
cg02711886	II	37	5	177762428	COL23A1	0.67	2.34E-05	4.63072	0.29932752	0.419180276	-0.12
cg26525121	II	37	6	14129370	CD83;CD83	0.46	0.01197	1.92207	0.83584513	0.715380902	0.12
cg20605134	I	37	6	15400462	JARID2	0.42	0.02991	1.52413	0.29678177	0.4169074	-0.12
cg08415973	II	37	6	40346114	TDRG1	0.5	0.00432	2.36443	0.34295048	0.462700693	-0.12
cg09199562	I	37	7	182369		0.42	0.02991	1.52413	0.8447078	0.725083395	0.12
cg10022248	I	37	7	1686836		0.5	0.00432	2.36443	0.63140807	0.511156269	0.12
cg24939194	II	37	7	2563611	LFNG;LFNG;LFNG;LI	0.54	0.0014	2.85345	0.59093772	0.711120719	-0.12
cg12362478	II	37	7	32497799		0.46	0.01197	1.92207	0.26773429	0.387638711	-0.12

cg26460483	II	37	7	97914016	BRI3;BRI3	0.67	2.34E-05	4.63072	0.30846855	0.428532037	-0.12
cg00374672	II	37	7	100463416	SLC12A9	0.58	0.00041	3.39184	0.21252813	0.332149753	-0.12
cg12086281	II	37	7	134924584	STRA8	0.46	0.01197	1.92207	0.80706066	0.686923764	0.12
cg11848254	II	37	7	134939464	STRA8	0.54	0.0014	2.85345	0.22583782	0.346221838	-0.12
cg06333800	II	37	7	151442481	PRKAG2;PRKAG2	0.46	0.01197	1.92207	0.38047579	0.500209737	-0.12
cg03978658	II	37	8	37761565		0.58	0.00041	3.39184	0.27746668	0.397167293	-0.12
cg16408820	II	37	8	134165361		0.46	0.01197	1.92207	0.76301237	0.642901674	0.12
cg15920669	II	37	8	142037502		0.62	0.0001	3.98291	0.7212525	0.84152149	-0.12
cg10632966	II	37	10	105001051		0.54	0.0014	2.85345	0.79553938	0.915632119	-0.12
cg17333973	II	37	11	2847019	KCNQ1;KCNQ1	0.62	0.0001	3.98291	0.39067698	0.510807709	-0.12
cg09039751	II	37	11	45672100	CHST1	0.58	0.00041	3.39184	0.56169332	0.682158426	-0.12
cg07267600	II	37	12	2750053	CACNA1C;CACNA1(	0.46	0.01197	1.92207	0.31022394	0.430152225	-0.12
cg00156995	II	37	13	41055588	LOC646982;LOC64(	0.54	0.0014	2.85345	0.45064757	0.330459953	0.12
cg03917803	II	37	13	44947247	SERP2	0.46	0.01197	1.92207	0.58807152	0.708126543	-0.12
cg16229875	II	37	14	23589066	CEBPE	0.46	0.01197	1.92207	0.65999951	0.780353859	-0.12
cg04193065	II	37	15	31528995		0.46	0.01197	1.92207	0.5286167	0.648834219	-0.12
cg27141517	II	37	15	66923526		0.62	0.0001	3.98291	0.38727016	0.5071332	-0.12
cg19570897	II	37	15	90191316	KIF7	0.5	0.00432	2.36443	0.60991253	0.729932134	-0.12
cg27576259	II	37	16	2567316	ATP6V0C	0.5	0.00432	2.36443	0.72321576	0.843086023	-0.12
cg16520815	I	37	16	4733181	MGRN1;MGRN1;M(	0.46	0.01197	1.92207	0.6795838	0.799520144	-0.12
cg09781650	II	37	16	66285054		0.5	0.00432	2.36443	0.76129729	0.641397602	0.12
cg27614309	II	37	16	85478580		0.5	0.00432	2.36443	0.69606352	0.575844623	0.12
cg01898661	I	37	16	90114063	LOC100130015;LO(	0.46	0.01197	1.92207	0.06503194	0.184771956	-0.12
cg23630423	II	37	17	1903022	RTN4RL1	0.54	0.0014	2.85345	0.50531239	0.625217816	-0.12
cg04324276	II	37	17	17817462	TOM1L2;TOM1L2	0.62	0.0001	3.98291	0.31101337	0.43127373	-0.12
cg26829529	II	37	17	31317536	SPACA3	0.5	0.00432	2.36443	0.76226403	0.64247456	0.12
cg25306838	II	37	17	39597402	KRT38;KRT38	0.54	0.0014	2.85345	0.73059179	0.610900524	0.12
cg19982668	II	37	17	61753497	MAP3K3;MAP3K3	0.58	0.00041	3.39184	0.32521947	0.445300943	-0.12
cg11183935	I	37	17	80733167	TBCD	0.46	0.01197	1.92207	0.75342527	0.633259493	0.12
cg21088259	II	37	17	81039990	METRNL	0.46	0.01197	1.92207	0.55927623	0.679387409	-0.12
cg25627226	II	37	18	4455337		0.58	0.00041	3.39184	0.09016722	0.210628529	-0.12
cg00220661	II	37	18	74241269	LOC284276	0.58	0.00041	3.39184	0.11377954	0.234101783	-0.12
cg02194396	II	37	19	427162	SHC2	0.62	0.0001	3.98291	0.51899137	0.638548911	-0.12

cg08676438	II	37	19	1763695	ONECUT3	0.58	0.00041	3.39184	0.42182926	0.542176268	-0.12
cg24800754	II	37	19	6234327	MLLT1	0.5	0.00432	2.36443	0.20505984	0.325477678	-0.12
cg19913465	II	37	19	7767075	FCER2	0.46	0.01197	1.92207	0.36795377	0.248053814	0.12
cg18093635	II	37	19	14890139	EMR2;EMR2;EMR2;	0.5	0.00432	2.36443	0.61331477	0.733773821	-0.12
cg10738025	II	37	19	19702508	PBX4	0.5	0.00432	2.36443	0.85938221	0.739469858	0.12
cg06538345	II	37	19	49993186	RPL13AP5;SNORD3	0.42	0.02991	1.52413	0.69902598	0.578962674	0.12
cg25537993	I	37	19	58545182	ZSCAN1	0.46	0.01197	1.92207	0.13073595	0.250730521	-0.12
cg08642068	II	37	20	31591776	SPAG4L	0.42	0.02991	1.52413	0.74073911	0.861154389	-0.12
cg14679444	I	37	22	50470101	TTLL8	0.42	0.02991	1.52413	0.8112356	0.691126733	0.12
cg08945450	II	37	1	11795905	AGTRAP;AGTRAP;A	0.58	0.00041	3.39184	0.33415116	0.453293215	-0.119
cg07236781	I	37	1	25291041	RUNX3	0.42	0.02991	1.52413	0.85771247	0.738725366	0.119
cg10776244	II	37	1	28916695		0.58	0.00041	3.39184	0.31714147	0.435769277	-0.119
cg07979236	II	37	1	33516461		0.54	0.0014	2.85345	0.51088053	0.629800661	-0.119
cg04362706	II	37	1	57570305	DAB1	0.54	0.0014	2.85345	0.78498942	0.665850531	0.119
cg04079215	II	37	1	95095221		0.46	0.01197	1.92207	0.61835711	0.737229911	-0.119
cg12957986	II	37	1	111061829	KCNA10	0.46	0.01197	1.92207	0.69175603	0.57305035	0.119
cg17904988	II	37	1	161168451	NDUFS2;ADAMTS4;	0.5	0.00432	2.36443	0.34690239	0.466133602	-0.119
cg13101705	II	37	1	183248593	NMNAT2;NMNAT2	0.58	0.00041	3.39184	0.41147927	0.530093877	-0.119
cg26033504	II	37	1	201458737	CSRP1;CSRP1	0.62	0.0001	3.98291	0.71299863	0.831740654	-0.119
cg16986578	II	37	1	201979567	ELF3;ELF3	0.42	0.02991	1.52413	0.77401738	0.655265955	0.119
cg27281690	II	37	2	7171963	RNF144A	0.5	0.00432	2.36443	0.35229288	0.470826791	-0.119
cg19139589	II	37	2	25149093		0.5	0.00432	2.36443	0.57153878	0.690985976	-0.119
cg02039404	II	37	2	70368784		0.54	0.0014	2.85345	0.83735708	0.717886669	0.119
cg13355542	II	37	2	97199248		0.5	0.00432	2.36443	0.19845155	0.317027244	-0.119
cg19914238	II	37	2	113483728		0.42	0.02991	1.52413	0.84972992	0.731165199	0.119
cg07719512	II	37	2	219246576	SLC11A1	0.46	0.01197	1.92207	0.39916084	0.518381873	-0.119
cg27104695	II	37	3	15345841	SH3BP5;SH3BP5	0.46	0.01197	1.92207	0.4809089	0.362011051	0.119
cg17619311	I	37	3	42947565	ZNF662;ZNF662;ZN	0.46	0.01197	1.92207	0.12638585	0.245587208	-0.119
cg13344886	II	37	3	45167106	CDCP1;CDCP1	0.42	0.02991	1.52413	0.82100713	0.702036061	0.119
cg22090713	II	37	3	50379032	RASSF1;ZMYND10;I	0.5	0.00432	2.36443	0.38116214	0.499952105	-0.119
cg17408686	II	37	3	126422575	CHCHD6	0.42	0.02991	1.52413	0.34406172	0.463232213	-0.119
cg25197194	II	37	3	128758787	CCDC48	0.54	0.0014	2.85345	0.54888101	0.668318901	-0.119
cg09436767	II	37	3	184035154	EIF4G1;EIF4G1;EIF4	0.54	0.0014	2.85345	0.49815545	0.617372023	-0.119

cg25441176	I	37	4	178417930		0.79	1.06E-07	6.97389	0.47928027	0.360484747	0.119
cg23915282	II	37	5	135547572		0.5	0.00432	2.36443	0.73503765	0.616154003	0.119
cg13598881	II	37	6	2783665	WRNIP1;WRNIP1	0.42	0.02991	1.52413	0.62257691	0.74133821	-0.119
cg19886655	II	37	6	2958852	SERPINB6	0.5	0.00432	2.36443	0.19011335	0.308781716	-0.119
cg08560874	II	37	6	29759947	HCG4	0.54	0.0014	2.85345	0.18163794	0.300513265	-0.119
cg18950108	II	37	6	30920171	DPCR1	0.42	0.02991	1.52413	0.73821828	0.619486019	0.119
cg14306709	II	37	6	31547704		0.54	0.0014	2.85345	0.60306508	0.483901241	0.119
cg09080120	II	37	6	33246105	B3GALT4	0.42	0.02991	1.52413	0.69630433	0.577368275	0.119
cg18291422	I	37	6	147171560		0.46	0.01197	1.92207	0.83426596	0.7155584	0.119
cg05698763	II	37	7	91685		0.5	0.00432	2.36443	0.84331649	0.724739222	0.119
cg00541718	II	37	7	630823	PRKAR1B;PRKAR1B	0.42	0.02991	1.52413	0.54781326	0.667138455	-0.119
cg00511027	II	37	7	1062887	C7orf50;C7orf50;M	0.5	0.00432	2.36443	0.58670895	0.467546883	0.119
cg23367351	II	37	7	2106029	MAD1L1;MAD1L1;M	0.5	0.00432	2.36443	0.72489661	0.605649258	0.119
cg04919592	II	37	7	2607232	IQCE;IQCE	0.42	0.02991	1.52413	0.68810477	0.80661154	-0.119
cg18814699	II	37	7	36342851		0.67	2.34E-05	4.63072	0.7314199	0.850543929	-0.119
cg12361987	II	37	7	50109467	ZPBP;ZPBP	0.62	0.0001	3.98291	0.12918226	0.247708698	-0.119
cg00601368	II	37	7	149318081	ZNF767;ZNF767	0.46	0.01197	1.92207	0.57761993	0.458233647	0.119
cg08525461	II	37	8	40958085		0.46	0.01197	1.92207	0.70917587	0.589774735	0.119
cg16499923	I	37	8	142183642	DENND3	0.46	0.01197	1.92207	0.49823778	0.617144078	-0.119
cg11277662	II	37	8	143408047	TSNARE1	0.42	0.02991	1.52413	0.52217886	0.640845057	-0.119
cg18198461	II	37	8	143523293		0.54	0.0014	2.85345	0.52828809	0.409067656	0.119
cg13394182	II	37	8	144403454	TOP1MT	0.58	0.00041	3.39184	0.64271315	0.762187784	-0.119
cg06617876	II	37	9	139652874	LCN8	0.54	0.0014	2.85345	0.34422181	0.224839664	0.119
cg12038298	II	37	11	1951814	TNNT3;TNNT3;TNN	0.58	0.00041	3.39184	0.75212658	0.632820139	0.119
cg17696044	II	37	11	70449316	SHANK2;SHANK2	0.58	0.00041	3.39184	0.18821665	0.306782046	-0.119
cg23077606	II	37	11	70540206	SHANK2	0.5	0.00432	2.36443	0.71049523	0.591103376	0.119
cg04757806	I	37	11	94278595	FUT4	0.58	0.00041	3.39184	0.56575179	0.68426209	-0.119
cg22779972	I	37	12	3207241	TSPAN9;TSPAN9	0.42	0.02991	1.52413	0.86225023	0.743364101	0.119
cg07533239	II	37	12	6422131	PLEKHG6;PLEKHG6;	0.5	0.00432	2.36443	0.58520616	0.466136078	0.119
cg20114528	II	37	12	48552006	ASB8	0.54	0.0014	2.85345	0.76513063	0.646048422	0.119
cg02399464	II	37	12	50482541	SMARCD1;SMARCD	0.54	0.0014	2.85345	0.4138915	0.295379471	0.119
cg10097651	II	37	14	35802397		0.42	0.02991	1.52413	0.28387239	0.403318684	-0.119
cg13097800	II	37	14	47104140		0.5	0.00432	2.36443	0.60217652	0.48335561	0.119

cg11820833	II	37	14	65187054	PLEKHG3	0.54	0.0014	2.85345	0.61380783	0.732496655	-0.119
cg16405432	II	37	14	95973710		0.5	0.00432	2.36443	0.36602749	0.48473193	-0.119
cg00011861	II	37	14	103607265		0.58	0.00041	3.39184	0.23758787	0.119024593	0.119
cg24860562	II	37	15	31516481		0.54	0.0014	2.85345	0.70141475	0.820281197	-0.119
cg08145839	I	37	16	922126	LMF1	0.46	0.01197	1.92207	0.74654582	0.627941878	0.119
cg01881308	I	37	16	1389301	BAIAP3	0.5	0.00432	2.36443	0.70202145	0.58310494	0.119
cg08776660	II	37	16	1670473	CRAMP1L	0.46	0.01197	1.92207	0.65812913	0.539469182	0.119
cg06716402	II	37	16	18494340		0.54	0.0014	2.85345	0.55933911	0.67853078	-0.119
cg00355286	I	37	16	55525729	MMP2;MMP2	0.46	0.01197	1.92207	0.72974073	0.611167488	0.119
cg06575692	II	37	16	68112968	DUS2L	0.58	0.00041	3.39184	0.64218286	0.761328937	-0.119
cg00759807	I	37	16	89390789	ANKRD11	0.46	0.01197	1.92207	0.66851828	0.549615128	0.119
cg26748477	II	37	17	38516415		0.54	0.0014	2.85345	0.65141923	0.532152534	0.119
cg01248878	II	37	17	58155376	HEATR6	0.5	0.00432	2.36443	0.16078246	0.279754112	-0.119
cg19787125	II	37	17	71532174	SDK2	0.42	0.02991	1.52413	0.658544	0.539394956	0.119
cg16366686	II	37	17	75315837	SEPT9;SEPT9;SEPT9	0.54	0.0014	2.85345	0.36170628	0.481065348	-0.119
cg02836325	II	37	17	76403955	PGS1	0.58	0.00041	3.39184	0.57937969	0.698787199	-0.119
cg23628350	II	37	17	80561982	FOXK2	0.46	0.01197	1.92207	0.57123724	0.689931946	-0.119
cg23345292	II	37	18	44099184	LOXHD1;LOXHD1;L(	0.54	0.0014	2.85345	0.6881968	0.569519171	0.119
cg26988138	I	37	19	2639424	GNG7	0.42	0.02991	1.52413	0.27338393	0.392512623	-0.119
cg14402591	II	37	19	4543487	SEMA6B	0.46	0.01197	1.92207	0.48711492	0.605625663	-0.119
cg15463580	II	37	19	6113945		0.58	0.00041	3.39184	0.64866297	0.768089001	-0.119
cg21535253	I	37	19	12758416	MAN2B1	0.58	0.00041	3.39184	0.61594047	0.734970804	-0.119
cg18339718	II	37	19	12759034	MAN2B1	0.46	0.01197	1.92207	0.40785392	0.526883328	-0.119
cg17769836	II	37	19	45445437	APOC4	0.5	0.00432	2.36443	0.77146088	0.651987583	0.119
cg04401876	II	37	19	45445449	APOC4	0.67	2.34E-05	4.63072	0.78592038	0.666929346	0.119
cg16618260	II	37	19	50177057	BCL2L12;BCL2L12	0.46	0.01197	1.92207	0.77399288	0.654899671	0.119
cg01552272	I	37	20	13976096	MACROD2	0.58	0.00041	3.39184	0.09385685	0.213343587	-0.119
cg14801864	II	37	20	17540975	BFSP1	0.5	0.00432	2.36443	0.63458813	0.7533337	-0.119
cg09696044	II	37	20	55968294	RBM38;RBM38	0.54	0.0014	2.85345	0.2187174	0.099690207	0.119
cg06546183	II	37	21	44257072		0.5	0.00432	2.36443	0.66829834	0.549266642	0.119
cg18696237	II	37	22	39491975	APOBEC3H;APOBEC	0.46	0.01197	1.92207	0.70455916	0.585912688	0.119
cg24849633	II	37	22	51142900	SHANK3	0.54	0.0014	2.85345	0.49307309	0.612153479	-0.119
cg11080552	II	37	1	8556722	RERE;RERE	0.46	0.01197	1.92207	0.6540523	0.535883774	0.118

cg15527643	II	37	1	11795946	AGTRAP;AGTRAP;A	0.58	0.00041	3.39184	0.3657355	0.484081152	-0.118
cg00980060	II	37	1	14849511		0.54	0.0014	2.85345	0.61910967	0.500639094	0.118
cg24925701	II	37	1	18553942	IGSF21	0.46	0.01197	1.92207	0.68030664	0.797844329	-0.118
cg25138553	II	37	1	22223841	HSPG2	0.71	4.57E-06	5.34042	0.74160829	0.859644484	-0.118
cg13303520	II	37	1	32856704	BSDC1;BSDC1;BSDC	0.5	0.00432	2.36443	0.2027436	0.320560983	-0.118
cg06638529	II	37	1	44704073	ERI3	0.54	0.0014	2.85345	0.28581621	0.40390867	-0.118
cg27291710	II	37	1	115866441	NGF	0.46	0.01197	1.92207	0.57132208	0.453749717	0.118
cg11953775	I	37	1	207597011		0.5	0.00432	2.36443	0.8698046	0.751933962	0.118
cg16137147	I	37	1	221067896		0.46	0.01197	1.92207	0.17115215	0.28893561	-0.118
cg12136950	II	37	1	226849798	ITPKB	0.46	0.01197	1.92207	0.58871573	0.470735057	0.118
cg12280471	II	37	1	247587794	NLRP3;NLRP3;NLRP	0.58	0.00041	3.39184	0.84867747	0.730422787	0.118
cg14659606	II	37	2	20291806		0.54	0.0014	2.85345	0.78248558	0.664292281	0.118
cg04335293	II	37	2	24713157		0.58	0.00041	3.39184	0.27353614	0.391356872	-0.118
cg07338715	II	37	2	109649281		0.42	0.02991	1.52413	0.54253493	0.660595243	-0.118
cg20968678	II	37	2	216948982	TMEM169;TMEM16	0.5	0.00432	2.36443	0.68973469	0.572003129	0.118
cg13321077	I	37	2	220196755	RESP18	0.42	0.02991	1.52413	0.25205014	0.370358497	-0.118
cg23906687	II	37	2	239047029	KLHL30	0.42	0.02991	1.52413	0.47456971	0.356260383	0.118
cg07480446	II	37	2	239069576		0.58	0.00041	3.39184	0.61053616	0.728483226	-0.118
cg08276645	II	37	2	241459502	ANKMY1;ANKMY1	0.5	0.00432	2.36443	0.56939181	0.687124454	-0.118
cg18863595	I	37	3	6902845	GRM7;GRM7;GRM7	0.46	0.01197	1.92207	0.16921077	0.287566353	-0.118
cg03589820	II	37	3	11585825	ATG7;ATG7;ATG7	0.54	0.0014	2.85345	0.60504948	0.722907346	-0.118
cg02555923	II	37	3	14319984		0.54	0.0014	2.85345	0.30949436	0.427391608	-0.118
cg11931463	II	37	3	156807379		0.5	0.00432	2.36443	0.57091313	0.45328155	0.118
cg05937737	I	37	3	170303540	SLC7A14	0.58	0.00041	3.39184	0.30395395	0.421617859	-0.118
cg06085579	II	37	3	171509822	PLD1;PLD1	0.46	0.01197	1.92207	0.6459212	0.528401407	0.118
cg05027594	II	37	4	16225910	TAPT1	0.5	0.00432	2.36443	0.61999749	0.737756723	-0.118
cg02458885	II	37	4	81119249	PRDM8;PRDM8	0.5	0.00432	2.36443	0.28062634	0.398373353	-0.118
cg19431495	II	37	5	454456	EXOC3	0.58	0.00041	3.39184	0.74310386	0.624956997	0.118
cg10530883	I	37	5	3596207	IRX1;IRX1	0.46	0.01197	1.92207	0.13152586	0.249510177	-0.118
cg15422005	II	37	5	87991197		0.54	0.0014	2.85345	0.27046859	0.388685157	-0.118
cg00808170	II	37	5	140807787	PCDHGA4;PCDHGA	0.5	0.00432	2.36443	0.50438725	0.622353351	-0.118
cg15697257	II	37	5	150020212	SYNPO;SYNPO;SYNI	0.5	0.00432	2.36443	0.32749678	0.445668198	-0.118
cg25221615	II	37	5	158532962		0.46	0.01197	1.92207	0.63967086	0.758130993	-0.118

cg03228145	II	37	5	178204763	AACSL	0.62	0.0001	3.98291	0.15477143	0.272808418	-0.118
cg00582971	I	37	5	178422128	GRM6	0.54	0.0014	2.85345	0.0836711	0.201489035	-0.118
cg01905633	I	37	6	3849391	FAM50B	0.75	7.61E-07	6.11857	0.42574743	0.307376437	0.118
cg26514623	II	37	6	13295561		0.46	0.01197	1.92207	0.73253193	0.850870049	-0.118
cg12105190	II	37	6	30653407	KIAA1949;KIAA1949	0.46	0.01197	1.92207	0.30112239	0.182827507	0.118
cg00975876	II	37	6	41752769	PRICKLE4	0.5	0.00432	2.36443	0.5812614	0.699196539	-0.118
cg20732076	II	37	6	42335231	TRERF1	0.46	0.01197	1.92207	0.27471417	0.392834028	-0.118
cg15787744	II	37	6	44234175	NFKBIE	0.46	0.01197	1.92207	0.36018598	0.24221807	0.118
cg13629388	II	37	6	170687780	FAM120B	0.42	0.02991	1.52413	0.33044454	0.448602698	-0.118
cg26620655	I	37	7	1113029	C7orf50;C7orf50;C7	0.5	0.00432	2.36443	0.343585	0.461606968	-0.118
cg26724841	II	37	7	5816628	RNF216;RNF216	0.5	0.00432	2.36443	0.63761891	0.5196462	0.118
cg06682445	II	37	7	43915791	URGCP;URGCP;URG	0.46	0.01197	1.92207	0.47415389	0.592586239	-0.118
cg06807926	II	37	7	150773709	FASTK;FASTK	0.62	0.0001	3.98291	0.58539916	0.702928911	-0.118
cg26607620	I	37	8	1497078	DLGAP2	0.46	0.01197	1.92207	0.78110472	0.898655255	-0.118
cg20103825	II	37	8	1993545	MYOM2	0.42	0.02991	1.52413	0.34864829	0.230352587	0.118
cg00318347	II	37	8	25812500	EBF2	0.42	0.02991	1.52413	0.5961997	0.71387923	-0.118
cg25247520	II	37	8	128808017	MIR1204;PVT1	0.58	0.00041	3.39184	0.50295444	0.384561883	0.118
cg14708514	II	37	8	141609338	EIF2C2;EIF2C2	0.46	0.01197	1.92207	0.17537946	0.293679739	-0.118
cg03487430	II	37	9	35616244	CD72	0.42	0.02991	1.52413	0.59930818	0.481733928	0.118
cg14623306	II	37	9	84280537	TLE1	0.5	0.00432	2.36443	0.29714165	0.415027327	-0.118
cg16274762	II	37	11	1481779	BRSK2	0.46	0.01197	1.92207	0.37099298	0.489244984	-0.118
cg04959790	II	37	11	47278977	NR1H3;NR1H3;NR1	0.62	0.0001	3.98291	0.51370805	0.632114157	-0.118
cg05820396	II	37	11	111055240		0.42	0.02991	1.52413	0.64279741	0.524416697	0.118
cg10700424	I	37	11	134201952	GLB1L2;GLB1L2	0.5	0.00432	2.36443	0.03274224	0.150968957	-0.118
cg00766289	I	37	12	3311121	TSPAN9;TSPAN9	0.5	0.00432	2.36443	0.57887296	0.461367326	0.118
cg13135241	II	37	12	104765292		0.62	0.0001	3.98291	0.41748939	0.53543024	-0.118
cg01363734	II	37	12	117482953	TESC;TESC;TESC	0.5	0.00432	2.36443	0.38540883	0.503351128	-0.118
cg11050793	I	37	12	124876650	NCOR2;NCOR2	0.5	0.00432	2.36443	0.26444913	0.382498637	-0.118
cg11203293	I	37	13	25777762		0.5	0.00432	2.36443	0.62479991	0.506658452	0.118
cg01435643	I	37	13	113689776	MCF2L;MCF2L	0.46	0.01197	1.92207	0.44993405	0.56794216	-0.118
cg13061910	II	37	14	21288715		0.54	0.0014	2.85345	0.83441735	0.716682562	0.118
cg23198262	II	37	14	22978073		0.58	0.00041	3.39184	0.73420011	0.616694937	0.118
cg23373640	II	37	14	65696480		0.54	0.0014	2.85345	0.42418939	0.306172648	0.118

cg08796342	II	37	14	92334029	TC2N	0.46	0.01197	1.92207	0.71337105	0.594881909	0.118
cg23641237	II	37	14	106321870		0.54	0.0014	2.85345	0.66611682	0.548537472	0.118
cg01959287	II	37	15	83515335		0.54	0.0014	2.85345	0.78460971	0.666939869	0.118
cg27035480	II	37	16	3907755	CREBBP;CREBBP	0.5	0.00432	2.36443	0.75843022	0.876827964	-0.118
cg05000199	II	37	16	29701173	QPRT	0.67	2.34E-05	4.63072	0.55141748	0.669782946	-0.118
cg05329317	II	37	16	30126595	MAPK3;MAPK3;MA	0.5	0.00432	2.36443	0.65699586	0.539327976	0.118
cg04526858	II	37	16	66635626		0.42	0.02991	1.52413	0.50880731	0.390503258	0.118
cg09482050	I	37	16	67686832	RLTPR	0.42	0.02991	1.52413	0.59009671	0.707627674	-0.118
cg00268744	II	37	16	68281186	PLA2G15	0.58	0.00041	3.39184	0.68905604	0.80675714	-0.118
cg04170065	II	37	16	72153204	PMFBP1;PMFBP1	0.67	2.34E-05	4.63072	0.4400684	0.558479881	-0.118
cg27342781	II	37	16	84566279		0.54	0.0014	2.85345	0.47482241	0.593295316	-0.118
cg05295930	I	37	16	89180285	ACSF3;ACSF3;ACSF3	0.5	0.00432	2.36443	0.68508278	0.567153196	0.118
cg07888884	II	37	17	25886793	KSR1	0.46	0.01197	1.92207	0.55746061	0.67564448	-0.118
cg14834285	II	37	17	33897374		0.42	0.02991	1.52413	0.61524563	0.732785582	-0.118
cg14018141	II	37	17	72480644	CD300A	0.54	0.0014	2.85345	0.68822536	0.570620048	0.118
cg18767057	I	37	17	76175611	TK1	0.58	0.00041	3.39184	0.66638243	0.784816058	-0.118
cg19016694	II	37	17	80821826	TBCD	0.46	0.01197	1.92207	0.31948161	0.201287696	0.118
cg20656868	II	37	19	3177310	S1PR4	0.42	0.02991	1.52413	0.26011222	0.377906362	-0.118
cg12068366	II	37	19	4548354	SEMA6B	0.54	0.0014	2.85345	0.60413351	0.721838933	-0.118
cg02849956	II	37	19	4634827		0.67	2.34E-05	4.63072	0.21860798	0.336669985	-0.118
cg22688564	II	37	19	6271968	MLLT1	0.54	0.0014	2.85345	0.59595848	0.713670185	-0.118
cg20704774	II	37	19	15335041		0.58	0.00041	3.39184	0.39039674	0.508410965	-0.118
cg08365609	II	37	19	33726655		0.58	0.00041	3.39184	0.63799607	0.519660683	0.118
cg13918544	II	37	19	50921232	POLD1;SPIB	0.46	0.01197	1.92207	0.74005357	0.621726541	0.118
cg15838320	II	37	20	49461129	BCAS4;BCAS4;BCAS	0.54	0.0014	2.85345	0.76009216	0.642476128	0.118
cg20477147	II	37	20	57265991		0.46	0.01197	1.92207	0.3213327	0.439457408	-0.118
cg23208590	II	37	22	37915795	CARD10	0.71	4.57E-06	5.34042	0.15634788	0.274112646	-0.118
cg01652190	II	37	22	50026171	C22orf34	0.42	0.02991	1.52413	0.63292682	0.751055333	-0.118
cg07701579	II	37	1	2262232	MORN1	0.46	0.01197	1.92207	0.73340953	0.616467387	0.117
cg21826784	I	37	1	11795937	AGTRAP;AGTRAP;A	0.5	0.00432	2.36443	0.3674883	0.484765272	-0.117
cg26353598	II	37	1	14824776		0.54	0.0014	2.85345	0.71982347	0.602974091	0.117
cg24405951	I	37	1	19337477		0.42	0.02991	1.52413	0.85502948	0.738403589	0.117
cg24468934	II	37	1	26880767	MIR1976;RPS6KA1;	0.42	0.02991	1.52413	0.51574634	0.633034583	-0.117

cg24433287	II	37	1	38261057	MANEAL;MANEAL;I	0.46	0.01197	1.92207	0.26960547	0.386153177	-0.117
cg20591472	II	37	1	110008990	SYPL2	0.46	0.01197	1.92207	0.25736091	0.374789977	-0.117
cg07120889	II	37	1	150535935		0.5	0.00432	2.36443	0.21863985	0.101228105	0.117
cg24049880	II	37	1	161171211	NDUFS2;NDUFS2	0.58	0.00041	3.39184	0.2130308	0.329863955	-0.117
cg05799811	II	37	1	167487396	CD247;CD247	0.42	0.02991	1.52413	0.81095916	0.693886953	0.117
cg26359730	II	37	1	175642761	TNR	0.42	0.02991	1.52413	0.55431126	0.437761365	0.117
cg25020850	II	37	1	182642186	RGS8;RGS8	0.42	0.02991	1.52413	0.74348349	0.626632287	0.117
cg01437204	II	37	1	202130344	PTPN7;PTPN7	0.5	0.00432	2.36443	0.24912239	0.36596482	-0.117
cg01910727	II	37	1	226842455	ITPKB	0.46	0.01197	1.92207	0.61043233	0.72721881	-0.117
cg14093127	II	37	2	8714417		0.46	0.01197	1.92207	0.7286211	0.611365578	0.117
cg00484122	II	37	2	20579065		0.54	0.0014	2.85345	0.58979197	0.70716543	-0.117
cg01088579	II	37	2	85660497	SH2D6	0.42	0.02991	1.52413	0.64517046	0.762355736	-0.117
cg06442723	II	37	2	97484665	CNNM3;CNNM3	0.42	0.02991	1.52413	0.38252209	0.499185717	-0.117
cg26154999	II	37	2	225266346	FAM124B;FAM124I	0.42	0.02991	1.52413	0.15984418	0.276595275	-0.117
cg00153306	II	37	3	46741492	TMIE	0.67	2.34E-05	4.63072	0.3212075	0.437964994	-0.117
cg21926782	II	37	3	49459909	AMT;AMT;AMT;NIC	0.5	0.00432	2.36443	0.35903194	0.475615454	-0.117
cg26862175	II	37	3	52528955	STAB1	0.54	0.0014	2.85345	0.41905909	0.535626244	-0.117
cg18996141	II	37	3	71359873	FOXP1;FOXP1	0.62	0.0001	3.98291	0.72028527	0.837132089	-0.117
cg10755723	II	37	3	192576964	C3orf59	0.46	0.01197	1.92207	0.65108314	0.533849695	0.117
cg10631854	II	37	3	197383170		0.42	0.02991	1.52413	0.72079014	0.603566704	0.117
cg10413136	I	37	4	8207119	SH3TC1	0.5	0.00432	2.36443	0.45009397	0.567250708	-0.117
cg00753676	II	37	4	38662991	FLJ13197	0.58	0.00041	3.39184	0.66074375	0.77739381	-0.117
cg25940248	I	37	4	39448464	KLB	0.54	0.0014	2.85345	0.59163779	0.474690188	0.117
cg04085076	II	37	4	57547579	HOPX;HOPX;HOPX;	0.42	0.02991	1.52413	0.46138788	0.344189907	0.117
cg11293275	II	37	5	131543977	P4HA2;P4HA2;P4H/	0.5	0.00432	2.36443	0.27158823	0.388157898	-0.117
cg25140347	II	37	5	149303401	PDE6A	0.42	0.02991	1.52413	0.823529	0.706858349	0.117
cg15892650	II	37	5	150524635	ANXA6;ANXA6	0.5	0.00432	2.36443	0.76618484	0.648972027	0.117
cg12177334	II	37	5	176937350	DOK3;DOK3;DOK3;	0.54	0.0014	2.85345	0.32428127	0.207728125	0.117
cg18000306	II	37	6	288505		0.46	0.01197	1.92207	0.65829986	0.775565927	-0.117
cg26336265	II	37	6	25042955		0.46	0.01197	1.92207	0.43931543	0.321893111	0.117
cg01505556	II	37	6	30174125	TRIM26	0.46	0.01197	1.92207	0.5623608	0.445743384	0.117
cg03280235	II	37	6	32158953	PBX2;GPSM3	0.46	0.01197	1.92207	0.66350109	0.546577228	0.117
cg21974506	I	37	6	33141291	COL11A2;COL11A2;	0.42	0.02991	1.52413	0.66705481	0.549760324	0.117

cg11071155	II	37	6	157012049		0.5	0.00432	2.36443	0.79807905	0.681100455	0.117
cg18758976	II	37	6	158430311	SYNJ2	0.62	0.0001	3.98291	0.39771113	0.515164849	-0.117
cg07567256	II	37	6	163757048		0.71	4.57E-06	5.34042	0.22216752	0.339564424	-0.117
cg00088688	II	37	6	167560504		0.46	0.01197	1.92207	0.62683789	0.743358053	-0.117
cg05835726	II	37	7	27183861	HOXA5	0.46	0.01197	1.92207	0.69539446	0.812582907	-0.117
cg26283713	II	37	7	128375140		0.62	0.0001	3.98291	0.71068714	0.827318097	-0.117
cg02049210	II	37	8	48647583	KIAA0146	0.5	0.00432	2.36443	0.142116	0.258736425	-0.117
cg11749792	II	37	8	124730091	ANXA13;ANXA13	0.5	0.00432	2.36443	0.72967041	0.846247207	-0.117
cg22581352	II	37	8	142219329		0.54	0.0014	2.85345	0.37654539	0.493709841	-0.117
cg03924115	II	37	9	133768966	QRFP	0.5	0.00432	2.36443	0.55933808	0.676087634	-0.117
cg09098522	II	37	10	31435673		0.46	0.01197	1.92207	0.29431868	0.411483571	-0.117
cg08889480	II	37	10	47746789	ANXA8L2	0.58	0.00041	3.39184	0.74738227	0.864380487	-0.117
cg00495658	II	37	10	48417904	GDF2	0.46	0.01197	1.92207	0.69745311	0.580042352	0.117
cg17535314	II	37	10	48435475	GDF10	0.5	0.00432	2.36443	0.74627221	0.629512144	0.117
cg17664182	II	37	10	99696260	CRTAC1	0.42	0.02991	1.52413	0.77647055	0.659605199	0.117
cg09405083	II	37	10	104536121	C10orf26;C10orf26	0.67	2.34E-05	4.63072	0.19556823	0.312504446	-0.117
cg18143317	II	37	11	2037124		0.62	0.0001	3.98291	0.54714884	0.430174163	0.117
cg08376310	I	37	11	2858621	KCNQ1;KCNQ1	0.54	0.0014	2.85345	0.82366539	0.940545925	-0.117
cg23190089	II	37	11	2920209	SLC22A18AS;SLC22	0.5	0.00432	2.36443	0.17997295	0.297314162	-0.117
cg11822932	II	37	11	33913716	LMO2;LMO2	0.42	0.02991	1.52413	0.25039392	0.367067455	-0.117
cg06429555	II	37	11	72542625		0.5	0.00432	2.36443	0.50050099	0.617839998	-0.117
cg05705813	II	37	11	86383809	ME3;ME3;ME3	0.54	0.0014	2.85345	0.36810468	0.48478088	-0.117
cg08427067	II	37	11	95015466		0.54	0.0014	2.85345	0.75136659	0.867888462	-0.117
cg19308663	II	37	11	118741387		0.54	0.0014	2.85345	0.24096428	0.124087524	0.117
cg22036538	II	37	12	6554051	CD27;CD27;LOC678	0.54	0.0014	2.85345	0.26348296	0.145989314	0.117
cg07684519	II	37	12	29303184		0.5	0.00432	2.36443	0.14238378	0.25972083	-0.117
cg16242629	I	37	12	30354246		0.42	0.02991	1.52413	0.26812416	0.384729147	-0.117
cg23815853	II	37	12	48147034	RAPGEF3;RAPGEF3;	0.42	0.02991	1.52413	0.69350171	0.810702754	-0.117
cg24821554	II	37	13	51639953	GUCY1B2	0.46	0.01197	1.92207	0.37900047	0.496433079	-0.117
cg07258507	II	37	14	23842125	IL25;IL25;IL25;IL25	0.54	0.0014	2.85345	0.56083914	0.443956368	0.117
cg10199763	II	37	14	24458299	DHRS4L2	0.46	0.01197	1.92207	0.14198917	0.259082099	-0.117
cg13704629	II	37	14	73396469	DCAF4;DCAF4;DCAI	0.62	0.0001	3.98291	0.51644659	0.633156038	-0.117
cg04913803	II	37	14	77510549		0.54	0.0014	2.85345	0.6720405	0.789035061	-0.117

cg18091385	II	37	14	99502466		0.54	0.0014	2.85345	0.34651501	0.463800547	-0.117
cg10142436	II	37	15	42209178	EHD4	0.58	0.00041	3.39184	0.17078412	0.287345727	-0.117
cg20507228	II	37	15	91460071	MAN2A2	0.42	0.02991	1.52413	0.51848744	0.635816805	-0.117
cg00658411	II	37	16	4467558	CORO7	0.54	0.0014	2.85345	0.60062004	0.717862321	-0.117
cg16660091	II	37	16	56995856	CETP;CETP	0.5	0.00432	2.36443	0.52197011	0.638879907	-0.117
cg16594273	I	37	16	58131312		0.67	2.34E-05	4.63072	0.35124363	0.234536349	0.117
cg22381196	II	37	16	72041376	DHODH	0.46	0.01197	1.92207	0.82450626	0.94124905	-0.117
cg04016660	II	37	16	85075232	KIAA0513	0.42	0.02991	1.52413	0.72316849	0.840407334	-0.117
cg08472633	II	37	16	88907370	GALNS	0.42	0.02991	1.52413	0.62270936	0.740205102	-0.117
cg02431972	II	37	16	89023488	CBFA2T3	0.5	0.00432	2.36443	0.51672977	0.633654752	-0.117
cg06082883	II	37	16	89686744	DPEP1;DPEP1	0.42	0.02991	1.52413	0.67772667	0.560879685	0.117
cg02635449	II	37	17	109926	RPH3AL	0.46	0.01197	1.92207	0.49654192	0.379955809	0.117
cg02352716	II	37	17	1104805		0.46	0.01197	1.92207	0.79656547	0.67935688	0.117
cg14330293	I	37	17	1374051	MYO1C;MYO1C;MY	0.67	2.34E-05	4.63072	0.59969647	0.716987338	-0.117
cg06255609	II	37	17	3493563	TRPV1;TRPV1;TRPV	0.46	0.01197	1.92207	0.68403704	0.567145211	0.117
cg21509889	II	37	17	6683446	FBXO39	0.5	0.00432	2.36443	0.70988766	0.5926375	0.117
cg19536664	II	37	17	6899085	ALOX12	0.46	0.01197	1.92207	0.67219598	0.789626966	-0.117
cg14072027	II	37	17	15301904		0.5	0.00432	2.36443	0.18415355	0.301007661	-0.117
cg18748374	II	37	17	27482619	MYO18A;MYO18A	0.42	0.02991	1.52413	0.32156189	0.438553194	-0.117
cg20693334	II	37	17	46654330	HOXB4	0.54	0.0014	2.85345	0.2194881	0.336264933	-0.117
cg20614854	II	37	17	71331689	SDK2	0.46	0.01197	1.92207	0.65503839	0.537596118	0.117
cg26263675	II	37	17	72743191	RAB37;RAB37;RAB3	0.54	0.0014	2.85345	0.20972093	0.326677072	-0.117
cg20338276	I	37	17	73246585	GGA3;GGA3	0.5	0.00432	2.36443	0.72256143	0.605822187	0.117
cg22652378	II	37	17	78533842	RPTOR;RPTOR	0.42	0.02991	1.52413	0.7261479	0.843102384	-0.117
cg16584393	II	37	18	14747888	ANKRD30B	0.54	0.0014	2.85345	0.32166587	0.438851789	-0.117
cg16151261	I	37	18	75691297		0.54	0.0014	2.85345	0.82686232	0.710148433	0.117
cg03943674	II	37	18	77643329	KCNG2	0.5	0.00432	2.36443	0.71740664	0.834251219	-0.117
cg18465133	I	37	19	2252282	JSRP1	0.58	0.00041	3.39184	0.13824302	0.255099362	-0.117
cg00513288	II	37	19	2358394		0.58	0.00041	3.39184	0.50069628	0.383984508	0.117
cg10340117	II	37	19	3984058	EEF2;SNORD37	0.42	0.02991	1.52413	0.75653203	0.639642017	0.117
cg00479463	I	37	19	33726786		0.58	0.00041	3.39184	0.57688961	0.460326078	0.117
cg02385820	II	37	19	39359726	RINL	0.54	0.0014	2.85345	0.58848052	0.471409531	0.117
cg12362077	II	37	20	2081907	STK35	0.42	0.02991	1.52413	0.58841781	0.705193967	-0.117

cg24484138	II	37	20	31070190	C20orf112	0.54	0.0014	2.85345	0.23464963	0.351558939	-0.117
cg09727148	II	37	21	43560719	UMODL1;UMODL1	0.42	0.02991	1.52413	0.5980641	0.481361617	0.117
cg26722225	II	37	21	47710409	C21orf57;C21orf57	0.5	0.00432	2.36443	0.84218647	0.724914748	0.117
cg19343611	I	37	22	28010528		0.5	0.00432	2.36443	0.22192191	0.339181215	-0.117
cg17367078	II	37	22	35389501		0.58	0.00041	3.39184	0.67809783	0.560944631	0.117
cg02541592	II	37	22	45608686	C22orf9;C22orf9	0.62	0.0001	3.98291	0.79289173	0.909435543	-0.117
cg20366239	II	37	22	50841775	SAPS2	0.46	0.01197	1.92207	0.30124427	0.418233245	-0.117
cg09275693	II	37	1	3819354	LOC100133612	0.62	0.0001	3.98291	0.81021779	0.694101585	0.116
cg16023434	II	37	1	11395635		0.42	0.02991	1.52413	0.68641283	0.569994905	0.116
cg18229396	II	37	1	13845638		0.54	0.0014	2.85345	0.70495095	0.589326195	0.116
cg24875017	I	37	1	17026258	ESPNP	0.46	0.01197	1.92207	0.40956158	0.293649941	0.116
cg01519464	II	37	1	24861818	RCAN3	0.54	0.0014	2.85345	0.65873532	0.542451832	0.116
cg24691891	II	37	1	33829934	PHC2	0.42	0.02991	1.52413	0.75027609	0.634320605	0.116
cg17139085	II	37	1	36787356		0.5	0.00432	2.36443	0.46353673	0.579774642	-0.116
cg21785067	II	37	1	54587182		0.54	0.0014	2.85345	0.20983889	0.325546799	-0.116
cg21536783	II	37	1	59041407	TACSTD2;TACSTD2	0.42	0.02991	1.52413	0.68375291	0.800173906	-0.116
cg26422458	I	37	1	79472452	ELTD1;ELTD1	0.46	0.01197	1.92207	0.19930977	0.315761268	-0.116
cg13080606	II	37	1	110999462	PROK1	0.62	0.0001	3.98291	0.80419386	0.688518201	0.116
cg22706106	II	37	1	153607701	C1orf77;S100A13	0.5	0.00432	2.36443	0.59496408	0.479310657	0.116
cg25407979	I	37	1	204256846	PLEKHA6	0.46	0.01197	1.92207	0.1145073	0.23071001	-0.116
cg15152595	II	37	1	204972818	NFASC;NFASC;NFAS	0.42	0.02991	1.52413	0.81283897	0.696987276	0.116
cg24401487	II	37	1	230416880	GALNT2	0.42	0.02991	1.52413	0.30188046	0.418186901	-0.116
cg11166453	I	37	1	247681781		0.42	0.02991	1.52413	0.649795	0.765390635	-0.116
cg08621773	II	37	2	25438675		0.42	0.02991	1.52413	0.70939165	0.593015743	0.116
cg13156207	II	37	2	28873060		0.42	0.02991	1.52413	0.78678554	0.671162011	0.116
cg19445690	II	37	2	74210890		0.5	0.00432	2.36443	0.24475918	0.360336208	-0.116
cg20810222	II	37	2	109947649	SH3RF3	0.46	0.01197	1.92207	0.1774263	0.293749358	-0.116
cg23499749	I	37	2	113345365	CHCHD5	0.5	0.00432	2.36443	0.75327301	0.869524337	-0.116
cg11612905	II	37	2	234359654	DGKD;DGKD	0.46	0.01197	1.92207	0.72854983	0.844201875	-0.116
cg25921544	II	37	2	240040290	HDAC4	0.5	0.00432	2.36443	0.69653359	0.812453275	-0.116
cg26092233	I	37	2	240697636		0.46	0.01197	1.92207	0.84520848	0.729197787	0.116
cg06476685	II	37	2	241459638	ANKMY1;ANKMY1	0.5	0.00432	2.36443	0.39717185	0.513507613	-0.116
cg26121234	II	37	3	13899618	WNT7A	0.46	0.01197	1.92207	0.66119333	0.544948156	0.116

cg15681239	II	37	3	38080203	DLEC1;DLEC1	0.5	0.00432	2.36443	0.27380195	0.389426885	-0.116
cg20203592	II	37	3	111591382	PHLDB2;PHLDB2;PH	0.46	0.01197	1.92207	0.71816815	0.601681805	0.116
cg25357706	II	37	3	123123576	ADCY5	0.5	0.00432	2.36443	0.69803086	0.582023752	0.116
cg19497451	II	37	3	134331952	KY	0.46	0.01197	1.92207	0.70367378	0.587796325	0.116
cg01610632	II	37	3	147127579	ZIC1;ZIC1	0.67	2.34E-05	4.63072	0.17120829	0.287275973	-0.116
cg27313776	II	37	4	146809012	ZNF827	0.5	0.00432	2.36443	0.77709019	0.660872687	0.116
cg08347626	II	37	5	1850140		0.54	0.0014	2.85345	0.36593877	0.482310691	-0.116
cg16230626	I	37	5	124298608		0.5	0.00432	2.36443	0.75966167	0.876107826	-0.116
cg18424208	I	37	5	149546337	CDX1	0.42	0.02991	1.52413	0.79802327	0.682438804	0.116
cg19228647	II	37	6	6901002		0.5	0.00432	2.36443	0.18096514	0.29647743	-0.116
cg13534892	I	37	6	30704998	FLOT1	0.5	0.00432	2.36443	0.70218269	0.586329961	0.116
cg26695758	I	37	6	32063607	TNXB	0.42	0.02991	1.52413	0.70803516	0.824393638	-0.116
cg20765522	II	37	6	32154715	PBX2	0.5	0.00432	2.36443	0.34216223	0.458365356	-0.116
cg16602097	II	37	6	32182013	NOTCH4	0.42	0.02991	1.52413	0.23078325	0.346721553	-0.116
cg06535156	II	37	6	155538055	TIAM2;TIAM2	0.42	0.02991	1.52413	0.50789519	0.624139742	-0.116
cg12002139	II	37	6	158478872	SYNJ2	0.42	0.02991	1.52413	0.60881768	0.724368335	-0.116
cg27186851	II	37	7	2566750	LFNG;LFNG;LFNG;LI	0.5	0.00432	2.36443	0.66633833	0.550678402	0.116
cg12400790	II	37	7	2635181	IQCE;IQCE	0.42	0.02991	1.52413	0.67643342	0.560124363	0.116
cg02916332	II	37	7	27183591	HOXA5	0.46	0.01197	1.92207	0.55944258	0.675768841	-0.116
cg14073571	II	37	7	158823179	VIPR2	0.62	0.0001	3.98291	0.24756056	0.363257688	-0.116
cg13876315	II	37	9	35650526	SIT1	0.62	0.0001	3.98291	0.58378432	0.467364866	0.116
cg01422243	II	37	10	22725309		0.54	0.0014	2.85345	0.58306659	0.467324075	0.116
cg20286882	II	37	10	33432514		0.42	0.02991	1.52413	0.70317078	0.587337859	0.116
cg07128021	II	37	10	80824237	LOC283050;LOC283	0.67	2.34E-05	4.63072	0.51450452	0.398026905	0.116
cg14374521	I	37	10	124638874	FAM24B;LOC39981	0.42	0.02991	1.52413	0.21622827	0.099818075	0.116
cg16872613	II	37	10	131260015		0.5	0.00432	2.36443	0.74164409	0.625679629	0.116
cg05457684	II	37	11	2919808	SLC22A18AS;SLC22	0.46	0.01197	1.92207	0.37561082	0.492106439	-0.116
cg18988498	I	37	11	31824262	PAX6;PAX6;PAX6	0.42	0.02991	1.52413	0.1681106	0.283646617	-0.116
cg06059409	II	37	11	73036247	ARHGEF17	0.5	0.00432	2.36443	0.61406056	0.497800695	0.116
cg06180869	II	37	11	76446769		0.5	0.00432	2.36443	0.68123773	0.565180935	0.116
cg12830752	II	37	11	79108069	ODZ4	0.5	0.00432	2.36443	0.57554272	0.459484093	0.116
cg19624354	II	37	11	113113890	NCAM1;NCAM1;NC	0.5	0.00432	2.36443	0.36403101	0.480033895	-0.116
cg25577489	II	37	11	113950061	ZBTB16;ZBTB16	0.54	0.0014	2.85345	0.61350882	0.729572782	-0.116

cg03055065	II	37	12	4671310		0.46	0.01197	1.92207	0.14413622	0.260379031	-0.116
cg07052231	II	37	12	7363540	PEX5;PEX5;PEX5;PE	0.46	0.01197	1.92207	0.74694145	0.862646801	-0.116
cg15244101	II	37	12	49627624		0.54	0.0014	2.85345	0.17126643	0.287449865	-0.116
cg08888956	II	37	12	86267839	NTS	0.46	0.01197	1.92207	0.49461289	0.610601611	-0.116
cg10996589	II	37	12	125017134	NCOR2;NCOR2	0.54	0.0014	2.85345	0.65219969	0.768437752	-0.116
cg17180284	II	37	13	31783983	B3GALTL	0.58	0.00041	3.39184	0.43768288	0.553531625	-0.116
cg05168229	II	37	13	45390049		0.42	0.02991	1.52413	0.34132541	0.457571981	-0.116
cg00802237	II	37	14	94858066	SERPINA1;SERPINA	0.54	0.0014	2.85345	0.68461456	0.800259179	-0.116
cg21141030	II	37	15	65576504	PARP16	0.58	0.00041	3.39184	0.80793903	0.923462605	-0.116
cg08765811	I	37	15	77956125		0.54	0.0014	2.85345	0.79761871	0.682012645	0.116
cg09073799	II	37	15	82336202	MEX3B	0.46	0.01197	1.92207	0.63446084	0.749993604	-0.116
cg06876892	I	37	16	833231	MSLNL	0.42	0.02991	1.52413	0.77414594	0.657982486	0.116
cg06803853	I	37	16	2234031	CASKIN1	0.46	0.01197	1.92207	0.86177677	0.74573218	0.116
cg16548911	II	37	16	50347766	ADCY7	0.54	0.0014	2.85345	0.28018564	0.395805346	-0.116
cg02670686	II	37	16	57125674	CPNE2	0.5	0.00432	2.36443	0.1934088	0.309583624	-0.116
cg06204040	I	37	16	89037481	CBFA2T3	0.5	0.00432	2.36443	0.94795003	0.832324645	0.116
cg14375499	II	37	17	6899207	ALOX12	0.58	0.00041	3.39184	0.42575844	0.541609346	-0.116
cg19876689	II	37	17	44799518	NSF	0.75	7.61E-07	6.11857	0.59079458	0.707053761	-0.116
cg13698613	II	37	17	72518840	CD300LB	0.46	0.01197	1.92207	0.63524896	0.518955228	0.116
cg12061649	II	37	17	72563547		0.42	0.02991	1.52413	0.22258087	0.338257748	-0.116
cg03641032	II	37	17	78911768	RPTOR;RPTOR	0.75	7.61E-07	6.11857	0.70286705	0.819260201	-0.116
cg26739149	I	37	17	78997350		0.46	0.01197	1.92207	0.71509518	0.831302077	-0.116
cg21695089	II	37	17	79273418		0.54	0.0014	2.85345	0.29761127	0.41326797	-0.116
cg25877299	II	37	17	80273290	CD7	0.71	4.57E-06	5.34042	0.48820578	0.603713672	-0.116
cg06784563	I	37	18	77284509	NFATC1;NFATC1;NF	0.62	0.0001	3.98291	0.50104989	0.385534258	0.116
cg27374247	II	37	18	77555631		0.54	0.0014	2.85345	0.64387007	0.759915218	-0.116
cg26615224	I	37	19	1621124	TCF3;TCF3	0.42	0.02991	1.52413	0.81754069	0.701643446	0.116
cg22603569	II	37	19	3388047	NFIC;NFIC	0.62	0.0001	3.98291	0.59908291	0.715534655	-0.116
cg11904429	II	37	19	6592554	CD70	0.46	0.01197	1.92207	0.77778657	0.662159921	0.116
cg03649060	II	37	19	11665451	ELOF1	0.46	0.01197	1.92207	0.63381424	0.517350133	0.116
cg00384707	II	37	19	51128576	SYT3;SYT3;SYT3	0.5	0.00432	2.36443	0.71968093	0.604067073	0.116
cg15842722	II	37	20	23499644	CSTT	0.46	0.01197	1.92207	0.71811833	0.602081899	0.116
cg13127741	II	37	20	31331821	COMMD7;COMMD	0.58	0.00041	3.39184	0.48329137	0.367369101	0.116

cg06604199	II	37	20	31331973	COMMD7;COMMD	0.46	0.01197	1.92207	0.52772713	0.411984236	0.116
cg17571207	II	37	22	33257641	SYN3;SYN3;TIMP3;S	0.62	0.0001	3.98291	0.71295571	0.828736728	-0.116
cg26785823	II	37	1	2482001	LOC115110	0.46	0.01197	1.92207	0.46545025	0.350383835	0.115
cg12660813	II	37	1	3192343	PRDM16;PRDM16	0.42	0.02991	1.52413	0.65325887	0.538659808	0.115
cg22515654	II	37	1	10590672	PEX14	0.42	0.02991	1.52413	0.44421377	0.559481234	-0.115
cg12900314	II	37	1	21755315		0.42	0.02991	1.52413	0.59927374	0.713967767	-0.115
cg10965178	I	37	1	43766752	TIE1	0.5	0.00432	2.36443	0.66846352	0.783136241	-0.115
cg18235734	I	37	1	91301731		0.46	0.01197	1.92207	0.05721173	0.172310233	-0.115
cg11950860	II	37	1	152730231	KPRP	0.62	0.0001	3.98291	0.69100287	0.576023122	0.115
cg13423770	II	37	1	156338775	RHBG;RHBG	0.71	4.57E-06	5.34042	0.45168543	0.567043113	-0.115
cg25259754	II	37	1	157670220	FCRL3	0.5	0.00432	2.36443	0.71125851	0.596510086	0.115
cg13109911	II	37	1	160178210	PEA15	0.5	0.00432	2.36443	0.53775625	0.653034157	-0.115
cg06837040	II	37	1	180530353		0.5	0.00432	2.36443	0.69441429	0.579227713	0.115
cg19867250	II	37	1	225648715		0.46	0.01197	1.92207	0.79656967	0.681213802	0.115
cg10836509	II	37	1	226299603		0.46	0.01197	1.92207	0.84263789	0.72809506	0.115
cg25221919	II	37	1	234845770		0.54	0.0014	2.85345	0.63939596	0.754536671	-0.115
cg25432792	II	37	2	23546167		0.54	0.0014	2.85345	0.79062665	0.675675814	0.115
cg21368063	II	37	2	27338538	CGREF1;CGREF1;CG	0.46	0.01197	1.92207	0.7134757	0.598552307	0.115
cg22363368	II	37	2	59126860		0.42	0.02991	1.52413	0.77563034	0.661100341	0.115
cg10536276	II	37	2	113956344	PSD4;LOC440839	0.58	0.00041	3.39184	0.40848491	0.523009874	-0.115
cg25422880	II	37	2	135218333	TMEM163	0.46	0.01197	1.92207	0.69082642	0.575886137	0.115
cg22245446	II	37	2	218316451	DIRC3	0.62	0.0001	3.98291	0.80026131	0.685452991	0.115
cg00554192	I	37	2	241612715		0.46	0.01197	1.92207	0.63640152	0.751755477	-0.115
cg00697440	II	37	3	121795768	CD86;CD86	0.42	0.02991	1.52413	0.79564193	0.680292537	0.115
cg04509882	II	37	3	184038317	EIF4G1;EIF4G1;EIF4	0.5	0.00432	2.36443	0.67547982	0.790045444	-0.115
cg03725404	II	37	4	9998017	SLC2A9;SLC2A9	0.58	0.00041	3.39184	0.55143242	0.666825398	-0.115
cg06805348	II	37	4	37245195	KIAA1239	0.46	0.01197	1.92207	0.46310648	0.578154	-0.115
cg07327178	I	37	5	8457729		0.5	0.00432	2.36443	0.44055223	0.325169592	0.115
cg12516954	II	37	5	134714262	H2AFY;H2AFY;H2AF	0.5	0.00432	2.36443	0.30013878	0.414920283	-0.115
cg16162590	II	37	5	159360802	ADRA1B	0.46	0.01197	1.92207	0.6650303	0.549683286	0.115
cg06149159	II	37	6	7975374		0.42	0.02991	1.52413	0.15815103	0.272935832	-0.115
cg04274288	II	37	6	27648004		0.62	0.0001	3.98291	0.1031292	0.218188018	-0.115
cg05952498	II	37	6	31545257	TNF	0.42	0.02991	1.52413	0.79085477	0.675998494	0.115

cg00533183	II	37	6	32810742	PSMB8;PSMB8	0.58	0.00041	3.39184	0.66143572	0.546633289	0.115
cg14132167	II	37	6	90661052	BACH2;BACH2	0.58	0.00041	3.39184	0.70684577	0.821843836	-0.115
cg26618703	II	37	7	922962	C7orf20	0.42	0.02991	1.52413	0.51913532	0.634060833	-0.115
cg14983777	I	37	7	1052392	C7orf50;C7orf50;C7	0.62	0.0001	3.98291	0.75570536	0.870293313	-0.115
cg18297960	II	37	7	1142765	C7orf50;C7orf50;C7	0.5	0.00432	2.36443	0.71412031	0.59944337	0.115
cg21329649	II	37	7	1901840	MAD1L1;MAD1L1;M	0.46	0.01197	1.92207	0.62797041	0.512662413	0.115
cg27151303	II	37	7	27184821		0.5	0.00432	2.36443	0.51142368	0.626891236	-0.115
cg02538169	II	37	7	100287481	GIGYF1	0.5	0.00432	2.36443	0.77164812	0.656657503	0.115
cg12469001	II	37	7	129650050		0.46	0.01197	1.92207	0.57332184	0.458498136	0.115
cg18825427	II	37	7	155079356		0.62	0.0001	3.98291	0.56758521	0.682599009	-0.115
cg09027357	II	37	8	256911		0.46	0.01197	1.92207	0.60226248	0.716835584	-0.115
cg10395101	II	37	8	6420858	ANGPT2;ANGPT2;A	0.54	0.0014	2.85345	0.59519218	0.710230318	-0.115
cg12097222	I	37	8	16885000	EFHA2	0.42	0.02991	1.52413	0.07943768	0.194256474	-0.115
cg05313910	II	37	8	98361938		0.46	0.01197	1.92207	0.78792403	0.672683449	0.115
cg13837857	II	37	9	38672315		0.58	0.00041	3.39184	0.62550299	0.740019959	-0.115
cg14085060	I	37	9	107827215		0.54	0.0014	2.85345	0.17634035	0.291730949	-0.115
cg14236758	II	37	9	137252129	RXRA	0.54	0.0014	2.85345	0.44007891	0.55495721	-0.115
cg14507845	II	37	9	137418069		0.46	0.01197	1.92207	0.5943088	0.478934544	0.115
cg23865980	II	37	10	80904376	ZMIZ1	0.58	0.00041	3.39184	0.19739954	0.312148388	-0.115
cg24249916	II	37	10	95326178	GPR120	0.5	0.00432	2.36443	0.59884779	0.713900774	-0.115
cg07402396	II	37	10	134360977	INPP5A	0.5	0.00432	2.36443	0.71676223	0.601804498	0.115
cg10351829	II	37	10	134661550		0.58	0.00041	3.39184	0.35046276	0.235237464	0.115
cg03695260	II	37	10	134945452		0.42	0.02991	1.52413	0.6521231	0.536757809	0.115
cg14522803	II	37	11	818834	PNPLA2	0.46	0.01197	1.92207	0.52240564	0.636914613	-0.115
cg06964027	II	37	11	1073496	MUC2	0.62	0.0001	3.98291	0.2409162	0.355620868	-0.115
cg23500396	II	37	11	44896984	TSPAN18	0.5	0.00432	2.36443	0.76589186	0.881293895	-0.115
cg25324164	II	37	11	61598330	FADS2	0.58	0.00041	3.39184	0.19607744	0.311320202	-0.115
cg13924996	I	37	11	67053829	ADRBK1	0.42	0.02991	1.52413	0.48292381	0.598341121	-0.115
cg05229803	II	37	11	94278407	FUT4	0.62	0.0001	3.98291	0.39189526	0.506632102	-0.115
cg07534554	II	37	11	111250338	POU2AF1	0.5	0.00432	2.36443	0.21003269	0.095290428	0.115
cg19434937	II	37	12	7104184	LPCAT3	0.54	0.0014	2.85345	0.70153476	0.816145316	-0.115
cg05126095	II	37	12	50351980	AQP2	0.58	0.00041	3.39184	0.65114861	0.76643111	-0.115
cg00294538	I	37	13	114062109		0.54	0.0014	2.85345	0.73529544	0.620218384	0.115

cg05799596	II	37	13	114909333		0.62	0.0001	3.98291	0.63657365	0.52140021	0.115
cg23248887	I	37	14	38679643	SSTR1	0.46	0.01197	1.92207	0.21356354	0.328113009	-0.115
cg16004738	I	37	14	105155327	INF2;INF2;INF2	0.46	0.01197	1.92207	0.54969128	0.664306243	-0.115
cg24736933	II	37	15	40633294	C15orf52	0.42	0.02991	1.52413	0.25954058	0.374194873	-0.115
cg05897699	II	37	15	75146514	SCAMP2	0.54	0.0014	2.85345	0.70104491	0.58643324	0.115
cg09939948	II	37	15	90548043	ZNF710	0.5	0.00432	2.36443	0.21200356	0.326944976	-0.115
cg07811054	II	37	15	97319911		0.58	0.00041	3.39184	0.85465775	0.739340296	0.115
cg03217729	II	37	16	11222890	CLEC16A	0.46	0.01197	1.92207	0.29548114	0.410524499	-0.115
cg06534313	II	37	16	16405520		0.58	0.00041	3.39184	0.47265881	0.587506115	-0.115
cg27589742	II	37	16	85296503		0.42	0.02991	1.52413	0.7639007	0.878973594	-0.115
cg09642825	I	37	16	87416230	FBXO31;FBXO31	0.54	0.0014	2.85345	0.41340412	0.298649899	0.115
cg16553026	II	37	16	88020079	BANP;BANP	0.58	0.00041	3.39184	0.70361384	0.818308939	-0.115
cg06959205	I	37	16	88991917	CBFA2T3;CBFA2T3	0.42	0.02991	1.52413	0.42074171	0.306048475	0.115
cg16024891	I	37	17	2839082	RAP1GAP2;RAP1GA	0.46	0.01197	1.92207	0.18984423	0.304544585	-0.115
cg25921609	I	37	17	8379225	MYH10	0.54	0.0014	2.85345	0.68098527	0.795867299	-0.115
cg00192882	II	37	17	19291120	MFAP4	0.54	0.0014	2.85345	0.55608813	0.671121618	-0.115
cg08698159	II	37	17	27294743	SEZ6;SEZ6	0.42	0.02991	1.52413	0.15771362	0.272853077	-0.115
cg12882572	II	37	17	35851204	DUSP14	0.46	0.01197	1.92207	0.30450355	0.419545761	-0.115
cg04272820	II	37	17	38228538	THRA;THRA	0.46	0.01197	1.92207	0.46561327	0.580276552	-0.115
cg16276850	II	37	17	38498914	RARA;RARA;RARA;F	0.5	0.00432	2.36443	0.23643673	0.350956432	-0.115
cg23355492	II	37	17	45933593	SP6	0.46	0.01197	1.92207	0.64536943	0.760022733	-0.115
cg26841048	II	37	17	46622454	HOXB2	0.5	0.00432	2.36443	0.49565205	0.610678132	-0.115
cg08832695	II	37	17	46676375	LOC404266;LOC404	0.62	0.0001	3.98291	0.48951771	0.604049545	-0.115
cg06407137	II	37	17	72527515	CD300LB	0.5	0.00432	2.36443	0.74102602	0.855905721	-0.115
cg26963090	II	37	17	76887989	TIMP2	0.46	0.01197	1.92207	0.48782463	0.603081873	-0.115
cg02878831	II	37	17	78847752	RPTOR;RPTOR	0.5	0.00432	2.36443	0.73513188	0.850114927	-0.115
cg21942893	I	37	19	835499		0.5	0.00432	2.36443	0.82261246	0.937542817	-0.115
cg22004069	II	37	19	5585648		0.62	0.0001	3.98291	0.41314014	0.528084179	-0.115
cg02528768	II	37	19	18547358	ISYNA1;ISYNA1;ISYI	0.58	0.00041	3.39184	0.46365293	0.579034075	-0.115
cg05336188	II	37	19	18959506	UPF1	0.46	0.01197	1.92207	0.32050612	0.435145764	-0.115
cg05209306	II	37	19	39899376	ZFP36	0.5	0.00432	2.36443	0.52861335	0.413798437	0.115
cg06055845	II	37	19	40788787	MIR641;AKT2	0.54	0.0014	2.85345	0.74675619	0.631834041	0.115
cg23058911	II	37	19	47812780	C5AR1	0.5	0.00432	2.36443	0.51407533	0.629443442	-0.115

cg08861091	II	37	19	51891339	LIM2;LIM2	0.42	0.02991	1.52413	0.73825548	0.623554942	0.115
cg23953052	II	37	19	55586493	EPS8L1	0.46	0.01197	1.92207	0.25097015	0.366308633	-0.115
cg19592472	I	37	20	3052274	OXT;OXT	0.5	0.00432	2.36443	0.41249171	0.527929798	-0.115
cg14921416	I	37	20	3693158		0.42	0.02991	1.52413	0.29969181	0.414410942	-0.115
cg17978727	II	37	20	4991028	SLC23A2	0.46	0.01197	1.92207	0.40722613	0.521792885	-0.115
cg20039443	I	37	20	50108912	NFATC2;NFATC2;NF	0.5	0.00432	2.36443	0.42525311	0.54032527	-0.115
cg21279603	II	37	21	46963527	SLC19A1	0.54	0.0014	2.85345	0.58013325	0.694825225	-0.115
cg18247179	II	37	22	19436867	C22orf39;C22orf39	0.54	0.0014	2.85345	0.86169348	0.746860735	0.115
cg21444731	II	37	22	24034126	RGL4;LOC91316;RG	0.42	0.02991	1.52413	0.6496915	0.535037994	0.115
cg25044876	II	37	22	43041146	CYB5R3;CYB5R3;CY	0.42	0.02991	1.52413	0.3681245	0.483073751	-0.115
cg11344005	II	37	22	50987527	KLHDC7B	0.58	0.00041	3.39184	0.41909885	0.303831683	0.115
cg17023856	II	37	1	2036508	PRKCZ;PRKCZ;PRKC	0.46	0.01197	1.92207	0.64402722	0.758414534	-0.114
cg24439334	I	37	1	2345374	PEX10;PEX10	0.46	0.01197	1.92207	0.77748587	0.663572267	0.114
cg23040782	II	37	1	6762215	DNAJC11	0.46	0.01197	1.92207	0.4498806	0.563659765	-0.114
cg27538859	II	37	1	15106700	KIAA1026;KIAA102(	0.46	0.01197	1.92207	0.75941898	0.645688411	0.114
cg12206846	II	37	1	19292493		0.5	0.00432	2.36443	0.60742483	0.493594184	0.114
cg20146241	I	37	1	24861604	RCAN3	0.54	0.0014	2.85345	0.18805715	0.073783699	0.114
cg21909192	II	37	1	54692785	SSBP3;SSBP3;SSBP3	0.54	0.0014	2.85345	0.61726889	0.731581455	-0.114
cg23715603	II	37	1	55278975	C1orf177;C1orf177	0.46	0.01197	1.92207	0.70245818	0.588560036	0.114
cg13229972	II	37	1	110335018		0.46	0.01197	1.92207	0.26877357	0.382887762	-0.114
cg19718359	II	37	1	111035897		0.58	0.00041	3.39184	0.76517745	0.879538874	-0.114
cg18146927	II	37	1	200835888		0.5	0.00432	2.36443	0.67091801	0.557088066	0.114
cg23683497	II	37	1	203192517	CHIT1	0.5	0.00432	2.36443	0.72581594	0.612021603	0.114
cg09259843	II	37	2	25452362		0.46	0.01197	1.92207	0.52681872	0.640673145	-0.114
cg17786894	II	37	2	65131556		0.5	0.00432	2.36443	0.59828706	0.71269513	-0.114
cg23262488	II	37	2	112468472		0.42	0.02991	1.52413	0.2528254	0.366975653	-0.114
cg06867089	II	37	2	218867385		0.46	0.01197	1.92207	0.69704406	0.810640131	-0.114
cg18664514	II	37	2	219851181	FEV	0.54	0.0014	2.85345	0.43582032	0.321943757	0.114
cg02253499	II	37	3	12602046	MKRN2	0.46	0.01197	1.92207	0.77341277	0.659027996	0.114
cg27310486	II	37	3	49200037	CCDC71	0.54	0.0014	2.85345	0.51232664	0.625924698	-0.114
cg10748355	II	37	3	62861796	CADPS;CADPS;CADI	0.5	0.00432	2.36443	0.15475616	0.268397359	-0.114
cg03457142	II	37	3	71804859	EIF4E3;EIF4E3	0.58	0.00041	3.39184	0.522609	0.636905381	-0.114
cg09543255	II	37	3	170302640	SLC7A14	0.58	0.00041	3.39184	0.1250792	0.238942566	-0.114

cg11134430	II	37	3	193987426		0.5	0.00432	2.36443	0.24252145	0.356717454	-0.114
cg03296935	II	37	3	195946851	OSTalpha	0.46	0.01197	1.92207	0.34639128	0.45994139	-0.114
cg20296343	II	37	4	206442	ZNF876P	0.46	0.01197	1.92207	0.27455482	0.389030361	-0.114
cg01161042	II	37	4	2322052	ZFYVE28	0.54	0.0014	2.85345	0.72446629	0.609976178	0.114
cg15885430	II	37	5	57069		0.5	0.00432	2.36443	0.7744131	0.660116226	0.114
cg03335624	II	37	5	73934942	ENC1	0.42	0.02991	1.52413	0.23430338	0.347965742	-0.114
cg20417024	II	37	5	76028950	F2R	0.46	0.01197	1.92207	0.83089054	0.717251904	0.114
cg16246489	II	37	5	134735675	H2AFY;H2AFY;H2AF	0.42	0.02991	1.52413	0.18248048	0.296090202	-0.114
cg03814957	II	37	5	149493099	CSF1R	0.5	0.00432	2.36443	0.67665559	0.791030874	-0.114
cg07475151	II	37	5	172382825	LOC100268168;LO(	0.5	0.00432	2.36443	0.21858847	0.332850558	-0.114
cg27365342	II	37	6	12881417	PHACTR1	0.5	0.00432	2.36443	0.70858449	0.822145515	-0.114
cg27641532	II	37	6	27648317		0.5	0.00432	2.36443	0.28361927	0.397806899	-0.114
cg12991385	II	37	6	29599259	GABBR1;GABBR1	0.62	0.0001	3.98291	0.53402667	0.647692476	-0.114
cg19712979	II	37	6	33173307	HSD17B8	0.5	0.00432	2.36443	0.6949124	0.580942811	0.114
cg14375205	II	37	6	36618002		0.5	0.00432	2.36443	0.56233613	0.676368109	-0.114
cg15496866	II	37	6	40491590	LRFN2	0.46	0.01197	1.92207	0.806036	0.69203323	0.114
cg01055696	I	37	6	164255524		0.46	0.01197	1.92207	0.70900936	0.823020082	-0.114
cg04650322	II	37	7	119499		0.62	0.0001	3.98291	0.30518279	0.419669281	-0.114
cg21920560	II	37	7	1138716	C7orf50;C7orf50;C7	0.46	0.01197	1.92207	0.57042306	0.456089273	0.114
cg26402169	I	37	7	1883139	MAD1L1;MAD1L1;M	0.54	0.0014	2.85345	0.66501328	0.551241303	0.114
cg20297638	II	37	7	2563670	LFNG;LFNG;LFNG;LI	0.54	0.0014	2.85345	0.63895121	0.753282987	-0.114
cg10042478	II	37	7	48009556	HUS1	0.54	0.0014	2.85345	0.5291708	0.643609033	-0.114
cg18952796	I	37	7	98246633	NPTX2;NPTX2	0.42	0.02991	1.52413	0.03888484	0.152806413	-0.114
cg02564523	II	37	7	102073539	ORAI2;ORAI2	0.5	0.00432	2.36443	0.334575	0.220806565	0.114
cg21932452	II	37	7	150702471	NOS3	0.62	0.0001	3.98291	0.60571848	0.719604965	-0.114
cg26161004	II	37	8	11413186	BLK	0.46	0.01197	1.92207	0.4364771	0.550266253	-0.114
cg05486260	II	37	8	139206451	FAM135B	0.5	0.00432	2.36443	0.65027103	0.764279266	-0.114
cg00021855	II	37	8	144690956	PYCRL	0.42	0.02991	1.52413	0.54588465	0.431569087	0.114
cg20633213	II	37	10	25183825	PRTFDC1	0.42	0.02991	1.52413	0.70232256	0.588214971	0.114
cg26132114	II	37	10	44223934		0.42	0.02991	1.52413	0.59773793	0.712027439	-0.114
cg00928816	II	37	10	63809098	ARID5B	0.46	0.01197	1.92207	0.73484252	0.620585649	0.114
cg07630274	II	37	10	71583235	COL13A1;COL13A1;	0.54	0.0014	2.85345	0.81685543	0.703179921	0.114
cg11633461	II	37	10	73534338	C10orf54;CDH23	0.42	0.02991	1.52413	0.40617845	0.519726088	-0.114

cg17485681	I	37	10	73565625	CDH23	0.54	0.0014	2.85345	0.64055483	0.754343176	-0.114
cg20205477	I	37	10	74113178	DNAJB12;DNAJB12	0.46	0.01197	1.92207	0.79466124	0.909040936	-0.114
cg01481646	II	37	10	82217548	TSPAN14;TSPAN14	0.46	0.01197	1.92207	0.41913217	0.533468466	-0.114
cg00167275	I	37	10	88854588	FAM35A;GLUD1;GL	0.46	0.01197	1.92207	0.21921056	0.105522543	0.114
cg03628800	II	37	10	129702726		0.5	0.00432	2.36443	0.68702931	0.573267418	0.114
cg06354543	II	37	10	130376572		0.42	0.02991	1.52413	0.80227629	0.688115478	0.114
cg06715410	II	37	10	134407912	INPP5A	0.5	0.00432	2.36443	0.65185561	0.53786978	0.114
cg19025811	I	37	11	2206157		0.54	0.0014	2.85345	0.71358801	0.82782525	-0.114
cg25754143	II	37	11	2445316	TRPM5	0.54	0.0014	2.85345	0.70315424	0.589369827	0.114
cg03158561	II	37	11	8705959	RPL27A;SNORA45	0.46	0.01197	1.92207	0.62430917	0.510807252	0.114
cg21741081	II	37	11	60634606	ZP1	0.46	0.01197	1.92207	0.77106575	0.656860053	0.114
cg23917844	I	37	11	61314901	SYT7	0.46	0.01197	1.92207	0.81128074	0.696907665	0.114
cg10859442	II	37	11	73716367	UCP3;UCP3	0.46	0.01197	1.92207	0.76299408	0.648567314	0.114
cg14883291	II	37	11	86383761	ME3;ME3;ME3	0.54	0.0014	2.85345	0.25392997	0.36750616	-0.114
cg03970229	II	37	11	92702507	MTNR1B	0.71	4.57E-06	5.34042	0.40802345	0.521579396	-0.114
cg07188648	II	37	11	111402694	C11orf88;C11orf88	0.46	0.01197	1.92207	0.26773196	0.381239815	-0.114
cg12961889	I	37	11	113749001		0.58	0.00041	3.39184	0.64238574	0.528424636	0.114
cg12368752	II	37	11	117800233	TMPRSS13	0.54	0.0014	2.85345	0.54527576	0.659613428	-0.114
cg13019868	II	37	11	128694101		0.5	0.00432	2.36443	0.45132084	0.565545778	-0.114
cg22777668	II	37	12	6881303	LAG3	0.54	0.0014	2.85345	0.24878441	0.363239854	-0.114
cg19115204	II	37	12	64782908	C12orf56;C12orf56	0.54	0.0014	2.85345	0.81758426	0.703728102	0.114
cg11265839	II	37	12	96643565	ELK3	0.42	0.02991	1.52413	0.52181154	0.636275312	-0.114
cg16671238	II	37	13	20987145	CRYL1	0.5	0.00432	2.36443	0.47473446	0.588567195	-0.114
cg01004063	II	37	14	88606476		0.5	0.00432	2.36443	0.49295645	0.378657304	0.114
cg25132241	II	37	14	92396859	FBLN5	0.54	0.0014	2.85345	0.68202769	0.796444845	-0.114
cg14290450	II	37	14	100614523	DEGS2	0.46	0.01197	1.92207	0.82229712	0.708491019	0.114
cg26216433	II	37	14	104346591		0.58	0.00041	3.39184	0.19633005	0.30993644	-0.114
cg02342791	II	37	15	31508943		0.46	0.01197	1.92207	0.35496172	0.468880081	-0.114
cg01042641	I	37	16	1575979	IFT140	0.58	0.00041	3.39184	0.61340246	0.499848683	0.114
cg03163545	II	37	16	1593415	IFT140;TMEM204	0.58	0.00041	3.39184	0.21663921	0.330289705	-0.114
cg01232511	I	37	16	2867446	PRSS21;PRSS21;PRS	0.5	0.00432	2.36443	0.63168109	0.74615815	-0.114
cg03356595	I	37	16	51187807		0.46	0.01197	1.92207	0.11646592	0.229991233	-0.114
cg16471830	II	37	16	66400569	CDH5;CDH5	0.54	0.0014	2.85345	0.29404746	0.408372469	-0.114

cg08080174	I	37	16	72992580	ZFHX3;ZFHX3	0.54	0.0014	2.85345	0.73720447	0.850980424	-0.114
cg06389950	II	37	16	75240536	CTRB2	0.42	0.02991	1.52413	0.66251582	0.548515243	0.114
cg26947831	I	37	16	86018811		0.5	0.00432	2.36443	0.41032474	0.523846638	-0.114
cg00270654	I	37	16	87735724	LOC100129637	0.54	0.0014	2.85345	0.78409778	0.670079423	0.114
cg04032292	II	37	16	88973054	CBFA2T3;CBFA2T3	0.5	0.00432	2.36443	0.39507795	0.509444889	-0.114
cg04042333	I	37	17	1104665		0.62	0.0001	3.98291	0.86389684	0.749746829	0.114
cg17933764	II	37	17	27069671	TRAF4;NEK8	0.42	0.02991	1.52413	0.66930165	0.555621907	0.114
cg07146073	I	37	17	29852668	RAB11FIP4	0.46	0.01197	1.92207	0.73675503	0.622551011	0.114
cg25135457	I	37	17	40715244	COASY;COASY;COAS	0.42	0.02991	1.52413	0.80845439	0.694943565	0.114
cg04703951	I	37	17	43578652		0.71	4.57E-06	5.34042	0.62964976	0.516024088	0.114
cg01521220	II	37	17	46233799	MIR1203;SKAP1;SK	0.42	0.02991	1.52413	0.19121625	0.305103866	-0.114
cg25255850	II	37	17	46625332		0.71	4.57E-06	5.34042	0.24964587	0.36331189	-0.114
cg19710451	II	37	17	46654202	HOXB4	0.46	0.01197	1.92207	0.18401427	0.29829084	-0.114
cg10588962	II	37	17	46667587	LOC404266;LOC404	0.5	0.00432	2.36443	0.11451033	0.228546294	-0.114
cg03717570	I	37	17	73316496	GRB2;GRB2	0.42	0.02991	1.52413	0.92615845	0.812107332	0.114
cg10053073	II	37	17	73623182	RECQL5	0.46	0.01197	1.92207	0.4677403	0.353615316	0.114
cg08837215	II	37	17	74443080	UBE2O	0.5	0.00432	2.36443	0.71539521	0.829054269	-0.114
cg26623554	II	37	17	79984422	LRRC45	0.5	0.00432	2.36443	0.53201268	0.645825565	-0.114
cg15727507	II	37	17	80981575	B3GNTL1	0.54	0.0014	2.85345	0.53936164	0.653180716	-0.114
cg15439862	II	37	18	28622593	DSC3;DSC3	0.54	0.0014	2.85345	0.21567223	0.329600131	-0.114
cg24299136	I	37	19	2511707	GNG7	0.42	0.02991	1.52413	0.86068971	0.747095149	0.114
cg25597797	II	37	19	3589762	GIPC3	0.42	0.02991	1.52413	0.74269244	0.629048293	0.114
cg08315613	II	37	19	11074303	SMARCA4;SMARCA	0.46	0.01197	1.92207	0.61282825	0.499003487	0.114
cg24084564	II	37	19	39892799		0.42	0.02991	1.52413	0.62345748	0.50956056	0.114
cg10170269	II	37	20	62256006	GMEB2	0.5	0.00432	2.36443	0.77273102	0.886467191	-0.114
cg16431978	II	37	21	31797932	KRTAP13-3	0.58	0.00041	3.39184	0.75247744	0.638712936	0.114
cg23122901	II	37	22	19880135	TXNRD2	0.62	0.0001	3.98291	0.43270096	0.319039134	0.114
cg20339720	I	37	22	45072491	PRR5;PRR5;PRR5;PI	0.5	0.00432	2.36443	0.20550212	0.31969976	-0.114
cg23346237	II	37	22	46685655	TTC38	0.46	0.01197	1.92207	0.72155428	0.607677349	0.114
cg12705326	II	37	1	6158691	KCNAB2;KCNAB2	0.58	0.00041	3.39184	0.74698655	0.860353935	-0.113
cg02117102	II	37	1	22210229	HSPG2	0.46	0.01197	1.92207	0.67038675	0.557073538	0.113
cg19743891	II	37	1	26644573	UBXN11;UBXN11;C	0.58	0.00041	3.39184	0.18678695	0.074156486	0.113
cg12627726	II	37	1	41290409	KCNQ4;KCNQ4	0.42	0.02991	1.52413	0.66537592	0.552446962	0.113

cg01048810	II	37	1	45142815	C1orf228	0.54	0.0014	2.85345	0.46295105	0.575882362	-0.113
cg05132925	II	37	1	110438827		0.58	0.00041	3.39184	0.40279231	0.516241214	-0.113
cg19627034	II	37	1	110476668		0.46	0.01197	1.92207	0.2349911	0.347844922	-0.113
cg16488065	II	37	1	204656750		0.58	0.00041	3.39184	0.79486759	0.682070786	0.113
cg14284394	II	37	1	206947477		0.46	0.01197	1.92207	0.59305586	0.706328244	-0.113
cg04304121	II	37	2	29236134	FAM179A	0.54	0.0014	2.85345	0.40014431	0.513611165	-0.113
cg13576290	II	37	2	29256737	FAM179A	0.5	0.00432	2.36443	0.6754297	0.788776631	-0.113
cg16240816	II	37	2	65861662		0.46	0.01197	1.92207	0.72251952	0.609387275	0.113
cg12391643	II	37	2	85316997		0.42	0.02991	1.52413	0.71781631	0.604390472	0.113
cg26874229	I	37	2	105853672		0.42	0.02991	1.52413	0.58849125	0.475169924	0.113
cg07484739	II	37	2	177356020		0.42	0.02991	1.52413	0.53759153	0.424519778	0.113
cg19040077	II	37	2	242702749	D2HGDH	0.46	0.01197	1.92207	0.48710514	0.599958952	-0.113
cg20850016	II	37	3	5165254	ARL8B	0.46	0.01197	1.92207	0.14084197	0.253779386	-0.113
cg17183531	II	37	3	48603978	COL7A1	0.58	0.00041	3.39184	0.68819096	0.800951464	-0.113
cg14184400	II	37	3	49460057	AMT;AMT;AMT;NIC	0.46	0.01197	1.92207	0.37435381	0.487727534	-0.113
cg11932158	II	37	3	155422129	PLCH1	0.54	0.0014	2.85345	0.22323629	0.336008834	-0.113
cg10461878	II	37	3	158904717	IQCJ;IQCJ	0.46	0.01197	1.92207	0.70151871	0.58805892	0.113
cg04252044	II	37	3	188664747		0.67	2.34E-05	4.63072	0.78126177	0.894522984	-0.113
cg03891268	I	37	3	195920019		0.5	0.00432	2.36443	0.86723356	0.753925921	0.113
cg15652666	I	37	4	3487436	DOK7;DOK7	0.46	0.01197	1.92207	0.39269755	0.279879614	0.113
cg21186966	II	37	4	5401430	STK32B	0.42	0.02991	1.52413	0.71352131	0.600773235	0.113
cg08528970	II	37	4	76640579		0.54	0.0014	2.85345	0.20339646	0.316458621	-0.113
cg04425005	II	37	4	146783253	ZNF827	0.46	0.01197	1.92207	0.74643123	0.859669868	-0.113
cg00329411	II	37	5	55881858		0.46	0.01197	1.92207	0.56809398	0.681481572	-0.113
cg26549701	II	37	5	72599503		0.5	0.00432	2.36443	0.18584726	0.298756001	-0.113
cg10949007	II	37	5	95159614	GLRX;GLRX	0.5	0.00432	2.36443	0.15744317	0.270542319	-0.113
cg06003656	II	37	5	166405676		0.5	0.00432	2.36443	0.1732123	0.285920726	-0.113
cg27294324	II	37	5	169070065	DOCK2	0.5	0.00432	2.36443	0.77512593	0.888024679	-0.113
cg27063969	II	37	5	169133966	DOCK2	0.5	0.00432	2.36443	0.78934533	0.675993985	0.113
cg13054640	II	37	6	30647148	KIAA1949;KIAA194	0.62	0.0001	3.98291	0.41989894	0.306969802	0.113
cg04536765	II	37	6	31621761	BAT3;BAT3;BAT3	0.58	0.00041	3.39184	0.72803247	0.840946509	-0.113
cg22878489	I	37	6	33245701	B3GALT4	0.42	0.02991	1.52413	0.51032435	0.397270451	0.113
cg11075561	II	37	6	36088380		0.54	0.0014	2.85345	0.69259687	0.579770877	0.113

cg27158519	II	37	6	113580203		0.5	0.00432	2.36443	0.82155023	0.708546593	0.113
cg03392753	II	37	7	1005576	COX19	0.42	0.02991	1.52413	0.14011076	0.253166023	-0.113
cg07131210	I	37	7	1025825	CYP2W1	0.54	0.0014	2.85345	0.66710585	0.779929183	-0.113
cg19254152	II	37	7	1083473	C7orf50;C7orf50;C7	0.54	0.0014	2.85345	0.23439228	0.347030504	-0.113
cg00083596	II	37	7	3996856	SDK1	0.46	0.01197	1.92207	0.83538907	0.722855422	0.113
cg09022230	II	37	7	5457225	TNRC18	0.5	0.00432	2.36443	0.74745602	0.860245671	-0.113
cg12127159	II	37	7	33809972		0.54	0.0014	2.85345	0.68708592	0.574288924	0.113
cg00260937	II	37	7	128520193	KCP	0.42	0.02991	1.52413	0.30830854	0.421421775	-0.113
cg03059073	II	37	7	150130708		0.46	0.01197	1.92207	0.16104976	0.274288628	-0.113
cg17112266	II	37	7	150737198	ABCB8	0.54	0.0014	2.85345	0.33815351	0.451110453	-0.113
cg20514973	II	37	8	102387937		0.54	0.0014	2.85345	0.7105959	0.823889833	-0.113
cg24804707	II	37	8	134094173	TG;SLA;SLA	0.42	0.02991	1.52413	0.31775902	0.430913018	-0.113
cg13474450	II	37	8	142222685	SLC45A4	0.5	0.00432	2.36443	0.74977667	0.862375068	-0.113
cg24891660	I	37	8	145003653	PLEC1;PLEC1;PLEC1	0.58	0.00041	3.39184	0.55978002	0.446917554	0.113
cg14571284	II	37	9	130516077	SH2D3C;SH2D3C;SH	0.5	0.00432	2.36443	0.72134997	0.834810324	-0.113
cg13435263	I	37	9	139143522		0.58	0.00041	3.39184	0.5619621	0.67452801	-0.113
cg26494044	II	37	10	129760954	PTPRE	0.42	0.02991	1.52413	0.60031303	0.487757516	0.113
cg04780434	I	37	10	134402787	INPP5A	0.58	0.00041	3.39184	0.25006786	0.363567102	-0.113
cg08824847	II	37	11	35052388		0.5	0.00432	2.36443	0.35034702	0.463266458	-0.113
cg24674703	II	37	11	60869960	CD5;CD5	0.46	0.01197	1.92207	0.39214601	0.279642066	0.113
cg15829423	II	37	11	68138862	LRP5	0.42	0.02991	1.52413	0.79283278	0.680249173	0.113
cg00464927	I	37	11	68139121	LRP5	0.5	0.00432	2.36443	0.64720972	0.533965289	0.113
cg19478111	I	37	11	73359987	PLEKHB1;PLEKHB1;	0.42	0.02991	1.52413	0.67142233	0.558064927	0.113
cg23507834	II	37	11	93824553	HEPHL1	0.46	0.01197	1.92207	0.83135983	0.71831631	0.113
cg24983752	II	37	11	115931334		0.5	0.00432	2.36443	0.57528816	0.462159595	0.113
cg01519094	II	37	11	125034181	PKNOX2	0.54	0.0014	2.85345	0.17420373	0.287363161	-0.113
cg06826457	II	37	12	12867669		0.5	0.00432	2.36443	0.30241198	0.189386045	0.113
cg13700315	II	37	12	34489827		0.58	0.00041	3.39184	0.50177792	0.614927767	-0.113
cg17866692	II	37	12	110228049	TRPV4;TRPV4	0.58	0.00041	3.39184	0.67983795	0.566984448	0.113
cg15234492	II	37	12	122019076	KDM2B;KDM2B	0.5	0.00432	2.36443	0.30519409	0.418569723	-0.113
cg18637761	II	37	12	123468780	PITPNM2	0.46	0.01197	1.92207	0.20987995	0.322546377	-0.113
cg15243578	I	37	12	123469284	PITPNM2	0.5	0.00432	2.36443	0.46066568	0.573181528	-0.113
cg26764846	II	37	13	20797693	GJB6;GJB6;GJB6;GJ	0.54	0.0014	2.85345	0.71046579	0.823911958	-0.113

cg25221774	II	37	13	101173669	PCCA;PCCA	0.67	2.34E-05	4.63072	0.60537019	0.717910777	-0.113
cg01956420	I	37	13	110959668	COL4A1;COL4A2;CC	0.42	0.02991	1.52413	0.0217033	0.135118225	-0.113
cg02219601	II	37	14	35835511		0.58	0.00041	3.39184	0.25778696	0.371258556	-0.113
cg12827637	I	37	14	69256791	ZFP36L1	0.42	0.02991	1.52413	0.49947012	0.386740333	0.113
cg16267322	II	37	14	92864074	SLC24A4;SLC24A4;S	0.58	0.00041	3.39184	0.78496406	0.898325072	-0.113
cg10761315	I	37	14	104552034	ASPG	0.46	0.01197	1.92207	0.13395501	0.246989648	-0.113
cg12063580	II	37	14	105783627	PACS2;PACS2	0.58	0.00041	3.39184	0.31818798	0.431124868	-0.113
cg26767198	II	37	15	29078705		0.5	0.00432	2.36443	0.38114943	0.4941469	-0.113
cg06070324	II	37	15	75322058	PPCDC	0.42	0.02991	1.52413	0.26183298	0.375104017	-0.113
cg04643527	II	37	16	3312946		0.58	0.00041	3.39184	0.70363607	0.817087776	-0.113
cg00510320	II	37	16	67686943	RLTPR	0.42	0.02991	1.52413	0.60133541	0.714273221	-0.113
cg10520672	II	37	16	86018680		0.46	0.01197	1.92207	0.16061052	0.273581317	-0.113
cg25627098	II	37	17	7328734	C17orf74	0.5	0.00432	2.36443	0.26195619	0.374834912	-0.113
cg07710335	II	37	17	15151869	PMP22;PMP22;PM	0.46	0.01197	1.92207	0.81537425	0.70251817	0.113
cg00005437	II	37	17	18010740	DRG2;MYO15A	0.46	0.01197	1.92207	0.69404359	0.58131391	0.113
cg15036326	II	37	17	27048708	RPL23A;SNORD4A	0.5	0.00432	2.36443	0.73981178	0.626919232	0.113
cg15298286	II	37	17	62075324	C17orf72	0.67	2.34E-05	4.63072	0.23683347	0.35011593	-0.113
cg14020176	I	37	17	72764985	SLC9A3R1	0.67	2.34E-05	4.63072	0.72327077	0.836031465	-0.113
cg25923214	I	37	17	74864205	MGAT5B	0.42	0.02991	1.52413	0.45872908	0.571969959	-0.113
cg14011789	I	37	17	75452044	SEPT9;SEPT9;SEPT9	0.62	0.0001	3.98291	0.88121752	0.768084541	0.113
cg03633458	I	37	19	852284	ELANE	0.5	0.00432	2.36443	0.37562458	0.488573882	-0.113
cg09788082	I	37	19	1163485	SBNO2	0.5	0.00432	2.36443	0.81938456	0.93231936	-0.113
cg21550623	II	37	19	2734683	SLC39A3;SLC39A3	0.42	0.02991	1.52413	0.66508456	0.552094017	0.113
cg06561886	I	37	19	10736299	SLC44A2;SLC44A2;S	0.54	0.0014	2.85345	0.68875195	0.801493427	-0.113
cg10372770	II	37	19	12377156		0.42	0.02991	1.52413	0.61065798	0.723269507	-0.113
cg18232841	II	37	19	35615103	FXYD3;FXYD3;FXYD	0.5	0.00432	2.36443	0.56026366	0.447044041	0.113
cg18651578	II	37	20	5844315	C20orf196	0.54	0.0014	2.85345	0.80892407	0.922319296	-0.113
cg12413156	II	37	20	62368256	LIME1	0.46	0.01197	1.92207	0.48067783	0.367360488	0.113
cg13120798	II	37	22	21090264	PI4KA;PI4KA	0.46	0.01197	1.92207	0.4744126	0.587104909	-0.113
cg26999345	II	37	22	37584441	C1QTNF6;C1QTNF6	0.54	0.0014	2.85345	0.29254474	0.40511486	-0.113
cg21771250	II	37	22	40406049	FAM83F	0.42	0.02991	1.52413	0.45938439	0.572462274	-0.113
cg04090867	II	37	1	10794229	CASZ1;CASZ1	0.5	0.00432	2.36443	0.71537433	0.603210443	0.112
cg00719568	II	37	1	113239645	MOV10;MOV10	0.54	0.0014	2.85345	0.78313812	0.671390331	0.112

cg18645493	II	37	1	154167437	MIR190B	0.67	2.34E-05	4.63072	0.3416853	0.453959765	-0.112
cg01782486	II	37	1	154986512	ZBTB7B	0.54	0.0014	2.85345	0.64736739	0.535445537	0.112
cg00160981	II	37	1	161691911	FCRLB	0.46	0.01197	1.92207	0.23734458	0.34917322	-0.112
cg01598007	II	37	1	167063581	DUSP27	0.5	0.00432	2.36443	0.60213711	0.713681894	-0.112
cg27179622	I	37	1	226127290	LEFTY2	0.5	0.00432	2.36443	0.60321325	0.49094571	0.112
cg08571738	II	37	2	2605398		0.46	0.01197	1.92207	0.72801317	0.839913898	-0.112
cg18122743	II	37	2	41921124		0.5	0.00432	2.36443	0.73747905	0.625857346	0.112
cg07011538	II	37	2	47497026		0.54	0.0014	2.85345	0.71024948	0.822672149	-0.112
cg14325025	II	37	2	102608155	IL1R2	0.46	0.01197	1.92207	0.15701085	0.268920386	-0.112
cg21838979	II	37	2	106681945	C2orf40	0.58	0.00041	3.39184	0.14491103	0.257115781	-0.112
cg17112958	II	37	2	133426934	LYPD1;LYPD1	0.46	0.01197	1.92207	0.1531334	0.265093778	-0.112
cg03051392	II	37	3	50360176	HYAL2;HYAL2;HYAL	0.58	0.00041	3.39184	0.47377958	0.585543401	-0.112
cg26460678	I	37	3	50360667	HYAL2	0.54	0.0014	2.85345	0.55200249	0.664256238	-0.112
cg00171092	II	37	3	124531371	ITGB5	0.5	0.00432	2.36443	0.81323691	0.701501181	0.112
cg00701064	II	37	4	6280414	WFS1;WFS1	0.5	0.00432	2.36443	0.34556794	0.457123492	-0.112
cg06109284	II	37	4	6965057	TBC1D14;TBC1D14	0.42	0.02991	1.52413	0.7421671	0.629751812	0.112
cg18949666	II	37	4	170187451	SH3RF1	0.5	0.00432	2.36443	0.73759832	0.849503831	-0.112
cg00220335	II	37	5	1343783	CLPTM1L	0.46	0.01197	1.92207	0.60673247	0.494950294	0.112
cg26389330	II	37	5	151057860	SPARC	0.54	0.0014	2.85345	0.30535613	0.417842899	-0.112
cg26694831	I	37	5	178763419	ADAMTS2;ADAMTS	0.42	0.02991	1.52413	0.84448549	0.956113367	-0.112
cg09897374	I	37	6	28584076		0.42	0.02991	1.52413	0.11880563	0.230483582	-0.112
cg03343571	I	37	6	30039175	RNF39;RNF39	0.42	0.02991	1.52413	0.15461031	0.266138375	-0.112
cg05824482	II	37	6	30126161	TRIM10;TRIM10	0.5	0.00432	2.36443	0.73388894	0.845479901	-0.112
cg03216698	II	37	6	32021500	TNXB	0.42	0.02991	1.52413	0.54546485	0.433788054	0.112
cg10666909	II	37	6	32820249	TAP1	0.46	0.01197	1.92207	0.71857536	0.606560003	0.112
cg11796996	I	37	6	33280159	TAPBP;TAPBP;TAPE	0.5	0.00432	2.36443	0.54249424	0.430881411	0.112
cg08858220	II	37	6	36805317	CPNE5	0.42	0.02991	1.52413	0.64812608	0.536032985	0.112
cg08273233	II	37	6	87724979	HTR1E	0.46	0.01197	1.92207	0.73673262	0.624847266	0.112
cg15931839	II	37	6	111880530	TRAF3IP2;TRAF3IP2	0.42	0.02991	1.52413	0.28839962	0.175982133	0.112
cg07404200	II	37	6	166876585	RPS6KA2;RPS6KA2	0.54	0.0014	2.85345	0.42489639	0.536580607	-0.112
cg15196529	II	37	6	167658907		0.54	0.0014	2.85345	0.75463223	0.642579972	0.112
cg13588517	II	37	6	170562720		0.42	0.02991	1.52413	0.66461375	0.552936065	0.112
cg06789500	II	37	7	2109450	MAD1L1;MAD1L1;N	0.58	0.00041	3.39184	0.70619611	0.818188257	-0.112

cg17228698	II	37	7	2451914	CHST12	0.58	0.00041	3.39184	0.71496241	0.827225241	-0.112
cg03040622	II	37	7	5536937	MIR589;FBXL18	0.5	0.00432	2.36443	0.39869602	0.510372919	-0.112
cg01412469	II	37	7	41732237	INHBA;LOC285954;	0.62	0.0001	3.98291	0.34282567	0.454888396	-0.112
cg23192873	II	37	7	91809044		0.42	0.02991	1.52413	0.36289824	0.475007439	-0.112
cg17150663	II	37	7	128551672	KCP;KCP	0.5	0.00432	2.36443	0.59725178	0.709416382	-0.112
cg23630442	II	37	8	18245225		0.67	2.34E-05	4.63072	0.61132868	0.723791254	-0.112
cg15257489	II	37	8	49430059		0.46	0.01197	1.92207	0.70782608	0.595363636	0.112
cg14266248	II	37	8	62606211	ASPH;ASPH;ASPH;A	0.42	0.02991	1.52413	0.69170249	0.803955934	-0.112
cg25327343	II	37	8	120221797	MAL2	0.5	0.00432	2.36443	0.47865903	0.590774123	-0.112
cg25877009	I	37	8	142288517		0.5	0.00432	2.36443	0.65256829	0.765023177	-0.112
cg02086839	II	37	8	144403439	TOP1MT	0.54	0.0014	2.85345	0.67495521	0.787145726	-0.112
cg00055726	II	37	9	136445425	FAM163B	0.5	0.00432	2.36443	0.19624889	0.084241925	0.112
cg10556349	II	37	10	835070		0.46	0.01197	1.92207	0.34954898	0.461160438	-0.112
cg20341089	II	37	10	121447367		0.42	0.02991	1.52413	0.6269675	0.738721955	-0.112
cg19038027	II	37	10	134306852		0.5	0.00432	2.36443	0.73819966	0.626141968	0.112
cg19623624	I	37	10	135278901	LOC619207	0.46	0.01197	1.92207	0.61919095	0.731189746	-0.112
cg11611244	II	37	11	1507263	HCCA2	0.42	0.02991	1.52413	0.66518296	0.553363569	0.112
cg17229197	II	37	11	2542688	KCNQ1;KCNQ1	0.46	0.01197	1.92207	0.8312859	0.718951715	0.112
cg02251663	II	37	11	14281053	SPON1	0.42	0.02991	1.52413	0.52317568	0.634887914	-0.112
cg15675740	II	37	11	45874617	CRY2;CRY2	0.46	0.01197	1.92207	0.71314807	0.600678938	0.112
cg13526040	I	37	11	57093322	TNKS1BP1	0.42	0.02991	1.52413	0.46457137	0.352736441	0.112
cg26898077	II	37	11	67811114	TCIRG1;TCIRG1	0.62	0.0001	3.98291	0.72614014	0.838470001	-0.112
cg20267732	II	37	11	77787848	NDUFC2	0.46	0.01197	1.92207	0.65544923	0.766963535	-0.112
cg25473794	I	37	11	77921181	USP35	0.46	0.01197	1.92207	0.74658806	0.858125185	-0.112
cg04535665	II	37	11	104972135	CARD17;CASP1;CAS	0.46	0.01197	1.92207	0.46568936	0.577371105	-0.112
cg21623445	II	37	12	177995	IQSEC3	0.58	0.00041	3.39184	0.51456268	0.402845633	0.112
cg25354587	II	37	12	3590738		0.46	0.01197	1.92207	0.48609874	0.37388127	0.112
cg19374752	II	37	12	52404151	GRASP	0.67	2.34E-05	4.63072	0.41836899	0.53070528	-0.112
cg18148255	II	37	12	89476250		0.46	0.01197	1.92207	0.80866927	0.69688849	0.112
cg10283036	I	37	12	111853088	SH2B3	0.54	0.0014	2.85345	0.72563362	0.83788403	-0.112
cg14844236	I	37	12	123753212	CDK2AP1	0.58	0.00041	3.39184	0.32015313	0.432265311	-0.112
cg16692277	II	37	13	51640948	GUCY1B2	0.46	0.01197	1.92207	0.23936151	0.351022174	-0.112
cg27417606	II	37	14	21503144	RNASE13	0.58	0.00041	3.39184	0.79367957	0.681839061	0.112

cg08593009	II	37	14	94943184	SERPINA9;SERPINA9	0.5	0.00432	2.36443	0.65428236	0.541847965	0.112
cg02964434	II	37	15	31617537		0.5	0.00432	2.36443	0.57359469	0.461745892	0.112
cg06100161	II	37	15	60987894	RORA	0.54	0.0014	2.85345	0.80471747	0.693063645	0.112
cg07217653	II	37	15	101591436	LRRK1	0.46	0.01197	1.92207	0.74725224	0.634838702	0.112
cg01422009	II	37	16	125896	MPG	0.54	0.0014	2.85345	0.67223288	0.784025789	-0.112
cg00354542	II	37	16	434356	LOC100134368	0.5	0.00432	2.36443	0.24365493	0.356061547	-0.112
cg02453013	II	37	16	1297491		0.54	0.0014	2.85345	0.62142562	0.733122879	-0.112
cg01500402	II	37	16	2256716	MLST8	0.58	0.00041	3.39184	0.25976359	0.372243603	-0.112
cg26504467	II	37	16	11318653		0.5	0.00432	2.36443	0.76090803	0.64852389	0.112
cg01538731	II	37	16	87757018	KLHDC4	0.46	0.01197	1.92207	0.61839146	0.50680644	0.112
cg08721324	II	37	16	89384602	ANKRD11	0.58	0.00041	3.39184	0.5440074	0.656386812	-0.112
cg06940127	II	37	17	1665303	SERPINF1;SERPINF1	0.58	0.00041	3.39184	0.41391603	0.525781556	-0.112
cg04464719	I	37	17	3824422		0.58	0.00041	3.39184	0.50644828	0.394452844	0.112
cg26043460	II	37	17	39593631	KRT38	0.58	0.00041	3.39184	0.7598941	0.648208597	0.112
cg16826777	II	37	17	43376882	MAP3K14	0.67	2.34E-05	4.63072	0.42241669	0.534172578	-0.112
cg25839227	II	37	17	47288502	ABI3;ABI3	0.5	0.00432	2.36443	0.66279379	0.55079122	0.112
cg13747876	II	37	17	80195402	SLC16A3;SLC16A3;S	0.42	0.02991	1.52413	0.63173858	0.743407556	-0.112
cg22382836	I	37	19	723036	PALM;PALM	0.46	0.01197	1.92207	0.27526613	0.162829669	0.112
cg10643550	II	37	19	1603857	UQCR	0.58	0.00041	3.39184	0.58699302	0.69929857	-0.112
cg02020969	II	37	19	2484938		0.46	0.01197	1.92207	0.65570949	0.543274444	0.112
cg22548088	II	37	19	6271786	MLLT1	0.67	2.34E-05	4.63072	0.59672225	0.708908218	-0.112
cg14753070	II	37	19	14163842	IL27RA	0.46	0.01197	1.92207	0.76631479	0.654448279	0.112
cg18145683	II	37	19	17959281	JAK3	0.54	0.0014	2.85345	0.51713424	0.629493184	-0.112
cg14810329	II	37	19	35086653	SCGBL	0.42	0.02991	1.52413	0.61045171	0.498092158	0.112
cg24030173	II	37	19	48922140	GRIN2D	0.71	4.57E-06	5.34042	0.22031197	0.332569278	-0.112
cg17661220	II	37	20	3693179		0.46	0.01197	1.92207	0.27302412	0.384763465	-0.112
cg11316100	II	37	20	35233380	C20orf24;C20orf24	0.58	0.00041	3.39184	0.27869193	0.166397889	0.112
cg25541209	II	37	20	45947892	ZMYND8;ZMYND8;I	0.58	0.00041	3.39184	0.49047163	0.602064292	-0.112
cg12097791	II	37	20	50719777	ZFP64	0.54	0.0014	2.85345	0.2656983	0.377447438	-0.112
cg17929273	II	37	22	37967569	LGALS2	0.42	0.02991	1.52413	0.68874418	0.576384939	0.112
cg00400964	II	37	22	42325444		0.46	0.01197	1.92207	0.82069701	0.709028002	0.112
cg06967105	II	37	1	1104265	MIR429	0.54	0.0014	2.85345	0.51788228	0.406966578	0.111
cg00567930	II	37	1	2250796		0.46	0.01197	1.92207	0.76476491	0.653839536	0.111

cg15211996	II	37	1	2936768	ACTRT2	0.62	0.0001	3.98291	0.67367923	0.562815533	0.111
cg04898695	II	37	1	6208717	CHD5	0.71	4.57E-06	5.34042	0.16175597	0.272341198	-0.111
cg05944661	II	37	1	6457799		0.54	0.0014	2.85345	0.69241499	0.581345985	0.111
cg00648005	I	37	1	9086914	SLC2A7	0.5	0.00432	2.36443	0.63150512	0.742478239	-0.111
cg24435669	II	37	1	15546149	TMEM51;TMEM51;	0.46	0.01197	1.92207	0.66752292	0.556544664	0.111
cg24502330	II	37	1	20914028	CDA	0.46	0.01197	1.92207	0.45532897	0.565907839	-0.111
cg24492886	II	37	1	28474511	PTAFR;PTAFR;PTAF	0.46	0.01197	1.92207	0.83360279	0.722634348	0.111
cg13100137	II	37	1	37937945		0.71	4.57E-06	5.34042	0.48866107	0.600066416	-0.111
cg00944580	II	37	1	58333307	DAB1;MIR548D2	0.54	0.0014	2.85345	0.7438917	0.633265658	0.111
cg00571483	II	37	1	59046173		0.54	0.0014	2.85345	0.30940078	0.420307112	-0.111
cg17936236	II	37	1	167520305	CREG1	0.62	0.0001	3.98291	0.59147004	0.702409285	-0.111
cg12343591	II	37	2	16031275		0.58	0.00041	3.39184	0.84669944	0.736190294	0.111
cg20340242	II	37	2	102608349	IL1R2;IL1R2	0.46	0.01197	1.92207	0.24122281	0.352588677	-0.111
cg07992484	II	37	2	106724161	UXS1	0.42	0.02991	1.52413	0.55993001	0.449365412	0.111
cg09152259	II	37	2	128156114		0.54	0.0014	2.85345	0.31926809	0.430000902	-0.111
cg17513592	II	37	2	202894435		0.42	0.02991	1.52413	0.70912677	0.59844435	0.111
cg20300794	I	37	2	234359705	DGKD;DGKD	0.42	0.02991	1.52413	0.78100187	0.892264132	-0.111
cg20971407	II	37	3	5022392	BHLHE40	0.5	0.00432	2.36443	0.70849487	0.597356719	0.111
cg25664938	II	37	3	119030023	CDGAP	0.46	0.01197	1.92207	0.72832994	0.838983958	-0.111
cg25250358	II	37	3	145879084	PLOD2;PLOD2;PLOI	0.5	0.00432	2.36443	0.13293994	0.243738366	-0.111
cg10085474	II	37	4	1221935	CTBP1;CTBP1	0.5	0.00432	2.36443	0.39968095	0.510243177	-0.111
cg13673960	II	37	4	1742301	TACC3	0.54	0.0014	2.85345	0.63156905	0.742390787	-0.111
cg01787559	II	37	4	3450770	HGFAC	0.46	0.01197	1.92207	0.4008999	0.289633102	0.111
cg15406387	II	37	4	111397332	ENPEP;ENPEP	0.42	0.02991	1.52413	0.44633544	0.557572248	-0.111
cg03033182	II	37	4	141177619	SCOC	0.46	0.01197	1.92207	0.64514761	0.755839409	-0.111
cg01300684	II	37	4	141230118	SCOC	0.58	0.00041	3.39184	0.8099123	0.698489569	0.111
cg00049729	I	37	5	55776345		0.58	0.00041	3.39184	0.52401619	0.412941983	0.111
cg08490107	I	37	5	171774272	SH3PXD2B	0.5	0.00432	2.36443	0.87381723	0.763095903	0.111
cg03127104	II	37	6	29599250	GABBR1;GABBR1	0.58	0.00041	3.39184	0.56008669	0.670665769	-0.111
cg04276750	II	37	6	29856938	HLA-H	0.42	0.02991	1.52413	0.77459921	0.663809659	0.111
cg08420066	II	37	6	30647561	KIAA1949;KIAA194!	0.62	0.0001	3.98291	0.26258752	0.151247241	0.111
cg17755321	II	37	6	31546085	TNF	0.5	0.00432	2.36443	0.807508	0.696583055	0.111
cg22361816	I	37	6	33141798	COL11A2;COL11A2;	0.42	0.02991	1.52413	0.50400471	0.392812106	0.111

cg11772020	II	37	6	43806470		0.67	2.34E-05	4.63072	0.32010068	0.431013751	-0.111
cg03072286	II	37	6	150262044	ULBP2	0.58	0.00041	3.39184	0.14303181	0.254118798	-0.111
cg10251229	II	37	7	630581	PRKAR1B;PRKAR1B	0.54	0.0014	2.85345	0.56439844	0.675721727	-0.111
cg07690127	II	37	7	2077293	MAD1L1;MAD1L1;M	0.5	0.00432	2.36443	0.67594226	0.786697804	-0.111
cg18880986	I	37	7	5534951	FBXL18	0.5	0.00432	2.36443	0.62499089	0.513640353	0.111
cg19071544	II	37	7	30504941	NOD1	0.54	0.0014	2.85345	0.29468386	0.405705619	-0.111
cg05988548	II	37	7	38695932		0.54	0.0014	2.85345	0.42029557	0.531345733	-0.111
cg22138096	II	37	7	41772439	LOC285954	0.46	0.01197	1.92207	0.60681605	0.718290947	-0.111
cg14690467	II	37	7	148917905	ZNF282	0.54	0.0014	2.85345	0.7023022	0.812849692	-0.111
cg23903252	II	37	7	149569998	ATP6V0E2;ATP6V0E	0.5	0.00432	2.36443	0.23288132	0.343524015	-0.111
cg22615330	II	37	7	151502322	PRKAG2;PRKAG2	0.5	0.00432	2.36443	0.5551516	0.666013313	-0.111
cg03631656	I	37	8	6420770	ANGPT2;ANGPT2;A	0.42	0.02991	1.52413	0.67510766	0.786458072	-0.111
cg21926626	II	37	8	143382226	TSNARE1	0.5	0.00432	2.36443	0.50635171	0.617378084	-0.111
cg13300202	II	37	8	143407491	TSNARE1	0.46	0.01197	1.92207	0.30823224	0.419722286	-0.111
cg14381994	II	37	8	144262769		0.42	0.02991	1.52413	0.79824015	0.687010599	0.111
cg14289429	II	37	9	134139878	FAM78A	0.58	0.00041	3.39184	0.5977876	0.708767081	-0.111
cg13826666	I	37	9	136600241	SARDH;SARDH	0.5	0.00432	2.36443	0.72630126	0.615109586	0.111
cg14341131	I	37	9	138605049	KCNT1	0.54	0.0014	2.85345	0.53975443	0.428399423	0.111
cg02449762	II	37	10	5661741		0.42	0.02991	1.52413	0.50843174	0.397853045	0.111
cg26316423	II	37	10	6104137	IL2RA;IL2RA	0.46	0.01197	1.92207	0.63569597	0.524978392	0.111
cg14685948	II	37	10	71608654	COL13A1;COL13A1;	0.42	0.02991	1.52413	0.61827352	0.507667286	0.111
cg15763670	II	37	10	73473235	C10orf105;C10orf1	0.62	0.0001	3.98291	0.42257518	0.533425106	-0.111
cg09357462	II	37	10	112259305	DUSP5	0.54	0.0014	2.85345	0.64293838	0.53190406	0.111
cg24158157	I	37	10	124581726		0.5	0.00432	2.36443	0.7177396	0.606430303	0.111
cg06740578	II	37	10	131142473		0.58	0.00041	3.39184	0.80073701	0.689895889	0.111
cg26158270	II	37	11	12309622	MICALCL	0.62	0.0001	3.98291	0.6907544	0.801285853	-0.111
cg02886208	I	37	11	14281011	SPON1	0.5	0.00432	2.36443	0.80353194	0.914234496	-0.111
cg19045191	II	37	11	34167855	NAT10;NAT10	0.5	0.00432	2.36443	0.73128291	0.620334172	0.111
cg08442823	II	37	11	45951803	PHF21A;PHF21A	0.5	0.00432	2.36443	0.30947106	0.19845498	0.111
cg22101045	II	37	11	47927509		0.5	0.00432	2.36443	0.65220537	0.763267973	-0.111
cg04728402	II	37	11	62104662	ASRGL1;ASRGL1	0.62	0.0001	3.98291	0.702367	0.81313219	-0.111
cg10851168	II	37	11	70317508	SHANK2;SHANK2	0.5	0.00432	2.36443	0.76287405	0.652132796	0.111
cg20210482	I	37	11	126286493		0.54	0.0014	2.85345	0.08685572	0.198060862	-0.111

cg04468568	II	37	11	128776059	C11orf45;KCNJ5	0.54	0.0014	2.85345	0.3317107	0.443039876	-0.111
cg08707456	I	37	11	134829365		0.62	0.0001	3.98291	0.73927199	0.62836079	0.111
cg03879460	I	37	12	562272		0.62	0.0001	3.98291	0.27168568	0.160801786	0.111
cg23841186	II	37	12	53492662	IGFBP6	0.5	0.00432	2.36443	0.45429485	0.565657119	-0.111
cg16774946	I	37	13	24798195	SPATA13;SPATA13	0.46	0.01197	1.92207	0.42171749	0.310745841	0.111
cg11746996	II	37	13	69459533		0.46	0.01197	1.92207	0.56569393	0.454230851	0.111
cg20382493	II	37	13	114829170	RASA3	0.54	0.0014	2.85345	0.70776427	0.818896529	-0.111
cg04206572	II	37	13	114888999	RASA3	0.5	0.00432	2.36443	0.56068913	0.671472147	-0.111
cg20505457	II	37	14	69388953	ACTN1;ACTN1;ACTI	0.46	0.01197	1.92207	0.7123084	0.60096756	0.111
cg10530793	II	37	14	69821049	GALNTL1;GALNTL1	0.42	0.02991	1.52413	0.56257814	0.451249454	0.111
cg20385229	II	37	14	78175470	ALKBH1;C14orf156	0.46	0.01197	1.92207	0.81685957	0.705491044	0.111
cg26337624	I	37	14	105350915	KIAA0284;KIAA028	0.46	0.01197	1.92207	0.94088318	0.829945673	0.111
cg19928084	II	37	14	106855090		0.5	0.00432	2.36443	0.73365216	0.62219765	0.111
cg08369013	II	37	15	31693543		0.42	0.02991	1.52413	0.76910602	0.657870771	0.111
cg22107533	II	37	15	45028083	TRIM69;TRIM69	0.42	0.02991	1.52413	0.42001519	0.531331497	-0.111
cg25045893	II	37	15	83777051	TM6SF1;TM6SF1	0.46	0.01197	1.92207	0.16319584	0.274657645	-0.111
cg18569141	I	37	15	90727995	SEMA4B	0.5	0.00432	2.36443	0.31620835	0.205005034	0.111
cg11143485	II	37	15	101499700	LRRK1	0.58	0.00041	3.39184	0.2182857	0.329319699	-0.111
cg14237301	II	37	16	28506477	APOB48R	0.46	0.01197	1.92207	0.722836	0.834053099	-0.111
cg26663590	II	37	16	28959310		0.42	0.02991	1.52413	0.63782107	0.748440625	-0.111
cg27150412	II	37	16	31161431	PRSS36	0.46	0.01197	1.92207	0.44861648	0.559701485	-0.111
cg23684449	II	37	16	46919194	GPT2;GPT2;GPT2	0.5	0.00432	2.36443	0.43916722	0.549897205	-0.111
cg07957035	II	37	16	49623763	ZNF423	0.62	0.0001	3.98291	0.74971763	0.860760412	-0.111
cg00366252	II	37	16	85436841		0.58	0.00041	3.39184	0.35818144	0.469074746	-0.111
cg17442852	II	37	17	1478889	SLC43A2	0.71	4.57E-06	5.34042	0.65966277	0.770585624	-0.111
cg19022525	II	37	17	40664917	ATP6V0A1;ATP6V0/	0.46	0.01197	1.92207	0.84908541	0.737681225	0.111
cg19618279	I	37	17	40715228	COASY;COASY;COA:	0.46	0.01197	1.92207	0.76818739	0.657218422	0.111
cg14578363	II	37	17	42785078	DBF4B;DBF4B	0.46	0.01197	1.92207	0.84964866	0.739087075	0.111
cg23258611	II	37	17	70536160		0.58	0.00041	3.39184	0.29136753	0.402487007	-0.111
cg16548154	II	37	17	74565757	ST6GALNAC2	0.75	7.61E-07	6.11857	0.66924613	0.780257706	-0.111
cg13614440	II	37	17	76471012	DNAH17	0.42	0.02991	1.52413	0.64720339	0.536102258	0.111
cg21621482	I	37	17	79228937	SLC38A10;SLC38A1	0.5	0.00432	2.36443	0.4252592	0.536463354	-0.111
cg23596678	II	37	17	79793000	DYSFIP1	0.62	0.0001	3.98291	0.702318	0.813732986	-0.111

cg24213719	I	37	18	60263646		0.54	0.0014	2.85345	0.04360374	0.154557228	-0.111
cg25264268	I	37	19	427263	SHC2	0.5	0.00432	2.36443	0.7368548	0.848076166	-0.111
cg10746622	II	37	19	696946	PRSSL1	0.54	0.0014	2.85345	0.74976019	0.860972653	-0.111
cg02348462	II	37	19	807147	PTBP1;PTBP1;PTBP	0.46	0.01197	1.92207	0.65353521	0.764239158	-0.111
cg22372096	I	37	19	3810782	ZFR2	0.5	0.00432	2.36443	0.60304073	0.492457489	0.111
cg27552857	I	37	19	4542866	SEMA6B	0.5	0.00432	2.36443	0.54287363	0.654130167	-0.111
cg21928095	II	37	19	36100748		0.54	0.0014	2.85345	0.31817112	0.429113576	-0.111
cg14799446	II	37	19	47250607	FKRP;FKRP;STRN4;S	0.46	0.01197	1.92207	0.78393357	0.672483578	0.111
cg18655633	II	37	19	48107418		0.42	0.02991	1.52413	0.58330986	0.472123702	0.111
cg10783469	I	37	19	52391234	ZNF577;ZNF577;ZN	0.42	0.02991	1.52413	0.29551842	0.407004137	-0.111
cg01477133	II	37	20	33878142	FAM83C	0.5	0.00432	2.36443	0.66552734	0.554550455	0.111
cg20752831	II	37	20	43382731	RIMS4	0.46	0.01197	1.92207	0.74112458	0.630310188	0.111
cg13209907	II	37	20	44260207	WFDC9	0.42	0.02991	1.52413	0.64803541	0.536584378	0.111
cg17986701	II	37	20	44574422	PCIF1	0.71	4.57E-06	5.34042	0.64900524	0.760161506	-0.111
cg17283039	I	37	22	29787625		0.58	0.00041	3.39184	0.75671511	0.867524752	-0.111
cg15868105	II	37	22	30116953	CABP7	0.67	2.34E-05	4.63072	0.15452654	0.265828825	-0.111
cg01949002	II	37	1	2203589	SKI	0.62	0.0001	3.98291	0.69391896	0.804175134	-0.11
cg23666378	I	37	1	3663164	KIAA0495;KIAA049!	0.58	0.00041	3.39184	0.44756876	0.337248552	0.11
cg08926642	I	37	1	7887455	PER3	0.46	0.01197	1.92207	0.90523873	0.795242676	0.11
cg23228178	II	37	1	17633681	PADI4	0.46	0.01197	1.92207	0.59223625	0.482030374	0.11
cg18196063	II	37	1	24652248	GRHL3;GRHL3;GRH	0.5	0.00432	2.36443	0.64992099	0.759462471	-0.11
cg16417374	II	37	1	26098310	MAN1C1	0.58	0.00041	3.39184	0.48018132	0.370484896	0.11
cg01022916	II	37	1	31196938	MATN1	0.5	0.00432	2.36443	0.61887392	0.728505032	-0.11
cg26301690	II	37	1	50569610	ELAVL4;ELAVL4;ELA	0.62	0.0001	3.98291	0.81612597	0.706089807	0.11
cg10541466	II	37	1	113425263		0.54	0.0014	2.85345	0.2613732	0.371036299	-0.11
cg22424746	II	37	1	117753313	VTCN1	0.5	0.00432	2.36443	0.73958838	0.630034857	0.11
cg01076495	II	37	1	150480588	ECM1;ECM1;ECM1;	0.42	0.02991	1.52413	0.41381322	0.524194571	-0.11
cg20002843	II	37	1	204338373	LOC127841	0.58	0.00041	3.39184	0.36112742	0.471201936	-0.11
cg20680802	II	37	1	226299139		0.54	0.0014	2.85345	0.31045976	0.200320604	0.11
cg11915812	II	37	2	10554240	HPCAL1;HPCAL1	0.5	0.00432	2.36443	0.70166972	0.591798959	0.11
cg03514843	II	37	2	27301195	EMILIN1	0.67	2.34E-05	4.63072	0.60613678	0.716477778	-0.11
cg14628982	II	37	2	28717630	PLB1;PLB1	0.54	0.0014	2.85345	0.8161954	0.706014417	0.11
cg10954469	I	37	2	71115370		0.46	0.01197	1.92207	0.09630327	0.206431214	-0.11

cg05036173	II	37	2	108994528	SULT1C4;SULT1C4	0.58	0.00041	3.39184	0.16598016	0.275703551	-0.11
cg14966782	II	37	2	109812089	SH3RF3	0.46	0.01197	1.92207	0.79437633	0.684575628	0.11
cg08570243	II	37	2	110969853	NCRNA00116	0.46	0.01197	1.92207	0.20064209	0.090500878	0.11
cg14001664	I	37	2	132088801		0.46	0.01197	1.92207	0.12493955	0.23497203	-0.11
cg19275653	II	37	2	175532338	WIPF1	0.5	0.00432	2.36443	0.23350275	0.343787276	-0.11
cg12001304	II	37	2	229045020	SPHKAP;SPHKAP	0.46	0.01197	1.92207	0.16992972	0.279494333	-0.11
cg18267381	II	37	3	21792434	ZNF385D;ZNF385D	0.46	0.01197	1.92207	0.19192799	0.301813479	-0.11
cg27588384	II	37	3	45674281	LIMD1	0.42	0.02991	1.52413	0.66830084	0.778780594	-0.11
cg18644653	II	37	3	49696053	BSN	0.54	0.0014	2.85345	0.51785706	0.627966832	-0.11
cg18094261	II	37	3	52216318		0.5	0.00432	2.36443	0.55442211	0.663937532	-0.11
cg01454951	II	37	3	71730677	EIF4E3;EIF4E3;EIF4I	0.42	0.02991	1.52413	0.2247531	0.334260658	-0.11
cg12807187	II	37	3	71778392	EIF4E3;EIF4E3;EIF4I	0.5	0.00432	2.36443	0.59606517	0.705745353	-0.11
cg00466268	II	37	3	164914621	SLITRK3	0.46	0.01197	1.92207	0.17233868	0.282676264	-0.11
cg18171097	I	37	3	169532134		0.54	0.0014	2.85345	0.88397188	0.774387813	0.11
cg24671330	II	37	3	193066097	ATP13A5	0.46	0.01197	1.92207	0.66318049	0.553199133	0.11
cg03567896	II	37	4	7067375	GRPEL1	0.62	0.0001	3.98291	0.76101586	0.871023781	-0.11
cg03063309	II	37	4	24796919	SOD3	0.58	0.00041	3.39184	0.77526929	0.665584588	0.11
cg10227863	II	37	4	55120729	PDGFRA	0.5	0.00432	2.36443	0.73479784	0.624624543	0.11
cg01578875	I	37	4	146804010	ZNF827	0.54	0.0014	2.85345	0.8382896	0.728745878	0.11
cg00499700	II	37	5	76116088	F2RL1	0.46	0.01197	1.92207	0.28950313	0.399208746	-0.11
cg00840960	II	37	5	148034030	HTR4;HTR4;HTR4;H	0.42	0.02991	1.52413	0.16494865	0.275118288	-0.11
cg16646054	II	37	5	150157726	C5orf62	0.42	0.02991	1.52413	0.420409	0.530706266	-0.11
cg01603073	I	37	5	176101928		0.54	0.0014	2.85345	0.6655445	0.555158821	0.11
cg00471059	II	37	5	179562620	RASGEF1C	0.54	0.0014	2.85345	0.59425447	0.703877753	-0.11
cg02049405	I	37	6	30095265		0.42	0.02991	1.52413	0.5830787	0.47318748	0.11
cg07981266	II	37	6	30720311		0.46	0.01197	1.92207	0.18064219	0.290655263	-0.11
cg20371401	I	37	6	31895493	C2;C2;C2;C2	0.58	0.00041	3.39184	0.64565654	0.755817706	-0.11
cg23339482	II	37	6	32036278	TNXB	0.54	0.0014	2.85345	0.7143397	0.604786265	0.11
cg25636481	II	37	6	33241410	RPS18	0.42	0.02991	1.52413	0.7569872	0.646547309	0.11
cg09673807	II	37	6	33575719		0.42	0.02991	1.52413	0.64699411	0.536520997	0.11
cg12008034	II	37	6	33996580	GRM4	0.54	0.0014	2.85345	0.61964502	0.509895346	0.11
cg11870042	II	37	7	2060040	MAD1L1;MAD1L1;M	0.58	0.00041	3.39184	0.45839032	0.568131296	-0.11
cg20336172	I	37	7	2773782	GNA12	0.5	0.00432	2.36443	0.30966514	0.419989341	-0.11

cg06896909	II	37	7	19813297	TMEM196	0.42	0.02991	1.52413	0.30587836	0.415723883	-0.11
cg06496272	II	37	7	22895283	SNORD93	0.5	0.00432	2.36443	0.82585498	0.715727302	0.11
cg02389949	II	37	7	30176264	C7orf41	0.54	0.0014	2.85345	0.29567698	0.40587629	-0.11
cg12240237	II	37	7	73946402	GTF2IRD1;GTF2IRD	0.42	0.02991	1.52413	0.80514439	0.695511381	0.11
cg07719679	II	37	7	87936392	STEAP4	0.54	0.0014	2.85345	0.18702273	0.296786899	-0.11
cg02327530	II	37	7	100091786	C7orf51	0.5	0.00432	2.36443	0.24309924	0.35335783	-0.11
cg07428004	II	37	7	127912372		0.54	0.0014	2.85345	0.44880156	0.558344678	-0.11
cg23299919	I	37	7	157406096	PTPRN2;PTPRN2;PT	0.42	0.02991	1.52413	0.26074221	0.37097743	-0.11
cg07461572	II	37	8	1955117		0.5	0.00432	2.36443	0.71943758	0.829189528	-0.11
cg11919837	II	37	8	57350735		0.42	0.02991	1.52413	0.6266168	0.736233543	-0.11
cg06617456	II	37	8	68864769	PREX2;PREX2	0.54	0.0014	2.85345	0.08593606	0.195931969	-0.11
cg21399717	II	37	8	102263157		0.42	0.02991	1.52413	0.3851581	0.275425195	0.11
cg19352830	II	37	8	141599356	EIF2C2;EIF2C2	0.54	0.0014	2.85345	0.70926053	0.599001298	0.11
cg13985784	I	37	9	25678015	TUSC1	0.5	0.00432	2.36443	0.03859105	0.148306652	-0.11
cg05261759	II	37	9	130498309	TOR2A;TOR2A;TOR	0.5	0.00432	2.36443	0.7288412	0.838490159	-0.11
cg00698575	II	37	9	140388604	PNPLA7;PNPLA7	0.58	0.00041	3.39184	0.60404558	0.714457731	-0.11
cg25614935	I	37	10	1084866	C10orf110;C10orf1	0.58	0.00041	3.39184	0.88663334	0.777081225	0.11
cg09105442	I	37	10	1228439	ADARB2	0.62	0.0001	3.98291	0.84912945	0.738749201	0.11
cg09684112	I	37	10	13701447	FRMD4A	0.58	0.00041	3.39184	0.13945789	0.249648431	-0.11
cg11043993	II	37	10	50536966		0.46	0.01197	1.92207	0.74415947	0.633939628	0.11
cg07545081	II	37	10	126308381	FAM53B	0.46	0.01197	1.92207	0.66214118	0.552097393	0.11
cg05098512	I	37	10	134861083		0.54	0.0014	2.85345	0.86647746	0.756612797	0.11
cg16638248	II	37	11	10596059	MRVI1;MRVI1;MRV	0.42	0.02991	1.52413	0.73538585	0.625032827	0.11
cg24453664	II	37	11	33758413	CD59;CD59;CD59;C	0.42	0.02991	1.52413	0.21724933	0.326807899	-0.11
cg09554856	II	37	11	33914646	LMO2	0.42	0.02991	1.52413	0.24828867	0.358293776	-0.11
cg17338544	II	37	11	44559989		0.54	0.0014	2.85345	0.54203409	0.65160219	-0.11
cg10460350	II	37	11	60658361	PRPF19	0.54	0.0014	2.85345	0.35466273	0.465083648	-0.11
cg14654385	II	37	11	63973006	FERMT3;FERMT3	0.54	0.0014	2.85345	0.36641494	0.476867936	-0.11
cg22330021	II	37	11	64372277		0.46	0.01197	1.92207	0.73733178	0.627134015	0.11
cg00509451	I	37	11	64811775	SAC3D1	0.58	0.00041	3.39184	0.82773403	0.93726647	-0.11
cg01522592	II	37	11	75235311	GDPD5	0.46	0.01197	1.92207	0.59528994	0.484961415	0.11
cg07237926	II	37	11	117857630	IL10RA;IL10RA	0.58	0.00041	3.39184	0.51386559	0.404206565	0.11
cg14081270	II	37	11	118286105		0.46	0.01197	1.92207	0.24371584	0.354051003	-0.11

cg04176122	II	37	11	118779835	BCL9L	0.54	0.0014	2.85345	0.54133527	0.431069828	0.11
cg20306694	II	37	12	51718251	BIN2	0.42	0.02991	1.52413	0.43413179	0.543781895	-0.11
cg23060047	II	37	12	130819504		0.5	0.00432	2.36443	0.33352507	0.443662961	-0.11
cg24425149	II	37	13	75991251	TBC1D4	0.54	0.0014	2.85345	0.33750473	0.447599298	-0.11
cg00046899	II	37	13	97777690		0.46	0.01197	1.92207	0.56710904	0.456906035	0.11
cg03950873	II	37	14	60045040	C14orf38	0.5	0.00432	2.36443	0.80677664	0.696662195	0.11
cg05062889	II	37	16	687414	C16orf13;C16orf13	0.5	0.00432	2.36443	0.16597605	0.276164627	-0.11
cg05895403	II	37	16	31150380	PRSS36	0.54	0.0014	2.85345	0.58380174	0.694258122	-0.11
cg07223266	II	37	16	33961872		0.62	0.0001	3.98291	0.44614574	0.556090578	-0.11
cg06256596	II	37	16	66511944	BEAN	0.5	0.00432	2.36443	0.43295978	0.322795727	0.11
cg07285481	II	37	16	81667167	CMIP;CMIP	0.58	0.00041	3.39184	0.66237752	0.772254331	-0.11
cg03834411	I	37	16	87682036	JPH3	0.42	0.02991	1.52413	0.79588897	0.685773032	0.11
cg07983907	I	37	16	88697947	ZC3H18	0.58	0.00041	3.39184	0.6500288	0.760163458	-0.11
cg06419432	II	37	17	25707535		0.42	0.02991	1.52413	0.20039019	0.310021619	-0.11
cg13487284	II	37	17	26794878		0.62	0.0001	3.98291	0.44420269	0.554436339	-0.11
cg11826961	II	37	17	38221639	THRA;THRA	0.71	4.57E-06	5.34042	0.5168505	0.627251748	-0.11
cg01512089	II	37	17	41705043		0.58	0.00041	3.39184	0.44575013	0.556150347	-0.11
cg03661110	II	37	17	48581622		0.5	0.00432	2.36443	0.29875367	0.409204863	-0.11
cg08034797	II	37	17	75306498	SEPT9;SEPT9	0.62	0.0001	3.98291	0.84456536	0.734662451	0.11
cg02802072	II	37	17	79229124	SLC38A10;SLC38A1	0.5	0.00432	2.36443	0.36290974	0.473148795	-0.11
cg17616192	II	37	17	80009015	GPS1;RFNG;GPS1	0.67	2.34E-05	4.63072	0.60396456	0.714183011	-0.11
cg12549345	II	37	19	2789453	THOP1	0.46	0.01197	1.92207	0.7135368	0.603615611	0.11
cg01032675	I	37	19	3136430	GNA15;GNA15	0.42	0.02991	1.52413	0.11180519	0.22174284	-0.11
cg07685563	II	37	19	4954869	UHRF1;UHRF1	0.58	0.00041	3.39184	0.74593425	0.855968254	-0.11
cg02577963	I	37	19	5041570	KDM4B	0.54	0.0014	2.85345	0.7987819	0.908679393	-0.11
cg24911113	I	37	19	23185714		0.42	0.02991	1.52413	0.31942955	0.429911945	-0.11
cg25717032	II	37	19	35981549	KRTDAP	0.46	0.01197	1.92207	0.37078686	0.481060333	-0.11
cg18788725	II	37	19	45512122	RELB	0.46	0.01197	1.92207	0.6801198	0.789853197	-0.11
cg22027879	II	37	19	49469965	FTL	0.5	0.00432	2.36443	0.76771245	0.877860263	-0.11
cg08318732	II	37	19	53192577	ZNF83	0.62	0.0001	3.98291	0.5249892	0.635059256	-0.11
cg20038038	I	37	19	54804217	LILRA3;LILRA3	0.5	0.00432	2.36443	0.4798403	0.589569327	-0.11
cg12338552	II	37	20	690915		0.42	0.02991	1.52413	0.19132583	0.301360107	-0.11
cg07676859	I	37	20	23015932	SSTR4	0.46	0.01197	1.92207	0.21317459	0.322929519	-0.11

cg01371631	II	37	20	57267176	NPEPL1	0.67	2.34E-05	4.63072	0.64716886	0.757333522	-0.11
cg00673191	II	37	21	37536923	DOPEY2	0.54	0.0014	2.85345	0.70308931	0.81268621	-0.11
cg05949181	II	37	22	39154591		0.67	2.34E-05	4.63072	0.79831993	0.68818847	0.11
cg15911859	I	37	22	45810043	RIBC2;SMC1B	0.5	0.00432	2.36443	0.17572019	0.286105636	-0.11
cg05392448	I	37	1	2266933	MORN1	0.67	2.34E-05	4.63072	0.91427673	0.804849005	0.109
cg11226808	II	37	1	3581320	TP73	0.46	0.01197	1.92207	0.75775351	0.64826027	0.109
cg00534163	II	37	1	3740120	KIAA0562	0.46	0.01197	1.92207	0.77289711	0.882072854	-0.109
cg04438595	I	37	1	16176425	SPEN	0.46	0.01197	1.92207	0.35910968	0.250602411	0.109
cg27513684	I	37	1	27729053		0.46	0.01197	1.92207	0.29774009	0.406474278	-0.109
cg07570723	II	37	1	39875238	KIAA0754;KIAA075	0.46	0.01197	1.92207	0.17560054	0.284475465	-0.109
cg04718492	II	37	1	117753741	VTCN1	0.58	0.00041	3.39184	0.80054629	0.691479087	0.109
cg18002437	II	37	1	144522237	LOC728875	0.58	0.00041	3.39184	0.60982368	0.500392003	0.109
cg02459569	II	37	1	150944634	LASS2;LASS2	0.58	0.00041	3.39184	0.60584199	0.714922398	-0.109
cg15690542	II	37	1	151172905	PIP5K1A;PIP5K1A;P	0.54	0.0014	2.85345	0.68117278	0.572364044	0.109
cg12473916	II	37	1	154943651	SHC1;SHC1;SHC1;SI	0.54	0.0014	2.85345	0.28884169	0.397541695	-0.109
cg15487646	II	37	1	181077141		0.46	0.01197	1.92207	0.5949666	0.485547851	0.109
cg17954152	II	37	1	214160860	PROX1	0.67	2.34E-05	4.63072	0.08806765	0.196797821	-0.109
cg09699787	II	37	2	1711759	PXDN	0.42	0.02991	1.52413	0.31548531	0.424174252	-0.109
cg21484315	II	37	2	2712669		0.42	0.02991	1.52413	0.73098869	0.621785317	0.109
cg23422170	II	37	2	10345003	C2orf48	0.67	2.34E-05	4.63072	0.64253173	0.751788637	-0.109
cg12655542	II	37	2	20357306		0.46	0.01197	1.92207	0.26949249	0.378345776	-0.109
cg06481517	II	37	2	49084769		0.54	0.0014	2.85345	0.65578051	0.546349162	0.109
cg09404516	II	37	2	54832147	SPTBN1;SPTBN1	0.54	0.0014	2.85345	0.21343679	0.322674954	-0.109
cg20175390	II	37	2	119599459		0.5	0.00432	2.36443	0.20281071	0.311390415	-0.109
cg24544177	II	37	2	237771837		0.5	0.00432	2.36443	0.79482799	0.686113666	0.109
cg14208102	II	37	3	48507165	TREX1;TREX1	0.54	0.0014	2.85345	0.19191579	0.301128129	-0.109
cg14852276	II	37	3	197684729	IQCG	0.54	0.0014	2.85345	0.8062539	0.915526922	-0.109
cg20092199	II	37	4	1342459	KIAA1530	0.46	0.01197	1.92207	0.65370557	0.762318442	-0.109
cg14882700	I	37	4	4228571	OTOP1	0.46	0.01197	1.92207	0.23962819	0.348604445	-0.109
cg26296488	I	37	4	9783192	DRD5	0.42	0.02991	1.52413	0.27306643	0.382125147	-0.109
cg20971045	II	37	5	53075687		0.54	0.0014	2.85345	0.25287332	0.361699961	-0.109
cg20536364	II	37	5	55790102		0.46	0.01197	1.92207	0.65686767	0.76627031	-0.109
cg15538767	II	37	5	149870395		0.46	0.01197	1.92207	0.438731	0.547917162	-0.109

cg00059225	II	37	5	151304357	GLRA1;GLRA1;GLRA	0.62	0.0001	3.98291	0.36082643	0.470324098	-0.109
cg22570970	I	37	6	15401067	JARID2	0.54	0.0014	2.85345	0.34033625	0.449590839	-0.109
cg26888012	II	37	6	31091383	PSORS1C1	0.42	0.02991	1.52413	0.7230028	0.614457902	0.109
cg19367859	II	37	6	31529897		0.46	0.01197	1.92207	0.69496849	0.586026057	0.109
cg14324675	II	37	6	31554848	LST1;LST1;LST1;LST	0.54	0.0014	2.85345	0.26762229	0.37678452	-0.109
cg04567302	II	37	6	31846956	SLC44A4	0.54	0.0014	2.85345	0.6526586	0.761807727	-0.109
cg21232620	II	37	6	78172192	HTR1B	0.54	0.0014	2.85345	0.24125746	0.349907594	-0.109
cg02461269	II	37	6	156509874		0.42	0.02991	1.52413	0.31239135	0.421196878	-0.109
cg16298927	II	37	6	170191076	C6orf122;C6orf208	0.54	0.0014	2.85345	0.63935772	0.748500879	-0.109
cg04109092	I	37	7	2647852	IQCE;IQCE	0.58	0.00041	3.39184	0.48203729	0.59058587	-0.109
cg13034073	II	37	7	2769681	GNA12	0.58	0.00041	3.39184	0.38888203	0.498282841	-0.109
cg09832443	II	37	7	28612680	CREB5;CREB5;CREB	0.5	0.00432	2.36443	0.74420985	0.635542881	0.109
cg00486143	II	37	7	127774154		0.46	0.01197	1.92207	0.30391026	0.413398089	-0.109
cg16456596	II	37	8	8313504		0.5	0.00432	2.36443	0.58312747	0.692297813	-0.109
cg21163717	I	37	8	21769903	DOK2	0.42	0.02991	1.52413	0.75098981	0.641755818	0.109
cg16142218	II	37	8	23101221	CHMP7;CHMP7	0.5	0.00432	2.36443	0.31384067	0.204808568	0.109
cg03922423	II	37	8	144408588	TOP1MT	0.67	2.34E-05	4.63072	0.43515754	0.543829885	-0.109
cg22082046	II	37	9	19934447		0.42	0.02991	1.52413	0.21288483	0.321437417	-0.109
cg13842639	I	37	9	38488083		0.42	0.02991	1.52413	0.69087214	0.800139887	-0.109
cg16112945	II	37	9	136285806	ADAMTS13;ADAMT	0.42	0.02991	1.52413	0.61521411	0.505888472	0.109
cg17112382	II	37	10	21676930		0.58	0.00041	3.39184	0.80412879	0.695477715	0.109
cg24445116	II	37	10	48523589		0.46	0.01197	1.92207	0.78984292	0.680602023	0.109
cg13344587	II	37	10	63723919	ARID5B	0.46	0.01197	1.92207	0.54745856	0.438328089	0.109
cg12147622	II	37	10	74021432		0.5	0.00432	2.36443	0.60041831	0.491597541	0.109
cg14568203	II	37	10	79931578		0.46	0.01197	1.92207	0.78273663	0.674083677	0.109
cg24168991	II	37	10	106078922	ITPRIP	0.54	0.0014	2.85345	0.77344673	0.882193785	-0.109
cg03510732	II	37	10	114871931	TCF7L2;TCF7L2;TCF	0.46	0.01197	1.92207	0.83920169	0.729988132	0.109
cg10858945	II	37	10	116528318		0.58	0.00041	3.39184	0.2158781	0.324446978	-0.109
cg01160452	II	37	10	118957098	KCNK18	0.46	0.01197	1.92207	0.69180157	0.582644014	0.109
cg02695567	I	37	10	134728488		0.46	0.01197	1.92207	0.56336833	0.453878342	0.109
cg07047068	II	37	11	844686	TSPAN4;TSPAN4;TS	0.54	0.0014	2.85345	0.18110023	0.289660177	-0.109
cg18311341	II	37	11	7693674	CYB5R2	0.42	0.02991	1.52413	0.8385034	0.729582924	0.109
cg23195547	II	37	11	8740646	ST5;ST5;ST5	0.5	0.00432	2.36443	0.67636576	0.785515972	-0.109

cg11986385	II	37	11	19628828	NAV2	0.5	0.00432	2.36443	0.70962843	0.600490718	0.109
cg13974632	I	37	11	27740813	BDNF;BDNF;BDNF;E	0.46	0.01197	1.92207	0.1509558	0.259877012	-0.109
cg03732020	I	37	11	47282968	NR1H3;NR1H3;NR1	0.5	0.00432	2.36443	0.59752009	0.706118009	-0.109
cg06917325	II	37	11	62783123	SLC22A8	0.54	0.0014	2.85345	0.83642894	0.727905926	0.109
cg20930290	II	37	11	64138764	RPS6KA4;RPS6KA4	0.54	0.0014	2.85345	0.40432579	0.513051045	-0.109
cg09982224	II	37	11	67775972	ALDH3B1	0.46	0.01197	1.92207	0.35364595	0.463078635	-0.109
cg23423933	II	37	11	70269613	CTTN;CTTN	0.42	0.02991	1.52413	0.7730414	0.66407527	0.109
cg03780927	I	37	11	118781150	BCL9L;BCL9L	0.42	0.02991	1.52413	0.31610244	0.425397112	-0.109
cg09971562	II	37	11	128694679		0.42	0.02991	1.52413	0.48944346	0.59853986	-0.109
cg23378074	I	37	12	30947871		0.5	0.00432	2.36443	0.70794065	0.817008667	-0.109
cg23126152	II	37	12	109221407	SSH1;SSH1;SSH1	0.54	0.0014	2.85345	0.66343761	0.772930757	-0.109
cg05939560	II	37	12	131003775	RIMBP2	0.54	0.0014	2.85345	0.4930074	0.384037757	0.109
cg01692110	II	37	13	113365389	ATP11A;ATP11A	0.42	0.02991	1.52413	0.75210248	0.643067976	0.109
cg23620184	II	37	14	23772616	PPP1R3E	0.46	0.01197	1.92207	0.72698083	0.617566759	0.109
cg13803234	II	37	14	68830813	RAD51L1;RAD51L1;	0.62	0.0001	3.98291	0.50910425	0.617698134	-0.109
cg04738877	II	37	14	88459963	GALC;GALC	0.54	0.0014	2.85345	0.88901661	0.780138144	0.109
cg04189326	II	37	14	103481271	CDC42BPB	0.5	0.00432	2.36443	0.37503142	0.484382483	-0.109
cg16420200	I	37	14	106173779		0.42	0.02991	1.52413	0.85321037	0.743960888	0.109
cg00493389	II	37	15	41050377		0.5	0.00432	2.36443	0.74372486	0.635116996	0.109
cg09555736	II	37	16	474271	RAB11FIP3	0.46	0.01197	1.92207	0.65205152	0.761440683	-0.109
cg00472758	II	37	16	2552820	TBC1D24	0.5	0.00432	2.36443	0.70806203	0.817308849	-0.109
cg04735948	II	37	16	4661664	FAM100A	0.58	0.00041	3.39184	0.6127465	0.72191435	-0.109
cg26709300	II	37	16	30106682	YPEL3;YPEL3	0.62	0.0001	3.98291	0.52930536	0.419810327	0.109
cg00596184	II	37	16	69350052	VPS4A	0.46	0.01197	1.92207	0.71467466	0.823853063	-0.109
cg05830220	II	37	16	87757033	KLHDC4	0.42	0.02991	1.52413	0.71085092	0.601765088	0.109
cg04252928	II	37	16	89168599	ACSF3;ACSF3;ACSF3	0.54	0.0014	2.85345	0.61629742	0.724983382	-0.109
cg01097406	II	37	16	89675127		0.42	0.02991	1.52413	0.40765098	0.516823716	-0.109
cg09464268	II	37	17	32266	DOC2B	0.67	2.34E-05	4.63072	0.64091986	0.750197056	-0.109
cg01438467	I	37	17	1510080	SLC43A2	0.42	0.02991	1.52413	0.84012045	0.730623867	0.109
cg12065777	II	37	17	16311838		0.46	0.01197	1.92207	0.34416869	0.453063804	-0.109
cg15269394	II	37	17	40672083	ATP6V0A1;ATP6V0A	0.5	0.00432	2.36443	0.69229792	0.801168916	-0.109
cg20810975	II	37	17	42467199	ITGA2B	0.54	0.0014	2.85345	0.25373678	0.362812371	-0.109
cg12223258	II	37	17	43117026	DCAKD;DCAKD	0.5	0.00432	2.36443	0.53073561	0.422133211	0.109

cg14154330	II	37	17	43503401	ARHGAP27	0.67	2.34E-05	4.63072	0.53714099	0.645766843	-0.109
cg24958366	II	37	17	46952555		0.46	0.01197	1.92207	0.29988356	0.409094913	-0.109
cg27210390	II	37	17	52978583	TOM1L1	0.54	0.0014	2.85345	0.46450034	0.573824245	-0.109
cg03554335	II	37	17	62075151	C17orf72	0.58	0.00041	3.39184	0.43088093	0.540109401	-0.109
cg21441211	II	37	17	71313862		0.46	0.01197	1.92207	0.81110649	0.702396765	0.109
cg05727573	I	37	17	74557104	SNORD1A;SNORD1I	0.46	0.01197	1.92207	0.34514284	0.454504424	-0.109
cg22669656	II	37	17	76386825	PGS1	0.46	0.01197	1.92207	0.70471107	0.596202591	0.109
cg01681525	II	37	17	79374741	BAHCC1	0.54	0.0014	2.85345	0.5021829	0.611154935	-0.109
cg26828842	II	37	18	67952086		0.42	0.02991	1.52413	0.43710592	0.546248557	-0.109
cg01963696	II	37	19	851650	ELANE	0.5	0.00432	2.36443	0.62876546	0.738067705	-0.109
cg22900607	I	37	19	2546938	GNG7	0.5	0.00432	2.36443	0.75505727	0.864539143	-0.109
cg16242615	II	37	19	4059988	ZBTB7A	0.42	0.02991	1.52413	0.43400573	0.542640284	-0.109
cg07375851	II	37	19	15591424	PGLYRP2	0.42	0.02991	1.52413	0.76204752	0.653312635	0.109
cg01442620	II	37	19	33162885	ANKRD27	0.67	2.34E-05	4.63072	0.16441149	0.273605822	-0.109
cg27087650	II	37	19	45255796	BCL3	0.46	0.01197	1.92207	0.22334019	0.114054474	0.109
cg21456083	II	37	19	49524256		0.54	0.0014	2.85345	0.30132563	0.192144653	0.109
cg06538141	II	37	20	30229335	COX4I2	0.42	0.02991	1.52413	0.73405249	0.62516928	0.109
cg17775490	II	37	20	45179354	C20orf123	0.42	0.02991	1.52413	0.19873948	0.308189831	-0.109
cg14172108	I	37	21	34405553		0.58	0.00041	3.39184	0.5593371	0.450648441	0.109
cg03392571	II	37	21	46367939	C21orf70	0.54	0.0014	2.85345	0.63891773	0.747562701	-0.109
cg07685786	II	37	21	48025691	S100B	0.46	0.01197	1.92207	0.58928275	0.69801605	-0.109
cg26986147	II	37	22	30957711	GAL3ST1	0.58	0.00041	3.39184	0.42630801	0.535565504	-0.109
cg20330126	II	37	22	36125053	APOL5	0.5	0.00432	2.36443	0.68854498	0.579074609	0.109
cg01285652	II	37	22	50902555	SBF1	0.42	0.02991	1.52413	0.69402987	0.585445208	0.109
cg04562757	II	37	1	17882998	ARHGEF10L	0.5	0.00432	2.36443	0.74455359	0.636923069	0.108
cg27552711	I	37	1	27332282	FAM46B	0.58	0.00041	3.39184	0.72571362	0.833934694	-0.108
cg11529819	II	37	1	27695677	FCN3;FCN3	0.58	0.00041	3.39184	0.29245136	0.400179222	-0.108
cg08857221	II	37	1	37941361	ZC3H12A	0.5	0.00432	2.36443	0.58857104	0.480843062	0.108
cg00778190	II	37	1	95068029		0.5	0.00432	2.36443	0.16101445	0.269407816	-0.108
cg07906625	II	37	1	145021703	PDE4DIP	0.46	0.01197	1.92207	0.32868756	0.437048167	-0.108
cg00006397	II	37	1	160336699	NHLH1	0.46	0.01197	1.92207	0.75121579	0.643290631	0.108
cg14031054	II	37	1	164681652	PBX1	0.58	0.00041	3.39184	0.26407247	0.371729668	-0.108
cg04391048	II	37	2	18766022	NT5C1B;NT5C1B	0.46	0.01197	1.92207	0.56508518	0.673535253	-0.108

cg23401251	II	37	2	27683905	IFT172	0.5	0.00432	2.36443	0.65480236	0.762883124	-0.108
cg02646394	I	37	2	89065379	FLJ40330	0.62	0.0001	3.98291	0.38900275	0.280746261	0.108
cg00790071	II	37	2	102731407		0.62	0.0001	3.98291	0.52826836	0.635849229	-0.108
cg02256105	II	37	2	118593478		0.42	0.02991	1.52413	0.53928835	0.647603872	-0.108
cg01420564	II	37	2	179076804	OSBPL6	0.58	0.00041	3.39184	0.76350763	0.655646241	0.108
cg02234120	II	37	2	234626351	UGT1A10;UGT1A6;	0.5	0.00432	2.36443	0.81671685	0.708728097	0.108
cg16869108	II	37	3	10184319	VHL;VHL	0.46	0.01197	1.92207	0.76430057	0.656163357	0.108
cg03565274	II	37	3	50629708		0.5	0.00432	2.36443	0.45191112	0.559744899	-0.108
cg11663376	II	37	3	147089629		0.54	0.0014	2.85345	0.29752381	0.405172822	-0.108
cg09122442	II	37	4	6894410		0.54	0.0014	2.85345	0.3784146	0.486351922	-0.108
cg12114584	II	37	4	54518744		0.5	0.00432	2.36443	0.76033123	0.652634876	0.108
cg12307314	II	37	5	142206724	ARHGAP26;ARHGAI	0.5	0.00432	2.36443	0.30531131	0.413667363	-0.108
cg25583651	II	37	6	29570040	GABBR1;GABBR1;G	0.5	0.00432	2.36443	0.24782338	0.355953659	-0.108
cg22425467	II	37	6	30131189	TRIM15;TRIM15	0.46	0.01197	1.92207	0.47446018	0.366514387	0.108
cg23163653	II	37	6	30565385		0.46	0.01197	1.92207	0.5119745	0.404214767	0.108
cg02989255	II	37	6	32063774	TNXB	0.42	0.02991	1.52413	0.55546493	0.663793202	-0.108
cg16634404	II	37	6	33161438	COL11A2;RXRB;COI	0.5	0.00432	2.36443	0.57880641	0.686503679	-0.108
cg26500914	I	37	6	33288323	DAXX;DAXX;DAXX;D	0.58	0.00041	3.39184	0.81459444	0.92256467	-0.108
cg14302083	II	37	6	43608326	MAD2L1BP;MAD2L	0.54	0.0014	2.85345	0.79465194	0.902195302	-0.108
cg00073460	II	37	6	149806502	ZC3H12D	0.5	0.00432	2.36443	0.45232261	0.344479396	0.108
cg18319852	I	37	6	168436099	KIF25;KIF25	0.46	0.01197	1.92207	0.32974681	0.43788735	-0.108
cg21495349	II	37	7	1081344	C7orf50;C7orf50;C7	0.46	0.01197	1.92207	0.54818589	0.656059327	-0.108
cg00624477	II	37	7	1406560		0.46	0.01197	1.92207	0.57985397	0.471626376	0.108
cg02613295	II	37	7	2564256	LFNG;LFNG;LFNG;LI	0.5	0.00432	2.36443	0.67248396	0.564711506	0.108
cg05774699	II	37	7	27184316	HOXA5	0.58	0.00041	3.39184	0.67394008	0.782140148	-0.108
cg24471254	I	37	7	100253792	ACTL6B	0.54	0.0014	2.85345	0.64963182	0.758070976	-0.108
cg11111139	II	37	7	157620685	PTPRN2;PTPRN2;PT	0.46	0.01197	1.92207	0.48912857	0.596781461	-0.108
cg11313780	II	37	8	103817950		0.54	0.0014	2.85345	0.64940795	0.75769621	-0.108
cg22284058	II	37	8	142237359	SLC45A4	0.58	0.00041	3.39184	0.54212784	0.64990289	-0.108
cg04064588	II	37	8	144945619	EPPK1	0.42	0.02991	1.52413	0.61845114	0.510640038	0.108
cg07672814	II	37	9	140356830	PNPLA7;PNPLA7	0.42	0.02991	1.52413	0.27056009	0.163012187	0.108
cg17319294	II	37	10	71588009	COL13A1;COL13A1;	0.46	0.01197	1.92207	0.41886399	0.310605779	0.108
cg14530290	II	37	10	80853985	ZMIZ1	0.46	0.01197	1.92207	0.19541441	0.303772207	-0.108

cg11944139	I	37	10	133957543	JAKMIP3	0.46	0.01197	1.92207	0.51705159	0.408622483	0.108
cg05493509	II	37	10	134041783	STK32C	0.5	0.00432	2.36443	0.75160137	0.643844016	0.108
cg26376030	I	37	11	646373	DEAF1	0.46	0.01197	1.92207	0.50480809	0.396786737	0.108
cg05178291	II	37	11	18287689	SAA1;SAA1	0.54	0.0014	2.85345	0.21491204	0.322690343	-0.108
cg15060115	II	37	11	45672248	CHST1	0.58	0.00041	3.39184	0.69297841	0.801188018	-0.108
cg16004377	II	37	11	63272909	LGALS12;LGALS12;l	0.46	0.01197	1.92207	0.28396912	0.391890535	-0.108
cg22933847	II	37	11	68780136	MRGPRF;MRGPRF	0.5	0.00432	2.36443	0.27692294	0.384562713	-0.108
cg03958883	II	37	11	73020729	ARHGEF17	0.58	0.00041	3.39184	0.44829083	0.556740055	-0.108
cg23189044	II	37	11	74178114	KCNE3	0.54	0.0014	2.85345	0.21886049	0.32707611	-0.108
cg09692396	I	37	12	7023346	LRRC23;ENO2;LRRC	0.58	0.00041	3.39184	0.40220429	0.293720371	0.108
cg13175739	II	37	12	10789214	STYK1	0.5	0.00432	2.36443	0.635823	0.744120847	-0.108
cg10923838	II	37	12	120242330	CIT	0.46	0.01197	1.92207	0.34454157	0.452389833	-0.108
cg14256511	II	37	12	123347684		0.42	0.02991	1.52413	0.78339639	0.675057763	0.108
cg09967479	II	37	12	133010684		0.46	0.01197	1.92207	0.49491367	0.38711536	0.108
cg17903776	II	37	13	61989726	PCDH20	0.54	0.0014	2.85345	0.16211616	0.269637789	-0.108
cg25320816	II	37	13	114099871	ADPRHL1;ADPRHL1	0.46	0.01197	1.92207	0.67900582	0.571404641	0.108
cg08198540	I	37	14	22963867		0.5	0.00432	2.36443	0.85940446	0.751433647	0.108
cg12142424	II	37	14	94759873	SERPINA10;SERPIN/	0.54	0.0014	2.85345	0.5113051	0.402927604	0.108
cg10018203	II	37	14	106179136		0.58	0.00041	3.39184	0.5968168	0.48902959	0.108
cg24640101	II	37	15	25482669	SNORD115-37	0.46	0.01197	1.92207	0.36158311	0.253261859	0.108
cg02208504	II	37	15	26042919	ATP10A	0.42	0.02991	1.52413	0.6588413	0.550422578	0.108
cg05678749	I	37	15	27216819	GABRG3	0.42	0.02991	1.52413	0.19889006	0.306415471	-0.108
cg11342764	II	37	15	29428738	FAM189A1	0.67	2.34E-05	4.63072	0.66372502	0.772040379	-0.108
cg20317748	II	37	15	40633124	C15orf52	0.5	0.00432	2.36443	0.36579	0.473367237	-0.108
cg17244218	II	37	15	42258950	EHD4	0.5	0.00432	2.36443	0.22597888	0.334160273	-0.108
cg19639490	II	37	15	75146663	SCAMP2	0.42	0.02991	1.52413	0.78791573	0.67991988	0.108
cg21840434	II	37	15	79297210	RASGRF1;RASGRF1;	0.46	0.01197	1.92207	0.37894254	0.486913592	-0.108
cg08413390	II	37	15	84944048		0.62	0.0001	3.98291	0.53386011	0.641559572	-0.108
cg05394456	II	37	16	129230	MPG;MPG;MPG	0.42	0.02991	1.52413	0.17467372	0.282379164	-0.108
cg15337006	II	37	16	31271009	ITGAM;ITGAM	0.46	0.01197	1.92207	0.28625766	0.394420713	-0.108
cg08797704	II	37	16	65692605		0.5	0.00432	2.36443	0.76505802	0.656745268	0.108
cg03157862	II	37	16	66519174		0.42	0.02991	1.52413	0.73501386	0.62746232	0.108
cg01824618	II	37	16	85315212		0.42	0.02991	1.52413	0.74201331	0.633798819	0.108

cg03386347	II	37	16	88261775		0.46	0.01197	1.92207	0.72448372	0.61677402	0.108
cg01046511	II	37	17	7742971	KDM6B	0.67	2.34E-05	4.63072	0.75217818	0.859970941	-0.108
cg21376526	I	37	17	8659409	SPDYE4	0.42	0.02991	1.52413	0.8343737	0.726117441	0.108
cg20525527	II	37	17	17056629	MPRIP;MPRIP	0.5	0.00432	2.36443	0.61643017	0.724634066	-0.108
cg26612727	II	37	17	38024636	ZPBP2;ZPBP2	0.58	0.00041	3.39184	0.43282458	0.541006787	-0.108
cg20669292	I	37	17	40823420	PLEKHH3	0.46	0.01197	1.92207	0.38015251	0.272647285	0.108
cg20508508	II	37	17	48473757	LRRC59	0.46	0.01197	1.92207	0.18907004	0.297232322	-0.108
cg14427590	II	37	17	60695089		0.54	0.0014	2.85345	0.62996376	0.738397679	-0.108
cg21659714	II	37	17	79785991	FAM195B;FAM195I	0.58	0.00041	3.39184	0.76874658	0.660852076	0.108
cg17931529	I	37	19	2080023	MOBKL2A	0.46	0.01197	1.92207	0.81311163	0.7048271	0.108
cg08035555	II	37	19	3558417	C19orf28;C19orf28	0.42	0.02991	1.52413	0.21685576	0.324675397	-0.108
cg04672103	II	37	19	16396929		0.54	0.0014	2.85345	0.11154619	0.219980552	-0.108
cg14085840	II	37	19	40939429		0.54	0.0014	2.85345	0.2641384	0.371791918	-0.108
cg16702690	II	37	19	51686316		0.5	0.00432	2.36443	0.25962489	0.367466523	-0.108
cg06000530	I	37	20	30135108	HM13;HM13;HM13	0.54	0.0014	2.85345	0.49353331	0.385041058	0.108
cg17271585	II	37	20	36153947	BLCAP;BLCAP;BLCA	0.46	0.01197	1.92207	0.62871168	0.520536952	0.108
cg27461254	II	37	20	39135472		0.42	0.02991	1.52413	0.19291316	0.300984816	-0.108
cg24365215	II	37	20	61315208		0.54	0.0014	2.85345	0.63606309	0.744097987	-0.108
cg23085846	II	37	20	62522518	TPD52L2;TPD52L2;T	0.42	0.02991	1.52413	0.64233976	0.750089641	-0.108
cg24974982	I	37	22	46403316	LOC730668	0.62	0.0001	3.98291	0.7008217	0.808847296	-0.108
cg25152348	I	37	22	50946712	NCAPH2;LMF2;NCA	0.71	4.57E-06	5.34042	0.47304174	0.364714528	0.108
cg17122213	I	37	1	1564920	MIB2;MIB2;MIB2;M	0.5	0.00432	2.36443	0.38037832	0.273362482	0.107
cg07708453	II	37	1	14032034	PRDM2;PRDM2;PRI	0.46	0.01197	1.92207	0.75801478	0.864744878	-0.107
cg10057098	II	37	1	39991426	LOC728448;BMP8A	0.54	0.0014	2.85345	0.75459714	0.647495533	0.107
cg25099516	II	37	1	48288813		0.42	0.02991	1.52413	0.70648339	0.599746261	0.107
cg13697378	I	37	1	68512845	DIRAS3	0.58	0.00041	3.39184	0.58870976	0.481772022	0.107
cg11839020	II	37	1	90309998	LRRC8D;LRRC8D	0.54	0.0014	2.85345	0.54479824	0.437980889	0.107
cg15081720	II	37	1	119866618		0.62	0.0001	3.98291	0.83163539	0.724190632	0.107
cg24843609	I	37	1	156211434	BGLAP	0.46	0.01197	1.92207	0.34775691	0.455034945	-0.107
cg09584785	II	37	1	162333354	NOS1AP;NOS1AP;N	0.67	2.34E-05	4.63072	0.87199171	0.764859399	0.107
cg06834507	II	37	1	200876957	C1orf106;C1orf106	0.42	0.02991	1.52413	0.30079411	0.407649035	-0.107
cg03422911	II	37	1	237205295	RYR2	0.58	0.00041	3.39184	0.23863756	0.345458171	-0.107
cg17214023	II	37	2	10176748		0.5	0.00432	2.36443	0.46250326	0.569015521	-0.107

cg27375286	II	37	2	11518493		0.42	0.02991	1.52413	0.7894183	0.682240319	0.107
cg03377767	II	37	2	17997138	MSGN1	0.46	0.01197	1.92207	0.78606907	0.678970721	0.107
cg10464312	II	37	2	66672688	MEIS1	0.67	2.34E-05	4.63072	0.25768166	0.36488363	-0.107
cg13522882	II	37	2	102316496	MAP4K4;MAP4K4;M	0.46	0.01197	1.92207	0.79680484	0.689961478	0.107
cg05733104	II	37	3	9032419	SRGAP3;SRGAP3	0.46	0.01197	1.92207	0.79437307	0.687719772	0.107
cg19551485	II	37	3	55693182	C3orf51;ERC2	0.46	0.01197	1.92207	0.76485986	0.658109172	0.107
cg23730027	II	37	3	57995180	FLNB;FLNB;FLNB;FL	0.54	0.0014	2.85345	0.24616144	0.353087966	-0.107
cg21464115	II	37	3	138157982	ESYT3	0.42	0.02991	1.52413	0.6142895	0.721312287	-0.107
cg21794419	II	37	3	182973032	MCF2L2;B3GNT5	0.58	0.00041	3.39184	0.71141653	0.818749605	-0.107
cg04398861	II	37	4	670694	MYL5	0.5	0.00432	2.36443	0.5061626	0.399322141	0.107
cg21705324	II	37	4	24200012		0.46	0.01197	1.92207	0.77358003	0.666185598	0.107
cg20367304	II	37	4	154391944	KIAA0922;KIAA092	0.5	0.00432	2.36443	0.76336848	0.656159653	0.107
cg14116129	I	37	5	1140748		0.58	0.00041	3.39184	0.43831997	0.331346662	0.107
cg22490780	I	37	5	16867064	MYO10	0.5	0.00432	2.36443	0.76072197	0.653388817	0.107
cg22437221	II	37	5	39203007	FYB;FYB	0.46	0.01197	1.92207	0.67172868	0.778912425	-0.107
cg08477768	II	37	5	57925482	RAB3C	0.54	0.0014	2.85345	0.32594522	0.432928267	-0.107
cg01772439	II	37	5	149012102	FLJ41603	0.54	0.0014	2.85345	0.67341148	0.780187801	-0.107
cg03172212	II	37	5	175873937	FAF2	0.5	0.00432	2.36443	0.52234623	0.628931383	-0.107
cg05143092	II	37	6	30619209	C6orf136;C6orf136	0.5	0.00432	2.36443	0.80724482	0.700050725	0.107
cg04985482	II	37	6	31382065	MICA	0.42	0.02991	1.52413	0.73196027	0.838613851	-0.107
cg00175838	I	37	6	31695027	DDAH2	0.46	0.01197	1.92207	0.64325861	0.749990709	-0.107
cg02272968	I	37	6	32015773	TNXB	0.42	0.02991	1.52413	0.59386546	0.48733225	0.107
cg14023774	II	37	6	33246157	B3GALT4	0.42	0.02991	1.52413	0.62533019	0.518666363	0.107
cg10479493	II	37	6	33772694	MLN;MLN	0.42	0.02991	1.52413	0.71329326	0.606632408	0.107
cg21130236	I	37	6	44246755	TMEM151B	0.54	0.0014	2.85345	0.75854422	0.651782271	0.107
cg12545949	II	37	6	71571446	SMAP1;SMAP1;B3G	0.46	0.01197	1.92207	0.70554487	0.812812567	-0.107
cg02159996	II	37	6	89927233	GABRR1;GABRR1	0.58	0.00041	3.39184	0.44601433	0.553475953	-0.107
cg06270737	II	37	6	138785358	NHSL1;NHSL1	0.46	0.01197	1.92207	0.51573862	0.622866786	-0.107
cg17310882	I	37	6	158066609	ZDHHC14;ZDHHC14	0.5	0.00432	2.36443	0.48633975	0.592851604	-0.107
cg13828236	II	37	7	80970		0.5	0.00432	2.36443	0.66363622	0.770272517	-0.107
cg24505167	II	37	7	1915268	MAD1L1;MAD1L1;M	0.5	0.00432	2.36443	0.23261035	0.339259098	-0.107
cg11724970	I	37	7	27182493	HOXA5	0.5	0.00432	2.36443	0.68640207	0.79306226	-0.107
cg00552235	I	37	7	100943163		0.46	0.01197	1.92207	0.11578135	0.222376019	-0.107

cg14839837	II	37	8	1834174	ARHGEF10	0.5	0.00432	2.36443	0.78226606	0.675609405	0.107
cg01588250	II	37	8	54427263		0.42	0.02991	1.52413	0.74976887	0.642443072	0.107
cg20162696	II	37	8	102167694		0.5	0.00432	2.36443	0.27501681	0.382280119	-0.107
cg22283754	II	37	8	128999428	PVT1	0.5	0.00432	2.36443	0.85650653	0.749276315	0.107
cg25398315	II	37	8	135938022		0.46	0.01197	1.92207	0.52398057	0.416613291	0.107
cg24002158	II	37	8	142369468	GPR20	0.5	0.00432	2.36443	0.43494665	0.541562256	-0.107
cg13448704	II	37	8	143596845	BAI1	0.54	0.0014	2.85345	0.55221665	0.65910671	-0.107
cg13845692	II	37	9	94259238		0.46	0.01197	1.92207	0.80190456	0.694826576	0.107
cg14400354	I	37	9	101570703	GALNT12	0.46	0.01197	1.92207	0.14628296	0.252987007	-0.107
cg02539754	II	37	9	139012954		0.67	2.34E-05	4.63072	0.71223529	0.818848079	-0.107
cg15449516	II	37	10	48593014		0.5	0.00432	2.36443	0.82614812	0.719038845	0.107
cg00234046	II	37	10	73169245	CDH23;CDH23	0.54	0.0014	2.85345	0.75970131	0.866999711	-0.107
cg14341467	II	37	10	121201554	GRK5	0.46	0.01197	1.92207	0.59914137	0.492207244	0.107
cg07055616	I	37	10	134600600	NKX6-2	0.42	0.02991	1.52413	0.08490641	0.191554173	-0.107
cg15352671	I	37	11	1331497	LOC255512;TOLLIP	0.42	0.02991	1.52413	0.37651167	0.269432846	0.107
cg22040301	II	37	11	2919798	SLC22A18AS;SLC22	0.5	0.00432	2.36443	0.47852026	0.585573477	-0.107
cg11623855	II	37	11	33563720	C11orf41	0.42	0.02991	1.52413	0.66381445	0.556410267	0.107
cg04171808	II	37	11	35188437	CD44;CD44;CD44;C	0.5	0.00432	2.36443	0.84879083	0.741479351	0.107
cg02937674	I	37	11	49579997	LOC440040	0.46	0.01197	1.92207	0.15856736	0.265574327	-0.107
cg11658781	II	37	11	73241335	FAM168A	0.5	0.00432	2.36443	0.78934716	0.895847277	-0.107
cg14190451	II	37	11	118083942	AMICA1;AMICA1;AI	0.54	0.0014	2.85345	0.78388015	0.891343918	-0.107
cg16592378	II	37	11	126498687	KIRREL3;KIRREL3	0.5	0.00432	2.36443	0.62696558	0.520132892	0.107
cg19796532	I	37	12	50426901		0.54	0.0014	2.85345	0.07870352	0.185344606	-0.107
cg06303168	II	37	12	56322959	WIBG	0.42	0.02991	1.52413	0.56001788	0.452541407	0.107
cg24448421	II	37	12	68737061		0.62	0.0001	3.98291	0.26472251	0.157250911	0.107
cg12596182	II	37	12	70082885	BEST3;BEST3;BEST3	0.62	0.0001	3.98291	0.60142687	0.708762774	-0.107
cg08337166	II	37	12	108686134	CMKLR1;CMKLR1;C	0.5	0.00432	2.36443	0.54896124	0.442120369	0.107
cg08318726	I	37	12	114844265	TBX5;TBX5;TBX5	0.46	0.01197	1.92207	0.10493099	0.212093405	-0.107
cg06072822	I	37	13	113652069	MCF2L;MCF2L	0.62	0.0001	3.98291	0.61786781	0.725029316	-0.107
cg10418289	II	37	14	23308294	MMP14	0.54	0.0014	2.85345	0.56929732	0.67675155	-0.107
cg04728863	II	37	14	58863362	TOMM20L	0.5	0.00432	2.36443	0.690821	0.797340769	-0.107
cg16577789	II	37	14	94186558	PRIMA1	0.5	0.00432	2.36443	0.64143634	0.534258973	0.107
cg00946921	I	37	14	106025021		0.5	0.00432	2.36443	0.75874409	0.651402069	0.107

cg21594043	II	37	15	92491578	SLCO3A1;SLCO3A1	0.42	0.02991	1.52413	0.26467739	0.371615432	-0.107
cg00925492	II	37	16	29165632		0.46	0.01197	1.92207	0.41152953	0.518334384	-0.107
cg27659768	II	37	16	71523436	ZNF19	0.46	0.01197	1.92207	0.72487203	0.832152192	-0.107
cg09363128	II	37	16	85478932		0.58	0.00041	3.39184	0.51282605	0.406275534	0.107
cg26935333	I	37	16	90148962		0.46	0.01197	1.92207	0.44535556	0.338570849	0.107
cg04196298	I	37	17	1480710	SLC43A2	0.5	0.00432	2.36443	0.125156	0.231719798	-0.107
cg23530553	II	37	17	46622536	HOXB2	0.5	0.00432	2.36443	0.52518022	0.631741434	-0.107
cg05230392	II	37	17	55976522	CUEDC1	0.5	0.00432	2.36443	0.64782713	0.754938975	-0.107
cg16463557	II	37	17	79296334	TMEM105	0.54	0.0014	2.85345	0.64868599	0.541542199	0.107
cg16776981	I	37	17	79428142	BAHCC1	0.42	0.02991	1.52413	0.51672511	0.62335511	-0.107
cg15085044	II	37	17	80668757		0.42	0.02991	1.52413	0.70405629	0.597325343	0.107
cg06931418	II	37	19	1402419	GAMT;GAMT	0.58	0.00041	3.39184	0.17097379	0.278296397	-0.107
cg05376219	II	37	19	2346279	SPPL2B;SPPL2B	0.62	0.0001	3.98291	0.64134025	0.748721839	-0.107
cg18229071	II	37	19	2695245	GNG7	0.46	0.01197	1.92207	0.23089053	0.337603816	-0.107
cg20091384	I	37	19	2700927	GNG7	0.5	0.00432	2.36443	0.79772322	0.69065263	0.107
cg14950321	II	37	19	4530233	PLIN5	0.5	0.00432	2.36443	0.50232775	0.394997606	0.107
cg27052073	II	37	19	45654213	NKPD1	0.58	0.00041	3.39184	0.6411351	0.747970814	-0.107
cg15114651	II	37	19	47289410	SLC1A5;SLC1A5;SLC	0.42	0.02991	1.52413	0.54848411	0.441672638	0.107
cg11311352	II	37	19	56058710		0.62	0.0001	3.98291	0.22298993	0.330434257	-0.107
cg04432319	I	37	20	13200931	ISM1	0.54	0.0014	2.85345	0.03259036	0.139722145	-0.107
cg27529037	II	37	20	44575021	PCIF1	0.62	0.0001	3.98291	0.63365177	0.740229457	-0.107
cg06653796	II	37	20	62367805	LIME1	0.5	0.00432	2.36443	0.19628408	0.089325592	0.107
cg07806328	II	37	21	45774664	TRPM2	0.5	0.00432	2.36443	0.69946982	0.806436295	-0.107
cg07443748	II	37	22	17073594	CCT8L2;CCT8L2	0.46	0.01197	1.92207	0.80714325	0.70028172	0.107
cg06458106	II	37	22	17591807		0.71	4.57E-06	5.34042	0.70612711	0.813334015	-0.107
cg11522804	I	37	22	19438246	UFD1L;UFD1L	0.54	0.0014	2.85345	0.74848057	0.855338486	-0.107
cg07313319	II	37	22	38714426	CSNK1E;CSNK1E	0.46	0.01197	1.92207	0.39864011	0.505504484	-0.107
cg10219223	II	37	1	6420713	ACOT7;ACOT7;ACO	0.54	0.0014	2.85345	0.58284012	0.688681016	-0.106
cg04578903	II	37	1	21754899		0.5	0.00432	2.36443	0.22366581	0.329837163	-0.106
cg22526531	II	37	1	35913782	KIAA0319L	0.46	0.01197	1.92207	0.7481485	0.853708329	-0.106
cg11674933	II	37	1	36948313	CSF3R;CSF3R;CSF3F	0.42	0.02991	1.52413	0.18769667	0.294067853	-0.106
cg02451774	II	37	1	147801218		0.5	0.00432	2.36443	0.31282936	0.419291323	-0.106
cg05848175	II	37	1	153330257	S100A9	0.5	0.00432	2.36443	0.15388583	0.259918828	-0.106

cg16986748	II	37	1	154971109		0.71	4.57E-06	5.34042	0.27907311	0.385175151	-0.106
cg00714874	II	37	1	161679461	FCRLA	0.46	0.01197	1.92207	0.48720682	0.380747009	0.106
cg15166311	II	37	1	178007283		0.58	0.00041	3.39184	0.12306097	0.228776554	-0.106
cg15677434	II	37	1	206226009	AVPR1B	0.54	0.0014	2.85345	0.65971422	0.765499216	-0.106
cg20316614	II	37	1	226065637	TMEM63A	0.54	0.0014	2.85345	0.25903718	0.36553696	-0.106
cg20594765	II	37	1	230276238	GALNT2	0.62	0.0001	3.98291	0.71878677	0.82482486	-0.106
cg08458637	II	37	1	231747906	TSNAX-DISC1;TSNA	0.42	0.02991	1.52413	0.68412364	0.78975897	-0.106
cg04011474	II	37	2	28904455		0.46	0.01197	1.92207	0.76971071	0.663945106	0.106
cg02582355	II	37	2	38001560		0.46	0.01197	1.92207	0.70899718	0.603427155	0.106
cg04674832	II	37	2	72078638		0.58	0.00041	3.39184	0.61913005	0.725444095	-0.106
cg08493581	II	37	2	85472279	TCF7L1	0.46	0.01197	1.92207	0.74666129	0.640257272	0.106
cg24715680	II	37	2	215675429	BARD1	0.46	0.01197	1.92207	0.39788855	0.504272332	-0.106
cg17316300	II	37	2	237381593	IQCA1	0.5	0.00432	2.36443	0.71463354	0.608774554	0.106
cg16887422	II	37	2	240035107	HDAC4	0.54	0.0014	2.85345	0.52489302	0.630560893	-0.106
cg27125504	II	37	3	52865637	ITIH4;ITIH4	0.5	0.00432	2.36443	0.7011928	0.807082669	-0.106
cg05116378	II	37	3	141377105		0.5	0.00432	2.36443	0.46488021	0.571314463	-0.106
cg23980776	I	37	4	657074	PDE6B;PDE6B;PDE6	0.42	0.02991	1.52413	0.61759585	0.723263195	-0.106
cg06943431	II	37	4	3475041	DOK7;DOK7	0.42	0.02991	1.52413	0.73389123	0.627659465	0.106
cg04710179	II	37	4	37891376	TBC1D1	0.46	0.01197	1.92207	0.68720177	0.793246377	-0.106
cg02661034	II	37	5	1477269	LPCAT1	0.5	0.00432	2.36443	0.65526051	0.761711113	-0.106
cg23987134	II	37	5	17158319	LOC285696	0.62	0.0001	3.98291	0.51793916	0.624026005	-0.106
cg26078977	I	37	5	59189467	PDE4D;PDE4D;PDE4	0.54	0.0014	2.85345	0.12019983	0.226163365	-0.106
cg12643293	I	37	5	176101883		0.62	0.0001	3.98291	0.57379977	0.468209349	0.106
cg24843380	I	37	5	178367827	ZNF454	0.42	0.02991	1.52413	0.09214325	0.198455179	-0.106
cg18505691	II	37	6	29723320		0.5	0.00432	2.36443	0.73830918	0.844456993	-0.106
cg13079571	II	37	6	30297257	TRIM39;TRIM39	0.5	0.00432	2.36443	0.79825198	0.904050369	-0.106
cg04425551	II	37	6	30297338	TRIM39;TRIM39	0.42	0.02991	1.52413	0.77411009	0.88026225	-0.106
cg06321045	I	37	6	31088343	CDSN;PSORS1C1	0.46	0.01197	1.92207	0.45421016	0.559855388	-0.106
cg27490128	II	37	6	31560441	NCR3;NCR3;NCR3	0.42	0.02991	1.52413	0.79023987	0.684481434	0.106
cg08355863	II	37	6	33587496		0.5	0.00432	2.36443	0.67787569	0.783884923	-0.106
cg23372795	II	37	6	39284679	KCNK16;KCNK16;KC	0.46	0.01197	1.92207	0.67828815	0.571936788	0.106
cg00191957	II	37	6	43476766	C6orf154	0.54	0.0014	2.85345	0.38325043	0.489250007	-0.106
cg24863152	II	37	6	151134876	PLEKHG1	0.5	0.00432	2.36443	0.53907893	0.432593606	0.106

cg18747197	II	37	6	158110124		0.54	0.0014	2.85345	0.62392913	0.729634096	-0.106
cg07007035	II	37	7	1776076	ELFN1	0.46	0.01197	1.92207	0.70563103	0.599661015	0.106
cg13998945	II	37	7	45035665		0.54	0.0014	2.85345	0.71441442	0.608861153	0.106
cg08798349	II	37	7	102613822	FBXL13;FBXL13	0.42	0.02991	1.52413	0.60762719	0.714034664	-0.106
cg09322278	II	37	7	134233791		0.5	0.00432	2.36443	0.73461964	0.629002048	0.106
cg19418190	II	37	7	158221076	PTPRN2;PTPRN2;PT	0.46	0.01197	1.92207	0.78230676	0.675984156	0.106
cg06892907	II	37	8	103818062		0.67	2.34E-05	4.63072	0.37317189	0.478930065	-0.106
cg21934273	I	37	8	143620593	BAI1	0.58	0.00041	3.39184	0.78994481	0.684079194	0.106
cg23629150	II	37	8	144416404	TOP1MT	0.46	0.01197	1.92207	0.6947167	0.800695454	-0.106
cg14032964	II	37	9	92159306		0.42	0.02991	1.52413	0.77001618	0.875759021	-0.106
cg13630095	II	37	9	125137594	PTGS1;PTGS1	0.71	4.57E-06	5.34042	0.35892906	0.464691472	-0.106
cg13914004	II	37	9	130659670	ST6GALNAC6	0.42	0.02991	1.52413	0.22697891	0.333395578	-0.106
cg13549174	II	37	9	133910264	LAMC3	0.54	0.0014	2.85345	0.77363597	0.668113046	0.106
cg20017995	II	37	9	136216076	SNORD24;SNORD36	0.5	0.00432	2.36443	0.6553641	0.549376147	0.106
cg25200616	II	37	9	138800390	CAMSAP1	0.5	0.00432	2.36443	0.59469456	0.488712716	0.106
cg11873854	II	37	9	139642875	LCN6	0.5	0.00432	2.36443	0.55759543	0.451761163	0.106
cg10880928	II	37	9	139715701	C9orf86;C9orf86	0.46	0.01197	1.92207	0.28962985	0.395671769	-0.106
cg14334460	II	37	9	140346899	NELF;NELF;NELF;NE	0.54	0.0014	2.85345	0.72823531	0.834133929	-0.106
cg07121693	II	37	10	44148437		0.46	0.01197	1.92207	0.57265767	0.466592079	0.106
cg12161982	II	37	10	49802919	ARHGAP22	0.5	0.00432	2.36443	0.85095443	0.745303582	0.106
cg23786747	II	37	10	64320150	ZNF365;ZNF365	0.5	0.00432	2.36443	0.75421562	0.859724179	-0.106
cg18719787	II	37	10	79929586		0.5	0.00432	2.36443	0.69398162	0.587522752	0.106
cg16536739	II	37	10	99532636	SFRP5	0.54	0.0014	2.85345	0.70835474	0.602833737	0.106
cg15903032	II	37	10	101297605		0.67	2.34E-05	4.63072	0.41096121	0.517229788	-0.106
cg11229101	II	37	10	134230885	PWWP2B;PWWP2E	0.5	0.00432	2.36443	0.62434592	0.730622671	-0.106
cg07420362	II	37	11	3647419	TRPC2	0.46	0.01197	1.92207	0.22289246	0.329312718	-0.106
cg11096993	II	37	11	67417958	ACY3	0.46	0.01197	1.92207	0.5478474	0.441458816	0.106
cg03161498	II	37	11	75265195		0.46	0.01197	1.92207	0.77850925	0.67242125	0.106
cg16100721	II	37	11	102300650	TMEM123	0.58	0.00041	3.39184	0.54545441	0.439293075	0.106
cg18268562	II	37	11	118842392	FOXR1	0.46	0.01197	1.92207	0.47842027	0.584471787	-0.106
cg10462961	II	37	11	130493253		0.58	0.00041	3.39184	0.60704093	0.500928991	0.106
cg08317801	I	37	12	8286738	CLEC4A;CLEC4A;CLE	0.58	0.00041	3.39184	0.88461765	0.778164556	0.106
cg21422475	II	37	12	51890999	SLC4A8;SLC4A8	0.5	0.00432	2.36443	0.78973576	0.89622815	-0.106

cg18845433	II	37	12	53193325		0.5	0.00432	2.36443	0.68456104	0.578308969	0.106
cg10157862	II	37	12	69725695		0.58	0.00041	3.39184	0.46111349	0.566666987	-0.106
cg01616876	II	37	12	113544928	RASAL1	0.5	0.00432	2.36443	0.16995121	0.275517142	-0.106
cg10839475	II	37	12	113959012		0.5	0.00432	2.36443	0.78866986	0.682720654	0.106
cg19945931	I	37	12	133022760		0.42	0.02991	1.52413	0.6501391	0.755977588	-0.106
cg04947907	II	37	12	133345285		0.5	0.00432	2.36443	0.30083179	0.407226261	-0.106
cg14688299	I	37	13	30728099		0.5	0.00432	2.36443	0.65390936	0.54745834	0.106
cg03865667	II	37	13	58205678	PCDH17	0.62	0.0001	3.98291	0.1711203	0.276832022	-0.106
cg25006823	II	37	14	67982456	TMEM229B	0.5	0.00432	2.36443	0.4457372	0.339280985	0.106
cg08079330	II	37	14	76847792	ESRRB	0.54	0.0014	2.85345	0.49972995	0.394198804	0.106
cg18633154	II	37	14	77296234	C14orf166B	0.46	0.01197	1.92207	0.76821291	0.662386269	0.106
cg09285543	II	37	14	101296134	MEG3;MEG3;MEG3	0.46	0.01197	1.92207	0.80528592	0.699273976	0.106
cg22799691	II	37	14	103821330		0.62	0.0001	3.98291	0.75605138	0.64962471	0.106
cg14172849	II	37	14	104171259	XRCC3;XRCC3;XRCC	0.5	0.00432	2.36443	0.49870935	0.604540864	-0.106
cg24632582	I	37	15	41233701		0.62	0.0001	3.98291	0.50233937	0.608661403	-0.106
cg05737682	I	37	16	3021802	PAQR4	0.62	0.0001	3.98291	0.76982441	0.663846718	0.106
cg13574337	II	37	16	4016720	ADCY9	0.54	0.0014	2.85345	0.70064977	0.594362344	0.106
cg06323332	II	37	16	4714229	MGRN1;MGRN1;M	0.54	0.0014	2.85345	0.19571049	0.30200545	-0.106
cg10505257	I	37	16	4731639	MGRN1;MGRN1;M	0.5	0.00432	2.36443	0.7996393	0.905233593	-0.106
cg00504410	II	37	16	4732262	MGRN1;MGRN1;M	0.5	0.00432	2.36443	0.65612017	0.761730248	-0.106
cg00417823	II	37	16	56330268	GNAO1;GNAO1	0.46	0.01197	1.92207	0.64972085	0.543663468	0.106
cg04252938	II	37	16	88101013	BANP;BANP	0.42	0.02991	1.52413	0.65704022	0.55074359	0.106
cg04694209	II	37	16	89228970	C16orf81	0.46	0.01197	1.92207	0.76164057	0.655881181	0.106
cg18900669	II	37	17	7482456	CD68;CD68	0.42	0.02991	1.52413	0.2459643	0.352244197	-0.106
cg08197105	II	37	17	27284655	SEZ6;SEZ6	0.54	0.0014	2.85345	0.74018971	0.846207802	-0.106
cg26500312	II	37	17	30821755	MYO1D	0.54	0.0014	2.85345	0.72971867	0.835773122	-0.106
cg25451120	II	37	17	47287444	ABI3;GNGT2;ABI3	0.58	0.00041	3.39184	0.5765337	0.470659297	0.106
cg05310240	II	37	17	47979069		0.54	0.0014	2.85345	0.58571674	0.479794706	0.106
cg19716073	II	37	17	65039817	CACNG1	0.46	0.01197	1.92207	0.82837686	0.72239595	0.106
cg08352115	II	37	17	66356057	ARSG	0.71	4.57E-06	5.34042	0.69956869	0.805411627	-0.106
cg23120541	II	37	17	75422456	SEPT9;SEPT9;SEPT9	0.54	0.0014	2.85345	0.42133786	0.527446011	-0.106
cg24867468	I	37	17	76109222	TMC6;TMC6	0.5	0.00432	2.36443	0.83179402	0.725613009	0.106
cg00945209	I	37	17	76801579	USP36	0.46	0.01197	1.92207	0.71505278	0.609402186	0.106

cg25792439	I	37	17	78163268	CARD14;CARD14	0.5	0.00432	2.36443	0.73279881	0.838452298	-0.106
cg07737135	II	37	19	1356032	MUM1;MUM1	0.54	0.0014	2.85345	0.64243892	0.748316953	-0.106
cg19477361	II	37	19	2607903	GNG7	0.58	0.00041	3.39184	0.61134525	0.504913373	0.106
cg13078421	II	37	19	2624622	GNG7	0.54	0.0014	2.85345	0.27837731	0.172845949	0.106
cg21207418	II	37	19	11688711	ACP5;ACP5;ACP5;A	0.42	0.02991	1.52413	0.75491904	0.648957194	0.106
cg17320378	II	37	19	17664088	FAM129C	0.46	0.01197	1.92207	0.57954293	0.685264654	-0.106
cg09466818	II	37	19	18965708	UPF1	0.5	0.00432	2.36443	0.55029262	0.656170022	-0.106
cg21107581	II	37	19	49258805	FGF21;FUT1	0.42	0.02991	1.52413	0.76493706	0.65862744	0.106
cg17204562	II	37	20	48568916	RNF114	0.46	0.01197	1.92207	0.73447681	0.840107778	-0.106
cg04343971	I	37	20	62570696		0.46	0.01197	1.92207	0.7008524	0.806366538	-0.106
cg13033054	II	37	21	33948561	TCP10L	0.42	0.02991	1.52413	0.60361084	0.709930114	-0.106
cg00520380	II	37	22	31643997	LIMK2;LIMK2;LIMK2	0.54	0.0014	2.85345	0.55766181	0.663543601	-0.106
cg01360115	II	37	22	50747878		0.62	0.0001	3.98291	0.48606494	0.591840609	-0.106
cg12718519	I	37	1	2058417	PRKCZ;PRKCZ;PRKC	0.46	0.01197	1.92207	0.15790961	0.052612421	0.105
cg21108519	II	37	1	3425603	MEGF6	0.42	0.02991	1.52413	0.70744049	0.602595689	0.105
cg04934305	II	37	1	17421315	PADI2	0.46	0.01197	1.92207	0.70873647	0.604090723	0.105
cg18931815	II	37	1	36565772	COL8A2	0.5	0.00432	2.36443	0.50026935	0.604862393	-0.105
cg18172358	II	37	1	76734569	ST6GALNAC3;ST6G/	0.46	0.01197	1.92207	0.78592885	0.680987737	0.105
cg12139069	II	37	1	145004468	PDE4DIP	0.62	0.0001	3.98291	0.46687582	0.361431065	0.105
cg04922029	II	37	1	159174728	DARC;DARC;DARC	0.58	0.00041	3.39184	0.53284796	0.638313175	-0.105
cg00071250	II	37	1	172628263	FASLG;FASLG	0.5	0.00432	2.36443	0.79234743	0.687717349	0.105
cg00091302	II	37	1	182602986		0.42	0.02991	1.52413	0.39572154	0.290866862	0.105
cg09186051	II	37	1	231981906	DISC1;DISC1;DISC1;	0.5	0.00432	2.36443	0.70884471	0.603469848	0.105
cg16576033	II	37	1	235268621		0.42	0.02991	1.52413	0.65384125	0.549070619	0.105
cg11644052	II	37	2	69665132	NFU1;NFU1;NFU1;N	0.62	0.0001	3.98291	0.61477596	0.719791843	-0.105
cg13758186	II	37	2	101959136		0.46	0.01197	1.92207	0.72699938	0.621616218	0.105
cg17501982	II	37	2	106417701	NCK2;NCK2	0.42	0.02991	1.52413	0.71090918	0.605983146	0.105
cg22478317	II	37	2	110271930		0.42	0.02991	1.52413	0.64817818	0.542910874	0.105
cg06197616	II	37	2	128348899	MYO7B	0.46	0.01197	1.92207	0.48966308	0.384601563	0.105
cg27211696	II	37	2	191398769	TMEM194B	0.46	0.01197	1.92207	0.24889541	0.144345814	0.105
cg02281854	II	37	2	217428125		0.54	0.0014	2.85345	0.77062642	0.665581556	0.105
cg01604404	I	37	2	237460590		0.5	0.00432	2.36443	0.51451347	0.409647997	0.105
cg21184951	II	37	2	240168413	HDAC4	0.54	0.0014	2.85345	0.57186738	0.677213882	-0.105

cg24574692	I	37	2	241926608		0.71	4.57E-06	5.34042	0.46985355	0.574777837	-0.105
cg17071731	II	37	3	11127293		0.5	0.00432	2.36443	0.46605548	0.5708829	-0.105
cg24877842	II	37	3	42201898	TRAK1;TRAK1	0.42	0.02991	1.52413	0.52490997	0.629966807	-0.105
cg01363198	II	37	3	134878739	EPHB1	0.5	0.00432	2.36443	0.54451847	0.439600339	0.105
cg13575298	II	37	3	170626725	EIF5A2	0.54	0.0014	2.85345	0.57269912	0.677860299	-0.105
cg11935638	II	37	3	184972145	EHHADH;EHHADH	0.5	0.00432	2.36443	0.39513895	0.499783896	-0.105
cg02519208	II	37	3	190105821	CLDN16	0.5	0.00432	2.36443	0.60565056	0.500942874	0.105
cg01409693	I	37	4	1542171		0.5	0.00432	2.36443	0.82607315	0.720762086	0.105
cg03925294	II	37	4	6247629		0.62	0.0001	3.98291	0.28989935	0.394516251	-0.105
cg11636702	I	37	4	11370466	MIR572	0.5	0.00432	2.36443	0.42592873	0.531092083	-0.105
cg05476182	II	37	5	133913122	PHF15	0.46	0.01197	1.92207	0.69122792	0.795996971	-0.105
cg10257049	II	37	5	154230308	C5orf4	0.42	0.02991	1.52413	0.26241507	0.367018823	-0.105
cg15901722	II	37	5	175974973	PCDH24	0.42	0.02991	1.52413	0.42264564	0.527405409	-0.105
cg12230983	II	37	6	15439701	JARID2	0.42	0.02991	1.52413	0.21022794	0.315669332	-0.105
cg10507304	II	37	6	27637520		0.42	0.02991	1.52413	0.2675565	0.372727496	-0.105
cg01400884	II	37	6	28583971		0.46	0.01197	1.92207	0.46787776	0.572978465	-0.105
cg03096746	II	37	6	29795436	HLA-G	0.46	0.01197	1.92207	0.36052294	0.465878842	-0.105
cg07333021	II	37	6	30612330	C6orf134	0.46	0.01197	1.92207	0.73126166	0.836592122	-0.105
cg18335326	I	37	6	30653659	KIAA1949;KIAA194	0.62	0.0001	3.98291	0.23527723	0.130005113	0.105
cg09232906	II	37	6	30684280	MDC1	0.42	0.02991	1.52413	0.74708212	0.64213686	0.105
cg10740660	II	37	6	31740849	C6orf27	0.67	2.34E-05	4.63072	0.42244792	0.527726921	-0.105
cg05767720	I	37	6	32117281	PRRT1	0.54	0.0014	2.85345	0.45853901	0.353386644	0.105
cg19350197	I	37	6	32782988	HLA-DOB	0.54	0.0014	2.85345	0.60861477	0.503821232	0.105
cg17931986	II	37	6	33131274	COL11A2;COL11A2;	0.46	0.01197	1.92207	0.49985385	0.604403458	-0.105
cg02266086	II	37	6	33161336	COL11A2;COL11A2;	0.42	0.02991	1.52413	0.48190202	0.586847273	-0.105
cg10494848	II	37	6	57127617		0.5	0.00432	2.36443	0.36760453	0.47300181	-0.105
cg00847453	II	37	6	110720501	DDO;DDO	0.54	0.0014	2.85345	0.63910075	0.744386625	-0.105
cg17852032	II	37	6	158066811	ZDHHC14;ZDHHC14	0.54	0.0014	2.85345	0.30714335	0.412559732	-0.105
cg23249717	I	37	6	167507105		0.46	0.01197	1.92207	0.77905155	0.673656636	0.105
cg10248100	II	37	7	872053	UNC84A;UNC84A	0.54	0.0014	2.85345	0.26013583	0.155570028	0.105
cg27205928	I	37	7	1062652	C7orf50;C7orf50;C7	0.42	0.02991	1.52413	0.72254135	0.617827959	0.105
cg07556911	II	37	7	2044787	MAD1L1;MAD1L1;M	0.54	0.0014	2.85345	0.53829785	0.432932578	0.105
cg01986619	II	37	7	2613963	IQCE;IQCE	0.42	0.02991	1.52413	0.82240341	0.717777612	0.105

cg08241694	II	37	7	50633896	DDC	0.42	0.02991	1.52413	0.68184664	0.576402657	0.105
cg24620463	I	37	7	95917902	SLC25A13;SLC25A1	0.79	1.06E-07	6.97389	0.4495441	0.344824173	0.105
cg26847503	II	37	7	143056341	FAM131B;FAM131I	0.46	0.01197	1.92207	0.70081126	0.595871993	0.105
cg24966363	II	37	8	8932588		0.5	0.00432	2.36443	0.19328317	0.298617911	-0.105
cg13518968	II	37	8	20138378		0.5	0.00432	2.36443	0.79913155	0.69408517	0.105
cg26027576	II	37	8	27441373		0.58	0.00041	3.39184	0.62563027	0.520132579	0.105
cg13708803	II	37	8	138898243		0.58	0.00041	3.39184	0.58184434	0.476783033	0.105
cg06671450	II	37	9	36985987	PAX5	0.62	0.0001	3.98291	0.23477563	0.33932554	-0.105
cg15282417	II	37	9	129245246	FAM125B	0.42	0.02991	1.52413	0.59978545	0.704565095	-0.105
cg13786567	II	37	9	137258920	RXRA	0.5	0.00432	2.36443	0.6674762	0.772369363	-0.105
cg10119075	II	37	10	7709738	ITIH5;ITIH5	0.54	0.0014	2.85345	0.12895536	0.233773672	-0.105
cg14156751	I	37	10	50976571		0.42	0.02991	1.52413	0.38489298	0.489666661	-0.105
cg23196756	II	37	10	72165580	EIF4EBP2	0.58	0.00041	3.39184	0.66287479	0.767925729	-0.105
cg12500300	II	37	10	133810178		0.46	0.01197	1.92207	0.81430459	0.709796814	0.105
cg04537282	II	37	11	331179		0.54	0.0014	2.85345	0.58200209	0.477225165	0.105
cg02871021	II	37	11	10715715	MRVI1;MRVI1;MRV	0.46	0.01197	1.92207	0.78975071	0.684266332	0.105
cg14780449	II	37	11	44578801		0.58	0.00041	3.39184	0.58474346	0.690085887	-0.105
cg18048562	II	37	11	57232463	RTN4RL2	0.5	0.00432	2.36443	0.41942283	0.524778572	-0.105
cg25104637	II	37	11	64405452	NRXN2;NRXN2;NRX	0.5	0.00432	2.36443	0.54273957	0.438154003	0.105
cg23227726	II	37	11	72143885	CLPB	0.5	0.00432	2.36443	0.44034539	0.545330639	-0.105
cg07744502	II	37	11	93271083	C11orf75	0.5	0.00432	2.36443	0.21941514	0.324016727	-0.105
cg04131101	II	37	11	94427846		0.54	0.0014	2.85345	0.78075781	0.675397372	0.105
cg09993718	II	37	11	117670657		0.54	0.0014	2.85345	0.48603301	0.38075014	0.105
cg17465423	II	37	12	54784180	ZNF385A;ZNF385A	0.54	0.0014	2.85345	0.30090784	0.406346852	-0.105
cg20051772	II	37	12	56325015	DGKA;DGKA;DGKA;	0.54	0.0014	2.85345	0.24192941	0.137408267	0.105
cg14280382	II	37	12	96094182	NTN4	0.5	0.00432	2.36443	0.76428223	0.659223214	0.105
cg04347477	I	37	12	125002007	NCOR2;NCOR2	0.42	0.02991	1.52413	0.3159386	0.421285518	-0.105
cg23222604	II	37	12	125110933		0.5	0.00432	2.36443	0.15608787	0.261089011	-0.105
cg25840538	II	37	13	114146138	TMCO3;DCUN1D2	0.67	2.34E-05	4.63072	0.13675524	0.242091089	-0.105
cg24686983	II	37	14	33832387	NPAS3;NPAS3;NPA!	0.54	0.0014	2.85345	0.76617306	0.661572771	0.105
cg01711160	II	37	14	85996499	FLRT2;FLRT2	0.58	0.00041	3.39184	0.13439432	0.23963599	-0.105
cg02540736	II	37	14	100774063	SLC25A29;MIR345	0.42	0.02991	1.52413	0.51760599	0.622913443	-0.105
cg02883666	II	37	14	101526670	MIR496	0.42	0.02991	1.52413	0.70893835	0.604423882	0.105

cg11831431	I	37	15	73639105	HCN4	0.46	0.01197	1.92207	0.5292993	0.423819037	0.105
cg21242079	II	37	15	79101063	ADAMTS7	0.46	0.01197	1.92207	0.48145072	0.376903185	0.105
cg05251269	II	37	16	2083128	SLC9A3R2;SLC9A3R	0.58	0.00041	3.39184	0.25667496	0.361393707	-0.105
cg04242728	II	37	16	2536153	TBC1D24	0.54	0.0014	2.85345	0.52250427	0.627212056	-0.105
cg01126567	I	37	16	20085047	GPR139;GPR139	0.46	0.01197	1.92207	0.03555035	0.140201342	-0.105
cg07726287	II	37	16	84318109		0.46	0.01197	1.92207	0.53175383	0.636855935	-0.105
cg02689514	I	37	16	88992127	CBFA2T3;CBFA2T3	0.5	0.00432	2.36443	0.69049608	0.585869711	0.105
cg07058377	II	37	17	2041773	SMG6;SMG6	0.62	0.0001	3.98291	0.28419777	0.389513715	-0.105
cg16513459	I	37	17	7832932	KCNAB3	0.5	0.00432	2.36443	0.77334529	0.668044355	0.105
cg01678701	I	37	17	8250623		0.46	0.01197	1.92207	0.55663449	0.661342169	-0.105
cg11851174	II	37	17	17712609	RAI1	0.46	0.01197	1.92207	0.69153574	0.586984076	0.105
cg19942495	II	37	17	32484027	ACCN1	0.58	0.00041	3.39184	0.20731458	0.311999484	-0.105
cg09795027	II	37	17	74006440	EVPL	0.79	1.06E-07	6.97389	0.37761582	0.482906638	-0.105
cg10138630	II	37	17	74024966	EVPL	0.5	0.00432	2.36443	0.30894716	0.413995369	-0.105
cg12098949	II	37	17	75367442	SEPT9;SEPT9;SEPT9	0.42	0.02991	1.52413	0.58342305	0.478174615	0.105
cg19654743	II	37	17	75446592	SEPT9;SEPT9;SEPT9	0.5	0.00432	2.36443	0.52421215	0.419071773	0.105
cg06513247	II	37	17	75446661	SEPT9;SEPT9;SEPT9	0.62	0.0001	3.98291	0.36127888	0.2567133	0.105
cg04268643	II	37	17	76037239	TNRC6C;TNRC6C	0.42	0.02991	1.52413	0.47705186	0.581924539	-0.105
cg22833809	II	37	17	76129984	TMC8;TMC6	0.46	0.01197	1.92207	0.36614795	0.261153797	0.105
cg01498832	II	37	17	78682934	RPTOR;RPTOR	0.42	0.02991	1.52413	0.74167315	0.846960973	-0.105
cg26638570	I	37	17	79409020	BAHCC1	0.5	0.00432	2.36443	0.73136419	0.835956822	-0.105
cg19284277	II	37	17	80190054	SLC16A3;SLC16A3;S	0.5	0.00432	2.36443	0.19201218	0.297499383	-0.105
cg22432760	I	37	17	80829718	TBCD	0.5	0.00432	2.36443	0.70412706	0.599324271	0.105
cg00663986	II	37	17	80866232	TBCD	0.46	0.01197	1.92207	0.66132723	0.556269583	0.105
cg01635063	I	37	19	728385	PALM;PALM	0.58	0.00041	3.39184	0.69986286	0.804726458	-0.105
cg04804052	II	37	19	11094658	SMARCA4;SMARCA	0.46	0.01197	1.92207	0.45454189	0.559489889	-0.105
cg07938642	II	37	19	18338579	PDE4C;PDE4C	0.46	0.01197	1.92207	0.72186754	0.616651839	0.105
cg01485075	II	37	19	22817371	ZNF492	0.46	0.01197	1.92207	0.08860297	0.193401244	-0.105
cg15473329	II	37	19	39862918	SAMD4B	0.46	0.01197	1.92207	0.73393197	0.628672338	0.105
cg03313447	II	37	19	41829042	CCDC97	0.54	0.0014	2.85345	0.39762858	0.502752227	-0.105
cg14267671	II	37	19	45572412	SFRS16	0.54	0.0014	2.85345	0.66161613	0.766875552	-0.105
cg09293816	II	37	19	50015532	FCGRT;FCGRT	0.54	0.0014	2.85345	0.2917116	0.397109503	-0.105
cg03117379	II	37	20	17943971	SNORD17;SNX5;SN)	0.5	0.00432	2.36443	0.68048599	0.575785324	0.105

cg08271622	I	37	20	37053969	LOC388796;SNORA	0.5	0.00432	2.36443	0.78678978	0.682112582	0.105
cg26674160	I	37	21	48025100	S100B	0.46	0.01197	1.92207	0.72578485	0.830318412	-0.105
cg17336139	I	37	22	22901648	PRAME;PRAME;PRA	0.46	0.01197	1.92207	0.26217766	0.157344983	0.105
cg19603202	II	37	22	38431726		0.42	0.02991	1.52413	0.68915194	0.584162582	0.105
cg21345826	II	37	22	39353650	APOBEC3A;APOBEC	0.58	0.00041	3.39184	0.68110369	0.785894337	-0.105
cg04934530	II	37	22	47080171		0.42	0.02991	1.52413	0.63017093	0.525081463	0.105
cg17848797	II	37	1	1198269	UBE2J2;UBE2J2;LO(	0.54	0.0014	2.85345	0.78757342	0.89165108	-0.104
cg03427058	I	37	1	1565025	MIB2;MIB2;MIB2;M	0.62	0.0001	3.98291	0.33124292	0.227580874	0.104
cg19809157	II	37	1	9422438	SPSB1	0.46	0.01197	1.92207	0.1619908	0.26565385	-0.104
cg13454846	II	37	1	16068375	TMEM82	0.62	0.0001	3.98291	0.63377972	0.529378066	0.104
cg18082979	II	37	1	32085118	HCRTR1	0.5	0.00432	2.36443	0.63444382	0.530144558	0.104
cg10092779	II	37	1	57294085		0.46	0.01197	1.92207	0.76357942	0.659461993	0.104
cg22488256	II	37	1	66258046	PDE4B;PDE4B	0.46	0.01197	1.92207	0.18922824	0.293031479	-0.104
cg09028383	II	37	1	150980411	FAM63A;PRUNE;FA	0.5	0.00432	2.36443	0.53011137	0.633731113	-0.104
cg22012476	II	37	1	168664941	DPT	0.46	0.01197	1.92207	0.75879279	0.654886475	0.104
cg13209335	II	37	1	180878648		0.5	0.00432	2.36443	0.85055674	0.746681232	0.104
cg26822728	II	37	1	202164063	LGR6	0.46	0.01197	1.92207	0.59842806	0.494000791	0.104
cg15490070	II	37	1	203148132	CHI3L1	0.5	0.00432	2.36443	0.31928125	0.423652505	-0.104
cg15732451	II	37	1	228997949		0.42	0.02991	1.52413	0.72999902	0.833589026	-0.104
cg15723468	II	37	1	230387268	GALNT2	0.46	0.01197	1.92207	0.82160996	0.717562311	0.104
cg12647574	II	37	2	12824729		0.5	0.00432	2.36443	0.25048201	0.354889458	-0.104
cg05360477	II	37	2	12959313		0.5	0.00432	2.36443	0.60652422	0.710297858	-0.104
cg25784219	II	37	2	28618328	FOSL2	0.46	0.01197	1.92207	0.19662939	0.300258176	-0.104
cg23688350	II	37	2	110317956	SEPT10;SEPT10	0.54	0.0014	2.85345	0.78725467	0.683748626	0.104
cg05208178	II	37	2	233928423	INPP5D;INPP5D	0.5	0.00432	2.36443	0.21701336	0.112977618	0.104
cg03626672	II	37	2	237478664	CXCR7	0.46	0.01197	1.92207	0.68817784	0.584549159	0.104
cg02135859	II	37	2	239140634	LOC643387;LOC15:	0.46	0.01197	1.92207	0.5069797	0.611425352	-0.104
cg15978561	II	37	2	240196996	HDAC4	0.46	0.01197	1.92207	0.29052993	0.394871139	-0.104
cg06519434	II	37	3	38664257	SCN5A;SCN5A;SCN5	0.5	0.00432	2.36443	0.61998905	0.515754395	0.104
cg01368219	II	37	3	54999791	CACNA2D3	0.5	0.00432	2.36443	0.56862498	0.672835635	-0.104
cg05963085	II	37	3	112359648	CCDC80;CCDC80;C(	0.5	0.00432	2.36443	0.36890697	0.472569487	-0.104
cg14456683	II	37	3	147127010	ZIC1	0.58	0.00041	3.39184	0.27050424	0.374426936	-0.104
cg10498502	II	37	3	185928164	DGKG;DGKG;DGKG	0.42	0.02991	1.52413	0.30140515	0.405603167	-0.104

cg01945624	II	37	4	8230847	SH3TC1	0.54	0.0014	2.85345	0.48783108	0.384239356	0.104
cg14366742	I	37	4	8546989		0.46	0.01197	1.92207	0.49172651	0.388166355	0.104
cg01294808	I	37	5	3599686	IRX1	0.46	0.01197	1.92207	0.30958958	0.413194272	-0.104
cg10581071	II	37	5	10653304	ANKRD33B	0.5	0.00432	2.36443	0.66876906	0.772490897	-0.104
cg03602014	II	37	5	126564681		0.46	0.01197	1.92207	0.37524386	0.479073297	-0.104
cg24119225	II	37	5	140344483	PCDHA7;PCDHAC2;	0.42	0.02991	1.52413	0.69782582	0.593337477	0.104
cg19646897	II	37	5	156536379	HAVCR2	0.46	0.01197	1.92207	0.53128728	0.426847119	0.104
cg26729204	II	37	6	2932241		0.5	0.00432	2.36443	0.41108541	0.306871107	0.104
cg18113826	II	37	6	31583942	AIF1;AIF1;AIF1	0.5	0.00432	2.36443	0.14154509	0.245782678	-0.104
cg16072814	II	37	6	32189397	NOTCH4	0.46	0.01197	1.92207	0.72382069	0.619938405	0.104
cg12699756	II	37	6	33173482	HSD17B8	0.46	0.01197	1.92207	0.79339385	0.68890386	0.104
cg17762073	I	37	6	34024220	GRM4	0.5	0.00432	2.36443	0.49590906	0.392348935	0.104
cg07037963	II	37	6	43265846	SLC22A7;SLC22A7	0.46	0.01197	1.92207	0.60749059	0.503282197	0.104
cg09783253	I	37	6	157932130	ZDHHC14;ZDHHC14	0.46	0.01197	1.92207	0.45100062	0.554943113	-0.104
cg08973950	I	37	7	1083309	C7orf50;C7orf50;C7	0.42	0.02991	1.52413	0.57128854	0.675044534	-0.104
cg00589581	II	37	7	1883760	MAD1L1;MAD1L1;M	0.42	0.02991	1.52413	0.48341898	0.379184599	0.104
cg00502509	I	37	7	1956121	MAD1L1;MAD1L1;M	0.54	0.0014	2.85345	0.82612927	0.930239925	-0.104
cg04260633	II	37	7	2687305	TTYH3	0.54	0.0014	2.85345	0.53295803	0.637433096	-0.104
cg12190994	II	37	7	4305061	SDK1;SDK1	0.46	0.01197	1.92207	0.40213376	0.506371266	-0.104
cg06480942	II	37	7	73116029	STX1A;STX1A	0.62	0.0001	3.98291	0.6776104	0.7814968	-0.104
cg09343421	II	37	7	100483511	SRRT;SRRT;SRRT;SR	0.62	0.0001	3.98291	0.64248454	0.5383789	0.104
cg07783094	II	37	7	157956912	PTPRN2;PTPRN2;PT	0.62	0.0001	3.98291	0.53206772	0.636561511	-0.104
cg01770362	II	37	8	23385913	SLC25A37	0.46	0.01197	1.92207	0.61701332	0.721233787	-0.104
cg13822256	II	37	8	24811266	NEFL	0.54	0.0014	2.85345	0.7631956	0.659369863	0.104
cg22995724	I	37	8	144639363	GSDMD;GSDMD	0.54	0.0014	2.85345	0.86327374	0.967472834	-0.104
cg13832290	I	37	9	35649906	SIT1	0.54	0.0014	2.85345	0.80129449	0.697512455	0.104
cg02385728	II	37	9	133803377	FIBCD1;FIBCD1	0.42	0.02991	1.52413	0.76790275	0.663988284	0.104
cg14331623	II	37	9	134155084		0.46	0.01197	1.92207	0.80531466	0.7011355	0.104
cg26129108	II	37	9	134698926		0.5	0.00432	2.36443	0.61795533	0.513710105	0.104
cg05493344	II	37	9	136567064	SARDH;SARDH	0.42	0.02991	1.52413	0.58789553	0.484017351	0.104
cg21120249	II	37	9	139921971	C9orf139;C9orf139	0.58	0.00041	3.39184	0.56903062	0.673088823	-0.104
cg02313434	II	37	10	5508980		0.46	0.01197	1.92207	0.69885805	0.802587613	-0.104
cg24385652	II	37	10	50329828		0.46	0.01197	1.92207	0.73460271	0.630714674	0.104

cg12800012	I	37	10	105600413	SH3PXD2A	0.58	0.00041	3.39184	0.73890553	0.843253129	-0.104
cg17624196	II	37	10	131841008		0.46	0.01197	1.92207	0.4660025	0.362389086	0.104
cg16129800	II	37	11	2920414	SLC22A18AS;SLC22.	0.67	2.34E-05	4.63072	0.30550574	0.409170291	-0.104
cg01607369	II	37	11	7598673	PPFIBP2	0.54	0.0014	2.85345	0.37035741	0.474102535	-0.104
cg14338590	II	37	11	16848924	PLEKHA7	0.42	0.02991	1.52413	0.74269325	0.638319433	0.104
cg08479635	II	37	11	45114570	PRDM11	0.54	0.0014	2.85345	0.25759104	0.36152119	-0.104
cg14472806	II	37	11	66055327	YIF1A	0.62	0.0001	3.98291	0.23861485	0.342286367	-0.104
cg18002602	II	37	11	66138449	SLC29A2	0.54	0.0014	2.85345	0.27192199	0.37545832	-0.104
cg23456595	I	37	11	67203434	PTPRCAP	0.42	0.02991	1.52413	0.71242896	0.608612114	0.104
cg23130075	II	37	11	67418365	ACY3	0.54	0.0014	2.85345	0.45781779	0.353971487	0.104
cg25633067	II	37	11	68037299	C11orf24	0.62	0.0001	3.98291	0.66166219	0.765404602	-0.104
cg06441398	II	37	11	70317455	SHANK2;SHANK2	0.42	0.02991	1.52413	0.68340688	0.579008812	0.104
cg25899154	II	37	11	72897143		0.46	0.01197	1.92207	0.69437022	0.798485853	-0.104
cg11308643	II	37	11	105480788	GRIA4;GRIA4;GRIA4	0.5	0.00432	2.36443	0.1607409	0.265144309	-0.104
cg09251429	I	37	11	124735128	ROBO3	0.42	0.02991	1.52413	0.18394678	0.287902587	-0.104
cg22372285	II	37	11	125951005		0.42	0.02991	1.52413	0.80225037	0.698154809	0.104
cg06823681	II	37	12	324682	SLC6A12	0.54	0.0014	2.85345	0.76175851	0.657619747	0.104
cg13186830	II	37	12	4141193		0.5	0.00432	2.36443	0.21914531	0.322769215	-0.104
cg01661897	II	37	12	4671207		0.54	0.0014	2.85345	0.30578952	0.409325489	-0.104
cg00470817	II	37	12	6741882	LPAR5;LPAR5	0.42	0.02991	1.52413	0.74802137	0.644001968	0.104
cg13575925	I	37	12	9217583	LOC144571	0.42	0.02991	1.52413	0.27963785	0.383326523	-0.104
cg06547285	II	37	12	47630263	FAM113B	0.42	0.02991	1.52413	0.75992621	0.864230893	-0.104
cg23539261	II	37	12	48228889		0.42	0.02991	1.52413	0.57648261	0.680643314	-0.104
cg12541478	II	37	12	52444020	NR4A1;NR4A1	0.46	0.01197	1.92207	0.76739567	0.663561784	0.104
cg01815626	II	37	12	52890212		0.46	0.01197	1.92207	0.61820956	0.513863216	0.104
cg01797169	II	37	12	107711489	BTBD11	0.54	0.0014	2.85345	0.38455554	0.488368722	-0.104
cg03884572	II	37	12	111521899	CUX2	0.54	0.0014	2.85345	0.57884198	0.474580236	0.104
cg14602393	II	37	12	133343405		0.42	0.02991	1.52413	0.56207291	0.665715016	-0.104
cg27152219	I	37	13	20139028		0.42	0.02991	1.52413	0.57538839	0.471540491	0.104
cg02192281	II	37	13	48478591		0.5	0.00432	2.36443	0.73926678	0.842996753	-0.104
cg07861790	I	37	13	112761844		0.46	0.01197	1.92207	0.51971656	0.623338775	-0.104
cg23013564	II	37	13	114786887	RASA3	0.54	0.0014	2.85345	0.83727943	0.733561447	0.104
cg24456744	II	37	13	114849719	RASA3	0.75	7.61E-07	6.11857	0.75683704	0.86069986	-0.104

cg18617527	I	37	14	100014679	CCDC85C	0.42	0.02991	1.52413	0.84273573	0.738719795	0.104
cg14245199	II	37	14	100540291	EVL	0.46	0.01197	1.92207	0.78938885	0.685026888	0.104
cg07452706	II	37	14	105649097	NUDT14	0.54	0.0014	2.85345	0.39224364	0.49596761	-0.104
cg19927100	II	37	15	68902085	CORO2B	0.5	0.00432	2.36443	0.71126454	0.606937	0.104
cg27294796	II	37	15	90456387	C15orf38	0.5	0.00432	2.36443	0.30269628	0.406293472	-0.104
cg06810647	II	37	16	1665094	CRAMP1L	0.54	0.0014	2.85345	0.10572637	0.209613192	-0.104
cg16454902	II	37	16	27414272	IL21R;IL21R	0.54	0.0014	2.85345	0.25142165	0.147796752	0.104
cg03427663	II	37	16	30445585		0.54	0.0014	2.85345	0.1095959	0.213547102	-0.104
cg02364831	II	37	16	52417584		0.5	0.00432	2.36443	0.41652017	0.312713941	0.104
cg17463287	II	37	16	71475335		0.5	0.00432	2.36443	0.43417479	0.537979066	-0.104
cg05918327	II	37	16	72955295	ZFHX3;ZFHX3	0.54	0.0014	2.85345	0.45574301	0.559416428	-0.104
cg27132152	II	37	16	88705016	IL17C;IL17C	0.42	0.02991	1.52413	0.35685803	0.252643376	0.104
cg24071064	II	37	16	88765233	RNF166	0.42	0.02991	1.52413	0.55824716	0.454328637	0.104
cg27299033	I	37	17	1883179	RTN4RL1	0.42	0.02991	1.52413	0.92121185	0.817307753	0.104
cg17580614	II	37	17	15849512	ADORA2B	0.42	0.02991	1.52413	0.6995099	0.803353184	-0.104
cg14270687	I	37	17	32953574	TMEM132E	0.42	0.02991	1.52413	0.25260388	0.356641846	-0.104
cg13443575	I	37	17	33775961	SLFN13	0.46	0.01197	1.92207	0.09793697	0.202270914	-0.104
cg02627216	II	37	17	38599056	IGFBP4	0.46	0.01197	1.92207	0.37301314	0.477085251	-0.104
cg00679763	II	37	17	46993161	UBE2Z	0.42	0.02991	1.52413	0.83373149	0.729566657	0.104
cg16723994	II	37	17	56401623	BZRAP1;BZRAP1	0.54	0.0014	2.85345	0.70712016	0.811329942	-0.104
cg10275770	II	37	17	62084205	ICAM2;ICAM2;ICAM	0.54	0.0014	2.85345	0.29845759	0.402198159	-0.104
cg27180671	II	37	17	65527566	PITPNC1;PITPNC1	0.46	0.01197	1.92207	0.79204382	0.688420383	0.104
cg04908905	II	37	17	71434049	SDK2	0.42	0.02991	1.52413	0.71343202	0.60984154	0.104
cg17529058	II	37	17	72886179	FADS6	0.5	0.00432	2.36443	0.75718228	0.653241583	0.104
cg07316730	II	37	17	73316721	GRB2;GRB2	0.46	0.01197	1.92207	0.81964249	0.716040454	0.104
cg04498014	II	37	17	76037250	TNRC6C;TNRC6C	0.46	0.01197	1.92207	0.46384156	0.567551682	-0.104
cg00249503	II	37	17	76565078	DNAH17	0.71	4.57E-06	5.34042	0.71338265	0.817176601	-0.104
cg22549408	II	37	18	57566300	PMAIP1	0.54	0.0014	2.85345	0.55237046	0.656097757	-0.104
cg06773488	II	37	18	74726002	MBP;MBP;MBP;MB	0.54	0.0014	2.85345	0.73647368	0.840814075	-0.104
cg10929690	II	37	18	77376689		0.46	0.01197	1.92207	0.59065157	0.694604683	-0.104
cg25806655	I	37	19	4543498	SEMA6B	0.42	0.02991	1.52413	0.54569404	0.649642442	-0.104
cg04512965	II	37	19	6038846	RFX2;RFX2	0.54	0.0014	2.85345	0.75534087	0.65124603	0.104
cg18530645	I	37	19	12759156	MAN2B1	0.67	2.34E-05	4.63072	0.63730273	0.741214158	-0.104

cg22625098	I	37	19	13054718	CALR	0.46	0.01197	1.92207	0.54161145	0.64581978	-0.104
cg24917775	I	37	19	46526675	PGLYRP1	0.46	0.01197	1.92207	0.26537847	0.369414796	-0.104
cg22312354	II	37	19	56158714	CCDC106	0.42	0.02991	1.52413	0.7381587	0.634196974	0.104
cg11546251	II	37	19	58322078	ZNF552	0.5	0.00432	2.36443	0.74694631	0.850988691	-0.104
cg10118435	II	37	20	390551	RBCK1;RBCK1	0.5	0.00432	2.36443	0.36991559	0.26597781	0.104
cg20993403	II	37	20	34700329	EPB41L1	0.42	0.02991	1.52413	0.78191341	0.677834107	0.104
cg01700462	II	37	20	45179230	C20orf123	0.5	0.00432	2.36443	0.20149576	0.305324982	-0.104
cg18523477	II	37	20	55967503	RBM38;RBM38	0.54	0.0014	2.85345	0.37997231	0.276210037	0.104
cg15004136	I	37	20	62687986	TCEA2	0.5	0.00432	2.36443	0.23002103	0.334185913	-0.104
cg23366234	I	37	21	45713704	AIRE;AIRE	0.62	0.0001	3.98291	0.93397187	0.829502164	0.104
cg03171478	II	37	22	37572916		0.67	2.34E-05	4.63072	0.12317043	0.227338488	-0.104
cg14153654	II	37	1	8001027	TNFRSF9	0.58	0.00041	3.39184	0.456683	0.353731601	0.103
cg07233952	II	37	1	22881200		0.5	0.00432	2.36443	0.52033659	0.62381534	-0.103
cg00576075	II	37	1	85725654	C1orf52;C1orf52	0.58	0.00041	3.39184	0.58931399	0.692647652	-0.103
cg16315020	II	37	1	109359175	AKNAD1	0.46	0.01197	1.92207	0.58934772	0.691912802	-0.103
cg06180389	II	37	1	111436823	CD53;CD53	0.46	0.01197	1.92207	0.62005416	0.517212931	0.103
cg09330596	II	37	1	151313760	RFX5;RFX5	0.46	0.01197	1.92207	0.74581199	0.848849201	-0.103
cg23092820	II	37	1	153329512	S100A9	0.42	0.02991	1.52413	0.59363361	0.696195451	-0.103
cg15723874	II	37	1	156457945	MEF2D	0.46	0.01197	1.92207	0.63992786	0.537205106	0.103
cg05937055	II	37	1	181128764		0.46	0.01197	1.92207	0.26051016	0.157068376	0.103
cg06875985	II	37	1	182572153	RGS16	0.46	0.01197	1.92207	0.78533041	0.682664161	0.103
cg19240637	II	37	2	7172297	RNF144A	0.5	0.00432	2.36443	0.62054433	0.723839755	-0.103
cg24351076	II	37	2	96992044	ITPRIPL1;ITPRIPL1;I	0.46	0.01197	1.92207	0.80380097	0.701053545	0.103
cg22931002	II	37	2	97541870	FAM178B;FAM178I	0.79	1.06E-07	6.97389	0.66360041	0.766923186	-0.103
cg11636504	II	37	2	99439883	C2orf55	0.58	0.00041	3.39184	0.45214789	0.555141037	-0.103
cg22016779	II	37	2	230452311	DNER	0.54	0.0014	2.85345	0.255293	0.152082599	0.103
cg10901633	II	37	3	4814459	ITPR1;ITPR1;ITPR1	0.42	0.02991	1.52413	0.66156519	0.764901479	-0.103
cg26692003	I	37	3	13063165	IQSEC1	0.5	0.00432	2.36443	0.53783501	0.434523274	0.103
cg01565013	II	37	3	46785957	PRSS45	0.46	0.01197	1.92207	0.74771254	0.644354857	0.103
cg15475502	II	37	3	122662340	SEMA5B	0.54	0.0014	2.85345	0.38690044	0.284170668	0.103
cg14549906	I	37	3	197500745	FYTTD1;FYTTD1;FYT	0.42	0.02991	1.52413	0.87542778	0.77213788	0.103
cg08029287	II	37	4	1503238		0.54	0.0014	2.85345	0.52145335	0.418358885	0.103
cg18883033	I	37	4	3742181		0.46	0.01197	1.92207	0.70227243	0.599270189	0.103

cg25970575	I	37	4	6010164		0.5	0.00432	2.36443	0.50583704	0.608399197	-0.103
cg08813325	II	37	4	7699611	SORCS2	0.5	0.00432	2.36443	0.34366403	0.446831662	-0.103
cg16581308	II	37	4	16862080	LDB2;LDB2	0.46	0.01197	1.92207	0.84707655	0.744525014	0.103
cg13184823	I	37	4	187476599	MTNR1A	0.46	0.01197	1.92207	0.05360807	0.156292518	-0.103
cg21558614	II	37	5	754184		0.54	0.0014	2.85345	0.69135996	0.588457454	0.103
cg21510348	II	37	5	72596752		0.42	0.02991	1.52413	0.26182085	0.364329538	-0.103
cg16549809	II	37	5	149821291		0.67	2.34E-05	4.63072	0.75425847	0.857435307	-0.103
cg18465082	II	37	5	176734745	MXD3;MXD3	0.58	0.00041	3.39184	0.52932291	0.631838764	-0.103
cg03910874	II	37	6	209712		0.54	0.0014	2.85345	0.53233264	0.429094911	0.103
cg16787652	II	37	6	29759955	HCG4	0.46	0.01197	1.92207	0.14303975	0.245910001	-0.103
cg06887580	II	37	6	31550699	LTB;LTB	0.42	0.02991	1.52413	0.76046243	0.657889011	0.103
cg25403205	II	37	6	31584215	AIF1;AIF1;AIF1	0.42	0.02991	1.52413	0.17118992	0.273900056	-0.103
cg04938317	I	37	6	32015737	TNXB	0.5	0.00432	2.36443	0.90133887	0.798515815	0.103
cg08331398	II	37	6	32808918	PSMB8;PSMB8	0.46	0.01197	1.92207	0.81222178	0.70944403	0.103
cg12241963	II	37	6	33807279		0.54	0.0014	2.85345	0.31700887	0.420079051	-0.103
cg07544796	I	37	6	36817048		0.46	0.01197	1.92207	0.18121478	0.283972683	-0.103
cg07541160	II	37	6	36985879	FGD2	0.54	0.0014	2.85345	0.76199162	0.659399231	0.103
cg00389341	I	37	6	37190260	TMEM217;TMEM2[illegible]	0.71	4.57E-06	5.34042	0.32904786	0.225668074	0.103
cg14422240	II	37	6	37425031	FTSJD2	0.58	0.00041	3.39184	0.79102882	0.89448058	-0.103
cg04652496	II	37	6	46294097	RCAN2	0.46	0.01197	1.92207	0.79715354	0.694529261	0.103
cg18192491	II	37	6	114384871	HS3ST5	0.46	0.01197	1.92207	0.73174276	0.629059964	0.103
cg03678062	I	37	6	149772716	ZC3H12D	0.5	0.00432	2.36443	0.51147149	0.614596999	-0.103
cg03209720	II	37	6	157727126	C6orf35	0.62	0.0001	3.98291	0.34564875	0.448585886	-0.103
cg25820728	I	37	7	1684706		0.46	0.01197	1.92207	0.91172008	0.809189369	0.103
cg11796827	I	37	7	6435978	RAC1;RAC1	0.54	0.0014	2.85345	0.27186014	0.375002029	-0.103
cg20720686	II	37	7	75582881	POR	0.5	0.00432	2.36443	0.75933563	0.861854811	-0.103
cg11860434	I	37	7	102067440		0.58	0.00041	3.39184	0.19409627	0.0914761	0.103
cg09507934	II	37	7	102072549	ORAI2;ORAI2	0.5	0.00432	2.36443	0.40738455	0.304143394	0.103
cg21253590	II	37	7	102073120	ORAI2;ORAI2	0.5	0.00432	2.36443	0.79480758	0.691430599	0.103
cg14938677	II	37	7	127231698	ARF5	0.5	0.00432	2.36443	0.62960157	0.732151713	-0.103
cg08360009	II	37	7	150433540	GIMAP5	0.58	0.00041	3.39184	0.80996376	0.707451367	0.103
cg00030005	II	37	7	155129409		0.54	0.0014	2.85345	0.84765746	0.74463386	0.103
cg09963024	II	37	8	6663226		0.46	0.01197	1.92207	0.80880972	0.706217183	0.103

cg14301348	I	37	8	20109523	LZTS1	0.5	0.00432	2.36443	0.80858197	0.705508911	0.103
cg21514735	II	37	8	61325952		0.46	0.01197	1.92207	0.55413016	0.6575036	-0.103
cg00831710	I	37	8	144240945	LY6H;LY6H;LY6H	0.42	0.02991	1.52413	0.26753156	0.370702225	-0.103
cg19405177	II	37	8	145001428	PLEC1;PLEC1;PLEC1	0.54	0.0014	2.85345	0.70231845	0.804955859	-0.103
cg07173635	II	37	9	971480		0.54	0.0014	2.85345	0.14867509	0.251642723	-0.103
cg14187724	II	37	9	93799798		0.54	0.0014	2.85345	0.14092823	0.244228563	-0.103
cg13781414	II	37	9	138951648	NACC2	0.62	0.0001	3.98291	0.6417613	0.744322615	-0.103
cg08100069	II	37	9	139014709		0.71	4.57E-06	5.34042	0.38309024	0.486313731	-0.103
cg17505469	II	37	10	8373450		0.46	0.01197	1.92207	0.80317572	0.700329788	0.103
cg04276417	II	37	10	33227909	ITGB1;ITGB1	0.62	0.0001	3.98291	0.36276731	0.465339351	-0.103
cg01839993	II	37	10	74034644	DDIT4	0.58	0.00041	3.39184	0.30665506	0.203859386	0.103
cg25965774	II	37	10	126841033	CTBP2;CTBP2	0.58	0.00041	3.39184	0.24053321	0.34364499	-0.103
cg08926253	II	37	11	614761	IRF7;IRF7;IRF7	0.46	0.01197	1.92207	0.56554948	0.668114689	-0.103
cg07096953	I	37	11	2154255	INS-IGF2;IGF2;IGF2;	0.5	0.00432	2.36443	0.32647978	0.223618252	0.103
cg11821865	II	37	11	2308511		0.5	0.00432	2.36443	0.18295685	0.286236903	-0.103
cg11201177	II	37	11	2961805		0.42	0.02991	1.52413	0.67582715	0.778330322	-0.103
cg05756220	II	37	11	30038685	KCNA4	0.5	0.00432	2.36443	0.14427149	0.247221511	-0.103
cg05445008	II	37	11	64811819	SAC3D1	0.58	0.00041	3.39184	0.71584503	0.819272832	-0.103
cg20887442	I	37	11	71725424	NUMA1	0.58	0.00041	3.39184	0.83894212	0.942381295	-0.103
cg13641591	II	37	11	75528534	UVRAG	0.58	0.00041	3.39184	0.71663669	0.613213536	0.103
cg10880163	II	37	11	116455589		0.62	0.0001	3.98291	0.47606023	0.373414225	0.103
cg06114171	II	37	11	117561139	DSCAML1	0.54	0.0014	2.85345	0.50080656	0.39796205	0.103
cg20483374	II	37	11	119211646	C1QTNF5;MFRP	0.46	0.01197	1.92207	0.30543093	0.408628582	-0.103
cg23079808	I	37	12	6493003	LTBR	0.46	0.01197	1.92207	0.12989383	0.233148273	-0.103
cg14199423	II	37	12	6721892		0.5	0.00432	2.36443	0.25646066	0.153323836	0.103
cg12580783	II	37	12	49362475	WNT10B	0.54	0.0014	2.85345	0.73308038	0.630508977	0.103
cg14242936	II	37	12	52404134	GRASP	0.58	0.00041	3.39184	0.36742904	0.470630002	-0.103
cg06015525	I	37	12	57872123	ARHGAP9;ARHGAP9	0.58	0.00041	3.39184	0.39283191	0.290163861	0.103
cg25027799	II	37	12	104659306	TXNRD1	0.79	1.06E-07	6.97389	0.66969743	0.772318065	-0.103
cg13150925	II	37	12	124529439		0.58	0.00041	3.39184	0.72518873	0.828423138	-0.103
cg07241090	II	37	12	124864594	NCOR2;NCOR2	0.54	0.0014	2.85345	0.32057609	0.423938116	-0.103
cg17387577	II	37	12	124864657	NCOR2;NCOR2	0.46	0.01197	1.92207	0.22492823	0.328298425	-0.103
cg21558509	I	37	12	125299800	SCARB1;SCARB1	0.58	0.00041	3.39184	0.82537675	0.928009015	-0.103

cg21703988	II	37	12	132549404	EP400	0.46	0.01197	1.92207	0.51439861	0.411652347	0.103
cg00760872	II	37	13	33514837		0.5	0.00432	2.36443	0.18410547	0.287313477	-0.103
cg08218799	II	37	14	24804930	ADCY4	0.58	0.00041	3.39184	0.56105874	0.664177822	-0.103
cg15456082	II	37	14	59444979		0.54	0.0014	2.85345	0.85681407	0.753924179	0.103
cg08015762	II	37	14	100014785	CCDC85C	0.54	0.0014	2.85345	0.83282167	0.729771558	0.103
cg26312888	II	37	14	101875604		0.42	0.02991	1.52413	0.69053552	0.587758832	0.103
cg10669129	II	37	15	25444152	SNORD115-16	0.58	0.00041	3.39184	0.79607975	0.693048757	0.103
cg16729415	I	37	15	35047203	GJD2	0.5	0.00432	2.36443	0.01684142	0.119604017	-0.103
cg25687358	I	37	15	45409942	DUOXA1;DUOXA2	0.5	0.00432	2.36443	0.15833577	0.261216822	-0.103
cg06836102	II	37	15	70744534		0.5	0.00432	2.36443	0.73272322	0.629607675	0.103
cg05975881	II	37	16	3148982		0.62	0.0001	3.98291	0.54016938	0.642886242	-0.103
cg03014241	II	37	16	11348611	SOCS1	0.5	0.00432	2.36443	0.66618366	0.563624	0.103
cg27040709	II	37	16	19191723	SYT17	0.5	0.00432	2.36443	0.68480899	0.581418879	0.103
cg23580000	II	37	16	50322156	ADCY7	0.5	0.00432	2.36443	0.67594062	0.778586557	-0.103
cg11323198	II	37	16	62069806	CDH8	0.5	0.00432	2.36443	0.13052249	0.233522285	-0.103
cg09276655	II	37	16	87735689	LOC100129637	0.42	0.02991	1.52413	0.6462854	0.543058218	0.103
cg07027457	I	37	16	89025522	CBFA2T3	0.62	0.0001	3.98291	0.82649387	0.92986704	-0.103
cg02948862	I	37	16	89035532	CBFA2T3	0.46	0.01197	1.92207	0.75109411	0.64803998	0.103
cg07835443	II	37	16	89734986	C16orf55	0.58	0.00041	3.39184	0.24372867	0.347159426	-0.103
cg10241823	II	37	17	841205	NXN	0.46	0.01197	1.92207	0.54566845	0.648372516	-0.103
cg18278729	II	37	17	1657676	SERPINF2;SERPINF2	0.62	0.0001	3.98291	0.37311265	0.475638777	-0.103
cg03161309	II	37	17	6563585		0.58	0.00041	3.39184	0.33179314	0.434697322	-0.103
cg01323777	I	37	17	7832943	KCNAB3	0.5	0.00432	2.36443	0.87985868	0.776369416	0.103
cg04460364	II	37	17	8370017	NDEL1;NDEL1	0.46	0.01197	1.92207	0.75456682	0.857544249	-0.103
cg22360318	II	37	17	10030599	GAS7	0.58	0.00041	3.39184	0.16294448	0.265872767	-0.103
cg20089715	II	37	17	37354769	CACNB1;CACNB1;CA	0.54	0.0014	2.85345	0.74544195	0.848153594	-0.103
cg22303227	II	37	17	41018992	LOC90586	0.5	0.00432	2.36443	0.73313932	0.630468742	0.103
cg05502349	II	37	17	41969698	MPP2	0.5	0.00432	2.36443	0.80329312	0.700347962	0.103
cg27410679	II	37	17	43866278	CRHR1;CRHR1;CRHI	0.54	0.0014	2.85345	0.67778971	0.575241758	0.103
cg11971423	II	37	17	46653711	HOXB4	0.54	0.0014	2.85345	0.70225299	0.805432293	-0.103
cg20590797	II	37	17	48253122	SGCA;SGCA	0.46	0.01197	1.92207	0.78995286	0.687156774	0.103
cg17319889	II	37	17	48683678	CACNA1G;CACNA1G	0.42	0.02991	1.52413	0.68090277	0.577490465	0.103
cg11061343	II	37	17	73993843	C17orf106	0.5	0.00432	2.36443	0.27561656	0.378373592	-0.103

cg05414903	II	37	17	76573574	DNAH17	0.42	0.02991	1.52413	0.40307707	0.299915158	0.103
cg05668703	II	37	17	79380493	BAHCC1	0.42	0.02991	1.52413	0.49372065	0.596911518	-0.103
cg08574915	I	37	17	79924772		0.46	0.01197	1.92207	0.12459726	0.22745081	-0.103
cg10460657	II	37	17	80560479	FOXK2	0.42	0.02991	1.52413	0.576512	0.679587402	-0.103
cg16071091	II	37	18	77196147	NFATC1;NFATC1;NF	0.58	0.00041	3.39184	0.69805704	0.59471192	0.103
cg19774868	I	37	18	77905408	LOC100130522;LOC	0.54	0.0014	2.85345	0.33811831	0.23521037	0.103
cg12170787	I	37	19	1130965	SBNO2;SBNO2	0.58	0.00041	3.39184	0.578172	0.475449619	0.103
cg12277416	II	37	19	2859153		0.54	0.0014	2.85345	0.64778498	0.750615647	-0.103
cg05350224	II	37	19	3053510	AES;AES;AES	0.5	0.00432	2.36443	0.57077263	0.467563704	0.103
cg14817241	II	37	19	4851575	PLIN3;PLIN3;PLIN3	0.42	0.02991	1.52413	0.62501016	0.521914904	0.103
cg24041556	II	37	19	10736059	SLC44A2;SLC44A2	0.42	0.02991	1.52413	0.58565432	0.688715946	-0.103
cg12928578	II	37	19	14890004	EMR2;EMR2;EMR2;	0.5	0.00432	2.36443	0.68793062	0.790942017	-0.103
cg23331421	I	37	19	30866202	ZNF536	0.5	0.00432	2.36443	0.08416263	0.187568641	-0.103
cg15430294	II	37	19	47125648	PTGIR	0.62	0.0001	3.98291	0.57941916	0.682761927	-0.103
cg00565090	II	37	19	48258258	SNORD23;GLTSCR2	0.58	0.00041	3.39184	0.49821954	0.601150389	-0.103
cg27427054	II	37	19	55859154	SUV420H2	0.5	0.00432	2.36443	0.71493611	0.611493255	0.103
cg08146323	II	37	19	57183118	ZNF835	0.58	0.00041	3.39184	0.11757351	0.220566724	-0.103
cg12493107	II	37	20	23344079	GZF1	0.42	0.02991	1.52413	0.71584189	0.613064031	0.103
cg06913219	II	37	20	25433878	NINL	0.54	0.0014	2.85345	0.67530757	0.777954241	-0.103
cg26553763	II	37	20	31352874	DNMT3B;DNMT3B;	0.5	0.00432	2.36443	0.42067786	0.523889917	-0.103
cg13279055	II	37	21	33789312	C21orf63	0.42	0.02991	1.52413	0.76629281	0.66311109	0.103
cg26646427	II	37	22	46770870	CELSR1	0.42	0.02991	1.52413	0.46944251	0.572930682	-0.103
cg18399427	II	37	22	50724844	PLXNB2	0.5	0.00432	2.36443	0.76194289	0.658549907	0.103
cg09864227	I	37	1	1008207		0.58	0.00041	3.39184	0.82669711	0.724967037	0.102
cg10888031	I	37	1	2938799	ACTRT2	0.54	0.0014	2.85345	0.88900664	0.786744229	0.102
cg08732466	II	37	1	2982592	FLJ42875;FLJ42875	0.5	0.00432	2.36443	0.76852409	0.666273746	0.102
cg14018153	I	37	1	3098864	PRDM16;PRDM16	0.42	0.02991	1.52413	0.17376014	0.275613565	-0.102
cg02954884	II	37	1	6638504	TAS1R1;TAS1R1;TA	0.5	0.00432	2.36443	0.53309556	0.431235695	0.102
cg06640822	II	37	1	25291472	RUNX3;RUNX3	0.67	2.34E-05	4.63072	0.58816937	0.486475743	0.102
cg22429121	II	37	1	27901805	AHDC1	0.46	0.01197	1.92207	0.36129075	0.463627173	-0.102
cg04219099	II	37	1	38261052	MANEAL;MANEAL;I	0.5	0.00432	2.36443	0.3734773	0.475316448	-0.102
cg11958827	II	37	1	44252126	ST3GAL3;ST3GAL3;S	0.54	0.0014	2.85345	0.80097288	0.903050892	-0.102
cg18231025	II	37	1	59573471		0.46	0.01197	1.92207	0.77203231	0.873842472	-0.102

cg07558455	II	37	1	62784762	KANK4	0.54	0.0014	2.85345	0.07925889	0.181648266	-0.102
cg22040158	II	37	1	112527540	KCND3;KCND3	0.58	0.00041	3.39184	0.55966429	0.45792721	0.102
cg09996284	II	37	1	156211851	BGLAP	0.46	0.01197	1.92207	0.24482348	0.346537085	-0.102
cg12662929	II	37	1	160368823		0.5	0.00432	2.36443	0.68663144	0.788464416	-0.102
cg09671955	II	37	1	169429972		0.5	0.00432	2.36443	0.45826441	0.560699539	-0.102
cg05378938	I	37	1	179560983	TDRD5	0.54	0.0014	2.85345	0.15175754	0.254099143	-0.102
cg01040850	II	37	1	181002669	MR1	0.54	0.0014	2.85345	0.82410546	0.722138105	0.102
cg06621080	II	37	1	201631166	NAV1	0.42	0.02991	1.52413	0.62970962	0.528135138	0.102
cg00964446	II	37	1	203023637	PPFIA4	0.5	0.00432	2.36443	0.77890573	0.677383428	0.102
cg03583857	II	37	1	208085022	CD34;CD34	0.46	0.01197	1.92207	0.29275599	0.39467727	-0.102
cg21934230	II	37	1	228202608	WNT3A	0.58	0.00041	3.39184	0.52250087	0.624703391	-0.102
cg09327855	II	37	1	236156587	NID1	0.42	0.02991	1.52413	0.68881163	0.587217368	0.102
cg00804354	II	37	1	249111407	SH3BP5L	0.42	0.02991	1.52413	0.2007246	0.303199998	-0.102
cg21047367	II	37	2	11732905	GREB1	0.42	0.02991	1.52413	0.73672009	0.838679842	-0.102
cg00270460	II	37	2	97509353	ANKRD23	0.54	0.0014	2.85345	0.56317574	0.665659903	-0.102
cg01114124	I	37	2	240171748	HDAC4	0.54	0.0014	2.85345	0.84233813	0.944346141	-0.102
cg26058474	I	37	2	241975035	SNED1	0.5	0.00432	2.36443	0.88310595	0.781053451	0.102
cg23733502	I	37	3	577629		0.5	0.00432	2.36443	0.71842239	0.820364496	-0.102
cg00472373	II	37	3	9833414	ARPC4;TADA3;ARP(	0.46	0.01197	1.92207	0.76611351	0.663774669	0.102
cg00625351	II	37	3	34048130		0.5	0.00432	2.36443	0.79802423	0.696262947	0.102
cg16947394	II	37	3	52930628	TMEM110	0.46	0.01197	1.92207	0.3275672	0.429887546	-0.102
cg07791011	II	37	3	62363922		0.58	0.00041	3.39184	0.33066754	0.432192398	-0.102
cg04816013	II	37	3	123987591	KALRN;KALRN	0.5	0.00432	2.36443	0.67694739	0.575015663	0.102
cg16510460	II	37	3	184434972		0.5	0.00432	2.36443	0.74152702	0.8430636	-0.102
cg14626309	II	37	4	299051		0.54	0.0014	2.85345	0.24816626	0.350291617	-0.102
cg27149937	II	37	4	640594	PDE6B;PDE6B	0.62	0.0001	3.98291	0.5237197	0.625625945	-0.102
cg11411884	I	37	4	1016333	FGFRL1;FGFRL1;FGI	0.42	0.02991	1.52413	0.83961324	0.737460114	0.102
cg08320703	II	37	4	2792582		0.5	0.00432	2.36443	0.53209795	0.63443917	-0.102
cg18912103	I	37	4	2944692	C4orf10;NOP14	0.46	0.01197	1.92207	0.60099872	0.703384896	-0.102
cg18630748	I	37	4	3387499	RGS12;RGS12;RGS1	0.5	0.00432	2.36443	0.73141657	0.833035808	-0.102
cg03835987	II	37	4	111120249	ELOVL6;ELOVL6	0.54	0.0014	2.85345	0.09163018	0.193972047	-0.102
cg19052164	II	37	5	80900		0.5	0.00432	2.36443	0.67172069	0.569867509	0.102
cg21946374	II	37	5	1108401	SLC12A7	0.5	0.00432	2.36443	0.29881584	0.40105168	-0.102

cg27346707	II	37	5	126626348	MEGF10	0.54	0.0014	2.85345	0.16919442	0.271077194	-0.102
cg16247269	II	37	5	148513941		0.54	0.0014	2.85345	0.63528902	0.533259118	0.102
cg09813400	II	37	5	150431636	TNIP1	0.54	0.0014	2.85345	0.55516054	0.65720313	-0.102
cg07802909	II	37	5	169817797	KCNMB1;KCNIP1	0.46	0.01197	1.92207	0.85852356	0.756833264	0.102
cg14830791	II	37	6	1407935		0.46	0.01197	1.92207	0.4010239	0.503208128	-0.102
cg00184953	II	37	6	31146222	PSORS1C3	0.46	0.01197	1.92207	0.81015926	0.708413041	0.102
cg22306579	I	37	6	31869057	ZBTB12	0.54	0.0014	2.85345	0.79161127	0.893585844	-0.102
cg16437896	I	37	6	32098346	FKBPL	0.54	0.0014	2.85345	0.36949414	0.267087376	0.102
cg03037684	II	37	6	152421333	ESR1;ESR1;ESR1;ES	0.46	0.01197	1.92207	0.52757547	0.629917827	-0.102
cg18698681	II	37	6	170571686	LOC154449	0.46	0.01197	1.92207	0.33704506	0.439450689	-0.102
cg01727625	II	37	7	35373		0.42	0.02991	1.52413	0.49448852	0.596366449	-0.102
cg16770054	II	37	7	1955738	MAD1L1;MAD1L1;M	0.46	0.01197	1.92207	0.76110658	0.863390737	-0.102
cg15997393	I	37	7	1961869	MAD1L1;MAD1L1;M	0.46	0.01197	1.92207	0.87538992	0.773678812	0.102
cg17890233	II	37	7	6465684	DAGLB;DAGLB	0.62	0.0001	3.98291	0.79416258	0.895857356	-0.102
cg06114556	II	37	7	47541543	TNS3	0.67	2.34E-05	4.63072	0.5455019	0.647146603	-0.102
cg06809544	II	37	7	79081666	MAGI2	0.5	0.00432	2.36443	0.19852562	0.300534532	-0.102
cg24531955	II	37	8	23154691	LOXL2	0.62	0.0001	3.98291	0.19260001	0.294569218	-0.102
cg13620439	II	37	8	27462513	CLU;CLU;CLU	0.42	0.02991	1.52413	0.8302912	0.728701506	0.102
cg04127342	II	37	8	57358130	PENK;PENK	0.58	0.00041	3.39184	0.15969885	0.261546654	-0.102
cg03335386	II	37	8	134469021	ST3GAL1;ST3GAL1	0.5	0.00432	2.36443	0.38058283	0.483050029	-0.102
cg08418332	II	37	9	34691001	CCL19	0.54	0.0014	2.85345	0.52294341	0.625157137	-0.102
cg13010497	II	37	9	38526947		0.46	0.01197	1.92207	0.62489384	0.726774958	-0.102
cg14113203	II	37	9	97346942	FBP2	0.58	0.00041	3.39184	0.79169704	0.689839741	0.102
cg14491667	II	37	9	100267701	TMOD1;TMOD1	0.5	0.00432	2.36443	0.81007857	0.707654682	0.102
cg00664581	II	37	9	115707717		0.54	0.0014	2.85345	0.66687601	0.564834086	0.102
cg14326196	I	37	9	116860650	KIF12	0.46	0.01197	1.92207	0.14315973	0.244870546	-0.102
cg25884399	II	37	9	139237461	GPSM1	0.42	0.02991	1.52413	0.23728518	0.338907324	-0.102
cg00407944	II	37	9	139424152	NOTCH1	0.46	0.01197	1.92207	0.21988715	0.321476099	-0.102
cg06223120	II	37	9	140022932		0.62	0.0001	3.98291	0.41310767	0.311309159	0.102
cg14568830	I	37	10	23463243		0.5	0.00432	2.36443	0.10003857	0.201846921	-0.102
cg14961117	II	37	10	71632008	COL13A1;COL13A1;	0.5	0.00432	2.36443	0.79050673	0.688450273	0.102
cg13836550	II	37	10	118084504	C10orf96	0.46	0.01197	1.92207	0.52308355	0.624957293	-0.102
cg00244001	II	37	10	126336805	FAM53B	0.5	0.00432	2.36443	0.21315584	0.31552556	-0.102

cg08380478	I	37	10	132683454		0.46	0.01197	1.92207	0.65150473	0.549549825	0.102
cg06376402	II	37	10	134404945	INPP5A	0.58	0.00041	3.39184	0.74141128	0.843822732	-0.102
cg25843174	II	37	11	12811716	TEAD1	0.46	0.01197	1.92207	0.63784716	0.535798345	0.102
cg23646614	II	37	11	48083100	PTPRJ;PTPRJ	0.54	0.0014	2.85345	0.19914166	0.301114597	-0.102
cg23208430	II	37	11	60483174	MS4A8B	0.46	0.01197	1.92207	0.74536714	0.643844258	0.102
cg23907108	II	37	11	64405993	NRXN2;NRXN2;NRX	0.42	0.02991	1.52413	0.40597398	0.303507648	0.102
cg24423806	II	37	11	66310467	ZDHHC24	0.58	0.00041	3.39184	0.74304392	0.845104337	-0.102
cg11921958	II	37	11	67068769	ANKRD13D;ANKRD1	0.46	0.01197	1.92207	0.57933594	0.680862682	-0.102
cg09545730	I	37	11	67071885	SSH3	0.46	0.01197	1.92207	0.62752936	0.729827119	-0.102
cg18160691	II	37	11	68081686	LRP5	0.67	2.34E-05	4.63072	0.2485405	0.351006677	-0.102
cg25101936	II	37	11	113929164	ZBTB16	0.62	0.0001	3.98291	0.6356912	0.738075185	-0.102
cg24621362	II	37	12	6492890	LTBR	0.67	2.34E-05	4.63072	0.12597237	0.228388116	-0.102
cg17718377	II	37	12	113495619	DTX1	0.5	0.00432	2.36443	0.57179305	0.469415891	0.102
cg17002771	I	37	13	19174841		0.5	0.00432	2.36443	0.50934991	0.40704823	0.102
cg17241776	I	37	13	51417469	DLEU7	0.46	0.01197	1.92207	0.07963179	0.181909873	-0.102
cg16404157	II	37	14	38724648	CLEC14A	0.42	0.02991	1.52413	0.10208145	0.204543612	-0.102
cg20496061	I	37	14	107210949		0.75	7.61E-07	6.11857	0.36962508	0.267401236	0.102
cg15744692	II	37	15	45671195	LOC145663;GATM	0.46	0.01197	1.92207	0.41374508	0.31169024	0.102
cg18022554	II	37	15	73657801	HCN4	0.58	0.00041	3.39184	0.61655093	0.71818309	-0.102
cg23481246	II	37	16	731772	STUB1;JMJD8	0.54	0.0014	2.85345	0.36768369	0.469979822	-0.102
cg06263943	II	37	16	1202015	CACNA1H;CACNA1H	0.46	0.01197	1.92207	0.46463388	0.362727641	0.102
cg07651316	I	37	16	3641320	BTBD12	0.58	0.00041	3.39184	0.87001518	0.971635934	-0.102
cg10908196	II	37	16	4732911	MGRN1;MGRN1;MG	0.62	0.0001	3.98291	0.67679465	0.779270541	-0.102
cg02525597	II	37	16	10976835	CIITA	0.5	0.00432	2.36443	0.8057869	0.704173146	0.102
cg26651122	II	37	16	56641777	MT2A	0.5	0.00432	2.36443	0.36266171	0.464284578	-0.102
cg26734350	I	37	16	57662008	GPR56;GPR56;GPR5	0.46	0.01197	1.92207	0.67843068	0.576907154	0.102
cg00620976	II	37	16	65612895		0.54	0.0014	2.85345	0.73733369	0.635520484	0.102
cg04760708	II	37	16	88988340	CBFA2T3;CBFA2T3	0.46	0.01197	1.92207	0.58649502	0.484586095	0.102
cg06419212	II	37	16	89161567	ACSF3;ACSF3;ACSF3	0.42	0.02991	1.52413	0.87396839	0.771896595	0.102
cg06934654	I	37	16	89180742	ACSF3;ACSF3;ACSF3	0.42	0.02991	1.52413	0.65034276	0.548764346	0.102
cg12655416	II	37	17	38077870	ORMDL3	0.46	0.01197	1.92207	0.26436152	0.36611405	-0.102
cg27470486	II	37	17	40073688	ACLY;ACLY	0.54	0.0014	2.85345	0.71334954	0.611781007	0.102
cg02108135	II	37	17	42017758		0.5	0.00432	2.36443	0.67006668	0.568144964	0.102

cg10575089	II	37	17	56606542	SEPT4;SEPT4;SEPT4	0.58	0.00041	3.39184	0.62819722	0.525939946	0.102
cg07433152	II	37	17	60782222	MARCH10;MARCH1	0.46	0.01197	1.92207	0.33646502	0.234043854	0.102
cg18271897	II	37	17	75316784	SEPT9;SEPT9;SEPT9	0.46	0.01197	1.92207	0.40131813	0.502874781	-0.102
cg22563987	I	37	17	76130496	TMC8	0.67	2.34E-05	4.63072	0.13602018	0.033636154	0.102
cg21357291	II	37	18	55471021		0.5	0.00432	2.36443	0.1800771	0.28161277	-0.102
cg15363134	I	37	18	77161214	NFATC1;NFATC1;NF	0.42	0.02991	1.52413	0.77111431	0.66870951	0.102
cg24377495	I	37	19	6660075		0.58	0.00041	3.39184	0.63963355	0.74184595	-0.102
cg05231098	I	37	19	11070915	SMARCA4;SMARCA	0.46	0.01197	1.92207	0.42761033	0.325940807	0.102
cg22041417	II	37	19	16178195	TPM4	0.42	0.02991	1.52413	0.63555026	0.737741845	-0.102
cg19462712	I	37	19	34744703	KIAA0355	0.5	0.00432	2.36443	0.79605113	0.69375872	0.102
cg02768785	II	37	19	45912979	CD3EAP;ERCC1;ERC	0.46	0.01197	1.92207	0.21388145	0.316309282	-0.102
cg11269599	II	37	19	52391304	ZNF577;ZNF577;ZN	0.46	0.01197	1.92207	0.22572537	0.327366672	-0.102
cg11267810	II	37	20	19867026		0.5	0.00432	2.36443	0.75518813	0.856890815	-0.102
cg01608731	II	37	20	30190720		0.46	0.01197	1.92207	0.79476826	0.692942148	0.102
cg14950044	II	37	20	49457327	BCAS4;BCAS4;BCAS	0.71	4.57E-06	5.34042	0.46074033	0.563077626	-0.102
cg06356785	II	37	21	45844767	TRPM2	0.42	0.02991	1.52413	0.8231353	0.721618324	0.102
cg05546044	II	37	22	22222597	MAPK1;MAPK1	0.54	0.0014	2.85345	0.24853294	0.146183519	0.102
cg07187268	II	37	22	24105186	C22orf15	0.54	0.0014	2.85345	0.47459884	0.57655548	-0.102
cg16166559	II	37	22	45072588	PRR5;PRR5;PRR5;PI	0.54	0.0014	2.85345	0.1297971	0.231857612	-0.102
cg06057569	II	37	22	50219754	BRD1	0.42	0.02991	1.52413	0.46769429	0.366148956	0.102
cg10219816	I	37	1	2427655	PLCH2	0.58	0.00041	3.39184	0.82235193	0.721269433	0.101
cg16022081	II	37	1	2564329	MMEL1;MMEL1	0.5	0.00432	2.36443	0.73124254	0.629836944	0.101
cg20014974	II	37	1	8271918		0.5	0.00432	2.36443	0.29333813	0.394329643	-0.101
cg22877366	II	37	1	12107668		0.5	0.00432	2.36443	0.43506498	0.334097488	0.101
cg14041921	II	37	1	15130959	KIAA1026;KIAA102(	0.54	0.0014	2.85345	0.80227328	0.701626366	0.101
cg21293216	II	37	1	24469768	IL22RA1	0.42	0.02991	1.52413	0.81687581	0.715864025	0.101
cg11254522	II	37	1	27950343	FGR;FGR;FGR	0.46	0.01197	1.92207	0.5307474	0.631337115	-0.101
cg25470758	I	37	1	31280143		0.54	0.0014	2.85345	0.68291543	0.581844759	0.101
cg22509164	II	37	1	46766957	LRRC41	0.58	0.00041	3.39184	0.46845858	0.569370401	-0.101
cg17167852	I	37	1	55505189	PCSK9	0.46	0.01197	1.92207	0.11825667	0.218793412	-0.101
cg23277715	II	37	1	153330068	S100A9	0.46	0.01197	1.92207	0.14200346	0.243001009	-0.101
cg22700686	II	37	1	153538764	S100A2	0.42	0.02991	1.52413	0.14938226	0.250554658	-0.101
cg26230851	II	37	1	161231273	PCP4L1	0.5	0.00432	2.36443	0.81003538	0.708785158	0.101

cg15492834	II	37	1	162351057	C1orf226;C1orf226	0.5	0.00432	2.36443	0.84347875	0.742367578	0.101
cg17756730	II	37	1	172608644		0.46	0.01197	1.92207	0.66729261	0.566066695	0.101
cg06644515	II	37	1	173834831	SNORD47;GAS5;SN[illegible]	0.46	0.01197	1.92207	0.64233387	0.541385291	0.101
cg16419354	I	37	1	179713349	FAM163A	0.46	0.01197	1.92207	0.13419261	0.235582346	-0.101
cg22622057	II	37	1	182053203		0.42	0.02991	1.52413	0.25113556	0.351644798	-0.101
cg24315209	II	37	1	205494299	CDK18;CDK18;CDK[illegible]	0.5	0.00432	2.36443	0.6029419	0.502098209	0.101
cg06852461	II	37	1	207975182		0.42	0.02991	1.52413	0.17329895	0.274784312	-0.101
cg24371574	II	37	1	230428167		0.5	0.00432	2.36443	0.65237712	0.551004565	0.101
cg04415297	II	37	1	231243034		0.42	0.02991	1.52413	0.71715342	0.615761286	0.101
cg06523516	I	37	2	794646		0.46	0.01197	1.92207	0.64365482	0.543048314	0.101
cg07628841	I	37	2	27851430	GPN1;CCDC121;GP[illegible]	0.54	0.0014	2.85345	0.41741544	0.316203391	0.101
cg13820899	II	37	2	42991343	OXER1;OXER1	0.62	0.0001	3.98291	0.20903691	0.310487131	-0.101
cg06949933	II	37	2	61406491	AHSA2	0.42	0.02991	1.52413	0.70518003	0.604168988	0.101
cg22737154	II	37	2	64631614		0.54	0.0014	2.85345	0.5240746	0.624766925	-0.101
cg01271812	II	37	2	66671478	MEIS1	0.54	0.0014	2.85345	0.20632205	0.307182344	-0.101
cg25299364	II	37	2	68882144	PROKR1	0.54	0.0014	2.85345	0.68138755	0.78261322	-0.101
cg06869641	II	37	2	71947929		0.62	0.0001	3.98291	0.59451111	0.493829612	0.101
cg04670857	II	37	2	79740208	CTNNA2;CTNNA2;C[illegible]	0.42	0.02991	1.52413	0.15483106	0.255529677	-0.101
cg13176012	II	37	2	157176971		0.46	0.01197	1.92207	0.20470572	0.30600644	-0.101
cg02915785	II	37	2	219233544		0.46	0.01197	1.92207	0.32843855	0.429196672	-0.101
cg16360836	II	37	2	240207606	HDAC4	0.42	0.02991	1.52413	0.36743779	0.468315289	-0.101
cg08440077	II	37	2	241854588		0.58	0.00041	3.39184	0.71931431	0.618198914	0.101
cg13339454	II	37	2	242127690	ANO7;ANO7	0.42	0.02991	1.52413	0.79896104	0.69759221	0.101
cg02914097	II	37	3	31118948		0.46	0.01197	1.92207	0.47253952	0.573254876	-0.101
cg24436196	II	37	3	56448270	ERC2	0.42	0.02991	1.52413	0.65908631	0.557816139	0.101
cg04481170	II	37	3	158445995	RARRES1;RARRES1	0.42	0.02991	1.52413	0.42282469	0.524128969	-0.101
cg17574471	I	37	4	1834678	LETM1	0.5	0.00432	2.36443	0.8300252	0.72910023	0.101
cg22077313	II	37	4	6691093		0.42	0.02991	1.52413	0.4739811	0.575450763	-0.101
cg11624060	II	37	5	1316038		0.58	0.00041	3.39184	0.17861855	0.077242314	0.101
cg26468696	II	37	5	141331010	PCDH12	0.42	0.02991	1.52413	0.47929446	0.378129303	0.101
cg04709822	I	37	5	149823868	RPS14;RPS14;RPS1[illegible]	0.42	0.02991	1.52413	0.61447404	0.71569583	-0.101
cg10981651	I	37	5	153853545		0.42	0.02991	1.52413	0.14322329	0.244377743	-0.101
cg19537558	II	37	5	167550996	ODZ2	0.58	0.00041	3.39184	0.88617428	0.785524399	0.101

cg20952257	II	37	5	171074407		0.5	0.00432	2.36443	0.3772415	0.478293563	-0.101
cg08815340	II	37	6	5026435		0.5	0.00432	2.36443	0.15449096	0.255015991	-0.101
cg21644740	I	37	6	29599248	GABBR1;GABBR1	0.46	0.01197	1.92207	0.58588499	0.686792624	-0.101
cg02188225	I	37	6	30459255	HLA-E	0.46	0.01197	1.92207	0.7478678	0.646371709	0.101
cg19215110	II	37	6	30850913	DDR1;DDR1	0.42	0.02991	1.52413	0.76016105	0.659482313	0.101
cg27198497	I	37	6	33870701		0.58	0.00041	3.39184	0.79194253	0.892789638	-0.101
cg07699454	II	37	6	39274353	KCNK17;KCNK17	0.5	0.00432	2.36443	0.67650298	0.575898246	0.101
cg16540391	II	37	6	151042035	PLEKHG1	0.42	0.02991	1.52413	0.18311872	0.08186647	0.101
cg12816748	II	37	7	752743	PRKAR1B;PRKAR1B	0.58	0.00041	3.39184	0.22138974	0.322738488	-0.101
cg05861879	II	37	7	2284723	NUDT1;NUDT1;NUI	0.5	0.00432	2.36443	0.51936406	0.418547821	0.101
cg01492538	II	37	7	2774543	GNA12	0.5	0.00432	2.36443	0.22757084	0.32809749	-0.101
cg18833928	II	37	7	38357684		0.46	0.01197	1.92207	0.67852059	0.77981773	-0.101
cg19465268	I	37	7	75517941	RHBDD2;RHBDD2	0.58	0.00041	3.39184	0.80902552	0.910137688	-0.101
cg11202871	II	37	7	134851177	C7orf49;C7orf49;C7	0.54	0.0014	2.85345	0.58460346	0.685937258	-0.101
cg23896514	II	37	7	150103902	LOC728743	0.54	0.0014	2.85345	0.1572383	0.056574229	0.101
cg24842334	II	37	7	157647224	PTPRN2;PTPRN2;PT	0.54	0.0014	2.85345	0.22306822	0.323843119	-0.101
cg11548083	II	37	8	10208156	MSRA;MSRA;MSRA	0.5	0.00432	2.36443	0.46195716	0.562707512	-0.101
cg24387126	II	37	8	11617293	GATA4	0.46	0.01197	1.92207	0.48962057	0.591077278	-0.101
cg12271419	II	37	8	22855616	RHOBTB2;RHOBTB2	0.42	0.02991	1.52413	0.43574317	0.33435455	0.101
cg25922329	II	37	8	128911766	PVT1	0.54	0.0014	2.85345	0.65731277	0.75846993	-0.101
cg11547696	II	37	8	142288508		0.5	0.00432	2.36443	0.49895774	0.600286365	-0.101
cg20100987	II	37	8	143868262	LY6D	0.54	0.0014	2.85345	0.4249512	0.525712692	-0.101
cg10061906	I	37	8	146013430	ZNF34	0.42	0.02991	1.52413	0.75687638	0.655975111	0.101
cg17558772	II	37	10	1406218	ADARB2	0.54	0.0014	2.85345	0.57501341	0.676366065	-0.101
cg18264092	II	37	10	6183575		0.46	0.01197	1.92207	0.79871914	0.697456185	0.101
cg09389824	II	37	10	31883099		0.5	0.00432	2.36443	0.78928773	0.688178225	0.101
cg08514530	II	37	10	44163270		0.42	0.02991	1.52413	0.50341583	0.604508657	-0.101
cg27074971	II	37	10	71072149	HK1;HK1;HK1	0.42	0.02991	1.52413	0.74435633	0.844886338	-0.101
cg13630239	II	37	10	99116547	RRP12;RRP12	0.46	0.01197	1.92207	0.62138925	0.722258608	-0.101
cg11228197	I	37	11	708640	EPS8L2	0.71	4.57E-06	5.34042	0.37049599	0.269977385	0.101
cg02556649	I	37	11	1945564	TNNT3;TNNT3;TNN	0.42	0.02991	1.52413	0.85346236	0.752841319	0.101
cg23162201	I	37	11	8280741	LMO1	0.54	0.0014	2.85345	0.70339584	0.804283969	-0.101
cg09767822	I	37	11	20178040	DBX1	0.54	0.0014	2.85345	0.31797948	0.418513905	-0.101

cg07438103	II	37	11	47175143	C11orf49;C11orf49	0.58	0.00041	3.39184	0.60640592	0.505262103	0.101
cg22705929	II	37	11	57417974	YPEL4	0.58	0.00041	3.39184	0.49688093	0.597824075	-0.101
cg25835179	II	37	11	67418291	ACY3	0.46	0.01197	1.92207	0.32109887	0.220261063	0.101
cg19787556	I	37	12	34514461		0.46	0.01197	1.92207	0.48277694	0.382257925	0.101
cg22193385	II	37	12	52638005	KRT7	0.5	0.00432	2.36443	0.16803793	0.268917436	-0.101
cg18689332	II	37	12	114837666	TBX5;TBX5;TBX5;TE	0.58	0.00041	3.39184	0.47674612	0.578070727	-0.101
cg14094027	II	37	12	120669080	PXN;PXN;PXN	0.71	4.57E-06	5.34042	0.37813283	0.478737282	-0.101
cg23110109	II	37	12	122092127	MORN3	0.42	0.02991	1.52413	0.22472276	0.325357254	-0.101
cg11147471	I	37	12	125087686		0.46	0.01197	1.92207	0.44445259	0.545857602	-0.101
cg05514909	II	37	12	133022853		0.46	0.01197	1.92207	0.6212174	0.722294553	-0.101
cg05868813	II	37	13	26594483	ATP8A2	0.5	0.00432	2.36443	0.3258231	0.426851667	-0.101
cg12861503	II	37	13	52734071	NEK3;NEK3;NEK3;N	0.46	0.01197	1.92207	0.41231023	0.513333042	-0.101
cg01421309	II	37	13	114084041	ADPRHL1;ADPRHL1	0.58	0.00041	3.39184	0.35260556	0.453960667	-0.101
cg16512163	II	37	13	114888551	RASA3	0.42	0.02991	1.52413	0.74868118	0.849801457	-0.101
cg03526920	II	37	14	77291706	C14orf166B	0.42	0.02991	1.52413	0.79444726	0.693601019	0.101
cg02220617	II	37	14	102312533	PPP2R5C;PPP2R5C;	0.46	0.01197	1.92207	0.7840077	0.683047812	0.101
cg15457934	I	37	14	104652835		0.62	0.0001	3.98291	0.93716292	0.836569893	0.101
cg06861988	II	37	15	25319513		0.54	0.0014	2.85345	0.62498927	0.726201476	-0.101
cg18738367	II	37	15	79238723	CTSH;CTSH	0.42	0.02991	1.52413	0.13306824	0.233907268	-0.101
cg09830866	I	37	16	771714	FAM173A	0.62	0.0001	3.98291	0.51824798	0.417419575	0.101
cg16524242	I	37	16	1538355	C16orf38	0.5	0.00432	2.36443	0.57836931	0.679501253	-0.101
cg00809164	I	37	16	56651049	MT1L	0.46	0.01197	1.92207	0.23855634	0.339094268	-0.101
cg09317371	I	37	16	57180175	CPNE2	0.5	0.00432	2.36443	0.82061894	0.921346539	-0.101
cg04250732	II	37	16	57391655	CCL22	0.42	0.02991	1.52413	0.60623769	0.70722538	-0.101
cg08310837	II	37	16	67572860	FAM65A	0.46	0.01197	1.92207	0.27928213	0.380203704	-0.101
cg27262054	I	37	16	73067525	ZFHX3;ZFHX3	0.46	0.01197	1.92207	0.64948257	0.548518509	0.101
cg13391235	II	37	16	85316611		0.62	0.0001	3.98291	0.64753698	0.54660631	0.101
cg21491107	II	37	16	85649026	KIAA0182;KIAA018:	0.58	0.00041	3.39184	0.36252505	0.463112811	-0.101
cg04267828	II	37	16	85741460	C16orf74	0.46	0.01197	1.92207	0.81089734	0.710092359	0.101
cg01810713	II	37	16	85966031		0.46	0.01197	1.92207	0.74707628	0.646325362	0.101
cg08161325	I	37	16	88122741		0.71	4.57E-06	5.34042	0.8502953	0.951610261	-0.101
cg06647693	I	37	16	88590340	ZFPM1	0.5	0.00432	2.36443	0.56536083	0.666225482	-0.101
cg01366670	II	37	16	89004378	CBFA2T3;CBFA2T3	0.58	0.00041	3.39184	0.42799004	0.529350336	-0.101

cg05231226	II	37	16	89171772	ACSF3;ACSF3;ACSF3	0.5	0.00432	2.36443	0.72268142	0.622147288	0.101
cg20972466	II	37	16	89679595	DPEP1	0.42	0.02991	1.52413	0.32050792	0.219017756	0.101
cg26771557	II	37	17	16955261	MPRIP;MPRIP	0.62	0.0001	3.98291	0.33279203	0.433845806	-0.101
cg05114606	II	37	17	21274193		0.46	0.01197	1.92207	0.32809533	0.429329612	-0.101
cg01809214	II	37	17	39781055	KRT17	0.5	0.00432	2.36443	0.46546829	0.566133334	-0.101
cg25109721	II	37	17	48125064		0.5	0.00432	2.36443	0.26803725	0.368882158	-0.101
cg00443981	I	37	17	58499679	C17orf64	0.5	0.00432	2.36443	0.84373281	0.944488035	-0.101
cg22122808	II	37	17	61511683	CYB561;CYB561;CY	0.46	0.01197	1.92207	0.25622521	0.357171413	-0.101
cg12884495	II	37	17	73842017	WBP2;UNC13D	0.58	0.00041	3.39184	0.7370206	0.838491214	-0.101
cg23601515	II	37	17	74017805	EVPL	0.5	0.00432	2.36443	0.58959247	0.690521879	-0.101
cg10584478	I	37	17	79422530	BAHCC1	0.46	0.01197	1.92207	0.80121541	0.901863473	-0.101
cg15982419	II	37	18	9474707	RALBP1	0.46	0.01197	1.92207	0.41905481	0.520223956	-0.101
cg00266920	II	37	18	55021277	ST8SIA3	0.54	0.0014	2.85345	0.18510644	0.286300033	-0.101
cg19165390	II	37	18	56932082		0.58	0.00041	3.39184	0.28431107	0.385732972	-0.101
cg25697727	I	37	19	291986	PPAP2C;PPAP2C;PP	0.58	0.00041	3.39184	0.65539378	0.756542925	-0.101
cg21586152	I	37	19	853540	ELANE	0.42	0.02991	1.52413	0.54123863	0.642311732	-0.101
cg04340258	I	37	19	3398706	NFIC;NFIC	0.58	0.00041	3.39184	0.70081033	0.801932358	-0.101
cg05718253	II	37	19	11347359	LOC55908;DOCK6	0.42	0.02991	1.52413	0.65807324	0.759443528	-0.101
cg14366490	II	37	19	17571825	NXNL1	0.46	0.01197	1.92207	0.73096875	0.630368794	0.101
cg24091474	II	37	19	36399185	TYROBP;TYROBP;TY	0.46	0.01197	1.92207	0.17491088	0.275906994	-0.101
cg17110586	II	37	19	36454623		0.58	0.00041	3.39184	0.29280365	0.393885795	-0.101
cg05896295	II	37	19	41812492	HNRNPUL1;HNRNP	0.42	0.02991	1.52413	0.31503041	0.415739325	-0.101
cg25528786	II	37	19	42082267	CEACAM21;CEACAM	0.46	0.01197	1.92207	0.25311446	0.353743861	-0.101
cg14511156	II	37	19	54604124	OSCAR;OSCAR;OSCA	0.62	0.0001	3.98291	0.27864938	0.379408084	-0.101
cg15892280	II	37	21	40180000	ETS2	0.46	0.01197	1.92207	0.26606054	0.367020995	-0.101
cg15245051	II	37	22	36563117	APOL3;APOL3	0.42	0.02991	1.52413	0.38123842	0.482112125	-0.101
cg04931184	II	37	22	38840560	KCNJ4;KCNJ4	0.46	0.01197	1.92207	0.59724501	0.496074365	0.101
cg16154810	II	37	22	47135258	CERK	0.5	0.00432	2.36443	0.42172355	0.522518641	-0.101
cg07596065	II	37	22	50984393		0.42	0.02991	1.52413	0.57969189	0.680885298	-0.101
					1869					hypomethylat	4731
					5749					hypermethylat	2887
										total	7618
